# [1,3]/[1,4]-Sulfur atom migration in β-hydroxyalkylphosphine sulfides

**DOI:** 10.3762/bjoc.16.11

**Published:** 2020-01-21

**Authors:** Katarzyna Włodarczyk, Piotr Borowski, Marek Stankevič

**Affiliations:** 1 Department of Organic Chemistry, Faculty of Chemistry, Marie Curie-Skłodowska University in Lublin, 33 Gliniana St., 20-614 Lublin, Poland; 2 Department of Chromatographic Methods, Faculty of Chemistry, Marie Curie-Skłodowska University in Lublin, 3 Marie Curie-Skłodowska Sq., 20-031 Lublin, Poland

**Keywords:** Brønsted and Lewis acids, DFT, mechanistic studies, rearrangement, stereochemistry, sulfur atom migration

## Abstract

β-Hydroxyalkylphosphine sulfides undergo [1,3]- or [1,4]-sulfur atom phosphorus-to-carbon migration in the presence of Lewis or Brønsted acids. The direction of sulfur atom migration depends on the type of acid used for the reaction. In the presence of a Brønsted acid, mainly [1,3]-rearrangement is observed, whereas a Lewis acid catalyzes the [1,4]-sulfur migration. To gain insight into the mechanism of these transformations, the stereochemistry of these rearrangements have been tested, along with the conduction of some control experiments and DFT calculations.

## Introduction

**Scheme 1:**
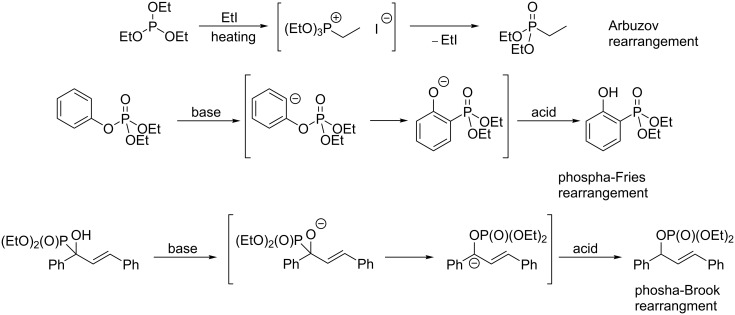
Arbusov, phospha-Fries, and phospha-Brook rearrangements.

Among all organic reactions, rearrangements are an exciting class of transformations where unusual or even unexpected products can be obtained. Many rearrangements are of practical use in synthetic organic chemistry, including Beckmann [1–4], Claisen [5–8], pinacol [9–12], Wagner–Meerwein [13–16], Curtius [17–20], Hofmann [21–24], Overmann [25–28] rearrangements, and many others. In organophosphorus chemistry, rearrangements are less developed transformations, however, some examples can be found in the literature (Scheme 1). These include the famous Arbuzov rearrangement [29–32] as well as phospha-Fries ([1,3]-rearrangements) [33–37], phospha-Brook ([1,2]-rearrangement) [38–42], and [2,3]-sigmatropic rearrangements of propargyl and allylphosphinites [43–46].

Other [1,2]-rearrangements include α,β-epoxyphosphonate opening [47], β-halo-α-hydroxyphosphonate rearrangement [48], phosphirene rearrangement [49], or the unusual phosphonate–phosphinate rearrangement of dimethyl phosphonates [50]. Other [1,3]-rearrangements of organophosphorus compounds include phosphoenolpyruvate formation from phosphonopyruvate [51], benzylphosphonium salt formation from the corresponding *o*-methylaryl-substituted precursors [52], or the formation of ketophosphonates from vinylphosphates [53]. [1,4]-Rearrangements include that of *o*-phosphorus-substituted benzyl carbanions [34], phosphorus group migration in *O*-phosphorylated 1,4-benzodiazepines [54], or phosphoryl group carbon-to-oxygen transfer [55]. The common feature of every rearrangement presented above is the cleavage of one single bond between phosphorus and either carbon or oxygen atom, while the multiple bond remains intact.

Herein, we present the results concerning an unusual transformation of β-hydroxyalkylphosphine sulfides, which undergo [1,3]- or [1,4]-rearrangement in the presence of an acid, yielding the corresponding β/γ-mercaptoalkylphosphine oxides. In this rearrangement, a sulfur atom transfers from phosphorus to carbon, whereas the phosphorus–carbon bonds remain intact. Depending on the type of acid, sulfur atom migration may occur at the β- or the γ-carbon atom.

## Results and Discussion

**Scheme 2:**
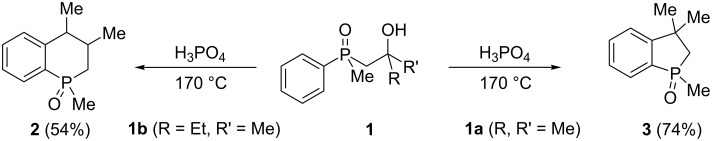
Cyclization of **1a** and **1b** under acidic conditions.

In our previous papers [56–57], we described the intramolecular cationic cyclization of β-hydroxyalkylphosphine oxides (Scheme 2).

**Scheme 3:**
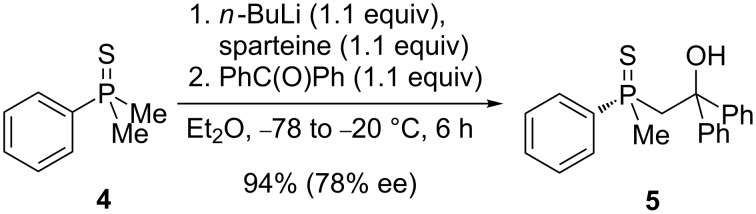
The synthesis of P-stereogenic β-hydroxyalkylphosphine sulfides.

Depending on the structure, the formation of either the phosphaindane **3** or the benzophosphorinane **2** skeleton was observed. This method could also be performed in a stereoselective manner, given that corresponding chiral substrates were used for the cyclization. The chiral compounds suitable for cyclization could be obtained by desymmetrization of phosphine sulfides (Scheme 3) [58].

In order to gain insight into the cationic cyclization of β-hydroxyalkylphosphine sulfides, a set of substrates **6**–**25** was prepared from dimethylphenylphosphine sulfide **4** (Table 1).

**Table 1:** Synthesis of substrates for this study.

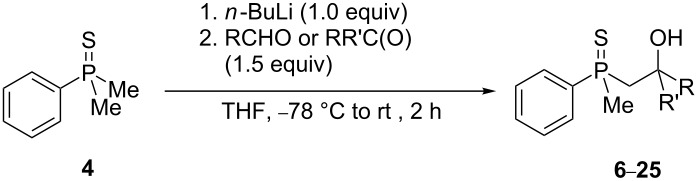						

entry	R		R^’^	compound	yield, %	dr

1	Me		H	**6**	77	51:49
2	Et		H	**7**	90	57:43
3	iPr		H	**8**	75	55:45
4	*t*-Bu		H	**9**	84	61.5:38.5
5	Ph		H	**10**	71	60:40
6	cyclohexane		H	**11**	89	57:43
7	Et		Me	**12**	91	52:48
8	Ph		Me	**13**	92	62.5:37.5
9	*n*-Bu		Me	**14**	76	58:42
10	*n*-Pr		Me	**15**	84	51:49
11	iPr		Me	**16**	85	53:47
12	*t*-Bu		Me	**17**	84	55:45
13	*t*-BuCH_2_		Me	**18**	86	53:47
14	Me		Me	**19**	88	
15	Et		Et	**20**	78	
16	iPr		iPr	**21**	70	
17		–(CH_2_)_4_–		**22**	72	
18		–(CH_2_)_5_–		**23**	88	
19		–(CH_2_)_6_–		**24**	88	
20	*n*-Bu		*n*-Bu	**25**	78	

All compounds were obtained in good yields, both from aldehydes and ketones. For aldehydes and unsymmetrically substituted ketones, the formation of the products as diastereomeric mixtures could be observed. Unfortunately, the diastereomeric excess of the reaction was low in each case.

**Scheme 4:**
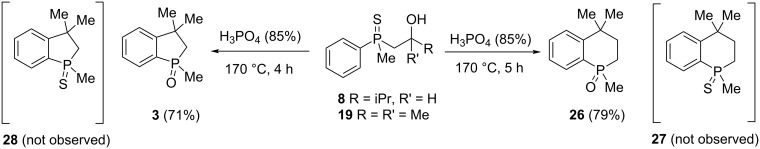
Cyclization of **8** and **19** in the presence of H_3_PO_4_.

In order to analyze the reactivity of β-hydroxyalkylphosphine sulfides under acidic conditions, the cyclization of **8** and **19** was attempted (Scheme 4).

**Scheme 5:**
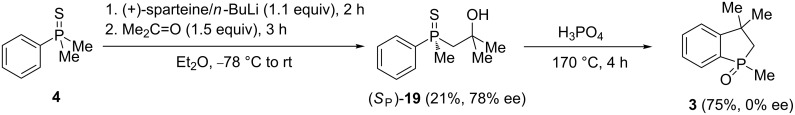
Cyclization of (*S*_P_)**-19** in the presence of H_3_PO_4_.

Unexpectedly, when the reaction was performed under conditions developed originally for β-hydroxyalkylphosphine oxides [56–57], this led to the formation of cyclic phosphine oxides **3** and **26** rather than phosphine sulfides. It may be assumed that hydrolysis of the P=S bond occurred under these reaction conditions and, probably, racemization of the phosphorus center in the case of nonracemic β-hydroxyalkylphosphine sulfides. Indeed, the attempted cyclization of (*S*_P_)**-19** under standard reaction conditions led to the formation of the completely racemic phosphine oxide **3** (Scheme 5).

The above results clearly show that phosphoric acid was inappropriate for the cyclization of nonracemic β-hydroxyalkylphosphine sulfides, and therefore, an alternative methodology had to be developed for the synthesis of chiral organophosphorus compounds possessing either phosphaindane or benzophosphorinane skeletons. As such, it was decided to test the ability of Lewis acids to catalyze the cyclization of β-hydroxyalkylphosphine sulfides (Table 2).

**Table 2:** Reaction of β-hydroxyalkylphosphine sulfides with Lewis-acidic AlCl_3_.

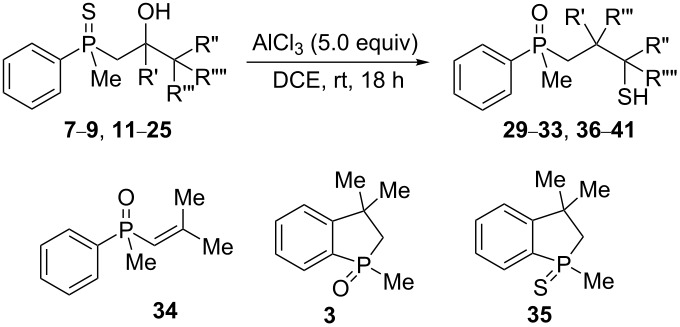		

entry	substrate	product (yield)

1	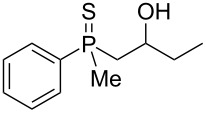 **7**	no reaction
2	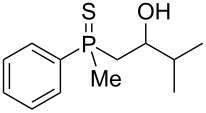 **8**	no reaction
3	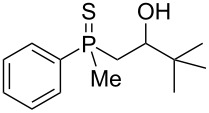 **9**	no reaction
4	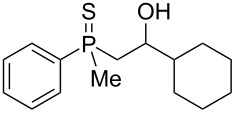 **11**	no reaction
5	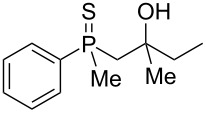 **12**	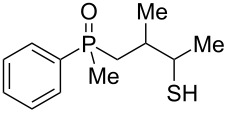 **29** (74%)
6	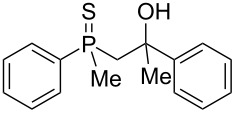 **13**	mixture
7	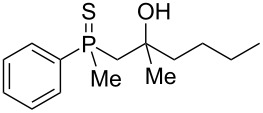 **14**	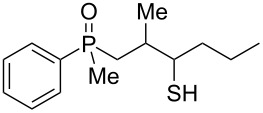 **30** (94%)
8	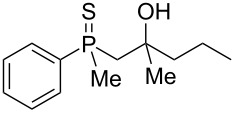 **15**	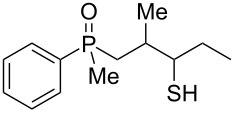 **31** (81%)
9	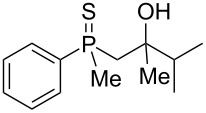 **16**	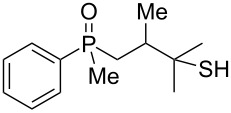 **32** (74%)
10	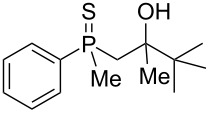 **17**	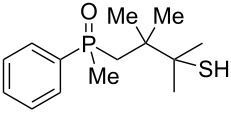 **33** (74%)
11	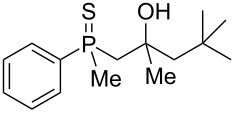 **18**	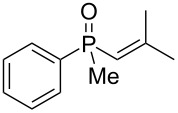 **34** (96%)
12	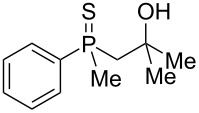 **19**	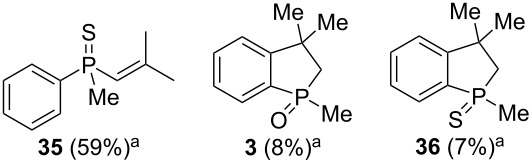
13	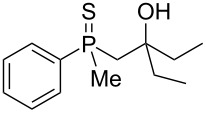 **20**	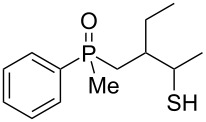 **37** (59%)
14	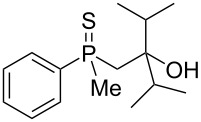 **21**	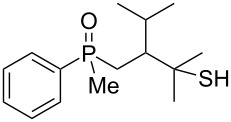 **38** (59%)
15	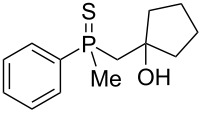 **22**	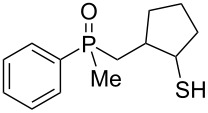 **39** (40%)
16	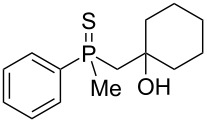 **23**	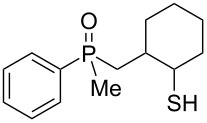 **40** (60%)
17	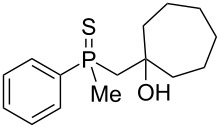 **24**	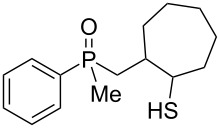 **41** (88%)
18	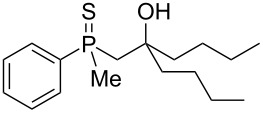 **25**	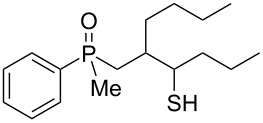 **42** (60%)

^a^Conversion based on ^31^P NMR spectroscopic analysis of the crude mixture.

As it turned out, the sulfides derived from aldehydes **7–9** and **11** failed to undergo a rearrangement (Table 2, entries 1–4). On the other hand, compounds derived from ketones **12**, **14**–**17**, and **20**–**25** underwent a ‘clean’ reaction under the conditions. However, instead of cyclization, a phosphorus-to-carbon sulfur migration took place, affording the corresponding mercaptoalkylphosphine oxides **29–33** and **37–42** with satisfying yields (Table 2, entries 5, 7–10, and 13–18). This sulfur atom phosphorus-to-carbon migration in phosphine sulfides has previously been mentioned only once in the literature [59]. Another example of such a migration has been reported by Creary and Innocencio for functionalized α-hydroxythiophosphonic acid diesters [60].

What is more interesting, the sulfur atom did not simply replace oxygen at the β-carbon atom: in every case, the mercapto group was placed at the γ-carbon atom, suggesting that the reaction followed a more complex mechanism. All reactions tested proceeded with the formation of an additional chirality center at the γ-carbon atom in the alkyl substituent, and therefore, almost all products were obtained as mixtures of epimers. Interestingly, compound **18** underwent isobutene elimination rather than rearrangement under the reaction conditions, whereas compound **19** led to the formation of a mixture of three products, with alkenylphosphine sulfide **35** being the major compound.

**Figure 1:**
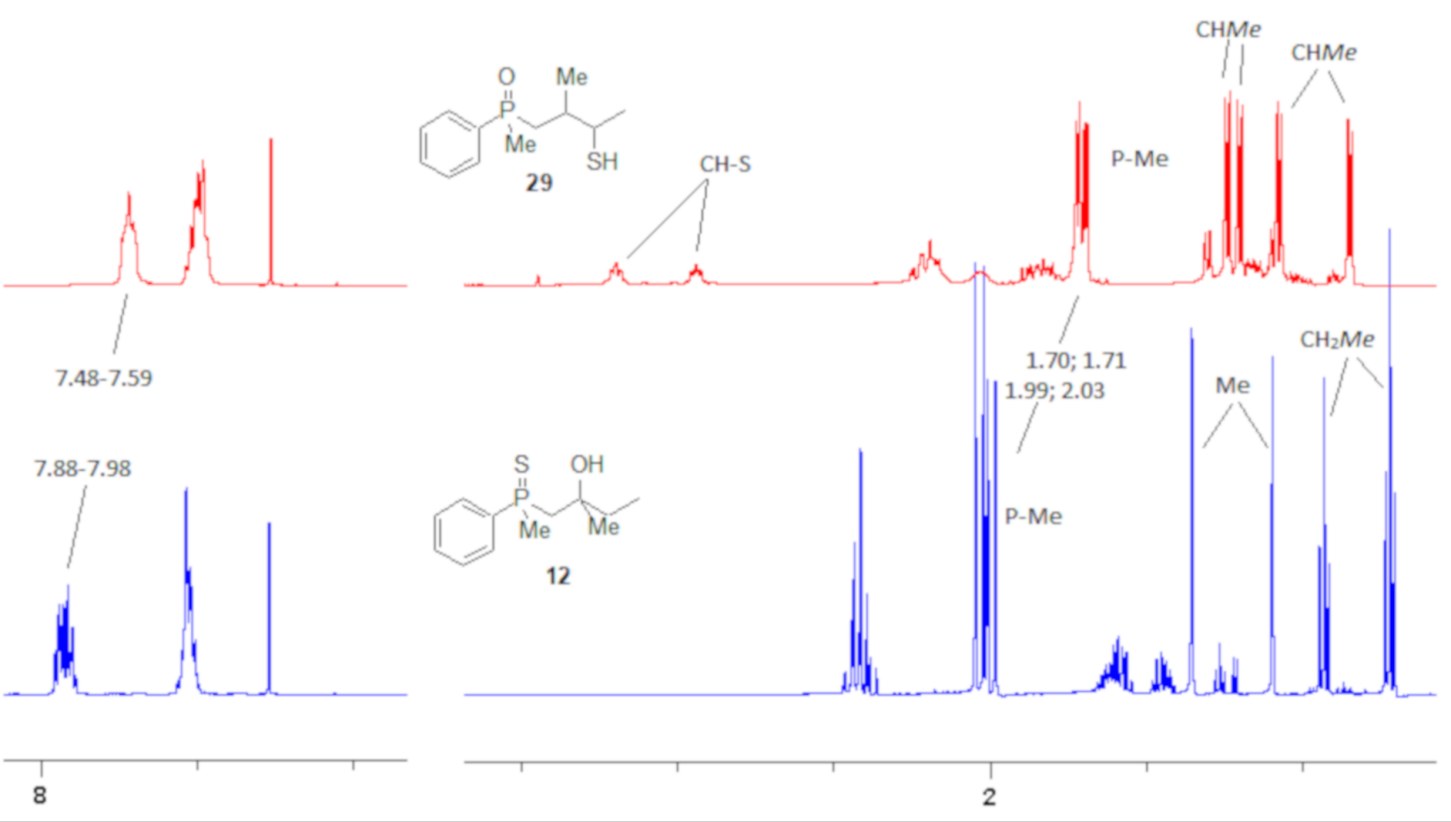
^1^H NMR spectra of compounds **12** and **29**.

The structures were assigned based on NMR analysis as all compounds failed to give single crystals for X-ray analysis. The presence of an oxygen atom attached to the phosphorus atom in the products could be deduced based on ^1^H and ^13^C NMR analysis. In the ^1^H NMR spectra of phosphine sulfide **12**, the chemical shifts of the signals belonging to the aromatic protons differed significantly more compared to the appropriate phosphine oxide. In **29** (a mixture of two diastereomers), the same chemical shifts were remarkably closer to each other (Figure 1).

In addition, signals of methyl groups attached to phosphorus could be found at 1.70 and 1.71 ppm, respectively, which was in accordance with the shifts of this group in other phosphine oxides [61–63]. Moreover, the two signals at 2.91–2.98 and 3.16–3.24 ppm, respectively, corresponded to a hydrogen atom bonded to a sulfur atom [64–65].

**Figure 2:**
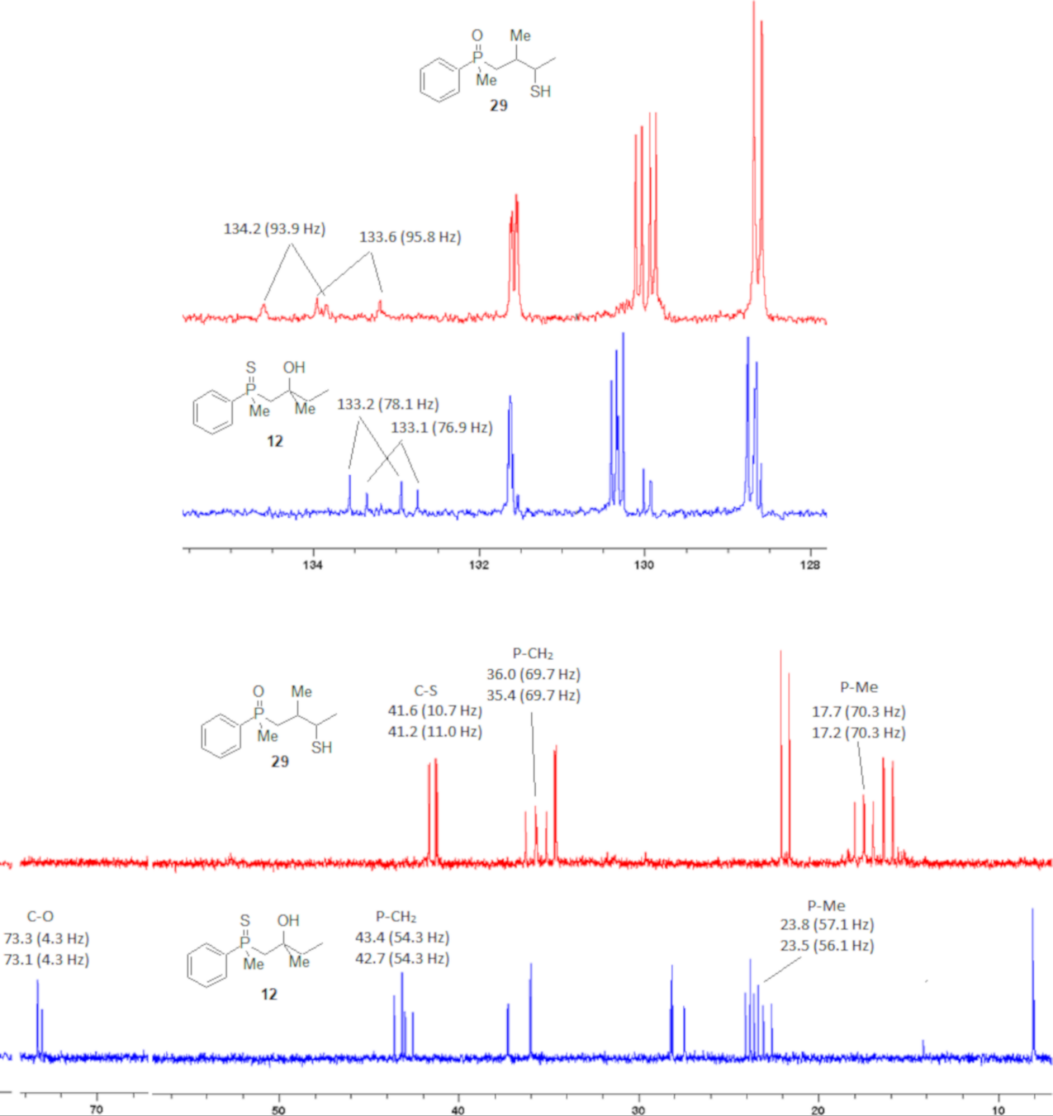
^13^C NMR spectra of compounds **12** and **29**.

Even more conclusive was the analysis of the ^13^C NMR spectra of **12** and **29** (Figure 2).

In the ^13^C NMR spectra of compound **12**, the signal of the *ipso*-carbon atom could be found at 133.1 and 133.2 ppm, respectively (a mixture of two diastereomers), with ^1^*J* coupling constants of 76.9 and 78.1 Hz, respectively. In **29**, the signal of the same carbon atom could be found at 133.6 and 134.2 ppm, respectively, with ^1^*J* coupling constants of 95.8 and 93.9 Hz, respectively, values typical for arylphosphine derivatives possessing a P=O bond [61,63,66]. Similarly, shifts and coupling constants of CH_3_ groups bound to phosphorus (17.2 ppm/70.3 Hz and 17.7 ppm/70.3 Hz) and α-CH_2_ groups (35.4 ppm/69.7 Hz and 36.0 ppm/69.7 Hz) indicated the presence of a P=O bond within the molecule. The biggest difference, however, was seen for the C–X bond. For **12**, the signals of the tertiary carbinolic carbon atom were found at 73.1 ppm/4.3 Hz and 73.3 ppm/4.3 Hz, respectively. For **29**, the same signals were found at 41.2 ppm/11.0 Hz and 41.6 ppm/10.7 Hz, respectively. The shifts indicated the presence of a C–S and not a C–O bond in the carbon skeleton [64–65], and the coupling constant value was typical for an γ-carbon atom present in a phosphine derivative [67–69].

In this regard, ^31^P NMR analysis provides very little information about the reaction mechanism. For substrates **6–25**, the ^31^P NMR shifts were in the range of 32.66–39.33 ppm, which was the typical range for aryldialkylphosphine sulfides [70–71]. The signal of aryldialkylphosphine oxides are usually found around 30–50 ppm [61,72–73], and hence the overlapping signals make this technique inappropriate for the analysis of the reaction products.

The outcome of the above-discussed reaction may suggest that the substrate underwent water elimination followed by intramolecular carbon–sulfur bond formation, and that the formed cationic intermediates underwent hydrolysis during aqueous workup, leading to the observed products. It was assumed that a similar sulfur atom migration should occur in the presence of Brønsted acid under dry conditions. Therefore, a set of phosphine sulfides prepared above was subjected to the reaction using a H_2_SO_4_/AcOH mixture (Table 3).

**Table 3:** Reaction of β-hydroxyalkylphosphine sulfides with Brønsted-acidic H_2_SO_4_.

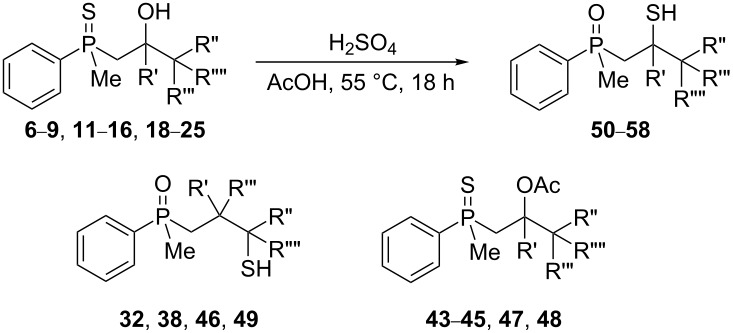		

entry	substrate	product (yield)

1	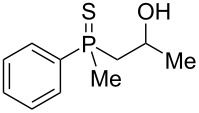 **6**	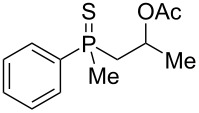 **43** (83%)
2	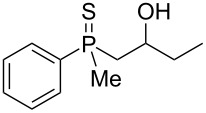 **7**	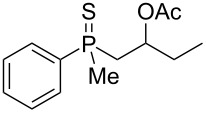 **44** (95%)
3	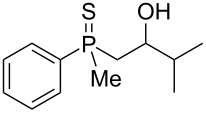 **8**	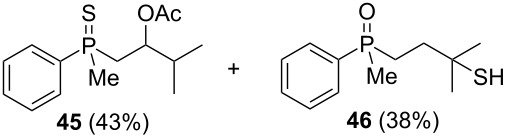
4	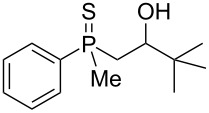 **9**	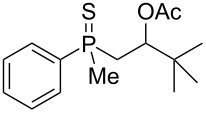 **47** (34%)^a^ (25%)
5	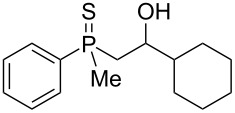 **11**	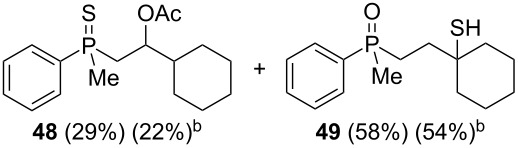
6	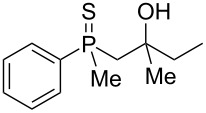 **12**	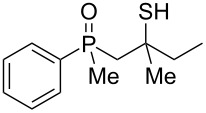 **50** (87%)
7	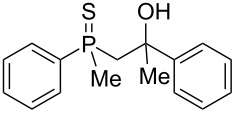 **13**	mixture of products
8	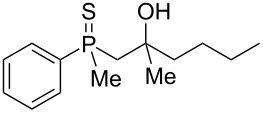 **14**	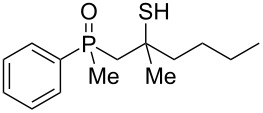 **51** (88%)
9	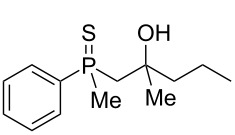 **15**	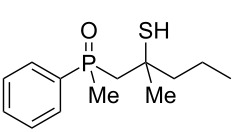 **52** (95%)
10	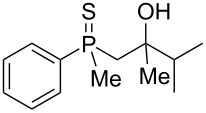 **16**	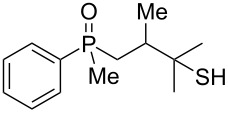 **32** (79%)
11	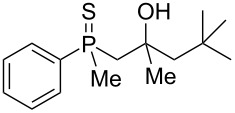 **18**	mixture of products
12	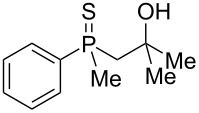 **19**	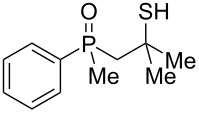 **53** (69%)
13	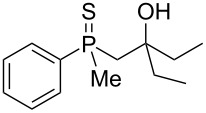 **20**	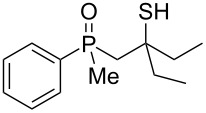 **54** (89%)
14	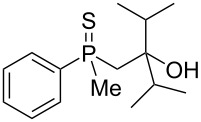 **21**	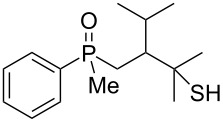 **38** (91%)
15	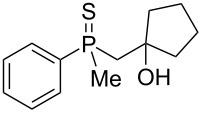 **22**	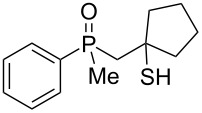 **55** (26%)
16	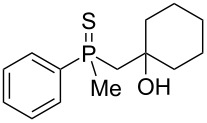 **23**	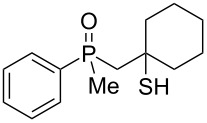 **56** (96%)
17	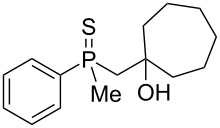 **24**	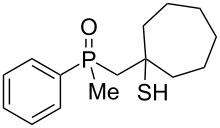 **57** (27%)
18	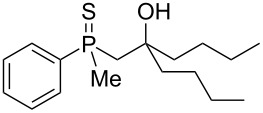 **25**	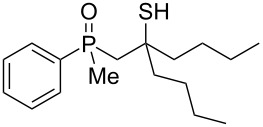 **58** (37%)

^a^Yield of the product as isolated as a mixture with substrate. ^b^*t* = 96 h.

It was found that the outcome of each reaction depended on the structure of the substrate. β-Hydroxyalkylphosphine sulfides **6–9** and **11**, derived from aldehydes, underwent estrification reactions, yielding the corresponding acetates **43–45**, **47**, and **48** (Table 3, entries 1–5). On the other hand, most of the substrates derived from ketones **12–16** and **18–25** underwent intramolecular sulfur migration with the formation of mainly β-mercaptoalkylphosphine oxides in very good yields (Table 3, entries 6, 8**–**10, and 12**–**18). Only in the case of compounds **16** and **21**, the corresponding γ-mercaptoalkylphosphine oxides **32** and **38** could be isolated as the main products, and both **13** and **18** afforded product mixtures (Table 3, entries 7 and 11). Generally, the rearrangement proceeded efficiently and most of the products were isolated in good yields except from compounds possessing cyclopentyl and cycloheptyl fragments, which afforded the β-mercaptophosphine oxides in low yields.

In order to gain insight into the reaction mechanism when using Brønsted acid, it was decided to perform the reactions at different temperatures and to monitor the reaction mixture at different times (Table 4).

**Table 4:** Control experiments under Brønsted acid catalysis.

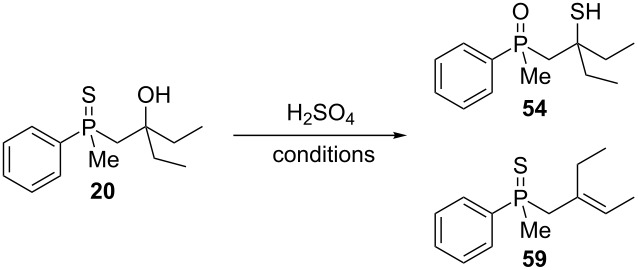				

entry	*T*, °C	*T*, h	yield of **54**^a^, %	yield of **59**^a^, %

1	55	1	93	7
2	55	2	94	6
3	55	24	100	0
4	25	1	17	83
5	25	2	30	70
6	25	4	56	44
7	25	24	100	0

^a^Conversion based on NMR analysis of the crude reaction mixture.

When the reaction was performed at 55 °C, the conversion of substrate **20** to the corresponding product **54** reached 93% after 1 hour (Table 4, entry 1). Apart from this product, the presence of alkenylphosphine sulfide **59** was detected in the reaction mixture. Prolonged heating led to the consumption of alkene **59** and its transformation to **54**. For the reaction performed at 25 °C, the principle observations remained the same except that the reaction speed was remarkably lower. Nevertheless, these experiments allowed to conclude that the rearrangement proceeded via a two-step mechanism, with the first step being hydroxy group removal followed by intramolecular formation of a sulfur–carbon bond. The second step seemed to be slow, and the intermediate carbocation could be trapped in the form of an alkenylphosphine sulfide.

**Scheme 6:**
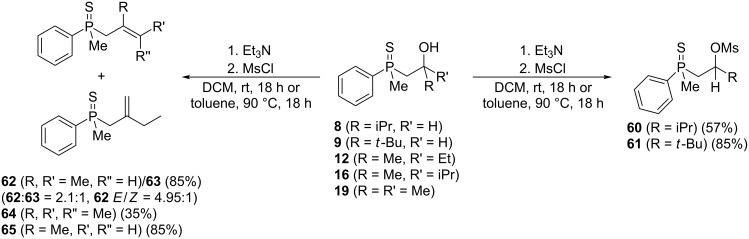
Synthesis of the alkenylphosphine sulfides used in study.

The comparison of the reactivity of the same substrate under two different reaction conditions clearly showed that distinct reaction mechanisms were responsible for the substrate’s transformation under Lewis and Brønsted acid catalysis, but both seemed to include the preliminary formation of alkenylphosphine sulfide. This was shown for the Brønsted acid-mediated reaction (Table 4) but remains to be proved for Lewis acid use. It was then decided to prepare substrates possessing double bonds at the β position (Scheme 6).

It appeared that the sulfides derived from aldehydes **8** and **9** failed to undergo transformation to the corresponding alkenylphosphine sulfides when treated with a MsCl/NEt_3_ mixture. Instead, the corresponding mesylates **60** and **61** were isolated as the sole products (Scheme 6). The compounds derived from ketones **12**, **16**, and **19** underwent elimination reactions to the β,γ-alkenylphosphine sulfides **62**, **64**, and **65** under these conditions. For sulfide **12**, a mixture of two β-alkenylphosphine sulfides **62** and **63** (a mixture of *E*- and *Z*-isomer) was obtained under each reaction condition tested.

**Scheme 7:**
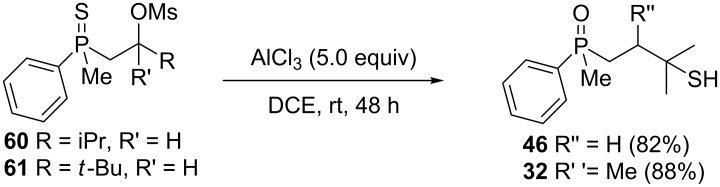
The reaction of mesylate compounds with Lewis-acidic AlCl_3_.

Once all the compounds had been prepared, it was decided to test the reactivity of mesylates **60** and **61** in the presence of AlCl_3_ (Scheme 7).

The reaction of these compounds with aluminum chloride proceeded smoothly, leading to the corresponding γ-mercaptoalkylphosphine oxides in good yields. Surprisingly, the reactivity of the mesylates **60** and **61** appeared to be completely different from the parent β-hydroxyalkylphosphine sulfides **8** and **9**, which failed to undergo a rearrangement (see Table 2, entries 2 and 3). The difference must therefore have arisen from the different C–O bond dissociation energies of the alcohols and mesylates.

Assuming that the Lewis acid coordinated to either hydroxy oxygen atom or to one of the oxygen atoms of the mesyl group, the activation energies for C–O bond dissociation are ca. 34.6 kcal/mol for the secondary alcohol **8**, ca. 23.3 kcal/mol for the mesylate **60**, and ca. 25.0 kcal/mol for the tertiary alcohol **20**, as estimated by DFT calculations. The value for mesylate roughly fitted the range according to which the reaction may proceed at room temperature. For mesylate **61**, with a *tert*-butyl group at the β-carbon atom, the initial dissociation of the C–O bond must have been followed by CH_3_ group migration before the observed sulfur transfer took place.

**Scheme 8:**
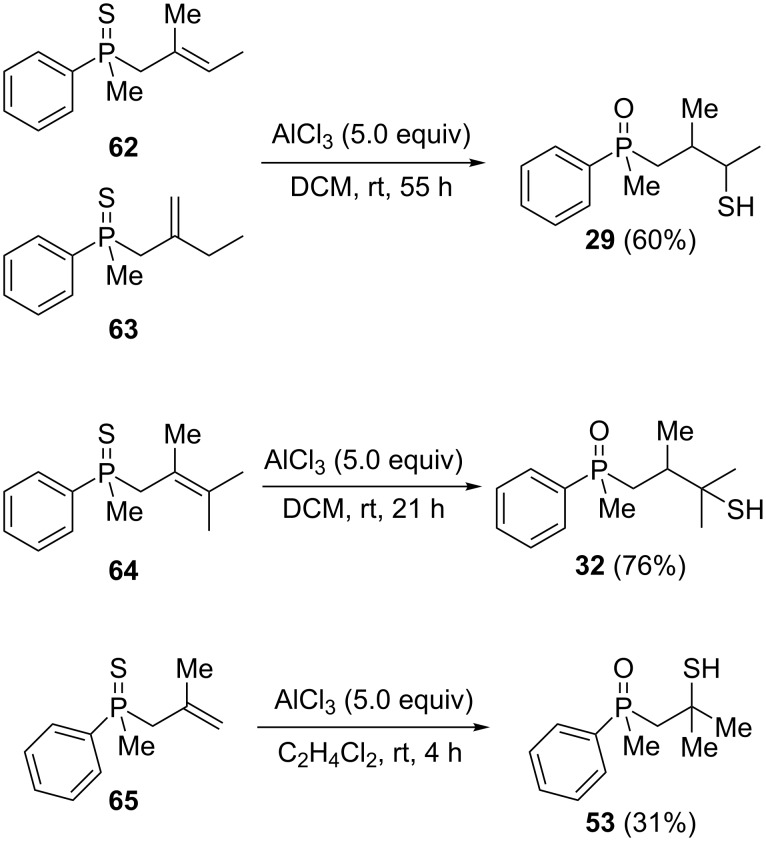
The reaction of alkenylphosphine sulfides with AlCl_3_.

Analogous attempts were made for compounds **62**–**65** (Scheme 8).

A mixture of alkenylphosphine sulfides **62** and **63** afforded the same γ-mercaptoalkylphosphine oxide **29** in good yield in the presence of AlCl_3_. Similarly, sulfide **64** afforded the phosphine oxide **32**, possessing a mercapto group at the γ-carbon atom, under the same reaction conditions. Finally, sulfide **65** underwent a transformation into oxide **53**, possessing an SH group at the β-carbon atom, under the reaction conditions. The latter result was slightly different from the two previous ones, as here, the sulfur atom migrated to the β and not the γ-carbon atom. Moreover, the parent alcohol **19** underwent mainly a dehydration reaction and the formation of α,β-alkenylphosphine sulfide **35** in the presence of AlCl_3_.

All of these experiments suggested that the transformation of β-hydroxyalkylphosphine sulfides in the presence of Lewis acid most probably proceeded through the initial formation of an intermediate alkenylphosphine sulfide, which then underwent an intramolecular reaction leading to a formal [1,4]-rearrangement product. It seemed that the position of the double bond was crucial for the rearrangement, which had to be situated between the β- and the γ-carbon atom. In this position, there was no coupling with the thiophosphoryl group, and the double bond retained its nucleophilic character. This allowed the coordination of an AlCl_3_ molecule to the C=C bond, which then became susceptible to an intramolecular nucleophilic attack by a sulfur atom. For α,β-alkenylphosphine sulfide **35**, the conjugation of the double bond with thiophosphoryl fragment made the activation by AlCl_3_ impossible, which prevented the rearrangement.

**Scheme 9:**
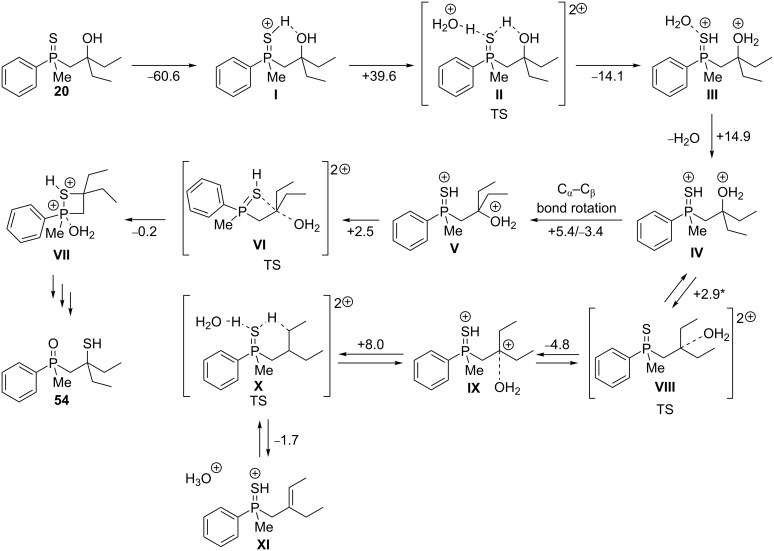
Rearrangement of **20** in the presence of Brønsted acid. The calculated energies next to the arrows are reported in kcal/mol.

To gain detailed insights into the possible reaction mechanism, DFT calculations were performed on model β-hydroxyalkylphosphine sulfide **20**, possessing two ethyl groups at the β-carbon atom. First, the Brønsted acid-catalyzed rearrangement was investigated by molecular modeling (Scheme 9).

The first step of the rearrangement involved the protonation of **20**, which afforded intermediate **I**, where the proton inserted between the sulfur and the oxygen atom. This intermediate appeared to be stable and failed to undergo further transformation, according to calculations. The reason of this behavior was most probably the cyclic structure of the intermediate. However, if the intermediate **I** can undergo a second protonation process, intermediate **III** forms through transition state **II**. Water elimination from **III** proceeded with slight increase in energy, affording intermediate **IV**, which may undergo two different transformations: The first transformation involves dissociation of a water molecule, which leads to the formation of the dication **IX** through transition state **VIII**. Taking into account that all energy barriers were well below 10 kcal/mol, it seemed that this transformation was reversible, and therefore the dication **IV** may have entered an alternative pathway, which involved the rotation of the molecule around the C_α_–C_β_ bond (activation barrier: +5.4 kcal/mol, stabilization: −3.4 kcal/mol), which was feasible under the reaction conditions. The formed dication **V** underwent an intramolecular nucleophilic substitution through transition state **VI**, which proceeded with a small activation barrier, leading to the cyclic intermediate **VII** and further to the rearranged product **54**.

The tentative mechanism discussed above does not explain the formation of the γ-mercaptoalkylphosphine sulfides from β-hydroxyalkylphosphine sulfides **16** and **21** and, to some extent, **8** and **11**. In these cases, DFT calculations allowed to propose a slightly different mechanism (see Supporting Information File 1). In this case, a water molecule, formed after protonation of the OH group, facilitated proton transfer from the γ- to β-carbon atom via a process similar to the elimination/electrophilic addition to alkenes pathway.

**Scheme 10:**
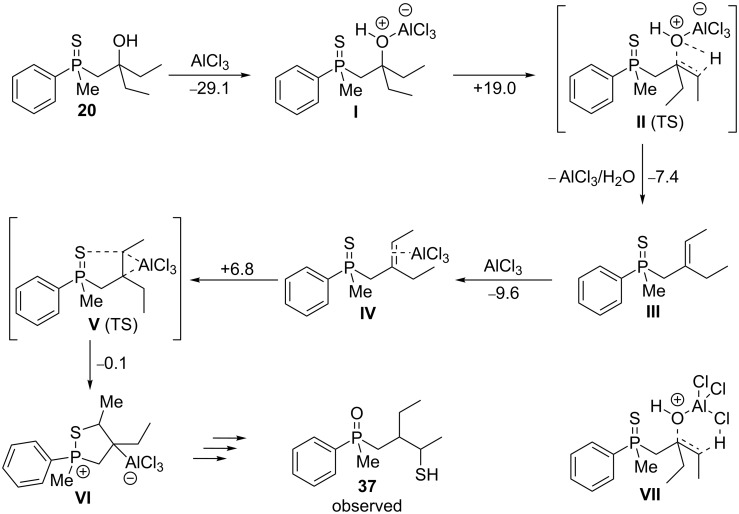
Rearrangement of **20** in the presence of Lewis acid. The calculated energies next to the arrows are reported in kcal/mol.

The computational results discussed above showed that the crucial point for sulfur atom migration was the formation of a tertiary carbocation at the β-carbon atom, which was quite straightforward under these acidic conditions. To understand the completely different selectivity in Lewis acid-catalyzed rearrangements, DFT was again applied to the reaction between **20** and AlCl_3_ (Scheme 10).

Compound **20** underwent a complexation with an AlCl_3_ molecule, leading to intermediate **I**, and the overall transformation proceeded with remarkable stabilization. In the next step, dissociation of the C−O bond occurred through transition state **II**, finally leading to alkene **III**. The activation energy for the C−O bond cleavage in **20**, a tertiary alcohol, was similar to the value calculated for the mesylate **60** (23.3 kcal/mol) and was lower than that for β-hydroxyalkylphosphine sulfide **8**, formally a secondary alcohol (34.6 kcal/mol).

One could consider an alternative mechanism for the formation of **III** including the six-membered transition state **VII**. In this case, proton migration would occur from the γ-carbon atom to chloride, resulting in the formation of HCl, Al(OH)Cl_2_, and intermediate **III**. However, all attempts to monitor this particular transition state failed.

The formed intermediate **III** underwent a reaction with a second AlCl_3_ molecule, which associated with the double bond, yielding **IV** in a slightly exothermic process. The formed intermediate possessing an activated C=C bond underwent intramolecular carbon–sulfur bond formation through the transition state **V**. In this case, the reaction proceeded solely at the γ-carbon atom, and all attempts directed at the discovery of the transition state where the sulfur attack occurred at the β-carbon atom failed. It was possible that the electron density of the C=C double bond was slightly shifted towards the β-carbon atom due to possible interaction with the P=S fragment, and coordination of an AlCl_3_ molecule additionally activated the γ-carbon atom for a sulfur attack.

Regarding the correctness of the proposed mechanism, two issues associated with the reactivity of **17** and **19**, respectively, in the presence of AlCl_3_ must be discussed. The first is the formation of the adduct with *tert*-butyl methyl ketone, and the second is the formation of the adduct with acetone. For **17**, the formation of β,γ-alkenylphosphine sulfide was not possible, whereas the transformation of **19** afforded mainly the α,β-unsaturated compound **35**. DFT analysis of the C–O bond cleavage in the adducts analogous to **I** revealed two different pathways. A complex of **19** with AlCl_3_ underwent simultaneous C–O bond cleavage and α-hydrogen (but not γ-hydrogen) atom abstraction, which led to **35**, with activation barrier of +24.8 kcal/mol and a stabilization energy of −16.1 kcal/mol. Yet, at the moment, this preference remains to be further investigated.

On the other hand, the complex of **17** and AlCl_3_ underwent C–O bond cleavage and γ-hydrogen atom abstraction, with an activation barrier of +16.5 kcal/mol, which led to the corresponding alkene, with a stabilization energy of −13.5 kcal/mol. The loss of AlCl_3_/H_2_O afforded the β,γ-alkenylphosphine sulfide, which then underwent coordination of the second AlCl_3_ molecule, with an overall slight stabilization (+6.0/−8.5 kcal/mol). The adduct analogous to **IV** underwent methyl group migration to the β-carbon atom, along with the formation of a covalent Al–C bond. The last step required a remarkable activation energy (31.0 kcal/mol), which was followed by slight stabilization of the formed γ-carbanion (−6.1 kcal/mol). The latter should then undergo intramolecular C–S bond formation, followed by hydrolysis, finally leading to the rearranged product.

**Scheme 11:**
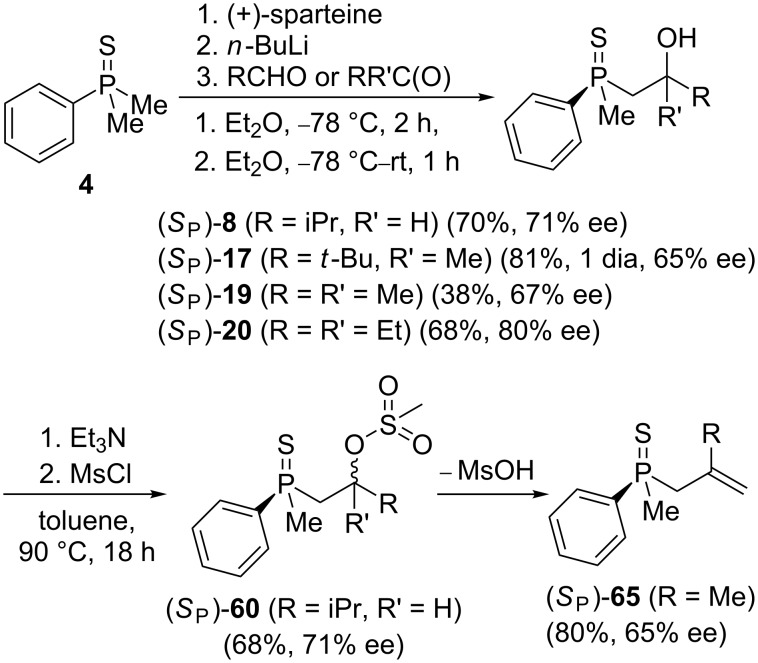
The synthesis of chiral substrates for rearrangement reactions.

It was then decided to investigate the stereoselectivity of the acid-catalyzed rearrangement using enantiomerically enriched phosphine sulfides. Therefore, the chiral substrates (*S*_P_)**-60**, (*S*_P_)**-65**, (*S*_P_)**-19**, and (*S*_P_)**-20** were prepared from dimethylphenylphosphine sulfide **4** using sparteine chemistry (Scheme 11).

**Scheme 12:**
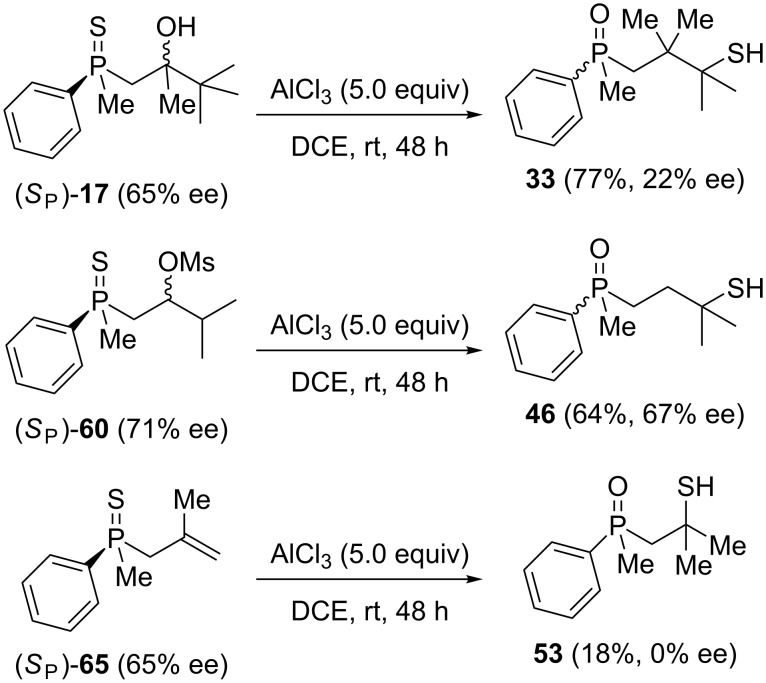
The reaction of (*S*_P_)**-60** and (*S*_P_)**-65** with AlCl_3_.

First, the enantioenriched alcohol (*S*_P_)**-17**, mesylate (*S*_P_)**-60**, and alkenylphosphine sulfide (*S*_P_)**-65** were subjected to the reaction with Lewis-acidic AlCl_3_ (Scheme 12).

Rearrangement of the tertiary alcohol (*S*_P_)**-17** proceeded very efficiently, affording the corresponding product in high yield. Unfortunately, the stereoselectivity of the reaction appeared to be quite low due to the significant racemization of the phosphorus center under the applied conditions. On the other hand, the treatment of (*S*_P_)**-60** with Lewis acid led to the formation of chiral γ-mercaptoalkylphosphine oxide **46** in good yield and with only slight decrease in enantiomeric excess. The transformation of alkenylphosphine sulfide (*S*_P_)**-65** under similar reaction conditions led to the formation of β-mercaptoalkylphosphine oxide **53**, albeit in low yield and with a complete lack of stereoselectivity.

The results presented above show that, at least for mesylates, the sulfur atom migration may have been a stereospecific process. What was more striking, the complete racemization of (*S*_P_)**-65** and the extended racemization of (*S*_P_)**-17** occurred under the reaction conditions. This suggested that the rearrangement may have proceeded either via two different pathways, or one of the reaction components may have influence the rate of racemization. In the case of (*S*_P_)**-17**, the postulated dissociation of AlCl_3_⋅OH may have generated traces of HCl, which may have react with the cyclic intermediate, leading to a pentavalent compound. The latter would have been very susceptible to Berry or turnstile pseudorotation. This, in turn, should have led to a remarkable deterioration of the enantiomeric excess of the product. The same might be postulated for the rearrangement of alkenylphosphine sulfide **65**, which should form a pentavalent intermediate equally well.

**Scheme 13:**
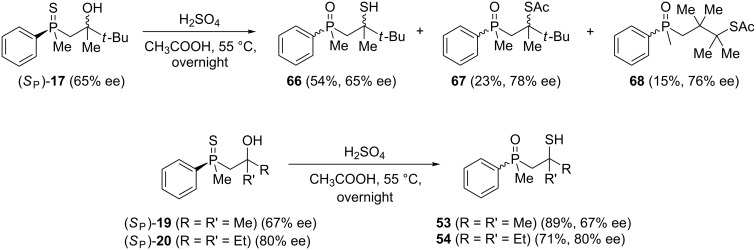
Reaction of chiral β-hydroxyalkylphosphine sulfides with Brønsted acid.

In the next step, the enantiomerically enriched β-hydroxyalkylphosphine sulfides (*S*_P_)**-19** and (*S*_P_)**-20** were subjected to the rearrangement in the presence of Brønsted-acidic H_2_SO_4_ (Scheme 13).

It was found that phosphine sulfide (*S*_P_)**-17** underwent efficient rearrangement, affording a mixture of three compounds: β-mercaptoalkylphosphine oxide **66**, its S-acylated derivative **67**, and S-acylated γ-mercaptoalkylphosphine oxide **68**. Compound **66** was obtained with complete stereoselectivity, whereas the enantiomeric excess of both **67** and **68** was higher, 78% ee and 76% ee, respectively. The phosphine sulfides (*S*_P_)**-19** and (*S*_P_)**-20** underwent rearrangement to the corresponding chiral β-mercaptoalkylphosphine oxides **53** and **54** in good yields and with complete stereoselectivity as compared to the rearrangement of (*S*_P_)**-17**. Based on this, it can be concluded that sulfur atom migration occurred via a highly stereospecific pathway through an intramolecular substitution mechanism.

Here, the stereochemical differences in the outcome of the rearrangement of (*S*_P_)**-17** must be noticed. In the presence of Lewis-acidic AlCl_3_, the rearrangement led effectively to the corresponding γ-mercaptoalkylphosphine oxide **33**, albeit with a remarkable deterioration of enantiomeric excess. The rearrangement in the presence of Brønsted-acidic H_2_SO_4_ afforded only minor amounts of S-acylated γ-mercaptoalkylphosphine oxide **68**, but the enantiomeric excess was even higher than in the substrate. This suggested that the [1,3]-rearrangement was stereospecific in the presence of Brønsted acid, whereas the [1,4]-rearrangement proceeded with additional partial enrichment of one enantiomer. The latter may have occurred through the preferential acylation of one of two diastereoisomers under the reaction conditions where the second chirality center at the phosphorus atom influenced the ratio of the acylation reaction.

**Scheme 14:**
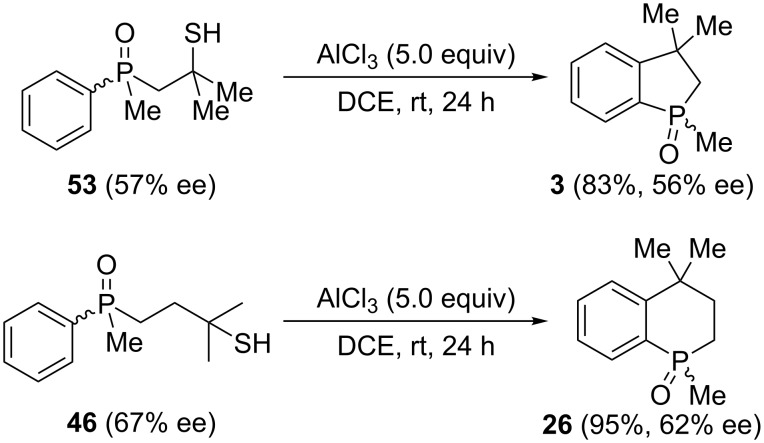
Attempted cyclization of enantiomerically enriched **53** and **46**.

With chiral β-mercaptoalkylphosphine sulfides in hand, it was decided to check their ability to undergo cyclization in the presence of AlCl_3_ (Scheme 14).

These results differed remarkably from the reactivity of the analogous hydroxyalkylphosphine sulfides, which exclusively underwent sulfur atom migration. The nature of this difference is currently a subject of further research in our laboratory. Nevertheless, the above results opened a new pathway for the synthesis of bicyclic organophosphorus compounds possessing a chirality center at phosphorus. Moreover, the formation of the bicyclic compounds proceeded under mild reaction conditions and with a high degree of stereoselectivity.

## Conclusion

In conclusion, in the present paper, we describe the rearrangement of a series of β-hydroxyalkylphosphine sulfides. Both experimental and theoretical analysis of the reaction mechanism were performed. Depending on the structure of the initial alcohol, the acid used (Brønsted or Lewis), and the reaction conditions (temperature, time), the acid-promoted transformations of the alcohols may lead to two different products: β- or γ-mercaptoalkylphosphine oxides. The formation of γ-mercaptoalkylphosphine oxides proceeded with the formation of an additional chirality center at the γ-carbon atom. Attempted rearrangements of P-stereogenic β-hydroxy- and β-mesyloxyalkylphosphine sulfides in the presence of either Brønsted or Lewis acid led to the corresponding rearranged products with variable degree of stereoselectivity. It was found that the P-stereogenic β-/γ-mercaptoalkylphosphine oxides underwent intramolecular cyclization to the corresponding bicyclic phosphine oxides with high stereoselectivity and high yield under mild reaction conditions.

## Supporting Information

File 1 Experimental procedures, compound characterization data, a mechanism for the rearrangement of **16** in the presence of Brønsted acid assessed by computational methods, and coordinates of computed compounds.

File 2 Copies of NMR spectra of all pure compounds and their mixtures.
