# Supplementary material for: [1,3]/[1,4]-Sulfur atom migration in β-hydroxyalkylphosphine sulfides
Source: Beilstein J Org Chem. 2020 Jan 21;16:88–105. doi: 10.3762/bjoc.16.11 (PMC7006489; doi:10.3762/bjoc.16.11)
Supplement: File 1 — Experimental procedures, compound characterization data, a mechanism for the rearrangement of 16 in the presence of Brønsted acid assessed by computational methods, and coordinates of computed compounds. [file Beilstein_J_Org_Chem-16-88-s001.pdf]

BEILSTEIN JOURNAL OF ORGANIC CHEMISTRY

Supporting Information

for

[1,3]/[1,4]-Sulfur atom migration in β-hydroxyalkylphosphine sulfides

Katarzyna Włodarczyk, Piotr Borowski and Marek Stankevič

Beilstein J. Org. Chem. **2020,** *16,* 88–105. doi:10.3762/bjoc.16.11

Experimental procedures, compound characterization data, a mechanism for the rearrangement of 16 in the presence of Brønsted acid assessed by computational methods, and coordinates of computed compounds

License and Terms: This is a supporting information file under the terms of the Creative Commons Attribution License (https://creativecommons.org/licenses/by/4.0). Please note that the reuse, redistribution and reproduction in particular requires that the authors and source are credited.
The license is subject to the *Beilstein Journal of Organic Chemistry* terms and conditions: (https://www.beilstein-journals.org/bjoc)

Experimental

All reactions were performed under an argon atmosphere using Schlenk techniques. Only dry solvents were used, and the glassware was heated under vacuum prior to use. Solvents for chromatography were distilled once before use, and solvents for extraction were used as received. Tetrahydrofuran and diethyl ether were dried over sodium/benzophenone ketyl. Dichloromethane and ethylene dichloride were dried over anhydrous calcium chloride. *n*-BuLi and aluminium chloride were commercially available and used as received, and (+)-sparteine was purchased from ABCR GmbH.

The NMR spectra were recorded with 500 MHz spectrometers in $CDCl_3$ as a solvent at room temperature unless otherwise noted. Chemical shifts (δ) are reported in ppm relative to the residual solvent peak. Mass spectra were recorded with a spectrometer using EI and a standard column with the following parameters: pressure 65kPa, total flow 33.9 mL/min, column flow 1.0 mL/min, linear velocity 36.8 cm/s, split 30, temperature program 80 °C hold 0.5 min, 80–340 °C/19 °C/min, hold 2 min, 300–340 °C/15 °C/min, hold 3.26 min, total 20 min). High resolution mass spectrometry analyses were obtained using a spectrometer equipped with an IT–TOF detector. Thin-layer chromatography (TLC) was performed with precoated silica gel plates and visualized by UV light or iodine on silica gel. The reaction mixtures were purified by column chromatography over silica gel (60−240 mesh). Melting points were determined in a capillary tube.

The theoretical results were obtained with the aid of density functional theory (DFT) [1]. In all reported cases, the B3LYP hybrid functional [2] in conjunction with the polarized valence triple zeta (VTZP) basis set 6-311++G**[3,4] with diffuse functions was used. Geometry optimization for all the systems, followed by the frequency calculations, were performed. The type of a stationary point during the optimization procedure was determined based on the

analysis of the obtained frequencies. In the case of minima (stable molecules), all computed frequencies were real. One imaginary frequency was obtained in the case of each transition state. All reported energies were corrected for the zero-point vibrational energies (ZPVE).

Dimethylphenylphosphine sulfide (4)

In a flame-dried two-necked round-bottom flask (250 mL) equipped with magnetic stirrer, reflux condenser, argon inlet and a septum was placed dimethylphenylphosphine oxide (0.90 g, 5.89 mmol) in 50 mL of dry toluene. Then P_2S_5 (1.31 mL, 5.89 mmol) was added in portion. The reaction mixture was stirred at 110 °C for 16 h. The mixture was quenched with 1 M NaOH (50 mL) and extracted with DCM (3 × 20 mL). The combined organic phases were dried over $MgSO_4$, filtered, and evaporated under reduced pressure. The residue was purified by column chromatography using $CHCl_3$ /MeOH 15:1 as eluent yielding 4 as pale yellow solid in 95% (0.95 g); mp 44.6–45.5°C; R_f 0,32 (Hexane/AcOEt 4:1); ^{1}H NMR (500 MHz, $CDCl_3$) δ 2.00 (d, $J_{P\text{–}H}$ = 12.9 Hz, 6H), 7.46–7.57 (m, 3H), 7.85–7.95 (m, 2H); ^{13}C NMR (126 MHz, $CDCl_3$) δ 22.8 (d, $J_{P\text{–}C}$ = 57.2 Hz), 128.6 (d, $J_{P\text{–}C}$ = 11.8 Hz), 129.9 (d, $J_{P\text{–}C}$ = 10.9 Hz), 131.5 (d, $J_{P\text{–}C}$ = 2.7 Hz), 133.6 (d, $J_{P\text{–}C}$ = 79.9 Hz); ^{31}P NMR (202 MHz, $CDCl_3$) δ 32.61; GC t_R = 8.13 min; GCMS (EI, 70 eV) *m/z* = 171 (9), 170 (M) (94), 155 (100), 153 (20), 137 (12), 123 (11), 121 (16), 109 (27), 107 (10), 91 (39), 77 (37), 65 (14), 63 (17), 51 (19), 45 (9); HRMS (ESI–TOF) m/z: [M+H] Calcd for $C_8H_{11}OPS$ 233.0541; Found 233.0541. Analytical data are in accordance with the literature [5,6].

General procedure for the synthesis of β-hydroxyalkylphosphine sulfides 6–25

In a flame-dried Schlenk tube (50 mL) equipped with magnetic stirrer and inert gas inlet was placed dimethylphenylphosphine sulfide (1.0 mmol) in THF (5 mL). The mixture was cooled to −78 °C (dry ice-acetone) and *n*-BuLi (1.1 mmol, 1.6 M in hexane) was added. After the orange mixture was stirred for 1 h at −78 °C, aldehyde or ketone (1.5 mmol) was added until no color remained. Reaction was allowed to warm to room temperature and was stirred for 1 h. The reaction was quenched by addition of saturated NH_4Cl solution (10 mL) and extracted with DCM (3 × 20 mL). The combined organic phases were dried over $MgSO_4$, filtered, and evaporated under reduced pressure. The residue was purified by column chromatography using $CHCl_3$ as eluent.

(2-Hydroxypropyl)methylphenylphosphine sulfide (6). This compound was prepared according to the general procedure from dimethylphenylphosphine sulfide **4** (0.201 g, 1.18 mmol) and acetaldehyde (0.078 g, 1.77 mmol) as a yellow oil; yield of two diastereomers 0.195 g (77%), (dr = 51:49).

Major diastereomer: Yield 39%, (0.008 g, 3% isolated as a pure compound); yellow oil; R_f 0.64 (Hexane/AcOEt/iPrOH 5:1:1); ^{1}H NMR (500 MHz, $CDCl_3$) δ 1.29 (dd, $J_{H\text{–}H}$ = 6.3 Hz, $J_{H\text{–}H}$ = 1.9 Hz, 3H), 2.02 (d, $J_{P\text{–}H}$ = 12.9 Hz, 3H), 2.21–2.33 (m, 2H), 3.34 (bs, 1H), 4.26–4.52 (m, 1H), 7.40–7.65 (m, 3H), 7.77–7.98 (m, 2H); ^{13}C NMR (126 MHz, $CDCl_3$) δ 21.2 (d, $J_{P\text{–}C}$ = 57.2 Hz), 24.9 (d, $J_{P\text{–}C}$ = 14.5 Hz), 42.5 (d, $J_{P\text{–}C}$ = 54.5 Hz), 63.7 (d, $J_{P\text{–}C}$ = 3.6 Hz), 128.7 (d, $J_{P\text{–}C}$ = 12.7 Hz), 130.2 (d, $J_{P\text{–}C}$ = 10.0 Hz), 131.7 (d, $J_{P\text{–}C}$ = 2.7 Hz), 132.6 (d, $J_{P\text{–}C}$ = 78.1 Hz); ^{31}P NMR (202 MHz, $CDCl_3$) δ 35.48; GC t_R = 10.11 min; GCMS (EI, 70 eV) *m/z* = 214

(M) (3), 157 (10), 156 (100), 155 (20), 141 (28), 123 (15), 109 (12), 91 (15); HRMS (ESI–TOF) m/z: [M+H] Calcd for $C_{10}H_{15}OPS$ 215.0662; Found 215.0654.

Minor diastereomer: Yield 38%, (0.047 g, 18% isolated as a pure compound); yellow oil; R_f 0.68 (Hexane/AcOEt/iPrOH 5:1:1); ^{1}H NMR (500 MHz, $CDCl_3$) δ 1.22 (dd, $J_{H–H}$ = 6.1 Hz, $J_{H–H}$ = 2.0 Hz, 3H), 2.02 (d, $J_{P–H}$ = 13.2 Hz, 3H), 2.17-2.25 (m, 1H), 2.26-2.35 (m, 1H), 3.74 (bs, 1H), 4.11–4.26 (m, 1H), 7.49–7.61 (m, 3H), 7.86–7.96 (m, 2H); ^{13}C NMR (126 MHz, $CDCl_3$) δ 22.3 (d, $J_{P–C}$ = 57.2 Hz), 24.9 (d, $J_{P–C}$ = 15.4 Hz), 42.1 (d, $J_{P–C}$ = 56.3 Hz), 63.6 (d, $J_{P–C}$ = 4.5 Hz), 128.8 (d, $J_{P–C}$ = 12.7 Hz), 130.5 (d, $J_{P–C}$ = 10.9 Hz), 131.9 (d, $J_{P–C}$ = 78.1 Hz), 131.8 (d, $J_{P–C}$ = 2.7 Hz); ^{31}P NMR (202 MHz, $CDCl_3$) δ 36.51; GC t_R = 10.05 min; GCMS (EI, 70 eV) *m/z* = 214 (M) (4), 156 (100), 155 (24), 141 (30), 123 (17), 121 (13), 109 (15), 91 (20) 78 (40) 77 (22), 63 (31), 51 (11); HRMS (ESI–TOF) m/z: Calcd for $C_{10}H_{15}OPS$ [M+H] 215.0646; Found 215.0654.

(2-Hydroxybutyl)methylphenylphosphine sulfide (7). This compound was prepared according to the general procedure from dimethylphenylphosphine sulfide **4** (0.464 g, 2.73 mmol) and propionaldehyde (0.237 g, 4.09 mmol); yield of two diastereomers 0.562 g (90%), (dr = 57:43).

Major diastereomer: Yield 45%; 0.106 g (17% isolated as a pure compound); white solid; mp 85.2–86.8 °C; R_f 0.38 ($CHCl_3$); ^{1}H NMR (500 MHz, $CDCl_3$) δ 0.94 (t, $J_{H–H}$ = 7.4 Hz, 3H), 1.44–1.49 (m, 1H), 1.51–1.59 (m, 1H), 2.02 (d, $J_{P–H}$ = 12.9 Hz, 3H), 2.15–2.33 (m, 2H), 3.35 (bs, 1H), 4.04–4.16 (m, 1H), 7.45–7.57 (m, 3H), 7.83–7.94 (m, 2H); ^{13}C NMR (126 MHz, $CDCl_3$) δ 9.6, 21.2 (d, $J_{P–C}$ = 56.3 Hz), 31.4 (d, $J_{P–C}$ = 13.6 Hz), 40.7 (d, $J_{P–C}$ = 55.4 Hz), 68.6 (d, $J_{P–C}$ = 4.5 Hz), 128.7 (d, $J_{P–C}$ = 12.7 Hz), 130.2 (d, $J_{P–C}$ = 10.9 Hz), 131.7 (d, $J_{P–C}$ = 2.7 Hz), 132.7 (d, $J_{P–C}$ = 78.1 Hz); ^{31}P NMR (202 MHz, $CDCl_3$) δ 36.08; GC t_R = 10.84 min;

GCMS (EI, 70 eV) *m/z* = 228 (2) (M), 157 (11), 156 (100), 155 (30), 141 (22), 123 (12), 109 (12), 91 (17), 78 (32) 77 (17) 63 (20); HRMS (ESI–TOF) m/z: [M+H] Calcd for $C_{11}H_{17}OPS$ 229.0802; Found 229.0810.

Minor diastereomer: Yield 45%; 0.131 g (24% isolated as a pure compound); colorless oil; R_f 0.34 ($CHCl_3$); ^{1}H NMR (500 MHz, $CDCl_3$) δ 0.89 (t, $J_{H–H}$ = 7.4 Hz, 3H), 1.40–1.51 (m, 1H), 1.51–1.63 (m, 1H), 2.03 (d, $J_{P–H}$ = 13.2 Hz, 3H), 2.15–2.33 (m, 2H), 2.77 (bs, 2H), 3.88–4.01 (m, 1H), 7.47–7.60 (m, 3H), 7.84–7.97 (m, 2H); ^{13}C NMR (126 MHz, $CDCl_3$) δ 9.5, 22.5 (d, $J_{P–C}$ = 57.2 Hz), 31.4 (d, $J_{P–C}$ = 14.5 Hz), 40.4 (d, $J_{P–C}$ = 56.3 Hz), 68.5 (d, $J_{P–C}$ = 4.5 Hz), 128.7 (d, $J_{P–C}$ = 12.7 Hz), 130.5 (d, $J_{P–C}$ = 10.0 Hz), 131.7 (d, $J_{P–C}$ = 3.6 Hz), 132.3 (d, $J_{P–C}$ = 78.1 Hz); ^{31}P NMR (202 MHz, $CDCl_3$) δ 37.17; GC t_R = 10.70 min; GCMS (EI, 70 eV) *m/z* = 228 (2) (M), 157 (11), 156 (100), 155 (29), 141 (23), 123 (13), 109 (11), 91 (17), 78 (32) 77 (17) 63 (20); HRMS (ESI–TOF) m/z: [M+H] Calcd for $C_{11}H_{17}OPS$ 229.0813; Found 229.0810.

(2-Hydroxy-3-methylbutyl)methylphenylphosphine sulfide (8). This compound was prepared according to the general procedure from dimethylphenylphosphine sulfide **4** (0.315 g, 1.85 mmol) and isobutyraldehyde (0.200 g, 2.77 mmol); yield of two diastereomers 0.338 g, (75%), (dr = 55:45).

Major diastereomer: Yield 42%; 0.163 g (36% isolated as a pure compound); pale yellow oil; R_f 0.42 (Hexane/EtOAc 6:1); ^{1}H NMR (500 MHz, $CDCl_3$) δ 0.92 (dd, $J_{H–H}$ = 9.8 Hz, $J_{H–H}$ = 6.8 Hz, 6H), 1.70–1.79 (m, 1H), 2.01 (d, $J_{P–H}$ =12.9 Hz, 3H), 2.12–2.21 (m, 1H), 2.21–2.29 (m, 1H), 3.57 (s, 1H), 3.91–3.99 (m, 1H), 7.46–7.57 (m, 3H), 7.83–7.93 (m, 2H); ^{13}C NMR (126 MHz, $CDCl_3$) δ 17.3, 18.0, 20.9 (d, $J_{P–C}$ = 56.3 Hz), 34.5 (d, $J_{P–C}$ = 13.6 Hz), 38.2 (d, $J_{P–C}$ = 56.3 Hz), 71.7 (d, $J_{P–C}$ = 4.5 Hz), 128.7 (d, $J_{P–C}$ = 11.8 Hz), 130.2 (d, $J_{P–C}$ = 10.9 Hz),

131.7 (d, $J_{P–C}$ = 3.6 Hz), 132.7 (d, $J_{P–C}$ = 79.0 Hz); ^{31}P NMR (202 MHz, $CDCl_3$) δ 37.15; GC t_R = 9.51 min; GCMS (EI, 70 eV) *m/z* = 224 (M–H_2O) (17), 157 (11), 156 (100), 155 (11), 141 (22), 123 (13), 109 (11), 91 (19); HRMS (ESI–TOF) m/z: [M+H] Calcd for $C_{12}H_{19}OPS$ 243.0966; Found 243.0967.

Minor diastereomer: Yield 33%; 0.116g (26% isolated as a pure compound); pale yellow oil; R_f 0.38 (Hexane/EtOAc 6:1); ^{1}H NMR (500 MHz, $CDCl_3$) δ 0.86 (dd, $J_{H–H}$ = 9.8 Hz, $J_{H–H}$ = 6.9 Hz, 6H), 1.64–1.71 (m, 1H), 2.03 (d, $J_{P–H}$ =13.2 Hz, 3H), 2.14–2.27 (m, 2H), 3.56 (s, 1H), 3.75–3.84 (m, 1H), 7.48–7.58 (m, 3H), 7.87–7.95 (m, 2H); ^{13}C NMR (126 MHz, $CDCl_3$) δ 17.3, 17.9, 22.4 (d, $J_{P–C}$ = 56.3 Hz), 34.4 (d, $J_{P–C}$ = 13.6 Hz), 37.7 (d, $J_{P–C}$ = 56.3 Hz), 71.6 (d, $J_{P–C}$ = 4.5 Hz), 128.7 (d, $J_{P–C}$ = 11.8 Hz), 130.5 (d, $J_{P–C}$ = 10.0 Hz), 131.7 (d, $J_{P–C}$ = 2.7 Hz), 132.2 (d, $J_{P–C}$ = 77.2 Hz); ^{31}P NMR (202 MHz, $CDCl_3$) δ 38.36; GC t_R = 9.45 min; GCMS (EI, 70 eV) *m/z* = 224 (M–H_2O) (17), 157 (11), 156 (100), 155 (39), 141 (23), 123 (13), 109 (11), 91 (18); HRMS (ESI–TOF) m/z: [M+H] Calcd for $C_{12}H_{19}OPS$ 243.0967; Found 243.0967.

(2-Hydroxy-3,3-dimethylbutyl)methylphenylphosphine sulfide (**9**). This compound was prepared according to the general procedure from dimethylphenylphosphine sulfide **4** (0.375 g, 2.21 mmol) and pivalaldehyde (0.285 g, 3.31 mmol) as a white solid; yield 0.474 g (84%). Isolated as a mixture of diastereomers (dr = 61.5:38.5).

Major diastereomer: R_f 0.77 (Hexane/AcOEt/iPrOH 10:1:1); ^{1}H NMR (500 MHz, $CDCl_3$) δ 0.93 (s, 9H), 2.03 (d, $J_{P–H}$ =12.9 Hz, 3H), 2.09–2.20 (m, 1H), 2.23–2.36 (m, 1H), 3.48 (bs, 1H), 3.78–3.86 (m, 1H), 7.49–7.59 (m, 3H), 7.86–7.95 (m, 2H); ^{13}C NMR (126 MHz, $CDCl_3$) δ 20.6 (d, $J_{P–C}$ = 56.3 Hz), 25.3, 35.2 (d, $J_{P–C}$ = 11.8 Hz), 36.5 (d, $J_{P–C}$ = 56.3 Hz), 74.5 (d, $J_{P–C}$ = 4.5 Hz), 128.6 (d, $J_{P–C}$ = 11.8 Hz), 130.1 (d, $J_{P–C}$ = 10.0 Hz), 131.5 (d, $J_{P–C}$ = 2.7 Hz),

132.7 (d, $J_{P–C}$ = 79.0 Hz); ^{31}P NMR (202 MHz, $CDCl_3$) δ 38.08; GC t_R = 10.76 min; GCMS (EI, 70 eV) *m/z* = 256 (2), 157 (11), 156 (100), 155(29), 141 (17), 78 (23), 77 (10), 63 (11).

Minor diastereomer: R_f 0.77 (Hexane/AcOEt/iPrOH 10:1:1); ^{1}H NMR (500 MHz, $CDCl_3$) δ 0.85 (s, 9H), 2.04 (d, $J_{P–H}$ =12.9 Hz, 3H), 2.09–2.21 (m, 1H), 2.23–2.37 (m, 1H), 3.48 (bs, 1H), 3.57–3.67 (m, 1H), 7.48–7.60 (m, 2H), 7.85–7.96 (m, 3H); ^{13}C NMR (126 MHz, $CDCl_3$) δ 22.2 (d, $J_{P–C}$ = 56.3 Hz), 25.2, 35.0 (d, $J_{P–C}$ = 11.8 Hz), 35.9 (d, $J_{P–C}$ = 56.3 Hz), 74.5 (d, $J_{P–C}$ = 4.5 Hz), 128.5 (d, $J_{P–C}$ =.8 Hz), 130.4 (d, $J_{P–C}$ = 10.0 Hz), 131.5 (d, $J_{P–C}$ = 2.7 Hz), 131.9 (d, $J_{P–C}$ = 78.1 Hz); ^{31}P NMR (202 MHz, $CDCl_3$) δ 39.33; GC t_R = 10.68 min; GCMS (EI, 70 eV) *m/z* = 256 (2), 157 (11), 156 (100), 155(29), 141 (17), 78 (24), 77 (11), 63 (11).

HRMS (ESI–TOF) m/z (the analysis has been performed for diastereomeric mixture): [M+H] Calcd for $C_{13}H_{21}OPS$ 257.1111; Found 257.1123.

(2-Hydroxy-2-phenylethyl)methylphenylphosphine sulfide (10). This compound was prepared according to the general procedure from dimethylphenylphosphine sulfide **4** (0.549 g, 3.22 mmol) and benzaldehyde (0.513 g, 4.84 mmol) as a white solid; yield 0.628 g (71%). Isolated as a mixture of diastereomers (dr = 60:40).

Major diastereomer: R_f 0.36 ($CHCl_3$); ^{1}H NMR (500 MHz, $CDCl_3$) δ 2.06 (d, $J_{P–H}$ = 13.6 Hz, 3H), 2.34–2.41 (m, 1H), 2.56–2.66 (m, 1H), 3.38 (bs, 1H), 5.13–5.20 (m, 1H), 7.23–7.27 (m, 1H), 7.27–7.41 (m, 4H), 7.48–7.59 (m, 3H), 7.86–7.97 (m, 2H); ^{13}C NMR (126 MHz, $CDCl_3$) δ 22.3 (d, $J_{P–C}$ = 58.1 Hz), 43.4 (d, $J_{P–C}$ = 52.7 Hz), 69.7 (d, $J_{P–C}$ = 3.6 Hz), 125.4, 127.7, 128.7 (d, $J_{P–C}$ = 11.8 Hz), 128.8, 130.5 (d, $J_{P–C}$ = 10.0 Hz), 131.8 (d, $J_{P–C}$ = 2.7 Hz), 132.2 (d, $J_{P–C}$ = 78.1 Hz), 143.8 (d, $J_{P–C}$ = 13.6 Hz); ^{31}P NMR (202 MHz, $CDCl_3$) δ 36.98; GC t_R = 13.71 min; GCMS (EI, 70 eV) *m/z* = 276 (M) (4), 157 (11), 156 (100), 141 (22), 123 (12), 121 (18), 109 (10), 91 (12), 78 (32), 77 (21), 63 (15).

Minor diastereomer: R_f 0.36 ($CHCl_3$); ^{1}H NMR (500 MHz, $CDCl_3$) δ 2.03 (d, $J_{P–H}$ = 12.9 Hz, 3H), 2.45–2.53 (m, 1H), 2.54–2.66 (m, 1H), 3.38 (bs, 1H), 5.26–5.33 (m, 1H), 7.23–7.27 (m, 1H), 7.28–7.40 (m, 4H), 7.48–7.59 (m, 3H), 7.86–7.96 (m, 2H); ^{13}C NMR (126 MHz, $CDCl_3$) δ 21.2 (d, $J_{P–C}$ = 56.3 Hz), 43.6 (d, $J_{P–C}$ = 52.7 Hz), 69.9 (d, $J_{P–C}$ = 3.6 Hz), 125.7, 127.9, 128.6, 128.7 (d, $J_{P–C}$ = 11.8 Hz), 130.2 (d, $J_{P–C}$ = 10.9 Hz), 131.8 (d, $J_{P–C}$ = 2.7 Hz), 132.6 (d, $J_{P–C}$ = 79.0 Hz), 143.5 (d, $J_{P–C}$ = 12.7 Hz); ^{31}P NMR (202 MHz, $CDCl_3$) δ 35.54; GC t_R = 13.84 min; GCMS (EI, 70 eV) *m/z* = 276 (M) (4), 157 (11), 156 (100), 141 (22), 123 (12), 121 (17), 109 (10), 91 (12), 78 (32), 77 (21), 63 (14).

HRMS (ESI–TOF) m/z (the analysis has been performed for diastereomeric mixture): [M+H] Calcd for $C_{15}H_{17}OPS$ 277.0812; Found 257.0810.

(2-Cyclohexyl-2-hydroxyethyl)methylphenylphosphine sulfide (11). This compound was prepared according to the general procedure from dimethylphenylphosphine sulfide **4** (0.275 g, 1.62 mmol) and cyclohexanal (0.272 g, 2.43 mmol) as a colorless oil; yield 0.411 g (89%). Isolated as a mixture of diastereomers (dr = 57:43).

Major diastereomer: R_f 0.47 ($CHCl_3$); ^{1}H NMR (500 MHz, $CDCl_3$) δ 0.90–1.25 (m, 6H), 1.36–1.45 (m, 1H), 1.59–1.83 (m, 4H), 2.01 (d, $J_{P–H}$ = 12.6 Hz, 3H), 2.16–2.33 (m, 2H), 3.43 (bs, 1H), 3.91–4.00 (m, 1H), 7.47–7.57 (m, 3H), 7.85–7.93 (m, 2H); ^{13}C NMR (126 MHz, $CDCl_3$) δ 21.0 (d, $J_{P–C}$ = 55.4 Hz), 26.0 (d, $J_{P–C}$ = 9.1 Hz), 26.3, 27.7, 28.4, 38.5 (d, $J_{P–C}$ = 56.3 Hz), 44.5 (d, $J_{P–C}$ = 11.8 Hz), 71.3 (d, $J_{P–C}$ = 4.5 Hz), 128.7 (d, $J_{P–C}$ = 11.8 Hz), 130.2 (d, $J_{P–C}$ = 10.0 Hz), 131.7 (d, $J_{P–C}$ = 2.7 Hz), 132.8 (d, $J_{P–C}$ = 79.0 Hz); ^{31}P NMR (202 MHz, $CDCl_3$) δ 37.13; GC t_R = 13.51 min; GCMS (EI, 70 eV) *m/z* = 264 (M–H_2O) (9), 157 (16), 156 (100), 155 (27), 141 (14), 91 (13), 78 (21), 77 (11), 63 (10), 55 (10).

Minor diastereomer: R_f 0.47 ($CHCl_3$); ^{1}H NMR (500 MHz, $CDCl_3$) δ 0.90–1.27 (m, 6H), 1.28–1.36 (m, 1H), 1.60–1.82 (m, 4H), 2.02 (d, J_{P-H} = 13.6 Hz, 3H), 2.16–2.33 (m, 2H), 3.43 (bs, 1H), 3.75–3.83 (m, 1H), 7.45–7.58 (m, 3H), 7.85–7.93 (m, 2H); ^{13}C NMR (126 MHz, $CDCl_3$) δ 22.4 (d, J_{P-C} = 56.3 Hz), 26.0 (d, J_{P-C} = 8.2 Hz), 26.3, 27.8, 28.6, 38.0 (d, J_{P-C} = 56.3 Hz), 44.4 (d, J_{P-C} = 12.7 Hz), 71.1 (d, J_{P-C} = 3.6 Hz), 128.7 (d, J_{P-C} = 11.8 Hz), 130.5 (d, J_{P-C} = 10.0 Hz), 131.6 (d, J_{P-C} = 2.7 Hz), 132.2 (d, J_{P-C} = 78.1 Hz); ^{31}P NMR (202 MHz, $CDCl_3$) δ 38.38; GC t_R = 13.57 min; GCMS (EI, 70 eV) *m/z* = 264 (M–H_2O) (10), 157 (18), 156 (100), 155 (26), 141 (13), 91 (11), 78 (20), 77 (11), 63 (10), 55 (10).

HRMS (ESI–TOF) m/z (the analysis has been performed for diastereomeric mixture): [M+H] Calcd for $C_{15}H_{23}OPS$ 283.1273; Found 283.1280.

(2-Hydroxy-2-methylbutyl)methylphenylphosphine sulfide (12). This compound was prepared according to the general procedure from dimethylphenylphosphine sulfide **4** (0.204 g, 1.20 mmol) and butan-2-one (0.129 g, 1.79 mmol); yield of two diastereomers 0.264 g (91%), (dr = 52:48).

Major diastereomer: Yield 37%; 0,033g (11% isolated as a pure compound); colorless oil; R_f 0.79 (Hexane/AcOEt/iPrOH, 5:1:1); ^{1}H NMR (500 MHz, $CDCl_3$) δ 0.92 (t, J_{H-H} = 7.4 Hz, 3H), 1.09 (s, 3H), 1.50–1.68 (m, 2H), 1.99 (d, J_{P-H} = 12.9 Hz, 3H), 2.36–2.46 (m, 2H), 4.63 (s, 1H), 7.46–7.58 (m, 3H), 7.86–7.99 (m, 2H); ^{13}C NMR (126 MHz, $CDCl_3$) δ 8.0, 23.8 (d, J_{P-C} = 57.2 Hz), 27.4 (d, J_{P-C} = 6.4 Hz), 37.2 (d, J_{P-C} = 10.0 Hz), 42.7 (d, J_{P-C} = 55.4 Hz), 73.0 (d, J_{P-C} = 4.5 Hz), 128.7 (d, J_{P-C} = 11.8 Hz), 130.3 (d, J_{P-C} = 10.9 Hz), 131.6 (d, J_{P-C} = 2.7 Hz), 133.0 (d, J_{P-C} = 78.1 Hz); ^{31}P NMR (202 MHz, $CDCl_3$) δ 33.37; GC t_R = 10.59 min; GCMS (EI, 70 eV) *m/z* = 242 (M) (8), 224 (M–H_2O) (26), 170 (12), 156 (51), 155(100), 109

(13), 91 (21); HRMS (ESI–TOF) m/z: [M+H] Calcd for $C_{12}H_{19}OPS$ 243.0970; Found 243.0967.

Minor diastereomer: Yield 36%; 0.106 g (4% isolated as a pure compound); white solid; mp 56.5–58.2 °C; R_f 0.74 (Hexane/AcOEt/iPrOH, 5:1:1); ^{1}H NMR (500 MHz, $CDCl_3$) δ 0.72 (t, J_{H-H} = 7.4 Hz, 3H), 1.35 (d, J_{H-H} = 1.9 Hz, 3H), 1.40–1.49 (m, 1H), 1.53–1.63 (m, 1H), 2.03 (d, J_{P-H} = 12.9 Hz, 3H), 2.34–2.48 (m, 2H), 4.50–4.72 (m, 1H), 7.46–7.59 (m, 3H), 7.88–7.97 (m, 2H); ^{13}C NMR (126 MHz, $CDCl_3$) δ 8.1, 23.6 (d, J_{P-C} = 57.2 Hz), 28.2 (d, J_{P-C} = 9.1 Hz), 36.0 (d, J_{P-C} = 8.2 Hz), 43.4 (d, J_{P-C} = 53.6 Hz), 73.3 (d, J_{P-C} = 4.5 Hz), 128.7 (d, J_{P-C} = 12.7 Hz), 130.3 (d, J_{P-C} = 10.9 Hz), 131.6 (d, J_{P-C} = 3.6 Hz), 133.3 (d, J_{P-C} = 78.1 Hz); ^{31}P NMR (202 MHz, $CDCl_3$) δ 33.14; GC t_R = 10.60 min; GCMS (EI, 70 eV) *m/z* = 242 (M) (4), 224 (M–H_2O) (26), 170 (12), 156 (52), 155(100), 109 (13), 91 (21); HRMS (ESI–TOF) m/z: [M+H] Calcd for $C_{12}H_{19}OPS$ 243.0955; Found 243.0967.

(2-Hydroxy-2-phenylpropyl)methylphenylphosphine sulfide (13). This compound was prepared according to the general procedure from dimethylphenylphosphine sulfide **4** (0.392 g, 1.37 mmol) and acetophenone (0.415 g, 3.46 mmol); yield of two diastereomers 0.618 g (92%), (dr = 62.5:37.5).

Major diastereomer: Yield 61%; (0,331g, 49% isolated as a pure compound); white solid; mp 129.3–130.2 °C; R_f 0.38 ($CHCl_3$); ^{1}H NMR (500 MHz, $CDCl_3$) δ 1.34 (d, J_{P-H} = 13.2 Hz, 3H), 1.57 (d, J_{H-H} = 2.5 Hz, 3H), 2.52–2.61 (m, 1H), 2.76–2.84 (m, 1H), 5.61 (bs, 1H), 7.27–7.33 (m, 1H), 7.37–7.43 (m, 2H), 7.47–7.59 (m, 5H), 7.82–7.90 (m, 2H); ^{13}C NMR (126 MHz, $CDCl_3$) δ 21.2 (d, J_{P-C} = 55.4 Hz), 33.0 (d, J_{P-C} = 10.0 Hz), 46.3 (d, J_{P-C} = 52.7 Hz), 74.3 (d, J_{P-C} = 4.5 Hz), 125.1, 127.1, 128.3, 128.7 (d, J_{P-C} = 12.7 Hz), 130.0 (d, J_{P-C} = 10.0 Hz), 131.7 (d, J_{P-C} = 2.7 Hz), 133.2 (d, J_{P-C} = 79.9 Hz), 146.7 (d, J_{P-C} = 5.4 Hz); ^{31}P NMR (202 MHz,

$CDCl_3$) δ 34.03; GC t_R = 13.36 min; GCMS (EI, 70 eV) m/z = 290 (6) (M), 272 (M−H_2O) (3), 233 (10), 232 (64), 231 (19), 170 (15), 156 (16), 155 (100), 137 (12), 123 (19), 121 (26), 109 (23), 91 (41), 78 (16) 77 (36), 63 (15) 51 (14); HRMS (ESI−TOF) m/z: [M+H] Calcd for $C_{16}H_{19}OPS$ 291.0975; Found 291.0967.

Minor diastereomer: Yield 31%; (0,137g, 20% isolated as a pure compound); white solid; mp 158.0–159.9 °C; R_f 0.32 ($CHCl_3$); ^1H NMR (500 MHz, $CDCl_3$) δ 1.64 (d, $J_{H–H}$ = 2.5 Hz, 3H), 1.97 (d, $J_{P–H}$ = 12.9 Hz, 3H), 2.67−2.75 (m, 1H), 2.84−2.95 (m, 1H), 5.38 (bs, 1H), 6.94−7.00 (m, 3H), 7.12−7.18 (m, 2H), 7.22−7.27 (m, 2H), 7.31−7.40 (m, 1H), 7.41−7.52 (m, 2H); ^{13}C NMR (126 MHz, $CDCl_3$) δ 23.1 (d, $J_{P–C}$ = 57.2 Hz), 33.4 (d, $J_{P–C}$ = 10.0 Hz), 45.8 (d, $J_{P–C}$ = 55.4 Hz), 74.3 (d, $J_{P–C}$ = 4.5 Hz), 125.0, 126.5, 127.5, 128.2 (d, $J_{P–C}$ = 11.8 Hz), 130.2 (d, $J_{P–C}$ = 10.9 Hz), 131.0 (d, $J_{P–C}$ = 2.7 Hz), 131.5 (d, $J_{P–C}$ = 78.1 Hz), 145.2 (d, $J_{P–C}$ = 5.4 Hz); ^{31}P NMR (202 MHz, $CDCl_3$) δ 32.89; GC t_R = 13.16 min; GCMS (EI, 70 eV) m/z = 290 (6) (M), 272 (M−H_2O) (3), 233 (12), 232 (72), 231 (24), 170 (14), 156 (16), 155 (100), 137 (13), 123 (20), 121 (28), 109 (25), 91 (44), 78 (18) 77 (41), 63 (16) 51 (15); HRMS (ESI−TOF) m/z: [M+H] Calcd for $C_{16}H_{19}OPS$ 291.0972; Found 291.0968.

(2-Hydroxy-2-methylhexyl)methylphenylphosphine sulfide (14). This compound was prepared according to the general procedure from dimethylphenylphosphine sulfide **4** (0.203 g, 1.19 mmol) and hexan-2-one (0.179 g, 1.79 mmol); yield of two diastereomers 0.244 g (76%), (dr = 58:42).

Major diastereomer: Yield 34%; (0,043g, 14% isolated as a pure compound); colorless oil; R_f 0.36 ($CHCl_3$); ^1H NMR (500 MHz, $CDCl_3$) δ 0.71 (t, $J_{H–H}$ = 7.1 Hz, 3H), 0.83−0.99 (m, 2H), 1.01−1.12 (m, 1H), 1.13−1.24 (m, 1H), 1.34 (d, $J_{P–H}$ = 1.9 Hz, 3H), 1.37−1.45 (m, 1H), 1.46−1.55 (m, 1H), 2.02 (d, J_{P-H} = 12.9 Hz, 3H), 2.32−2.53 (m, 2H), 4.28 (bs, 1H), 7.46−7.58

(m, 3H), 7.88–7.97 (m, 2H); ^{13}C NMR (126 MHz, $CDCl_3$) δ 13.8, 22.8, 23.6 (d, $J_{P–C}$ = 58.1 Hz), 26.3, 28.9 (d, $J_{P–C}$ = 9.1 Hz), 43.2 (d, $J_{P–C}$ = 7.3 Hz), 43.5 (d, $J_{P–C}$ = 54.5 Hz), 73.2 (d, $J_{P–C}$ = 4.5 Hz), 128.7 (d, $J_{P–C}$ = 11.8 Hz), 130.4 (d, $J_{P–C}$ = 10.9 Hz), 131.6 (d, $J_{P–C}$ = 2.7 Hz), 133.2 (d, $J_{P–C}$ = 78.1 Hz); ^{31}P NMR (202 MHz, $CDCl_3$) δ 33.06; GC t_R = 11.40 min; GCMS (EI, 70 eV) *m/z* = 270 (2) (M), 253 (M–H_2O) (4), 252 (23),170 (14), 157 (17),156 (91),155 (100), 123 (10),121 (10), 109 (13), 97 (14), 91 (22),78 (13), 77 (16),63 (11); HRMS (ESI–TOF) m/z: [M+H] Calcd for $C_{14}H_{23}OPS$ 271.1289; found 271.1280.

Minor diastereomer: Yield 42%; (0,074g, 23% isolated as a pure compound); white solid; mp 82.2–84.0 °C; R_f 0.33 ($CHCl_3$); ^{1}H NMR (500 MHz, $CDCl_3$) δ 0.90 (t, $J_{H–H}$ = 6.6 Hz, 3H), 1.10 (s, 3H), 1.26–1.40 (m, 4H), 1.46–1.64 (m, 2H), 1.98 (d, $J_{P–H}$ = 12.9 Hz, 3H), 2.36–2.47 (m, 2H), 4.14 (bs, 1H), 7.47–7.58 (m, 3H), 7.87–7.98 (m, 2H); ^{13}C NMR (126 MHz, $CDCl_3$) δ 14.0, 23.0, 23.8 (d, $J_{P–C}$ = 58.1 Hz), 25.8, 28.0 (d, $J_{P–C}$ = 6.4 Hz), 43.3 (d, $J_{P–C}$ = 54.5 Hz), 44.6 (d, $J_{P–C}$ = 10.0 Hz), 72.9 (d, $J_{P–C}$ = 4.5 Hz), 128.7 (d, $J_{P–C}$ = 11.8 Hz), 130.3 (d, $J_{P–C}$ = 10.9 Hz), 131.6 (d, $J_{P–C}$ = 3.6 Hz), 133.1 (d, $J_{P–C}$ = 77.2 Hz); ^{31}P NMR (202 MHz, $CDCl_3$) δ 33.31; GC t_R = 11.35 min; GCMS (EI, 70 eV) *m/z* = 270 (5)(M), 253 (M–H_2O) (4), 252 (24),170 (14), 157 (18),156 (92),155 (100), 123 (12),121 (11), 109 (14), 91 (25),78 (14), 77 (17),63 (12); HRMS (ESI–TOF) m/z: [M+H] Calcd for $C_{14}H_{23}OPS$ 271.1270; Found 271.1280.

(2-Hydroxy-2-methylpentyl)methylphenylphosphine sulfide (15). This compound was prepared according to the general procedure from dimethylphenylphosphine sulfide **4** (0.199 g, 1.17 mmol) and pentan-2-one (0.197 g, 1.75 mmol) as a white solid; yield 0.253 g (84%). Isolated as a mixture of diastereomers (dr = 51:49); R_f 0.38 ($CHCl_3$).

Major diastereomer: ^{1}H NMR (500 MHz, $CDCl_3$) δ 0.63 (t, J_{H-H} = 7.3 Hz, 3H), 0.93–1.03 (m, 1H), 1.16–1.29 (m, 1H), 1.33 (d, J_{H-H} = 1.6 Hz, 3H), 1.35–1.56 (m, 2H), 2.01 (d, J_{P-H} = 12.9 Hz, 3H), 2.33–2.49 (m, 2H), 4.37 (bs, 1H), 7.47–7.56 (m, 3H), 7.88–7.96 (m, 2H); ^{13}C NMR (126 MHz, $CDCl_3$) δ 14.0, 17.3, 23.5 (d, J_{P-C} = 57.2 Hz), 28.8 (d, J_{P-C} = 9.1 Hz), 43.5 (d, J_{P-C} = 50.0 Hz), 45.7 (d, J_{P-C} = 7.3 Hz), 73.1 (d, J_{P-C} = 4.5 Hz), 128.6 (d, J_{P-C} = 11.8 Hz), 130.3 (d, J_{P-C} = 10.0 Hz), 131.6 (d, J_{P-C} = 2.7 Hz), 133.1 (d, J_{P-C} = 78.1 Hz); ^{31}P NMR (202 MHz, $CDCl_3$) δ 33.15; GC t_R = 10.92 min; GC−MS (EI, 70 eV) *m/z* = 256 (3) (M), 238 (M–H_2O) (27), 170 (13), 157 (15), 156 (88), 155 (100), 123 (11), 121 (11), 109 (15), 91 (24), 78 (15), 77 (18), 63 (13).

Minor diastereomer: ^{1}H NMR (500 MHz, $CDCl_3$) δ 0.90 (t, J_{H-H} = 6.9 Hz, 3H), 1.09 (s, 3H), 1.34–1.59 (m, 4H), 1.97 (d, J_{P-H} = 12.9 Hz, 3H), 2.30–2.48 (m, 2H), 4.37 (bs, 1H), 7.47–7.56 (m, 3H), 7.86–7.97 (m, 2H); ^{13}C NMR (126 MHz, $CDCl_3$) δ 14.3, 16.9, 23.8 (d, J_{P-C} = 56.3 Hz), 28.0 (d, J_{P-C} = 6.4 Hz), 43.2 (d, J_{P-C} = 52.7 Hz), 47.1 (d, J_{P-C} = 10.0 Hz), 72.8 (d, J_{P-C} = 4.5 Hz), 128.6 (d, J_{P-C} = 11.8 Hz), 130.3 (d, J_{P-C} = 10.0 Hz), 131.5 (d, J_{P-C} = 2.7 Hz), 133.1 (d, J_{P-C} = 77.2 Hz); ^{31}P NMR (202 MHz, $CDCl_3$) δ 33.32; GC t_R = 10.95 min; GCMS (EI, 70 eV) *m/z* = 256 (4) (M), 238 (M–H_2O) (25), 170 (13), 157 (14), 156 (86), 155 (100), 121 (10), 109 (14), 91 (23), 83 (18), 78 (14), 77 (18), 63 (12).

HRMS (ESI–TOF) m/z (the analysis has been performed for diastereomeric mixture): [M+H] Calcd for $C_{13}H_{21}OPS$ 257.1121; Found 257.1123.

(2-Hydroxy-2,3-dimethylbutyl)methylphenylphosphine sulfide (16). This compound was prepared according to the general procedure from dimethylphenylphosphine sulfide **4** (0.968 g, 5.69 mmol) and 3-methylbutan-2-one (0.735 g, 8.53 mmol) as a white solid; yield 1.240 g (85%). Isolated as a mixture of diastereomers (dr = 53:47); R_f 0.57 ($CHCl_3$).

Major diastereomer: ^{1}H NMR (500 MHz, $CDCl_3$) δ 0.89 (d, $J_{H–H}$ = 6.6 Hz, 6H), 1.05 (s, 3H), 1.71–1.80 (m, 1H), 1.98 (d, $J_{P–H}$ = 12.9 Hz, 3H), 2.38–2.46 (m, 2H), 4.25 (bs, 1H), 7.44–7.58 (m, 3H), 7.85–8.00 (m, 2H); ^{13}C NMR (126 MHz, $CDCl_3$) δ 16.7, 17.3, 23.9 (d, $J_{P–C}$ = 68.1 Hz), 24.9 (d, $J_{P–C}$ = 5.4 Hz), 39.7 (d, $J_{P–C}$ = 9.1 Hz), 40.2 (d, $J_{P–C}$ = 55.4 Hz), 75.1 (d, $J_{P–C}$ = 5.4 Hz), 128.7 (d, $J_{P–C}$ = 4.5 Hz), 130.3 (d, $J_{P–C}$ = 10.9 Hz), 131.5 (d, $J_{P–C}$ = 4.5 Hz), 133.1 (d, $J_{P–C}$ = 76.3 Hz); ^{31}P NMR (202 MHz, $CDCl_3$) δ 34.11; GC t_R = 10.89 min; GCMS (EI, 70 eV) *m/z* = 238 (M–H_2O) (25), 170 (13), 157 (11), 156 (60), 155 (100), 138 (20), 123 (12), 121 (12), 109 (14), 91 (28), 78 (10), 77 (18), 63 (12).

Minor diastereomer: ^{1}H NMR (500 MHz, $CDCl_3$) δ 0.63 (d, $J_{H–H}$ = 6.9 Hz, 3H), 0.94 (d, $J_{H–H}$ = 7.3 Hz, 3H), 1.34 (d, $J_{H–H}$ = 0.9 Hz, 3H), 1.80–1.87 (m, 1H), 2.06 (d, $J_{P–H}$ = 12.9 Hz, 3H), 2.30–2.39 (m, 2H), 4.05–4.50 (bs, 1H), 7.48–7.57 (m, 3H), 7.88–7.97 (m, 2H); ^{13}C NMR (126 MHz, $CDCl_3$) δ 16.7, 17.4, 23.4 (d, $J_{P–C}$ = 66.3 Hz), 24.6 (d, $J_{P–C}$ = 6.4 Hz), 38.6 (d, $J_{P–C}$ = 8.2 Hz), 41.7 (d, $J_{P–C}$ = 54.5 Hz), 75.4 (d, $J_{P–C}$ = 5.4 Hz), 128.6 (d, $J_{P–C}$ = 4.5 Hz), 130.3 (d, $J_{P–C}$ = 10.9 Hz), 131.6 (d, $J_{P–C}$ = 4.5 Hz), 133.5 (d, $J_{P–C}$ = 79.0 Hz); ^{31}P NMR (202 MHz, $CDCl_3$) δ 33.89; GC t_R = 10.92 min; GCMS (EI, 70 eV) *m/z* = 238 (M–H_2O) (25), 170 (14), 157 (10), 156 (57), 155 (100), 109 (13), 91 (22), 78 (10), 77 (17), 63 (12).

HRMS (ESI–TOF) m/z (the analysis has been performed for diastereomeric mixture): [M+H] Calcd for $C_{13}H_{21}OPS$ 257.1131; Found 257.1123.

(2-Hydroxy-2,3,3-trimethylbutyl)methylphenylphosphine sulfide (17). This was prepared according to the general procedure from dimethylphenylphosphine sulfide **4** (1.128 g, 6.63 mmol) and 3,3-dimethylbutan-2-one (0.995 g, 9.94 mmol) as a white solid; yield 1.511 g (84%). Isolated as a mixture of diastereomers (dr = 55:45); R_f 0.74 ($CHCl_3$).

Major diastereomer: ^{1}H NMR (500 MHz, $CDCl_3$) δ 0.93 (s, 9H), 1.12 (d, $J_{H\text{–}H}$ = 0.6 Hz, 3H), 1.98 (d, $J_{P\text{–}H}$ = 12.9 Hz, 3H), 2.31–2.61 (m, 2H), 4.39 (bs, 1H), 7.47–7.58 (m, 3H), 7.85–8.00 (m, 2H); ^{13}C NMR (126 MHz, $CDCl_3$) δ 23.6 (d, $J_{P\text{–}C}$ = 3.6 Hz), 23.9 (d, $J_{P\text{–}C}$ = 58.1 Hz), 24.7, 38.8 (d, $J_{P\text{–}C}$ = 8.2 Hz), 39.1 (d, $J_{P\text{–}C}$ = 56.3 Hz), 76.5 (d, $J_{P\text{–}C}$ = 5.4 Hz), 128.7 (d, $J_{P\text{–}C}$ = 11.8 Hz), 130.4 (d, $J_{P\text{–}C}$ = 10.0 Hz), 131.5 (d, $J_{P\text{–}C}$ = 2.7 Hz), 133.2 (d, $J_{P\text{–}C}$ = 76.3 Hz); ^{31}P NMR (202 MHz, $CDCl_3$) δ 35.11; GC t_R = 11.08 min; GCMS (EI, 70 eV) *m/z* = 255 (M−CH_3) (2),214 (5), 213 (47), 157 (6), 156 (17), 155 (100), 121 (5), 109 (6), 91 (10).

Minor diastereomer: ^{1}H NMR (500 MHz, $CDCl_3$) δ 0.92 (s, 9H), 1.55 (d, $J_{H\text{–}H}$ = 0.6 Hz, 3H), 2.11 (d, $J_{P\text{–}H}$ = 12.9 Hz, 3H), 2.31–2.60 (m, 2H), 4.34 (bs, 1H), 7.48–7.58 (m, 3H), 7.86–7.98 (m, 2H); ^{13}C NMR (126 MHz, $CDCl_3$) δ 22.9 (d, $J_{P\text{–}C}$ = 55.4 Hz), 23.9 (d, $J_{P\text{–}C}$ = 2.7 Hz), 24.8, 39.0 (d, $J_{P\text{–}C}$ = 9.1 Hz), 40.4 (d, $J_{P\text{–}C}$ = 54.5 Hz), 76.4 (d, $J_{P\text{–}C}$ = 6.4 Hz), 128.7 (d, $J_{P\text{–}C}$ = 11.8 Hz), 130.1 (d, $J_{P\text{–}C}$ = 10.0 Hz), 131.5 (d, $J_{P\text{–}C}$ = 2.7 Hz), 134.0 (d, $J_{P\text{–}C}$ = 80.8 Hz); ^{31}P NMR (202 MHz, $CDCl_3$) δ 35.73; GC t_R = 11.19 min; GCMS (EI, 70 eV) *m/z* = 255 (M−CH_3) (1), 214 (5), 213 (45), 157 (6), 156 (18), 155 (100), 121 (5), 109 (6), 91 (10).

HRMS (ESI–TOF) m/z (the analysis has been performed for diastereomeric mixture): [M+H] Calcd for $C_{14}H_{23}OPS$ 271.1287; found 271.1280.

(2-Hydroxy-2,4,4-trimethylpentyl)methylphenylphosphine sulfide (18). This compound was prepared according to the general procedure from dimethylphenylphosphine sulfide **4** (0.194 g, 1.14 mmol) and 4,4-dimethylpentan-2-one (0.195 g, 1.71 mmol) as a white solid; yield 0.279 g (86%). Isolated as a mixture of diastereomers (dr = 53:47); R_f 0.41 ($CHCl_3$).

Major diastereomer: ^{1}H NMR (500 MHz, $CDCl_3$) δ 1.02 (s, 9H), 1.21 (s, 3H), 1.61–1.74 (m, 2H), 1.97 (d, $J_{P\text{–}H}$ = 12.6 Hz, 3H), 2.33–2.58 (m, 2H), 4.22 (bs, 1H), 7.46–7.55 (m, 3H), 7.87–7.97 (m, 2H); ^{13}C NMR (126 MHz, $CDCl_3$) δ 24.0 (d, $J_{P\text{–}C}$ = 57.2 Hz), 29.7 (d, $J_{P\text{–}C}$ =

6.4 Hz), 31.5 (d, $J_{P–C}$ = 12.7 Hz), 31.6, 45.7 (d, $J_{P–C}$ = 52.7 Hz), 56.6 (d, $J_{P–C}$ = 9.1 Hz), 74.5 (d, $J_{P–C}$ = 5.4 Hz), 128.6 (d, $J_{P–C}$ = 11.8 Hz), 130.3 (d, $J_{P–C}$ = 10.0 Hz), 131.5 (d, $J_{P–C}$ = 2.7 Hz), 133.2 (d, $J_{P–C}$ = 77.2 Hz); ^{31}P NMR (202 MHz, $CDCl_3$) δ 32.87; GC t_R = 11.42 min; GCMS (EI, 70 eV) *m/z* = 266 (M–H_2O) (5), 213 (13), 210 (10), 209 (70), 170 (14), 156 (13), 155 (100), 109 (11), 91 (18), 77 (13), 57 (18).

Minor diastereomer: ^{1}H NMR (500 MHz, $CDCl_3$) δ 0.90 (s, 9H), 1.12–1.19 (m, 1H), 1.48 (s, 3H), 1.63–1.70 (m, 1H), 2.00 (d, $J_{P–H}$ = 12.3 Hz, 3H), 2.31–2.59 (m, 2H), 4.22 (bs, 1H), 7.46–7.56 (m, 3H), 7.82–8.00 (m, 2H); ^{13}C NMR (126 MHz, $CDCl_3$) δ 23.6 (d, $J_{P–C}$ = 56.3 Hz), 30.3 (d, $J_{P–C}$ = 8.2 Hz), 31.4, 31.5 (d, $J_{P–C}$ = 12.7 Hz), 46.7 (d, $J_{P–C}$ = 52.7 Hz), 55.1 (d, $J_{P–C}$ = 7.3 Hz), 74.7 (d, $J_{P–C}$ = 5.5 Hz), 128.6 (d, $J_{P–C}$ = 11.8 Hz), 130.2 (d, $J_{P–C}$ = 10.0 Hz), 131.5 (d, $J_{P–C}$ = 2.7 Hz), 133.5 (d, $J_{P–C}$ = 78.1 Hz); ^{31}P NMR (202 MHz, $CDCl_3$) δ 32.95; GC t_R = 11.36 min; GCMS (EI, 70 eV) *m/z* = 266 (M–H_2O) (4), 213 (14), 210 (10), 209 (74), 170 (13), 156 (13), 155 (100), 109 (12), 91 (21), 77 (14), 57 (22).

HRMS (ESI–TOF) m/z (the analysis has been performed for diastereomeric mixture): [M+H] Calcd for $C_{15}H_{25}OPS$ 285.1423; Found 285.1436.

(2-Hydroxy-2-methylpropyl)methylphenylphosphine sulfide (19). This compound was prepared according to the general procedure from dimethylphenylphosphine sulfide **4** (0.194 g, 1.14 mmol) and acetone (0.099 g, 1.71 mmol) as a sticky solid; yield 0.229 g (88%); R_f 0.38 ($CHCl_3$); ^{1}H NMR (500 MHz, $CDCl_3$) δ 1.17 (s, 3H), 1.39 (d, $J_{H–H}$ = 2.2 Hz, 3H), 2.01 (d, $J_{P–H}$ = 12.9 Hz, 3H), 2.46 (d, $J_{P–H}$ = 10.4 Hz, 2H), 4.71 (bs, 1H), 7.49–7.59 (m, 3H), 7.87–7.98 (m, 2H); ^{13}C NMR (126 MHz, $CDCl_3$) δ 23.7 (d, $J_{P–C}$ = 57.2 Hz), 30.7 (d, $J_{P–C}$ = 7.3 Hz), 32.0 (d, $J_{P–C}$ = 9.1 Hz), 44.9 (d, $J_{P–C}$ = 53.6 Hz), 70.9 (d, $J_{P–C}$ = 4.5 Hz), 128.7 (d, $J_{P–C}$ = 11.8 Hz), 130.3 (d, $J_{P–C}$ = 10.0 Hz), 131.6 (d, $J_{P–C}$ = 2.7 Hz), 133.1 (d, $J_{P–C}$ = 77.2 Hz);

^{31}P NMR (202 MHz, $CDCl_3$) δ 32.98; GC t_R = 10.24 min; GCMS (EI, 70 eV) *m/z* = 228 (M) (21), 170 (13), 155 (100), 137 (11), 123 (15), 121 (13), 109 (18), 91 (32); HRMS (ESI−TOF) m/z: [M+H] Calcd for $C_{11}H_{17}OPS$ 229.0820; Found 229.0810.

(2-Ethyl-2-hydroxybutyl)methylphenylphosphine sulfide (20). This compound was prepared according to the general procedure from dimethylphenylphosphine sulfide **4** (1.013 g, 5.95 mmol) and pentan-3-one (0.769 g, 8.93 mmol) as acolorless solid; yield 1.196 g (78%); mp 38.9−40.6°C; R_f 0.54 (Hexane/AcOEt/iPrOH 10:1:1); ^{1}H NMR (500 MHz, $CDCl_3$) δ 0.62 (t, $J_{H\text{–}H}$ = 7.4 Hz, 3H), 0.90 (t, $J_{H\text{–}H}$ = 7.6 Hz, 3H), 1.38−1.48 (m, 1H), 1.55−1.72 (m, 3H), 2.02 (d, $J_{P\text{–}H}$ = 12.9 Hz, 3H), 2.40 (d, $J_{P\text{–}H}$ = 11.0 Hz, 2H), 4.02 (bs, 1H), 7.47−7.58 (m, 3H), 7.89−7.97 (m, 2H); ^{13}C NMR (126 MHz, $CDCl_3$) δ 7.7, 23.7 (d, $J_{P\text{–}C}$ = 57.2 Hz), 32.0 (d, $J_{P\text{–}C}$ = 7.3 Hz), 32.6 (d, $J_{P\text{–}C}$ = 9.1 Hz), 41.3 (d, $J_{P\text{–}C}$ = 54.5 Hz), 75.4 (d, $J_{P\text{–}C}$ = 4.5 Hz), 128.7 (d, $J_{P\text{–}C}$ = 11.8 Hz), 130.3 (d, $J_{P\text{–}C}$ = 10.9 Hz), 131.5 (d, $J_{P\text{–}C}$ = 2.7 Hz), 133.3 (d, $J_{P\text{–}C}$ = 73.6 Hz); ^{31}P NMR (202 MHz, $CDCl_3$) δ 33.39; GC t_R = 11.00 min; GCMS (EI, 70 eV) *m/z* = 256 (2), 238 (M−H_2O) (21), 170 (13), 157 (11), 156 (58), 155 (100), 121 (10), 109 (14), 91 (23); HRMS (ESI−TOF) m/z: [M+H] Calcd for $C_{13}H_{21}OPS$ 257.1112; Found 257.1123.

(2-Hydroxy-2-isopropyl-3-methylbutyl)methylphenylphosphine sulfide (21) This compound was prepared according to the general procedure from dimethylphenylphosphine sulfide **4** (0.285 g, 1.7 mmol)) and 2,4-dimethylpentan-3-one (0.287 g, 2.6 mmol) as a colorless oil; yield 0.333 g (70%); R_f 0.74 ($CHCl_3$); ^{1}H NMR (500 MHz, $CDCl_3$) δ 0.74 (d, $J_{H\text{–}H}$ = 6.9 Hz, 3H), 0.94 (dd, $J_{H\text{–}H}$ = 6.9 Hz, $J_{H\text{–}H}$ = 4.9 Hz, 6H), 1.01 (d, $J_{H\text{–}H}$ = 6.9 Hz, 3H), 1.89−1.96 (m, 1H), 1.96−2.03 (m, 1H), 2.11 (d, $J_{P\text{–}H}$ = 13.2 Hz, 3H), 2.25−2.34 (m, 1H), 2.39−2.49 (m, 1H), 4.20 (bs, 1H), 7.45−7.58 (m, 3H), 7.85−8.00 (m, 2H); ^{13}C NMR (126

MHz, $CDCl_3$) δ 17.5, 17.7, 17.8, 17.9, 23.2 (d, $J_{P–C}$ = 57.2 Hz), 35.6 (d, $J_{P–C}$ = 3.6 Hz), 36.3 (d, $J_{P–C}$ = 3.6 Hz), 37.3 (d, $J_{P–C}$ = 53.6 Hz), 78.6 (d, $J_{P–C}$ = 5.4 Hz), 128.6 (d, $J_{P–C}$ = 11.8 Hz), 130.5 (d, $J_{P–C}$ = 10.0 Hz), 131.4 (d, $J_{P–C}$ = 2.7 Hz), 133.7 (d, $J_{P–C}$ = 78.1 Hz); ^{31}P NMR (202 MHz, $CDCl_3$) δ 36.18; GC t_R = 11.57 min; GC−MS (EI, 70 eV) m/z (%) = 266 (M–H_2O) (10), 241 (16), 223 (14), 170 (15), 156 (46), 155 (100), 138 (23), 123 (12), 121 (11), 109 (13), 91 (24), 77 (15), 71 (11), 69 (16), 55 (12); HRMS (ESI–TOF) m/z: [M+H] Calcd for $C_{15}H_{25}OPS$ 285.1428; found 285.1436.

((1-Hydroxy)cyclopentylmethyl)methylphenylphosphine sulfide (22). This compound was prepared according to the general procedure from dimethylphenylphosphine sulfide **4** (0.211 g, 1.24 mmol)) and cyclopentanone (0.156 g, 1.86 mmol) as a yellow solid; yield 0.228 g (72%); mp 87.1–88.4 °C; R_f 0.28 ($CHCl_3$); ^{1}H NMR (500 MHz, $CDCl_3$) δ 1.09–1.21 (m, 1H), 1.40–1.63 (m, 3H), 1.64–1.87 (m, 4H), 2.01 (d, $J_{P–H}$ = 13.2 Hz, 3H), 2.49–2.57 (m, 1H), 2.58–2.66 (m, 1H), 3.81 (bs, 1H), 7.48–7.59 (m, 3H), 7.87–7.99 (m, 2H); ^{13}C NMR (126 MHz, $CDCl_3$) δ 22.9, 23.4 (d, $J_{P–C}$ = 56.3 Hz), 23.3, 40.5 (d, $J_{P–C}$ = 6.4 Hz), 42.0 (d, $J_{P–C}$ = 8.2 Hz), 43.6 (d, $J_{P–C}$ = 53.6 Hz), 80.8 (d, $J_{P–C}$ = 5.4 Hz), 128.7 (d, $J_{P–C}$ = 11.8 Hz), 130.4 (d, $J_{P–C}$ = 10.0 Hz), 131.6 (d, $J_{P–C}$ = 3.6 Hz), 133.1 (d, $J_{P–C}$ = 77.2 Hz); ^{31}P NMR (202 MHz, $CDCl_3$) δ 33.58; GC t_R = 11.81 min; GCMS (EI, 70 eV) *m/z* = 254 (M) (2), 236 (M–H_2O) (28), 157 (21), 156 (64), 155 (100), 109 (13), 91 (20), 81 (18); HRMS (ESI–TOF) m/z: [M+H] Calcd for $C_{13}H_{19}OPS$ 255.0977; Found 255.0967.

((1-Hydroxy)cyclohexylmethyl)methylphenylphosphine sulfide (23). This compound was prepared according to the general procedure from dimethylphenylphosphine sulfide **4** (0.218 g, 1.28 mmol) and cyclohexanone (0.189 g, 1.92 mmol) as a pale yellow solid; yield 0.302 g

(88%); mp 80.5–81.7 °C; R_f 0.38 ($CHCl_3$); ^1H NMR (500 MHz, $CDCl_3$) δ 1.08–1.32 (m, 3H), 1.35–1.59 (m, 4H), 1.63–1.76 (m, 2H), 1.80–1.92 (m, 1H), 2.00 (d, J_{P-H} = 13.2 Hz, 3H), 2.34–2.49 (m, 2H), 3.74 (bs, 1 H), 7.47–7.58 (m, 3H), 7.87–8.00 (m, 2H); ^{13}C NMR (126 MHz, $CDCl_3$) δ 21.7, 21.9, 23.8 (d, J_{P-C} = 57.2 Hz), 25.3, 38.8 (d, J_{P-C} = 7.3 Hz), 40.1 (d, J_{P-C} = 9.1 Hz), 44.1 (d, J_{P-C} = 53.6 Hz), 72.1 (d, J_{P-C} = 5.4 Hz), 128.7 (d, J_{P-C} = 11.8 Hz), 130.3 (d, J_{P-C} = 10.0 Hz), 131.5 (d, J_{P-C} = 2.7 Hz), 133.4 (d, J_{P-C} = 77.2 Hz); ^{31}P NMR (202 MHz, $CDCl_3$) δ 32.66; GC t_R = 12.37 min; GCMS (EI, 70 eV) *m/z* = 250 (M–H_2O) (31), 170 (13), 157 (34), 156 (67), 155 (100), 121 (10), 109 (14), 95 (22), 91 (23); HRMS (ESI–TOF) m/z: [M+H] Calcd for $C_{14}H_{21}OPS$ 269.1116; Found 269.1123.

((1-Hydroxy)cycloheptylmethyl)methylphenylphosphine sulfide (24). This compound was prepared according to the general procedure from dimethylphenylphosphine sulfide **4** (0.199 g, 1.17 mmol) and cycloheptanone (0.197 g, 1.75 mmol) as apale yellow solid; yield 0.290 g (88%); mp 53.8–54.6 °C; R_f 0.43 ($CHCl_3$); ^1H NMR (500 MHz, $CDCl_3$) δ 1.08–1.19 (m, 1H), 1.29–1.41 (m, 2H), 1.41–1.61 (m, 6H), 1.63–1.74 (m, 2H), 1.76–1.85 (m, 1H), 2.00 (d, J_{P-H} = 12.9 Hz, 3H), 2.35–2.53 (m, 2H), 3.95 (bs, 1 H), 7.48–7.56 (m, 3H), 7.87–7.98 (m, 2H); ^{13}C NMR (126 MHz, $CDCl_3$) δ 21.7, 21.7, 23.8 (d, J_{P-C} = 56.3 Hz), 29.5, 29.7, 42.2 (d, J_{P-C} = 7.3 Hz), 43.7 (d, J_{P-C} = 9.1 Hz), 45.1 (d, J_{P-C} = 53.6 Hz), 76.1 (d, J_{P-C} = 5.4 Hz), 128.7 (d, J_{P-C} = 11.8 Hz), 130.3 (d, J_{P-C} = 10.9 Hz), 131.5 (d, J_{P-C} = 2.7 Hz), 133.3 (d, J_{P-C} = 77.2 Hz); ^{31}P NMR (202 MHz, $CDCl_3$) δ 32.98; GC t_R = 13.20 min; GCMS (EI, 70 eV) *m/z* = 264 (M–H_2O) (27), 170 (16), 157 (27), 156 (87), 155 (100), 123 (10), 121 (11), 109 (44), 108 (10), 91 (24); HRMS (ESI–TOF) m/z: [M+H] Calcd for $C_{15}H_{23}OPS$ 283.1289; Found 283.1280.

(2-Butyl-2-hydroxyhexyl)methylphenylphosphine sulfide (25). This compound was prepared according to the general procedure from dimethylphenylphosphine sulfide **4** (0.198 g, 1.16 mmol)) and nonan-5-one (0.248 g, 1.74 mmol) as a white solid; yield 0.283 g (78%); mp 53.9–54.8 °C; R_f 0.68 ($CHCl_3$); ^{1}H NMR (500 MHz, $CDCl_3$) δ 0.68 (t, J_{H-H} = 7.3 Hz, 3H), 0.71–0.89 (m, 3H), 0.91 (t, J_{H-H} = 7.3 Hz, 3H), 0.94–1.04 (m, 1H), 1.06–1.17 (m, 1H), 1.24–1.36 (m, 4H), 1.46–1.62 (m, 3H), 2.00 (d, J_{P-H} = 12.9 Hz, 3H), 2.36–2.49 (m, 2H), 3.69 (bs, 1H), 7.42–7.66 (m, 3H), 7.88–8.01 (m, 2H); ^{13}C NMR (126 MHz, $CDCl_3$) δ 13.8, 14.1, 22.8, 23.1, 23.7 (d, J_{P-C} = 57.2 Hz), 25.4, 26.1, 39.9 (d, J_{P-C} = 6.4 Hz), 40.8 (d, J_{P-C} = 9.1 Hz), 41.9 (d, J_{P-C} = 52.7 Hz), 75.1 (d, J_{P-C} = 4.5 Hz), 128.7 (d, J_{P-C} = 12.7 Hz), 130.5 (d, J_{P-C} = 10.0 Hz), 131.6 (d, J_{P-C} = 2.7 Hz), 133.2 (d, J_{P-C} = 77.2 Hz); ^{31}P NMR (202 MHz, $CDCl_3$) δ 33.18; GC t_R = 12.22 min; GCMS (EI, 70 eV) *m/z* = 294 (M–H_2O) (14), 255 (15), 170 (19), 157 (19), 156 (95), 155 (100), 141 (10), 140 (21), 139 (25), 138 (22), 124 (20),123 (14),121 (13), 109 (17), 91 (26), 85 (11); HRMS (ESI–TOF) m/z: [M+H] Calcd for $C_{17}H_{29}OPS$ 313.1734; Found 313.1749.

(S_P)-(2-Hydroxy-3-methylbutyl)methylphenylphosphine sulfide (S_P)-(8). To a cooled (–78°C) solution of (+)-sparteine (0.343 g, 1.46 mmol) in 4 mL Et_2O was added *n*-BuLi (1.6 mL, 1.46 mmol). After stirring for 40 min, dimethylphenylphosphine sulfide **4** (0.207 g, 1.22 mmol) was added as a solution in 15 mL Et_2O. After 2 h at –78°C, isobutyraldehyde (0.132 g, 1.83 mmol) was added to the reaction. The reaction was allowed to warm to room temperature and was stirred for 1 h. The reaction was quenched by addition of 1 M HCl (5 mL) and extracted with DCM (3 × 20 mL). The combined organic phases were dried over $MgSO_4$, filtered, and evaporated under reduced pressure. The residue was purified by flash column chromatography using $CHCl_3$ as eluent yielding a pale yellow oil; yield 0.211 g (70%);

Isolated as a mixture of diastereomers (dr = 53:47). HPLC (Chiralcel OJ–H, Hexane/iPrOH = 95:5, 0.5 mL/min) t_{R1} = 24.00 min, t_{R2} = 25.72 min, t_{R3} = 33.33 min, t_{R4} = 38.66 min (71% ee).

(S_P)-(2-Hydroxy-2,3,3-trimethylbutyl)methylphenylphosphine sulfide (S_P)-17. This compound was prepared as described for (S_P)-**8** using dimethylphenylphosphine sulfide **4** (0.110 g, 0.65 mmol), (+)-sparteine (0.182 g, 0.78 mmol), *n*-BuLi (0.48 mL, 0.78 mmol) and 3,3-dimethylbutan-2-one (0.097 g, 0.97 mmol). Yield 0.141 g (81%, single diastereomer). HPLC (Chiralcel OJ–H, Hexane/iPrOH = 95:5, 1.0 mL/min) t_{R1} = 14.11 min, t_{R2} = 18.61 min (65% ee).

(S_P)-(2-Hydroxy-2-methylpropyl)methylphenylphosphine sulfide (S_P)-(19). This compound was prepared as described for (S_P)-**8** using dimethylphenylphosphine sulfide **4** (0.292 g, 1.71 mmol), (+)-sparteine (0.442 g, 1.89 mmol), *n*-BuLi (1.6 mL, 2.57 mmol) and acetone (0.149 g, 2.57 mmol). Yield 0.147 g (38%). HPLC (Chiralcel OJ–H, Hexane/iPrOH = 90:10, 1.0 mL/min) t_{R1} = 13.86 min, t_{R2} = 19.75 min (67% ee).

(S_P)-(2-Ethyl-2-hydroxybutyl)methylphenylphosphine sulfide (S_P)-(20). This compound was prepared as described for (S_P)-**8** using dimethylphenylphosphine sulfide (**4**) (0.242 g, 1.42 mmol), (+)-sparteine (0.399 g, 1.71 mmol), *n*-BuLi (1.1 mL, 1.71 mmol) and pentan-3-one (0.184 g, 2.13 mmol). Yield 0.246 g (68%). HPLC (Chiralcel OJ–H, Hexane/iPrOH = 95:5, 1.0 mL/min) t_{R1} = 14.92 min, t_{R2} = 17.72 min (80% ee).

General procedure for the reaction of β-hydroxyalkylphosphine sulfides and β-mesyloxyalkylphosphine sulfides with $AlCl_3$

$AlCl_3$ (5 mmol) was added to a solution of substrate (1.0 mmol) in dichloroethane (5 mL). The reaction mixture was stirred at room temperature for 18 h. The mixture was quenched with water (5 mL) and 20% $NaOH_{aq}$ and extracted with DCM (3 × 10 mL). The combined organic phases were dried over $MgSO_4$, filtered, and evaporated under reduced pressure. The residue was purified by column chromatography using $CHCl_3$ as eluent.

(2-Methyl-3-mercaptobutyl)methylphenylphosphine oxide (29). This compound was prepared according to the general procedure from (2-hydroxy-2-methylbutyl)methylphenylphosphine sulfide **12** (0.052 g, 0.22 mmol) as a pale yellow oil; yield 0.039 g (74%); Isolated as a mixture of two diastereomers (dr = 51:49); R_f 0.44 ($CHCl_3$).
Major diastereomer: ^{1}H NMR (500 MHz, $CDCl_3$) δ 0.85 (d, $J_{H–H}$ = 6.6 Hz, 3H), 1.24 (d, $J_{H–H}$ = 6.9 Hz, 3H), 1.70 (d, $J_{P–H}$ = 12.6 Hz, 3H), 1.77–1.92 (m, 1H), 2.13–2.30 (m, 3H), 3.15–3.27 (m, 1H), 7.43–7.57 (m, 3H), 7.68–7.80 (m, 2H); ^{13}C NMR (126 MHz, $CDCl_3$) δ 15.9 (d, $J_{P–C}$ = 4.5 Hz), 17.7 (d, $J_{P–C}$ = 70.0 Hz), 22.1, 34.7, 35.4 (d, $J_{P–C}$ = 70.0 Hz), 41.2 (d, $J_{P–C}$ = 10.9 Hz), 128.6 (d, $J_{P–C}$ = 10.9 Hz), 129.9 (d, $J_{P–C}$ = 9.1 Hz), 131.5 (d, $J_{P–C}$ = 1.8 Hz), 133.6 (d, $J_{P–C}$ = 95.4 Hz); ^{31}P NMR (202 MHz, $CDCl_3$) δ 36.64; GC t_R = 10.90 min; GCMS (EI, 70 eV) *m/z* = 209 (M–SH) (12), 181 (32),155 (10), 154 (73), 140 (52), 139 (100), 125 (33), 92 (17), 91 (62), 77 (35), 51 (13), 47 (28).
Minor diastereomer: ^{1}H NMR (500 MHz, $CDCl_3$) δ 1.08 (d, $J_{P–H}$ = 6.9 Hz, 3H), 1.20 (d, $J_{P–H}$ = 6.9 Hz, 3H), 1.72 (d, $J_{P–H}$ = 12.8 Hz, 3H), 1.77–1.92 (m, 1H), 1.97–2.08 (m, 1H), 2.11–2.29 (m, 1H), 2.90–2.99 (m, 1H), 7.45–7.56 (m, 3H), 7.68–7.77 (m, 2H); ^{13}C NMR (126 MHz, $CDCl_3$) δ 16.4 (d, $J_{P–C}$ = 4.5 Hz), 17.2 (d, $J_{P–C}$ = 70.0 Hz), 21.6, 34.6, 36.0 (d, $J_{P–C}$ = 70.0 Hz),

41.6 (d, $J_{P–C}$ = 10.9 Hz), 128.6 (d, $J_{P–C}$ = 10.9 Hz), 130.1 (d, $J_{P–C}$ = 9.1 Hz), 131.6 (d, $J_{P–C}$ = 1.8 Hz), 134.2 (d, $J_{P–C}$ = 95.4 Hz); ^{31}P NMR (202 MHz, $CDCl_3$) δ 37.12; GC t_R = 10.85 min; GCMS (EI, 70 eV) *m/z* = 209 (M–SH) (11), 181 (38), 154 (73), 140 (53), 139 (100), 125 (32), 109 (11), 92 (16), 91 (63), 78 (13), 77 (35), 65 (10), 63 (13), 51 (13), 47 (32).

HRMS (ESI–TOF) m/z (the analysis has been performed for diastereomeric mixture): [M+H] Calcd for $C_{12}H_{19}OPS$ 243.0965; Found 243.0967.

(2-Methyl-3-mercaptohexyl)methylphenylphosphine oxide (30). This compound was prepared according to the general procedure from (2-hydroxy-2-methylhexyl)methylphenylphosphine sulfide **14** (0.047 g, 0.17 mmol) as a pale yellow oil; yield 0.044 g (94%); Isolated as a mixture of epimers; R_f 0.32 ($CHCl_3$); ^{1}H NMR (500 MHz, $CDCl_3$) (due to overlapping only selected peaks of epimers are described) δ 0.80 (t, $J_{H–H}$ = 7.3 Hz, 3H) and 0.86 (t, $J_{H–H}$ = 7.3 Hz, 3H), and 0.90 (t, J_{H-H} = 7.3 Hz, 3H), 0.92 (d, $J_{H–H}$ = 6.4 Hz, 3H), 0.96 (t, $J_{H–H}$ = 7.3 Hz, 3H), 1.03 (d, $J_{H–H}$ = 6.3 Hz, 3H) and 1.04 (d, $J_{H–H}$ = 6.6 Hz, 3H), 1.16–1.67 (m, 20H), 1.70 (d, $J_{P–H}$ = 12.6 Hz, 3H), 1.71 (d, $J_{P–H}$ = 11.7 Hz, 3H), 1.71 (d, $J_{P–H}$ = 12.6 Hz, 3H), and 1.73 (d, $J_{P–H}$ = 12.3 Hz, 3H), 1.79–2.05 (m, 4H), 2.12–2.32 (m, 4H), 2.53–2.76 (m, 2H), 2.89–3.11 (m, 2H), 7.40–7.56 (m, 12H), 7.66–7.79 (m, 8H); ^{13}C NMR (126 MHz, $CDCl_3$) δ 1.3 and 11.4, and 13.5, and 13.7, 15.3 (d, $J_{P–C}$ = 5.5 Hz) and 15.8 (d, $J_{P–C}$ = 5.5 Hz), 17.0 (d, $J_{P–C}$ = 69.0 Hz) and 17.4 (d, $J_{P–C}$ = 70.0 Hz), and 17.7 (d, $J_{P–C}$ = 70.8 Hz), 20.6 (d, $J_{P–C}$ = 6.4 Hz), 20.7 and 20.8, 21.0 (d, $J_{P–C}$ = 7.3 Hz), 26.2, 32.6, 32.8, 33.2, 36.8 (d, $J_{P–C}$ = 69.0 Hz), 37.6 and 38.4, and 38.6, 39.3 (d, $J_{P–C}$ = 69.0 Hz), 40.4, 46.7 (d, $J_{P–C}$ = 9.1 Hz) and 47.1 (d, $J_{P–C}$ = 9.4 Hz), and 47.2 (d, $J_{P–C}$ = 10.9 Hz), 128.6 (d, $J_{P–C}$ = 10.9 Hz), 129.9 (d, $J_{P–C}$ = 10.0 Hz), 131.5 (d, $J_{P–C}$ = 2.9 Hz); ^{31}P NMR (202 MHz, $CDCl_3$) δ 36.69 and 36.81, and 37.06; GC(compound 1) t_R = 11.55 min; GCMS (EI, 70 eV) *m/z* = 270 (1), 237 (M–SH)

(22), 181 (35), 155 (13), 154 (100), 141 (11), 140 (43), 139 (86), 125 (28), 92 (15), 91 (52), 77 (24), 55 (19), 47 (24); (compound 2) t_R = 11.57 min; GCMS (EI, 70 eV) *m/z* = 270 (1), 237 (M–SH) (22), 181 (28), 155 (12), 154 (100), 141 (10), 140 (42), 139 (81), 125 (22), 92 (15), 91 (51), 77 (20), 55 (16), 47 (20); (compound 3) t_R = 11.64 min; GCMS (EI, 70 eV) *m/z* = 270 (1), 237 (M–SH) (40), 181 (13), 173 (11), 154 (23), 141 (12), 140 (100), 139 (86), 125 (34), 91 (32), 77 (23), 55 (17), 47 (21); (compound 4) t_R = 11.67 min; GCMS (EI, 70 eV) *m/z* = 270 (0.34), 237 (M–SH) (39), 181 (12), 173 (12), 154 (25), 141 (13), 140 (100), 139 (87), 125 (33), 91 (32), 77 (25), 55 (17), 47 (21); HRMS (ESI–TOF) m/z (the analysis has been performed for the mixture of epimers): [M+H] Calcd for $C_{14}H_{23}OPS$ 271.1276; Found 271.1280.

(2-Methyl-3-mercaptopentyl)methylphenylphosphine oxide (31). This compound was prepared according to the general procedure from (2-hydroxy-2-methylpentyl)methylphenylphosphine sulfide **15** (0.053 g, 0.21 mmol) as a pale yellow oil; yield 0.043 g (81%); Isolated as a mixture of epimers; R_f 0.26 ($CHCl_3$); ^{1}H NMR (500 MHz, $CDCl_3$) (due to overlapping only selected peaks of epimers are described) δ 0.80 (d, $J_{H–H}$ = 6.3 Hz, 3H), 0.91 (t, $J_{H–H}$ = 7.6 Hz, 6H) and 0.97 (t, $J_{H–H}$ = 7.9 Hz, 3H), 1.05 (d, $J_{H–H}$ = 6.6 Hz, 3H), 1.21 (d, $J_{H–H}$ = 6.6 Hz, 3H) and 1.31 (d, $J_{H–H}$ = 6.8 Hz, 3H), 1.38–1.50 (m, 4H), 1.52–1.65 (m, 4H), 1.70 (d, $J_{P–H}$ = 12.6 Hz, 3H) and 1.72 (m, $J_{P–H}$ = 12.3 Hz, 3H) and 1.73 (d, $J_{H–H}$ = 12.6 Hz, 3H), 1.79–2.04 (m, 6H), 2.09–2.37 (m, 6H), 2.53–2.62 (m, 5H), 2.86–2.94 (m, 2H), 7.45–7.55 (m, 12H), 7.66–7.84 (m, 8H); ^{13}C NMR (126 MHz, $CDCl_3$) δ 12.5, 15.2 (d, $J_{P–C}$ = 5.5 Hz) and 15.7 (d, $J_{P–C}$ = 5.5 Hz), 17.1 (d, $J_{P–C}$ = 69.0 Hz) and 17.5 (d, $J_{P–C}$ = 70.8 Hz) and 17.6 (d, $J_{P–C}$ = 70.0 Hz), 20.7 (d, $J_{P–C}$ = 7.3 Hz) and 21.1 (d, $J_{P–C}$ = 6.4 Hz), 26.4, 26.6, 26.6, 29.4, 29.7, 32.9, 33.0, 33.1, 36.9 (d, $J_{P–C}$ = 68.1 Hz), 49.3 (d, $J_{P–C}$ = 9.1 Hz) and

49.4 (d, $J_{P–C}$ = 9.1 Hz) and 49.6 (d, $J_{P–C}$ = 9.08 Hz), 128.6 (d, $J_{P–C}$ = 10.9 Hz), 130.0 (d, $J_{P–C}$ = 11.8 Hz), 131.6 (d, $J_{P–C}$ = 1.8 Hz); ^{31}P NMR (202 MHz, $CDCl_3$) δ 36.72 and 36.96 and 37.09 and 37,78; GC (compound 1) t_R = 11.22 min; GCMS (EI, 70 eV) *m/z* = 256 (1), 223 (M–SH) (25), 181 (24), 154 (56), 141 (13), 140 (85), 139 (100), 125 (31), 92 (13), 91 (46), 77 (25), 47 (22); (compound 2) t_R = 11.26 min; GCMS (EI, 70 eV) *m/z* = 256 (1), 223 (M–SH) (25), 181 (25), 154 (76), 141 (11), 140 (68), 139 (100), 125 (26), 92 (14), 91 (51), 77 (23), 47 (21); (compound 3) t_R = 11.29 min; GCMS (EI, 70 eV) *m/z* = 256 (1), 223 (M–SH) (30), 181 (39), 154 (48), 140 (48), 139 (100), 125 (23), 92 (11), 91 (41), 77 (26), 47 (20); (compound 4) t_R = 11.36 min; GCMS (EI, 70 eV) *m/z* = 256 (1), 223 (M–SH) (34), 181 (37), 154 (46), 140 (42), 139 (100), 125 (22), 92 (12), 91 (41), 77 (23), 55 (11), 47 (20).

HRMS (ESI–TOF) m/z (the analysis has been performed for the mixture of epimers): [M+H] Calcd for $C_{13}H_{21}OPS$ 257.1111; Found 257.1123.

(2,3-Dimethyl-3-mercaptobutyl)methylphenylphosphine oxide (32). This compound was prepared according to the general procedure from (2-mesyloxy-3,3-dimethylbutyl)methylphenylphosphine sulfide **60** (0.051 g, 0.15 mmol) running the reaction for 48 h. The product was obtained as a pale yellow oil; yield 0.034 g (88%); Isolated as a mixture of diastereomers (dr = 52:48); R_f 0.72 ($CHCl_3$:MeOH 15:1).

Major diastereomer: ^{1}H NMR (500 MHz, $CDCl_3$) δ 1.17 (d, $J_{H–H}$ = 6.6 Hz, 3H), 1.23 (s, 3H), 1.28 (s, 3H), 1.36 (s, 1H), 1.72 (d, $J_{P–H}$ = 12.6 Hz, 3H), 1.76–1.88 (m, 1H), 2.09–2.18 (m, 1H), 2.26–2.39 (m, 1H), 7.44–7.56 (m, 3H), 7.68–7.76 (m, 2H); ^{13}C NMR (126 MHz, $CDCl_3$) δ 16.7, 17.8 (d, $J_{P–C}$ = 70.0 Hz), 31.6, 31.7, 34.8 (d, $J_{P–C}$ = 69.0 Hz), 40.1 (d, $J_{P–C}$ = 3.6 Hz), 48.9 (d, $J_{P–C}$ = 13.6 Hz), 128.5 (d, $J_{P–C}$ = 10.9 Hz), 130.3 (d, $J_{P–C}$ = 9.1 Hz), 131.6 (d, $J_{P–C}$ = 2.7 Hz), 133.2 (d, $J_{P–C}$ = 95.4 Hz); ^{31}P NMR (202 MHz, $CDCl_3$) δ 37.90; GC t_R = 11.15 min;

GCMS (EI, 70 eV) *m/z* = 223(M–SH) (25), 207 (11), 181 (48), 141 (15), 140 (100), 139 (97), 125 (52), 91 (35), 83 (11), 77 (31), 55 (13), 51 (11), 47 (30).

Minor diastereomer: ^{1}H NMR (500 MHz, $CDCl_3$) δ 0.95 (d, $J_{H\text{–}H}$ = 6.6 Hz, 3H), 1.21 (s, 3H), 1.42 (s, 3H), 1.54 (s, 1H), 1.71 (d, $J_{H\text{–}H}$ = 12.6 Hz, 3H), 1.78–1.87 (m, 1H), 2.24–2.41 (m, 2H), 7.42–7.56 (m, 3H), 7.68–7.76 (m, 2H); ^{13}C NMR (126 MHz, $CDCl_3$) δ 16.6 (d, $J_{P\text{–}C}$ = 70.0 Hz), 16.7, 28.4, 28.6, 35.2 (d, $J_{P\text{–}C}$ = 40.0 Hz), 40.1 (d, $J_{P\text{–}C}$ = 4.5 Hz), 49.1 (d, $J_{P\text{–}C}$ = 13.6 Hz), 128.6 (d, $J_{P\text{–}C}$ = 11.8 Hz), 130.3 (d, $J_{P\text{–}C}$ = 9.1 Hz), 131.6 (d, $J_{P\text{–}C}$ = 2.7 Hz), 134.7 (d, $J_{P\text{–}C}$ = 95.4 Hz);^{31}P NMR (202 MHz, $CDCl_3$) δ 36.63. GC t_R = 11.29 min; GCMS (EI, 70 eV) *m/z* = 223 (M–SH) (29), 181 (42), 154 (10), 141 (17), 140 (100), 139 (91), 125 (54), 91 (28), 83 (11), 77 (34), 55 (16), 51 (11), 47 (30).

HRMS (ESI–TOF) m/z (the analysis has been performed for diastereomeric mixture): [M+H] Calcd for $C_{13}H_{21}OPS$ 257.1112; Found 257.1123.

(2,2,3-Trimethyl-3-mercaptobutyl)methylphenylphosphine oxide (33). This compound was prepared according to the general procedure from (2-hydroxy-2,3,3-trimethylbutyl)methylphenylphosphine sulfide **17** (0.055 g, 0.20 mmol) as a pale yellow oil; yield 0.041 g (74%); R_f 0.29 ($CHCl_3$); ^{1}H NMR (500 MHz, $CDCl_3$) δ 0.96 (s, 3H), 1.34 (s, 3H), 1.37 (s, 6H), 1.69 (d, $J_{P\text{–}H}$ = 12.6 Hz, 3H), 2.22–2.32 (m, 1H), 2.32–2.42 (m, 1H), 2.80 (bs, 1H), 7.42–7.53 (m, 3H), 7.70–7.78 (m, 2H); ^{13}C NMR (126 MHz, $CDCl_3$) δ 19.7 (d, $J_{P\text{–}C}$ = 71.8 Hz), 22.9 (d, $J_{P\text{–}C}$ = 2.7 Hz), 23.3, 28.1, 28.3, 38.4 (d, $J_{P\text{–}C}$ = 70.0 Hz), 41.2 (d, $J_{P\text{–}C}$ = 3.6 Hz), 53.7 (d, $J_{P\text{–}C}$ = 13.6 Hz), 128.6 (d, $J_{P\text{–}C}$ = 10.9 Hz), 129.9 (d, $J_{P\text{–}C}$ = 9.1 Hz), 131.2 (d, $J_{P\text{–}C}$ = 2.6 Hz), 135.6 (d, $J_{P\text{–}C}$ = 95.4 Hz); ^{31}P NMR (202 MHz, $CDCl_3$) δ 36.58; GC t_R = 11.58 min; GCMS (EI, 70 eV) *m/z* = 237 (M–SH) (5), 195 (44), 140 (29), 139 (100), 91 (10), 77 (12); HRMS (ESI–TOF) m/z: [M+H] Calcd for $C_{14}H_{23}OPS$ 271.1276; Found 271.1280.

The rearrangement of (S_P)–(2–hydroxy–2,3,3–trimethyl-butyl)methylphenylphosphine sulfide (S_P)–**17** (0.141 g, 0.52 mmol) afforded a product as a pale yellow oil; yield 0.108 g (77%); HPLC (Chiralcel AS–H, Hexane/iPrOH = 9:1, 1.0 mL/min) t_{R1} = 8.97 min, t_{R2} = 12.99 min (22% ee).

(2-Methylprop-1-enyl)(methylphenyl)phosphine oxide (34). This compound was prepared according to the general procedure from (2-hydroxy-2,4,4-trimethylpentyl)methylphenylphosphine sulfide **18** (0.021 g, 0.07 mmol) as a white sticky solid; yield 0.014 g (96%); R_f 0.36 (AcOEt/MeOH 9:1). ^{1}H NMR (500 MHz, $CDCl_3$) δ 1.69 (d, $J_{P\text{–}H}$ = 13.2 Hz, 3H), 1.91 (s, 3H), 1.98 (dd, $J_{H\text{–}H}$ = 2.5 Hz, $J_{H\text{–}H}$ = 0.9 Hz, 3H), 5.67 (d, $J_{P\text{–}H}$ = 26.2 Hz, 1H), 7.41–7.51 (m, 3H), 7.68–7.76 (m, 2H); ^{13}C NMR (126 MHz, $CDCl_3$) δ 18.8 (d, $J_{P\text{–}C}$ = 74.5 Hz), 21.3 (d, $J_{P\text{–}C}$ = 8.2 Hz), 28.4 (d, $J_{P\text{–}C}$ = 17.3 Hz), 117.9 (d, $J_{P\text{–}C}$ = 102.6 Hz), 128.4 (d $J_{P\text{–}C}$ = 11.8 Hz), 129.9 (d, $J_{P\text{–}C}$ = 10.0 Hz), 131.1 (d, $J_{P\text{–}C}$ = 2.7 Hz), 135.6 (d, $J_{P\text{–}C}$ = 101.7 Hz), 158.7; ^{31}P NMR (202 MHz, $CDCl_3$) δ 24.52; GC t_R = 10.19 min; GCMS (EI, 70 eV): m/z = 194 (M) (32), 193 (100), 179 (19), 139 (59), 131 (16), 130 (18), 129 (26), 125 (15), 121 (12), 117 (13), 115 (26), 91 (41), 78 (17), 77 (62), 69 (11), 65 (17), 63 (28), 55 (10), 53 (19), 51 (60), 50 (19), 47 (66). Anal. Calcd. for $C_{11}H_{15}OP$: C 68.03%, H 7.78%, found: C 68.09%, H 7.86%; Analytical data are in accordance with those reported in the literature [7].

(2-Ethyl-3-mercaptobutyl)methylphenylphosphine oxide (37). This compound was prepared according to the general procedure from (2-ethyl-2-hydroxybutyl)methylphenylphosphine sulfide **20** (0.048 g, 0.19 mmol) as a pale yellow oil; yield 0.029 g (59%). Isolated as a mixture of epimers; R_f 0.35 ($CHCl_3$); ^{1}H NMR (500 MHz,

$CDCl_3$) (due to overlapping only selected peaks of epimers are described) δ 0.74 (t, $J_{H–H}$ = 7.4 Hz, 3H) and 0.78 (t, $J_{H–H}$ = 7.4 Hz, 3H) and 0.89 (t, $J_{H–H}$ = 7.3 Hz, 3H), 1.10 (d, $J_{H–H}$ = 6.9 Hz, 3H) and 1.19 (d, $J_{H–H}$ = 6.9 Hz, 3H) and 1.22 (d, $J_{H–H}$ = 6.9 Hz, 3H) and 1.33 (d, $J_{H–H}$ = 6.9 Hz, 3H), 1.35-1.67 (m, 8H), 1.74 (d, $J_{P–H}$ = 12.6 Hz, 3H) and 1.75 (d, $J_{P–H}$ = 12.6 Hz, 3H), 1.79–2.36 (m, 8H), 2.43 (bs, 3H), 3.19 (bs, 1H) and 3.30 (bs, 1H) and 3.49 (bs, 1H), 7.46–7.60 (m, 12H), 7.65–7.82 (m, 8H); ^{13}C NMR (126 MHz, $CDCl_3$) δ 11.0 and 11.2 and 11.6 and 11.7, 16.6 (d, $J_{P–C}$ = 70.0 Hz) and 17.0 (d, $J_{P–C}$ = 71.8 Hz) and 17.4 (d, $J_{P–C}$ = 70.0 Hz), 20.3 and 20.9 and 22.5, 23.8 (d, $J_{P–C}$ = 7.3 Hz) and 23.9 (d, $J_{P–C}$ = 6.4 Hz), 31.8 (d, $J_{P–C}$ = 69.0 Hz) and 32.3 (d, $J_{P–C}$ = 70.7 Hz), 37.3 (d, $J_{P–C}$ = 11.8 Hz) and 37.5 (d, $J_{P–C}$ = 10.9 Hz) and 37.6 (d, $J_{P–C}$ = 9.1 Hz) and 38.0 (d, $J_{P–C}$ = 7.3 Hz), 41.4 (d, $J_{P–C}$ = 3.6 Hz) and 41.9 (d, $J_{P–C}$ = 3.6 Hz), 128.6 (d, $J_{P–C}$ = 11.8 Hz), 130.0 (d, $J_{P–C}$ = 9.1 Hz) and 130.1 (d, $J_{P–C}$ = 10.0 Hz) and 130.2 (d, $J_{P–C}$ = 9.1 Hz), 131.6 (d, $J_{P–C}$ = 4.5 Hz) and 131.7 (d, $J_{P–C}$ = 3.6 Hz), 133.4 (d, $J_{P–C}$ = 95.4 Hz) and 134.1 (d, $J_{P–C}$ = 98.1 Hz); ^{31}P NMR (202 MHz, $CDCl_3$) δ 37.40 and 37.93 and 38.06; GC (compound 1) t_R = 11.02 min; GCMS (EI, 70 eV) *m/z* = 256 (1), 223 (M–SH) (10), 195 (50), 155 (10), 154 (92), 141 (10), 140 (39), 139 (100), 125 (24), 92 (16) 91 (58), 77 (25), 55 (13), 47 (23); (compound 2) t_R = 11.06 min; GCMS (EI, 70 eV) *m/z* = 256 (1), 223 (M–SH) (12), 195 (45), 155 (11), 154 (100), 141 (11), 140 (34), 139 (98), 125 (22), 92 (18) 91 (60), 77 (24), 55 (12), 47 (22); (compound 3) t_R = 11.10 min; GCMS (EI, 70 eV) *m/z* = 256 (1), 223 (M–SH) (19), 195 (54), 154 (60), 140 (31), 139 (100), 125 (20), 92 (12) 91 (43), 77 (22), 55 (11), 47 (20); HRMS (ESI–TOF) m/z (the analysis has been performed for the mixture of epimers): [M+H] Calcd for $C_{13}H_{21}OPS$ 257.1112; Found 257.1123.

(2-Isopropyl-3-methyl-3-mercaptobutyl)methylphenylphosphine oxide (38). This compound was prepared according to the general procedure from (2-hydroxy-2-isopropyl-3-

methylbutyl)methylphenylphosphine sulfide **21** (0.054 g, 0.19 mmol) as a pale yellow oil; yield 0.032 g (59%); R_f 0.31 ($CHCl_3$); Isolated as a mixture of diastereomers (dr = 52:48).

Major diastereomer: ^{1}H NMR (500 MHz, $CDCl_3$) δ 1.00 (d, $J_{H–H}$ = 6.6 Hz, 3H), 1.04 (d, $J_{H–H}$ = 6.9 Hz, 3H), 1.24 (s, 3H), 1.52 (s, 3H), 1.75 (d, $J_{H–H}$ = 12.9 Hz, 3H), 1.80 (s, 1H), 1.91–2.34 (m, 4H), 7.46–7.55 (m, 3H), 7.71–7.79 (m, 2H); ^{13}C NMR (126 MHz, $CDCl_3$) δ 17.7 (d, $J_{P–C}$ = 70.8 Hz), 19.2, 23.6, 28.5, 29.9 (d, $J_{P–C}$ = 69.0 Hz), 30.6, 33.4, 49.2 (d, $J_{P–C}$ = 3.6 Hz), 50.0 (d, $J_{P–C}$ = 9.1 Hz), 128.5 (d, $J_{P–C}$ = 10.9 Hz), 130.1 (d, $J_{P–C}$ = 9.1 Hz), 131.4 (d, $J_{P–C}$ = 3.6 Hz), 135.0 (d, $J_{P–C}$ = 95.4 Hz); ^{31}P NMR (202 MHz, $CDCl_3$) δ 37.09; GC t_R = 11.50 min; GCMS (EI, 70 eV) *m/z* = 251 (M–SH) (36), 210 (13), 209 (91), 207 (10), 154 (16), 141 (26), 140 (99),139 (100), 125 (43), 91 (23), 77 (23), 69 (27), 55 (10), 47 (26).

Minor diastereomer: ^{1}H NMR (500 MHz, $CDCl_3$) δ 0.73 (d, $J_{H–H}$ = 7.3 Hz, 3H), 0.87 (d, $J_{H–H}$ = 6.9 Hz, 3H), 1.32 (s, 3H), 1.39 (s, 3H), 1.76 (d, $J_{H–H}$ = 12.3 Hz, 3H), 1.93–2.33 (m, 4H), 1.97 (s, 1H), 7.45–7.56 (m, 3H), 7.70–7.79 (m, 2H); ^{13}C NMR (126 MHz, $CDCl_3$) δ 17.0 (d, $J_{P–C}$ = 70.8 Hz), 19.1, 23.4, 28.3, 29.8 (d, $J_{P–C}$ = 68.1 Hz), 30.1, 33.7, 49.3 (d, $J_{P–C}$ = 5.5 Hz), 50.4 (d, $J_{P–C}$ = 9.1 Hz), 128.6 (d, $J_{P–C}$ = 10.9 Hz), 130.4 (d, $J_{P–C}$ = 9.1 Hz), 131.4 (d, $J_{P–C}$ = 3.6 Hz), 134.0 (d, $J_{P–C}$ = 95.4 Hz); ^{31}P NMR (202 MHz, $CDCl_3$) δ 36.56; GC t_R = 11.59 min; GCMS (EI, 70 eV) *m/z* = 251 (M–SH) (35), 210 (12), 209 (78), 207 (11), 154 (15), 141 (25), 140 (100),139 (99), 125 (43), 91 (23), 77 (23), 69 (26), 55 (11), 47 (26).

HRMS (ESI–TOF) m/z (the analysis has been performed for diastereomeric mixture): [M+H] Calcd for $C_{15}H_{25}OPS$ 285.1422; Found 257.1436.

(((2-Mercapto)cyclopentyl)methyl)methylphenylphosphine oxide (39). This compound was prepared according to the general procedure from ((1-hydroxy)cyclopentylmethyl)methylphenylphosphine sulfide **22** (0.067 g, 0.26 mmol) as a pale

yellow oil; yield 0.026 g (40%); R_f 0.43 ($CHCl_3$); Isolated only as a mixture of two diastereomers (56:44).

Major diastereomer: ^{1}H NMR (500 MHz, $CDCl_3$) δ 1.16–1.35 (m, 1H), 1.44–1.72 (m, 4H), 1.75 (d, J_{P-H} = 12.6 Hz, 3H), 1.85–1.95 (m, 1H), 1.97–2.11 (m, 2H), 2.25–2.45 (m, 2H), 3.49 (bs, 1H), 7.46–7.59 (m, 3H), 7.69–7.79 (m, 2H); ^{13}C NMR (126 MHz, $CDCl_3$) δ 17.4 (d, J_{P-C} = 68.1 Hz), 21.4, 29.5 (d, J_{P-C} = 6.4 Hz), 33.7 (d, J_{P-C} = 68.1 Hz), 35.3, 38.9, 45.2 (d, J_{P-C} = 9.1 Hz), 128.7 (d, J_{P-C} = 11.8 Hz), 130.0 (d, J_{P-C} = 9.1 Hz), 131.7 (d, J_{P-C} = 2.7 Hz); ^{31}P NMR (202 MHz, $CDCl_3$) δ 37.65.

Minor diastereomer: ^{1}H NMR (500 MHz, $CDCl_3$) δ 1.16–1.35 (m, 1H), 1.44–1.72 (m, 4H), 1.75 (d, J_{P-H} = 12.6 Hz, 3H), 1.78–1.84 (m, 1H), 1.97–2.11 (m, 2H), 2.25–2.45 (m, 2H), 3.18 (bs, 1H), 7.46–7.59 (m, 3H), 7.69–7.79 (m, 2H); ^{13}C NMR (126 MHz, $CDCl_3$) δ 17.2 (d, J_{P-C} = 68.1 Hz), 21.2, 29.9 (d, J_{P-C} = 6.4 Hz), 33.4 (d, J_{P-C} = 68.2 Hz), 35.3, 38.9, 44.8 (d, J_{P-C} = 8.2 Hz), 128.7 (d, J_{P-C} = 11.8 Hz), 130.1 (d, J_{P-C} = 8.2 Hz), 131.8 (d, J_{P-C} = 1.8 Hz); ^{31}P NMR (202 MHz, $CDCl_3$) δ 37.82.

GC t_R = 12.14 min; GCMS (EI, 70 eV) *m/z* = 254 (M) (2), 221 (M–SH) (34), 155 (14), 154 (100), 141 (13), 140 (71),139 (93), 125 (40), 115 (16), 92 (17), 91 (59), 79 (18), 77 (39), 51 (12), 47 (31). HRMS (ESI–TOF) m/z (the analysis has been performed for diastereomeric mixture): [M+H] Calcd for $C_{13}H_{19}OPS$ 255.0967; Found 255.0967.

(((2-Mercapto)cyclohexyl)methyl)methylphenylphosphine oxide (40). This compound was prepared according to the general procedure from ((1-hydroxy)cyclohexylmethyl)methylphenylphosphine sulfide **23** (0.076 g, 0.28 mmol) as a pale yellow oil; yield 0.045 g (60%); R_f 0.38 ($CHCl_3$); Isolated as a mixture of epimers (80:20).

Major epimer: ^{1}H NMR (500 MHz, $CDCl_3$) (due to overlapping only selected peaks of epimer is described) δ 1.15–1.65 (m, 8H), 1.71 (d, $J_{P\text{–}H}$ = 12.9 Hz, 3H), 1.78–2.00 (m, 2H), 2.08–2.26 (m, 2H), 3.39–3.50 (m, 1H), 7.44–7.56 (m, 3H), 7.66–7.78 (m, 2H); ^{13}C NMR (126 MHz, $CDCl_3$) δ 17.6 (d, $J_{P\text{–}C}$ = 70.0 Hz), 20.4, 24.6, 28.2 (d, $J_{P\text{–}C}$ = 6.4 Hz), 33.0 (d, $J_{P\text{–}C}$ = 71.8 Hz), 34.1, 36.0, 43.0 (d, $J_{P\text{–}C}$ = 8.2 Hz), 128.6 (d, $J_{P\text{–}C}$ = 10.9 Hz), 129.8 (d, $J_{P\text{–}C}$ = 9.1 Hz), 131.5 (d, $J_{P\text{–}C}$ = 2.7 Hz), 134.4 (d, $J_{P\text{–}C}$ = 94.5 Hz); ^{31}P NMR (202 MHz, $CDCl_3$) δ 36.81.

Minor epimer: ^{1}H NMR (500 MHz, $CDCl_3$) (due to overlapping only selected peaks of epimer is described) δ 1.15–1.65 (m, 8H), 1.70 (d, $J_{P\text{–}H}$ = 12.9 Hz, 3H), 1.78–2.00 (m, 2H), 2.08–2.26 (m, 2H), 3.07–3.12 (m, 1H), 7.44–7.56 (m, 3H), 7.66–7.78 (m, 2H); ^{13}C NMR (126 MHz, $CDCl_3$) δ 17.6 (d, $J_{P\text{–}C}$ = 70.0 Hz), 20.5, 24.7, 28.7 (d, $J_{P\text{–}C}$ = 8.2 Hz), 33.0 (d, $J_{P\text{–}C}$ = 69.9 Hz), 34.0, 36.0, 42.6 (d, $J_{P\text{–}C}$ = 8.2 Hz), 128.6 (d, $J_{P\text{–}C}$ = 10.9 Hz), 130.0 (d, $J_{P\text{–}C}$ = 10.0 Hz), 131.6 (d, $J_{P\text{–}C}$ = 2.7 Hz) 133.9 (d, $J_{P\text{–}C}$ = 95.4 Hz); ^{31}P NMR (202 MHz, $CDCl_3$) δ 36.34.

GC t_R = 12.64 min; GCMS (EI, 70 eV) *m/z* = 268 (M) (1) 235 (M–SH) (14), 155 (12), 154 (100), 140 (22),139 (43), 125 (15), 92 (12), 91 (42), 77 (15), 47 (12). HRMS (ESI–TOF) m/z (the analysis has been performed for the mixture of epimers): [M+H] Calcd for $C_{14}H_{21}OPS$ 269.1116; Found 269.1123.

(((2-Mercapto)cycloheptyl)methyl)methylphenylphosphine oxide (41). This compound was prepared according to the general procedure from (((1-hydroxy)cycloheptyl)methyl)methylphenylphosphine sulfide **24** (0.032 g, 0.11 mmol) as a pale yellow oil; yield 0.028 g (88%); Isolated as a mixture of epimers; R_f 0.36 ($CHCl_3$); ^{1}H NMR (500 MHz, $CDCl_3$) (due to overlapping only selected peaks of epimers are described) δ 1.10–1.67 (m, 24H), 1.71 (d, $J_{P\text{–}H}$ = 12.6 Hz, 3H), 1.73 (d, $J_{P\text{–}H}$ = 12.6 Hz, 3H), 1.72 (d, $J_{P\text{–}H}$ = 12.6 Hz, 3H), 1.76–2.06 (m, 6H), 2.09–2.39 (m, 6H), 7.45–7.57 (m, 9H), 7.65–7.80 (m, 6H);

^{13}C NMR (126 MHz, $CDCl_3$) δ 20.46 and 20.53, 24.1 and 24.2, 25.5 and 25.6, 27.0 and 27.1 and 27.16 and 27.21, 31.97 and 32.02, 36.1 and 36.3, 37.0 and 37.1 and 37.3, 39.3 and 39.4, 45.6 (d, $J_{P\text{–}C}$ = 9.1 Hz) and 45.9 (d, $J_{P\text{–}C}$ = 9.1 Hz), 128.6 (d, $J_{P\text{–}C}$ = 10.9 Hz) and 128.7 (d, $J_{P\text{–}C}$ = 10.9 Hz), 129.9 (d, $J_{P\text{–}C}$ = 9.1 Hz) and 130.1 (d, $J_{P\text{–}C}$ = 9.1 Hz), 131.5 (d, $J_{P\text{–}C}$ = 2.7 Hz) and 131.6 (d, $J_{P\text{–}C}$ = 2.7 Hz); ^{31}P NMR (202 MHz, $CDCl_3$) δ 36.43 and 36.69 and 36.97 and 37.20; GC (compound 1) t_R = 12.74 min; GCMS (EI, 70 eV) *m/z* = 282 (1), 249 (M–SH) (17), 155 (14), 154 (100), 140 (21), 139 (37), 125 (13), 92 (12) 91 (41), 77 (14), 47 (11); (compound 2) t_R = 12.79 min; GCMS (EI, 70 eV) *m/z* = 282 (1), 250 (16), 249 (M–SH) (100), 155 (18), 154 (81), 141 (15), 140 (29), 139 (83), 125 (23), 109 (17), 92 (15) 91 (55), 77 (32), 67 (17), 47 (19); (compound 3) t_R = 12.88 min; GCMS (EI, 70 eV) *m/z* = 282 (1), 250 (17), 249 (M–SH) (100), 155 (14), 154 (45), 141 (14), 140 (24), 139 (75), 125 (19), 109 (16), 92 (10) 91 (42), 77 (29), 67 (16), 47 (17); HRMS (ESI–TOF) m/z (the analysis has been performed for the mixture of epimers): [M+H] Calcd for $C_{15}H_{23}OPS$ 283.1282; Found 283.1280.

(2-Butyl-3-mercaptohexyl)methylphenylphosphine oxide (42). This compound was prepared according to the general procedure from (2-butyl-2-hydroxyhexyl)methylphenylphosphine sulfide **25** (0.062 g, 0.19 mmol) as a pale yellow oil; yield 0.037 g (60%). Isolated as a mixture of epimers; R_f 0.68 ($CHCl_3$:MeOH, 50:1); ^{1}H NMR (500 MHz, $CDCl_3$) (due to overlapping only selected peaks of epimers are described): δ 0.73 (t, $J_{H\text{–}H}$ = 7.3 Hz, 3H) and 0.80 (t, $J_{H\text{–}H}$ = 7.3 Hz, 3H) and 0.86 (t, $J_{H\text{–}H}$ = 7.3 Hz, 3H) and 0.90 (t, $J_{H\text{–}H}$ = 7.3 Hz, 3H) and 0.95 (t, $J_{H\text{–}H}$ = 7.4 Hz, 3H), 1.00–1.67 (m, 40H), 1.73 (d, $J_{P\text{–}H}$ = 12.3 Hz, 12H), 1.82–2.21 (m, 4H), 2.22–2.43 (m, 4H), 2.52–2.68 (m, 4H), 2.83 (bs, 1H) and 3.09 (bs, 1H) and 3.27 (bs, 1H), 7.40–7.58 (m, 12H), 7.64–7.79 (m, 8H); ^{13}C NMR (126

MHz, $CDCl_3$) δ 11.2 and 11.3 and 11.4, 13.5 and 13.7 and 13.91 and 13.92 and 14.0, 17.3 (d, $J_{P–C}$ = 69.0 Hz), 20.8, 22.5, 22.6, 22.7, 22.8, 27.5, 28.0, 29.2, 29.4, 29.9, 30.1, 30.4, 30.7, 32.5, 32.7, 37.6, 37.8, 38.6, 38.9, 40.4, 40.5, 43.9, 44.0, 44.1, 44.2, 44.5, 128.6 (d, $J_{P–C}$ = 9.99 Hz), 130.1 (d, $J_{P–C}$ = 7.3 Hz), 131.5 (d, $J_{P–C}$ = 2.8 Hz); ^{31}P NMR (202 MHz, $CDCl_3$) δ 37.03 and 37.69 and 38.58; GC (compound 1) t_R = 12.10 min; GCMS (EI, 70 eV) *m/z* = 312 (1), 279 (M–SH) (14), 223 (29), 155 (12), 154 (100), 140 (17), 139 (50), 125 (12), 91 (31), 47 (11); (compound 2) t_R = 12.12 min; GCMS (EI, 70 eV) *m/z* = 312 (1), 279 (M–SH) (18), 223 (40), 155 (21), 154 (100), 140 (22), 139 (62), 125 (14), 92 (10), 91 (34), 77 (11), 55 (11), 47 (13); (compound 3) t_R = 12.14 min; GCMS (EI, 70 eV) *m/z* = 312 (1), 279 (M–SH) (38), 223 (56), 155 (13), 154 (100), 140 (35), 139 (93), 125 (23), 92 (12), 91 (41), 77 (18), 55 (17), 47 (20); (compound 4) t_R = 12.25 min; GCMS (EI, 70 eV) *m/z* = 312 (1), 279 (M–SH) (50), 223 (20), 155 (11), 154 (59), 141 (17), 140 (100), 139 (92), 125 (33), 92 (10), 91 (39), 77 (21), 55 (16), 47 (22); (compound 5) t_R = 12.30 min; GCMS (EI, 70 eV) *m/z* = 312 (1), 279 (M–SH) (46), 223 (11), 173 (18), 154 (29), 140 (100), 139 (75), 125 (29), 91 (27), 77 (15), 55 (12), 47 (16); (compound 6) t_R = 12.33 min; GCMS (EI, 70 eV) *m/z* = 312 (1), 279 (M–SH) (49), 223 (11), 173 (18), 154 (30), 140 (100), 139 (76), 125 (29), 91 (28), 77 (15), 55 (13), 47 (17); HRMS (ESI–TOF) m/z (the analysis has been performed for the mixture of epimers): [M+H] Calcd for $C_{17}H_{29}OPS$ 313.1742; Found 313.1749.

(3-Methyl-3-mercaptobutyl)methylphenylphosphine oxide (46). This compound was prepared according to the general procedure from (2-mesyloxy-3-methylbutyl)methylphenylphosphine sulfide **59** (0.087 g, 0.27 mmol) as a pale yellow oil; yield 0.054 g (82%); R_f 0.43 ($CHCl_3$); ^{1}H NMR (500 MHz, $CDCl_3$) δ 1.30 (s, 3H), 1.33 (s, 3H), 1.57–1.67 (m, 1H), 1.71 (d, $J_{P–H}$ = 12.6 Hz, 3H), 1.80–1.92 (m, 1H), 2.00–2.19 (m, 2H), 2.42 (bs, 1H), 7.44–7.55 (m, 3H), 7.63–7.75 (m, 2H); ^{13}C NMR (126 MHz, $CDCl_3$) δ 16.2 (d,

$J_{P–C}$ = 70.0 Hz), 27.7 (d, $J_{P–C}$ = 70.0 Hz), 32.0, 32.3, 37.8 (d, $J_{P–C}$ = 2.7 Hz), 44.5 (d, $J_{P–C}$ = 14.5 Hz), 128.6 (d, $J_{P–C}$ = 10.9 Hz), 129.9 (d, $J_{P–C}$ = 10.0 Hz), 131.6 (d, $J_{P–C}$ = 1.8 Hz), 133.1 (d, $J_{P–C}$ = 96.3 Hz); ^{31}P NMR (202 MHz, $CDCl_3$) δ 37.94; GC t_R = 11.02 min; GCMS (EI, 70 eV) *m/z* = 209 (M–SH) (42), 208 (12), 167 (87), 141 (28), 140 (100), 139 (86), 125 (80), 91 (23), 78 (10), 77 (40), 69 (29), 63 (12), 51 (18) 47 (42) HRMS (ESI–TOF) m/z: [M+H] Calcd for $C_{12}H_{19}OPS$ 243.0966; Found 243.0967.

The rearrangement of (S_P)–(2–mesyloxy–3–methylbutyl)methylphenylphosphine sulfide (S_P)–**61** (0.118 g, 0.37 mmol) afforded a product as a brown sticky solid; yield 0.057 g (64%); HPLC (Chiralcel OJ–H, Hexane/iPrOH = 98:2, 1.0 mL/min) t_{R1} = 19.24 min, t_{R2} = 22.74 min (67% ee).

General procedure for the reaction of β-hydroxyalkylphosphine sulfides with Brønsted acid

In a small tube (5 mL) equipped with magnetic stirrer and inert gas inlet was placed β-hydroxyphosphine sulfide (1.0 mmol) in CH_3COOH (2 mL). To mixture H_2SO_4 was added a few drops and stirred for 18 h at 55 °C. The reaction was quenched by addition of water (2 mL) and extracted with DCM (3 × 10 mL). The combined organic phases were dried over $MgSO_4$, filtered, and evaporated under reduced pressure. The residue was purified by flash column chromatography using $CHCl_3$.

(2-Acetoxypropyl)methylphenylphosphine sulfide (43). This compound was prepared according to the general procedure from (2-hydroxypropyl)methylphenylphosphine sulfide **6** (0.042 g, 0.20 mmol) as a pale yellow oil; yield 0.042 g (83%); Isolated as a mixture of diastereomers (dr = 67:33); R_f 0.39 ($CHCl_3$).

Major diastereomer: ^{1}H NMR (500 MHz, $CDCl_3$) δ 1.30 (dd, $J_{H–H}$ = 6.3 Hz, $J_{P–H}$ = 0.6 Hz, 3H), 1.97 (s, 3H), 2.01 (d, $J_{P–H}$ = 13.2 Hz, 3H), 2.12–2.25 (m, 1H), 2.55–2.64 (m, 1H), 5.30–5.41 (m, 1H), 7.47–7.56 (m, 3H), 7.84–7.93 (m, 2H); ^{13}C NMR (126 MHz, $CDCl_3$) δ 21.5 (d, $J_{P–C}$ = 77.2 Hz), 21.4, 21.6, 41.1 (d, $J_{P–C}$ = 53.6 Hz), 67.1, 128.6 (d, $J_{P–C}$ = 11.8 Hz), 130.2 (d, $J_{P–C}$ = 10.9 Hz), 131.6 (d, $J_{P–C}$ = 1.8 Hz), 133.0 (d, $J_{P–C}$ = 79.0 Hz), 169.8; ^{31}P NMR (202 MHz, $CDCl_3$) δ 35.19; GC t_R = 10.67 min; GCMS (EI, 70 eV) *m/z* = 256 (M) (10), 157 (11), 156 (100),155 (30), 141 (25), 140 (16), 123 (16), 109 (10), 91 (13), 78 (31), 77(16), 63 (17).

Minor diastereomer: ^{1}H NMR (500 MHz, $CDCl_3$) δ 1.32 (dd, $J_{H–H}$ = 6.3 Hz, $J_{H–H}$ = 1.3 Hz, 3H), 1.55 (s, 3H), 1.97 (d, $J_{P–H}$ = 12.9 Hz, 3H), 2.12–2.26 (m, 1H), 2.64–2.73 (m, 1H), 5.18–5.29 (m, 1H), 7.46–7.56 (m, 3H), 7.83–7.95 (m, 2H); ^{13}C NMR (126 MHz, $CDCl_3$) δ 20.6, 21.5, 22.0 (d, $J_{P–C}$ = 76.3 Hz), 41.1 (d, $J_{P–C}$ = 53.6 Hz), 66.4, 128.4 (d, $J_{P–C}$ = 11.8 Hz), 130.6 (d, $J_{P–C}$ = 10.9 Hz), 131.4 (d, $J_{P–C}$ = 1.8 Hz), 132.0 (d, $J_{P–C}$ = 78.1 Hz), 169.6; ^{31}P NMR (202 MHz, $CDCl_3$) δ 35.11; GC t_R = 10.57 min; GCMS (EI, 70 eV) *m/z* = 256 (M) (10), 157 (12), 156 (100), 155 (31), 141 (26), 140 (29), 123 (16), 121(11), 109 (14), 91 (16), 78 (32), 77(18), 63 (17).

HRMS (ESI–TOF) m/z (the analysis has been performed for diastereomeric mixture): [M+H] Calcd for $C_{12}H_{17}O_2PS$ 257.0753; Found 257.0760.

(2-Acetoxybutyl)methylphenylphosphine sulfide (44). This compound was prepared according to the general procedure from (2-hydroxybutyl)methylphenylphosphine sulfide **7** (0.046 g, 0.20 mmol) as a pale yellow oil; yield 0.051 g (95%); Isolated as a mixture of diastereomers (dr = 51:49); R_f 0.37 ($CHCl_3$).

Major diastereomer: ^{1}H NMR (500 MHz, $CDCl_3$) δ 0.86 (t, $J_{H–H}$ = 7.4 Hz, 3H), 1.56 (s, 3H), 1.60–1.70 (m, 2H), 1.98 (d, $J_{P–H}$ = 12.9 Hz, 3H), 2.16–2.29 (m, 2H), 5.13–5.21 (m, 1 H),

7.46–7.57 (m, 3H), 7.82–7.95 (m, 2H); ^{13}C NMR (126 MHz, $CDCl_3$) δ 9.1, 21.1, 22.1 (d, J_{P-C} = 58.1 Hz), 28.5 (d, J_{P-C} = 11.8 Hz), 38.9 (d, J_{P-C} = 54.5 Hz), 70.3 (d, J_{P-C} = 2.7 Hz), 128.5 (d, J_{P-C} = 11.8 Hz), 130.7 (d, J_{P-C} = 10.9 Hz), 131.5 (d, J_{P-C} = 1.8 Hz), 132.1 (d, J_{P-C} = 78.1 Hz), 169.8; ^{31}P NMR (202 MHz, $CDCl_3$) δ 35.52; GC t_R = 10.81 min; GCMS (EI, 70 eV) *m/z* = 270 (M) (4), 210 (18), 157 (12), 156 (100), 155 (54), 140 (20), 123 (10), 91 (12), 78 (24), 77(13), 63 (15).

Minor diastereomer: ^{1}H NMR (500 MHz, $CDCl_3$) δ 0.86 (t, J_{H-H} = 7.4 Hz, 3H), 1.61–1.70 (m, 1H), 1.70–1.81 (m, 1H), 1.95 (s, 3H), 2.02 (d, J_{P-H} = 13.2 Hz, 3H), 2.50–2.67 (m, 2H), 5.21–5.28 (m, 1H), 7.44–7.57 (m, 3H), 7.83–7.96 (m, 2H); ^{13}C NMR (126 MHz, $CDCl_3$) δ 9.2, 20.5, 21.4 (d, J_{P-C} = 57.2 Hz), 28.4 (d, J_{P-C} = 9.1 Hz), 39.1 (d, J_{P-C} = 53.6 Hz), 71.1 (d, J_{P-C} = 1.8 Hz), 128.7 (d, J_{P-C} = 11.8 Hz), 130.3 (d, J_{P-C} = 10.0 Hz), 131.6 (d, J_{P-C} = 2.7 Hz), 133.2 (d, J_{P-C} = 79.0 Hz), 170.1; ^{31}P NMR (202 MHz, $CDCl_3$) δ 35.51; GC t_R = 10.89 min; GCMS (EI, 70 eV) *m/z* = 270 (M) (4), 210 (17), 157 (12), 156 (100), 155 (50), 140 (21), 123 (10), 91 (12), 78 (24), 77(13), 63 (15).

HRMS (ESI–TOF) m/z (the analysis has been performed for diastereomeric mixture): [M+H] Calcd for $C_{13}H_{19}O_2PS$ 271.0905; Found 271.0916.

(2-Acetoxy-3-methylbutyl)methylphenylphosphine sulfide (45). This compound was prepared according to the general procedure from (2-hydroxy-3-methylbutyl)methylphenylphosphine sulfide **8** (0.069 g, 0.29 mmol) running the reaction for 48 h. The product was obtained as a pale yellow oil; yield 0.036 g (43%); Isolated as a mixture of diastereomers (dr = 51:49); R_f 0.76 (Hex:AcOEt, 2:1).

Major diastereomer: ^{1}H NMR (500 MHz, $CDCl_3$) δ 0.88 (t, J_{H-H} = 6.9 Hz, 6H), 1.53 (s, 3H), 1.93–1.96 (m, 1H), 1.98 (d, J_{P-H} = 12.9 Hz, 3H), 2.14–2.25 (m, 1H), 2.51–2.60 (m, 1H), 5.14–

5.24 (m, 1H), 7.45–7.59 (m, 3H), 7.80–7.95 (m, 2H); ^{13}C NMR (126 MHz, $CDCl_3$) δ 17.2, 17.4, 20.4, 21.7 (d, $J_{P\text{–}C}$ = 58.1 Hz), 32.3 (d, $J_{P\text{–}C}$ = 10.9 Hz), 36.2 (d, $J_{P\text{–}C}$ = 54.5 Hz), 72.7 (d, $J_{P\text{–}C}$ = 4.5 Hz), 128.5 (d, $J_{P\text{–}C}$ = 11.8 Hz), 130.7 (d, $J_{P\text{–}C}$ = 10.0 Hz), 131.4 (d, $J_{P\text{–}C}$ = 2.7 Hz), 132.1 (d, $J_{P\text{–}C}$ = 77.2 Hz), 169.7; ^{31}P NMR (202 MHz, $CDCl_3$) δ 36.42; GC t_R = 10.97 min; GCMS (EI, 70 eV) *m/z* = 284 (M) (1), 225 (18), 224 (35), 193 (17), 157 (19), 156 (100), 155 (74), 141 (21), 140 (34), 125 (21), 124 (15), 123 (18), 121 (18), 109 (21), 91 (26), 78 (35), 77(26), 69 (32), 51 (13), 45 (11).

Minor diastereomer: ^{1}H NMR (500 MHz, $CDCl_3$) δ 0.85 (t, $J_{H\text{–}H}$ = 6.8 Hz, 6H), 1.84–1.90 (m, 1H), 1.91 (s, 3H), 2.01 (d, $J_{P\text{–}H}$ = 12.9 Hz, 3H), 2.13–2.26 (m, 1H), 2.44–2.51 (m, 1H), 5.14–5.24 (m, 1H), 7.47–7.56 (m, 3H), 7.80–7.95 (m, 2H); ^{13}C NMR (126 MHz, $CDCl_3$) δ 17.5, 17.5, 20.9 (d, $J_{P\text{–}C}$ = 56.3 Hz), 21.0, 32.1 (d, $J_{P\text{–}C}$ = 10.0 Hz), 36.3 (d, $J_{P\text{–}C}$ = 53.6 Hz), 73.4 (d, $J_{P\text{–}C}$ = 3.6 Hz), 128.7 (d, $J_{P\text{–}C}$ = 11.8 Hz), 130.3 (d, $J_{P\text{–}C}$ = 10.0 Hz), 131.5 (d, $J_{P\text{–}C}$ = 2.7 Hz), 133.1 (d, $J_{P\text{–}C}$ = 79.0 Hz), 170.0; ^{31}P NMR (202 MHz, $CDCl_3$) δ 36.38; GC t_R = 11.05 min; GCMS (EI, 70 eV) *m/z* = 284 (M) (2), 225 (12), 224 (33), 193 (13), 157 (18), 156 (100), 155 (69), 141 (23), 140 (38), 125 (16), 124 (13), 123 (17), 121 (16), 109 (19), 91 (26), 78 (34), 77(28), 69 (26), 51 (14).

HRMS (ESI–TOF) m/z (the analysis has been performed for diastereomeric mixture): [M+H] Calcd for $C_{14}H_{21}O_2PS$ 285.1085; Found 285.1073.

(2-Acetoxy-3,3-dimethylbutyl)methylphenylphosphine sulfide (47). This compound was prepared according to the general procedure from (2-hydroxy-3,3-dimethylbutyl)methylphenylphosphine sulfide **9** (0.067 g, 0.26 mmol) as a pale yellow sticky solid; yield 34% as a mixture with SM **8,** (0,019 g, 25% isolated as a pure compound); Isolated as a mixture of diastereomers (dr = 63:37); R_f 0.57 ($CHCl_3$).

Major diastereomer: ^{1}H NMR (500 MHz, $CDCl_3$) δ 0.86 (s, 9H), 1.84 (s, 3H), 2.01 (d, J_{P-H} = 12.9 Hz, 3H), 2.21–2.33 (m, 1H), 2.41–2.54 (m, 1H), 5.03–5.13 (m, 1H), 7.44–7.61 (m, 3H), 7.73–7.98 (m, 2H); ^{13}C NMR (126 MHz, $CDCl_3$) δ 20.1 (d, J_{P-C} = 56.3 Hz), 21.0, 25.5, 35.6 (d, J_{P-C} = 10.9 Hz), 36.7 (d, J_{P-C} = 54.5 Hz), 74.7 (d, J_{P-C} = 5.5 Hz), 128.6 (d, J_{P-C} = 11.8 Hz), 130.3 (d, J_{P-C} = 10.0 Hz), 131.5 (d, J_{P-C} = 2.7 Hz), 132.8 (d, J_{P-C} = 79.0 Hz), 170.1; ^{31}P NMR (202 MHz, $CDCl_3$) δ 37.02; GC t_R = 11.24 min; GCMS (EI, 70 eV) *m/z* = 298 (M) (7), 157 (11), 156 (100), 155 (29), 141 (15), 140 (11), 78 (19).

Minor diastereomer: ^{1}H NMR (500 MHz, $CDCl_3$) δ 0.86 (s, 9H), 1.50 (s, 3H), 1.97 (d, J_{P-H} = 12.9 Hz, 3H), 2.20–2.34 (m, 1H), 2.40–2.54 (m, 1H), 5.18–5.33 (m, 1H), 7.44–7.60 (m, 3H), 7.78–7.97 (m, 2H); ^{13}C NMR (126 MHz, $CDCl_3$) δ 20.4, 21.3 (d, J_{P-C} = 57.2 Hz), 25.5, 35.5 (d, J_{P-C} = 10.9 Hz), 36.1 (d, J_{P-C} = 54.5 Hz), 74.2 (d, J_{P-C} = 4.5 Hz), 128.5 (d, J_{P-C} = 11.8 Hz), 130.9 (d, J_{P-C} = 10.0 Hz), 131.4 (d, J_{P-C} = 2.9 Hz), 132.0 (d, J_{P-C} = 77.2 Hz), 169.8; ^{31}P NMR (202 MHz, $CDCl_3$) δ 36.93; GC t_R = 11.18 min; GCMS (EI, 70 eV) *m/z* = 298 (M) (7), 157 (11), 156 (100), 155 (29), 141 (16), 140 (12), 78 (21), 77 (11), 63 (10).

HRMS (ESI–TOF) m/z (the analysis has been performed for diastereomeric mixture): [M+H] Calcd for $C_{15}H_{23}O_2PS$299.1229; Found 299.1229.

(2-Acetoxy-2-cyclohexylethyl)methylphenylphosphine sulfide (48). This compound was prepared according to the general procedure from (2-cyclohexyl-2-hydroxyethyl)methylphenylphosphine sulfide **11** (0.037 g, 0.13 mmol) as a pale yellow sticky solid; yield 0.012 g (29%); Isolated as a mixture of diastereomers (dr = 51:49); Rf 0.54 ($CHCl_3$).

Major diastereomer: ^{1}H NMR (500 MHz, $CDCl_3$) δ 0.90–1.02 (m, 2H), 1.02–1.23 (m, 3H), 1.53–1.76 (m, 6H), 1.90 (s, 3H), 1.97 (d, J_{P-H} = 12.9 Hz, 3H), 2.18–2.31 (m, 2H), 5.10–5.25

(m, 1H), 7.45–7.58 (m, 3H), 7.80–7.94 (m, 2H); ^{13}C NMR (126 MHz, $CDCl_3$) δ 20.4, 21.0 (d, J_{P-C} = 57.2 Hz), 25.9, 27.7, 28.3, 36.7 (d, J_{P-C} = 54.5 Hz), 42.4 (d, J_{P-C} = 10.0 Hz), 72.3 (d, J_{P-C} = 3.6 Hz), 128.5 (d, J_{P-C} = 11.8 Hz), 130.3 (d, J_{P-C} = 10.0 Hz), 131.4 (d, J_{P-C} = 2.7 Hz), 133.1 (d, J_{P-C} = 79.0 Hz), 169.8; ^{31}P NMR (202 MHz, $CDCl_3$) δ 36.42; GC t_R = 13.25 min; GCMS (EI, 70 eV) *m/z* = 324 (M) (0.1), 265(13), 264 (20), 157 (36), 156 (100), 155 (50), 141 (10), 110 (11), 109 (27), 91 (12), 78 (15), 77(12), 67 (23), 55 (11).

Minor diastereomer: ^{1}H NMR (500 MHz, $CDCl_3$) δ 0.88–1.02 (m, 2H), 1.03–1.23 (m, 3H), 1.51 (s, 3H), 1.54–1.78 (m, 6H), 2.00 (d, J_{P-H} = 13.2 Hz, 3H), 2.44–2.63 (m, 2H), 5.12–5.25 (m, 1H), 7.45–7.58 (m, 3H), 7.82–7.92 (m, 2H); ^{13}C NMR (126 MHz, $CDCl_3$) δ 21.1, 21.7 (d, J_{P-C} = 58.1 Hz), 26.2, 27.7, 28.2, 36.6 (d, J_{P-C} = 54.5 Hz), 42.1 (d, J_{P-C} = 9.1 Hz), 73.1 (d, J_{P-C} = 2.7 Hz), 128.6 (d, J_{P-C} = 11.8 Hz), 130.7 (d, J_{P-C} = 10.9 Hz), 131.5 (d, J_{P-C} = 2.7 Hz), 132.2 (d, J_{P-C} = 78.1 Hz), 170.0; ^{31}P NMR (202 MHz, $CDCl_3$) δ 36.38; GC t_R = 13.34 min; GCMS (EI, 70 eV) *m/z* = 265 (10), 264 (20), 157 (34), 156 (100), 155 (44), 141 (12), 109 (24), 91 (13), 78 (16), 77(11), 67 (20).

HRMS (ESI–TOF) m/z (the analysis has been performed for diastereomeric mixture): [M+H] Calcd for $C_{17}H_{25}O_2PS$ 325.1395; Found 325.1386.

(((2-Mercapto)cyclo)hexylethyl)methylphenylphosphine oxide (49). This compound was prepared according to the general procedure from (2-cyclohexyl-2-hydroxyethyl)methylphenylphosphine sulfide **11** (0.037 g, 0.13 mmol) as a pale yellow oil; yield 0.021 g (58%); R_f 0.26 ($CHCl_3$); ^{1}H NMR (500 MHz, $CDCl_3$) δ 1.14–1.25 (m, 1H), 1.26 (s, 1H), 1.33–1.64 (m, 9H), 1.65–1.71 (m, 1H), 1.75 (d, J_{P-H} = 12.6 Hz, 3H), 1.86–1.98 (m, 1H), 2.07–2.26 (m, 2H), 7.45–7.60 (m, 3H), 7.69–7.82 (m, 2H); ^{13}C NMR (126 MHz, $CDCl_3$) δ 16.3 (d, J_{P-C} = 69.9 Hz), 22.3, 25.7, 26.2 (d, J_{P-C} = 69.9 Hz), 36.9, 39.5, 39.8, 49.5 (d, J_{P-C} =

14.5 Hz), 128.7 (d, $J_{P\text{–}C}$ = 11.8 Hz), 130.0 (d, $J_{P\text{–}C}$ = 9.1 Hz), 131.7 (d, $J_{P\text{–}C}$ = 1.8 Hz), 132.9 (d, $J_{P\text{–}C}$ = 95.4 Hz); ^{31}P NMR (202 MHz, $CDCl_3$) δ 39.22; GC t_R = 13.48 min; GCMS (EI, 70 eV) *m/z* = 282 (1), 250 (13), 249 (M–SH) (79), 168 (10), 167 (100), 154 (22), 141(27),140 (40), 139 (52), 125 (28), 109 (13), 91 (30), 81 (11), 79 (17), 77 (22), 67 (32), 55 (11), 47 (20); HRMS (ESI–TOF) m/z: [M+H] Calcd for $C_{15}H_{23}OPS$ 283.1273; Found 283.1280.

(2-Methyl-2-mercaptobutyl)methylphenylphosphine oxide (50). This compound was prepared according to the general procedure from (2-hydroxy-2-methylbutyl)methylphenylphosphine sulfide **12** (0.046 g, 0.19 mmol) as a pale yellow oil; yield 0.040 g (87%); Isolated as a mixture of diastereomers (dr = 52:48); R_f 0.46 (Hexane/AcOEt/iPrOH 10:1:1).

Major diastereomer: ^{1}H NMR (500 MHz, $CDCl_3$) δ 0.97 (t, $J_{H\text{–}H}$ = 7.3 Hz, 3H), 1.41 (s, 3H), 1.67–1.74 (m, 2H), 1.76 (d, $J_{P\text{–}H}$ = 12.6 Hz, 3H), 2.31–2.39 (m, 1H), 2.40–2.47 (m, 1H), 2.58 (bs, 1H), 7.46–7.55 (m, 3H), 7.69–7.76 (m, 2H); ^{13}C NMR (126 MHz, $CDCl_3$) δ 9.4, 19.2 (d, $J_{P\text{–}C}$ = 70.8 Hz), 30.9 (d, $J_{P\text{–}C}$ = 4.5 Hz), 38.0 (d, $J_{P\text{–}C}$ = 5.5 Hz), 46.1 (d, $J_{P\text{–}C}$ = 67.2 Hz), 47.0 (d, $J_{P\text{–}C}$ = 4.5 Hz), 128.6 (d, $J_{P\text{–}C}$ = 11.8 Hz), 129.8 (d, $J_{P\text{–}C}$ = 9.1 Hz), 131.5 (d, $J_{P\text{–}C}$ = 1.8 Hz), 135.2 (d, $J_{P\text{–}C}$ = 96.3 Hz); ^{31}P NMR (202 MHz, $CDCl_3$) δ 33.87; GC t_R = 10.60 min; GCMS (EI, 70 eV) *m/z* = 209 (M–SH) (40), 140 (26), 139 (100), 125 (14), 91 (19), 77 (19), 55 (11), 47 (16).

Minor diastereomer: ^{1}H NMR (500 MHz, $CDCl_3$) δ 0.92 (t, $J_{H\text{–}H}$ = 7.3 Hz, 3H), 1.53 (s, 3H), 1.80 (d, $J_{P\text{–}H}$ = 12.6 Hz, 3H), 1.82–1.92 (m, 2H), 2.40–2.46 (m, 2H), 2.58 (bs, 1H), 7.46–7.55 (m, 3H), 7.69–7.76 (m, 2H); ^{13}C NMR (126 MHz, $CDCl_3$) δ 9.2, 19.2 (d, $J_{P\text{–}C}$ = 70.8 Hz), 30.9 (d, $J_{P\text{–}C}$ = 4.5 Hz), 38.7 (d, $J_{P\text{–}C}$ = 8.2 Hz), 46.0 (d, $J_{P\text{–}C}$ = 68.1 Hz), 46.9 (d, $J_{P\text{–}C}$ = 4.5 Hz), 128.6 (d, $J_{P\text{–}C}$ = 11.8 Hz), 129.8 (d, $J_{P\text{–}C}$ = 9.1 Hz), 131.5 (d, $J_{P\text{–}C}$ = 1.8 Hz), 135.3 (d,

J_{P-C} = 96.3 Hz); ^{31}P NMR (202 MHz, $CDCl_3$) δ 34.20; GC t_R = 10.58 min; GCMS (EI, 70 eV) *m/z* = 209 (M–SH) (41), 140 (28), 139 (100), 125 (15), 91 (18), 77 (18), 47 (16).

HRMS (ESI–TOF) m/z (the analysis has been performed for diastereomeric mixture): [M+H] Calcd for $C_{12}H_{19}OPS$ 243.0961; Found 243.0967.

(2-Methyl-2-mercaptohexyl)methylphenylphosphine oxide (51). This compound was prepared according to the general procedure from (2-hydroxy-2-methylhexyl)methylphenylphosphine sulfide **14** (0.051 g, 0.19 mmol) as a pale yellow oil; yield 0.045 g (88%); Isolated as a mixture of diastereomers (dr = 54:46); R_f 0.32 ($CHCl_3$).

Major diastereomer: ^{1}H NMR (500 MHz, $CDCl_3$) δ 0.84 (t, J_{H-H} = 6.9 Hz, 3H), 1.08–1.34 (m, 5H), 1.42 (s, 3H),1.56–1.62 (m, 1H), 1.74 (d, J_{P-H} = 13.6 Hz, 3H), 2.31–2.40 (m, 2H), 2.60 (bs, 1H), 7.44–7.54 (m, 3H), 7.65–7.78 (m, 2H); ^{13}C NMR (126 MHz, $CDCl_3$) δ 13.9, 19.2 (d, J_{P-C} = 69.9 Hz), 22.7, 27.1, 31.6 (d, J_{P-C} = 6.4 Hz), 45.2 (d, J_{P-C} = 5.5 Hz), 46.3 (d, J_{P-C} = 62.7 Hz), 46.5 (d, J_{P-C} = 4.5 Hz), 128.6 (d, J_{P-C} = 11.8 Hz), 129.9 (d, J_{P-C} = 10.9 Hz), 131.5 (d, J_{P-C} = 1.8 Hz), 135.2 (d, J_{P-C} = 96.3 Hz; ^{31}P NMR (202 MHz, $CDCl_3$) δ 33.91; GC t_R = 11.26 min; GCMS (EI, 70 eV) *m/z* = 237 (M–SH) (40), 141 (11), 140 (84), 139 (100), 125 (27), 91 (19), 77 (22), 56 (17), 55 (16), 51 (11), 47 (17).

Minor diastereomer: ^{1}H NMR (500 MHz, $CDCl_3$) δ 0.80 (t, J_{H-H} = 7.3 Hz, 3H), 1.06–1.33 (m, 5H), 1.54 (s, 3H), 1.56–1.63 (m, 1H), 1.78 (d, J_{P-H} = 12.9 Hz, 3H), 2.40–2.46 (m, 2H), 2.60 (bs, 1H), 7.44–7.54 (m, 3H), 7.67–7.76 (m, 2H); ^{13}C NMR (126 MHz, $CDCl_3$) δ 13.9, 19.2 (d, J_{P-C} = 69.9 Hz), 22.7, 27.0, 31.7 (d, J_{P-C} = 3.6 Hz), 45.7 (d, J_{P-C} = 7.3 Hz), 46.3 (d, J_{P-C} = 62.7 Hz),46.4 (d, J_{P-C} = 4.5 Hz), 128.6 (d, J_{P-C} = 11.8 Hz), 129.8 (d, J_{P-C} = 9.1 Hz), 131.5 (d, J_{P-C} = 1.8 Hz), 135.2 (d, J_{P-C} = 98.1 Hz); ^{31}P NMR (202 MHz, $CDCl_3$) δ 34.14; GC

t_R = 11.29 min; GCMS (EI, 70 eV) *m/z* = 237 (M–SH) (46), 140 (73), 139 (100), 125 (23), 91 (17), 77 (20), 56 (10), 55 (14), 47 (16).

HRMS (ESI–TOF) m/z (the analysis has been performed for diastereomeric mixture): [M+H] Calcd for $C_{14}H_{23}OPS$ 271.1270; Found 271.1280.

(2-Methyl-2-mercaptopentyl)methylphenylphosphine oxide (52). This compound was prepared according to the general procedure from (2-hydroxy-2-methylpentyl)methylphenylphosphine sulfide **15** (0.053 g, 0.21 mmol) as a pale yellow oil; yield 0.050 g (95%); Isolated as a mixture of diastereomers (dr = 51:49); R_f 0.39 ($CHCl_3$).

Major diastereomer: ^{1}H NMR (500 MHz, $CDCl_3$) δ 0.85 (t, $J_{H–H}$ = 7.4 Hz, 3H), 1.25–1.40 (m, 1H), 1.42 (s, 3H), 1.43–1.51 (m, 1H), 1.55–1.63 (m, 2H), 1.75 (d, $J_{P–H}$ = 12.6 Hz, 3H), 2.31–2.40 (m, 1H), 2.41–2.50 (m, 1H), 2.54 (bs, 1H), 7.43–7.55 (m, 3H), 7.68–7.76 (m, 2H); ^{13}C NMR (126 MHz, $CDCl_3$) δ 14.1, 18.3, 19.2 (d, $J_{P–C}$ = 69.9 Hz), 31.7, 46.3 (d, $J_{P–C}$ = 66.3 Hz), 46.5 (d, $J_{P–C}$ = 5.5 Hz), 47.6 (d, $J_{P–C}$ = 5.5 Hz), 128.6 (d, $J_{P–C}$ = 11.8 Hz), 129.9 (d, $J_{P–C}$ = 11.8 Hz), 131.5 (d, $J_{P–C}$ = 1.8 Hz), 135.1 (d, $J_{P–C}$ = 95.4 Hz); ^{31}P NMR (202 MHz, $CDCl_3$) δ 34.41; GC t_R = 10.88 min; GCMS (EI, 70 eV) *m/z* = 223 (M–SH) (38), 140 (75), 139 (100), 125 (26), 91 (19), 77 (21), 55 (11), 47 (18).

Minor diastereomer: ^{1}H NMR (500 MHz, $CDCl_3$) δ 0.78 (t, $J_{H–H}$ = 7.3 Hz, 3H), 1.25–1.41 (m, 3H), 1.54 (s, 3H), 1.68–1.73 (m, 1H), 1.78 (d, $J_{P–H}$ = 12.6 Hz, 3H), 2.40–2.49 (m, 2H), 2.60 (bs, 1H), 7.46–7.54 (m, 3H), 7.67–7.75 (m, 2H); ^{13}C NMR (126 MHz, $CDCl_3$) δ 14.0, 18.1, 19.2 (d, $J_{P–C}$ = 69.9 Hz), 31.6, 46.3 (d, $J_{P–C}$ = 67.2 Hz), 46.5 (d, $J_{P–C}$ = 5.5 Hz), 48.1 (d, $J_{P–C}$ = 7.3 Hz), 128.6 (d, $J_{P–C}$ = 11.8 Hz), 129.8 (d, $J_{P–C}$ = 11.8 Hz), 131.5 (d, $J_{P–C}$ = 1.8 Hz), 135.1 (d, $J_{P–C}$ = 96.3 Hz); ^{31}P NMR (202 MHz, $CDCl_3$) δ 34.14; GC t_R = 10.86 min; GCMS

(EI, 70 eV) *m/z* = 223 (M–SH) (36), 140 (79), 139 (100), 125 (27), 91 (20), 77 (22), 56 (12), 47 (18).

HRMS (ESI–TOF) m/z (the analysis has been performed for diastereomeric mixture): [M+H] Calcd for $C_{13}H_{21}OPS$ 257.1117; Found 257.1123.

(2-Methyl-2-mercaptopropyl)methylphenylphosphine oxide (53). This compound was prepared according to the general procedure from (2-hydroxy-2-methylpropyl)methylphenylphosphine oxide **19** (0.046 g, 0.20 mmol) as a pale yellow oil; yield 0.032 g (69%); R_f 0.43 ($CHCl_3$); ^{1}H NMR (500 MHz, $CDCl_3$) δ 1.45 (s, 3H), 1.58 (s, 3H), 1.75 (d, J_{P-H} = 12.6 Hz, 3H), 2.36–2.52 (m, 2H), 2.73 (s, 1H), 7.41–7.54 (m, 3H), 7.62–7.80 (m, 2H); ^{13}C NMR (126 MHz, $CDCl_3$) δ 19.1 (d, J_{P-C} = 72.7 Hz), 34.0 (d, J_{P-C} = 5.5 Hz), 34.5 (d, J_{P-C} = 7.3 Hz), 42.7 (d, J_{P-C} = 4.5 Hz), 47.9 (d, J_{P-C} = 67.2 Hz), 128.6 (d, J_{P-C} = 11.8 Hz), 129.9 (d, J_{P-C} = 11.8 Hz), 131.5 (d, J_{P-C} = 2.7 Hz), 135.0 (d, J_{P-C} = 97.2 Hz); ^{31}P NMR (202 MHz, $CDCl_3$) δ 33.65; GC t_R = 9.94 min; GCMS (EI, 70 eV) *m/z* = 228 (2), 195 (M–SH) (33), 154 (14), 140 (10), 139 (100), 91 (23), 77 (20), 47 (15); Anal. Calcd. for $C_{11}H_{17}OPS$: C 57.87%, H 7.51%. Found: C 57.98%, H 7.82%.

The rearrangement of (S_P)-(2-hydroxy-2-methylpropyl)methylphenylphosphine oxide (S_P)-**19** (0.146 g, 0.60 mmol) afforded a product as a pale yellow oil; yield 0.130 g (89%); HPLC (Chiralcel OD–H, Hexane/iPrOH = 95:5, 0.5 mL/min) t_{R1} = 29.99 min, t_{R2} = 33.17 min (67% ee).

(2-Ethyl-2-mercaptobutyl)methylphenylphosphine oxide (54). This compound was prepared according to the general procedure from (2-hydroxy-2-ethylbutyl)methylphenylphosphine sulfide **20** (0.068 g, 0.26 mmol) as a pale yellow oil; yield 0.061 g (89%); R_f 0.68 ($CHCl_3$/MeOH 15:1); ^{1}H NMR (500 MHz, $CDCl_3$) δ 0.86 (t, J_{H-H} =

7.3 Hz, 3H), 0.89 (t, $J_{H–H}$ = 7.3 Hz, 3H), 1.68–1.78 (m, 3H), 1.79 (d, $J_{P–H}$ = 12.9 Hz, 3H), 1.84–1.94 (m, 1H), 2.30–2.44 (m, 2H), 3.05 (bs, 1H), 7.45–7.55 (m, 3H), 7.69–7.75 (m, 2H); ^{13}C NMR (126 MHz, $CDCl_3$) δ 8.7, 8.9, 19.3 (d, $J_{P–C}$ = 70.8 Hz), 34.5 (d, $J_{P–C}$ = 4.5 Hz), 34.7 (d, $J_{P–C}$ = 7.3 Hz), 43.2 (d, $J_{P–C}$ = 68.1 Hz), 50.9 (d, $J_{P–C}$ = 4.5 Hz), 128.6 (d, $J_{P–C}$ = 11.8 Hz), 129.8 (d, $J_{P–C}$ = 9.1 Hz), 131.5 (d, $J_{P–C}$ = 2.7 Hz), 135.4 (d, $J_{P–C}$ = 96.3 Hz); ^{31}P NMR (202 MHz, $CDCl_3$) δ 34.72; GC t_R = 11.04 min; GCMS (EI, 70 eV) *m/z* = 223 (M–SH) (21), 222 (12), 141 (11), 140 (100), 139 (89), 125 (50), 91 (27), 77 (29), 55 (17), 51 (11), 47 (27); HRMS (ESI–TOF) m/z: [M+H] Calcd for $C_{13}H_{21}OPS$ 257.1112; Found 257.1123.

The rearrangement of (S_P)-(2-hydroxy-2-ethylbutyl)methylphenylphosphine sulfide (S_P)-**20** (0.087 g, 0.34 mmol) afforded a product as a pale yellow oil; yield 0.062 g (71%); HPLC (Chiralcel OD–H, Hexane/iPrOH = 95:5, 0.5 mL/min) t_{R1} = 24.38 min, t_{R2} = 26.74 min (80% ee).

(((1-Mercapto)cyclopentyl)methyl)methylphenylphosphine oxide (55). This compound was prepared according to the general procedure from [(1-hydroxy)cyclopentylmethyl)]methylphenylphosphine sulfide **22** (0.044 g, 0.17 mmol) as a pale yellow oil; yield 0.012 g (26%); R_f 0.41 ($CHCl_3$); ^{1}H NMR (500 MHz, $CDCl_3$) δ 1.63–1.71 (m, 2H), 1.74–1.81 (m, 2H), 1.84 (d, $J_{P–H}$ = 12.9 Hz, 3H), 1.87–1.97 (m, 4H), 2.46–2.54 (m, 1H), 2.58–2.63 (m, 1H), 2.64 (bs, 1H), 7.47–7.57 (m, 3 H), 7.71–7.78 (m, 2H); ^{13}C NMR (126 MHz, $CDCl_3$) δ 19.1 (d, $J_{P–C}$ = 70.8 Hz), 23.0, 23.2, 43.4 (d, $J_{P–C}$ = 6.4 Hz), 44.3 (d, $J_{P–C}$ = 7.3 Hz), 46.4 (d, $J_{P–C}$ = 69.0 Hz), 52.0 (d, $J_{P–C}$ = 5.5 Hz), 128.7 (d, $J_{P–C}$ = 11.8 Hz), 130.0 (d, $J_{P–C}$ = 9.1 Hz), 131.6 (d, $J_{P–C}$ = 2.7 Hz), 135.1 (d, $J_{P–C}$ = 95.4 Hz); ^{31}P NMR (202 MHz, $CDCl_3$) δ 35.27; GC t_R = 11.77 min; GCMS (EI, 70 eV) *m/z* = 221 (M–SH) (54), 220 (18), 219 (18), 154 (11), 141 (13), 140 (62), 139 (100), 125 (45), 115 (11), 91 (26), 79 (22), 77

(41), 67 (12), 51 (15), 47 (37); HRMS (ESI–TOF) m/z: [M+H] Calcd for $C_{13}H_{19}OPS$ 255.0955; Found 255.0967.

(((1-Mercapto)cyclohexyl)methyl)methylphenylphosphine oxide (56). This compound was prepared according to the general procedure from (((1–hydroxy)cyclohexyl)methyl)methylphenylphosphine sulfide **23** (0.044 g, 0.17 mmol) as a pale yellow oil; yield 0.044 g (96%); R_f 0.38 ($CHCl_3$); ^{1}H NMR (500 MHz, $CDCl_3$) δ 1.12–1.24 (m, 1H), 1.36–1.45 (m, 1H), 1.45–1.52 (m, 1H), 1.52–1.60 (m, 1H), 1.61–1.77 (m, 5H), 1.81 (d, $J_{P\text{–}H}$ = 12.9 Hz, 3H), 1.84–1.91 (m, 1H), 2.45 (d, $J_{P\text{–}H}$ = 10.4 Hz, 2H), 2.76 (bs, 1H), 7.44–7.57 (m, 3H), 7.68–7.79 (m, 2H); ^{13}C NMR (126 MHz, $CDCl_3$) δ 19.4 (d, $J_{P\text{–}C}$ = 70.8 Hz), 22.2, 25.3, 40.9 (d, $J_{P\text{–}C}$ = 5.5 Hz), 41.8 (d, $J_{P\text{–}C}$ = 8.2 Hz), 47.6 (d, $J_{P\text{–}C}$ = 4.5 Hz), 47.7 (d, $J_{P\text{–}C}$ = 69.0 Hz), 128.6 (d, $J_{P\text{–}C}$ = 10.9 Hz), 129.8 (d, $J_{P\text{–}C}$ = 10.0 Hz), 131.5, 135.4 (d, $J_{P\text{–}C}$ = 96.3 Hz); ^{31}P NMR (202 MHz, $CDCl_3$) δ 34.83; GC t_R = 11.23 min; GCMS (EI, 70 eV) *m/z* = 269 (M) (1), 235 (M–SH) (12), 234 (77), 233 (52), 205 (29), 142 (21), 141 (36), 140 (100), 139 (66), 129 (20), 125 (71), 91 (39), 79 (16), 77 (48), 51 (19), 47 (46); HRMS (ESI–TOF) m/z: [M+H] Calcd for $C_{14}H_{21}OPS$ 269.1119; Found 269.1123.

(((1-Mercapto)cycloheptyl)methyl)methylphenylphosphine oxide (57). This compound was prepared according to the general procedure from [(1-hydroxy)cycloheptylmethyl)]methylphenylphosphine sulfide **24** (0.063 g, 0.22 mmol) as a pale yellow oil; yield 0.017 g (27%); R_f 0.36 ($CHCl_3$); ^{1}H NMR (500 MHz, $CDCl_3$) δ 1.40–1.49 (m, 3H), 1.49–1.66 (m, 5H), 1.82 (d, $J_{P\text{–}H}$ = 12.9 Hz, 3H), 1.86–2.02 (m, 4H), 2.45 (d, $J_{P\text{–}H}$ = 10.1 Hz, 2H), 2.97 (bs, 1H), 7.46–7.58 (m, 3H), 7.68–7.81 (m, 2H); ^{13}C NMR (126 MHz, $CDCl_3$) δ 19.4 (d, $J_{P\text{–}C}$ = 70.8 Hz), 23.0 (d, $J_{P\text{–}C}$ = 10.0 Hz), 29.4 (d, $J_{P\text{–}C}$ = 10.9 Hz),

44.1 (d, $J_{P–C}$ = 5.5 Hz), 45.2 (d, $J_{P–C}$ = 8.2 Hz), 47.9 (d, $J_{P–C}$ = 69.0 Hz), 50.1 (d, $J_{P–C}$ = 4.5 Hz), 128.7 (d, $J_{P–C}$ = 10.9 Hz), 129.9 (d, $J_{P–C}$ = 9.1 Hz), 131.5, 135.4 (d, $J_{P–C}$ = 96.3 Hz); ^{31}P NMR (202 MHz, $CDCl_3$) δ 35.20; GC t_R = 11.77 min; GCMS (EI, 70 eV) *m/z* = 248 (M–SH) (20), 154 (12), 141 (25), 140 (100), 139 (31), 125 (47), 91 (18), 79 (10), 77 (21), 47 (22); HRMS (ESI–TOF) m/z: [M+H] Calcd for $C_{15}H_{23}OPS$ 283.1276; Found 283.1280.

(2-Butyl-2-mercaptohexyl)methylphenylphosphine sulfide (58) This compound was prepared according to the general procedure from (2-butyl-2-hydroxyhexyl)methylphenylphosphine sulfide **25** (0.073 g, 0.23 mmol) as a colorless oil; yield 0.027 g (37%); R_f 0.74 ($CHCl_3$:MeOH, 50:1); ^{1}H NMR (500 MHz, $CDCl_3$) δ 0.82 (t, $J_{H–H}$ = 7.3 Hz, 6H), 1.10–1.38 (m, 8H), 1.57–1.66 (m, 2H), 1.66–1.74 (m, 1H), 1.78 (d, $J_{P–H}$ = 12.9 Hz, 3H), 1.81–1.92 (m, 1H), 2.33–2.49 (m, 2H), 2.61 (bs, 1H), 7.46–7.56 (m, 3H), 7.70–7.78 (m, 2H); ^{13}C NMR (126 MHz, $CDCl_3$) δ 14.0, 19.4 (d, $J_{P–C}$ = 69.0 Hz), 22.8, 26.5, 26.8, 42.3 (d, $J_{P–C}$ = 4.5 Hz), 42.4 (d, $J_{P–C}$ = 7.3 Hz), 44.0 (d, $J_{P–C}$ = 67.2 Hz), 50.1 (d, $J_{P–C}$ = 4.5 Hz), 128.6 (d, $J_{P–C}$ = 11.8 Hz), 129.9 (d, $J_{P–C}$ = 9.1 Hz), 131.5 (d, $J_{P–C}$ = 1.8 Hz), 135.4 (d, $J_{P–C}$ = 97.2 Hz); ^{31}P NMR (202 MHz, $CDCl_3$) δ 34.34; GC t_R = 12.07 min; GCMS (EI, 70 eV) *m/z* = 280 (M–SH) (17), 279 (92), 141 (12), 140 (56), 139 (100), 125 (23), 91 (13), 77 (13), 55 (16), 47 (14);); HRMS (ESI–TOF) m/z: [M+H] Calcd for $C_{17}H_{29}OPS$ 313.1738; Found 313.1749.

The rearrangement of (S_P)-(2-hydroxy-2,3,3-trimethylbutyl)methylphenylphosphine sulfide (S_P)–**17** (0.141 g, 0.52 mmol) afforded a mixture of three products.

(2-Mercapto-2,3,3-trimethylbutyl)methylphenylphosphine oxide **66** was isolated as a mixture of diastereomers, partially separated, colorless semisolid; yield 0.131 g (54%).

Major diastereomer: R_f 0.55 ($CHCl_3$/MeOH, 50:1); ^{1}H NMR (500 MHz, $CDCl_3$) δ 1.02 (s, 9H), 1.50 (s, 3H), 1.87 (d, J_{P-H} = 12.9 Hz, 3H), 2.31–2.40 (m, 1H), 2.46–2.53 (m, 1H), 3.06 (bs, 1H), 7.47–7.56 (m, 3H), 7.69–7.77 (m, 2H); ^{13}C NMR (126 MHz, $CDCl_3$) δ 19.6 (d, J_{P-C} = 70.8 Hz), 25.4, 27.1, 38.9 (d, J_{P-C} = 10.0 Hz), 41.0 (d, J_{P-C} = 71.8 Hz), 52.9 (d, J_{P-C} = 4.5 Hz), 128.7 (d, J_{P-C} = 10.9 Hz), 129.7 (d, J_{P-C} = 9.1 Hz), 131.4 (d, J_{P-C} = 2.7 Hz), 135.8 (d, J_{P-C} = 96.3 Hz); ^{31}P NMR (202 MHz, $CDCl_3$) δ 36.41; GC t_R = 13.56 min; GCMS (EI, 70 eV) *m/z* = 237 (M–SH) (6), 236 (2), 235 (3), 214 (7), 213 (54), 155 (9), 140 (25), 139 (100), 125 (12), 91 (8), 77 (13), 47 (10); HRMS (ESI–TOF) m/z: [M+H] Calcd for $C_{14}H_{23}OPS$ 271.1280; Found 271.1284; HPLC (Chiralcel AS–H, Hexane/iPrOH = 9:1, 1.0 mL/min) t_{R1} = 7.72 min, t_{R2} = 8.55 min (65% ee).

Minor diastereomer: R_f 0.45 ($CHCl_3$/MeOH, 50:1); ^{1}H NMR (500 MHz, $CDCl_3$) δ 1.02 (s, 9H), 1.74 (s, 3H), 1.76 (d, J_{P-H} = 12.6 Hz, 3H), 2.34–2.39 (m, 1H), 2.53–2.61 (m, 1H), 3.06 (bs, 1H), 7.47–7.56 (m, 3H), 7.69–7.77 (m, 2H); ^{13}C NMR (126 MHz, $CDCl_3$) δ 19.4 (d, J_{P-C} = 70.8 Hz), 25.4, 27.1, 39.0 (d, J_{P-C} = 7.7 Hz), 41.0 (d, J_{P-C} = 71.8 Hz), 52.7 (d, J_{P-C} = 5.5 Hz), 128.6 (d, J_{P-C} = 10.9 Hz), 130.0 (d, J_{P-C} = 9.1 Hz), 131.4 (d, J_{P-C} = 2.7 Hz), 135.9 (d, J_{P-C} = 94.4 Hz); ^{31}P NMR (202 MHz, $CDCl_3$) δ 35.91; GC t_R = 13.57 min; GCMS (EI, 70 eV) *m/z* = 237 (M–SH) (6), 236 (2), 235 (3), 214 (7), 213 (53), 155 (9), 140 (27), 139 (100), 125 (12), 91 (9), 77 (14), 47 (11); HRMS (ESI–TOF) m/z: [2M+H] Calcd for $C_{28}H_{46}O_2P_2S_2$ 541.2487; Found 541.2501; HPLC (Chiralcel AS–H, Hexane/iPrOH = 9:1, 1.0 mL/min) t_{R1} = 9.75 min, t_{R2} = 10.33 min (65% ee).

(2-(*S*)-Thioacetoxy-2,3,3-trimethylbutyl)methylphenylphosphine oxide 67 was isolated as a mixture with **66**, yield 23% (as calculated from the relative ratios of the mixture in ^{1}H NMR); R_f 0.42 ($CHCl_3$/MeOH, 50:1); ^{1}H NMR (500 MHz, $CDCl_3$) δ 1.01 (s, 9H), 1.70 (s, 3H), 1.79 (d, J_{P-H} = 12.6 Hz, 3H), 2.06 (s, 3H), 2.38–2.45 (m, 1H), 2.54–2.61 (m, 1H), 7.46–7.55 (m,

3H), 7.72–7.79 (m, 2H); ^{13}C NMR (126 MHz, $CDCl_3$) δ 19.1 (d, J_{P-C} = 70.8 Hz), 21.0, 25.4, 27.0, 39.0 (d, J_{P-C} = 10.0 Hz), 40.9 (d, J_{P-C} = 73.6 Hz), 52.6 (d, J_{P-C} = 4.5 Hz), 128.6 (d, J_{P-C} = 10.9 Hz), 130.0 (d, J_{P-C} = 10.0 Hz), 135.0 (d, J_{P-C} = 99.0 Hz), 174.7; GC t_R = 13.58 min; GCMS (EI, 70 eV) *m/z* = 237 (M–SAc) (6), 214 (8), 213 (60), 155 (8), 140 (24), 139 (100), 125 (11), 77 (11), 47 (9); HRMS (ESI–TOF) m/z (title compound underwent acyl group cleavage during measurement): [2M+H] Calcd for $C_{28}H_{46}O_2P_2S_2$ 541.2487; Found 541.2469; HPLC (Chiralcel AS–H, Hexane/iPrOH = 95:5, 1.0 mL/min) t_{R1} = 20.35 min, t_{R2} = 21.70 min (78% ee).

(3-(*S*)-Thioacetoxy-2,2,3-trimethylbutyl)methylphenylphosphine oxide **68** was isolated as a mixture with **66**, yield 15% (as calculated from the relative ratios of the mixture in ^{1}H NMR); R_f 0.42 ($CHCl_3$/MeOH, 50:1); ^{1}H NMR (500 MHz, $CDCl_3$) δ 0.96 (s, 3H), 1.35 (s, 3H), 1.36 (s, 3H), 1.38 (s, 3H), 1.74 (d, J_{P-H} = 12.6 Hz, 3H), 2.06 (s, 3H), 2.29–2.43 (m, 2H), 7.46–7.55 (m, 3H), 7.72–7.79 (m, 2H); ^{13}C NMR (126 MHz, $CDCl_3$) δ 19.3 (d, J_{P-C} = 71.8 Hz), 21.0, 22.9, 23.2, 28.1, 28.3, 38.3 (d, J_{P-C} = 70.0 Hz), 41.2 (d, J_{P-C} = 4.5 Hz), 53.7 (d, J_{P-C} = 13.6 Hz), 128.6 (d, J_{P-C} = 10.9 Hz), 129.9 (d, J_{P-C} = 10.0 Hz), 134.9 (d, J_{P-C} = 96.3 Hz), 174.7; GC t_R = 13.83 min; GCMS (EI, 70 eV) *m/z* = 237 (M–SAc) (6), 196 (12), 195 (55), 140 (29), 139 (100), 125 (9), 91 (9), 77 (10); HRMS (ESI–TOF) m/z (title compound underwent acyl group cleavage during measurement): [2M+H] Calcd for $C_{28}H_{46}O_2P_2S_2$ 541.2487; Found 541.2485; HPLC (Chiralcel AS–H, Hexane/iPrOH = 95:5, 1.0 mL/min) t_{R1} = 18.17 min, t_{R2} = 26.51 min (76% ee).

General procedure for the synthesis of mesylate derivatives and unsaturated phosphine sulfides from β-hydroxyalkylphosphine sulfides

To a solution of β-hydroxyalkylphosphine sulfide (1.0 mmol) in dry toluene containing Et_3N (20.0 mmol) at 0 °C mesyl chloride (1.5 mmol) was slowly added. The reaction was allowed to stir at this temp for 0.5 h prior to heating to 90°C. The progress of the reaction was monitored by TLC. The reaction mixture was then diluted with 1 M HCl and extracted with DCM (3 × 20 mL). The combined organic phases were dried over $MgSO_4$, filtered, and evaporated under reduced pressure. The residue was purified by column chromatography using $CHCl_3$ as eluent.

(2-Ethylbut-2-enyl)(methylphenyl)phosphine sulfide (59). This compound was prepared according to the general procedure from (2-ethyl-2-hydroxybutyl)methylphenylphosphine sulfide **20** (0.107 g, 0.42 mmol) as a pale yellow oil; yield 0.013 g (13%); Isolated as a mixture of diastereomers (dr = 52:48); R_f 0.59 ($CHCl_3$).

Major diastereomer: ^{1}H NMR (500 MHz, $CDCl_3$) δ 0.88 (t, $J_{H–H}$ = 7.6 Hz, 3H), 1.60 (t, $J_{H–H}$ = 6.8 Hz, 3H), 1.93–2.04 (m, 1H), 2.00 (d, $J_{P–H}$ = 12.6 Hz, 3H), 2.07–2.16 (m, 1H), 2.83–3.09 (m, 2H), 5.17–5.25 (m, 1H), 7.45–7.56 (m, 3H), 7.84–7.94 (m, 2H); ^{13}C NMR (126 MHz, $CDCl_3$) δ 12.4 (d, $J_{P–C}$ = 3.6 Hz),13.4 (d, $J_{P–C}$ = 2.7 Hz), 19.7 (d, $J_{P–C}$ = 57.2 Hz), 30.8, 43.6 (d, $J_{P–C}$ = 50.9 Hz), 125.0 (d, $J_{P–C}$ = 11.8 Hz), 128.4 (d, $J_{P–C}$ = 10.9 Hz), 130.7 (d, $J_{P–C}$ = 10.9 Hz), 131.4 (d, $J_{P–C}$ = 2.7 Hz), 132.8 (d, $J_{P–C}$ = 75.4 Hz), 133.2 (d, $J_{P–C}$ = 10.9 Hz); ^{31}P NMR (202 MHz, $CDCl_3$) δ 37.30; GC t_R = 10.32 min; GCMS (EI, 70 eV) *m/z* = 238 (M) (27), 157 (13), 156 (100), 155 (66), 153 (11), 140 (20), 124 (11), 121 (11), 109 (10), 91 (16), 78 (26), 77 (23), 63 (21), 55 (15).

Minor diastereomer: ^{1}H NMR (500 MHz, $CDCl_3$) δ 0.92 (t, J_{H-H} = 7.3 Hz, 3H), 1.45 (dd, J_{H-H} = 6.6 Hz, J_{H-H} = 5.7 Hz, 3H), 1.93–2.04 (m, 1H), 1.97 (d, J_{P-H} = 12.9 Hz, 3H), 2.05–2.14 (m, 1H), 2.81–3.09 (m, 2H), 5.41–5.49 (m, 1H), 7.45–7.56 (m, 3H), 7.84–7.95 (m, 2H); ^{13}C NMR (126 MHz, $CDCl_3$) δ 12.6 (d, J_{P-C} = 1.8 Hz), 13.4 (d, J_{P-C} = 2.7 Hz), 20.2 (d, J_{P-C} = 57.2 Hz), 23.9, 38.6 (d, J_{P-C} = 50.9 Hz), 122.7 (d, J_{P-C} = 10.9 Hz), 128.5 (d, J_{P-C} = 10.9 Hz), 130.6 (d, J_{P-C} = 10.0 Hz), 131.5 (d, J_{P-C} = 3.6 Hz), 133.2 (d, J_{P-C} = 76.3 Hz), 133.2 (d, J_{P-C} = 10.9 Hz); ^{31}P NMR (202 MHz, $CDCl_3$) δ 37.89; GC t_R = 10.24 min; GCMS (EI, 70 eV) *m/z* = 238 (M) (28), 157 (13), 156 (100), 155 (73), 153 (12), 141 (17), 124 (12), 121 (12), 109 (11), 91 (17), 78 (25), 77 (25), 63 (20), 55 (19).

HRMS (ESI–TOF) m/z (the analysis has been performed for diastereomeric mixture): [M+H] Calcd for $C_{13}H_{19}OPS$ 239.1018; Found 239.1018.

(2-Mesyloxy-3-methylbutyl)methylphenylphosphine sulfide (60). This compound was prepared according to the general procedure from (2-hydroxy-3-methylbutyl)methylphenylphosphine sulfide **8** (0.177 g, 0.73 mmol) as sticky solid; yield 0.182 g (57%). Isolated as a mixture of diastereomers (dr = 52.5:47.5); R_f 0.57 (Hex:$CHCl_3$:iPrOH, 10:1:1).

Major diastereomer: ^{1}H NMR (500 MHz, $CDCl_3$) δ 0.98-1.01 (m, 6H), 2.05 (d, J_{P-H} = 13.2 Hz, 3H), 2.20–2.36 (m, 2H), 2.69–2.80 (m, 1H), 3.14 (s, 3H), 5.08–5.20 (m, 1H), 7.44–7.62 (m, 3H), 7.81–8.00 (m, 2H); ^{13}C NMR (126 MHz, $CDCl_3$) δ 16.4, 17.2, 21.8 (d, J_{P-C} = 57.2 Hz), 32.0 (d, J_{P-C} = 7.3 Hz), 35.2 (d, J_{P-C} = 51.8 Hz), 39.0, 83.1, 128.8 (d, J_{P-C} = 10.9 Hz), 130.3 (d, J_{P-C} = 10.0 Hz), 131.9 (d, J_{P-C} = 2.7 Hz), 132.6 (d, J_{P-C} = 79.9 Hz); ^{31}P NMR (202 MHz, $CDCl_3$) δ 35.97; GC t_R = 9.69 min; GCMS (EI, 70 eV) *m/z* = 225 (M–OMs) (4), 224

(31), 157 (10), 155 (100), 141 (24), 124 (31), 123 (16), 121 (21),109 (17),107 (10), 91 (16), 79 (11), 78 (34), 77 (21), 63 (27), 51 (11).

Minor diastereomer: ^{1}H NMR (500 MHz, $CDCl_3$) δ 0.76 (d, $J_{H–H}$ = 6.9 Hz, 3H), 0.95 (d, $J_{H–H}$ = 6.6 Hz, 3H), 1.98 (d, $J_{P–H}$ = 12.9 Hz, 3H), 2.20–2.37 (m, 2H), 2.54–2.60 (m, 1H), 2.61 (s, 3H), 4.91–5.00 (m, 1H), 7.46–7.62 (m, 3H), 7.81–8.00 (m, 2H); ^{13}C NMR (126 MHz, $CDCl_3$) δ 16.7, 17.1, 23.1 (d, $J_{P–C}$ = 59.0 Hz), 32.2 (d, $J_{P–C}$ = 8.2 Hz), 34.1 (d, $J_{P–C}$ = 54.5 Hz), 38.3, 82.4 (d, $J_{P–C}$ = 2.7 Hz), 128.7 (d, $J_{P–C}$ = 11.8 Hz), 130.7 (d, $J_{P–C}$ = 10.9 Hz), 131.5 (d, $J_{P–C}$ = 74.5 Hz), 131.9 (d, $J_{P–C}$ = 1.8 Hz); ^{31}P NMR (202 MHz, $CDCl_3$) δ 36.01; GC t_R = 9.88 min; GCMS (EI, 70 eV) *m/z* = 225 (M–OMs) (3), 224 (28), 157 (10), 155 (100), 141 (41), 123 (23), 121 (14), 109 (18),107 (10), 91 (19), 78 (50), 77 (22), 63 (37), 51 (11).

HRMS (ESI–TOF) m/z (the analysis has been performed for diastereomeric mixture): [M+H] Calcd for $C_{13}H_{21}O_3PS_2$ 321.0754; Found 321.0742.

(*S*P)-(2-Mesyloxy-3-methylbutyl)methylphenylphosphine sulfide (*S*P)-60. This compound was prepared according to the general procedure from (*S*P)-(2-hydroxy-3-methylbutyl)methylphenylphosphine sulfide (*S*P)-**8** (0.176g, 0.73 mmol) as sticky solid; yield 0.162 g (68%). Isolated as a mixture of diastereomers (dr = 52.5:47.5); HPLC (Chiralcel OJ–H, Hexane/iPrOH = 98:2, 1.0 mL/min) t_{R1} = 31.53 min, t_{R2} = 33.24 min, t_{R3} = 39.32 min, t_{R4} = 46.08 min (71% ee).

(2-Mesyloxy-3,3-dimethylbutyl)methylphenylphosphine sulfide (61). This compound was prepared according to the general procedure from (2-hydroxy-3,3-dimethylbutyl)methylphenylphosphine sulfide **9** (0.219 g, 0.86 mmol) as a pale yellow solid;

yield 0.244 g (85%). Isolated as a mixture of diastereomers (dr = 52:48); R_f 0.62 (Hex/$CHCl_3$/iPrOH, 10:1:1).

Major diastereomer: ^{1}H NMR (500 MHz, $CDCl_3$) δ 1.02 (s, 9H), 1.99 (d, $J_{P\text{–}H}$ = 12.9 Hz, 3H), 2.52–2.60 (m, 1H), 2.61 (s, 3H), 2.66–2.78 (m, 1H), 4.94–5.01 (m, 1H), 7.46–7.59 (m, 3H), 7.86–7.99 (m, 2H); ^{13}C NMR (126 MHz, $CDCl_3$) δ 22.9 (d, $J_{P\text{–}C}$ = 59.0 Hz), 26.0, 35.8 (d, $J_{P\text{–}C}$ = 53.6 Hz), 35.9 (d, $J_{P\text{–}C}$ = 9.1 Hz), 39.4, 85.3 (d, $J_{P\text{–}C}$ = 4.5 Hz), 128.8 (d, $J_{P\text{–}C}$ = 12.7 Hz), 130.6 (d, $J_{P\text{–}C}$ = 10.0 Hz), 131.8 (d, $J_{P\text{–}C}$ = 2.7 Hz), 132.4 (d, $J_{P\text{–}C}$ = 78.1 Hz); ^{31}P NMR (202 MHz, $CDCl_3$) δ 36.59; GC t_R = 8.83 min; GCMS (EI, 70 eV) *m/z* = 239 (M–OMs) (4), 238 (22), 157 (11), 156 (100), 155 (24), 141 (30), 123 (17), 121 (11), 109 (11), 91 (15), 78 (37), 77 (15), 63 (22).

Minor diastereomer: ^{1}H NMR (500 MHz, $CDCl_3$) δ 0.89 (s, 9H), 2.11 (d, $J_{P\text{–}H}$ = 13.2 Hz, 3H), 2.33–2.47 (m, 2H), 3.22 (s, 3H), 5.01–5.07 (m, 1H), 7.49–7.58 (m, 3H), 7.86–7.98 (m, 2H); ^{13}C NMR (126 MHz, $CDCl_3$) δ 21.9 (d, $J_{P\text{–}C}$ = 59.0 Hz), 25.7, 35.8 (d, $J_{P\text{–}C}$ = 8.2 Hz), 37.1 (d, $J_{P\text{–}C}$ = 52.7 Hz), 38.9, 85.2 (d, $J_{P\text{–}C}$ = 2.7 Hz), 128.7 (d, $J_{P\text{–}C}$ = 11.8 Hz), 130.4 (d, $J_{P\text{–}C}$ = 10.0 Hz), 131.8 (d, $J_{P\text{–}C}$ = 2.7 Hz), 132.8 (d, $J_{P\text{–}C}$ = 79.0 Hz); ^{31}P NMR (202 MHz, $CDCl_3$) δ 37.45; GC t_R = 8.77 min; GCMS (EI, 70 eV) *m/z* = 239 (M–OMs) (3), 238 (23), 157 (11), 156 (100), 155 (24), 141 (31), 123 (18), 121 (11), 109 (11), 91 (16), 78 (38), 77 (15), 63 (22).

HRMS (ESI–TOF) m/z (the analysis has been performed for diastereomeric mixture): [M+H] Calcd for $C_{14}H_{23}O_3PS_2$ 335.0893; Found 335.0899.

(2-Methylbut-2-enyl)(methylphenyl)phosphine sulfide (62). This compound was prepared according to the general procedure from (2-hydroxy-2-methylbutyl)methylphenylphosphine sulfide **12**; yield 85%, isolated as a mixture with **63**; R_f 0.56 ($CHCl_3$); Isolated as a mixture of *cis*- and *trans*-isomers.

trans-Isomer: ^{1}H NMR (500 MHz, $CDCl_3$) δ 1.57–1.62 (m, 36), 1.96 (d, $J_{P–H}$ = 12.9 Hz, 3H), 2.89 (d, $J_{P–H}$ = 14.5 Hz, 2H), 5.19–5.26 (m, 1H), 7.45–7.55 (m, 3H), 7.85–7.94 (m, 2H); ^{13}C NMR (126 MHz, $CDCl_3$) δ 13.78 (d, $J_{P–C}$ = 3.6 Hz), 17.89 (d, $J_{P–C}$ = 1.8 Hz), 19.87 (d, $J_{P–C}$ = 57.2 Hz), 47.01 (d, $J_{P–C}$ = 50.0 Hz), 125.52 (d, $J_{P–C}$ = 10.9 Hz), 127.18 (d, $J_{P–C}$ = 10.9 Hz), 128.4 (d, $J_{P–C}$ = 10.9 Hz), 130.69 (d, $J_{P–C}$ = 10.2 Hz), 131.4 (d, $J_{P–C}$ = 2.7 Hz), 133.2 (d, $J_{P–C}$ = 75.4 Hz); ^{31}P NMR (202 MHz, $CDCl_3$) δ 36.92.

cis-Isomer: ^{1}H NMR (500 MHz, $CDCl_3$) δ 1.40–1.44 (m, 3H), 1.67–1.70 (m, 3H), 2.01 (d, $J_{P–H}$ = 12.6 Hz, 3H), 3.03 (d, $J_{P–H}$ = 14.5 Hz, 2H), 5.42–5.49 (m, 1H), 7.45–7.55 (m, 3H), 7.85–7.94 (m, 2H); ^{13}C NMR (126 MHz, $CDCl_3$) δ 14.3 (d, $J_{P–C}$ = 2.7 Hz), 20.4 (d, $J_{P–C}$ = 56.3 Hz), 25.4 (d, $J_{P–C}$ = 1.8 Hz), 40.4 (d, $J_{P–C}$ = 50.0 Hz), 124.7 (d, $J_{P–C}$ = 10.9 Hz), 127.41 (d, $J_{P–C}$ = 10.9 Hz), 128.5 (d, $J_{P–C}$ = 10.9 Hz), 130.6 (d, $J_{P–C}$ = 10.0 Hz), 131.5 (d, $J_{P–C}$ = 2.7 Hz), 133.19 (d, $J_{P–C}$ = 75.7 Hz); ^{31}P NMR (202 MHz, $CDCl_3$) δ 37.50.

GC *(peak 1)* t_R = 9.90 min; GCMS (EI, 70 eV) *m/z* = 224 (M) (37), 157 (13), 156 (100), 155 (99), 153 (19), 141 (13), 124 (120), 123 (11), 121 (19), 109 (15), 107 (12), 91 (26), 78 (24), 77 (38), 63 (25), 51 (14); GC *(peak 2)* t_R = 10.09 min; GCMS (EI, 70 eV) *m/z* = 224 (M) (44), 157 (12), 156 (100), 155 (88), 153 (17), 141 (20), 124 (13), 121 (14), 109 (15), 107 (12), 91 (23), 78 (30), 77 (35), 63 (29), 51 (14).

HRMS (ESI–TOF) m/z (the analysis has been performed for the mixture of isomers): [M+H] Calcd for $C_{12}H_{18}PS$ 225.0861; Found 225.0853.

(2-Ethylprop-2-enyl)(methylphenyl)phosphine sulfide (63). This compound was prepared according to the general procedure from (2-hydroxy-2-methylbutyl)methylphenylphosphine sulfide **12**; yield 85%, isolated as a mixture with **62**; R_f 0.56 ($CHCl_3$).

^{1}H NMR (500 MHz, $CDCl_3$) δ 0.95 (t, $J_{H–H}$ = 7.3 Hz, 3H), 1.53–1.62 (m, 1H), 2.00 (d, $J_{P–H}$ = 12.9 Hz, 3H), 2.03–2.12 (m, 1H), 2.95 (d, $J_{P–H}$ = 14.8 Hz, 2H), 4.75–4.77 (m, 1H), 4.92–4.95 (m, 1H), 7.45–7.55 (m, 3H), 7.85–7.94 (m, 2H); ^{13}C NMR (126 MHz, $CDCl_3$) δ 12.0 (d, $J_{P–C}$ = 1.8 Hz), 20.2 (d, $J_{P–C}$ = 57.2 Hz), 30.1 (d, $J_{P–C}$ = 1.8 Hz), 43.9 (d, $J_{P–C}$ = 50.0 Hz), 113.9 (d, $J_{P–C}$ = 10.0 Hz), 128.5 (d, $J_{P–C}$ = 11.8 Hz), 130.7 (d, $J_{P–C}$ = 10.0 Hz), 131.5 (d, $J_{P–C}$ = 2.7 Hz), 132.6 (d, $J_{P–C}$ = 77.2 Hz), 142.56 (d, $J_{P–C}$ = 10.0 Hz); ^{31}P NMR (202 MHz, $CDCl_3$) δ 36.70; GC t_R = 10.05 min; GCMS (EI, 70 eV) *m/z* = 224 (M) (46), 157 (12), 156 (100), 155 (96), 153 (18), 141 (17), 124 (12), 121 (14), 109 (13), 107 (11), 91 (22), 78 (25), 77 (35), 63 (26), 51 (14). HRMS (ESI–TOF) m/z: [M+H] Calcd for $C_{12}H_{18}PS$ 225.0861; Found 225.0858.

(2,3-Dimethylbut-2-enyl)(methylphenyl)phosphine sulfide (64). This compound was prepared according to the general procedure from (2-hydroxy-2,3-dimethylbutyl)methylphenylphosphine sulfide **16** (0.115 g, 0.45 mmol) as a pale yellow oil; yield 0.038 g (35%); R_f 0.66 ($CHCl_3$); ^{1}H NMR (500 MHz, $CDCl_3$) δ 1.45 (d, $J_{H–H}$ = 3.8 Hz, 3H) 1.64 (s, 3H) 1.65 (s, 3H) 1.98 (d, $J_{P–H}$ = 13.6 Hz, 3H) 2.93–3.02 (m, 1H) 3.04–3.13 (m, 1H) 7.43–7.54 (m, 3H) 7.84–7.92 (m, 2H); ^{13}C NMR (126 MHz, $CDCl_3$) δ 20.0 (d, $J_{P–C}$ = 55.4 Hz), 20.6, 20.7 (d, $J_{P–C}$ = 2.7 Hz) 21.3 (d, $J_{P–C}$ = 3.6 Hz) 43.4 (d, $J_{P–C}$ = 50.9 Hz) 119.1 (d, $J_{P–C}$ = 11.8 Hz) 128.3 (d, $J_{P–C}$ = 11.8 Hz) 130.6 (d, $J_{P–C}$ = 10.0 Hz) 130.7 (d, $J_{P–C}$ = 11.8 Hz) 131.3 (d, $J_{P–C}$ = 2.7 Hz) 133.1 (d, $J_{P–C}$ = 75.4 Hz); ^{31}P NMR (202 MHz, $CDCl_3$) δ 37.96; GC t_R = 10.55 min; GCMS (EI, 70 eV) *m/z* = 238 (3), 157 (11), 156 (100), 155 (41), 141 (26), 124 (69), 123 (13), 121 (15), 109 (16), 108 (13), 107 (11), 91 (16), 83 (28), 79 (11), 78 (38), 77 (28), 63 (25) 55 (70), 51 (13); HRMS (ESI–TOF) m/z: [M+H] Calcd for $C_{13}H_{19}OPS$ 239.1009; Found 239.1018. Analytical data are in accordance with those reported in the literature [8].

(2-Methylprop-2-enyl)(methylphenyl)phosphine oxide (65). This compound was prepared according to the general procedure from (2-hydroxy-2-methylpropyl)methylphenylphosphine sulfide **18** (0.186 g, 0.82 mmol) as a pale yellow oil; yield 0.147 g (85%); R_f 0.33 (Hex/AcOEt, 6:1); ^{1}H NMR (500 MHz, $CDCl_3$) δ 1.69–1.74 (m, 3H), 2.00 (d, $J_{P–H}$ = 12.9 Hz, 3H), 2.94 (d, $J_{P–H}$ = 14.8 Hz, 2H), 4.64–4.75 (m, 1H), 4.91–4.95 (m, 1H), 7.44–7.55 (m, 3H), 7.85–7.93 (m, 2H); ^{13}C NMR (126 MHz, $CDCl_3$) δ 20.0 (d, $J_{P–C}$ = 57.2 Hz), 24.3 (d, $J_{P–C}$ = 1.8 Hz), 45.1 (d, $J_{P–C}$ = 50.0 Hz), 116.3 (d, $J_{P–C}$ = 10.9 Hz), 128.4 (d, $J_{P–C}$ = 11.8 Hz), 130.6 (d, $J_{P–C}$ = 10.0 Hz), 131.5 (d, $J_{P–C}$ = 3.6 Hz), 132.5 (d, $J_{P–C}$ = 76.3 Hz), 136.9 (d, $J_{P–C}$ = 10.0 Hz); ^{31}P NMR (202 MHz, $CDCl_3$) δ 36.34; GC t_R = 8.33 min; GCMS (EI, 70 eV) *m/z* = 210 (28), 155 (100), 153 (13), 91 (14), 77 (19), 63 (11); HRMS (ESI–TOF) m/z: [M+H] Calcd for $C_{11}H_{15}OPS$ 211.0697; Found 211.0705.

(S_P)-(2-Methylprop-2-enyl)(methylphenyl)phosphine oxide (S_P)-65. This compound was prepared according to the general procedure from (S_P)-(2-hydroxy-2-methylpropyl)methylphenylphosphine sulfide (S_P)-**19** (0.107 g, 0.47 mmol, 67% ee) as a pale yellow oil; yield 0.079 g (80%); R_f 0.74 (Hex:AcOEt, 2:1); ^{1}H NMR (500 MHz, $CDCl_3$) δ 1.69–1.73 (m, 3H), 2.00 (d, $J_{P–H}$ = 12.6 Hz, 3H), 2.94 (d, $J_{P–H}$ = 15.1 Hz, 2H), 4.66–4.73 (m, 1H), 4.90–4.95 (m, 1H), 7.44–7.54 (m, 3H), 7.85–7.94 (m, 2H); ^{13}C NMR (126 MHz, $CDCl_3$) δ 20.0 (d, $J_{P–C}$ = 59.0 Hz), 24.3, 45.1 (d, $J_{P–C}$ = 51.8 Hz), 116.3 (d, $J_{P–C}$ = 10.9 Hz), 128.4 (d, J_{P-C} = 12.7 Hz), 130.6 (d, $J_{P–C}$ = 10.0 Hz), 131.5 (d, $J_{P–C}$ = 2.7 Hz), 132.4 (d, $J_{P–C}$ = 77.2 Hz), 136.9 (d, $J_{P–C}$ = 10.9 Hz); ^{31}P NMR (202 MHz, $CDCl_3$) δ 36.37; GC t_R = 9.37 min; GCMS (EI, 70 eV) *m/z* = 210 (25), 155 (100), 153 (13), 91 (17), 77 (22), 63 (13); HRMS (ESI–TOF)

m/z: [M+H] Calcd for $C_{11}H_{15}OPS$ 211.0692; Found 211.0703; HPLC (Chiralcel OJ–H, Hexane/iPrOH = 95:5, 0.5 mL/min) t_{R1} = 31.18 min, t_{R2} = 37.39 min (65% ee).

1,4,4-Trimethyl-1,2,3,4-tetrahydrophosphinoline-1-oxide (26)

$AlCl_3$ (0.087 g, 0.65 mmol) was added to a solution of enantiomerically enriched (3-methyl-3-mercaptobutyl)methylphenylphosphine oxide **46** (0.031 g, 0.13 mmol) in dichloroethane (5 mL). The reaction mixture was stirred at room temperature for 24 h. The mixture was quenched with water (5 mL) and 20% $NaOH_{aq}$ and extracted with DCM (3 × 10 mL). The combined organic phases were dried over $MgSO_4$, filtered, and evaporated under reduced pressure. The residue was purified by column chromatography using $CHCl_3$ as eluent affording **26** as a pale yellow oil; yield 0.025 g (95%); R_f 0.27 (AcOEt/MeOH, 9:1); ^{1}H NMR (500 MHz, $CDCl_3$) δ 1.35 (s, 3H), 1.36 (s, 3H), 1.70 (d, $J_{P–H}$ = 12.6 Hz, 3H), 1.90–2.01 (m, 1H) 2.07–2.19 (m, 2H), 2.24–2.39 (m, 1H), 7.30–7.36 (m, 1H), 7.38–7.49 (m, 2H), 7.78–7.86 (m, 1H); ^{13}C NMR (126 MHz, $CDCl_3$) δ 17.4 (d, $J_{P–C}$ = 70.8 Hz), 23.4 (d, $J_{P–C}$ = 69.0 Hz), 30.9, 30.9, 34.9 (d, $J_{P–C}$ = 3.6 Hz), 35.5 (d, $J_{P–C}$ = 5.5 Hz), 126.5 (d, $J_{P–C}$ = 10.9 Hz), 126.6 (d, $J_{P–C}$ = 9.1 Hz), 129.1 (d, $J_{P–C}$ = 93.6 Hz), 130.5 (d, $J_{P–C}$ = 7.3 Hz), 131.9 (d, $J_{P–C}$ = 1.8 Hz), 150.6 (d, $J_{P–C}$ = 8.2 Hz); ^{31}P NMR (202 MHz, $CDCl_3$) δ 29.63; GC t_R = 10.58 min; GCMS (EI, 70 eV) *m/z* = 208 (M) (24), 207 (13), 194 (12), 193 (100), 179 (13), 178 (21), 165 (24), 131 (16), 130 (25), 129 (22), 128 (14), 116 (14), 115 (26), 91 (18), 78 (30), 77 (15), 63 (12); HRMS (ESI–TOF) m/z: [M+H] Calcd for $C_{12}H_{17}OP$ 209.1095, found 209.1090; HPLC (Chiralcel OJ–H, Hexane/iPrOH = 98:2, 1.0 mL/min) t_{R1} = 34.94 min, t_{R2} = 37.54 min (62% ee).

Synthesis of 1,3,3-trimethylphosphindoline-1-oxide (3)

$AlCl_3$ (0.100 g, 0.75 mmol) was added to a solution of enantiomerically enriched (2-methyl-2-mercaptopropyl)methylphenylphosphine oxide **53** (0.035 g, 0.15 mmol) in dichloroethane (5 mL). The reaction mixture was stirred at room temperature for 24 h. The mixture was quenched with water (5 mL) and 20% $NaOH_{aq}$ and extracted with DCM (3 × 10 mL). The combined organic phases were dried over $MgSO_4$, filtered, and evaporated under reduced pressure. The residue was purified by column chromatography using $CHCl_3$ as eluent affording **3** as a sticky yellow solid; yield 0.025 g (83%); R_f 0.38 ($CHCl_3$); ^{1}H NMR (500 MHz, $CDCl_3$) δ 1.39 (s, 3H), 1.50 (s, 3H), 1.81 (d, $J_{P–H}$ = 12.9 Hz, 3H), 2.08–2.17 (m, 1H), 2.20–2.32 (m, 1H), 7.33–7.43 (m, 2H), 7.50–7.58 (m, 1H), 7.67–7.75 (m, 1H); ^{13}C NMR (126 MHz, $CDCl_3$) δ 18.6 (d, $J_{P–C}$ = 69.0 Hz), 31.3 (d, $J_{P–C}$ = 5.5 Hz), 32.6 (d, $J_{P–C}$ = 4.5 Hz), 41.6 (d, $J_{P–C}$ = 3.6 Hz), 42.5 (d, $J_{P–C}$ = 68.1 Hz), 123.8 (d, $J_{P–C}$ = 12.7 Hz), 127.7 (d, $J_{P–C}$ = 8.2 Hz), 127.8 (d, $J_{P–C}$ = 7.3 Hz), 132.5 (d, $J_{P–C}$ = 99.0 Hz), 133.0 (d, $J_{P–C}$ = 1.8 Hz), 155.3 (d, $J_{P–C}$ = 27.3 Hz); ^{31}P NMR (202 MHz, $CDCl_3$) δ 53.40; GC t_R = 9.43 min; GCMS (EI, 70 eV) *m/z* = 194 (M) (51), 193 (46), 180 (11), 179 (100), 133 (16), 116 (11), 115 (31), 91 (14); HRMS (ESI–TOF) m/z: [M+H] Calcd for $C_{11}H_{15}OP$ 195.0924; Found 195.0933; HPLC (Chiralcel OJ–H, Hexane/iPrOH = 95:5, 1.0 mL/min) t_{R1} = 12.12 min, t_{R2} = 16.45 min (56% ee).

Mechanism for the Rearrangement of 16 in the presence of Brønsted acid

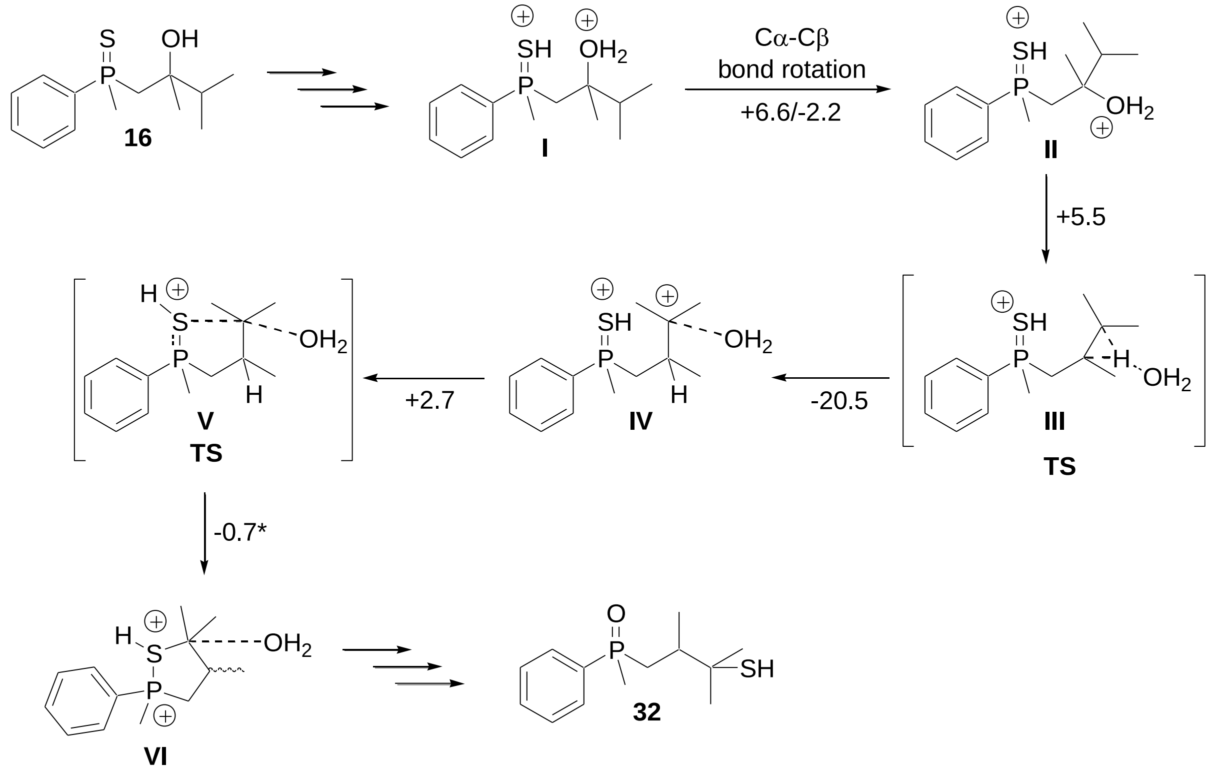

Figure S1: Rearrangement of **16** in the presence of Brønsted acid. The calculated energies next to the arrows are reported in kcal·mol^{-1}

Coordinates of Computed Compounds

Compound 20

p	-1.10120671966386	0.82521916136237	0.06149972946654
s	-0.76911014737305	2.78640719893859	0.06842905336924
h	1.47828845682363	2.08958438893331	-0.43401276430212
h	-2.72673385274634	1.27747805079217	4.72342498759360
h	-2.48336020062067	-0.76388719884539	-1.19984972857599
c	-1.82794334118304	0.19221765965561	1.63097467519423
c	-2.17446350655407	-1.15915427933133	1.76821791615640
c	-2.71470656945609	-1.62742622442634	2.96295746907587
c	-2.91465146016214	-0.75071068178887	4.02935360631895
c	-2.57259339000114	0.59285538032393	3.89707587236454
c	-2.03036581422094	1.06484092190222	2.70232157389523
h	-1.76110886060514	2.10891572251654	2.59000528188261
h	-2.02594170355929	-1.85610910057432	0.95042311988060
h	-2.97966895101765	-2.67414395982416	3.06101825936655
h	-3.33561608139181	-1.11613035278001	4.95926640490453
c	-2.31450893930892	0.31492144928255	-1.21857909659751
h	-3.25252687993733	0.83266921802935	-1.01391396721955
h	-1.95174790036019	0.61245121998063	-2.20144363415062
c	0.41258454144005	-0.22542295557951	-0.15378390214801
h	0.83489195997792	-0.29938551604066	0.84985099065958
h	0.06785715116435	-1.22568197285920	-0.43873236185603
c	1.53898317120063	0.24561145405678	-1.11905186471964
h	3.60034128999194	-0.31368057900420	0.72088302339012
h	0.44890874166317	-0.38694448622959	-2.88269670506652
c	0.98263648955232	0.50894553735377	-2.54065653568526
h	0.24263231968790	1.31127590965531	-2.46356895782115
h	2.69418176930261	0.10173698268822	-3.85272110659188
c	2.02404015181256	0.92220122665649	-3.58592744512199
h	1.52694650501379	1.24827855603764	-4.50351011778506
h	2.63096587386894	1.75191128439931	-3.21757131399672
c	2.64579742920016	-0.83126923817294	-1.15943854635809
h	2.24638361070812	-1.72404242737153	-1.65542586651553
h	3.43794002584969	-0.44238806444469	-1.80335665809697
c	3.26356330537469	-1.20692665420649	0.19128824416743
h	2.56618786427530	-1.74672565538104	0.83787796523148
h	4.12938710262350	-1.85722238513804	0.03965534731515
o	2.16373655863038	1.41973040943362	-0.60028294762401

Intermediate I, Scheme 9

p	-1.20417839853380	0.60997375848749	0.05337987828070
s	-0.94042951328451	2.68931993264153	0.13209811910660
h	0.35065586357951	2.57857949518837	-0.30919939317614
h	-1.67878472587988	0.54788973959206	4.95680700425941
h	-2.39965000780206	-0.89811053443868	-1.42663579280023
c	-1.95244136127034	0.05712663114967	1.60628771732092
c	-2.94652752876790	-0.93250917734697	1.60458122360643
c	-3.47172759481503	-1.38553952933478	2.81174107235564
c	-3.01364944199678	-0.85673679264521	4.01665320080023
c	-2.02704225390925	0.12915327504071	4.02041990521917
c	-1.49465482777709	0.58921142368392	2.82106156241141
h	-0.74644448113643	1.37308505387543	2.83274077336871
h	-3.32173027945384	-1.35254115436643	0.67984346513194
h	-4.24306839135971	-2.14607588135743	2.80778328542332
h	-3.42957380984288	-1.20833979400371	4.95336336075556
c	-2.33342762682486	0.18511627672750	-1.30915539021183
h	-3.32145493483689	0.59169805288695	-1.08692316876177
h	-1.96765683125294	0.62573853569933	-2.23450741983036
c	0.41805336470779	-0.25107136526631	-0.11264176032771
h	0.83195344926815	-0.22271814362551	0.89784953228955
h	0.15040090064238	-1.29480240664898	-0.31341172385275
c	1.49589362662113	0.23051552533270	-1.13080190475013
h	3.69681431222221	0.08317826660853	0.76118107711384
h	0.44493827969292	-0.65151344236497	-2.79288663633073
c	0.93323360127484	0.30253555994798	-2.56403790594114
h	0.15445145161665	1.07190721484689	-2.57397288325607
h	2.65924561197528	-0.16287305709825	-3.83483680950063
c	1.94538904696406	0.64424126905582	-3.66198693235013
h	1.42101143478582	0.81770483353137	-4.60394174956615
h	2.49901883374343	1.55259803100753	-3.41721310050826
c	2.71485531052794	-0.71427885946822	-1.04690619857830
h	2.40822056316996	-1.69730927905765	-1.41923862390554
h	3.45703629223585	-0.33840949926949	-1.75442900801813
c	3.37654760806884	-0.87165739517690	0.32843895956399
h	2.73454125823071	-1.37097304188217	1.05769952473021
h	4.27915108637323	-1.47753149846647	0.23032458660744
o	1.87925112348635	1.58114033166927	-0.78496260839149
h	2.64177898955708	1.57227764484526	-0.19456523828764

Transition state II, Scheme 9

p	-0.96952966478715	-0.25659828901031	0.12597858600132
s	-1.07652790780846	1.84906141585330	0.38604833581928
h	0.20447419991438	2.00785695680878	-0.12118266794059
h	-0.92487886979934	-1.02830586592550	4.98243230304628
h	-1.88981351152058	-1.83602578902823	-1.45146573630944
c	-1.53446727467069	-1.05403500206979	1.63912740882606
c	-2.47553527730294	-2.09470130683434	1.58812158369163
c	-2.84624882370929	-2.74447420419797	2.76248943335149
c	-2.28781647165272	-2.36265517668621	3.98043984235492
c	-1.35456203111012	-1.32536107815857	4.03348248069922
c	-0.97503317460910	-0.66889887114274	2.86952733699751
h	-0.26077731742123	0.14490148491902	2.92602233734140
h	-2.92041823247963	-2.41081497358369	0.65321556942199
h	-3.57079602479157	-3.54864271127651	2.72265403349029
h	-2.57956663003601	-2.87246409842973	4.89094672235129
c	-2.03133824243011	-0.77052860672814	-1.25680623239585
h	-3.07557764505724	-0.58415039107839	-1.00179150810976
h	-1.76928419199807	-0.20861557453371	-2.15096882942085
c	0.79795837575528	-0.70326209598350	-0.14213229221304
h	1.23229060600176	-0.65975133578442	0.85920222042513
h	0.76044035460067	-1.76314580225749	-0.42008592580291
c	1.70639040243989	0.07606146671675	-1.14171684637455
h	3.91267546202254	0.39260826792971	0.73186551554247
h	0.86501239357514	-0.94901363734353	-2.83872603185381
c	1.11935451018728	0.08141088513036	-2.56640747492306
h	0.18002580692595	0.64468218997018	-2.53487463070788
h	2.88298678612297	0.08866697473832	-3.86844197650360
c	2.00486442553384	0.69893892286924	-3.65354056393344
h	1.43927946868160	0.78332590802191	-4.58357576157625
h	2.33799443656331	1.70021335233500	-3.37336505090096
c	3.12138059048286	-0.54020657835814	-1.10779539752716
h	3.05850573243439	-1.55017180107101	-1.52541378542487
h	3.73466049626871	0.03599846074690	-1.80409216288642
c	3.82763857003398	-0.59025282286701	0.25351825730188
h	3.34748408139495	-1.26870023265650	0.96236377086101
h	4.84934427440853	-0.94796791429493	0.11607255323609
o	1.75611369154506	1.46643225173655	-0.71564236827754
h	2.55763190693881	1.63082725863422	-0.20315139743681
o	-3.12222879855597	5.36857211137188	0.00996027879707
h	-2.90821664193234	4.40949371630112	-0.02564876011096
h	-3.61687049789453	5.63706461144603	0.81288012349669
h	-3.50701934226472	5.72262792377106	-0.81952329242329

Intermediate III, Scheme 9

p	-0.97187584629459	0.14114500884632	0.31124760843851
s	-0.65774288717727	2.16786532169227	0.84669995737639
h	1.17904002056435	2.11054045967369	-0.25569987290482
h	-1.27095919332299	-1.00686919217178	5.09039527698525
h	-2.12398743839134	-1.01654641456531	-1.46352384475357
c	-1.56031371211802	-0.81556832718063	1.71156415719483
c	-2.38296836629521	-1.93419693138993	1.48629296469530
c	-2.78915394004743	-2.71085549388724	2.56644511831149
c	-2.38494759297654	-2.38011182123865	3.85960570739068
c	-1.57149757574402	-1.26817841325911	4.08311631665531
c	-1.15526787978530	-0.48186201465442	3.01527812388882
h	-0.54715734983304	0.39461923432674	3.20729580140637
h	-2.71618618640143	-2.20595337303185	0.49211827633671
h	-3.42748402994524	-3.56940247024519	2.39705800029142
h	-2.71070593477694	-2.98608171398689	4.69674038924016
c	-2.13602484713363	0.00457946768887	-1.07614766331362
h	-3.13598261693794	0.24126668190587	-0.70846808029633
h	-1.87604753929916	0.69533607526521	-1.87548068330467
c	0.73593545135547	-0.47608847615336	-0.09075075316014
h	1.23363632195266	-0.51627884191850	0.88226054911000
h	0.59569346240802	-1.52209504508591	-0.38724539499637
c	1.66409359078516	0.16510639964727	-1.14340192928986
h	3.97613805151731	0.29856532095323	0.67190906021881
h	0.61547224150727	-0.60077535843515	-2.80765949291655
c	1.03263699781960	0.37138575054539	-2.51950117018374
h	0.17982396923671	1.04812465624176	-2.40972352688431
h	2.71121009051974	0.17137149939212	-3.91629920347828
c	1.94448067765176	0.89371416003565	-3.63493116885881
h	1.34296438756936	1.08471416834156	-4.52492248541260
h	2.43015281422227	1.83228077741328	-3.36024421212397
c	3.04092686753822	-0.50266521352555	-1.17155424164112
h	2.88530489893841	-1.48861220437279	-1.62299969383691
h	3.66092416992636	0.04966487672131	-1.88069068313571
c	3.78709628134795	-0.65520404816529	0.16079224572481
h	3.28613400709918	-1.32203099366242	0.86455019035096
h	4.77267526094393	-1.08019819014964	-0.03444104038016
o	1.97609350762518	1.65411531490105	-0.65478048952161
h	2.73934520182571	1.70384624445071	-0.04978057362280
o	-3.26950222855062	3.30209511569437	-0.31578307588309
h	-1.82232990816574	2.70740747873661	0.26594058932628
h	-3.96266190501920	3.53977623787404	0.31536059772747
h	-3.31698129413902	3.96205428673216	-1.02064165077052

Interemediate IV, Scheme 9

p	-1.28491264109811	0.30784157663598	-0.03662883275639
s	-1.13160274828354	2.44566989190822	-0.25416759142659
h	-1.25315640859197	2.69763064149896	1.06989947519449
h	-1.17229199878652	-0.03017327124218	4.86803670794562
h	-2.41350143989165	-1.33805423525790	-1.38355158709524
c	-1.95754871021855	-0.12935730265905	1.56115352409100
c	-3.24125521035734	-0.69747113546929	1.64618998367599
c	-3.75926171922179	-1.02881976567593	2.89439341015262
c	-3.01676463367538	-0.78861836882169	4.04919736206347
c	-1.74426517316172	-0.21612611741300	3.96719283307939
c	-1.20938910806321	0.11418520749481	2.72997789159672
h	-0.22142717057793	0.56135386185054	2.69071115882581
h	-3.83798461955441	-0.89046732731930	0.76405185850690
h	-4.74511911912894	-1.47236305319812	2.96177838805746
h	-3.42866938907584	-1.04588924831251	5.01789189995487
c	-2.35105577952895	-0.24715959739345	-1.39190975628783
h	-3.34573598317146	0.18226687695524	-1.25950472622585
h	-1.95448677751547	0.08580641421751	-2.35075584203585
c	0.43140165112362	-0.39368347506518	-0.16046575428546
h	0.83235509057647	-0.34119477712077	0.85374693299811
h	0.28440881246602	-1.46141605529508	-0.36324240914890
c	1.47738888049893	0.11429357805880	-1.17512644704026
h	3.63622114300119	0.25057744744571	0.82103545650978
h	0.55327185434772	-0.72475384520273	-2.87309321515592
c	0.97301370763546	0.25402742871923	-2.61087550601212
h	0.13357204104417	0.95864200169378	-2.62424556105150
h	2.75562691211406	-0.08325971806072	-3.84095641759532
c	1.99366122133888	0.67825047974046	-3.67292388563982
h	1.47601079109692	0.82847307525793	-4.62141089236605
h	2.48575695715521	1.61817841491183	-3.41573814706987
c	2.80962052339983	-0.62528857034413	-1.04038815759384
h	2.63391113941348	-1.63043171715009	-1.43931207642250
h	3.51738201764654	-0.15643993124583	-1.72699949602857
c	3.43037619189465	-0.72490217681529	0.35954849512996
h	2.83234546146175	-1.31242485617510	1.05845773201873
h	4.40069688838441	-1.21709445344480	0.27976203645177
o	1.83859245156318	1.61612013378925	-0.75700091047729
h	2.53203339282553	1.67672775541785	-0.07403730997421
h	1.04078150091482	2.16534421308601	-0.52069062456329

Intermediate V, Scheme 9

p	-1.17773672508518	0.52740971533135	0.13155045659211
s	-0.72550033859022	2.57453726549627	0.35436922297316
h	-0.41094518433926	2.47281035767972	1.66574306923387
h	-0.94089114122793	-0.99764014671917	4.80256081893354
h	-2.62464211618420	-0.55068246223418	-1.47896768653787
c	-1.85260576051373	-0.19782818837113	1.62679456571090
c	-3.20530207297740	-0.57450149801943	1.67346485470060
c	-3.72415508365997	-1.11078835816307	2.84859120932380
c	-2.91136151079836	-1.26309059110697	3.97016000414307
c	-1.56784237606646	-0.88231827097765	3.92683609817881
c	-1.03307729370891	-0.35211457496278	2.76028598281761
h	0.01041013303486	-0.05375292577150	2.75241748634231
h	-3.85800460452298	-0.45975128734335	0.81766717648698
h	-4.76589449880495	-1.40514736515548	2.88551953014838
h	-3.32399852590712	-1.67633172228891	4.88286564906270
c	-2.37388852093849	0.48257475914650	-1.23112246055314
h	-3.27371087834533	1.01674933037460	-0.92102244851111
h	-1.96924798612232	0.98114374571215	-2.11242203660542
c	0.31669503705148	-0.52608174393671	-0.30948931067520
h	0.76496718704800	-0.78435377469114	0.65038851912485
h	-0.15189172826618	-1.43389284997729	-0.70423844600120
c	1.38271503210902	-0.01315592670731	-1.28024671690192
h	2.49498872735449	1.45685098289313	-3.58746265296001
h	3.21014039175866	1.04503661593524	-1.48332518647272
c	2.41209772778351	0.95492677503922	-0.74462250801702
h	1.89952272760800	1.92617966231065	-0.74950445644854
h	3.53527100599152	-0.25468988906693	0.72525353344278
c	3.00144834336899	0.69875883044812	0.64703209743047
h	3.73930243946022	1.47175671682381	0.86609174501679
h	2.25558834636190	0.73552162148850	1.44345706866809
c	0.91332414095222	0.22227420509701	-2.70150665185996
h	0.27282909050547	-0.61176010749030	-3.01108835399240
h	0.24466606507119	1.09101105867457	-2.62215753198401
c	1.97011388130121	0.51927697722313	-3.77260539535470
h	2.70667625531017	-0.28125997009379	-3.85541076568067
h	1.47433532693520	0.61219327219531	-4.73979559061210
o	2.37239063875288	-1.43824500981905	-1.49434758930807
h	1.96688114989846	-2.23295332885541	-1.88094680341340
h	2.94633269840139	-1.68867190011755	-0.75076649644131

Transition state VI, Scheme 9

p	-0.92261268870725	-0.09207439588679	-0.10841515248519
s	-0.32670647848915	2.03467252395144	-0.27597396309764
h	-0.93300525032161	2.29145727400986	-1.46060902732551
h	-0.63721849659849	-0.96872002050557	4.71196209018196
h	-2.11698747531868	-1.57641574433823	-1.51658119465261
c	-1.58160338413204	-0.51685123468148	1.47755562812251
c	-2.96878751225735	-0.74352790525871	1.60775109350193
c	-3.49211616955180	-1.05814390787970	2.85446656632465
c	-2.65356126704555	-1.13730033324210	3.96774631251297
c	-1.28087584796082	-0.90404372069367	3.84330224000206
c	-0.73830181494925	-0.59065387189694	2.60628790296714
h	0.32637261248992	-0.40788724810293	2.53048685206218
h	-3.63651626467899	-0.68982903087100	0.75748026060838
h	-4.55436809985855	-1.24291361708605	2.95731073162311
h	-3.07043446351311	-1.38162870994909	4.93784621656793
c	-2.03129887665637	-0.48583347252379	-1.48046554407167
h	-3.00526018363580	-0.01788563247495	-1.32936854979391
h	-1.60317527712759	-0.13613504991175	-2.42032124797946
c	0.81854636579829	-0.56043650500512	-0.42629111024201
h	1.27871211060757	-0.89507361692545	0.50329989026174
h	0.83977838954706	-1.39311544785673	-1.13857968463675
c	1.48090961982747	0.71861668952921	-0.97312599006459
h	1.27113066718718	2.98801058199412	-2.78426827488062
h	3.48179536600840	0.67171053670883	-0.56646275442325
c	2.63838670604520	1.29952719760180	-0.22030974148527
h	2.85581976995032	2.29553559820697	-0.61029552156753
h	2.58407427408167	0.31036324531080	1.74416460839978
c	2.62228967598351	1.31098552549069	1.30839548978135
h	3.54329848234888	1.77140781794302	1.66808088881011
h	1.79578063673383	1.90854673974139	1.70409829794775
c	1.49852093210725	0.83474532334889	-2.47157855799924
h	2.22603786250340	0.05187876784619	-2.75653305672645
h	0.55356208857292	0.48001474906019	-2.89296323819643
c	1.91984656935319	2.16419836044942	-3.09845667973868
h	2.94905020458758	2.43144846124953	-2.85856942960855
h	1.85011738711553	2.08924490577446	-4.18434452528986
o	-0.34764569826414	-3.24084822020769	-1.74821162037452
h	-0.33944418061049	-3.57581078357430	-2.65424383281626
h	-0.29411029117223	-4.03723582934479	-1.20426637221963

Intermediate VII, Scheme 9

p	-0.82380205131429	-0.41627096837829	-0.29898598743081
s	-0.43095773071820	1.82798900951851	-0.35647508286588
h	-1.01236398434927	2.11485505874522	-1.54765685192577
h	-0.32611638564110	-1.46917890085492	4.46793282361840
h	-2.16193518724480	-1.81570296452976	-1.67088847636033
c	-1.40616121482872	-0.88942783881454	1.29842942916551
c	-2.79111410331471	-1.09400136447737	1.48238793827750
c	-3.26521761120778	-1.43305920618122	2.74191105547215
c	-2.37812825216218	-1.56453097423853	3.81312202356042
c	-1.00691812640820	-1.36025395809020	3.63255510846693
c	-0.51281686544814	-1.01713154322341	2.38252687699192
h	0.55140307365489	-0.86174997085682	2.25974497177097
h	-3.49329096725845	-1.00216338149459	0.66299433841283
h	-4.32580924319866	-1.59674643823035	2.88878347471752
h	-2.75650712534592	-1.82857863110818	4.79385532886166
c	-1.97034958073431	-0.74122315939683	-1.66100823326047
h	-2.90018108796378	-0.18855541553224	-1.51337201131072
h	-1.51407507749903	-0.44252991291104	-2.60585992031946
c	0.96601032506739	-0.47068813056801	-0.68730392224964
h	1.51524415838059	-0.84775940966108	0.17499614859701
h	1.15256960209989	-1.15138469774831	-1.51816698825324
c	1.30300814690862	1.01404104955156	-1.03345527855820
h	0.74720532186970	3.34541771426779	-2.68541578507567
h	3.35181532322731	1.17269505359906	-0.63343488723477
c	2.44039566573053	1.63729269453862	-0.23091147075968
h	2.50752811141130	2.69460359578758	-0.49540335265539
h	2.52400345587920	0.45066595233658	1.61968576842564
c	2.42206124828339	1.48781798250727	1.29136253112303
h	3.27011296441815	2.02898305881580	1.71334780995981
h	1.52220025339199	1.91770581418797	1.74321325152778
c	1.39135712753325	1.24835500822330	-2.54307179717147
h	2.26276618928317	0.65722675303619	-2.85870636357250
h	0.53473130705135	0.78002994527425	-3.04098658029241
c	1.55862267045478	2.69110371851250	-3.02343416826416
h	2.50155281115576	3.13055930296526	-2.69799338128325
h	1.55086949491938	2.71222880855753	-4.11422047422480
o	-0.37289222398525	-3.18306291530052	-0.60557128154655
h	-0.05691990734852	-3.78397839830467	-1.29358719748307
h	-0.56790052474929	-3.75359234052414	0.14906061314904

Compound 54, Scheme 9

p	-1.26473039157697	0.77631697062218	-0.36294824273399
o	-0.99811147176391	2.14321131576712	0.21281927885326
h	0.58869637299479	2.71776779753554	-1.49284166421932
h	-3.55000211078237	-0.03657745050341	3.91987165104605
h	-2.53436556659444	-0.19156036932379	-2.22203387365144
c	-2.19558474825822	-0.28259366684823	0.80965421887216
c	-2.55229450824537	-1.60676913337736	0.52389077401829
c	-3.26259303220411	-2.35913900715697	1.45617190340424
c	-3.62253990162242	-1.79409744233946	2.68009736030472
c	-3.27057199983797	-0.47736895260012	2.96946823732791
c	-2.55857525294509	0.27786350555595	2.03825583973721
h	-2.27707621805753	1.30349998583410	2.24857014880474
h	-2.27910553545932	-2.06056391536096	-0.42352607689419
h	-3.53571812693650	-3.38348965631213	1.22868592343571
h	-4.17635713708629	-2.38051557510316	3.40470812904488
c	-2.28140064083545	0.81485484082596	-1.88120259713679
h	-3.20014840462526	1.36227172551674	-1.66270740821318
h	-1.72426479287047	1.33311279914318	-2.66302970020760
c	0.25553785294106	-0.20502200691549	-0.79688966097584
h	0.39689933273394	-0.94324213417177	-0.00178383899725
h	0.02340282714073	-0.76413658528248	-1.70839084245051
c	1.58258960542050	0.56825607038745	-1.00418742588542
h	1.55750032261700	-0.32707550338391	1.78476132252517
h	2.61355014978610	-1.31997580897013	-0.99609114937106
c	2.59574407481439	-0.42590529744243	-1.62985395648876
h	2.19444720409421	-0.75364123071515	-2.59540705996816
h	4.51822988791072	0.30140983546033	-0.87445536612508
c	4.02859365264671	0.07986638305882	-1.82554647865805
h	4.62514571168478	-0.68439000049641	-2.33108222427803
h	4.05696869508178	0.98494477297955	-2.43558173637571
c	2.10126927004066	1.20702032290587	0.30656999778195
h	2.99108875778029	1.79259385405931	0.06333858262292
h	1.34946113404654	1.91522848251264	0.66006810111977
c	2.43607621623255	0.22342400548200	1.43734745004121
h	3.19746013299826	-0.50639743873848	1.14836512537990
h	2.82473077064407	0.77543957080534	2.29731108510591
s	1.34604786809252	1.92537893658969	-2.28239582679567

Transition state VIII, Scheme 9

p	-1.36355181941266	0.31130168135002	-0.09033884486299
s	-1.49441835989624	2.38814753261136	-0.46193726022319
h	-1.34900675711813	2.73053516373493	0.83841410338116
h	-1.21104764764745	0.32031341019496	4.82150485798302
h	-2.25043774962121	-1.54548675299387	-1.36136757152033
c	-1.97290236598073	-0.12154439510935	1.53853190127946
c	-3.15770570031217	-0.86421706291844	1.67361607514042
c	-3.62282335243663	-1.17990487057584	2.94700188410370
c	-2.92369468599995	-0.75509688442572	4.07522687952482
c	-1.74864684192004	-0.01111505862429	3.94151565513060
c	-1.26724115477461	0.30744948823683	2.67859596843428
h	-0.35756537712254	0.89430526975704	2.59732051530121
h	-3.72050457091287	-1.19978925353232	0.81191528594563
h	-4.53451047301062	-1.75478593386721	3.05437233202352
h	-3.29499950058215	-1.00050180743295	5.06315664500264
c	-2.34415262486150	-0.45829374369043	-1.40747407026499
h	-3.38958778021598	-0.17376701815960	-1.27525710185287
h	-2.01828827772167	-0.10166683068158	-2.38518732483173
c	0.39329008669254	-0.31684900085050	-0.13401152720467
h	0.85199084929077	-0.01857027637555	0.81172464004711
h	0.29829583851651	-1.41192770678407	-0.04758643751056
c	1.38923782600872	-0.07653918831010	-1.22839852938226
h	3.28243344196852	-0.28851574423994	0.99618620434382
h	0.36586978231249	-0.45484219940599	-2.93379029339119
c	0.96892651113897	0.40012530443932	-2.56517030664292
h	0.24055084759552	1.20868517909298	-2.45068464873590
h	2.66303183984520	-0.10595489745467	-3.86129378530544
c	2.03511415546238	0.74803171741790	-3.60438482561219
h	1.54527091998497	1.07834362606466	-4.52096772838227
h	2.67488914508766	1.56264161792580	-3.25819749251665
c	2.71335777740262	-0.71315602107567	-1.06840604026427
h	2.61688600722656	-1.60390843039697	-1.72522377712011
h	3.45438563100103	-0.11133008123925	-1.60323524580857
c	3.20170809405309	-1.13978830750591	0.31571187026981
h	2.56303811557488	-1.89508903155355	0.77750610958531
h	4.19613028277393	-1.57677208144856	0.22057228049190
o	2.01294880253175	1.96685822075172	-0.33811346420427
h	2.94357350152631	2.17895274232548	-0.17862946163997
h	1.61015558355265	2.78372162474941	-0.66321747071114

Intermediate IX, Scheme 9

p	-1.24502533435975	-0.19093464622279	-0.17850723214765
s	-2.09114278120263	1.31683941290089	-1.37912972819640
h	-2.19444704205004	2.20627700618310	-0.36470219901696
h	-1.57269029364684	1.65810166103532	4.36350520244979
h	-1.21690728478807	-2.58246071716521	-0.51092224009937
c	-1.83530129432272	-0.16162950385722	1.50865276463987
c	-2.76662390393127	-1.12723811203599	1.92970368301400
c	-3.25326097950453	-1.07681907502722	3.23200516676573
c	-2.82437598908701	-0.07647600935363	4.10321003795069
c	-1.90184639060905	0.88407792545542	3.68107772685886
c	-1.40157118148375	0.84962532577128	2.38578672971887
h	-0.68401059911629	1.60311004073178	2.07713527435560
h	-3.11607855586725	-1.90966918460070	1.26794580660300
h	-3.96838046191619	-1.81996642359170	3.56305796486143
h	-3.20931446048816	-0.04350010144367	5.11559846914993
c	-1.64517885227639	-1.73200698201314	-1.04416036190508
h	-2.73032039306737	-1.83901329437620	-1.09424691399073
h	-1.25775846232392	-1.70129575995934	-2.06478788270666
c	0.63139638466477	0.00455386027163	-0.05745493097708
h	0.75223912769231	0.97791970924128	0.44013686659381
h	0.92478732478255	-0.80220201966518	0.61824883632114
c	1.43826504029782	0.00055178763907	-1.29828766593926
h	2.65932294558724	-2.26009042279629	0.12675955851882
h	0.50311566924751	1.65411292965472	-2.14894172986412
c	1.52659289303368	1.24116657747926	-2.06567239362535
h	1.94884244881474	1.95879833375287	-1.32582027155322
h	1.84476851941086	0.60451636944955	-4.13075502092698
c	2.28062516113603	1.27886207914077	-3.38918595330255
h	2.23533044557853	2.28809999555105	-3.79889539241666
h	3.33653019981957	1.02441667903705	-3.27280489003600
c	2.19605962129561	-1.18289364255187	-1.71405385544975
h	1.85895804019342	-1.38966806085469	-2.75045507260686
h	3.21670910856214	-0.81568456504998	-1.92924317048081
c	2.22452768857949	-2.44493880370561	-0.85834770758250
h	1.23498366995441	-2.88769456012631	-0.73020646681312
h	2.84787810643847	-3.19431362071295	-1.34714804192987
o	1.59455961902154	2.90850782121158	0.84422128913196
h	2.31842285131256	2.94778192606101	1.48319451747930
h	1.34031939461792	3.83117606454206	0.71348922715420

Transition state X, Scheme 9

p	-1.00769375866882	-0.01848237043175	0.00175894376820
s	-0.47274095930833	1.83120380771000	-0.84053036260459
h	-0.28299846113098	3.08948602909223	0.52670496938761
h	-3.90192830866014	-0.44923845737323	3.96608681817252
h	-2.65729513778768	-1.67247214667119	-0.60489692292869
c	-1.36554433990713	0.05228746113665	1.76250252256735
c	-0.33634830064301	0.36685597173161	2.66894825871097
c	-0.60314935750697	0.38687592115174	4.03250773954771
c	-1.88757609592068	0.09326459298654	4.49885823446729
c	-2.90839125860305	-0.21669277489540	3.60245793222714
c	-2.65649015758989	-0.23851613885928	2.23257227092518
h	-3.46270311534247	-0.48932516345047	1.55493253099445
h	0.66944829136568	0.58545804161730	2.32505028747966
h	0.19070639274356	0.61568297429329	4.73341951410331
h	-2.08826988621183	0.09976165761802	5.56371641098500
c	-2.42142430050586	-0.65902075333311	-0.93767481767806
h	-3.28836463042446	-0.01163434378763	-0.79927797994274
h	-2.16569036477533	-0.67115983699147	-1.99813491967298
c	0.49831332968234	-1.12963290053462	-0.28155799351241
h	1.08186616189035	-1.05381465446664	0.63725692946852
h	0.09192824841789	-2.14210197189710	-0.34981090140589
c	1.28796457176178	-0.74945437524158	-1.49643619937733
h	2.86433756234277	-2.52015308040814	-2.95818380593660
h	0.93689732697512	1.09614165334973	-1.27652567609194
c	2.20210772815094	0.27424016645432	-1.37291531654092
h	2.49535403234294	0.54323668018034	-0.35707837038464
h	2.72129487824070	0.65262968190780	-3.45665726207705
c	3.10648920874102	0.80804446590948	-2.44965898537022
h	3.28800773178880	1.87570829996417	-2.30797334210081
h	4.08155349349122	0.31463177547644	-2.38111032628102
c	0.96194636616502	-1.43605983186995	-2.78855526770937
h	-0.10034601899368	-1.69499758597299	-2.82537020222961
h	1.16948764741058	-0.78218715334365	-3.63655732395080
c	1.79538562586104	-2.73681381198882	-2.93601375847876
h	1.60264456418352	-3.43864588126555	-2.12163945813935
h	1.52650328952590	-3.22642924328731	-3.87297845125846
o	-0.16133090803914	3.88803629560614	1.25288018507014
h	0.13518616706249	4.73359130181238	0.86662621582038
h	-0.93913725812428	4.02969569807162	1.82325787997693

Intermediate XI, Scheme 9

p	-1.00134999252283	-0.13763403596659	0.04132920004299
s	-0.74240122517656	1.82441112766059	-0.84202540689462
h	-0.44579595704367	3.24183045122028	0.49051019820069
h	-3.88577880480325	-0.56019573455010	4.01010926538925
h	-2.53604729313175	-1.89776465664139	-0.52077060722139
c	-1.36214343901008	-0.03608232883235	1.79497525310926
c	-0.33734919016775	0.30949113963080	2.69710546493549
c	-0.60277204810125	0.33977040755824	4.06001296866286
c	-1.88069304259764	0.02710846589262	4.53192632637514
c	-2.89813267871555	-0.31144766963064	3.64125569282631
c	-2.64861112668037	-0.34300239451895	2.27212029519069
h	-3.45075659573499	-0.61921510200651	1.59960516073855
h	0.66421356423909	0.54006863717583	2.35045678867242
h	0.18781072887357	0.59041337256588	4.75703879918706
h	-2.07918312867666	0.04049059425577	5.59718276414138
c	-2.38559075622894	-0.87322154052156	-0.87069995774151
h	-3.29804528126459	-0.29476748573670	-0.71995203894584
h	-2.14795857813466	-0.88524644634305	-1.93468433599677
c	0.61083868391234	-1.02390020286585	-0.28140618052544
h	1.19444003174686	-0.89475216623159	0.63158954185255
h	0.31099035313065	-2.07759439399745	-0.32874415901595
c	1.38391526419829	-0.60237418096166	-1.50982184310512
h	2.51726633668724	-2.68690110792514	-3.03506610701820
h	0.52844967828833	1.47874212490809	-1.27586581032623
c	2.47635973978226	0.17504560833066	-1.33983735976105
h	2.73699664601155	0.46011898463923	-0.31993345916809
h	3.14271425535957	0.40380149291632	-3.39902622424714
c	3.45160764810183	0.62780287168692	-2.37932038464361
h	3.63869025652456	1.70305012491860	-2.29842363486729
h	4.41803650175305	0.14107633055753	-2.20595465128395
c	0.94220129465509	-1.18762735437276	-2.83425163992519
h	-0.15133388801082	-1.16552846485383	-2.91040562923568
h	1.30778345542235	-0.56928361875271	-3.65490183950142
c	1.42691247944395	-2.63752725256185	-3.03678941029887
h	1.05962861021713	-3.31060690685226	-2.25704615292284
h	1.07223330279297	-3.02057505184897	-3.99514251079168
o	-0.29515375603372	4.05901173258293	1.14122970416457
h	0.18190694497906	4.80985120267394	0.73754539212633
h	-1.10389899408455	4.36316342679767	1.59607652782228

Interemediate I, Scheme 10

p	-1.49795706509352	0.56959915550494	0.05165516198221
s	-1.07671298632947	2.51931931247805	0.03248774868799
h	0.89868821674388	1.86149951335797	-0.38884315283471
h	-2.71610731663103	1.01412572166229	4.82552209512341
h	-2.93635542330419	-0.98156588661105	-1.19136619947000
c	-2.17509838046800	-0.04077153087239	1.64254881693197
c	-2.64302163064089	-1.35672004673016	1.76581256670708
c	-3.13187567919965	-1.81253332407655	2.98672036648567
c	-3.15962471398228	-0.95902342934902	4.08970470725516
c	-2.69653689000223	0.34891146688553	3.97013762538158
c	-2.20427823263519	0.81019221602768	2.75037441306266
h	-1.84176903315620	1.82665500912157	2.65100660374385
h	-2.63010988271352	-2.03482738674536	0.91938286542532
h	-3.49186046266076	-2.83101732357564	3.07636521651620
h	-3.54117549139991	-1.31541572196751	5.03968447893412
c	-2.73945051306185	0.09219487591202	-1.20887254041688
h	-3.66138905098022	0.63308687943930	-0.99063041085512
h	-2.38337953358683	0.38475856144035	-2.19597277149000
c	0.00664105253060	-0.49054804911609	-0.21051102489521
h	0.43365165578048	-0.61913297592553	0.78531793703808
h	-0.33697870333785	-1.47483436434270	-0.54396770546826
c	1.12142944327103	-0.00304769323054	-1.16511922204767
h	3.02377536470082	-0.99699094524173	0.79510376723031
h	0.05253541704525	-0.51479924760473	-2.94343325168077
c	0.58975553330063	0.36311461231050	-2.56524871621134
h	-0.14685105142706	1.16311715309496	-2.45418693211119
h	2.33885796799231	0.00761157043742	-3.84285872151304
c	1.63998258038234	0.80631194724451	-3.58852086990497
h	1.14202749155646	1.11424243081363	-4.51103087928830
h	2.21804154077034	1.65758865055985	-3.22507844326353
c	2.23686100892541	-1.05838870279836	-1.25593609575190
h	1.86349880908361	-1.83025217647364	-1.93861062808412
h	3.08944015843279	-0.59905349016422	-1.75640843293989
c	2.70926026047870	-1.72180363892608	0.04268902878216
h	1.94159326874290	-2.36036777101180	0.48726070785023
h	3.57193098959087	-2.35567106573371	-0.17443105303732
o	1.67362524921192	1.24826979401521	-0.56250931758582
cl	3.46826348710495	1.44020041822178	2.10695892273252
al	3.29669747152946	2.04522127116356	0.06563699320131
cl	4.88465863115242	1.34938478640461	-1.17866608812068
cl	2.80931644228346	4.10135942440107	-0.19216756610116

Transition state II, Scheme 10

p	-1.80702359775660	-0.23456199639703	-0.32275343985137
s	-2.61231220446254	1.08086393914667	-1.54254222874050
h	2.35030673329321	3.43774476622358	0.35799735683016
h	-4.38425708006954	1.72255194800481	3.40938208934009
h	-1.58299727796585	-2.67253848043939	-0.15508553559609
c	-2.36626302473307	-0.14720895710408	1.41871530434142
c	-1.90099880167339	-1.06233876882872	2.37297949084206
c	-2.33096081478601	-0.97004076395423	3.69314161607357
c	-3.22594299883471	0.03202685087538	4.06718797631129
c	-3.68972392116667	0.94234733207797	3.12046575376121
c	-3.26107374543613	0.85731145761140	1.79740629828929
h	-3.61639306985757	1.56290739729557	1.05579144345147
h	-1.19878915873789	-1.84383622391301	2.10471345880489
h	-1.96302845642630	-1.67582097735471	4.42847929595812
h	-3.55694588403535	0.10365368081376	5.09684375604042
c	-2.04994344955757	-1.97130598794908	-0.84823600022631
h	-3.12456001776483	-2.15612473088694	-0.88249655753140
h	-1.63969894946194	-2.10474312200081	-1.84996158448338
c	0.08535379899795	-0.03759452348399	-0.09925078839985
h	0.15891249918168	0.90667397194356	0.44610653488065
h	0.41322560875939	-0.85047906643794	0.54571884731737
c	0.85455705728709	0.04765069680470	-1.34374209343207
h	2.12654451103845	-2.24361549276932	-0.09447645293442
h	-0.06122826298164	1.84404870315539	-1.86071934626829
c	0.90026943637543	1.32107964832911	-1.99742954969820
h	1.53140160945637	1.89096412505010	-1.20511599832459
h	0.94672509143858	0.89689943236758	-4.13293460638369
c	1.50346120371793	1.47067687128687	-3.38566914946670
h	1.47128314779255	2.51953120540439	-3.68378292482270
h	2.54850545570585	1.15612966457406	-3.40957619555507
c	1.63732964659905	-1.09350009775081	-1.87065402501671
h	1.30170997210392	-1.25189534005376	-2.90815320063211
h	2.65766744769883	-0.69290424043700	-1.99985451407580
c	1.69310361575006	-2.40048970804152	-1.08420420441901
h	0.71168766775753	-2.86704222929869	-0.97602029070054
h	2.33396564723038	-3.10883714849333	-1.61228290805826
o	2.38136780545462	2.49938493673168	0.15430587008204
cl	3.07709900691841	-0.12727228122062	1.81369025265998
al	3.82231976433454	1.58285728146083	0.68504787378437
cl	4.74231014757284	0.79018180583017	-1.12545546286470
cl	5.12303384124307	2.84666442182749	1.82242383871337

Intermediate III, Scheme 10

p	-1.16298948205708	0.62268535173686	-0.17827390090525
s	-1.73129203089691	2.41651882608736	-0.78062294127551
h	3.47025981595927	-2.42576005865730	-1.28530896564917
h	-3.87456614483260	1.32029048006625	3.91523270672451
h	-1.33602888482100	-1.69519889064046	-0.99173808226497
c	-1.89507842212022	0.10391519974515	1.43343746608064
c	-1.65167753781824	-1.17141447447313	1.96151731520555
c	-2.20646387139513	-1.54610169366516	3.18254497339499
c	-3.00735677133535	-0.64897450756517	3.88916899191038
c	-3.25165012861025	0.62022507460652	3.36987762250067
c	-2.69875188769046	0.99672383383068	2.14651811360200
h	-2.88730416406643	1.97993547720630	1.73049350669882
h	-1.03223885434621	-1.88375565212998	1.42667762183865
h	-2.01442736169017	-2.53599693966840	3.58112024749597
h	-3.43906691274880	-0.94080113517430	4.83999987531323
c	-1.62938720149216	-0.70742860335106	-1.35284153222689
h	-2.71141658679549	-0.67416953238256	-1.48493246990958
h	-1.14975452872818	-0.50502242618725	-2.31107007424016
c	0.67503635602542	0.45017532784991	0.10544764860819
h	0.91394168083221	1.28270390801050	0.77113119972687
h	0.83042462610208	-0.47251720022220	0.67194050333168
c	1.52282047274827	0.47168896747930	-1.14939744040206
h	4.06478545899312	-0.77351778716069	-1.05497774542272
h	1.47471880318201	2.54638079696222	-1.23515125960865
c	1.85096625647364	1.64380381708990	-1.71076518569579
h	3.01638199262562	-1.51112688383310	0.15643671236834
h	2.08849992233243	2.43509204099253	-3.68850497822899
c	2.67062444123262	1.87948497228283	-2.94476717653902
h	3.54556347150371	2.49720520822681	-2.71227365215303
h	3.02739197041571	0.96083909350278	-3.41216342775723
c	1.97375611003338	-0.88040173955677	-1.66634501820559
h	1.15221124060683	-1.59924479212210	-1.57026768308045
h	2.19983175124793	-0.82561319927661	-2.73347380816031
c	3.20223640113045	-1.43062285960949	-0.91866916307508

Intermediate IV, Scheme 10

p	-1.42394353546860	-0.14074225757503	-0.36248538657138
s	-2.02588876835122	1.31999293269953	-1.54135873150972
h	2.40038656485301	-2.60098138235629	-2.41552082445780
h	-4.56760090072357	1.47255057393722	3.10206792781843
h	-1.32304288777624	-2.59653716032428	-0.40224822694828
c	-2.24803614844701	-0.21156418452200	1.27851065052746
c	-1.92901700909367	-1.21418409894279	2.20458631777563
c	-2.55848491834591	-1.24537882330282	3.44588086495487
c	-3.50997683316983	-0.27855016156991	3.77023941272797
c	-3.82887077018515	0.71955744613324	2.85250221352474
c	-3.19922870062503	0.75647607328892	1.60945956252156
h	-3.44010366368017	1.52993971711070	0.88920440885917
h	-1.18987230314576	-1.97318067169582	1.97222077621831
h	-2.30524280847246	-2.02195205416037	4.15842508749704
h	-3.99925356341329	-0.30424873141191	4.73726937263397
c	-1.67719258542039	-1.81278218413973	-1.07467174213740
h	-2.74767449901468	-1.93741963545280	-1.24380861240424
h	-1.16193917578322	-1.88477636932288	-2.03237256508530
c	0.39642253145292	-0.03567244347600	0.11373655743475
h	0.41382052305078	0.68848292521154	0.92825511369258
h	0.69725164679912	-1.00019032779136	0.52446476453898
c	1.27698925482126	0.41075434785161	-1.01787718126067
h	2.92474240751412	-1.92600936666346	-0.87451986055967
h	1.09458247130442	2.39496819335050	-0.39546062224802
c	1.56130992428992	1.74422393363147	-1.13359816406703
h	1.26867011551141	-2.51693299350993	-1.07146400174329
h	1.19441420898136	2.70463028362187	-2.98691943587147
c	2.06445065335754	2.45263587627122	-2.37035962704548
h	2.56602619976219	3.38506902040702	-2.10952571559380
h	2.74534250244162	1.85385587238876	-2.97371266197093
c	1.66996247982554	-0.59757449559330	-2.07095912275408
h	0.80486385946218	-0.68566711623172	-2.74424231146003
h	2.47358436616844	-0.18645262231029	-2.68122285780002
c	2.08434985997313	-1.98797864137382	-1.56871100110809
al	3.54959704465023	1.42290655948104	0.17667589367418
cl	4.94909132827613	0.65689878410410	-1.23087995729938
cl	3.76119892307494	3.47913623631275	0.66946256497542
cl	3.23831220554592	0.16669694592507	1.86895712052099

Transition state V, Scheme 10

p	-1.03760617642246	-0.40960733035153	-0.27817099276366
s	-1.26809982359957	1.05310956129473	-1.65382029854352
h	2.94453470266134	-2.27504426190393	-2.40862618600160
h	-4.73368060017671	1.56964984011753	2.36790881905869
h	-1.03119026656211	-2.84111160959510	-0.22256400950016
c	-2.19311768571615	-0.31363549776649	1.13076469402391
c	-2.10018763422954	-1.26203933216879	2.15978665551745
c	-2.95477865671393	-1.18566642973015	3.25541043820174
c	-3.90573816965852	-0.16833075027570	3.32984718524835
c	-3.99758587587975	0.77633980697604	2.31043122709132
c	-3.14239777357514	0.70925873642817	1.21227613650945
h	-3.20781930724407	1.44673108850178	0.42144558110000
h	-1.36081078732532	-2.05480925992902	2.12225459689215
h	-2.87504756734790	-1.91748220843304	4.05068971239707
h	-4.57021721441769	-0.11104126712519	4.18421535852554
c	-1.22710605286697	-2.08506885049809	-0.98547419293742
h	-2.24572357732353	-2.20280803677111	-1.35674691207391
h	-0.52313403492875	-2.20704583828569	-1.80800566029830
c	0.70996815372876	-0.16768980421671	0.36064567373456
h	0.58179069624175	0.46869639077803	1.24133411550656
h	1.11700699839503	-1.12027988244642	0.70051646940177
c	1.55098566333742	0.51131778080931	-0.71582118630549
h	3.54809524478997	-1.61586011349323	-0.88767831311798
h	0.53860433058296	2.36335988387797	-0.33350019970584
c	0.89689845242869	1.73612455916023	-1.14231484020485
h	1.97204529813926	-2.39660823159545	-0.94783370859403
h	0.62969419005390	3.34513548004283	-2.53843027553393
c	1.30335146747634	2.50510661991285	-2.36654614237747
h	2.30998814303302	2.90911479622211	-2.20757561275716
h	1.33411460536130	1.88261310651695	-3.26080154214348
c	2.01527500978211	-0.40032366292132	-1.87487872778271
h	1.15473669521607	-0.58519953632063	-2.53497814318797
h	2.73360195942047	0.15727317814081	-2.47985346312810
c	2.65401608191597	-1.74233871223506	-1.49932692292495
cl	3.90706725717176	-0.36480829050706	1.66296107634636
al	3.23732780095913	1.25052737714832	0.39966833614449
cl	4.73282950672091	1.86063778950503	-1.01939035305816
cl	2.50230894657192	2.88180291113712	1.61218160724128

Intermediate VI, Scheme 10

p	-0.99632137978267	-0.44189716184330	-0.21191394402276
s	-1.06395689246212	0.99533613149766	-1.67163021204883
h	2.93130551505409	-2.47516439005053	-2.28179780988939
h	-4.48875546287370	2.05113703274817	2.26807169236729
h	-1.18577538908410	-2.85821539828955	-0.10027300225135
c	-2.20466447878159	-0.18696987501033	1.12630197907869
c	-2.29964838195184	-1.14196996071701	2.14951358462137
c	-3.18160956163497	-0.94138489122265	3.20675384197844
c	-3.97260416035384	0.20634451451734	3.24900315469281
c	-3.87732632812307	1.15707907709334	2.23561170243391
c	-2.99376701412986	0.96655804787546	1.17542713691785
h	-2.91627541118902	1.70850521415950	0.39011122467457
h	-1.68517401529783	-2.03546400333469	2.13799636315122
h	-3.24749301293722	-1.67900686075628	3.99781185024179
h	-4.65850155521159	0.35988250335498	4.07397044359904
c	-1.28868461661601	-2.11173719832697	-0.89014760624107
h	-2.29538035997492	-2.16437766338447	-1.30647873544903
h	-0.55616640059834	-2.30909330723263	-1.67197140301510
c	0.74779281483770	-0.23074849338166	0.40945848903923
h	0.64125404798649	0.43080083914927	1.27524855354751
h	1.13128457925527	-1.18544146320310	0.77129773839883
c	1.60075726457823	0.40451373587442	-0.69291202353263
h	3.55326328251914	-1.74782991104739	-0.79937476095258
h	0.61711491070780	2.30348233096373	-0.41666535657995
c	0.84964382672689	1.59924327472461	-1.21195088415218
h	1.97605734502651	-2.52684488796300	-0.80532742723723
h	0.69612594251758	3.17604045789035	-2.68219907630098
c	1.33823616268052	2.32382232285720	-2.45112895478342
h	2.34616331295741	2.70476420031044	-2.26333665258746
h	1.38242736407644	1.67198965056957	-3.32432250578792
c	2.01293147983534	-0.57240479450228	-1.82196862572039
h	1.14119284737819	-0.78672568869872	-2.45904494420951
h	2.72356041884263	-0.05376986192771	-2.46987702734754
c	2.65217095534924	-1.89951490995716	-1.39518474551747
cl	3.94573577240117	-0.39060226190946	1.71629835005052
al	3.27090559500281	1.14510530831675	0.34505240480998
cl	4.79722737368986	1.68057480755612	-1.08112162690513
cl	2.55695360957941	2.85398353330007	1.48069881492894

Compound 37, Scheme 10

p	-1.62472822185915	0.23933195752567	-0.75864787790931
s	0.88854847554417	2.23095020437233	-2.95203324498766
h	0.71065993446478	1.37011764990198	-0.06312295387043
h	-2.74059411723705	1.04905065247220	3.96623738503095
h	-2.60710809857965	-1.80906148864766	-1.68251052900978
c	-1.90843168250449	-0.31919303263840	0.96497792162125
c	-1.80831079002718	-1.65929843460048	1.36099674665932
c	-2.04282845169409	-2.02010224959096	2.68537314086805
c	-2.37775137228947	-1.04441352269543	3.62513189985748
c	-2.47915303612841	0.28992177012952	3.23754579948656
c	-2.24620609636052	0.65331178492754	1.91151930140584
h	-2.32482164518028	1.68654590365929	1.59279340132464
h	-1.54848730987683	-2.43003180541529	0.64223562925221
h	-1.96530123670033	-3.05940329103056	2.98454153787344
h	-2.55983627344454	-1.32637331811014	4.65618095580855
c	-2.77118196567952	-0.73499019459437	-1.79446030241425
h	-3.79602919146268	-0.49548868234186	-1.50619957869755
h	-2.62043552706781	-0.45171567686594	-2.83818398466111
c	0.05953584573597	-0.33430643342653	-1.23168609308861
h	0.17434916531823	-1.36960860203050	-0.89851024891927
h	0.08087653788600	-0.34155783034497	-2.32417974628053
c	1.18103988601855	0.57582622366413	-0.65476541077662
h	2.38059881225522	-2.02589390866277	-0.87827844971778
h	2.46565572569181	0.55719249117084	-2.40181099777853
c	1.98505721989379	1.30291607864958	-1.76132534159024
h	3.49764583713551	-1.85669018930590	0.47372255696944
h	3.81762102689719	1.69275992957008	-0.63558216296379
c	3.05632633361048	2.24192262981373	-1.19796507227260
h	3.55789422843784	2.77683590499736	-2.00592779600966
h	2.60812796767933	2.98745542141343	-0.53489512059170
c	2.08109323505973	-0.21716188067504	0.32614928427930
h	2.71150035121374	0.48343595198606	0.88071532920609
h	1.42343088692248	-0.67490203783137	1.07316661421574
c	2.96441250786677	-1.30132627778699	-0.30249862833114
h	3.71612819634730	-0.87840629993978	-0.97424315680757
h	-0.13284245269691	2.48219477432922	-2.09668439798285
o	-1.80645470518997	1.73015582795190	-0.88777640919775

References

[1] Parr, R. G.; Yang, W. in *Density-functional Theory of Atoms and Molecules*, Oxford University Press, New York, 1989.

[2] Becke, A. D. *J. Chem. Phys.* **1993**, *98*, 5648-5652.

[3] Krishnan, R.; Binkley, J. S.; Seeger, R.; Pople, J. A. *J Chem Phys.* **1980**, *72*, 650-654.

[4] Frisch, M. J.; People, J. A.; Binkley, J. S. *J Chem Phys.* **1984**, *80*, 3265-3269.

[5] Haake, P.; Cook, R. D.; Hurst, G. H. *J. Am. Chem. Soc.* **1967**, *89*, 2650-2654.

[6] Crofts, P. C.; Fox, S. *J. Chem. Soc. B* **1968**, 1416-1420.

[7] Włodarczyk, K.; Stankevič, M. *Tetrahedron* **2016,** *72,* 5074-5090.

[8] Perrier, A.; Comte, V.; Moïse, C.; Richard, P.; Le Gendre, P. *Eur. J. Org. Chem.* **2010**, 1562-1568.
