# Supplementary material for: [1,3]/[1,4]-Sulfur atom migration in β-hydroxyalkylphosphine sulfides
Source: Beilstein J Org Chem. 2020 Jan 21;16:88–105. doi: 10.3762/bjoc.16.11 (PMC7006489; doi:10.3762/bjoc.16.11)
Supplement: File 2 — Copies of NMR spectra of all pure compounds and their mixtures. [file Beilstein_J_Org_Chem-16-88-s002.pdf]

BEILSTEIN JOURNAL OF ORGANIC CHEMISTRY

Supporting Information

for

[1,3]/[1,4]-Sulfur atom migration in β-hydroxyalkylphosphine sulfides

Katarzyna Włodarczyk, Piotr Borowski and Marek Stankevič

Beilstein J. Org. Chem. **2020,** *16,* 88–105. doi:10.3762/bjoc.16.11

Copies of NMR spectra of all pure compounds and their mixtures

License and Terms: This is a supporting information file under the terms of the Creative Commons Attribution License (https://creativecommons.org/licenses/by/4.0). Please note that the reuse, redistribution and reproduction in particular requires that the authors and source are credited.
The license is subject to the *Beilstein Journal of Organic Chemistry* terms and conditions: (https://www.beilstein-journals.org/bjoc)

Table of Contents

^{1}H NMR spectrum of (2-hydroxypropyl)methylphenylphosphine sulfide (major diastereomer) (**6**) S11

^{31}P NMR spectrum of (2-hydroxypropyl)methylphenylphosphine sulfide (major diastereomer) (**6**) S12

^{13}C NMR spectrum of (2-hydroxypropyl)methylphenylphosphine sulfide (major diastereomer) (**6**) S13

^{1}H NMR spectrum of (2-hydroxypropyl)methylphenylphosphine sulfide (minor diastereomer) (**6**) S14

^{31}P NMR spectrum of (2-hydroxypropyl)methylphenylphosphine sulfide (minor diastereomer) (**6**) S15

^{13}C NMR spectrum of (2-hydroxypropyl)methylphenylphosphine sulfide (minor diastereomer) (**6**) S16

^{1}H NMR spectrum of (2-hydroxybutyl)methylphenylphosphine sulfide (major diastereomer) (**7**) S17

^{31}P NMR spectrum of (2-hydroxybutyl)methylphenylphosphine sulfide (major diastereomer) (**7**) S18

^{13}C NMR spectrum of (2-hydroxybutyl)methylphenylphosphine sulfide (major diastereomer) (**7**) S19

^{1}H NMR spectrum of (2-hydroxybutyl)methylphenylphosphine sulfide (minor diastereomer) (**7**) S20

^{31}P NMR spectrum of (2-hydroxybutyl)methylphenylphosphine sulfide (minor diastereomer) (**7**) S21

^{13}C NMR spectrum of (2-hydroxybutyl)methylphenylphosphine sulfide (minor diastereomer) (**7**) S22

^{1}H NMR spectrum of (2-hydroxy-3-methylbutyl)methylphenylphosphine sulfide (major diastereomer) (**8**) S23

^{31}P NMR spectrum of (2-hydroxy-3-methylbutyl)methylphenylphosphine sulfide (major diastereomer) (**8**) S24

^{13}C NMR spectrum of (2-hydroxy-3-methylbutyl)methylphenylphosphine sulfide (major diastereomer) (**8**) S25

^{1}H NMR spectrum of (2-hydroxy-3-methylbutyl)methylphenylphosphine sulfide (minor diastereomer) (**8**) S26

^{31}P NMR spectrum of (2-hydroxy-3-methylbutyl)methylphenylphosphine sulfide (minor diastereomer) (**8**) S27

^{13}C NMR spectrum of (2-hydroxy-3-methylbutyl)methylphenylphosphine sulfide (minor diastereomer) (**8**) S28

^{1}H NMR spectrum of (2-hydroxy-3,3-dimethylbutyl)methylphenylphosphine sulfide (**9**) S29

^{31}P NMR spectrum of (2-hydroxy-3,3-dimethylbutyl)methylphenylphosphine sulfide (**9**) S30

^{13}C NMR spectrum of (2-hydroxy-3,3-dimethylbutyl)methylphenylphosphine sulfide (**9**) S31

^{1}H NMR spectrum of (2-hydroxy-2-phenylethyl)methylphenylphosphine sulfide (**10**) S32

^{31}P NMR spectrum of (2-hydroxy-2-phenylethyl)methylphenylphosphine sulfide (**10**) S33

^{13}C NMR spectrum of (2-hydroxy-2-phenylethyl)methylphenylphosphine sulfide (**10**) S34

^{1}H NMR spectrum of (2-cyclohexyl-2-hydroxyethyl)methylphenylphosphine sulfide (**11**) S35

^{31}P NMR spectrum of (2-cyclohexyl-2-hydroxyethyl)methylphenylphosphine sulfide (**11**) S36

^{13}C NMR spectrum of (2-cyclohexyl-2-hydroxyethyl)methylphenylphosphine sulfide (**11**) S37

^{1}H NMR spectrum of (2-hydroxy-2-methylbutyl)methylphenylphosphine sulfide (major diastereomer) (**12**) S38

^{31}P NMR spectrum of (2-hydroxy-2-methylbutyl)methylphenylphosphine sulfide (major diastereomer) (**12**) S39

^{13}C NMR spectrum of (2-hydroxy-2-methylbutyl)methylphenylphosphine sulfide (major diastereomer) (**12**) S40

^{1}H NMR spectrum of (2-hydroxy-2-methylbutyl)methylphenylphosphine sulfide (minor diastereomer) (**12**) S41

^{31}P NMR spectrum of (2-hydroxy-2-methylbutyl)methylphenylphosphine sulfide (minor diastereomer) (**12**) S42

^{13}C NMR spectrum of (2-hydroxy-2-methylbutyl)methylphenylphosphine sulfide (minor diastereomer) (**12**) S43

^{1}H NMR spectrum of (2-hydroxy-2-phenylpropyl)methylphenylphosphine sulfide (major diastereomer) (**13**) S44

^{31}P NMR spectrum of (2-hydroxy-2-phenylpropyl)methylphenylphosphine sulfide (major diastereomer) (**13**) S45

^{13}C NMR spectrum of (2-hydroxy-2-phenylpropyl)methylphenylphosphine sulfide (major diastereomer) (**13**) S46

^{1}H NMR spectrum of (2-hydroxy-2-phenylpropyl)methylphenylphosphine sulfide (minor diastereomer) (**13**) S47

^{31}P NMR spectrum of (2-hydroxy-2-phenylpropyl)methylphenylphosphine sulfide (minor diastereomer) (**13**) S48

^{13}C NMR spectrum of (2-hydroxy-2-phenylpropyl)methylphenylphosphine sulfide(minor diastereomer) (**13**) S49

^{1}H NMR spectrum of (2-hydroxy-2-methylhexyl)methylphenylphosphine sulfide (major diastereomer) (**14**) S50

^{31}P NMR spectrum of (2-hydroxy-2-methylhexyl)methylphenylphosphine sulfide (major diastereomer) (**14**) S51

^{13}C NMR spectrum of (2-hydroxy-2-methylhexyl)methylphenylphosphine sulfide (major diastereomer) (**14**) S52

^{1}H NMR spectrum of (2-hydroxy-2-methylhexyl)methylphenylphosphine sulfide (minor diastereomer) (**14**) S53

^{31}P NMR spectrum of (2-hydroxy-2-methylhexyl)methylphenylphosphine sulfide (minor diastereomer) (**14**) S54

^{13}C NMR spectrum of (2-hydroxy-2-methylhexyl)methylphenylphosphine sulfide (minor diastereomer) (**14**) S55

^{1}H NMR spectrum of (2-hydroxy-2-methylpentyl)methylphenylphosphine sulfide (**15**) S56

^{31}P NMR spectrum of (2-hydroxy-2-methylpentyl)methylphenylphosphine sulfide (**15**) S57

^{13}C NMR spectrum of (2-hydroxy-2-methylpentyl)methylphenylphosphine sulfide (**15**) S58

^{1}H NMR spectrum of (2-hydroxy-2,3-dimethylbutyl)methylphenylphosphine sulfide (**16**) S59

^{31}P NMR spectrum of (2-hydroxy-2,3-dimethylbutyl)methylphenylphosphine sulfide (**16**) S60

^{13}C NMR spectrum of (2-hydroxy-2,3-dimethylbutyl)methylphenylphosphine sulfide (**16**) S61

^{1}H NMR spectrum of (2-hydroxy-2,3,3-trimethylbutyl)methylphenylphosphine sulfide (**17**) S62

^{31}P NMR spectrum of (2-hydroxy-2,3,3-trimethylbutyl)methylphenylphosphine sulfide (**17**) S63

^{13}C NMR spectrum of (2-hydroxy-2,3,3-trimethylbutyl)methylphenylphosphine sulfide (**17**) S64

^{1}H NMR spectrum of (2-hydroxy-2,4,4-trimethylpentyl)methylphenylphosphine sulfide (**18**) S65

^{31}P NMR spectrum of (2-hydroxy-2,4,4-trimethylpentyl)methylphenylphosphine sulfide (**18**) S66

^{13}C NMR spectrum of (2-hydroxy-2,4,4-trimethylpentyl)methylphenylphosphine sulfide (**18**) S67

^{1}H NMR spectrum of (2-hydroxy-2-methylpropyl)methylphenylphosphine sulfide (**19**) S68

^{31}P NMR spectrum of (2-hydroxy-2-methylpropyl)methylphenylphosphine sulfide (**19**) S69

^{13}C NMR spectrum of (2-hydroxy-2-methylpropyl)methylphenylphosphine sulfide (**19**) S70

^{1}H NMR spectrum of (2-ethyl-2-hydroxybutyl)methylphenylphosphine sulfide (**20**) S71

^{31}P NMR spectrum of (2-ethyl-2-hydroxybutyl)methylphenylphosphine sulfide (**20**) S72

^{13}C NMR spectrum of (2-ethyl-2-hydroxybutyl)methylphenylphosphine sulfide (**20**) S73

^{1}H NMR spectrum of (2-hydroxy-2-isopropyl-3-methylbutyl)methylphenylphosphine sulfide (**21**) S74

^{31}P NMR spectrum of (2-hydroxy-2-isopropyl-3-methylbutyl)methylphenylphosphine sulfide (**21**) S75

^{13}C NMR spectrum of (2-hydroxy-2-isopropyl-3-methylbutyl)methylphenylphosphine sulfide (**21**) S76

^{1}H NMR spectrum of [(1-hydroxy)cyclopentylmethyl)]methylphenylphosphine sulfide (**22**) S77

^{31}P NMR spectrum of [(1-hydroxy)cyclopentylmethyl)]methylphenylphosphine sulfide (**22**) S78

^{13}C NMR spectrum of [(1-hydroxy)cyclopentylmethyl)]methylphenylphosphine sulfide (**22**) S79

^{1}H NMR spectrum of [(1-hydroxy)cyclohexylmethyl)]methylphenylphosphine sulfide (**23**) S80

^{31}P NMR spectrum of [(1-hydroxy)cyclohexylmethyl)]methylphenylphosphine sulfide (**23**) S81

^{13}C NMR spectrum of [(1-hydroxy)cyclohexylmethyl)]methylphenylphosphine sulfide (**23**) S82

^{1}H NMR spectrum of [(1-hydroxy)cycloheptylmethyl)]methylphenylphosphine sulfide (**24**) S83

^{31}P NMR spectrum of [(1-hydroxy)cycloheptylmethyl)]methylphenylphosphine sulfide (**24**) S84

^{13}C NMR spectrum of [(1-hydroxy)cycloheptylmethyl)]methylphenylphosphine sulfide (**24**) S85

^{1}H NMR spectrum of (2-butyl-2-hydroxyhexyl)methylphenylphosphine sulfide (**25**) S86

^{31}P NMR spectrum of (2-butyl-2-hydroxyhexyl)methylphenylphosphine sulfide (**25**) S87

^{13}C NMR spectrum of (2-butyl-2-hydroxyhexyl)methylphenylphosphine sulfide (**25**) S88

^{1}H NMR spectrum of (S_P)-(2-hydroxy-3-methylbutyl)methylphenylphosphine sulfide (S_P)-(**8**) S89

^{31}P NMR spectrum of (S_P)-(2-hydroxy-3-methylbutyl)methylphenylphosphine sulfide (S_P)-(**8**) S90

^{13}C NMR spectrum of (S_P)-(2-hydroxy-3-methylbutyl)methylphenylphosphine sulfide (S_P)-(**8**) S91

^{1}H NMR spectrum of (S_P)-(2-hydroxy-2-methylpropyl)methylphenylphosphine sulfide (S_P)-(**19**) S92

^{31}P NMR spectrum of (S_P)-(2-hydroxy-2-methylpropyl)methylphenylphosphine sulfide (S_P)-(**19**) S93

^{13}C NMR spectrum of (S_P)-(2-hydroxy-2-methylpropyl)methylphenylphosphine sulfide (S_P)-(**19**) S94

^{1}H NMR spectrum of (S_P)-(2-ethyl-2-hydroxybutyl)methylphenylphosphine sulfide (S_P)-(**20**) S95

^{31}P NMR spectrum of (S_P)-(2-ethyl-2-hydroxybutyl)methylphenylphosphine sulfide (S_P)-(**20**) S96

^{13}C NMR spectrum of (S_P)-(2-ethyl-2-hydroxybutyl)methylphenylphosphine sulfide (S_P)-(**20**) S97

^{1}H NMR spectrum of (2-methyl-3-mercaptobutyl)methylphenylphosphine oxide (**29**) S98

^{31}P NMR spectrum of (2-methyl-3-mercaptobutyl)methylphenylphosphine oxide (**29**) S199

^{13}C NMR spectrum of (2-methyl-3-mercaptobutyl)methylphenylphosphine oxide (**29**) S100

^{1}H NMR spectrum of (2-methyl-3-mercaptohexyl)methylphenylphosphine oxide (**30**) S101

^{31}P NMR spectrum of (2-methyl-3-mercaptohexyl)methylphenylphosphine oxide (**30**) S102

^{13}C NMR spectrum of (2-methyl-3-mercaptohexyl)methylphenylphosphine oxide (**30**) S103

^{1}H NMR spectrum of (2-methyl-3-mercaptopentyl)methylphenylphosphine oxide (**31**) S104

^{31}P NMR spectrum of (2-methyl-3-mercaptopentyl)methylphenylphosphine oxide (**31**) S105

^{13}C NMR spectrum of (2-methyl-3-mercaptopentyl)methylphenylphosphine oxide (**31**) S106

^{1}H NMR spectrum of (2,3-dimethyl-3-mercaptobutyl)methylphenylphosphine oxide (**32**) S107

^{31}P NMR spectrum of (2,3-dimethyl-3-mercaptobutyl)methylphenylphosphine oxide (**32**) S108

^{13}C NMR spectrum of (2,3-dimethyl-3-mercaptobutyl)methylphenylphosphine oxide (**32**) S109

^{1}H NMR spectrum of (2,2-dimethyl-3-mercaptobutyl)methylphenylphosphine oxide (**33**) S110

^{31}P NMR spectrum of (2,2-dimethyl-3-mercaptobutyl)methylphenylphosphine oxide (**33**) S111

^{13}C NMR spectrum of (2,2-dimethyl-3-mercaptobutyl)methylphenylphosphine oxide (**33**) S112

^{1}H NMR spectrum of (2-methylprop-1-enyl)(methylphenyl)phosphine oxide (**34**) S113

^{31}P NMR spectrum of (2-methylprop-1-enyl)(methylphenyl)phosphine oxide (**34**) S114

^{13}C NMR spectrum of (2-methylprop-1-enyl)(methylphenyl)phosphine oxide (**34**) S115

^{1}P NMR spectrum of 1,3,3-trimethylphosphindoline 1-sulfide (**35**) with (2-methylprop-1-enyl)(methylphenyl)phosphine oxide (**34**) and 1,3,3-trimethylphosphindoline 1-oxide (**3**) and (2-hydroxy-2-methylpropyl)methylphenylphosphine sulfide (**19**) S116

^{1}H NMR spectrum of (2-ethyl-3-thiolobutyl)methylphenylphosphine oxide (**36**) S117

^{31}P NMR spectrum of (2-ethyl-3-mercaptobutyl)methylphenylphosphine oxide (**36**) S118

^{13}C NMR spectrum of (2-ethyl-3-mercaptobutyl)methylphenylphosphine oxide (**36**) S119

^{1}H NMR spectrum of (2-isopropyl-3-methyl-3-mercaptobutyl)methylphenylphosphine oxide (**37**) S120

^{31}P NMR spectrum of (2-isopropyl-3-methyl-3-mercaptobutyl)methylphenylphosphine oxide (**37**) S121

^{13}C NMR spectrum of (2-isopropyl-3-methyl-3-mercaptobutyl)methylphenylphosphine oxide (**37**) S122

^{1}H NMR spectrum of [(2-mercapto)cyclopentylmethyl)]methylphenylphosphine sulfide (**38**) S123

^{31}P NMR spectrum of [(2-mercapto)cyclopentylmethyl)]methylphenylphosphine sulfide (**38**) S124

^{13}C NMR spectrum of [(2-mercapto)cyclopentylmethyl)]methylphenylphosphine sulfide (**38**) S125

^{1}H NMR spectrum of [(2-mercapto)cyclohexylmethyl)]methylphenylphosphine sulfide (**39**) S126

^{31}P NMR spectrum of [(2-mercapto)cyclohexylmethyl)]methylphenylphosphine sulfide (**39**) S127

^{13}C NMR spectrum of [(2-mercapto)cyclohexylmethyl)]methylphenylphosphine sulfide (**39**) S128

^{1}H NMR spectrum of [(2-mercapto)cycloheptylmethyl)]methylphenylphosphine sulfide (**40**) S129

^{31}P NMR spectrum of [(2-mercapto)cycloheptylmethyl)]methylphenylphosphine sulfide (**40**) S130

^{13}C NMR spectrum of [(2-mercapto)cycloheptylmethyl)]methylphenylphosphine sulfide (**40**) S131

^{1}H NMR spectrum of (2-butyl-3-mercaptohexyl)methylphenylphosphine oxide (**41**) S132

^{31}P NMR spectrum of (2-butyl-3-mercaptohexyl)methylphenylphosphine oxide (**41**) S133

^{13}C NMR spectrum of (2-butyl-3-mercaptohexyl)methylphenylphosphine oxide (**41**) S134

^{1}H NMR spectrum of (2-acetoxypropyl)methylphenylphosphine sulfide (**42**) S135

^{31}P NMR spectrum of (2-acetoxypropyl)methylphenylphosphine sulfide (**42**) S136

^{13}C NMR spectrum of (2-acetoxypropyl)methylphenylphosphine sulfide (**42**) S137

^{1}H NMR spectrum of (2-acetoxybutyl)methylphenylphosphine sulfide (**43**) S138

^{31}P NMR spectrum of (2-acetoxybutyl)methylphenylphosphine sulfide (**43**) S139

^{13}C NMR spectrum of (2-acetoxybutyl)methylphenylphosphine sulfide (**43**) S140

^{1}H NMR spectrum of (2-acetoxy-3-methylbutyl)methylphenylphosphine sulfide (**44**) S141

^{31}P NMR spectrum of (2-acetoxy-3-methylbutyl)methylphenylphosphine sulfide (**44**) S142

^{13}C NMR spectrum of (2-acetoxy-3-methylbutyl)methylphenylphosphine sulfide (**44**) S143

^{1}H NMR spectrum of (3-methyl-3-thiolobutyl)methylphenylphosphine oxide (**45**) S144

^{31}P NMR spectrum of (3-methyl-3-thiolobutyl)methylphenylphosphine oxide (**45**) S145

^{13}C NMR spectrum of (3-methyl-3-thiolobutyl)methylphenylphosphine oxide (**45**) S146

^{1}H NMR spectrum of (R_P)-(3-methyl-3-mercaptobutyl)methylphenylphosphine oxide (R_P)-(**45**) S147

^{31}P NMR spectrum of (R_P)-(3-methyl-3-mercaptobutyl)methylphenylphosphine oxide (R_P)-(**45**) S148

^{13}C NMR spectrum of (R_P)-(3-methyl-3-mercaptobutyl)methylphenylphosphine oxide (R_P)-(**45**) S149

^{1}H NMR spectrum of (2-acetoxy-3,3-dimethylbutyl)methylphenylphosphine sulfide (**46**) S150

^{31}P NMR spectrum of (2-acetoxy-3,3-dimethylbutyl)methylphenylphosphine sulfide (**46**) S151

^{13}C NMR spectrum of (2-acetoxy-3,3-dimethylbutyl)methylphenylphosphine sulfide (**46**) S152

^{1}H NMR spectrum of (2-acetoxy-2-cyclohexylethyl)methylphenylphosphine sulfide (**47**) S153

^{31}P NMR spectrum of (2-acetoxy-2-cyclohexylethyl)methylphenylphosphine sulfide (**47**) S154

^{13}C NMR spectrum of (2-acetoxy-2-cyclohexylethyl)methylphenylphosphine sulfide (**47**) S155

^{1}H NMR spectrum of [(1-mercapto)cyclohexylethyl)]methylphenylphosphine sulfide (**48**) S156

^{31}P NMR spectrum of [(1-mercapto)cyclohexylethyl)]methylphenylphosphine sulfide (**48**) S157

^{13}C NMR spectrum of [(1-mercapto)cyclohexylethyl)]methylphenylphosphine sulfide (**48**) S158

^{1}H NMR spectrum of (2-methyl-2-mercaptobutyl)methylphenylphosphine oxide (**49**) S159

^{31}P NMR spectrum of (2-methyl-2-mercaptobutyl)methylphenylphosphine oxide (**49**) S160

^{13}C NMR spectrum of (2-methyl-2-mercaptobutyl)methylphenylphosphine oxide (**49**) S161

^{1}H NMR spectrum of (2-methyl-2-mercaptohexyl)methylphenylphosphine oxide (**50**) S162

^{31}P NMR spectrum of (2-methyl-2-mercaptohexyl)methylphenylphosphine oxide (**50**) S163

^{13}C NMR spectrum of (2-methyl-2-mercaptohexyl)methylphenylphosphine oxide (**50**) S164

^{1}H NMR spectrum of (2-methyl-2-mercaptopentyl)methylphenylphosphine oxide (**51**) S165

^{31}P NMR spectrum of (2-methyl-2-mercaptopentyl)methylphenylphosphine oxide (**51**) S166

^{13}C NMR spectrum of (2-methyl-2-mercaptopentyl)methylphenylphosphine oxide (**51**) S167

^{1}H NMR spectrum of (2-methyl-2-mercaptopropyl)methylphenylphosphine oxide (**52**) S168

^{31}P NMR spectrum of (2-methyl-2-mercaptopropyl)methylphenylphosphine oxide (**52**) S169

^{13}C NMR spectrum of (2-methyl-2-mercaptopropyl)methylphenylphosphine oxide (**52**) S170

^{1}H NMR spectrum of (2-ethyl-2-mercaptobutyl)methylphenylphosphine oxide (**53**) S171

^{31}P NMR spectrum of (2-ethyl-2-mercaptobutyl)methylphenylphosphine oxide (**53**) S172

^{13}C NMR spectrum of (2-ethyl-2-mercaptobutyl)methylphenylphosphine oxide (**53**) S173

^{1}H NMR spectrum of [(1-mercapto)cyclopentylmethyl)]methylphenylphosphine oxide (**54**) S174

^{31}P NMR spectrum of [(1-mercapto)cyclopentylmethyl)]methylphenylphosphine oxide (**54**) S175

^{13}C NMR spectrum of [(1-mercapto)cyclopentylmethyl)]methylphenylphosphine oxide (**54**) S176

^{1}H NMR spectrum of [(1-mercapto)cyclohexylmethyl)]methylphenylphosphine oxide (**55**) S177

^{31}P NMR spectrum of [(1-mercapto)cyclohexylmethyl)]methylphenylphosphine oxide (**55**) S178

^{13}C NMR spectrum of [(1-mercapto)cyclohexylmethyl)]methylphenylphosphine oxide (**55**) S179

^{1}H NMR spectrum of [(1-mercapto)cycloheptylmethyl)]methylphenylphosphine oxide (**56**) S180

^{31}P NMR spectrum of [(1-mercapto)cycloheptylmethyl)]methylphenylphosphine oxide (**56**) S181

^{13}C NMR spectrum of [(1-mercapto)cycloheptylmethyl)]methylphenylphosphine oxide (**56**) S182

^{1}H NMR spectrum of (2-butyl-2-mercaptohexyl)methylphenylphosphine sulfide (**57**) S183

^{31}P NMR spectrum of (2-butyl-2-mercaptohexyl)methylphenylphosphine sulfide (**57**) S184

^{13}C NMR spectrum of (2-butyl-2-mercaptohexyl)methylphenylphosphine sulfide (**57**) S185

^{1}H NMR spectrum of (R_P)-(2-methyl-2-mercaptopropyl)methylphenylphosphine oxide (R_P)-(**52**) S186

^{31}P NMR spectrum of (R_P)-(2-methyl-2-mercaptopropyl)methylphenylphosphine oxide (R_P)**-(52**) S187

^{13}C NMR spectrum of (R_P)-(2-methyl-2-mercaptopropyl)methylphenylphosphine oxide (R_P)**-(52**) S188

^{1}H NMR spectrum of (R_P)-(2-ethyl-2-mercaptobutyl)methylphenylphosphine oxide (R_P)**-(53**) S189

^{31}P NMR spectrum of (R_P)-(2-ethyl-2-mercaptobutyl)methylphenylphosphine oxide (R_P)**-(53**) S190

^{13}C NMR spectrum of (R_P)-(2-ethyl-2-mercaptobutyl)methylphenylphosphine oxide (R_P)-(**53**) S191

^{1}H NMR spectrum of (R_P)-1,4,4-Trimethyl-1,2,3,4-tetrahydrophosphinoline 1-oxide (R_P)**-26** S192

^{31}P NMR spectrum of (R_P)-1,4,4-Trimethyl-1,2,3,4-tetrahydrophosphinoline 1- oxide (R_P)**-26** S193

^{13}C NMR spectrum of (R_P)-1,4,4-Trimethyl-1,2,3,4-tetrahydrophosphinoline 1-oxide (R_P)**-26** S194

^{1}H NMR spectrum of (R_P)-1,3,3-Trimethylphosphindoline 1-oxide (R_P)**-3** S195

^{31}P NMR spectrum of (R_P)-1,3,3-Trimethylphosphindoline 1-oxide (R_P)**-3** S196

^{13}C NMR spectrum of (R_P)-1,3,3-Trimethylphosphindoline 1-oxide(R_P)**-3** S197

^{1}H NMR spectrum of (2-ethylbut-2-enyl)(methylphenyl)phosphine oxide (**58**) S198

^{31}P NMR spectrum of (2-ethylbut-2-enyl)(methylphenyl)phosphine oxide (**58**) S199

^{13}C NMR spectrum of (2-ethylbut-2-enyl)(methylphenyl)phosphine oxide (**58**) S200

^{1}H NMR spectrum of (2-mesyloxy-3-methylbutyl)methylphenylphosphine sulfide (**59**) S201

^{31}P NMR spectrum of (2- mesyloxy-3-methylbutyl)methylphenylphosphine sulfide (**59**)	S202
^{13}C NMR spectrum of (2- mesyloxy-3-methylbutyl)methylphenylphosphine sulfide (**59**)	S203
^{1}H NMR spectrum of (S_P)-(2- mesyloxy-3-methylbutyl)methylphenylphosphine sulfide (***S*P)-(59**)	S204
^{31}P NMR spectrum of (S_P)-(2- mesyloxy-3-methylbutyl)methylphenylphosphine sulfide (***S*P)-(59**)	S205
^{13}C NMR spectrum of (S_P)-(2- mesyloxy-3-methylbutyl)methylphenylphosphine sulfide (***S*P)-(59**)	S206
^{1}H NMR spectrum of (2- mesyloxy-3,3-dimethylbutyl)methylphenylphosphine sulfide (**60**)	S207
^{31}P NMR spectrum of (2- mesyloxy-3,3-dimethylbutyl)methylphenylphosphine sulfide (**60**)	S208
^{13}C NMR spectrum of (2- mesyloxy-3,3-dimethylbutyl)methylphenylphosphine sulfide (**60**)	S209
^{1}H NMR spectrum of mixture of (2-methylbut-2-enyl)(methylphenyl)phosphine oxide (**61**) and (2-ethylprop-2-enyl)(methylphenyl)phosphine oxide (**62**)	S210
^{31}P NMR spectrum of mixture of (2-methylbut-2-enyl)(methylphenyl)phosphine oxide (**61**) and (2-ethylprop-2-enyl)(methylphenyl)phosphine oxide (**62**) (CDCl3, 202 MHz).	S211
^{13}C NMR spectrum of mixture of (2-methylbut-2-enyl)(methylphenyl)phosphine oxide (**61**) and (2-ethylprop-2-enyl)(methylphenyl)phosphine oxide (**62**)	S212
^{1}H NMR spectrum of (2,3-dimethylbut-2-enyl)(methylphenyl)phosphine oxide (**63**)	S213
^{31}P NMR spectrum of (2,3-dimethylbut-2-enyl)(methylphenyl)phosphine oxide (**63**)	S214
^{13}C NMR spectrum of (2,3-dimethylbut-2-enyl)(methylphenyl)phosphine oxide (**63**)	S215
^{1}H NMR spectrum of (2-methylprop-2-enyl)(methylphenyl)phosphine sulfide (**64**)	S216
^{31}P NMR spectrum of (2-methylprop-2-enyl)(methylphenyl)phosphine sulfide (**64**)	S217
^{13}C NMR spectrum of (2-methylprop-2-enyl)(methylphenyl)phosphine sulfide (**64**)	S218
^{1}H NMR spectrum of (S_P)-(2-methylprop-2-enyl)(methylphenyl)phosphine oxide (***S*P)-(64**)	S219
^{31}P NMR spectrum of (S_P)-(2-methylprop-2-enyl)(methylphenyl)phosphine oxide (***S*P)-(64**)	S220
^{13}C NMR spectrum of (S_P)-(2-methylprop-2-enyl)(methylphenyl)phosphine oxide (***S*P)-(64**)	S221

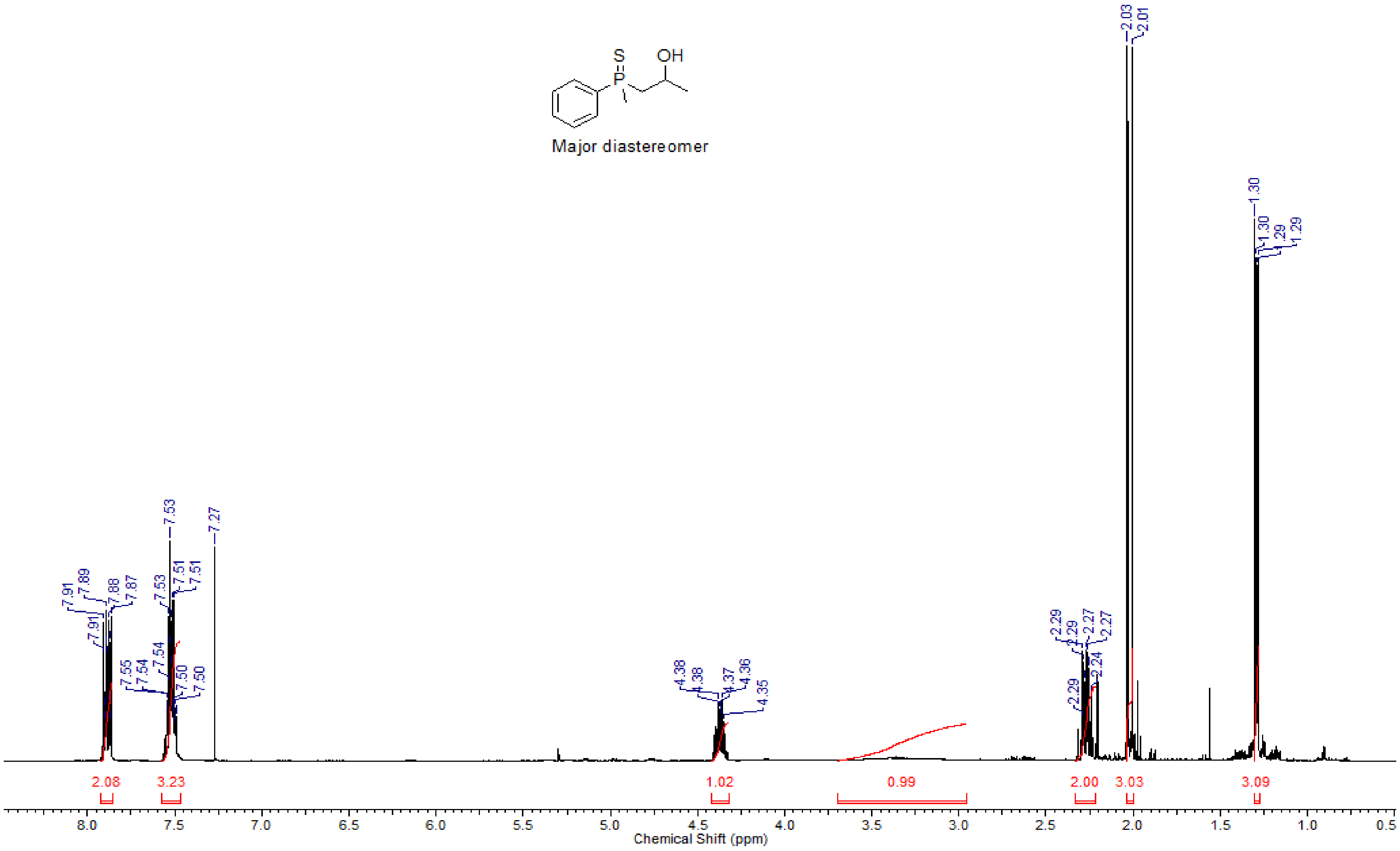

^{1}H NMR spectrum of (2-hydroxypropyl)methylphenylphosphine sulfide (major diastereomer) (**6**) (CDCl3, 500 MHz).

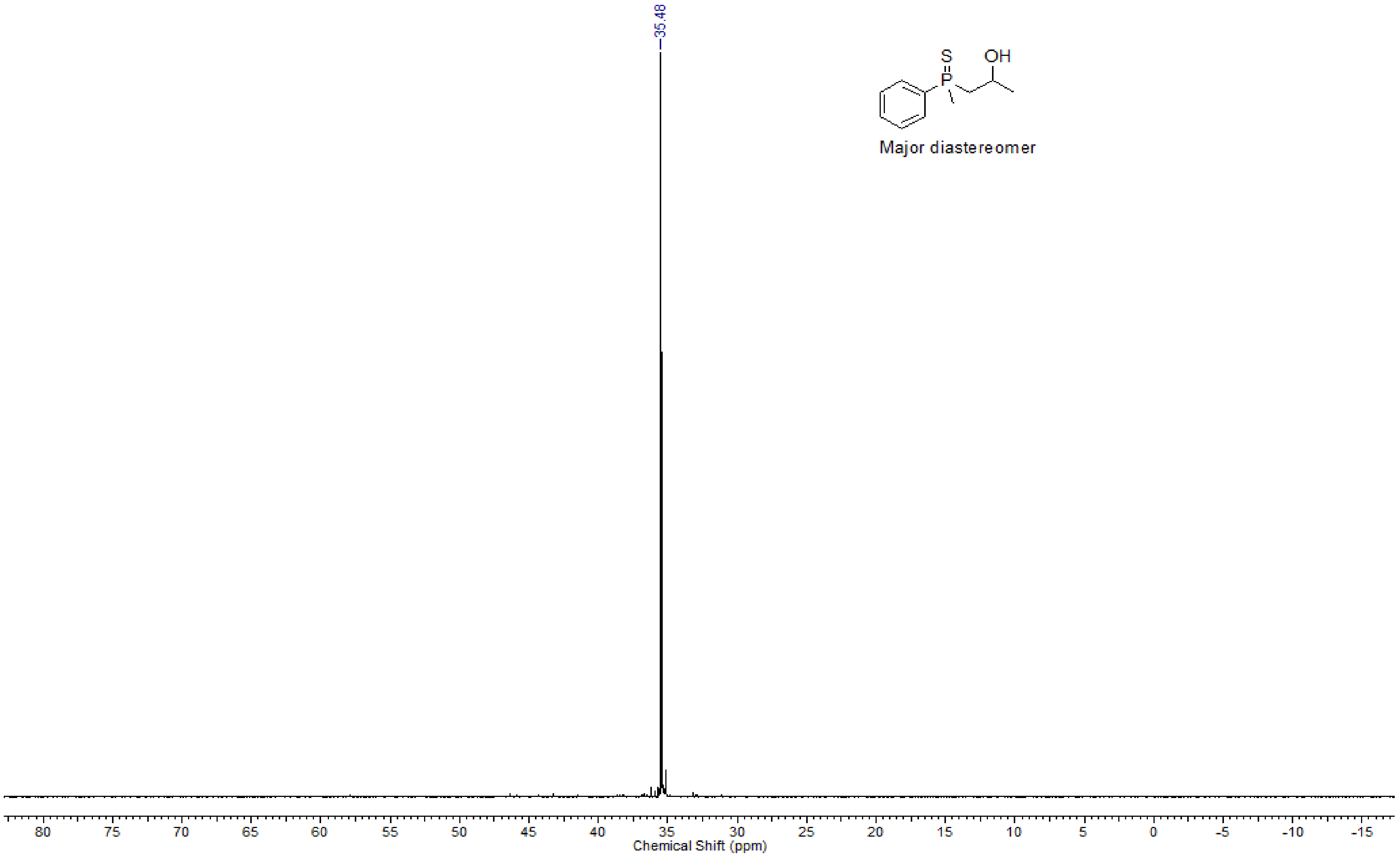

^{31}P NMR spectrum of (2-hydroxypropyl)methylphenylphosphine sulfide (major diastereomer) (**6**) (CDCl3, 202 MHz).

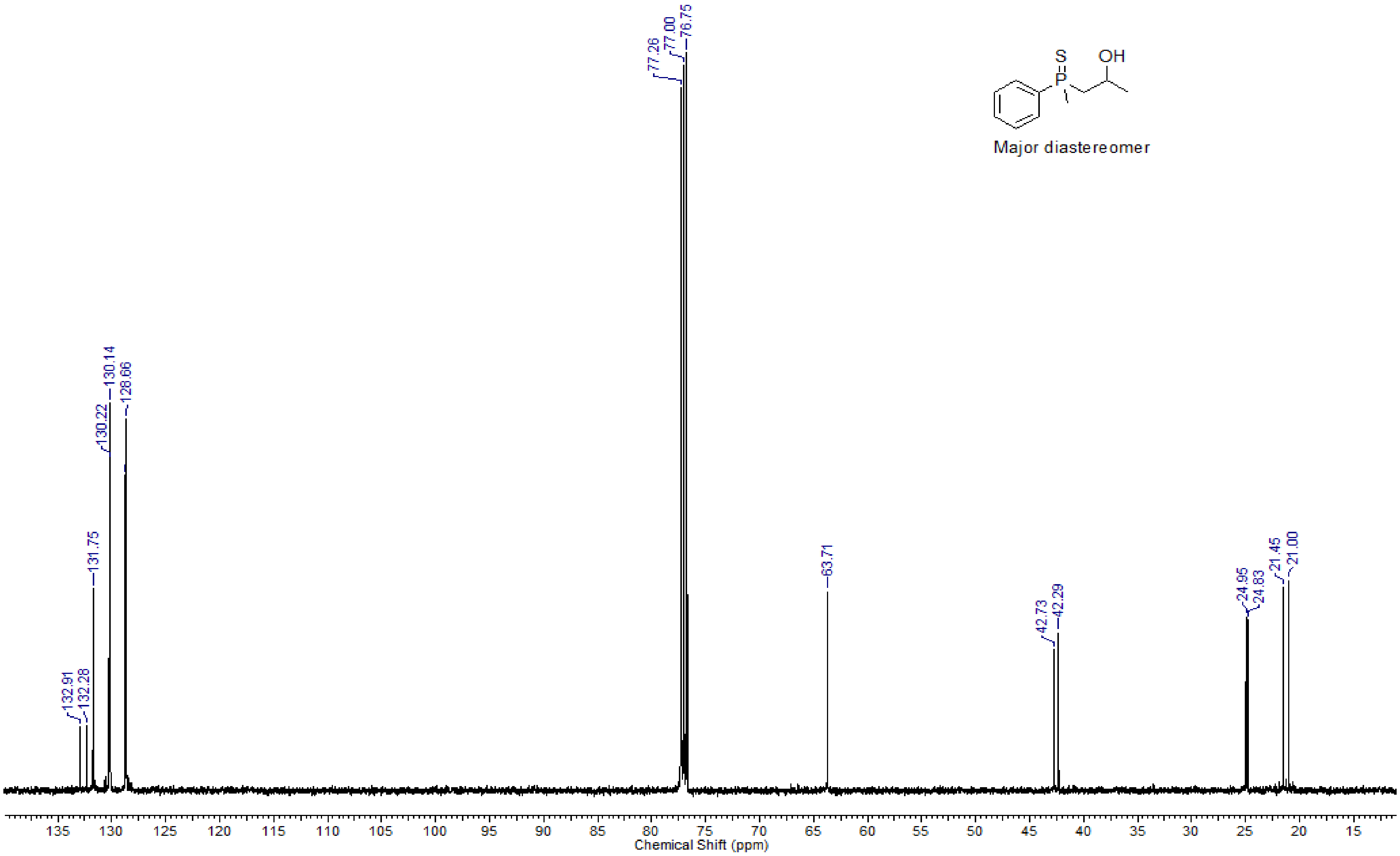

^{13}C NMR spectrum of (2-hydroxypropyl)methylphenylphosphine sulfide (major diastereomer) (**6**) (CDCl3, 126 MHz).

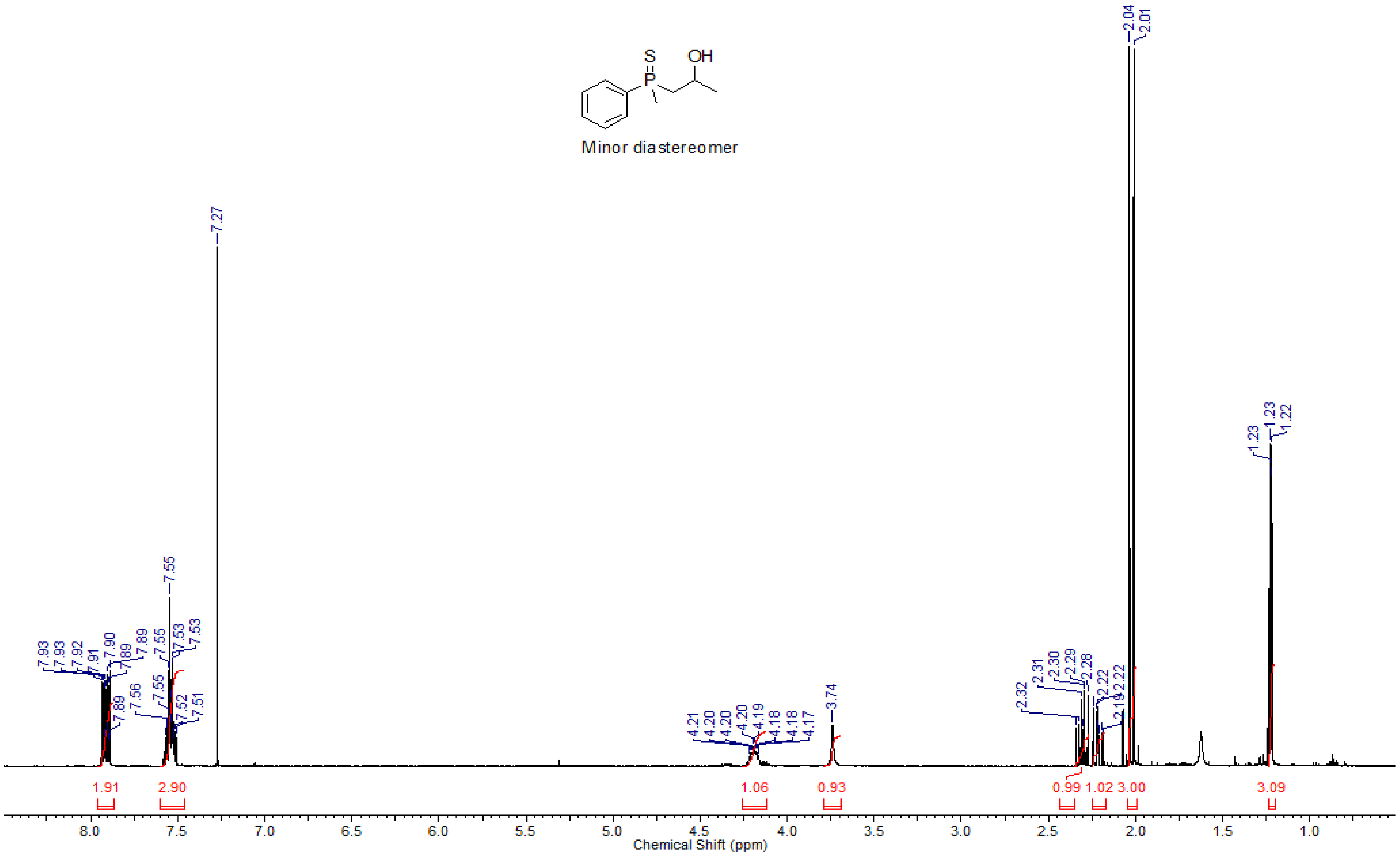

^{1}H NMR spectrum of (2-hydroxypropyl)methylphenylphosphine sulfide (minor diastereomer) (**6**) (CDCl3, 500 MHz).

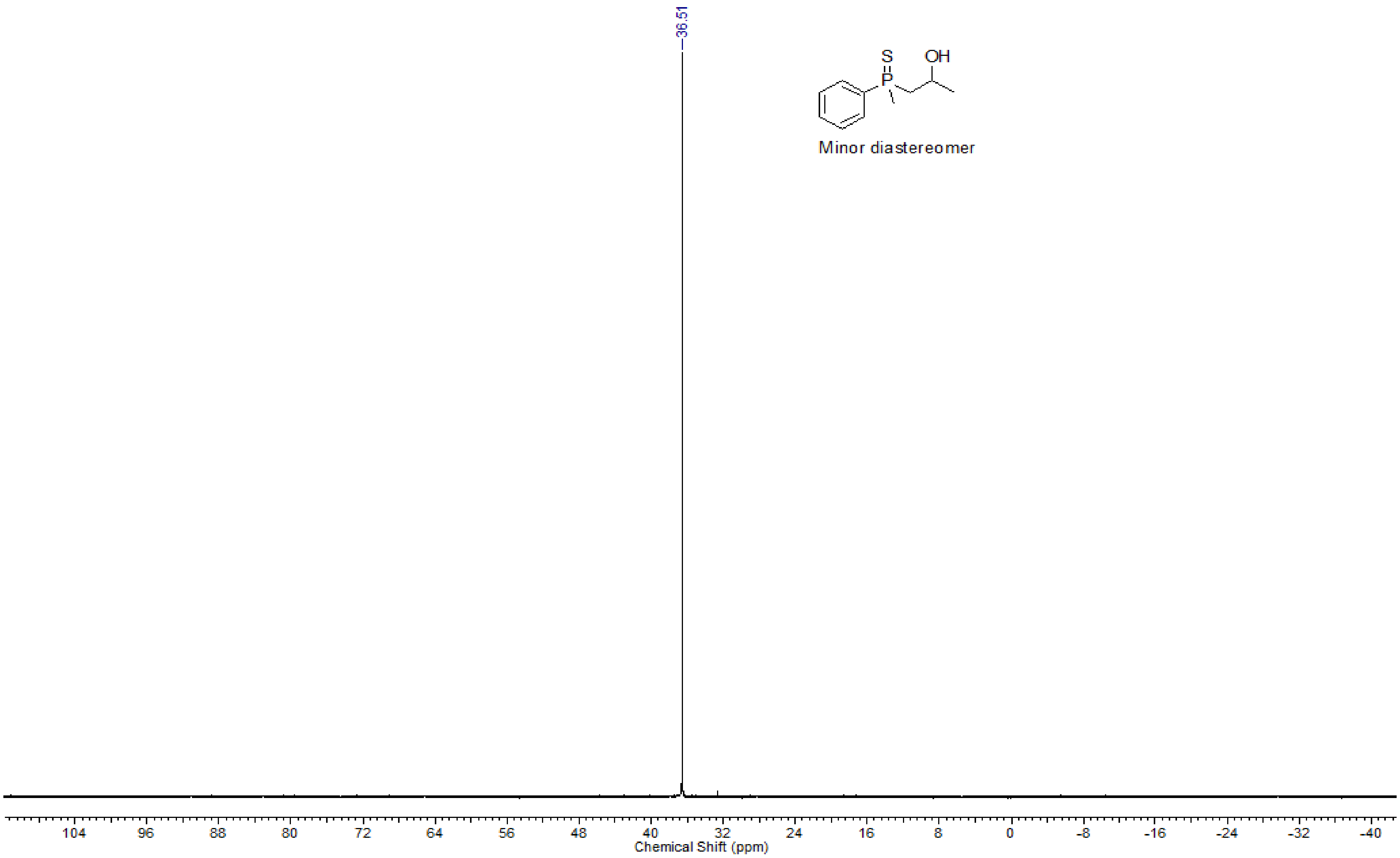

^{31}P NMR spectrum of (2-hydroxypropyl)methylphenylphosphine sulfide (minor diastereomer) (**6**) (CDCl3, 202 MHz).

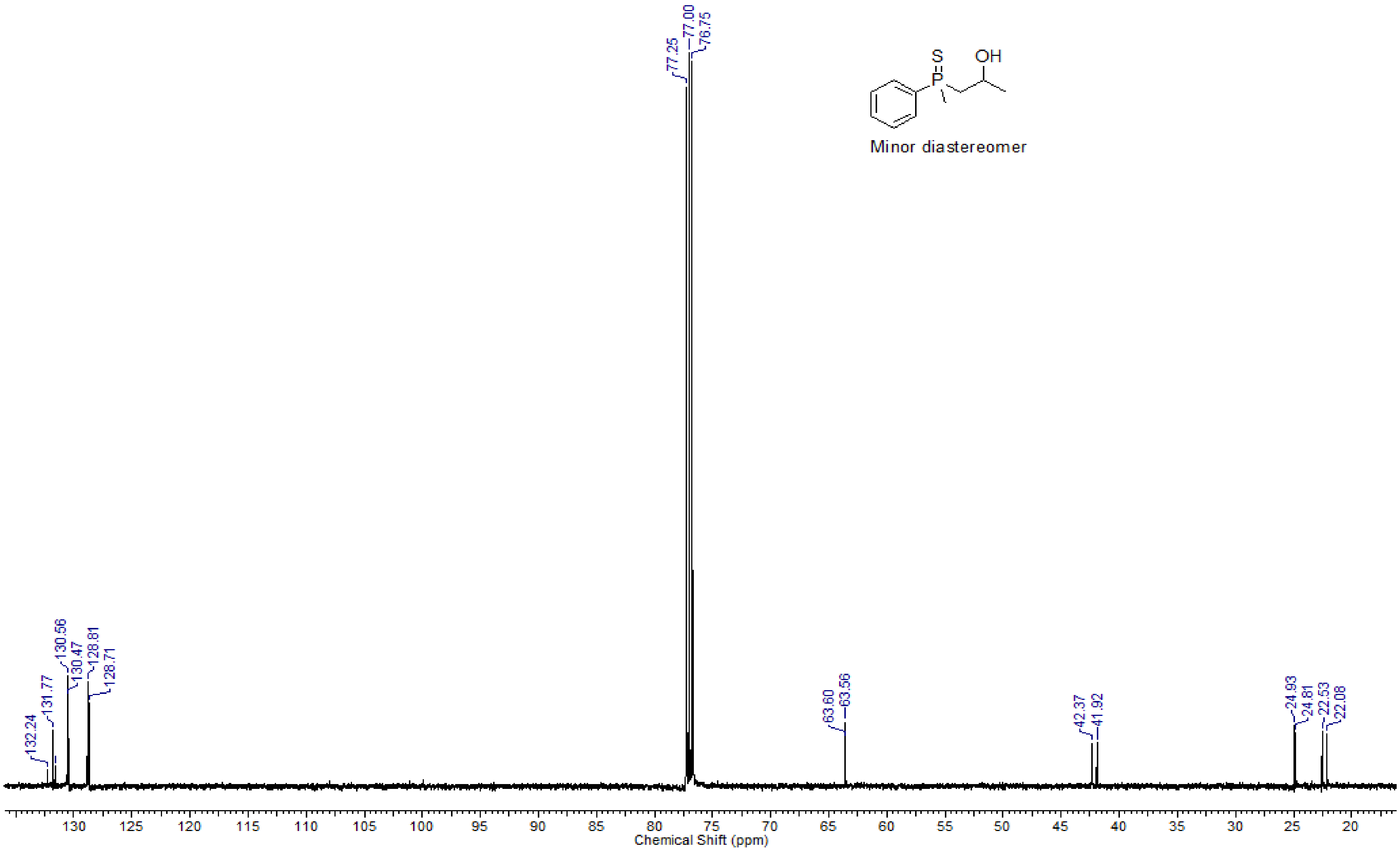

^{13}C NMR spectrum of (2-hydroxypropyl)methylphenylphosphine sulfide (minor diastereomer) (**6**) (CDCl3, 126 MHz).

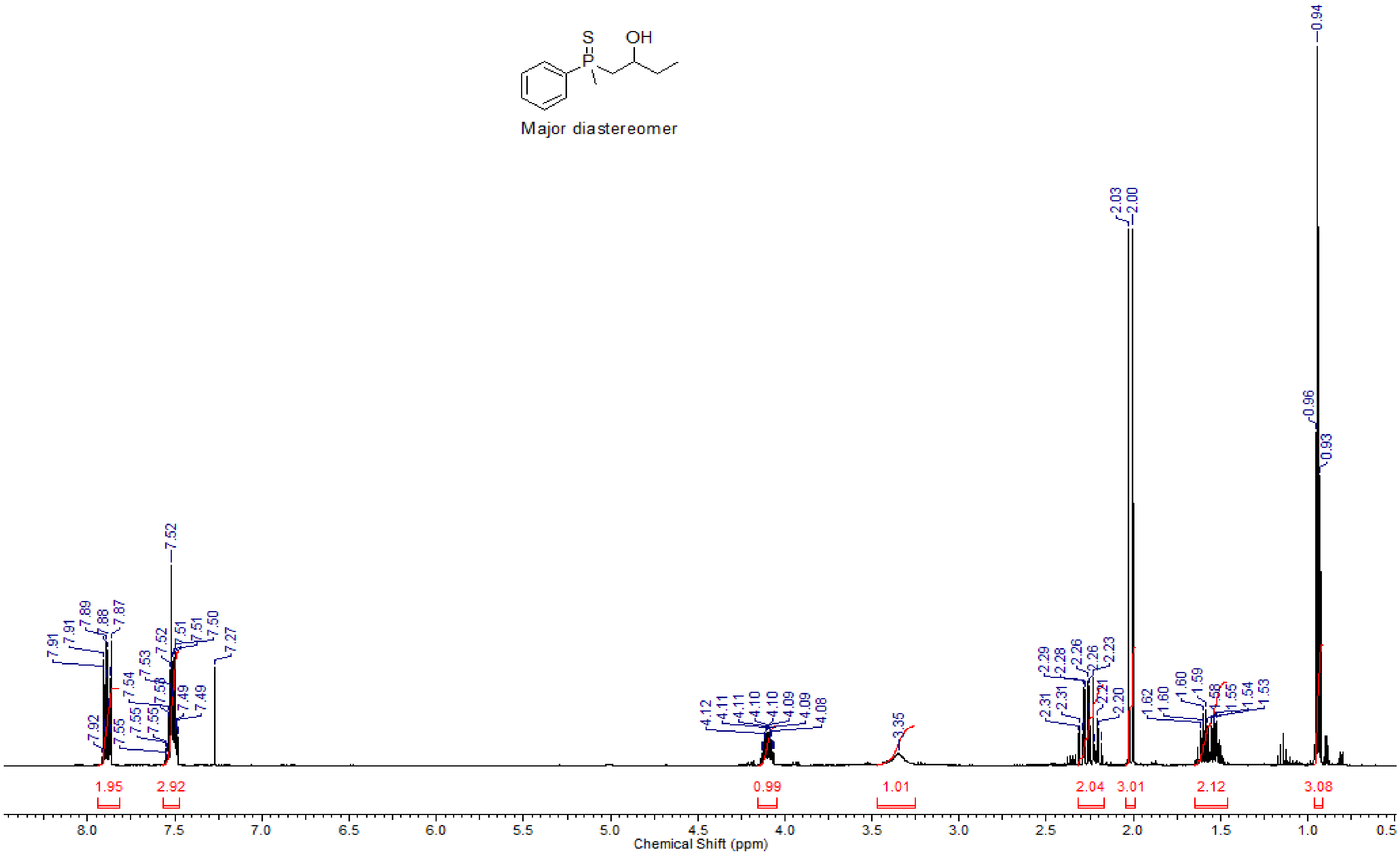

^{1}H NMR spectrum of (2-hydroxybutyl)methylphenylphosphine sulfide (major diastereomer) (**7**) (CDCl3, 500 MHz).

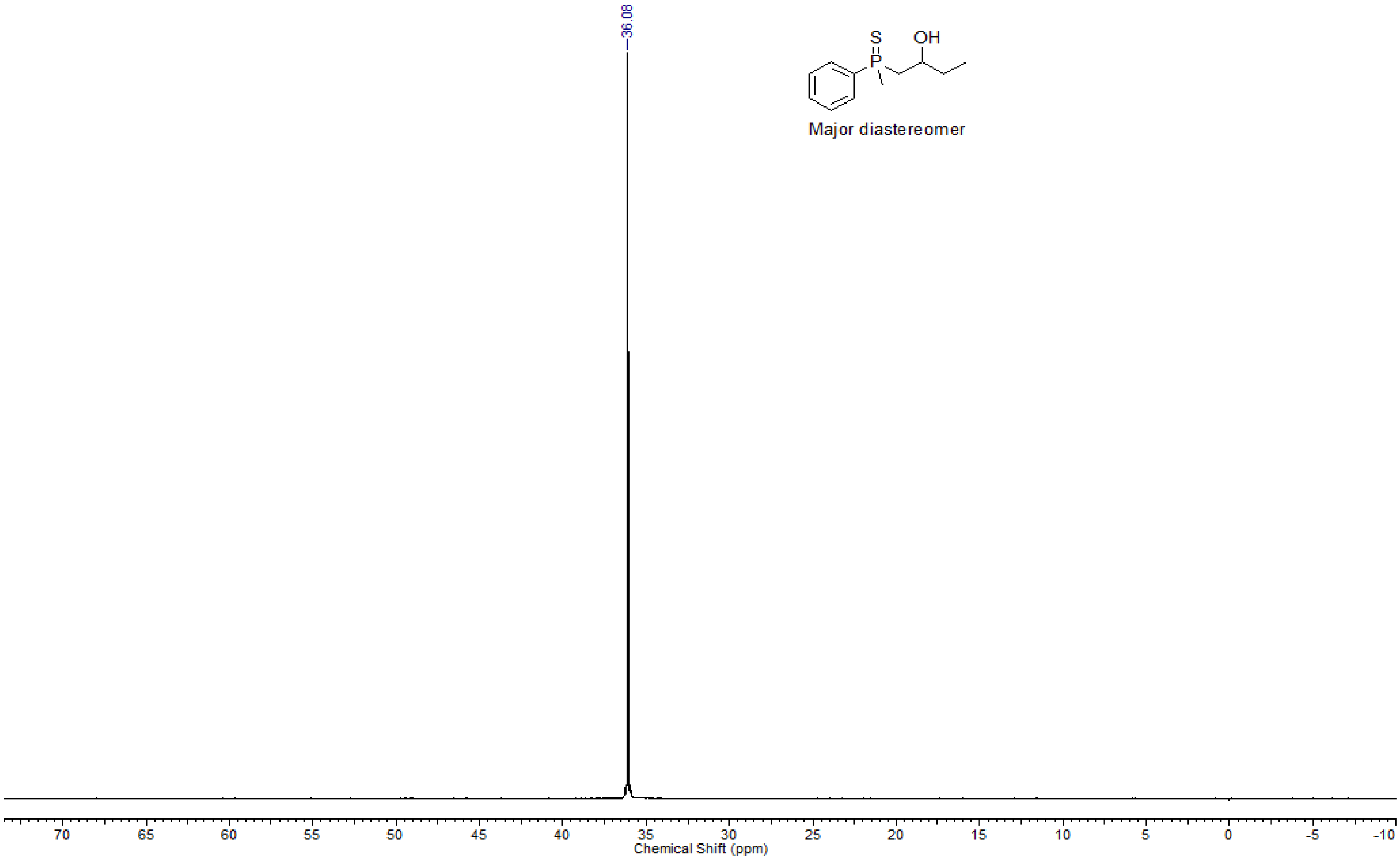

^{31}P NMR spectrum of (2-hydroxybutyl)methylphenylphosphine sulfide (major diastereomer) (**7**) (CDCl3, 202 MHz).

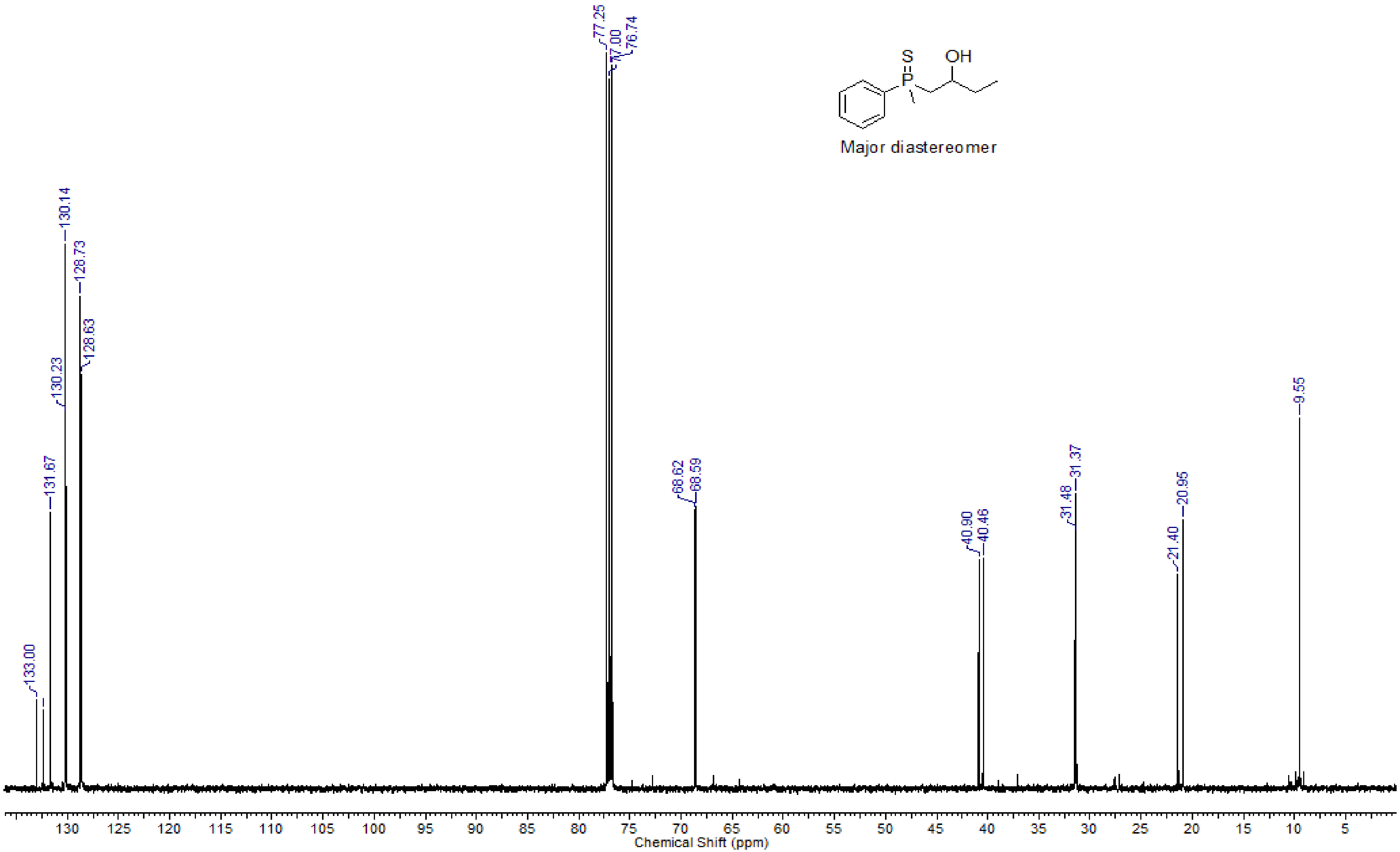

^{13}C NMR spectrum of (2-hydroxybutyl)methylphenylphosphine sulfide (major diastereomer) (**7**) (CDCl3, 126 MHz).

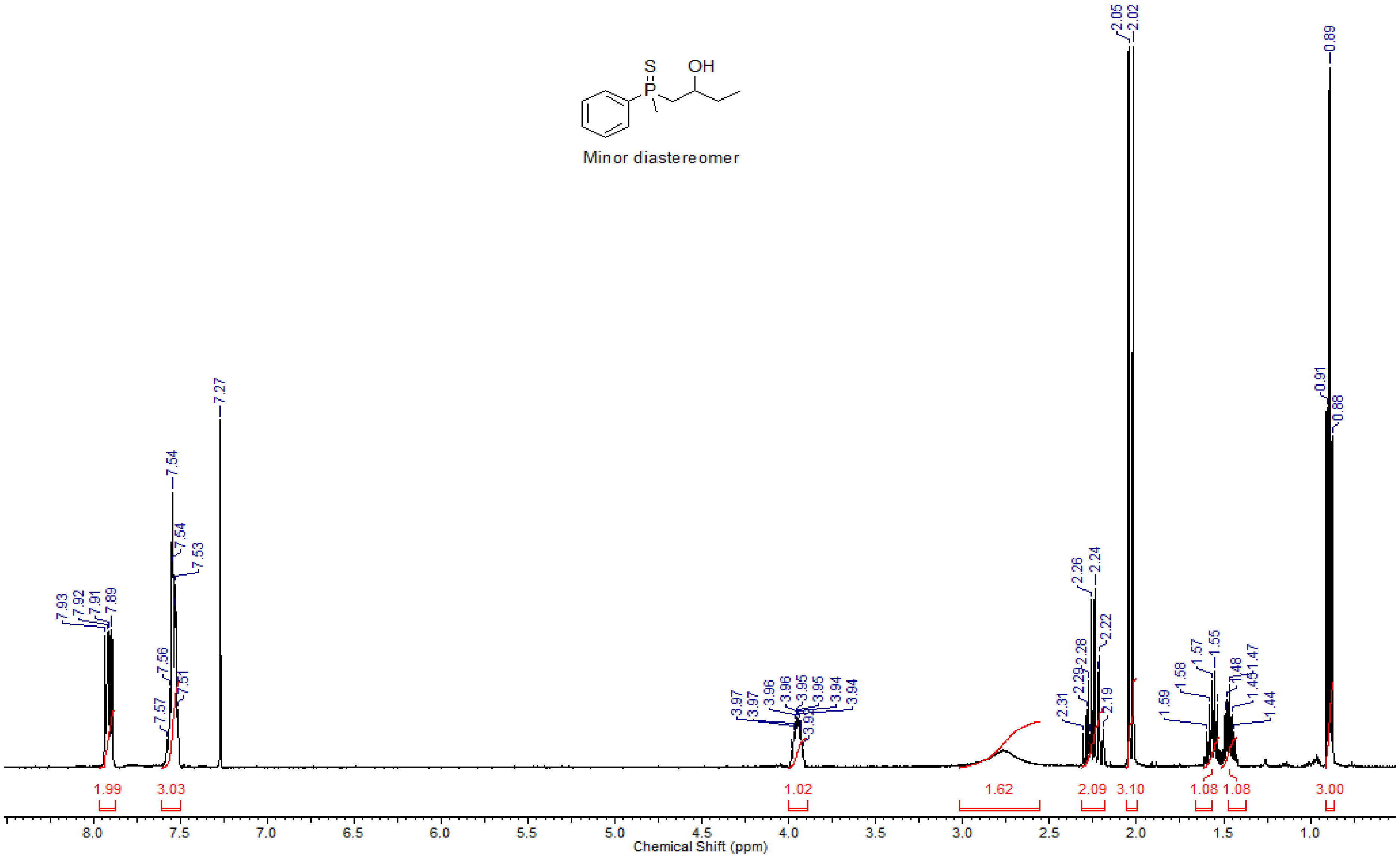

^{1}H NMR spectrum of (2-hydroxybutyl)methylphenylphosphine sulfide (minor diastereomer) (**7**) ($CDCl_3$, 500 MHz).

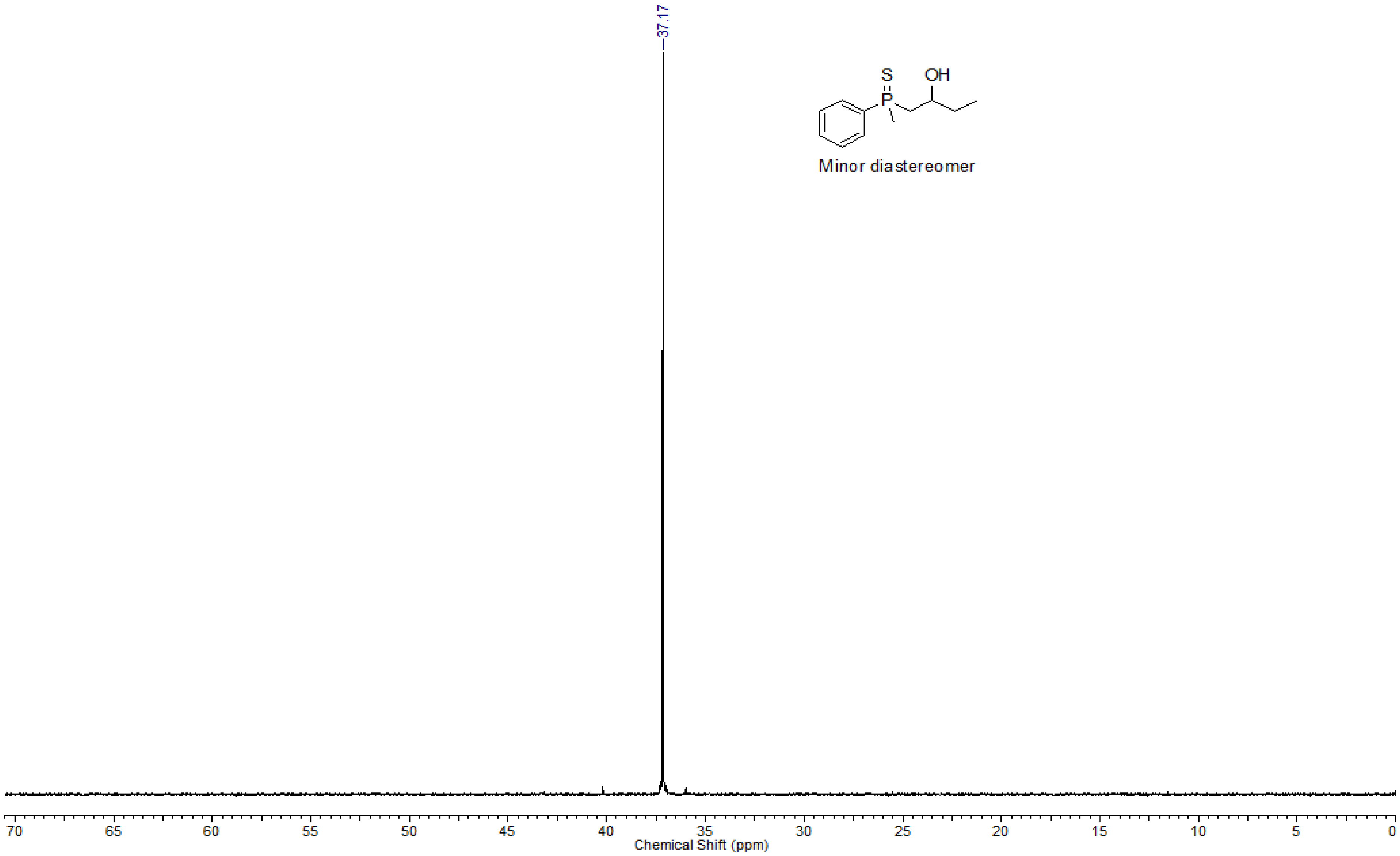

^{31}P NMR spectrum of (2-hydroxybutyl)methylphenylphosphine sulfide (minor diastereomer) (**7**) ($CDCl_3$, 202 MHz).

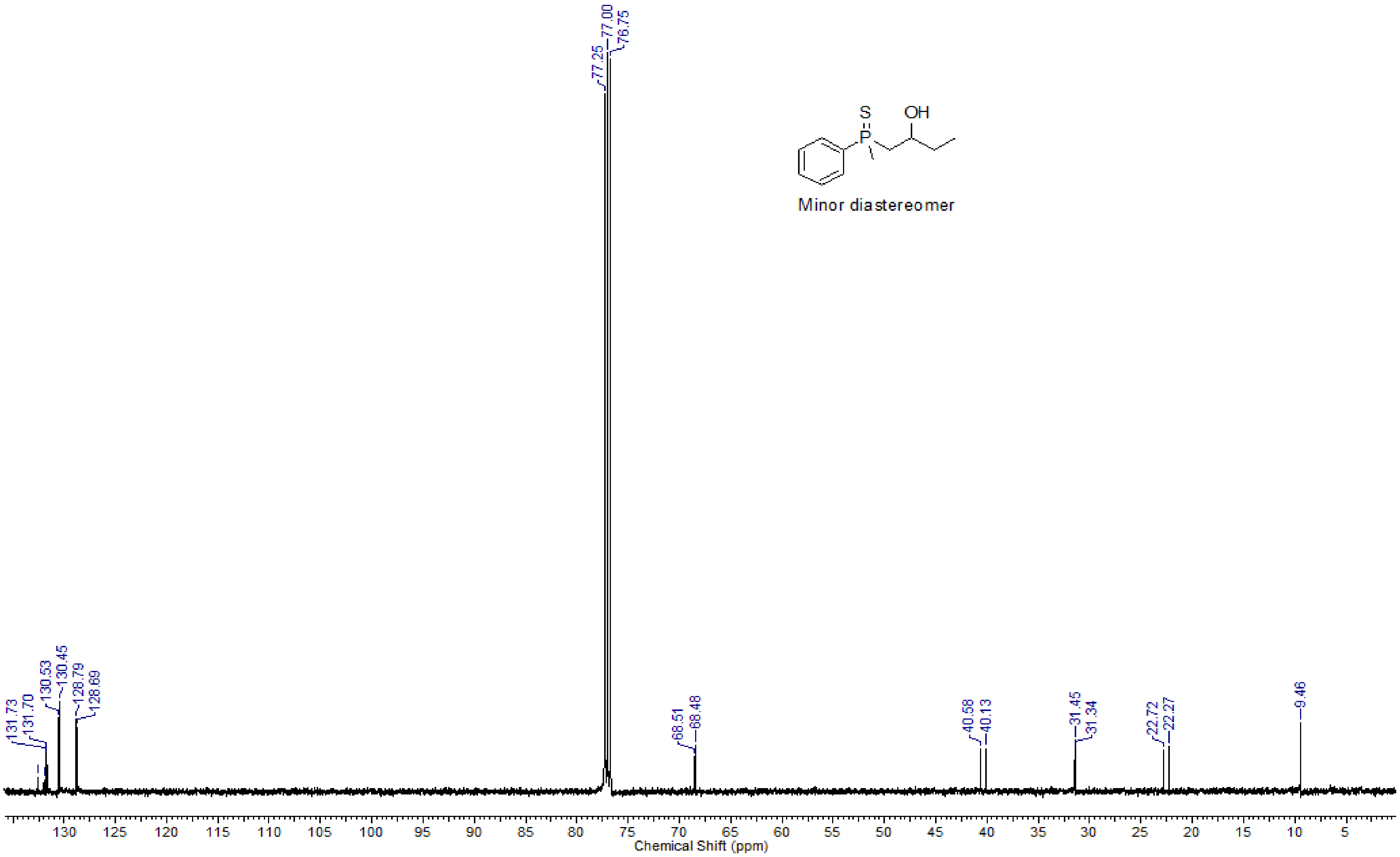

^{13}C NMR spectrum of (2-hydroxybutyl)methylphenylphosphine sulfide (minor diastereomer) (**7**) (CDCl3, 126 MHz).

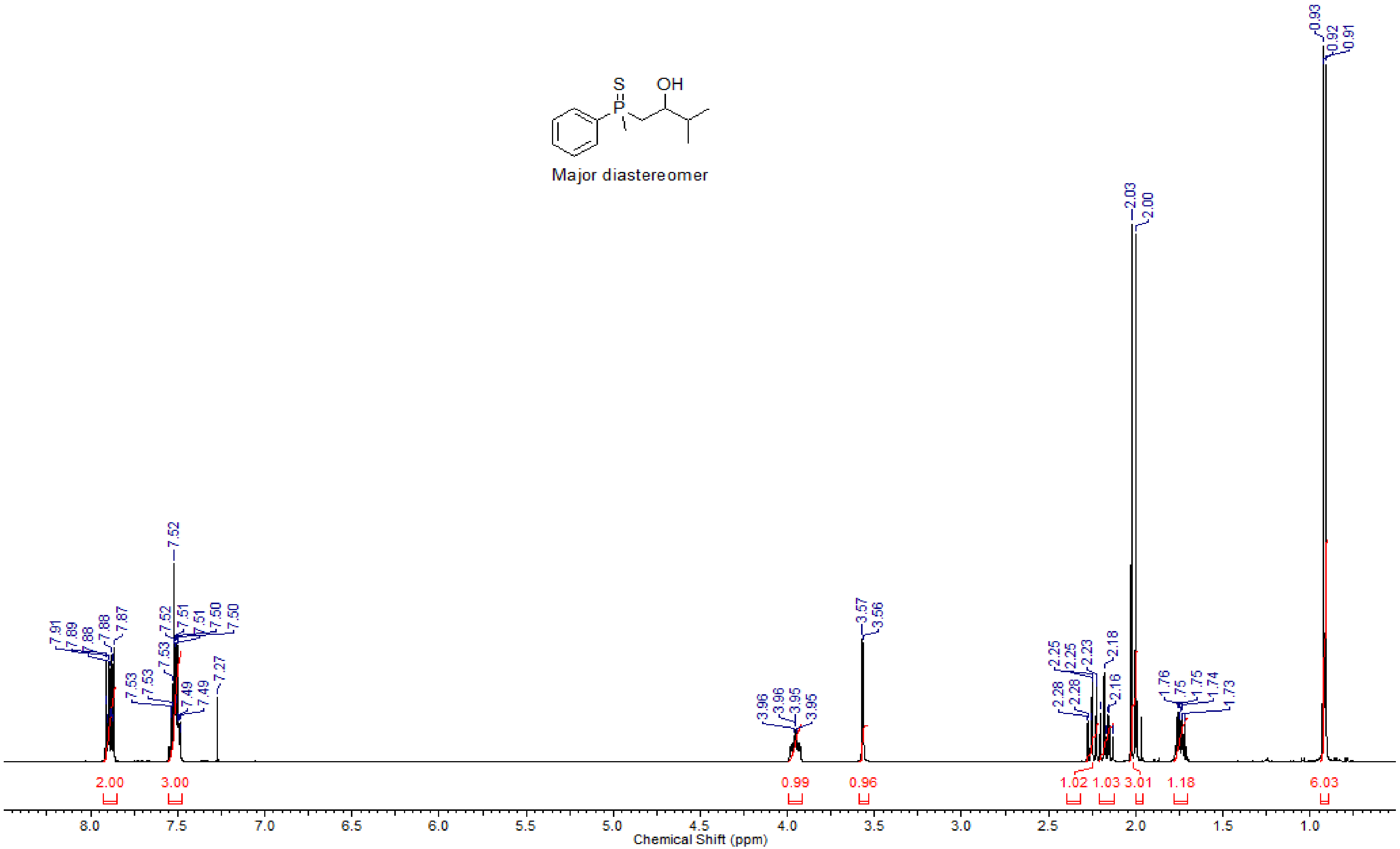

^{1}H NMR spectrum of (2-hydroxy-3-methylbutyl)methylphenylphosphine sulfide (major diastereomer) (**8**) (CDCl3, 500 MHz).

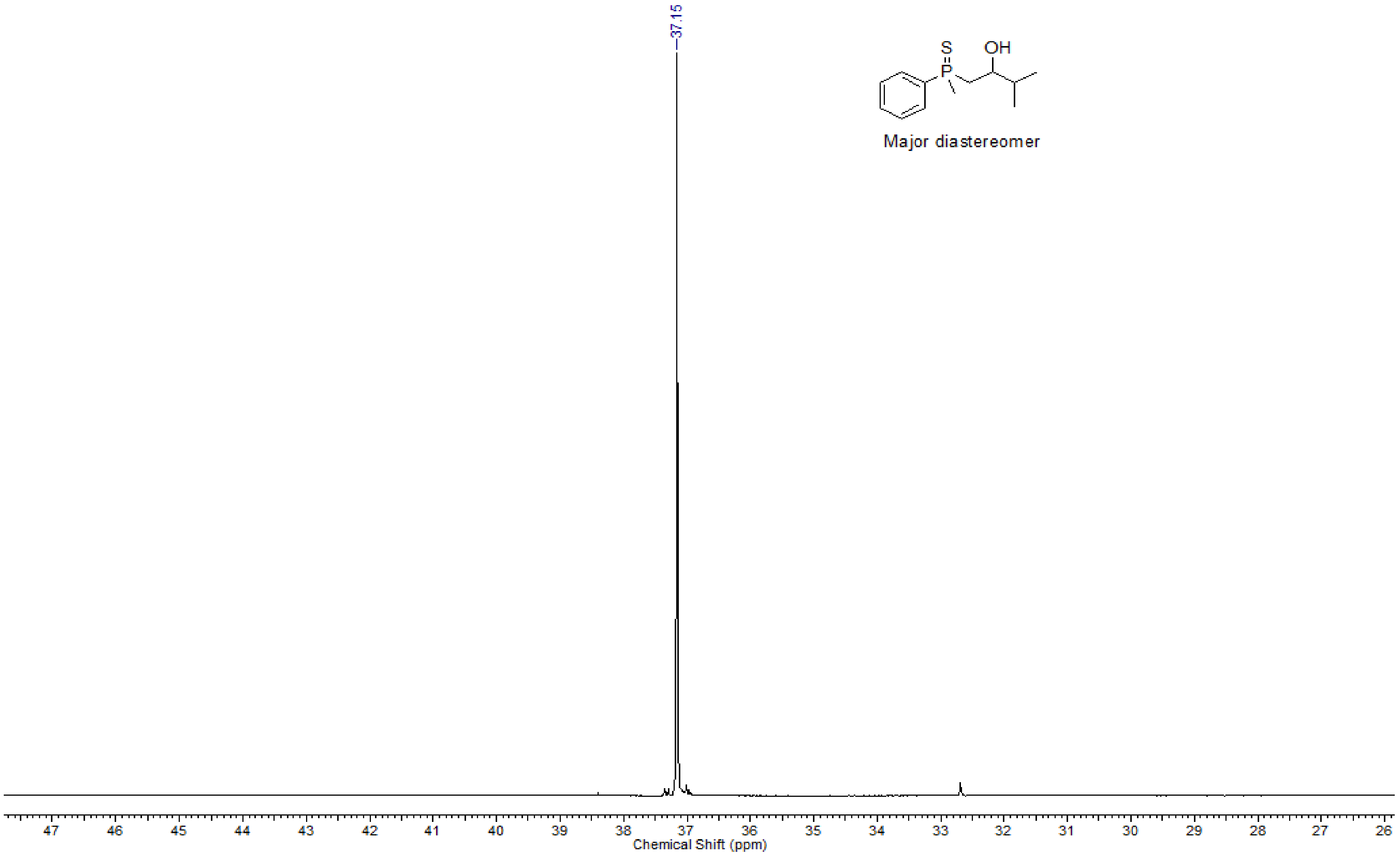

^{31}P NMR spectrum of (2-hydroxy-3-methylbutyl)methylphenylphosphine sulfide (major diastereomer) (**8**) (CDCl3, 202 MHz).

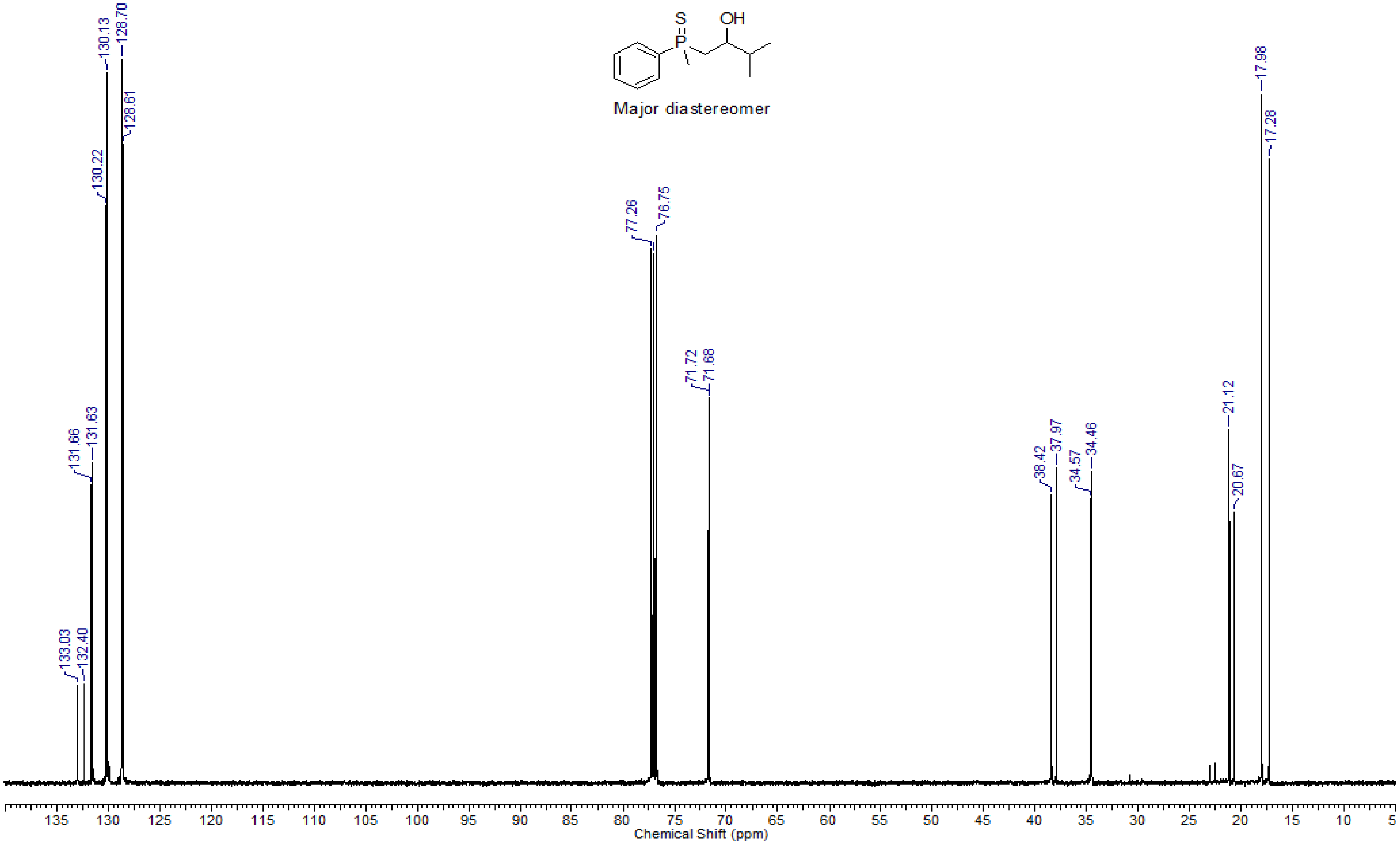

^{13}C NMR spectrum of (2-hydroxy-3-methylbutyl)methylphenylphosphine sulfide (major diastereomer) (**8**) (CDCl3, 126 MHz).

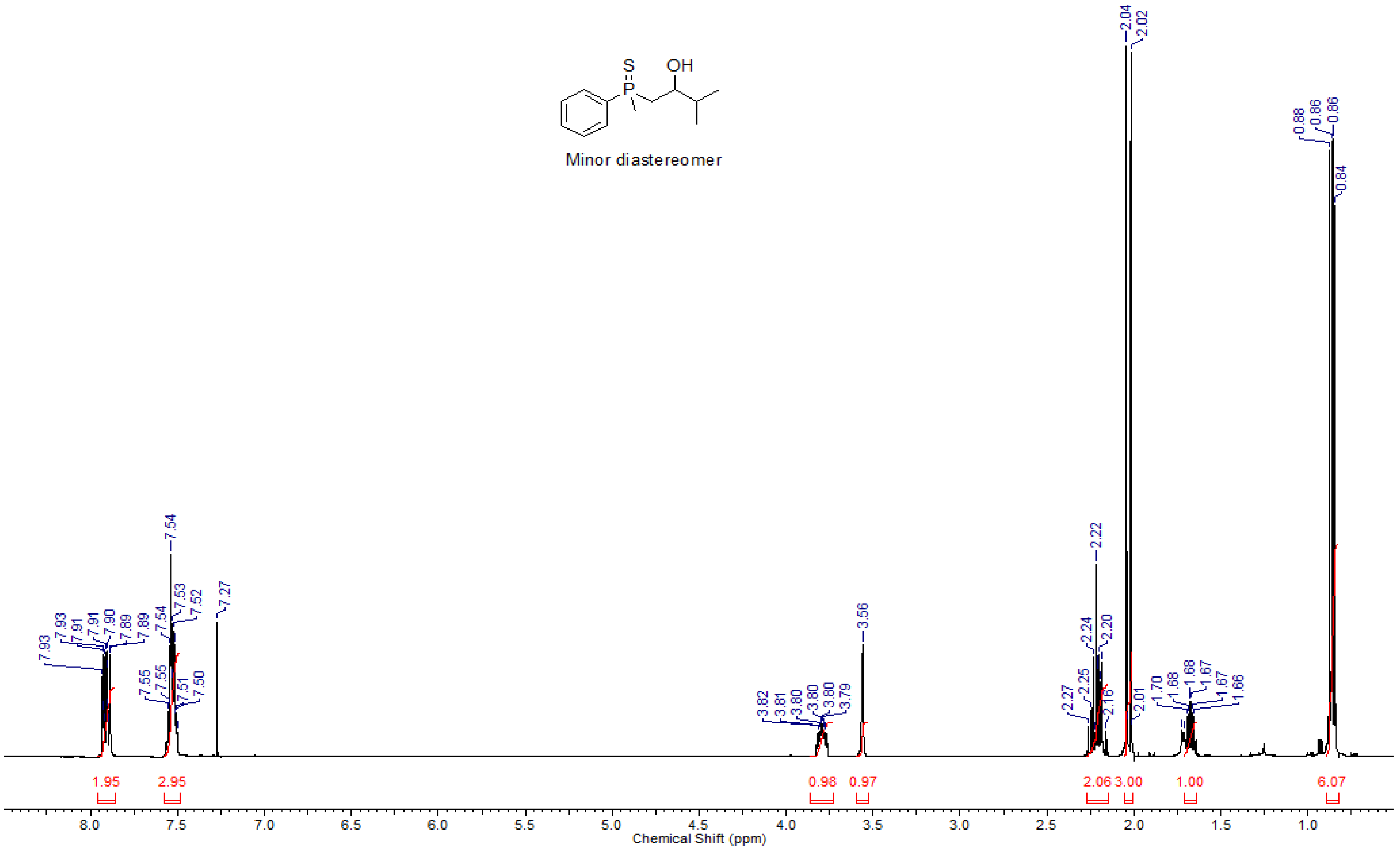

^{1}H NMR spectrum of (2-hydroxy-3-methylbutyl)methylphenylphosphine sulfide (minor diastereomer) (**8**) (CDCl3, 500 MHz).

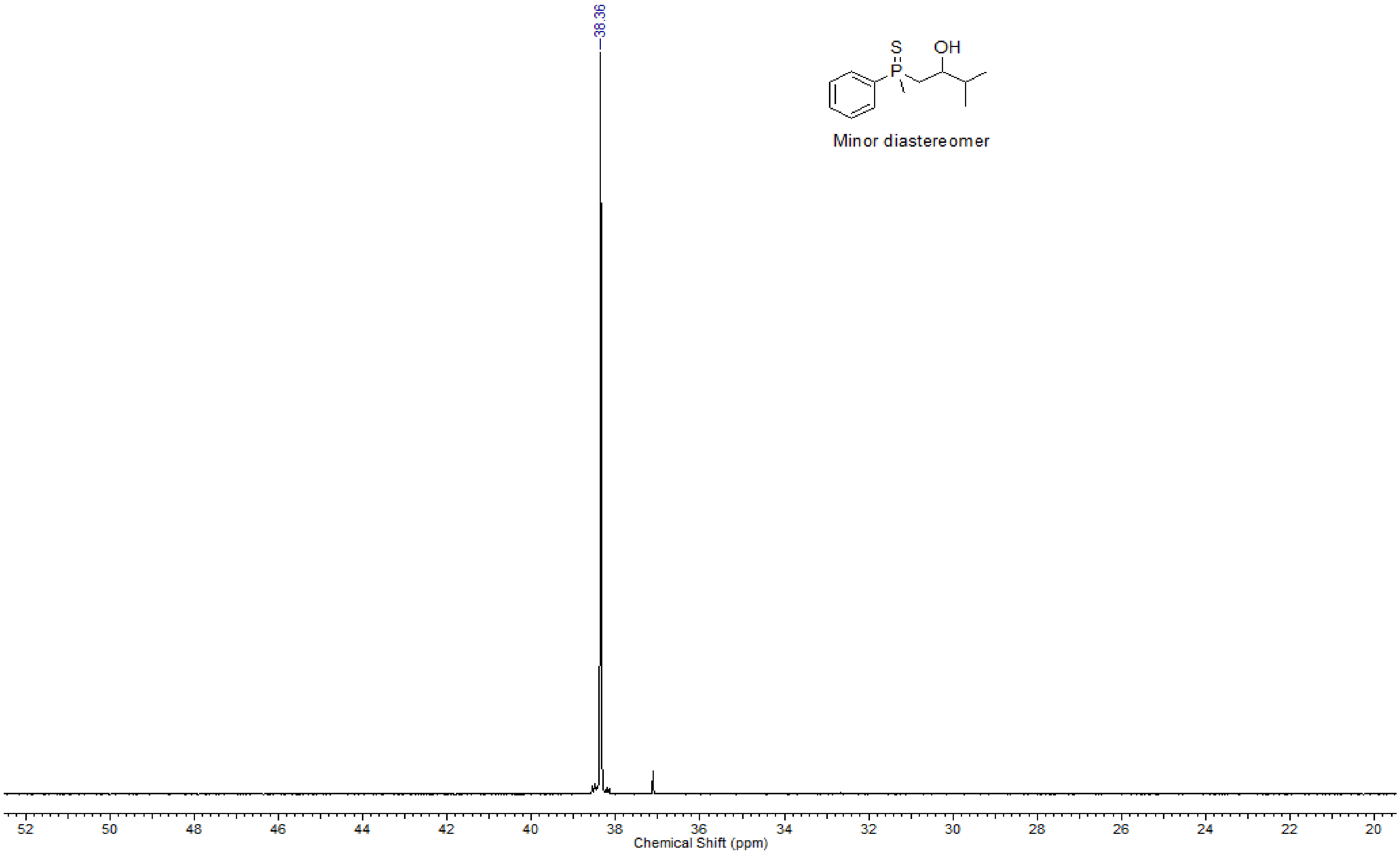

^{31}P NMR spectrum of (2-hydroxy-3-methylbutyl)methylphenylphosphine sulfide (minor diastereomer) (**8**) (CDCl3, 202 MHz).

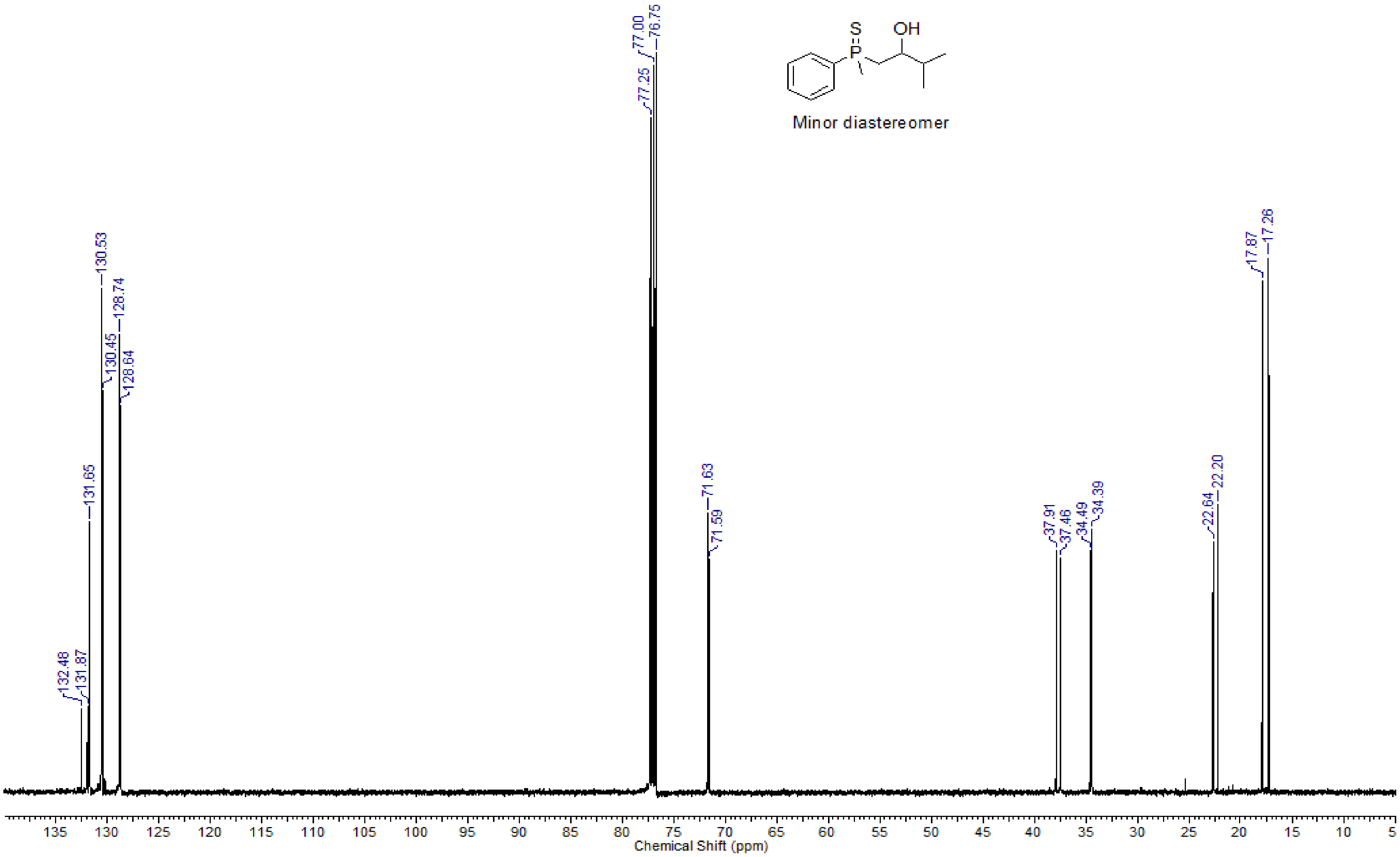

^{13}C NMR spectrum of (2-hydroxy-3-methylbutyl)methylphenylphosphine sulfide (minor diastereomer) (**8**) (CDCl3, 126 MHz).

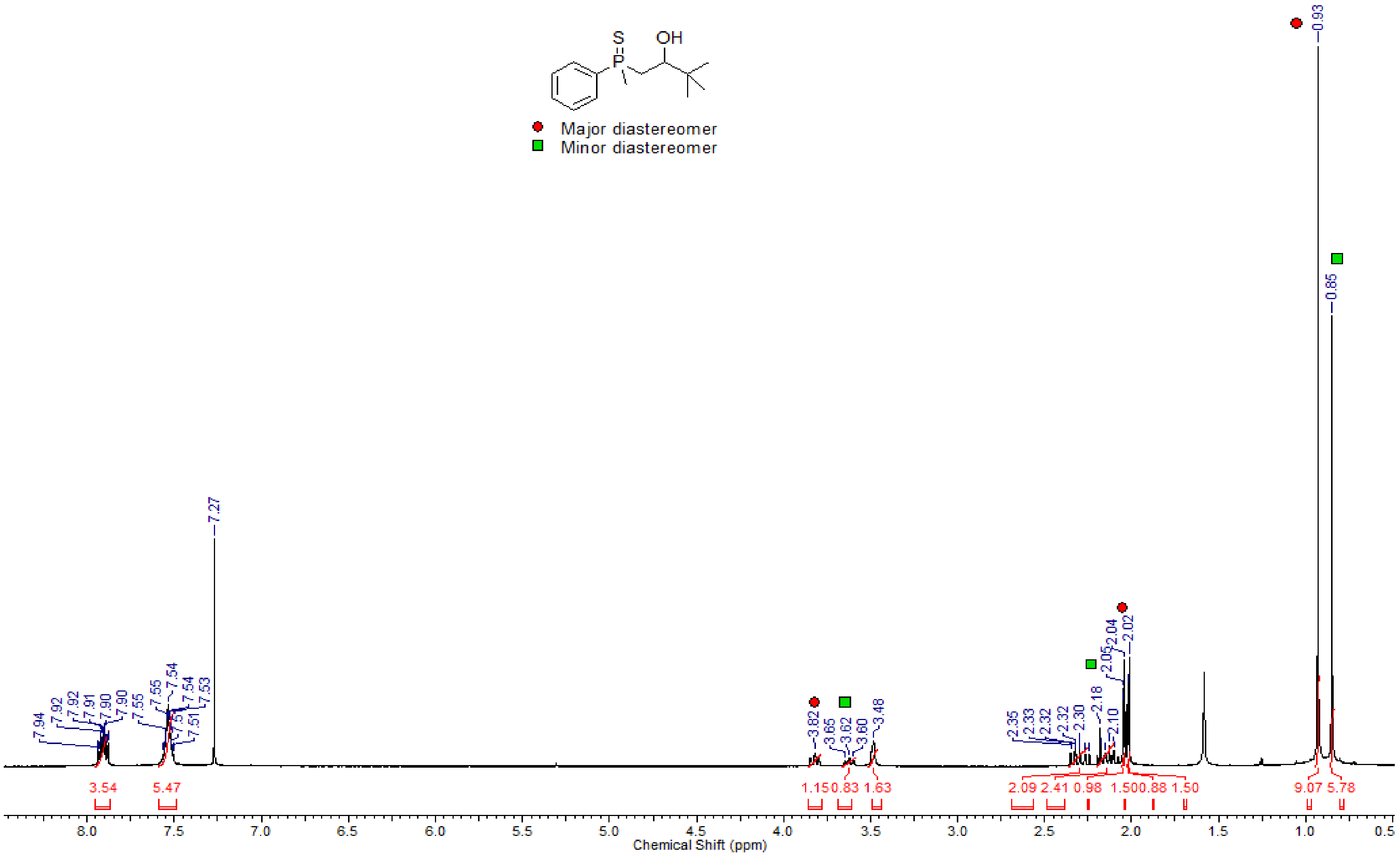

^{1}H NMR spectrum of (2-hydroxy-3,3-dimethylbutyl)methylphenylphosphine sulfide (**9**) (CDCl3, 500 MHz).

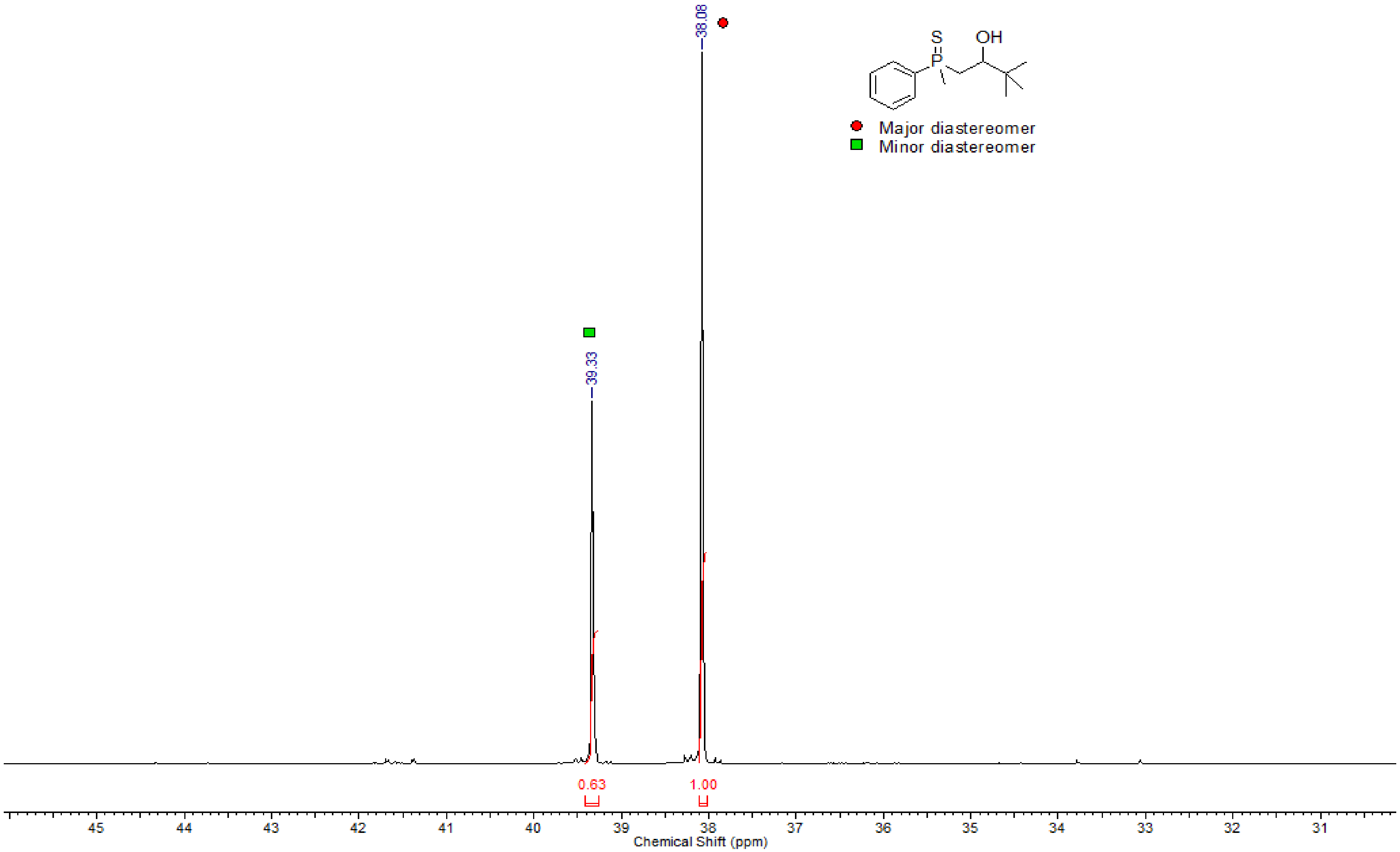

^{31}P NMR spectrum of (2-hydroxy-3,3-dimethylbutyl)methylphenylphosphine sulfide (**9**) (CDCl3, 202 MHz).

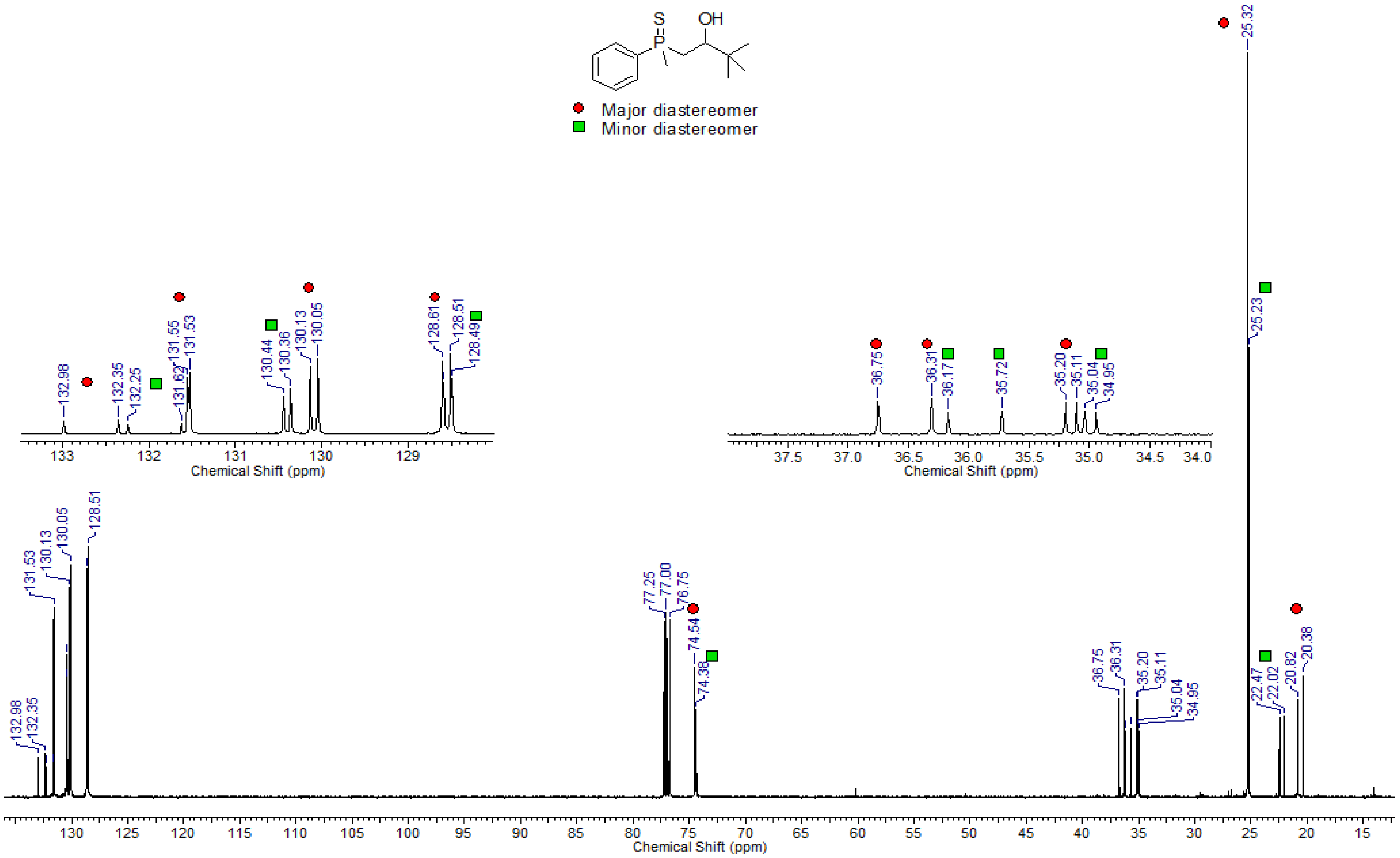

^{13}C NMR spectrum of (2-hydroxy-3,3-dimethylbutyl)methylphenylphosphine sulfide (**9**) (CDCl3, 126 MHz).

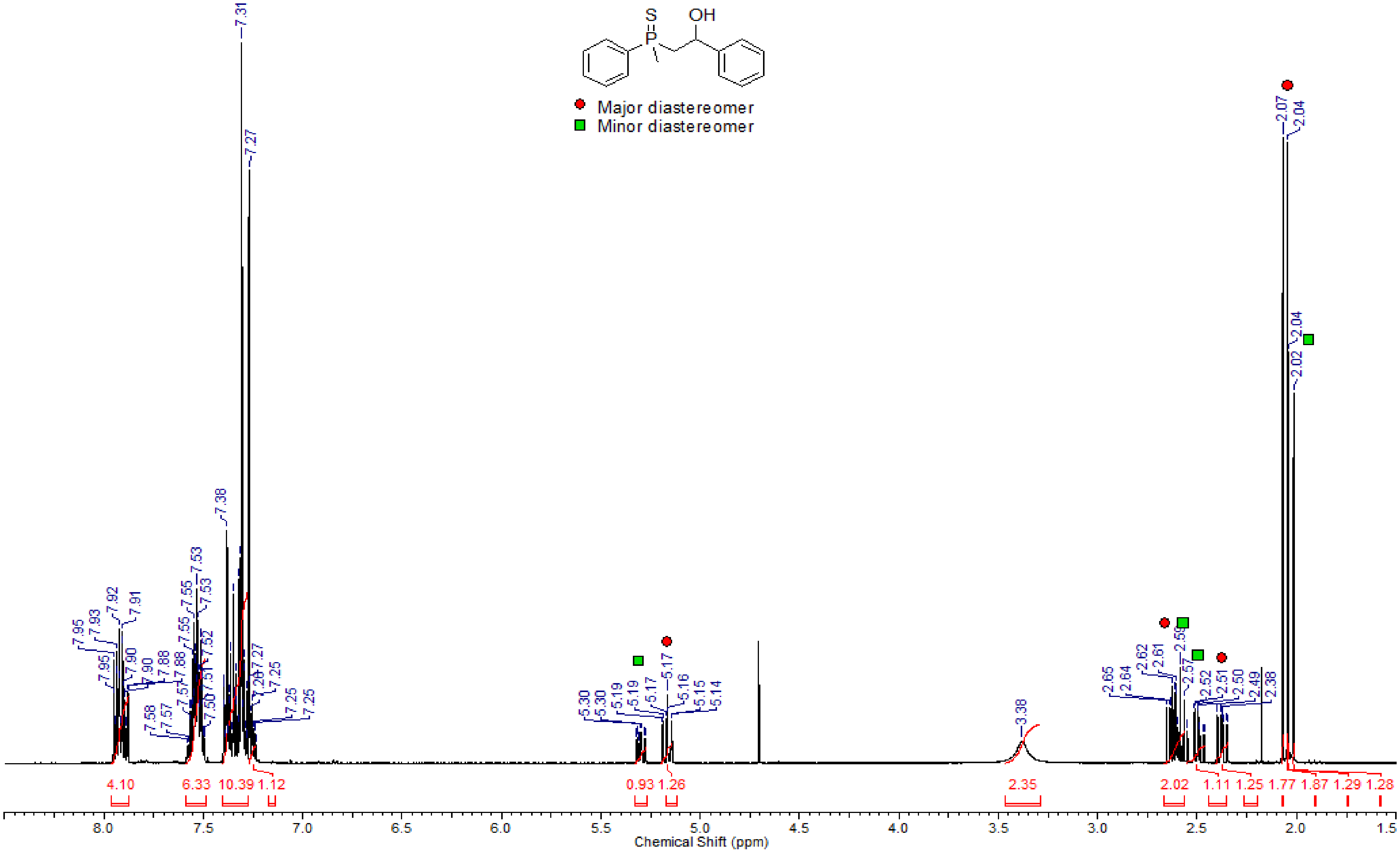

^{1}H NMR spectrum of (2-hydroxy-2-phenylethyl)methylphenylphosphine sulfide (**10**) (CDCl3, 500 MHz).

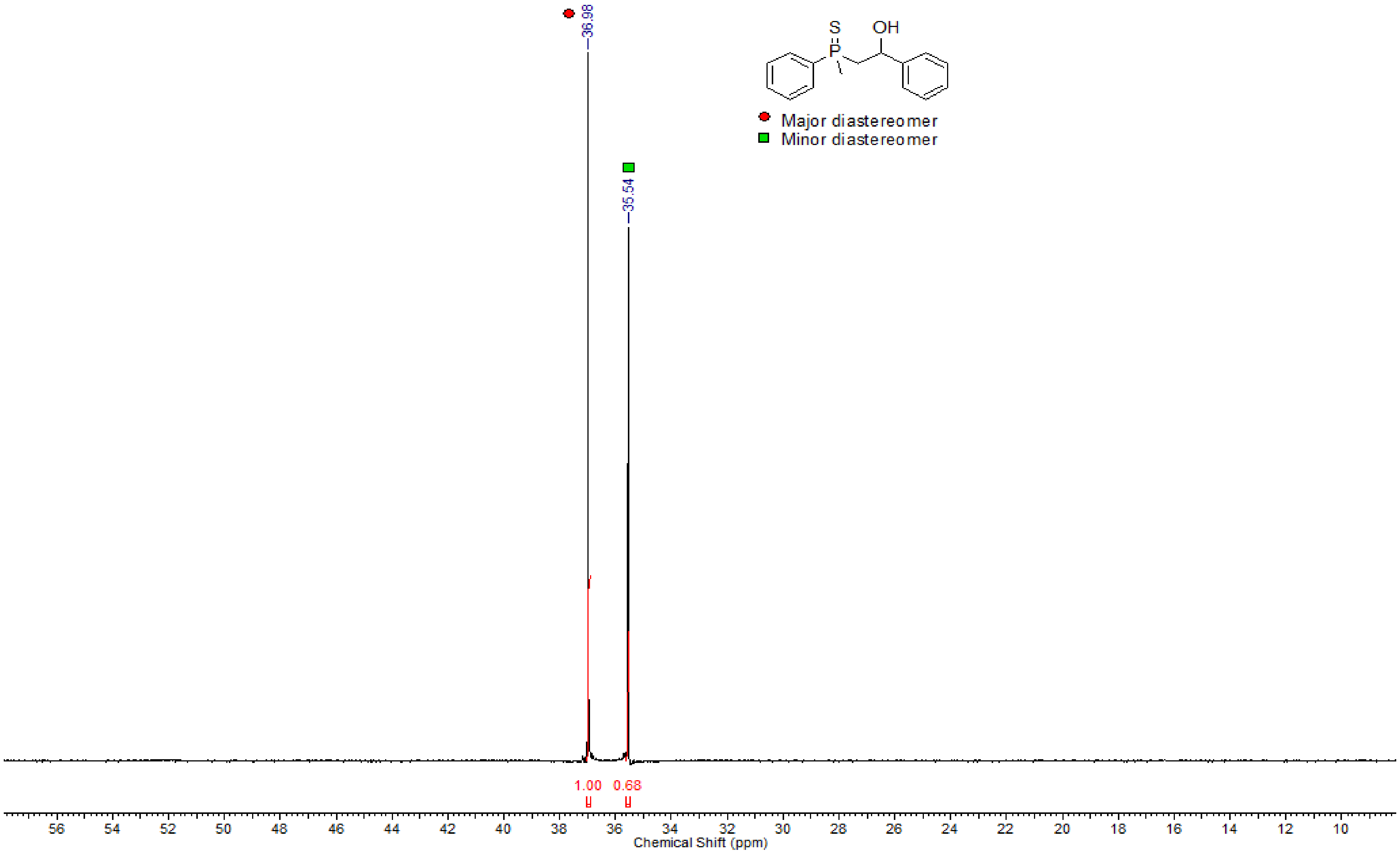

^{31}P NMR spectrum of (2-hydroxy-2-phenylethyl)methylphenylphosphine sulfide (**10**) (CDCl3, 202 MHz).

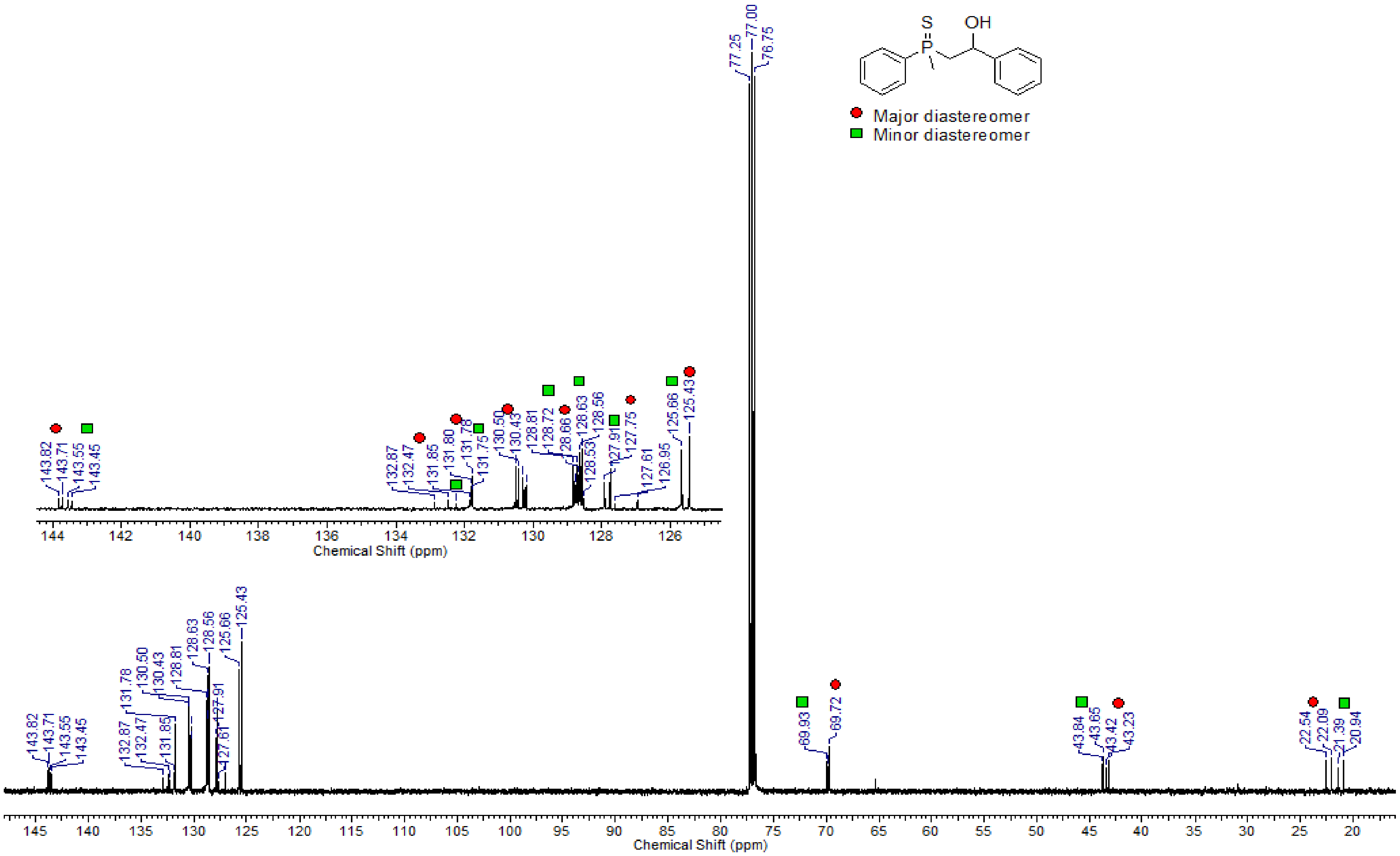

^{13}C NMR spectrum of (2-hydroxy-2-phenylethyl)methylphenylphosphine sulfide (**10**) (CDCl3, 126 MHz).

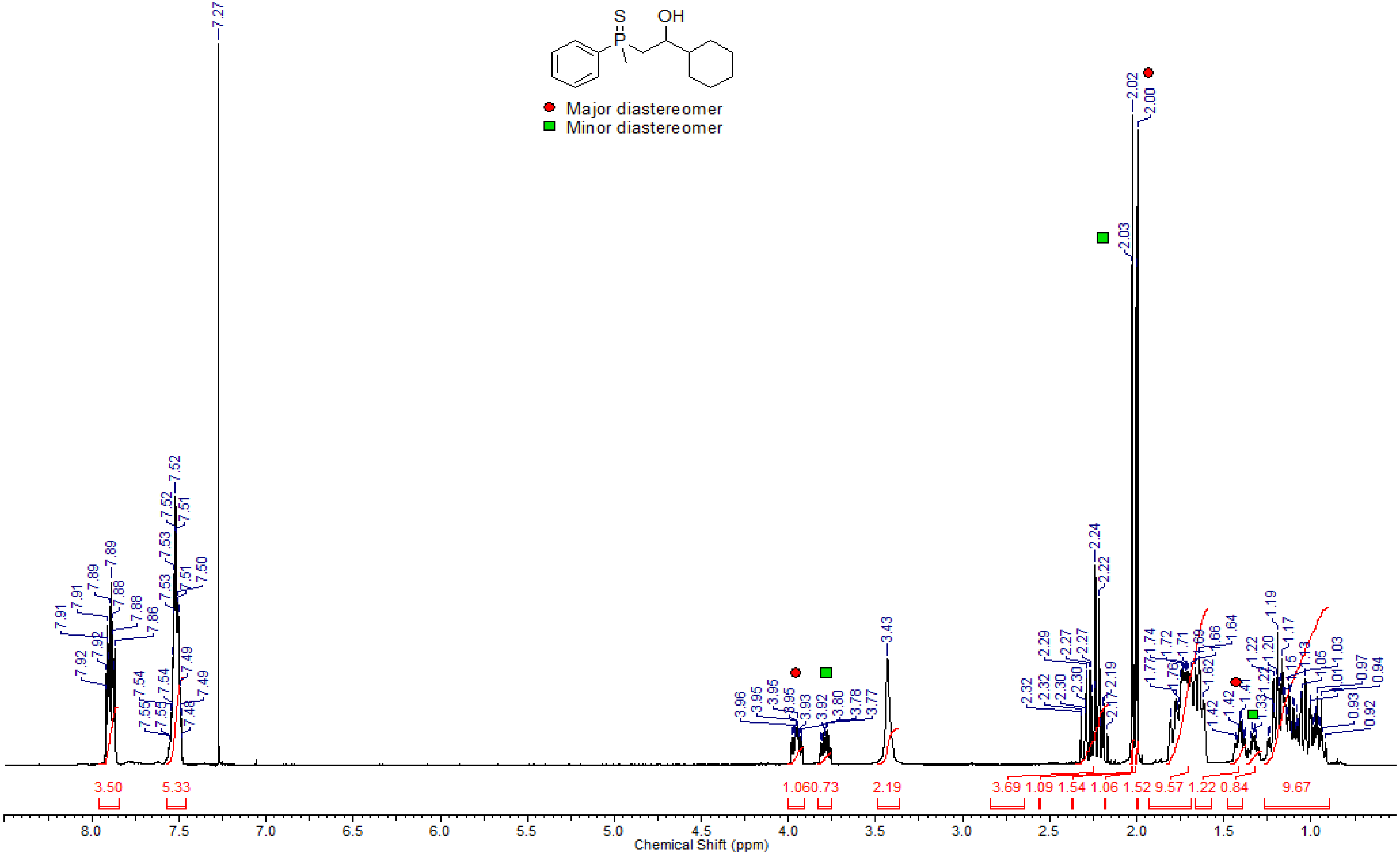

^{1}H NMR spectrum of (2-cyclohexyl-2-hydroxyethyl)methylphenylphosphine sulfide (**11**) (CDCl3, 500 MHz).

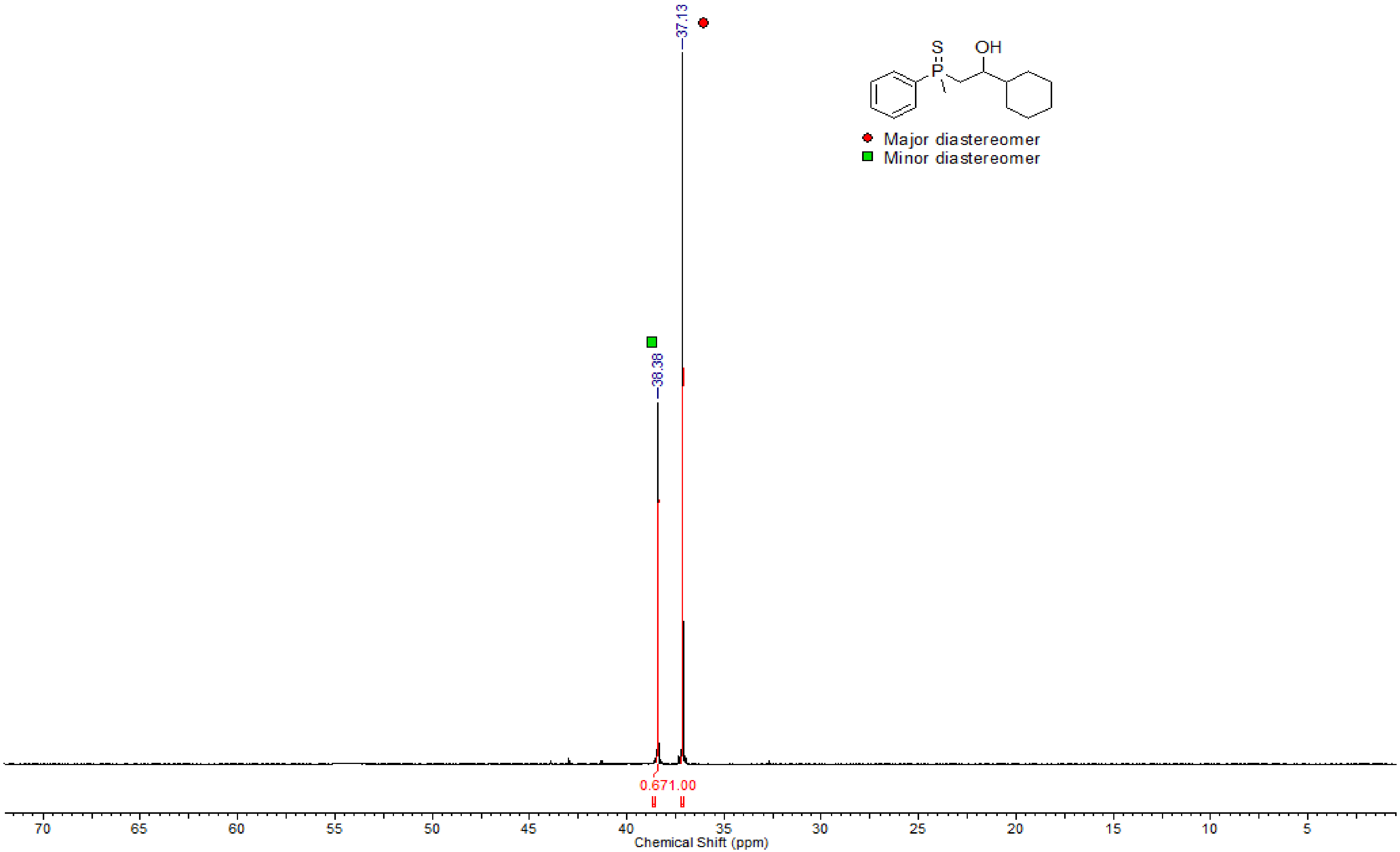

^{31}P NMR spectrum of (2-cyclohexyl-2-hydroxyethyl)methylphenylphosphine sulfide (**11**) (CDCl3, 202 MHz).

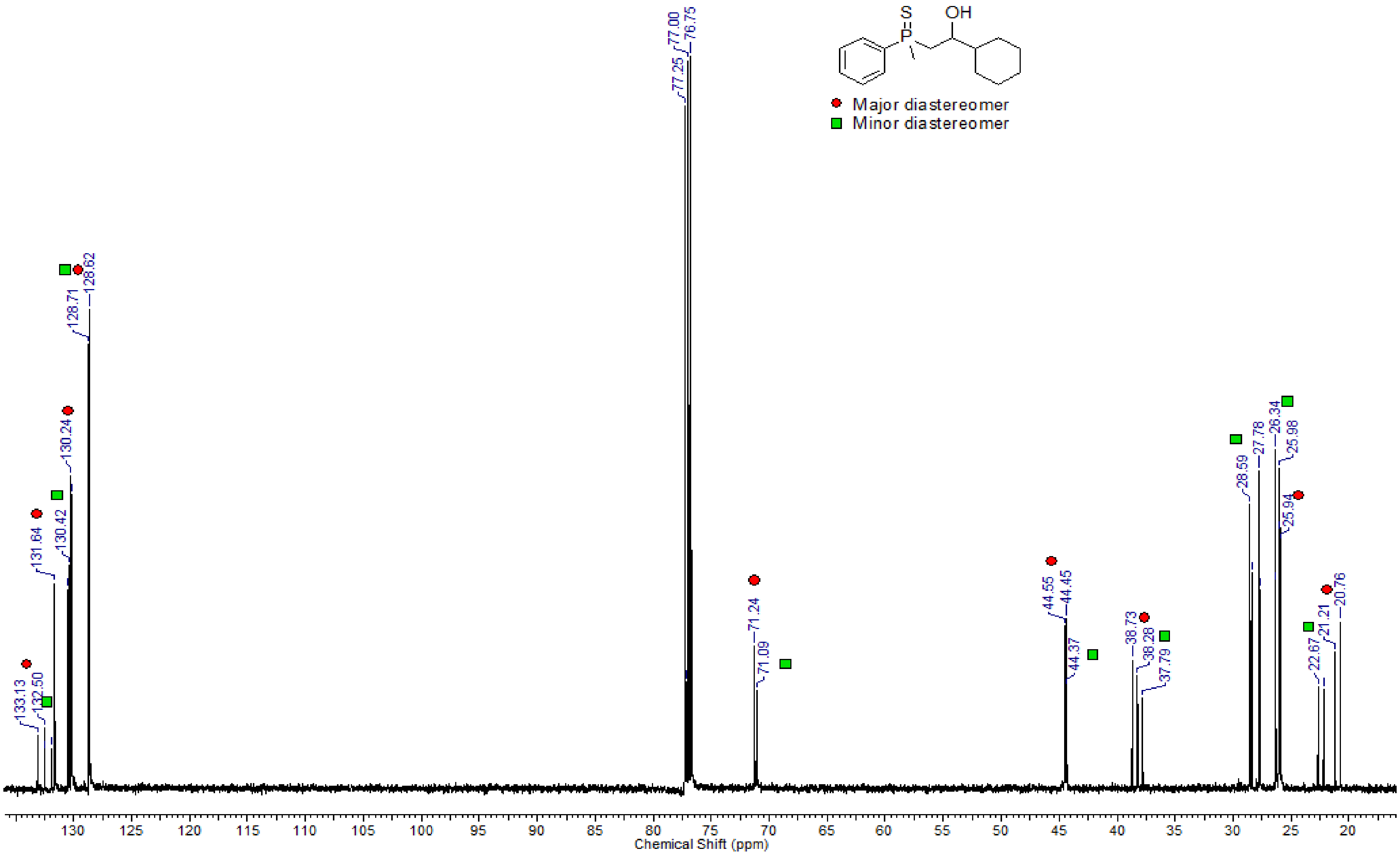

^{13}C NMR spectrum of (2-cyclohexyl-2-hydroxyethyl)methylphenylphosphine sulfide (**11**) (CDCl3, 126 MHz).

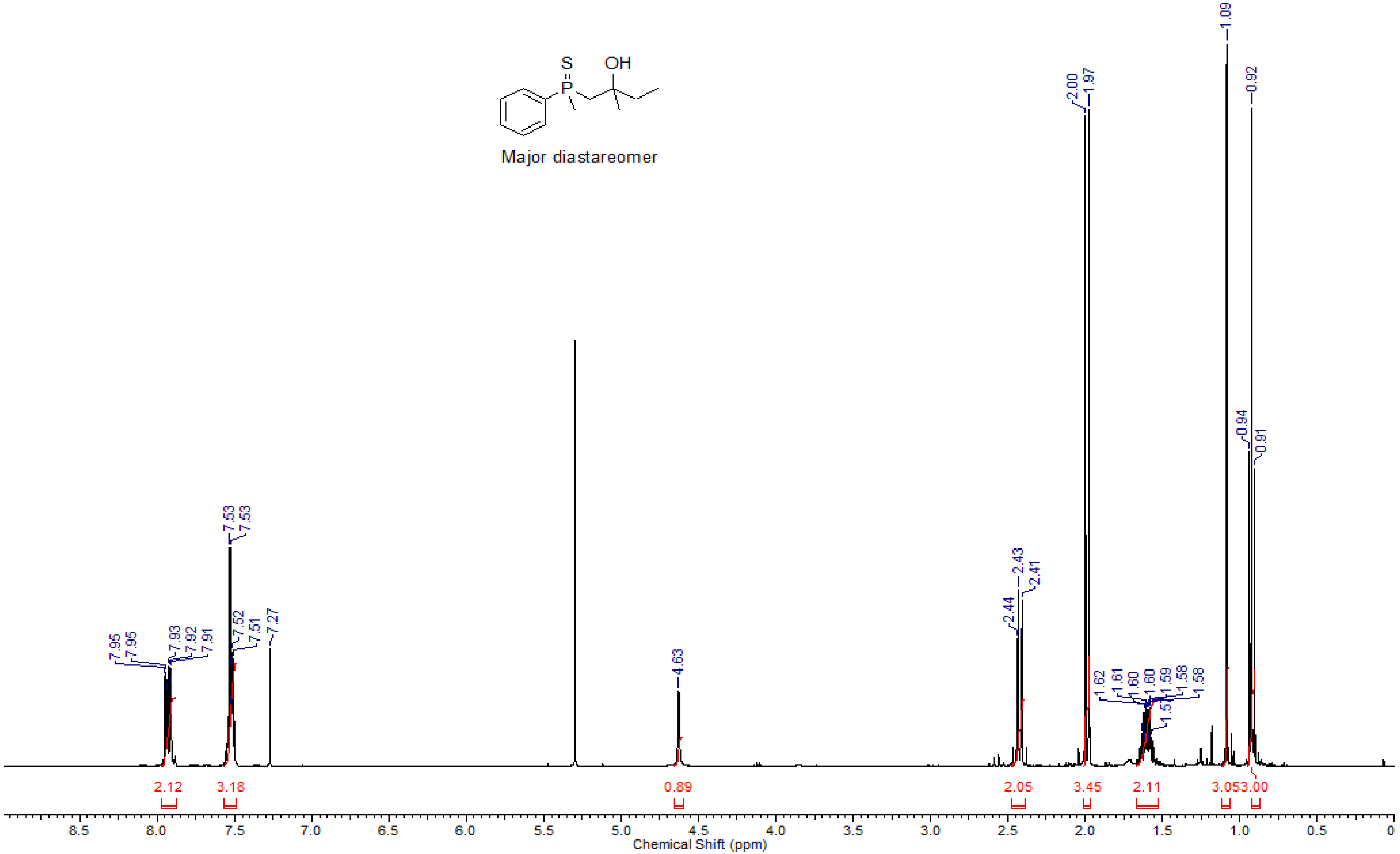

^{1}H NMR spectrum of (2-hydroxy-2-methylbutyl)methylphenylphosphine sulfide (major diastereomer) (**12**) (CDCl3, 500 MHz).

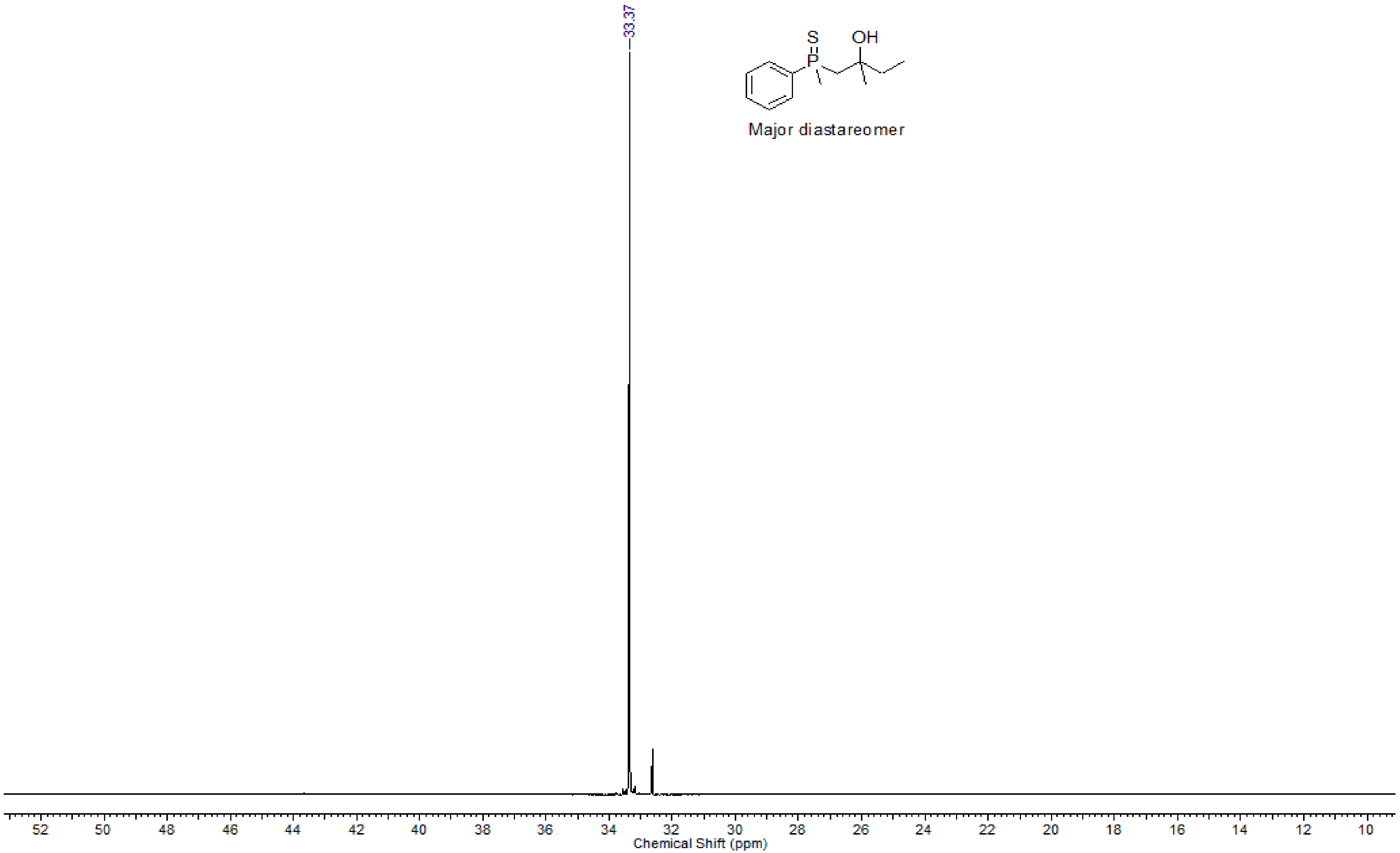

^{31}P NMR spectrum of (2-hydroxy-2-methylbutyl)methylphenylphosphine sulfide (major diastereomer) (**12**) (CDCl3, 202 MHz).

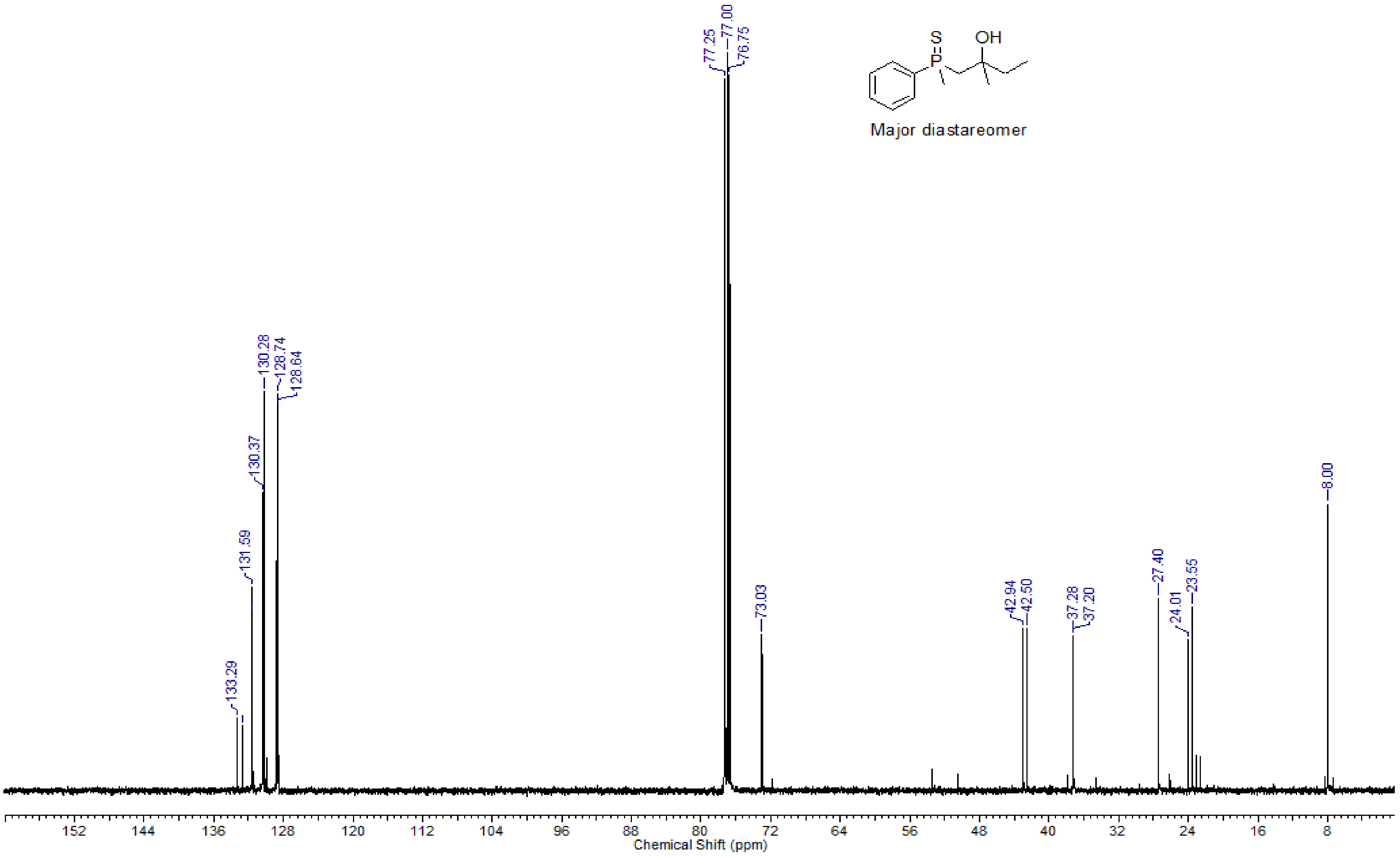

^{13}C NMR spectrum of (2-hydroxy-2-methylbutyl)methylphenylphosphine sulfide (major diastereomer) (**12**) (CDCl3, 126 MHz).

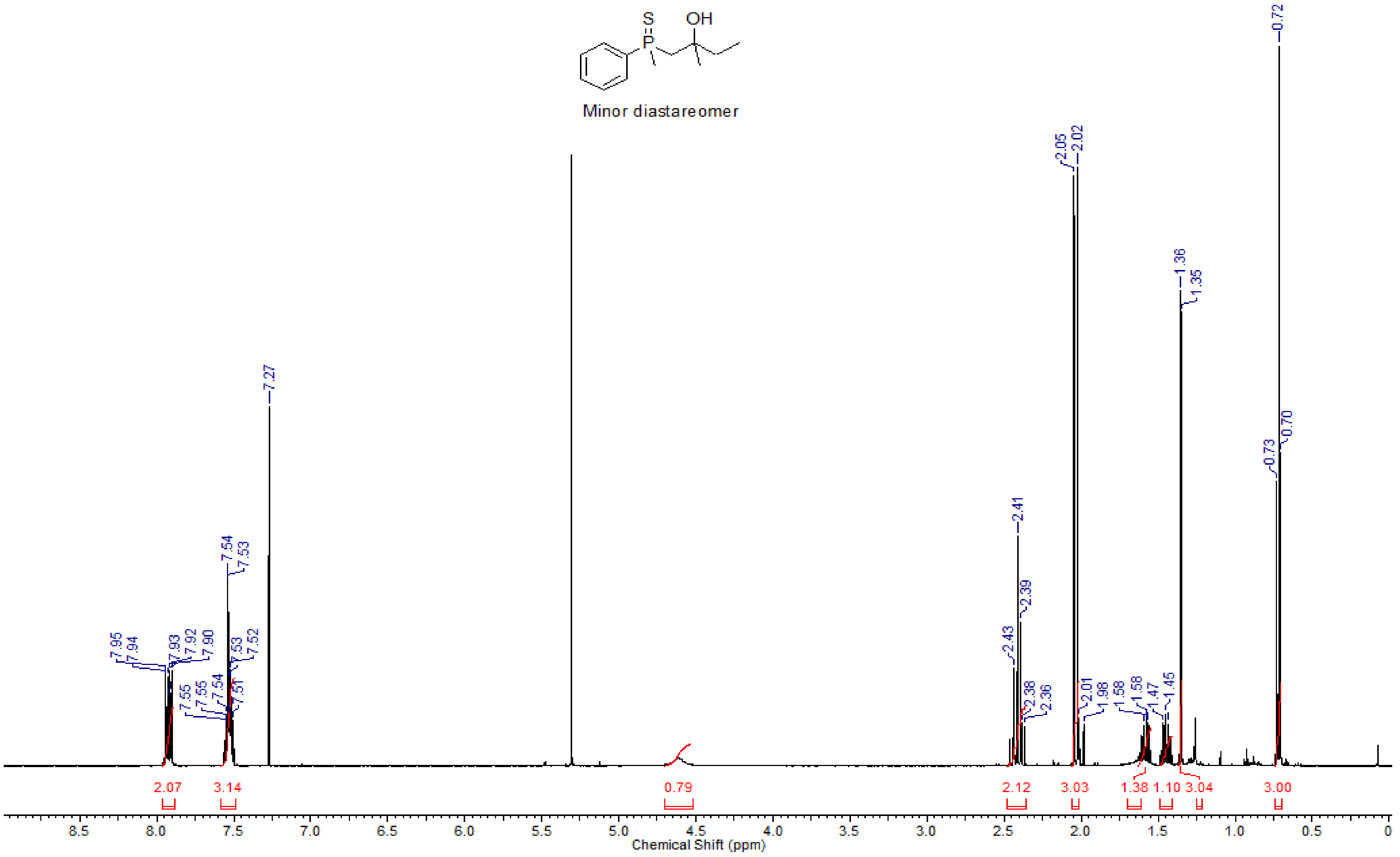

^{1}H NMR spectrum of (2-hydroxy-2-methylbutyl)methylphenylphosphine sulfide (minor diastereomer) (**12**) (CDCl3, 500 MHz).

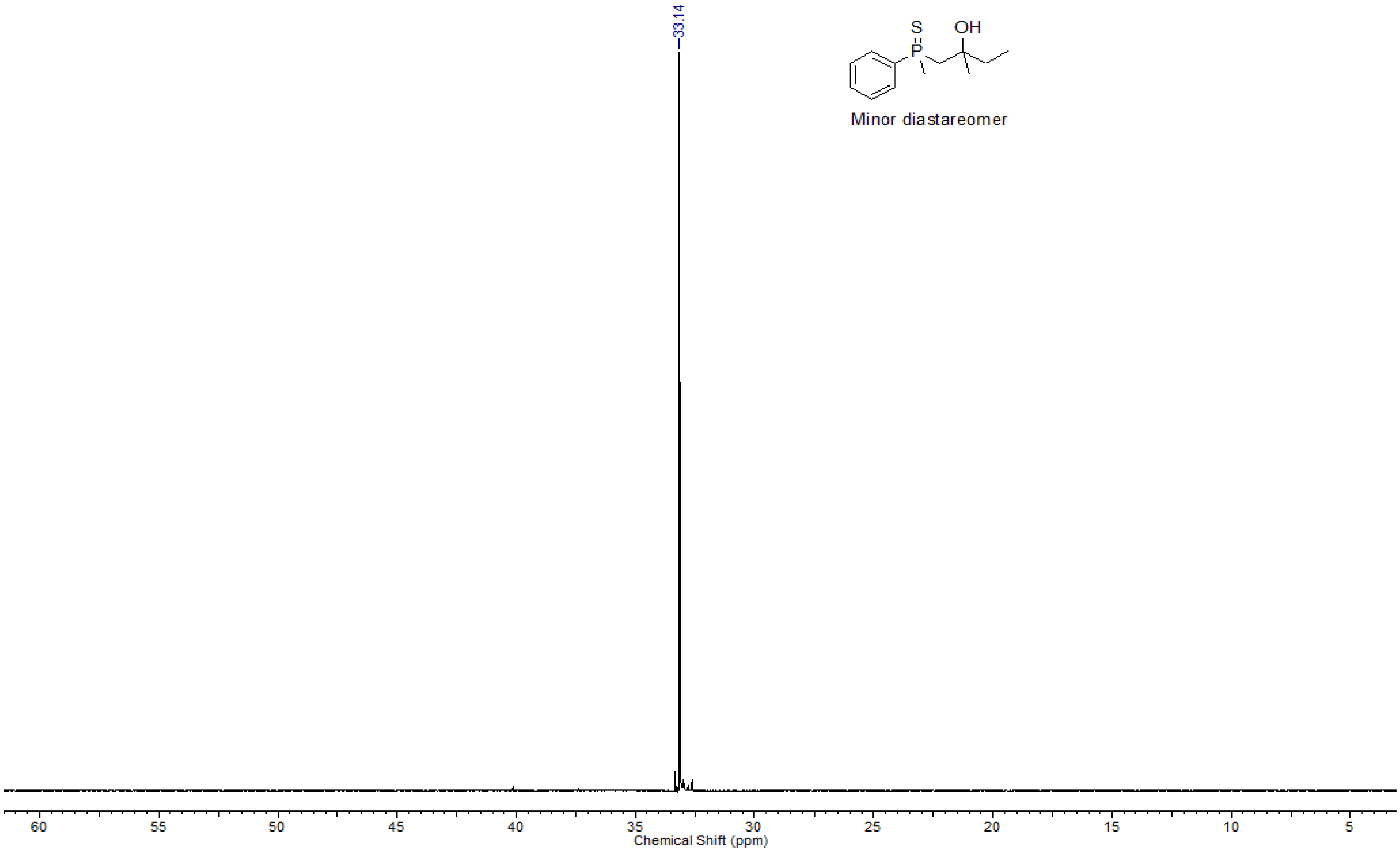

^{31}P NMR spectrum of (2-hydroxy-2-methylbutyl)methylphenylphosphine sulfide (minor diastereomer) (**12**) (CDCl3, 202 MHz).

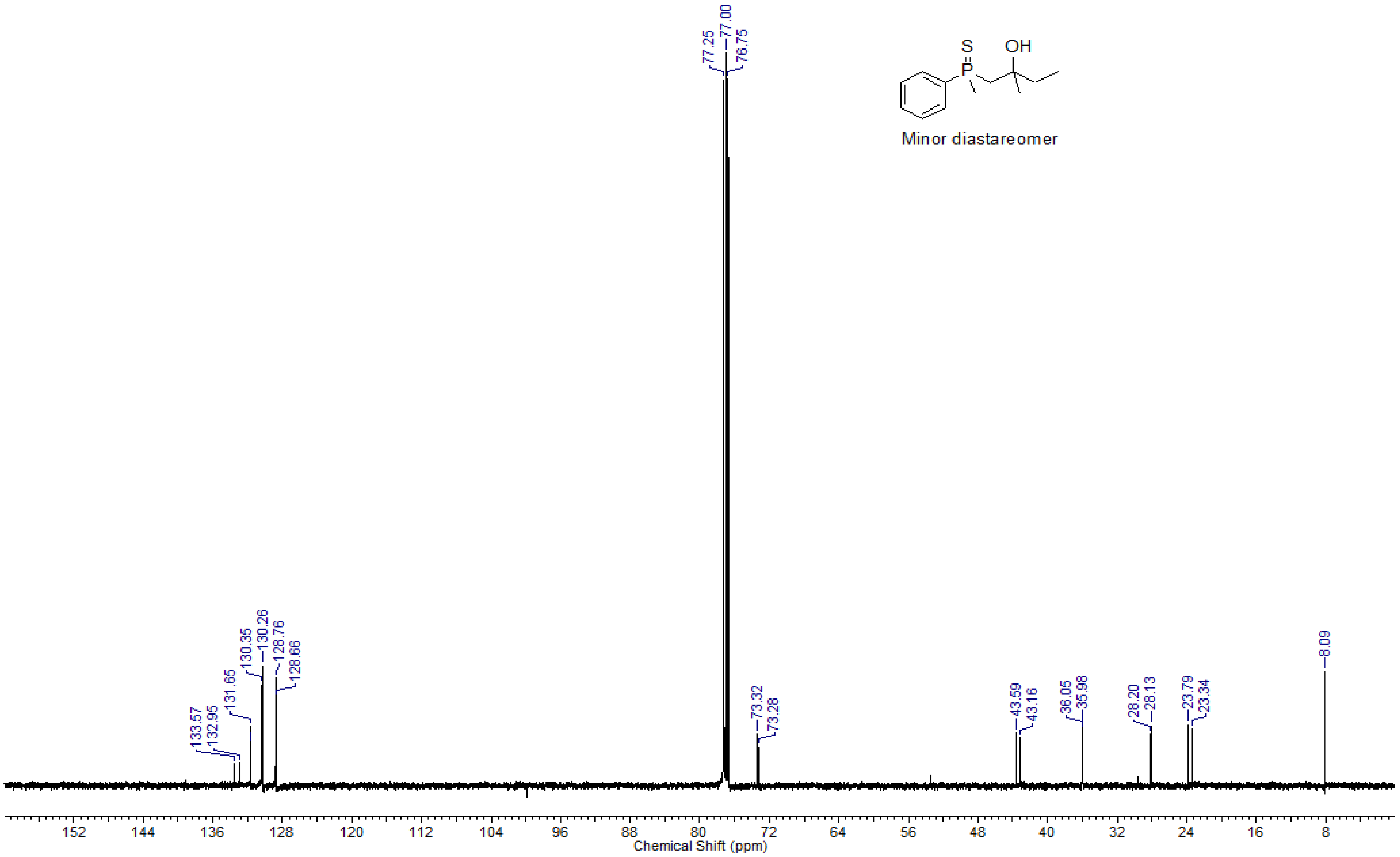

^{13}C NMR spectrum of (2-hydroxy-2-methylbutyl)methylphenylphosphine sulfide (minor diastereomer) (**12**) (CDCl3, 126 MHz).

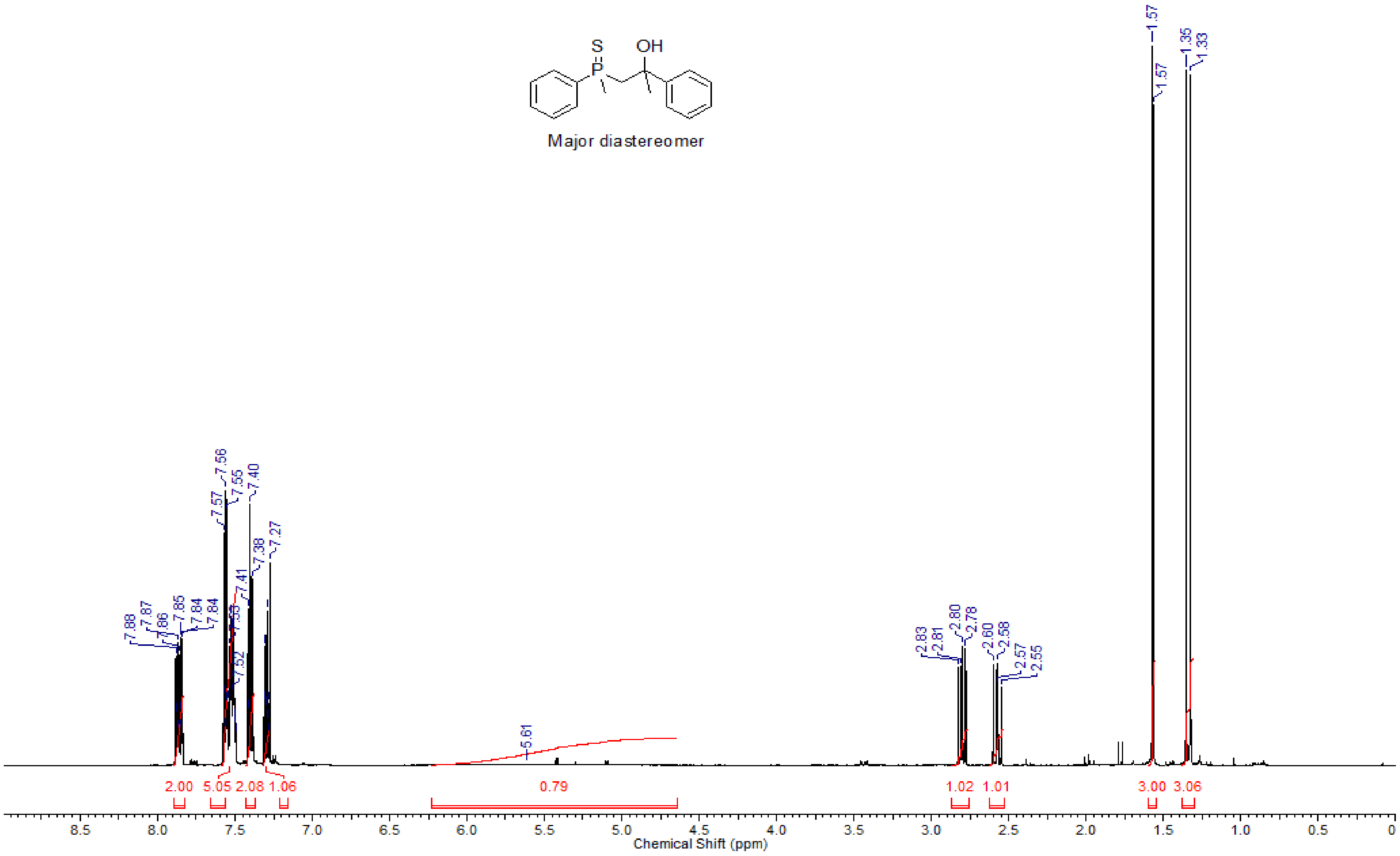

^{1}H NMR spectrum of (2-hydroxy-2-phenylpropyl)methylphenylphosphine sulfide (major diastereomer) (**13**) (CDCl3, 500 MHz).

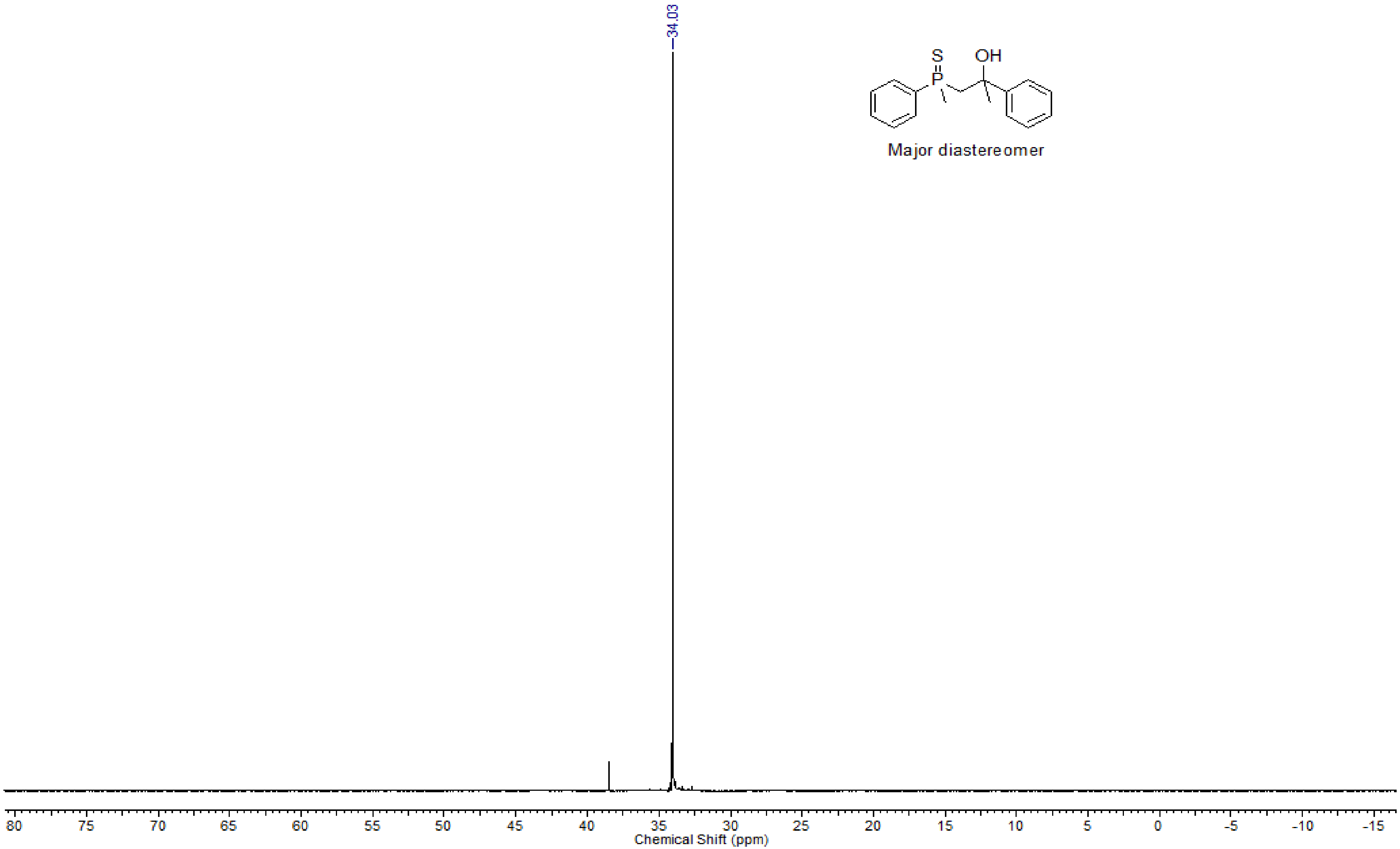

^{31}P NMR spectrum of (2-hydroxy-2-phenylpropyl)methylphenylphosphine sulfide (major diastereomer) (**13**) (CDCl3, 202 MHz).

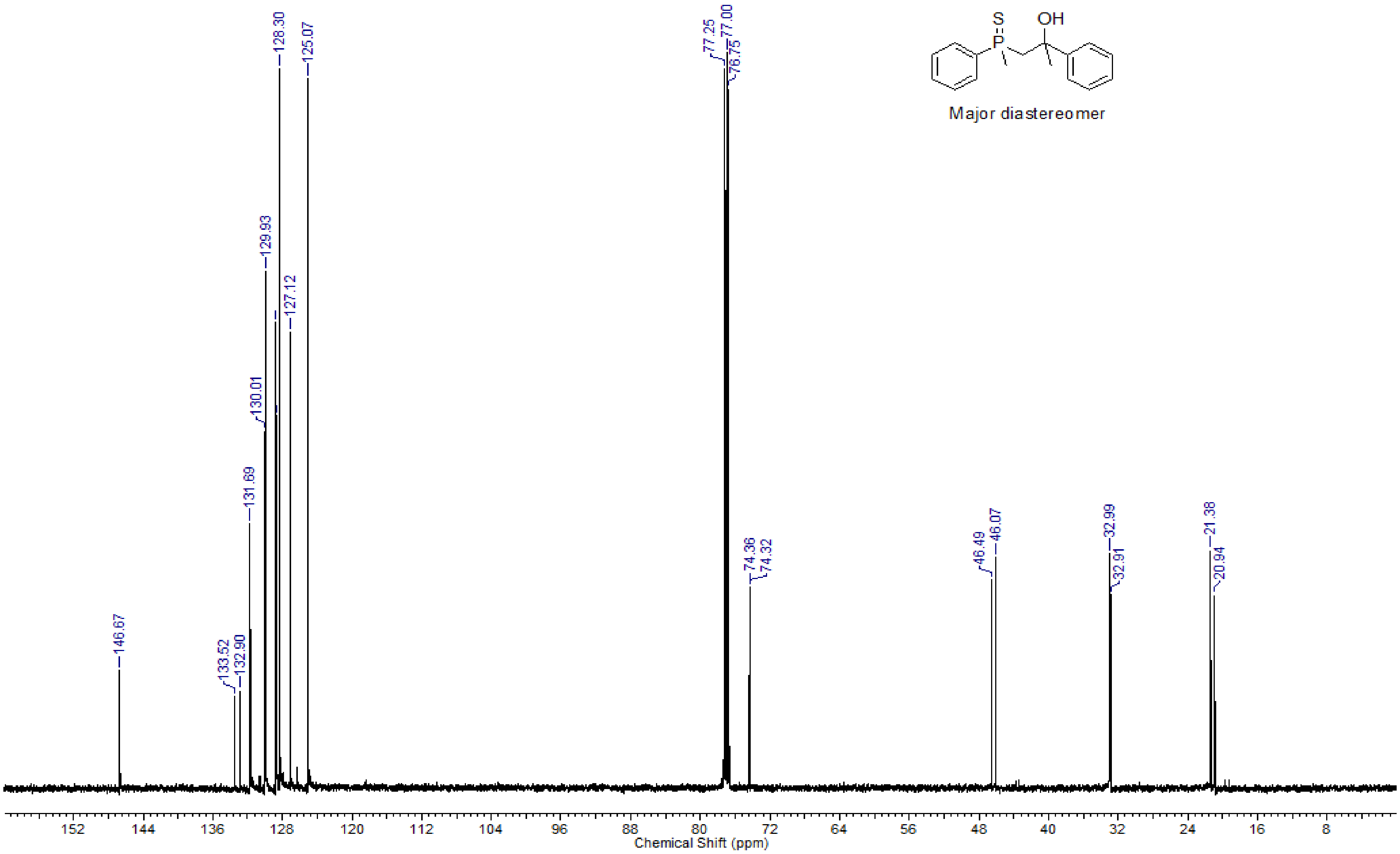

^{13}C NMR spectrum of (2-hydroxy-2-phenylpropyl)methylphenylphosphine sulfide (major diastereomer) (**13**) (CDCl3, 126 MHz).

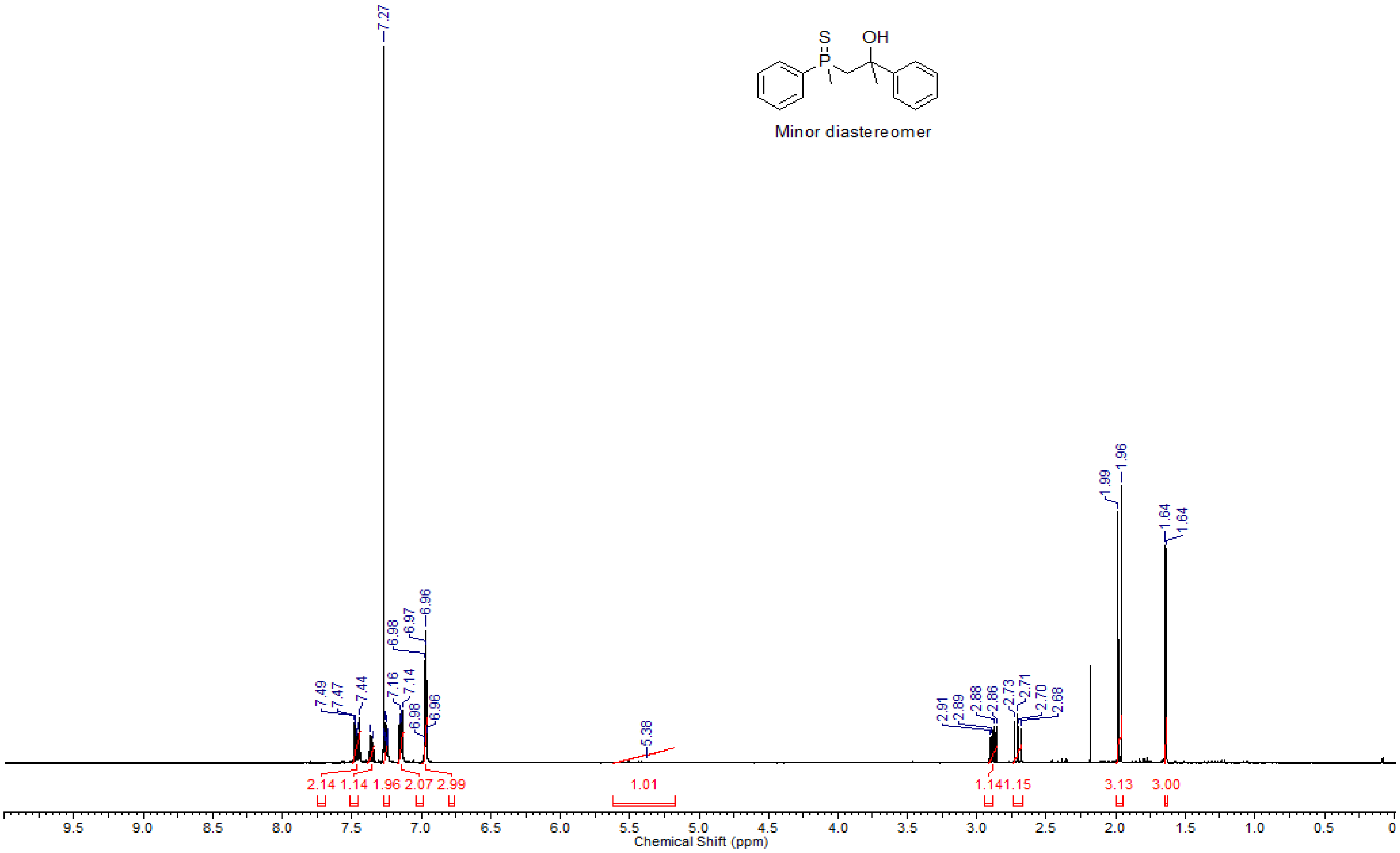

^{1}H NMR spectrum of (2-hydroxy-2-phenylpropyl)methylphenylphosphine sulfide (minor diastereomer) (**13**) (CDCl3, 500 MHz).

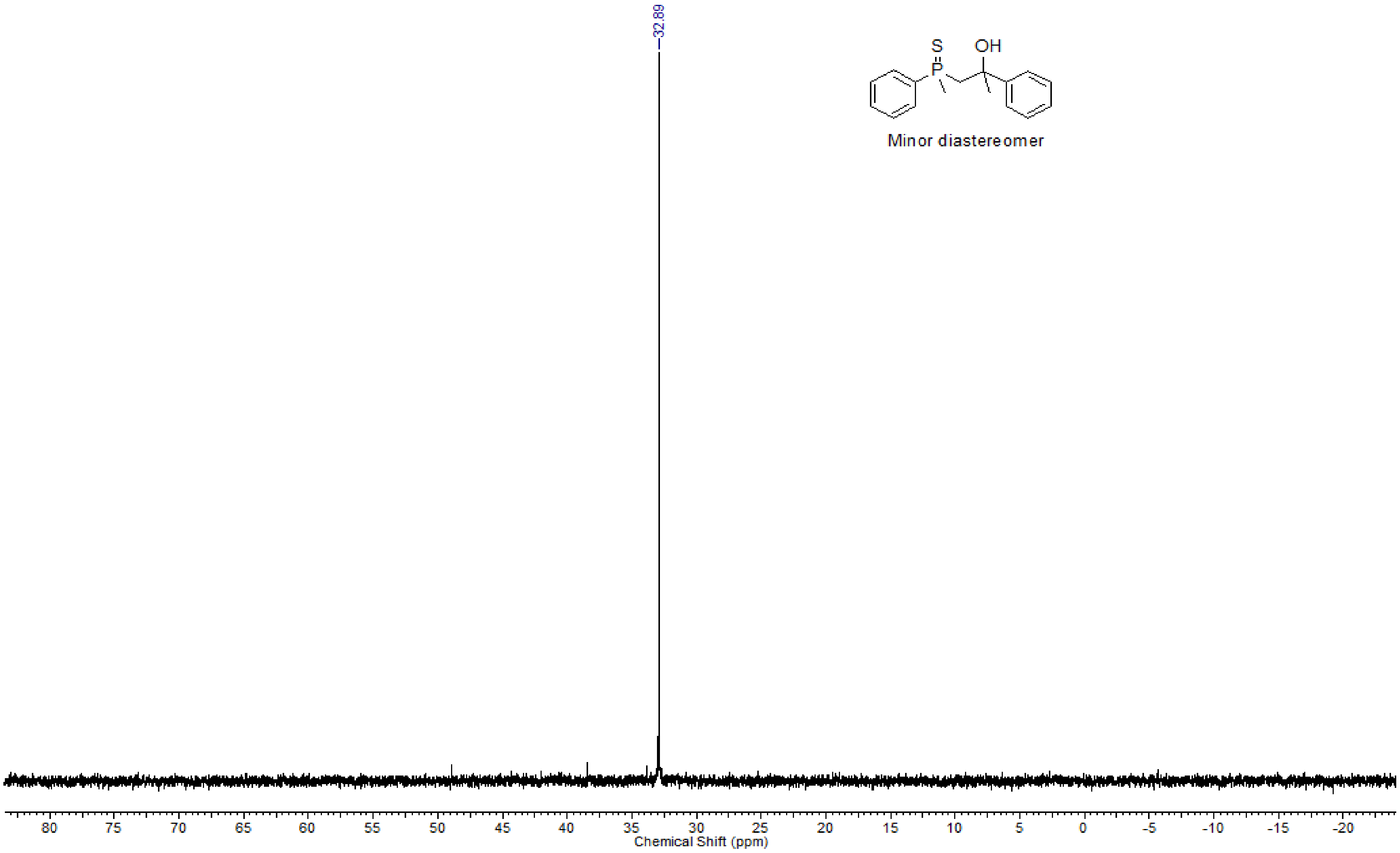

^{31}P NMR spectrum of (2-hydroxy-2-phenylpropyl)methylphenylphosphine sulfide (minor diastereomer) (**13**) (CDCl3, 202 MHz).

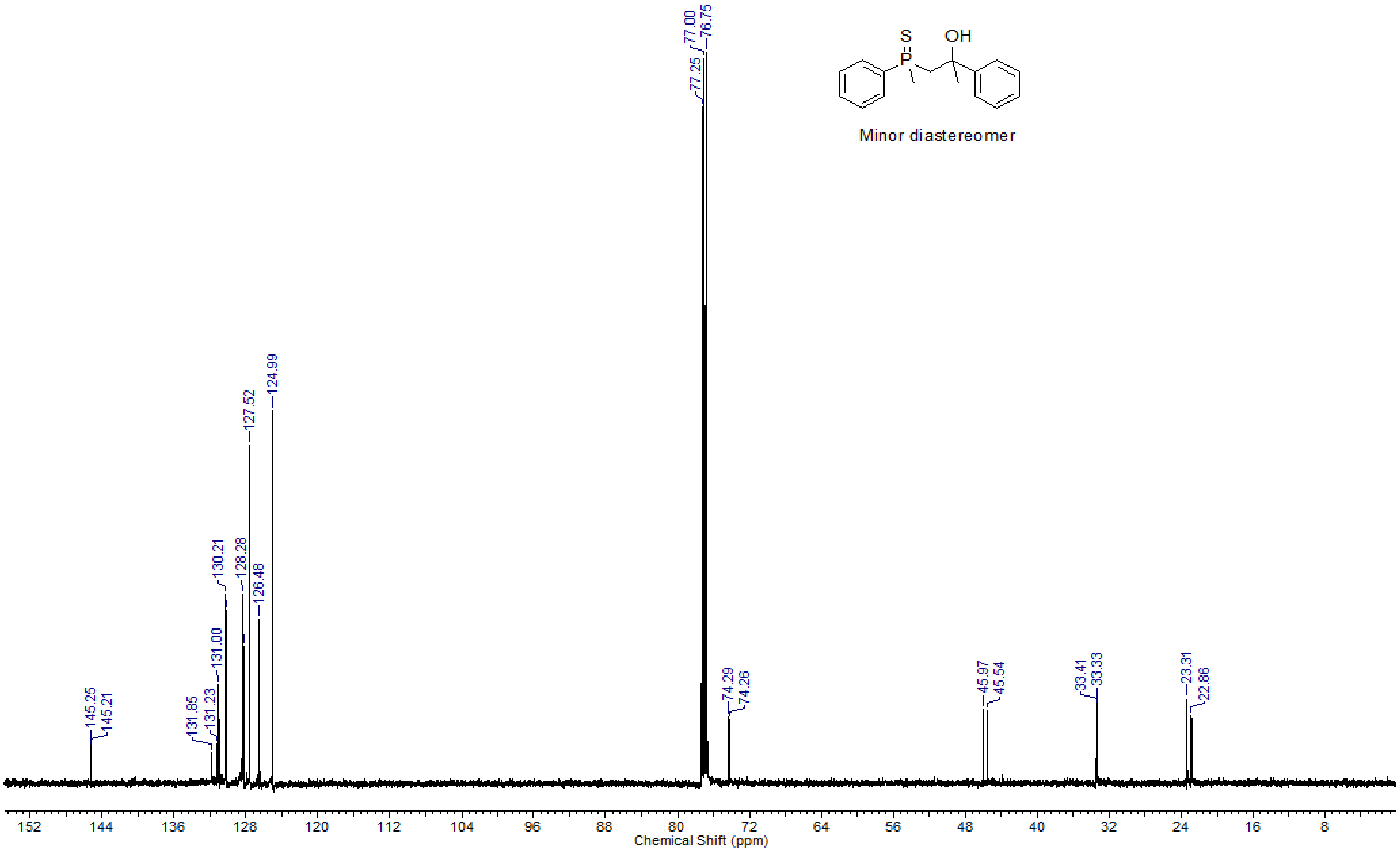

^{13}C NMR spectrum of (2-hydroxy-2-phenylpropyl)methylphenylphosphine sulfide(minor diastereomer) (**13**) (CDCl3, 126 MHz).

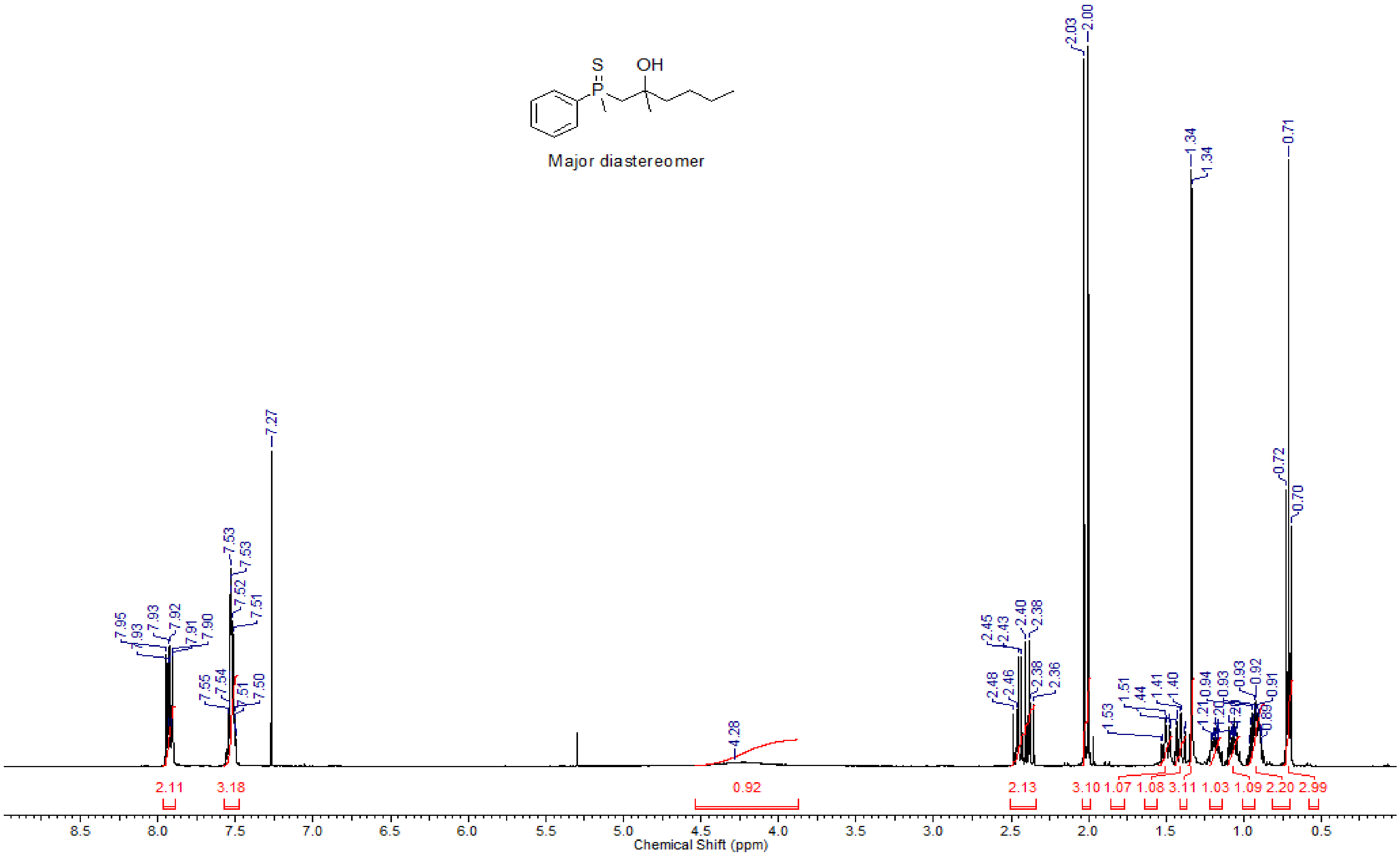

^{1}H NMR spectrum of (2-hydroxy-2-methylhexyl)methylphenylphosphine sulfide (major diastereomer) (**14**) (CDCl3, 500 MHz).

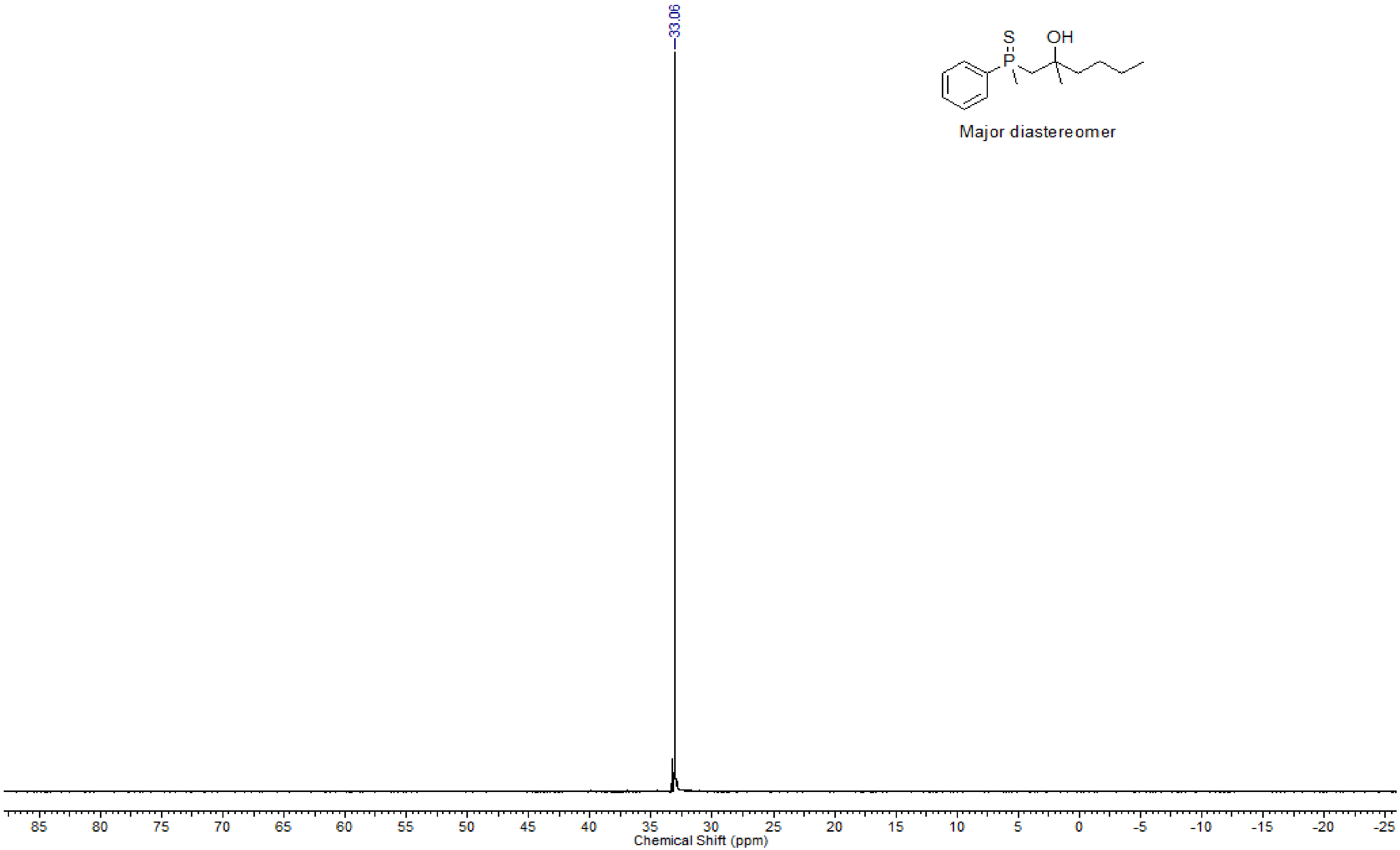

^{31}P NMR spectrum of (2-hydroxy-2-methylhexyl)methylphenylphosphine sulfide (major diastereomer) (**14**) (CDCl3, 202 MHz).

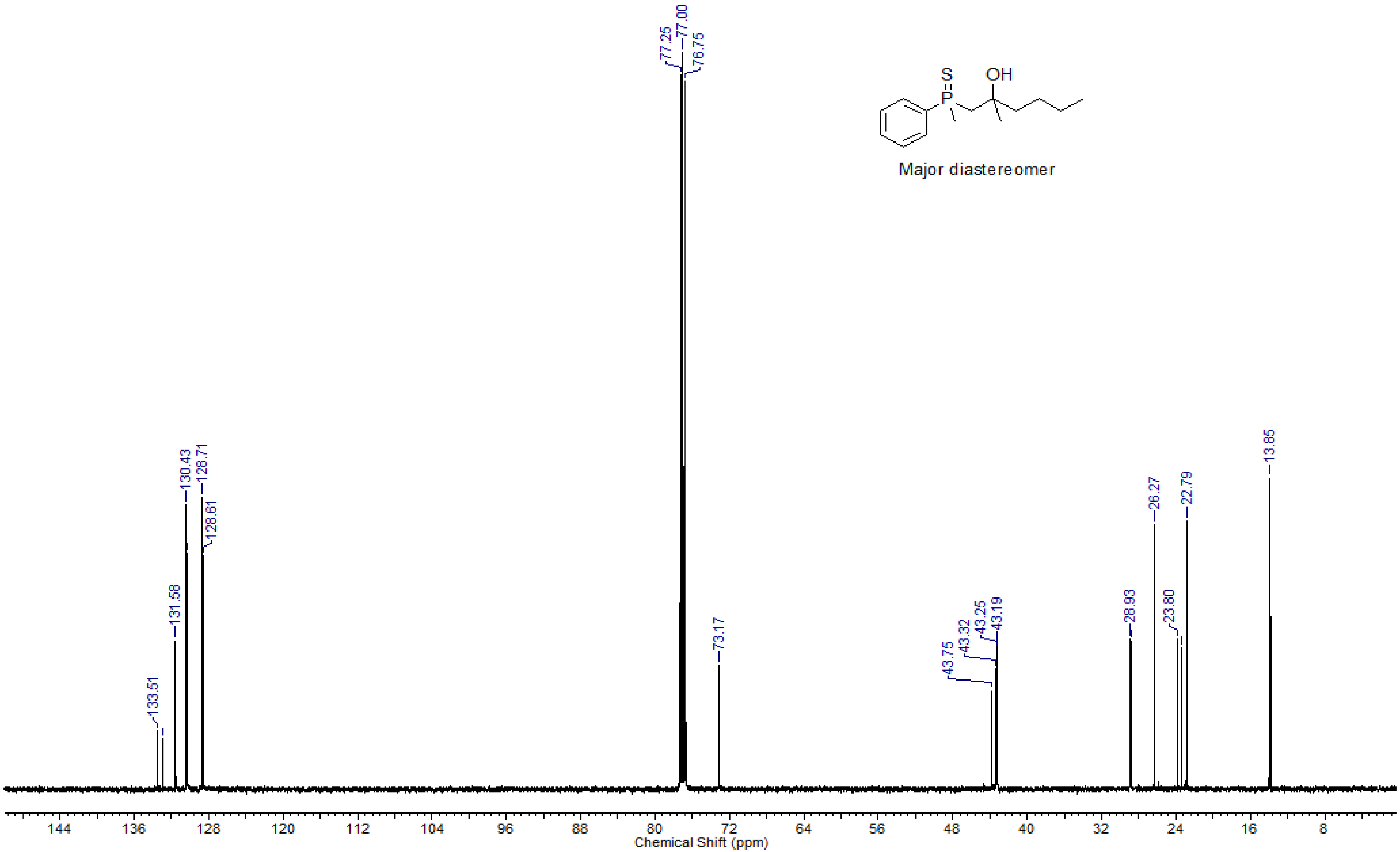

^{13}C NMR spectrum of (2-hydroxy-2-methylhexyl)methylphenylphosphine sulfide (major diastereomer) (**14**) (CDCl3, 126 MHz).

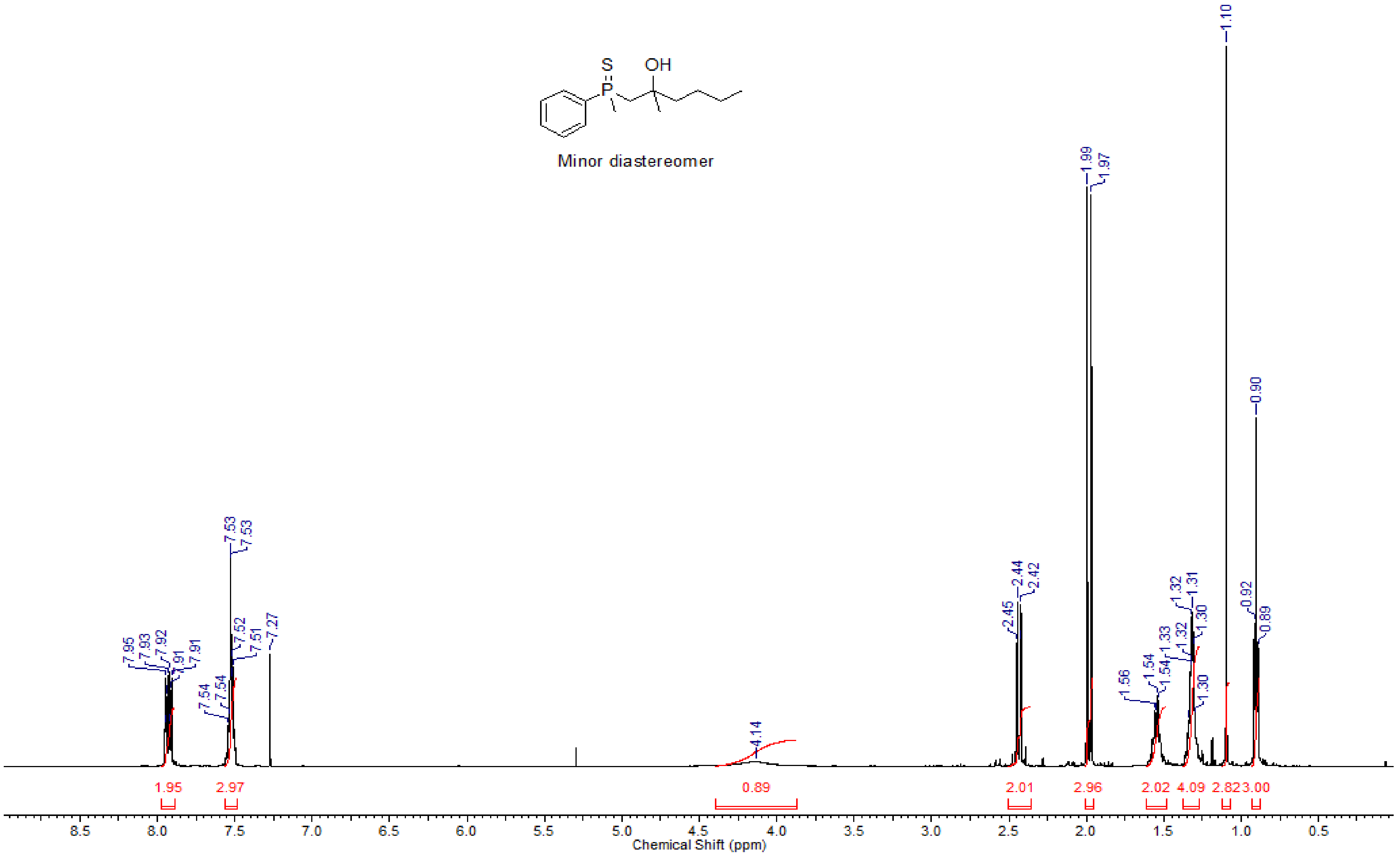

^{1}H NMR spectrum of (2-hydroxy-2-methylhexyl)methylphenylphosphine sulfide (minor diastereomer) (**14**) (CDCl3, 500 MHz).

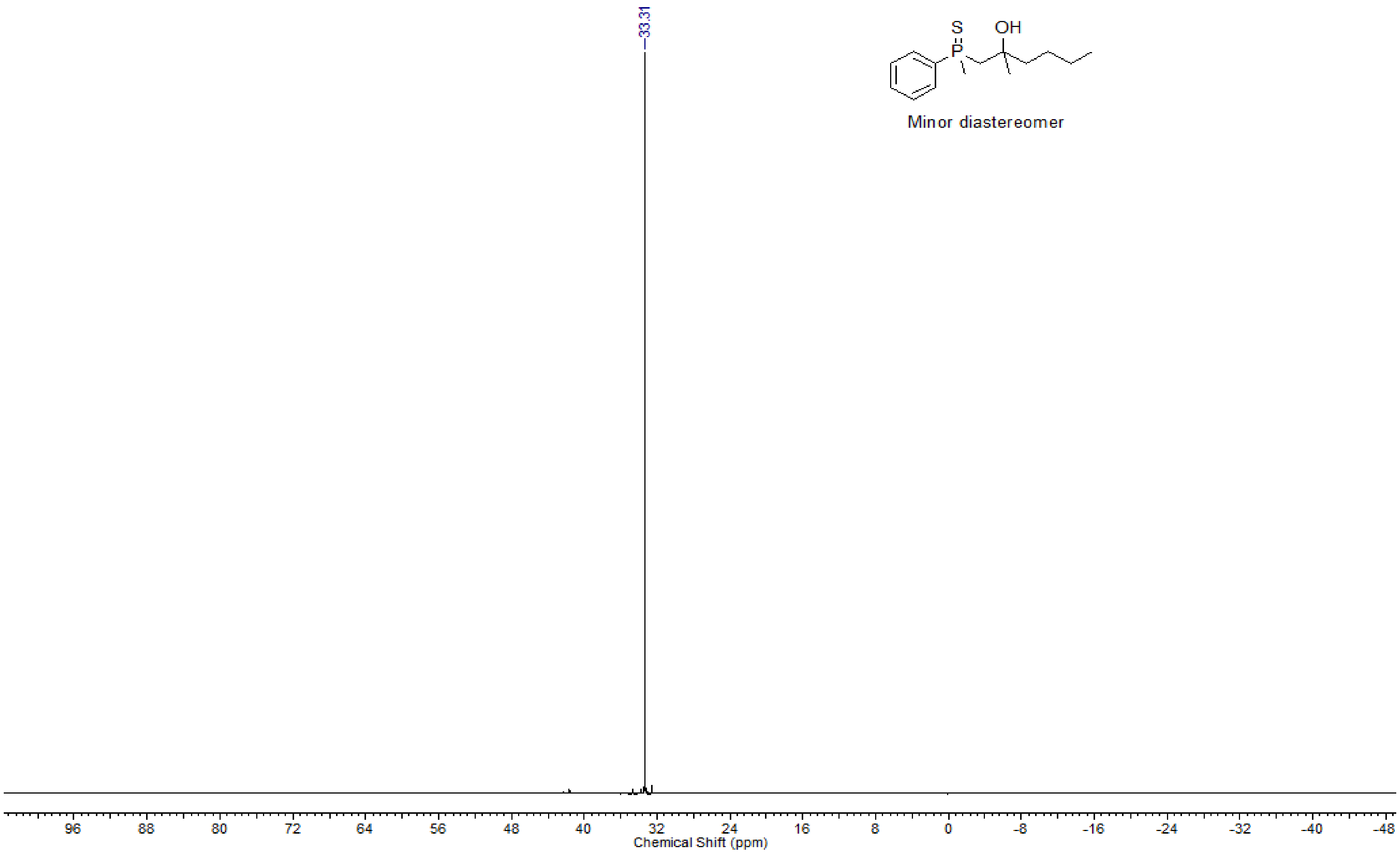

^{31}P NMR spectrum of (2-hydroxy-2-methylhexyl)methylphenylphosphine sulfide (minor diastereomer) (**14**) (CDCl3, 202 MHz).

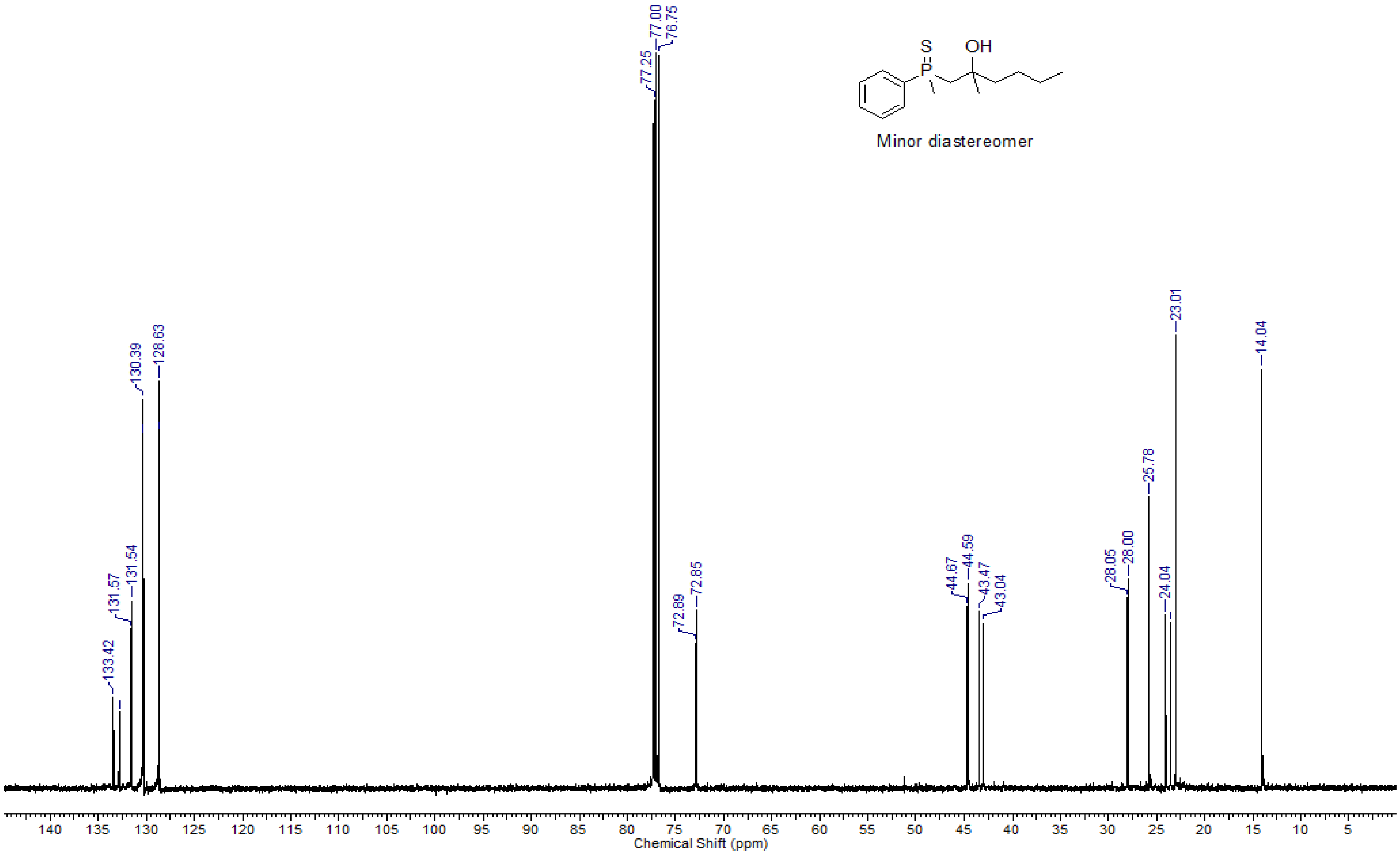

^{13}C NMR spectrum of (2-hydroxy-2-methylhexyl)methylphenylphosphine sulfide (minor diastereomer) (**14**) (CDCl3, 126 MHz).

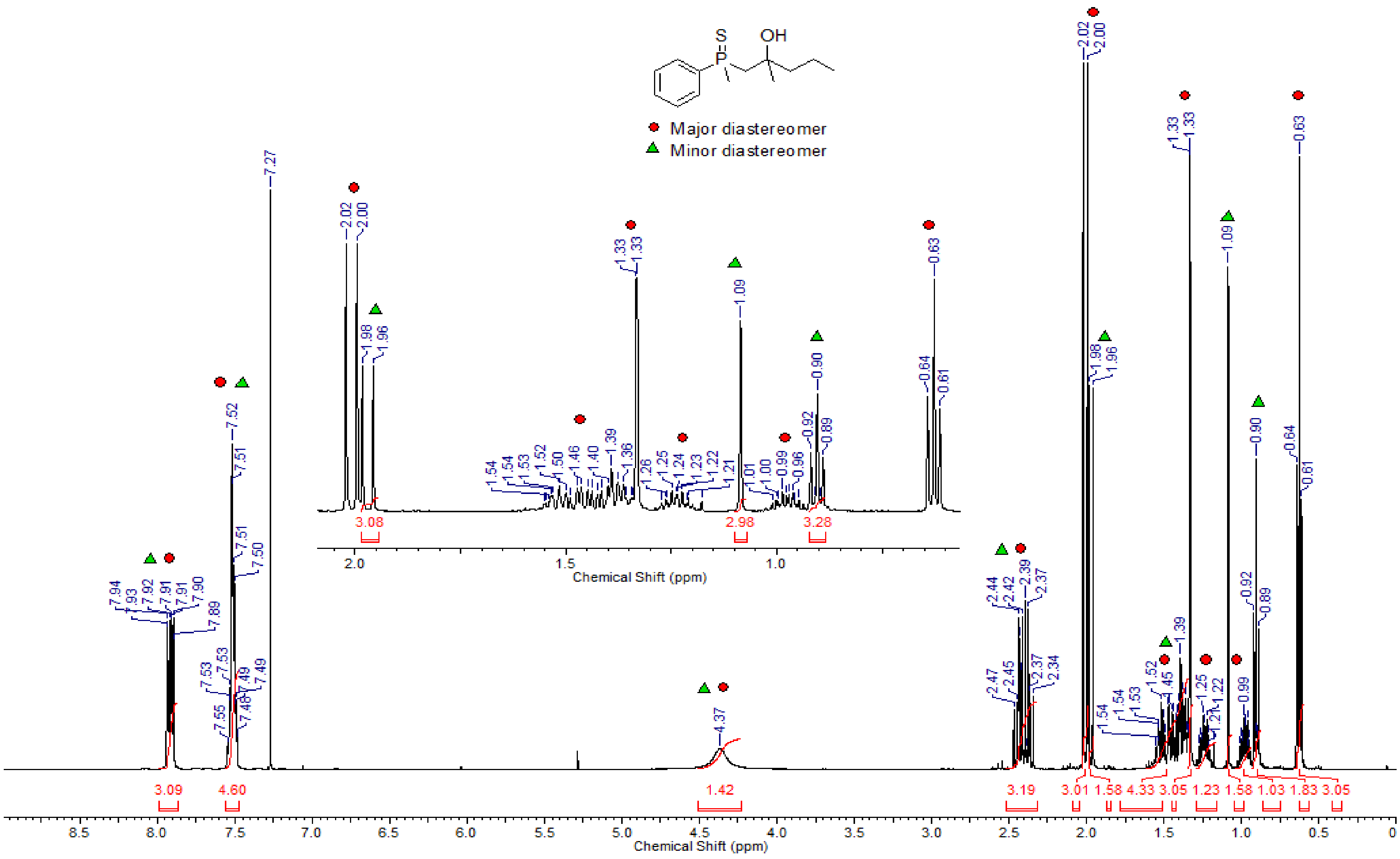

^{1}H NMR spectrum of (2-hydroxy-2-methylpentyl)methylphenylphosphine sulfide (**15**) (CDCl3, 500 MHz).

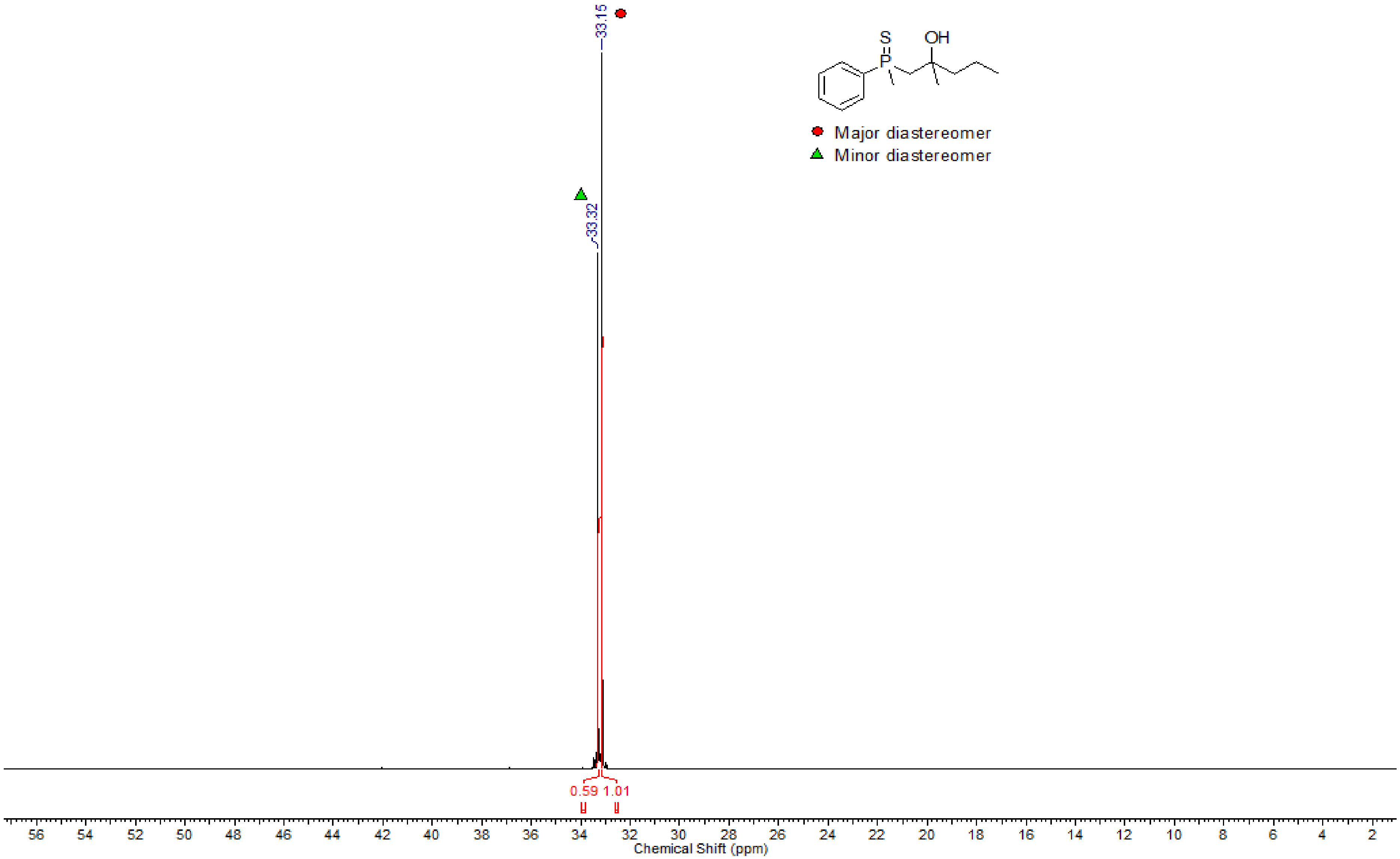

^{31}P NMR spectrum of (2-hydroxy-2-methylpentyl)methylphenylphosphine sulfide (**15**) (CDCl3, 202 MHz).

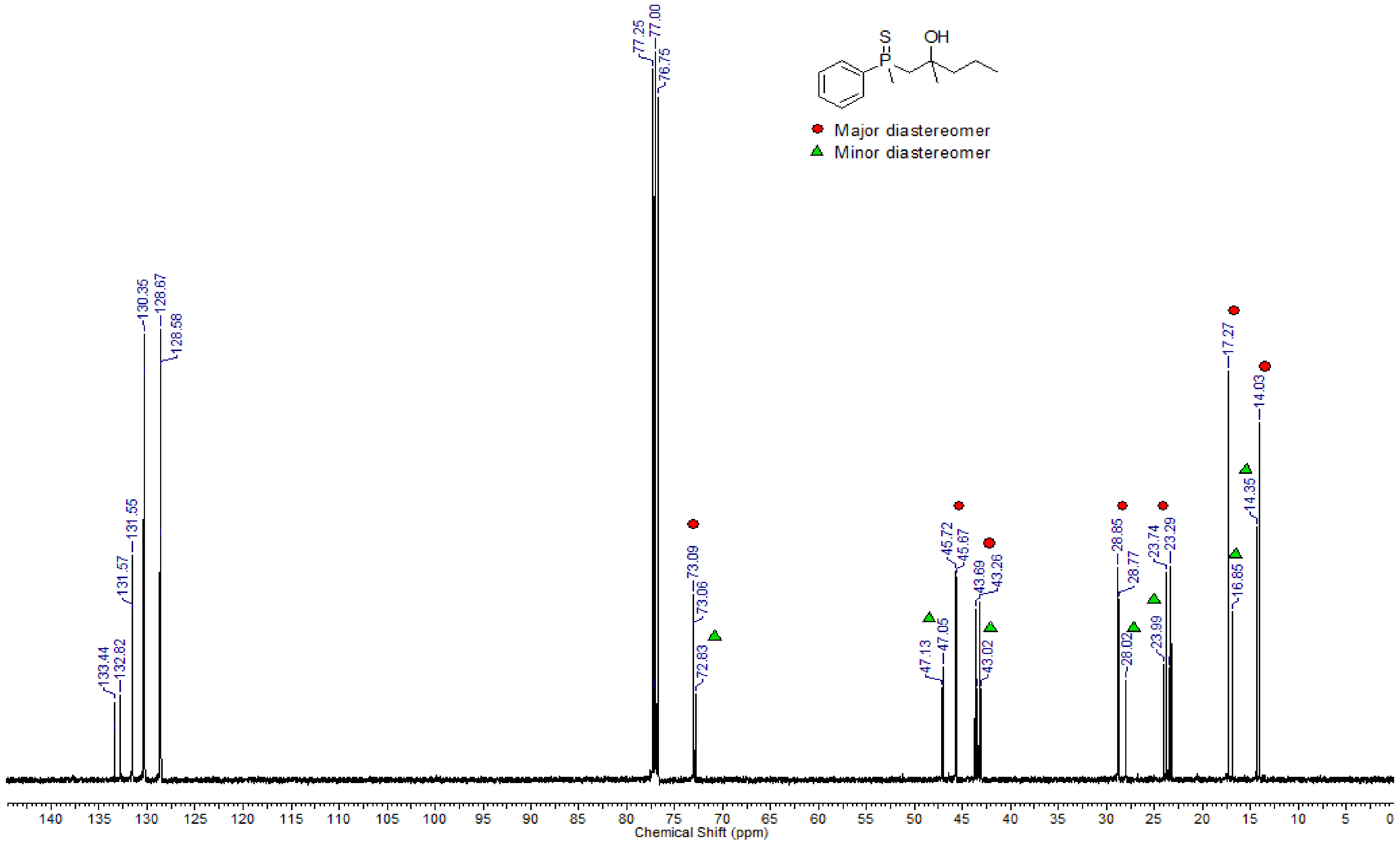

^{13}C NMR spectrum of (2-hydroxy-2-methylpentyl)methylphenylphosphine sulfide (**15**) (CDCl3, 126 MHz).

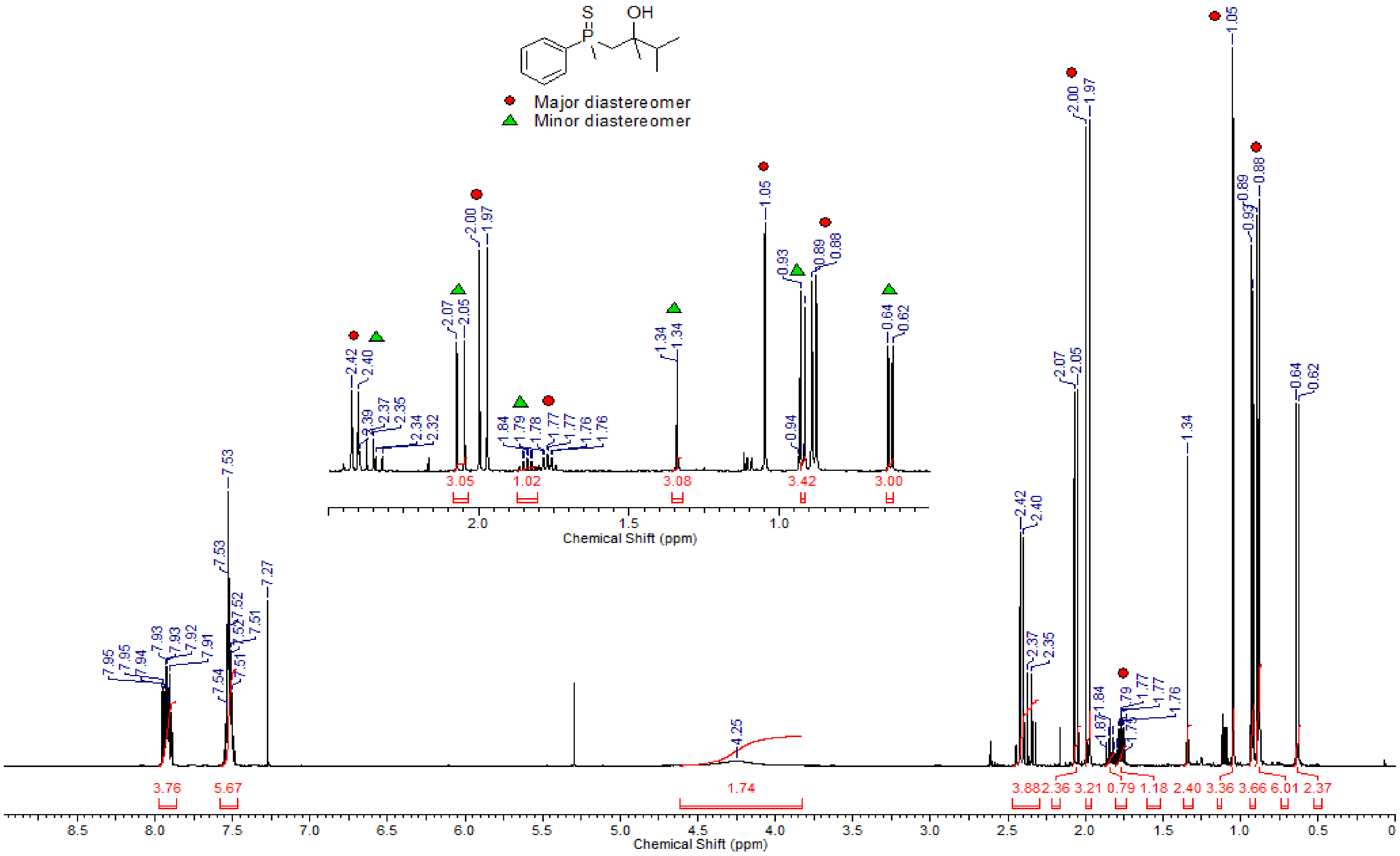

^{1}H NMR spectrum of (2-hydroxy-2,3-dimethylbutyl)methylphenylphosphine sulfide (**16**) (CDCl3, 500 MHz).

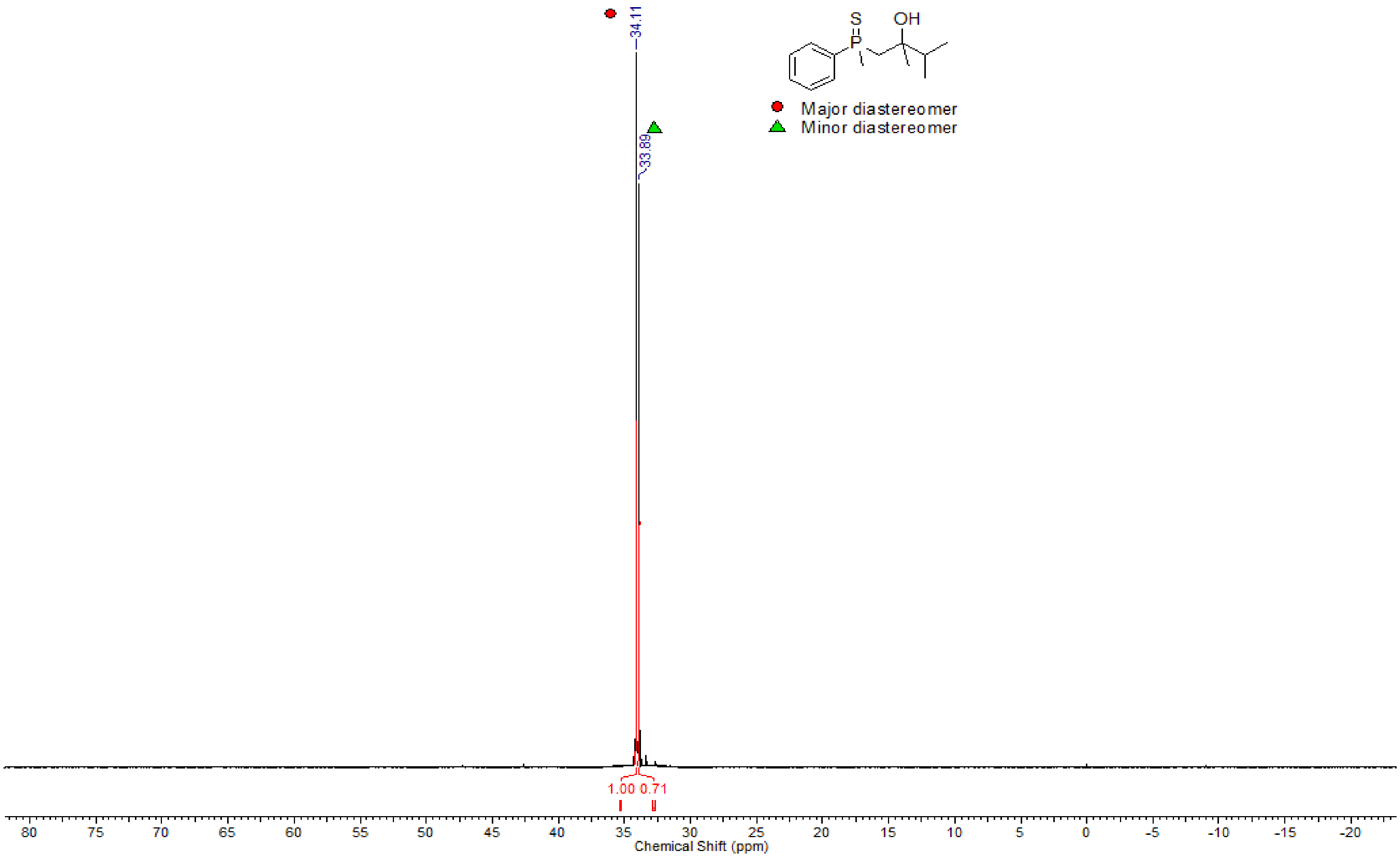

^{31}P NMR spectrum of (2-hydroxy-2,3-dimethylbutyl)methylphenylphosphine sulfide (**16**) (CDCl3, 202 MHz).

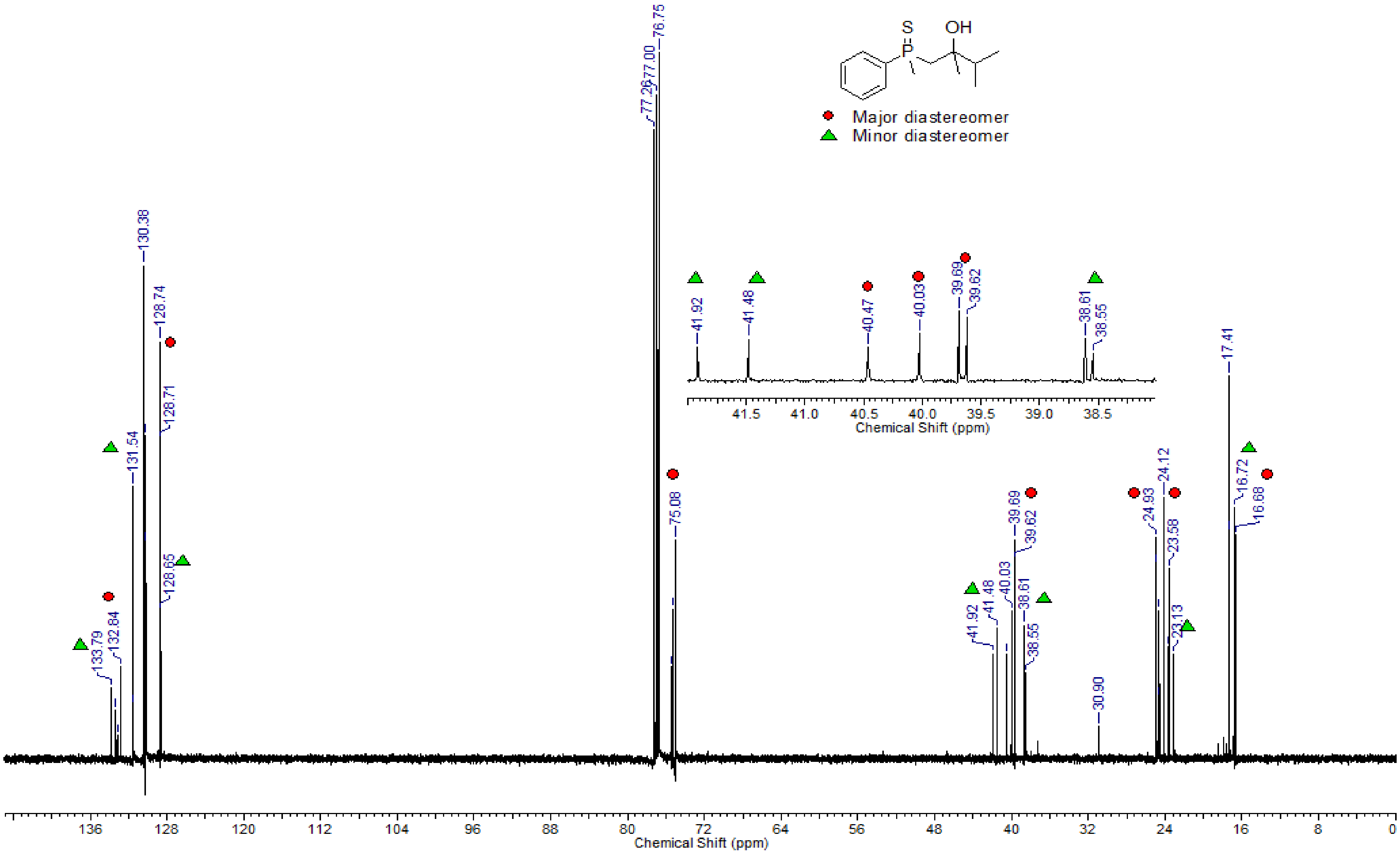

^{13}C NMR spectrum of (2-hydroxy-2,3-dimethylbutyl)methylphenylphosphine sulfide (**16**) (CDCl3, 126 MHz).

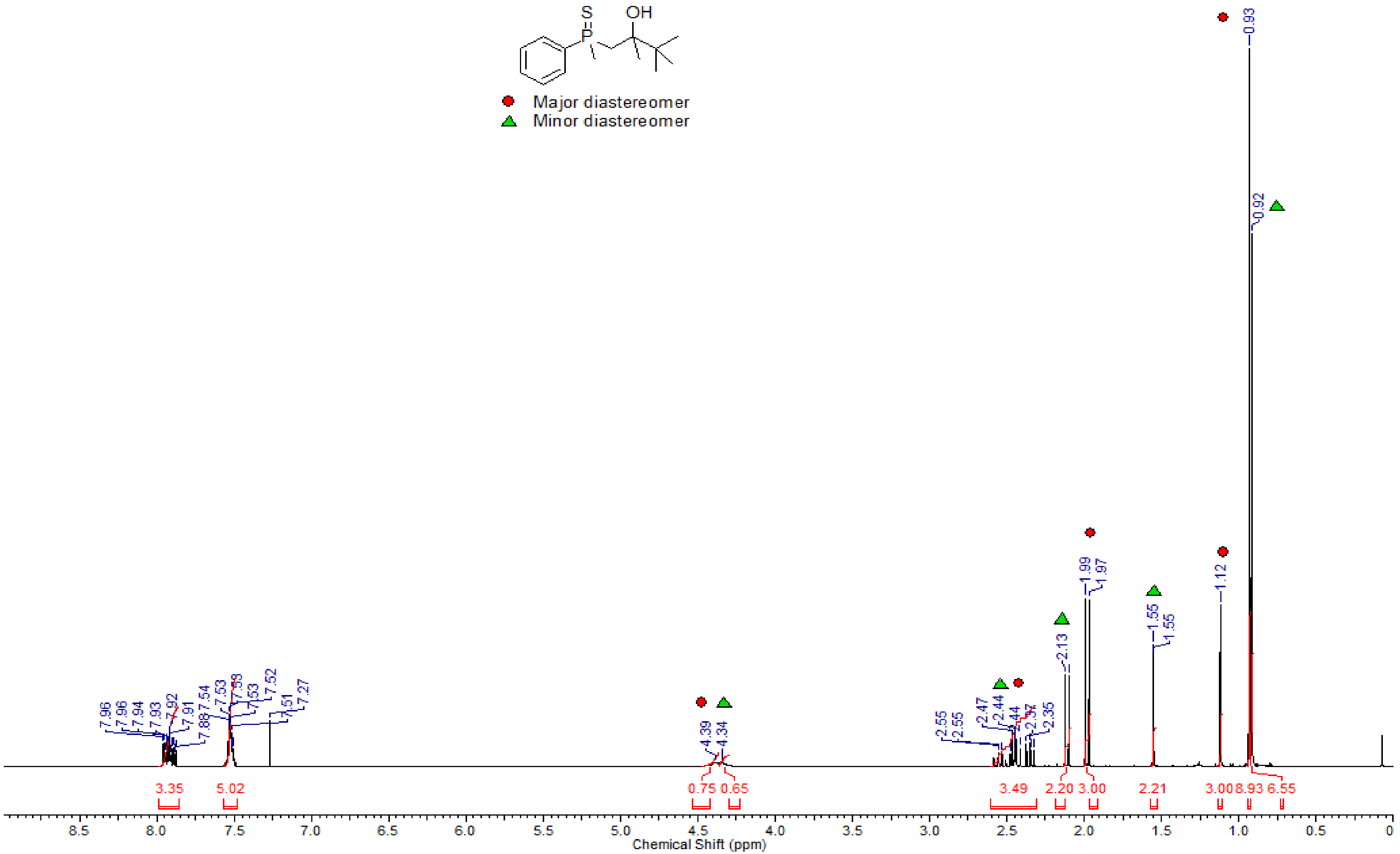

^{1}H NMR spectrum of (2-hydroxy-2,3,3-trimethylbutyl)methylphenylphosphine sulfide (**17**) (CDCl3, 500 MHz).

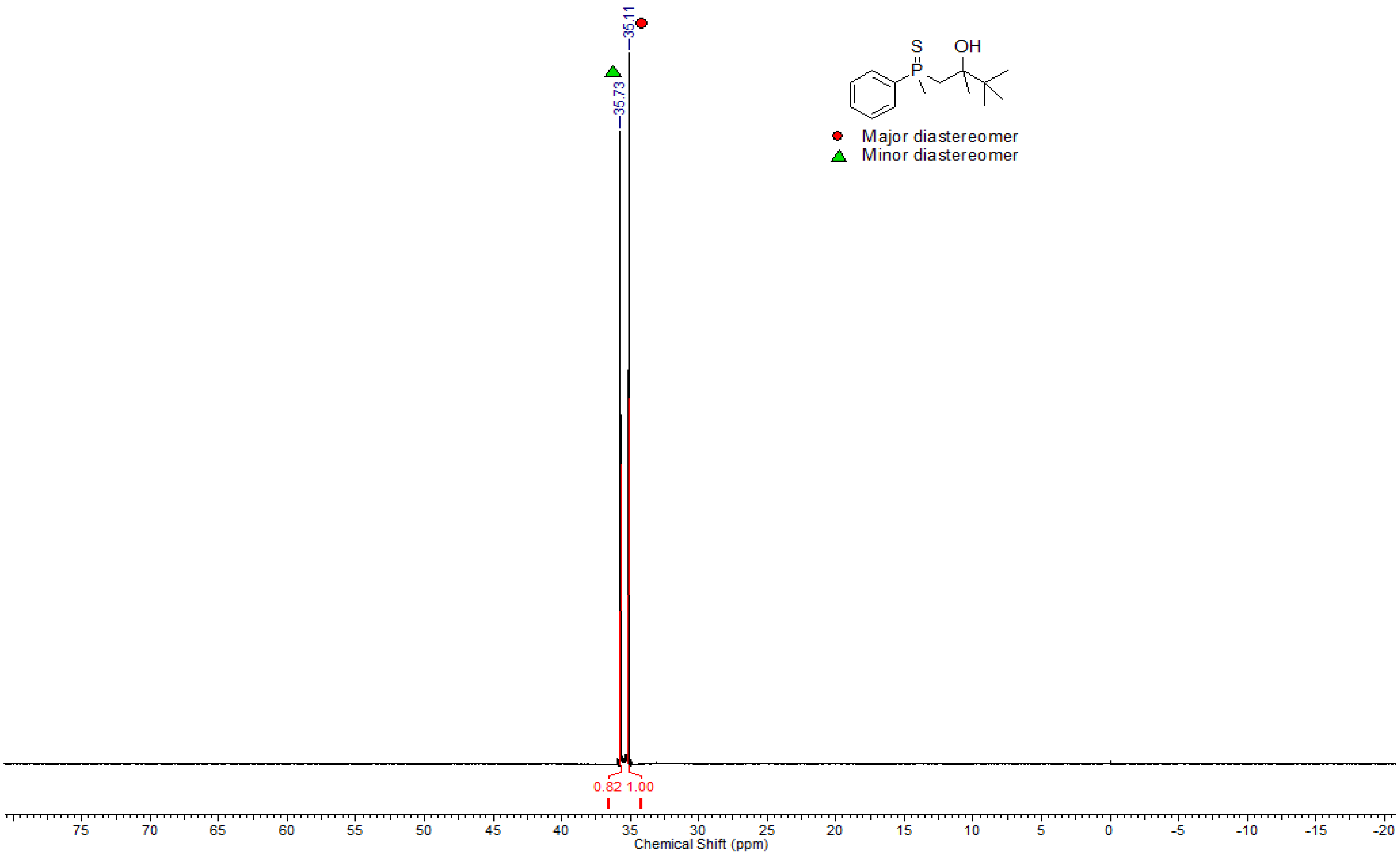

^{31}P NMR spectrum of (2-hydroxy-2,3,3-trimethylbutyl)methylphenylphosphine sulfide (**17**) (CDCl3, 202 MHz).

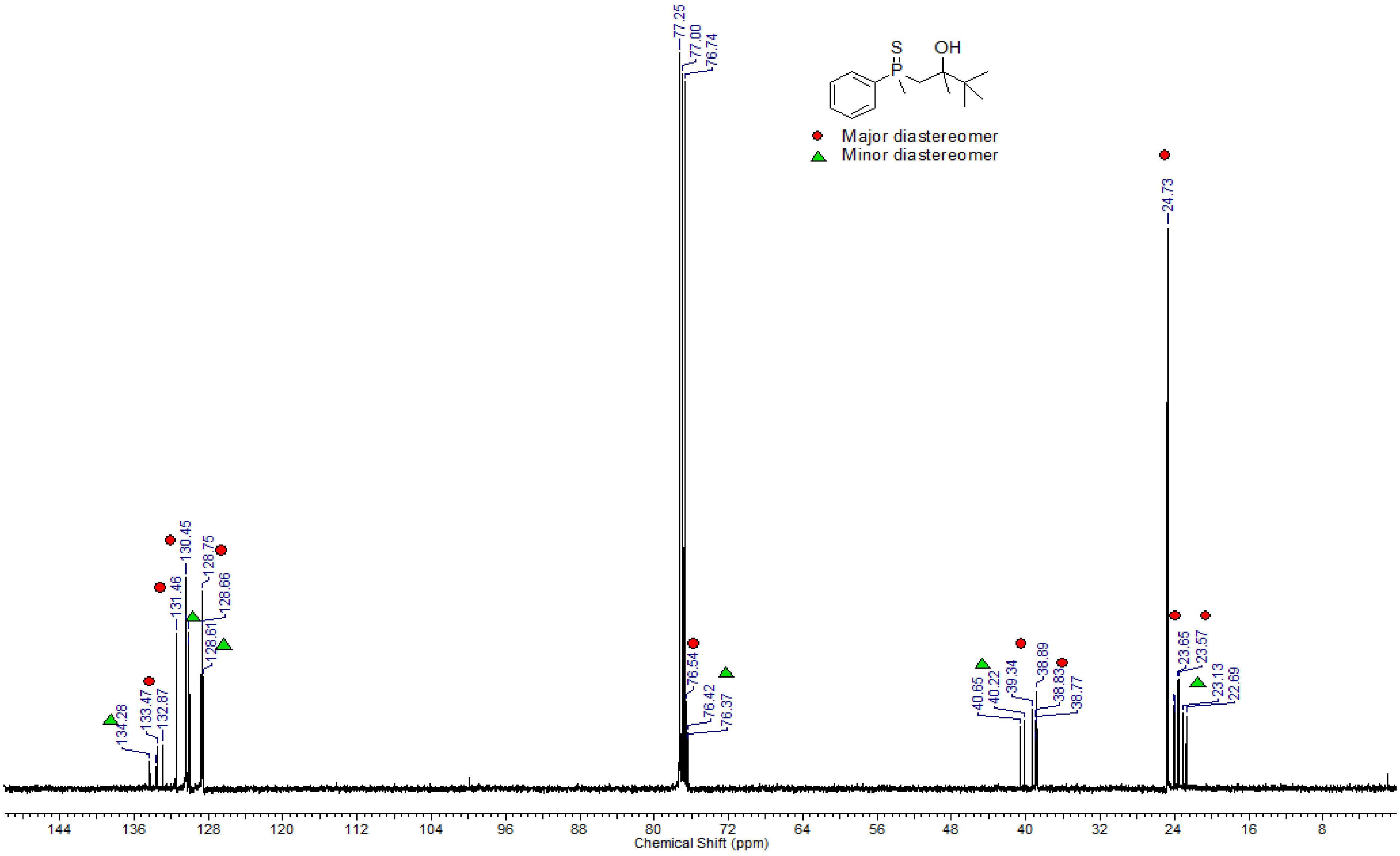

^{13}C NMR spectrum of (2-hydroxy-2,3,3-trimethylbutyl)methylphenylphosphine sulfide (**17**) (CDCl3, 126 MHz).

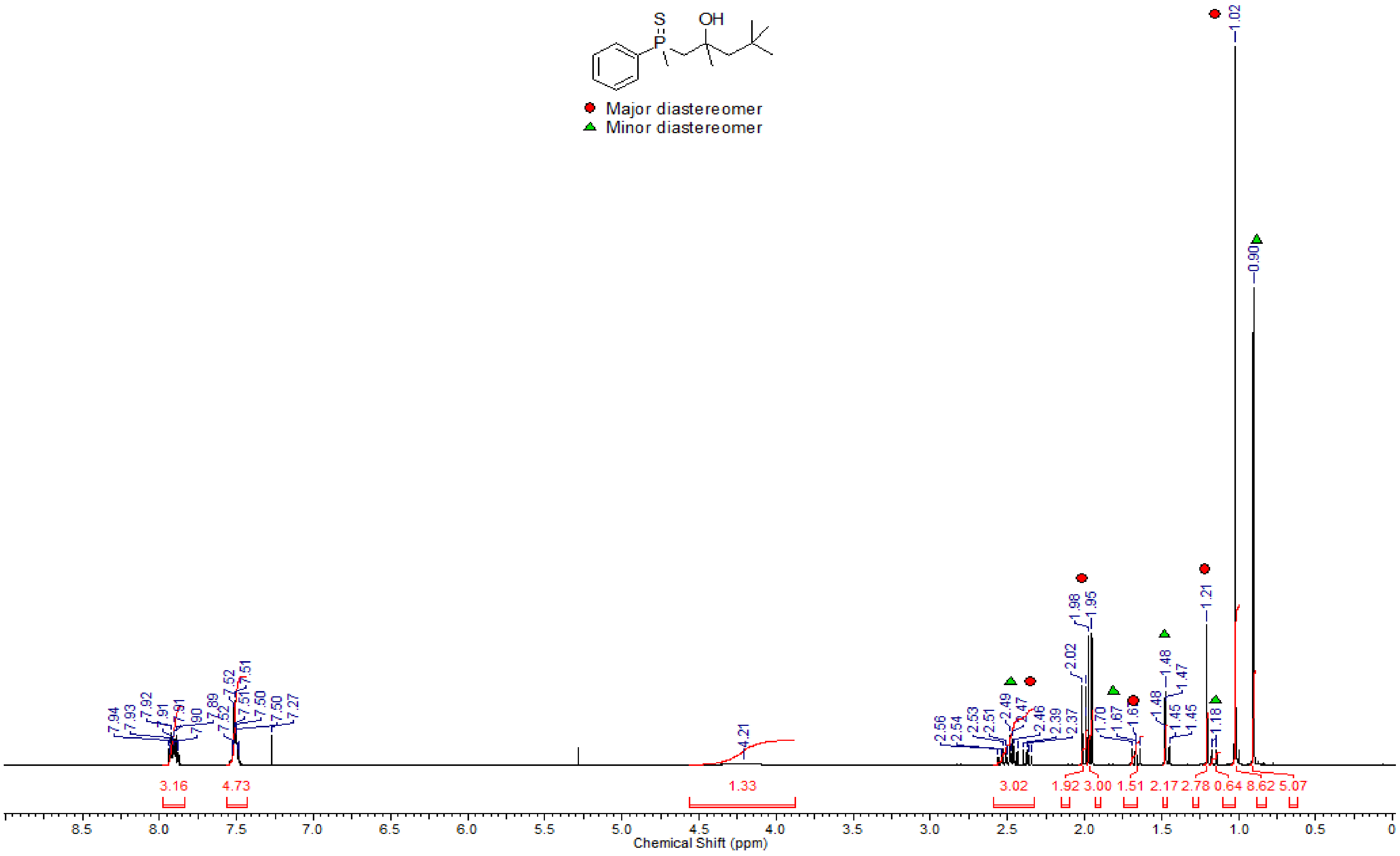

^{1}H NMR spectrum of (2-hydroxy-2,4,4-trimethylpentyl)methylphenylphosphine sulfide (**18**) (CDCl3, 500 MHz).

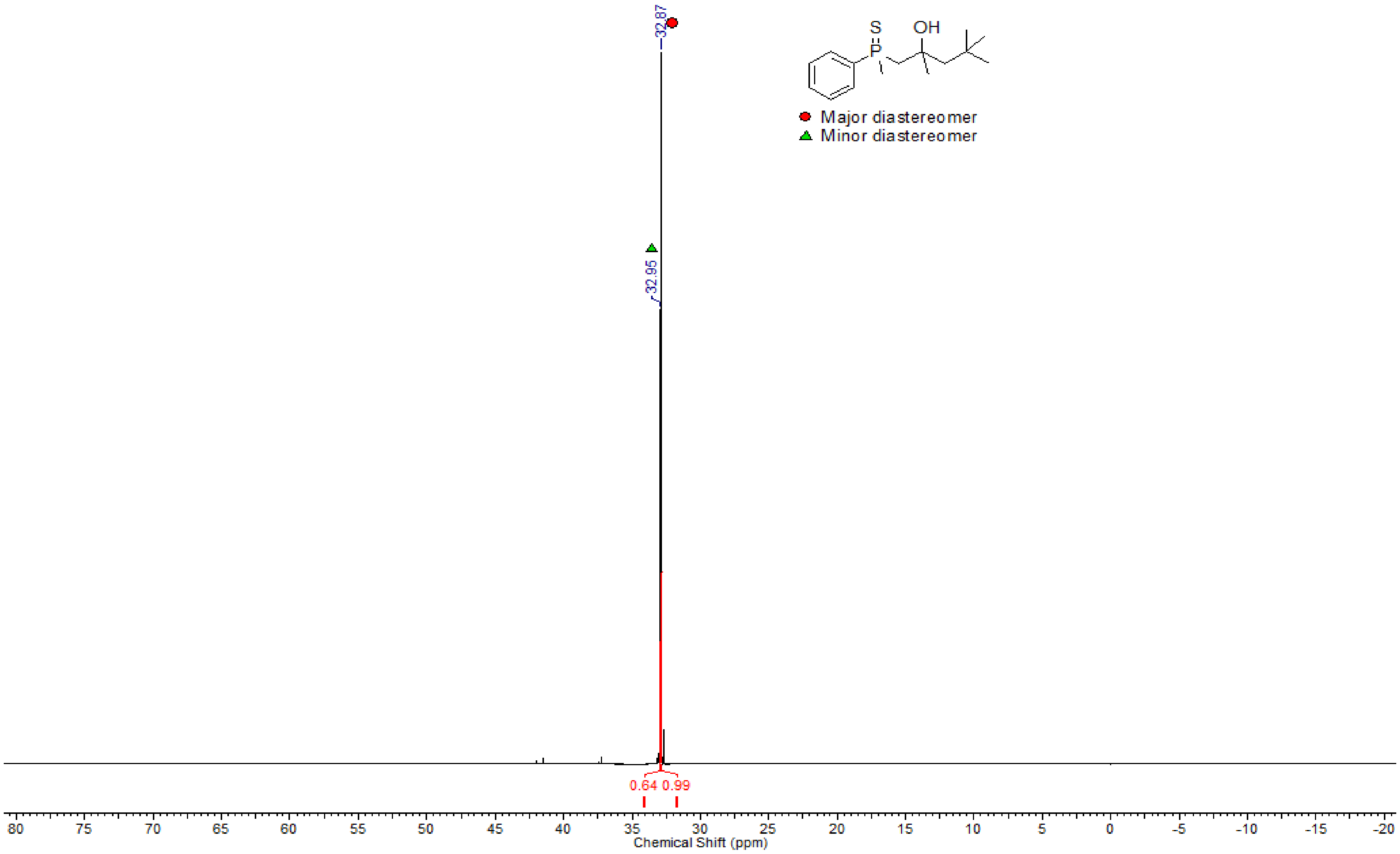

^{31}P NMR spectrum of (2-hydroxy-2,4,4-trimethylpentyl)methylphenylphosphine sulfide (**18**) (CDCl3, 202 MHz).

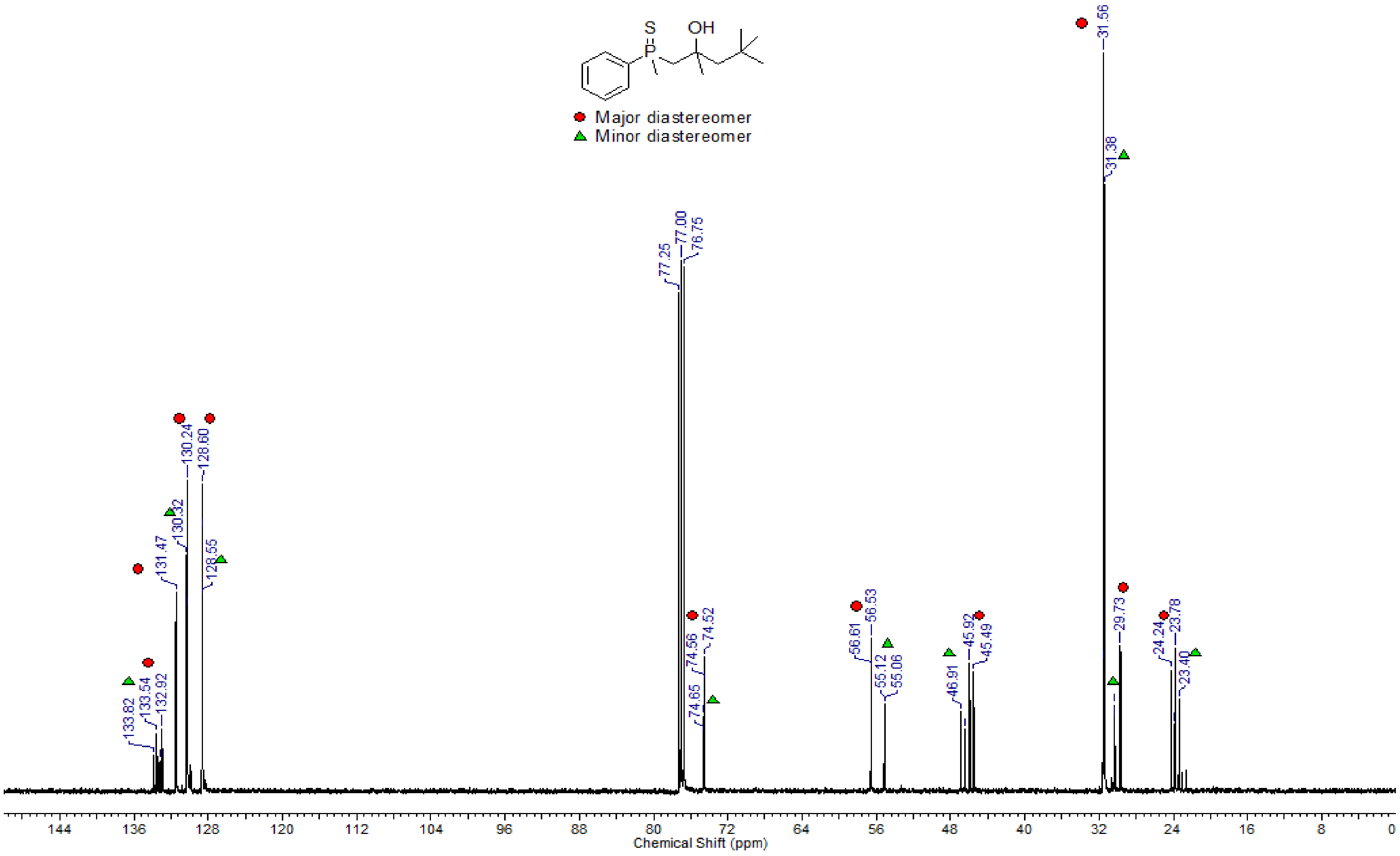

^{13}C NMR spectrum of (2-hydroxy-2,4,4-trimethylpentyl)methylphenylphosphine sulfide (**18**) (CDCl3, 126 MHz).

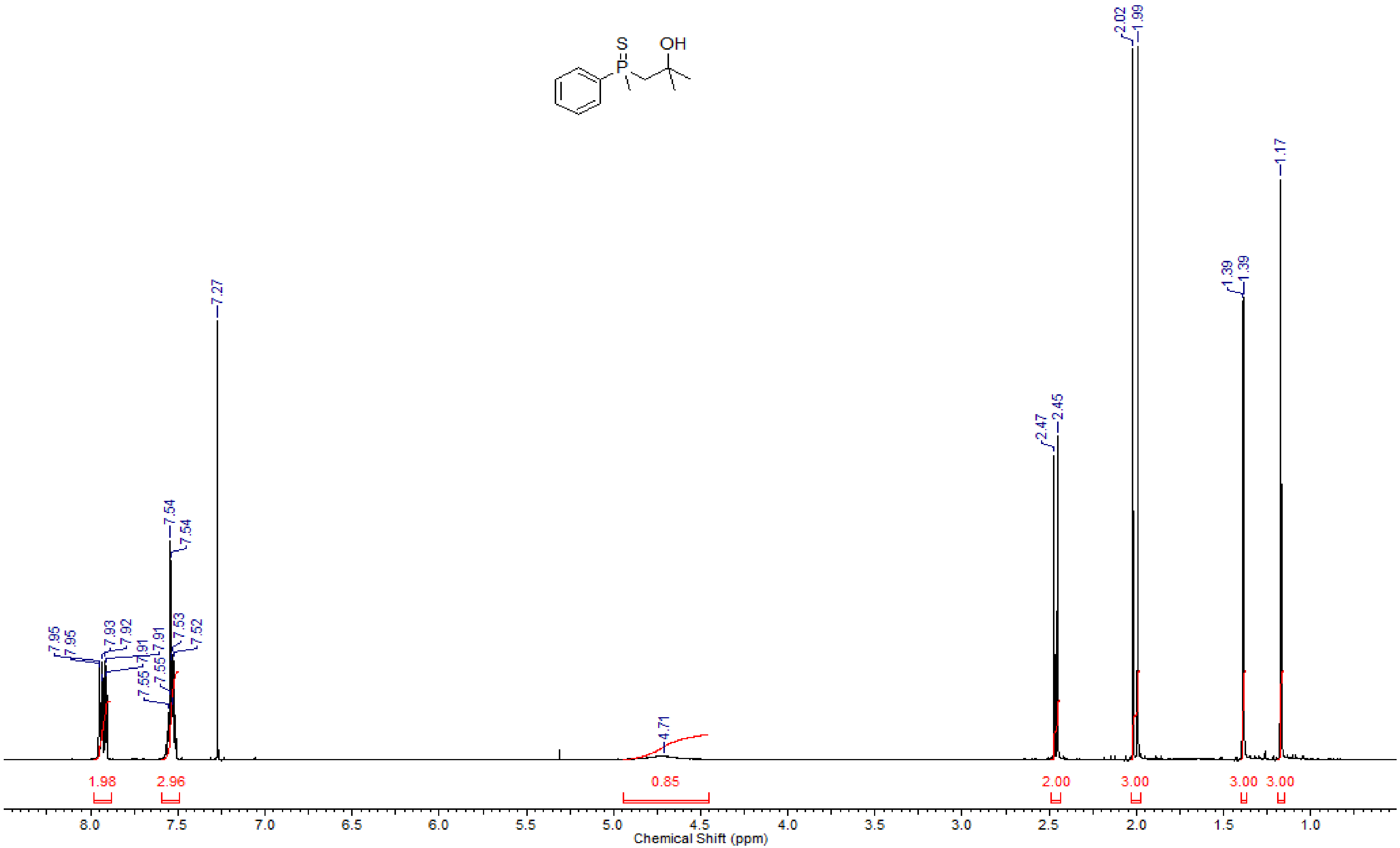

^{1}H NMR spectrum of (2-hydroxy-2-methylpropyl)methylphenylphosphine sulfide (**19**) ($CDCl3$, 500 MHz).

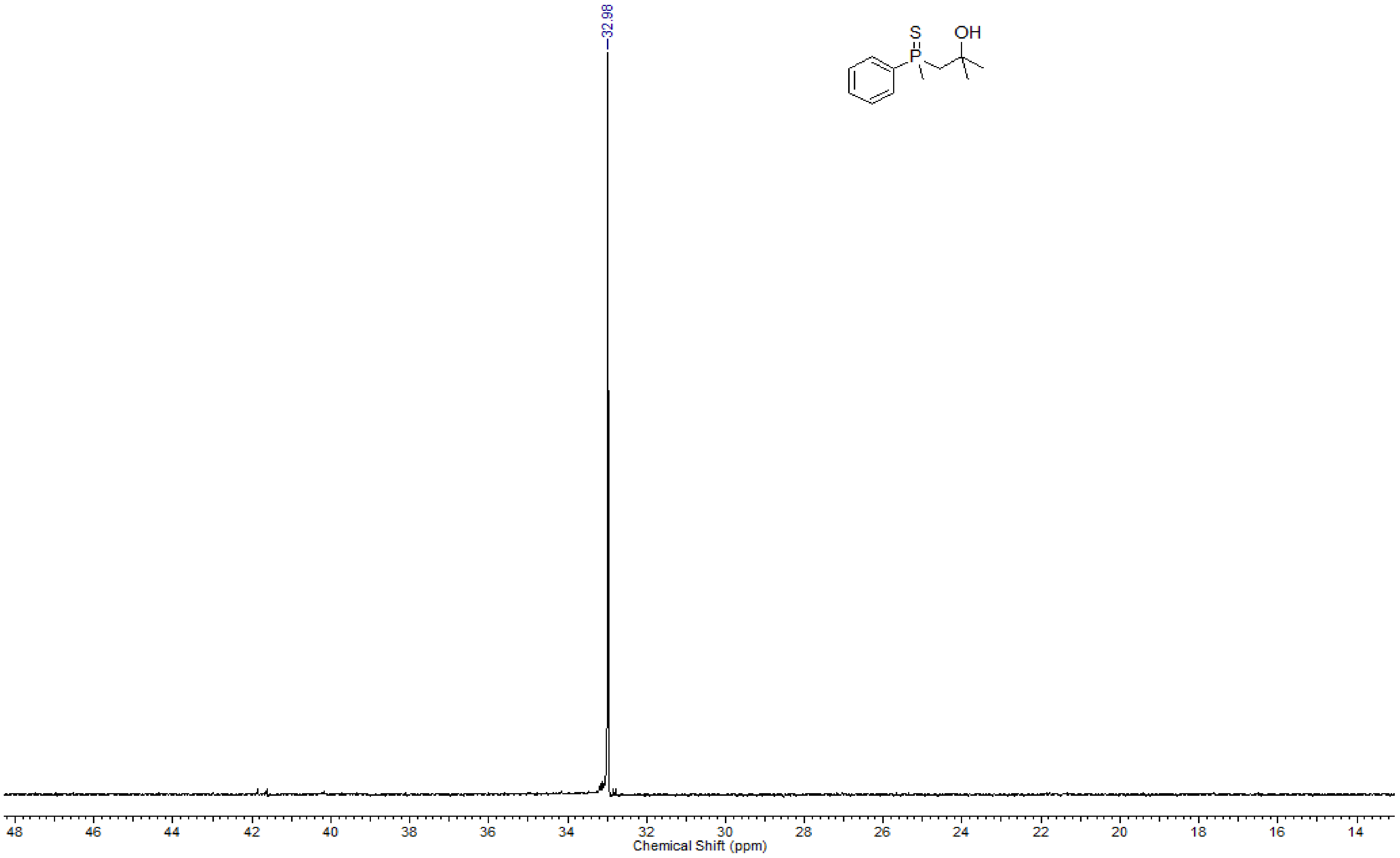

^{31}P NMR spectrum of (2-hydroxy-2-methylpropyl)methylphenylphosphine sulfide (**19**) (CDCl3, 202 MHz).

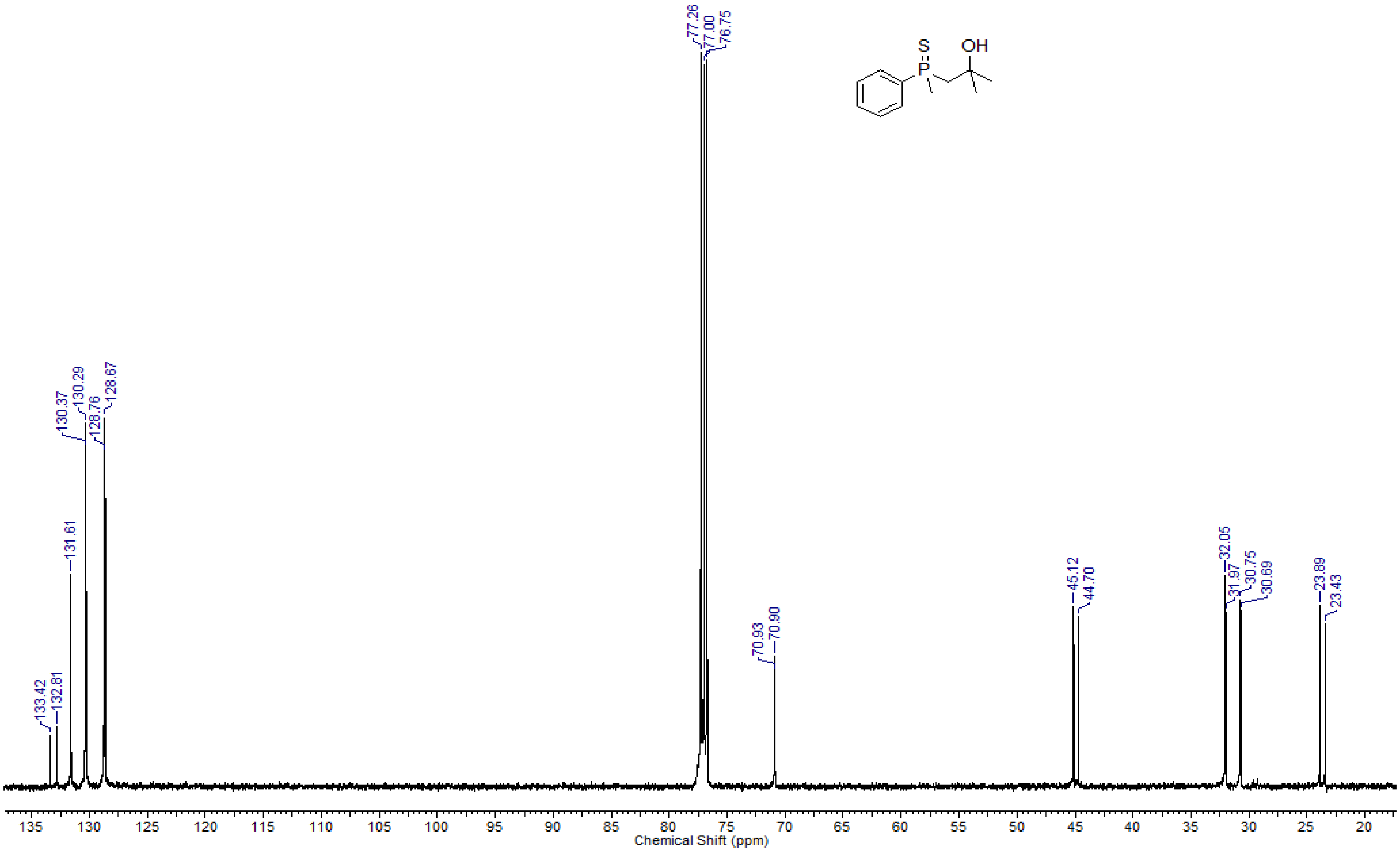

^{13}C NMR spectrum of (2-hydroxy-2-methylpropyl)methylphenylphosphine sulfide (**19**) (CDCl3, 126 MHz).

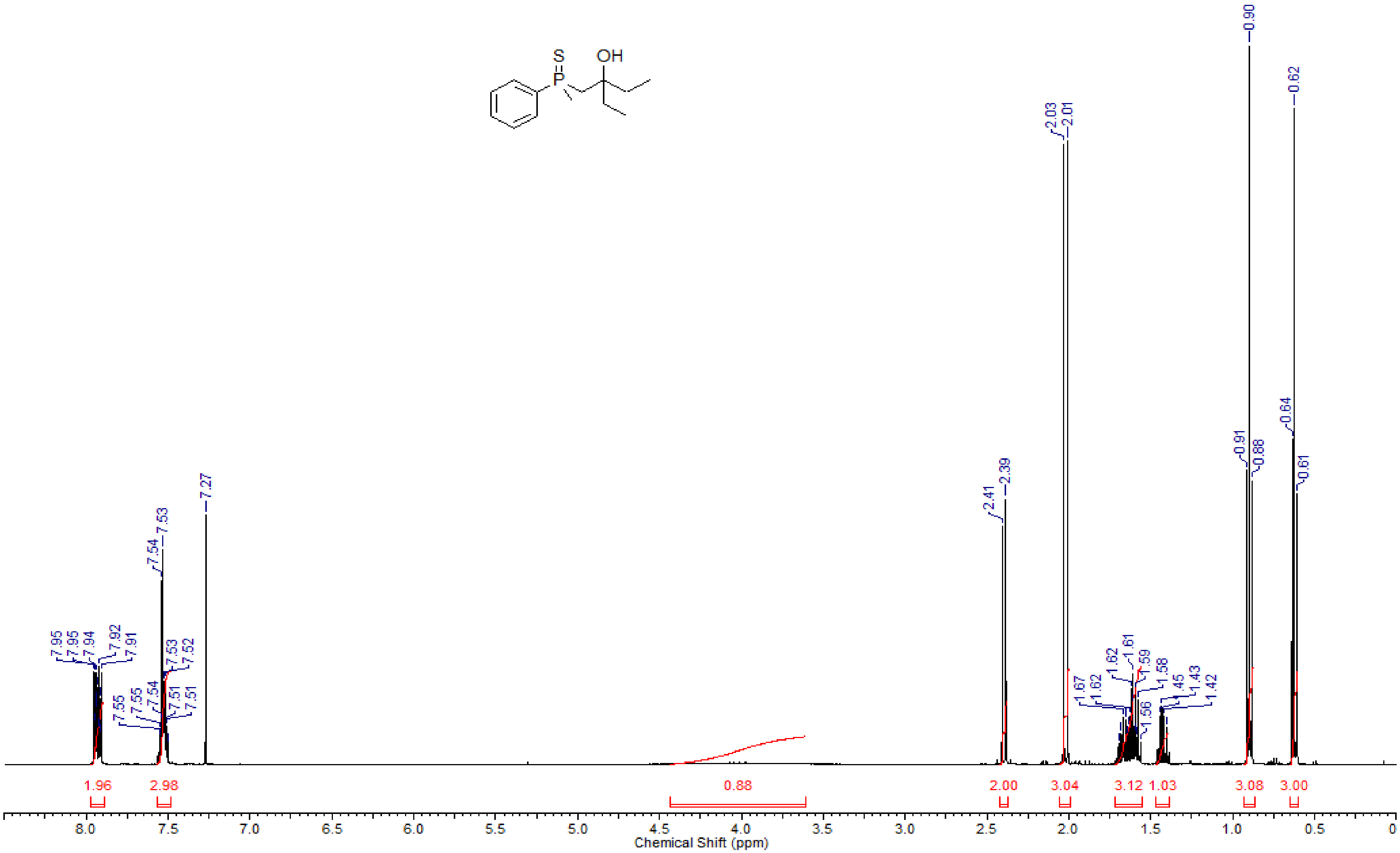

^{1}H NMR spectrum of (2-ethyl-2-hydroxybutyl)methylphenylphosphine sulfide (**20**) (CDCl3, 500 MHz).

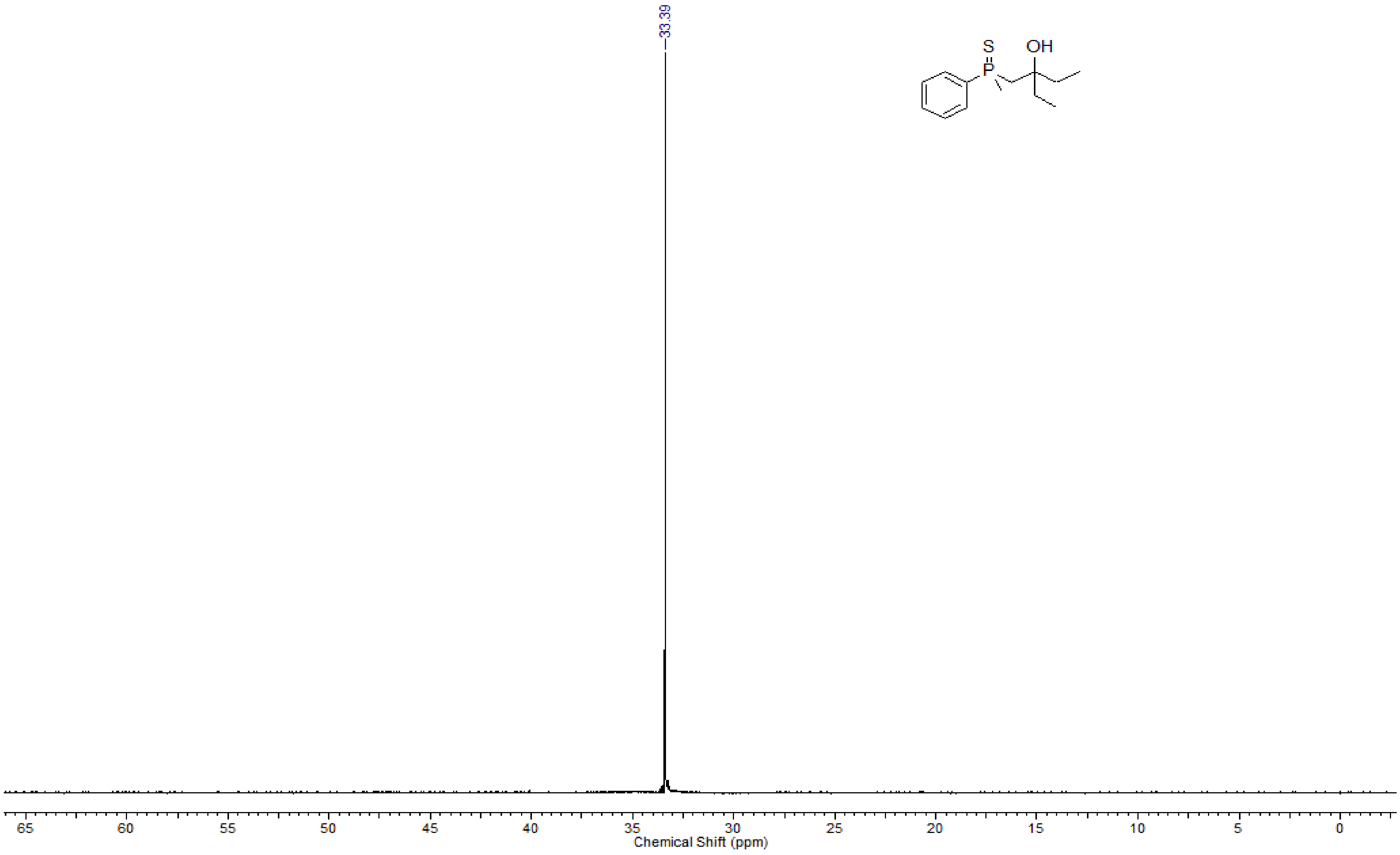

^{31}P NMR spectrum of (2-ethyl-2-hydroxybutyl)methylphenylphosphine sulfide (**20**) (CDCl3, 202 MHz).

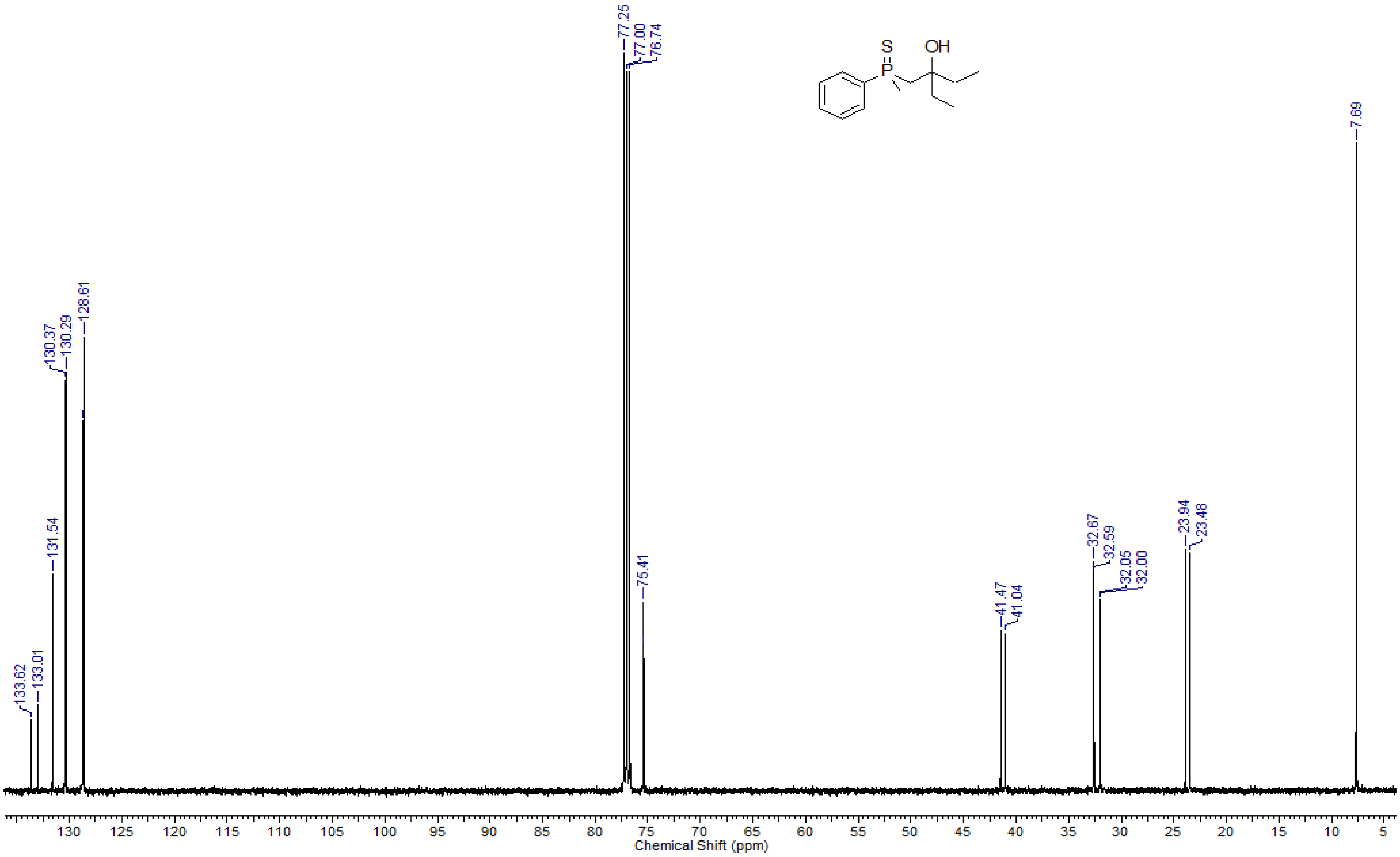

^{13}C NMR spectrum of (2-ethyl-2-hydroxybutyl)methylphenylphosphine sulfide (**20**) (CDCl3, 126 MHz).

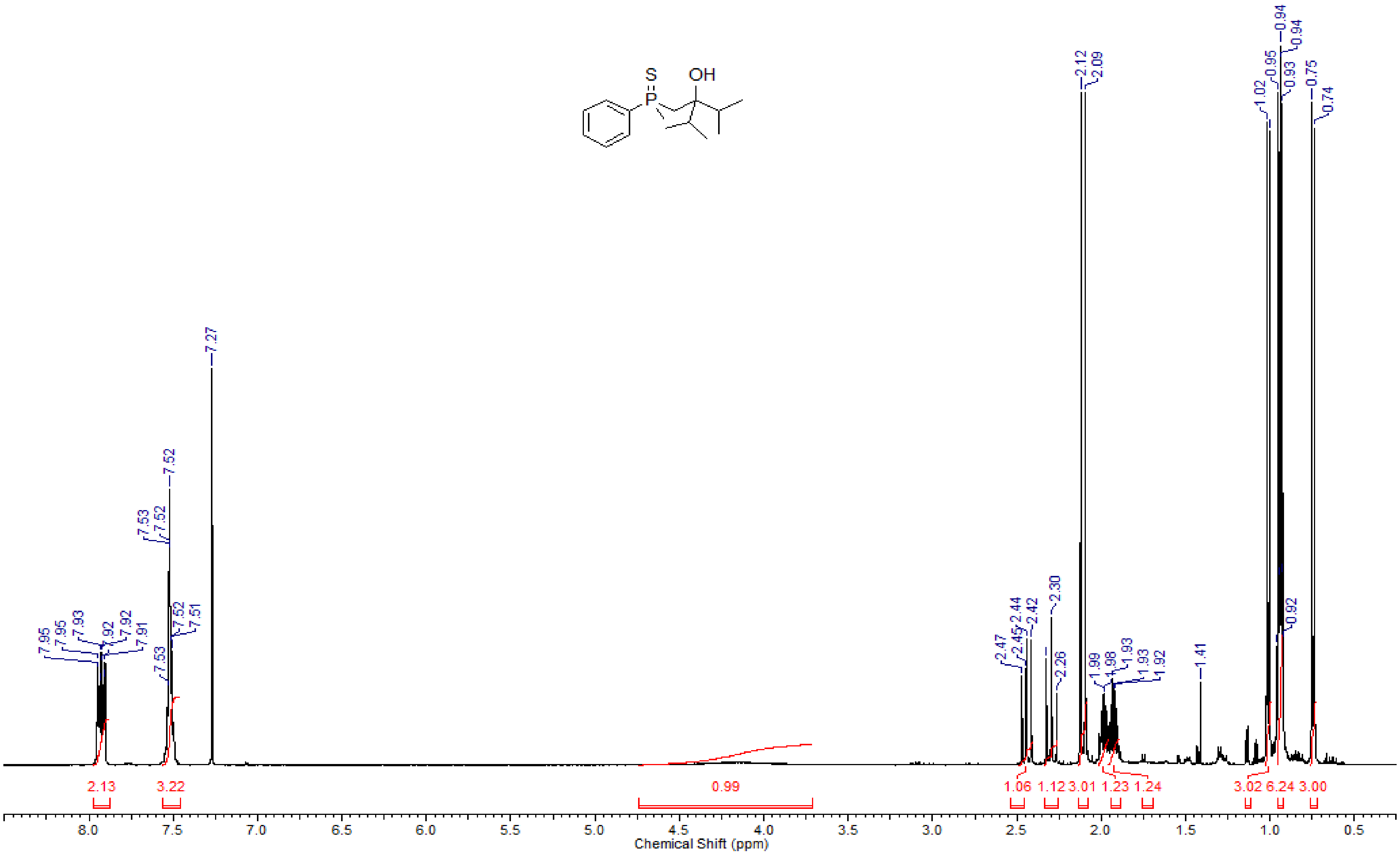

^{1}H NMR spectrum of (2-hydroxy-2-*iso*-propyl-3-methylbutyl)methylphenylphosphine sulfide (**21**) (CDCl3, 500 MHz).

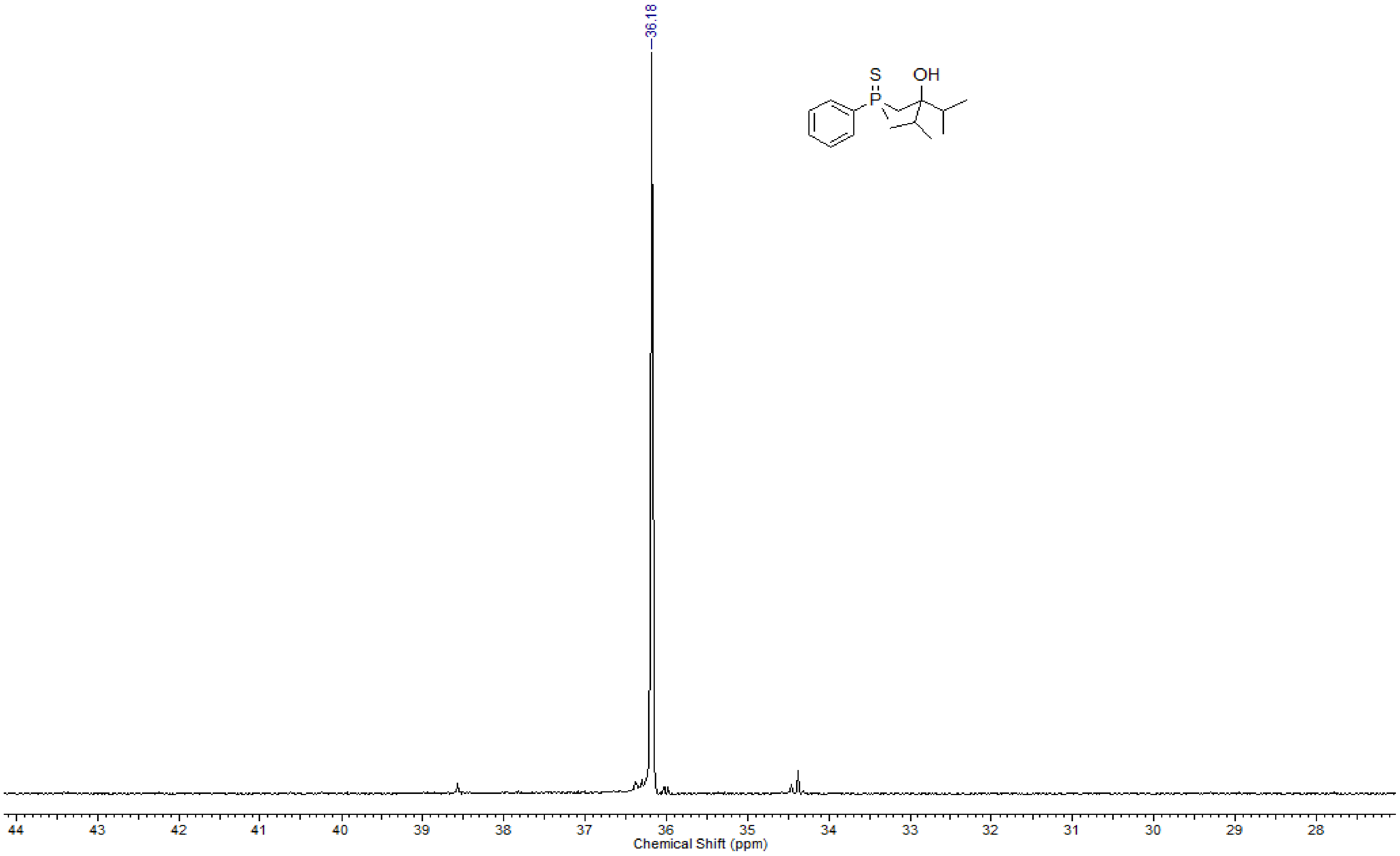

^{31}P NMR spectrum of (2-hydroxy-2-*iso*-propyl-3-methylbutyl)methylphenylphosphine sulfide (**21**) (CDCl3, 202 MHz).

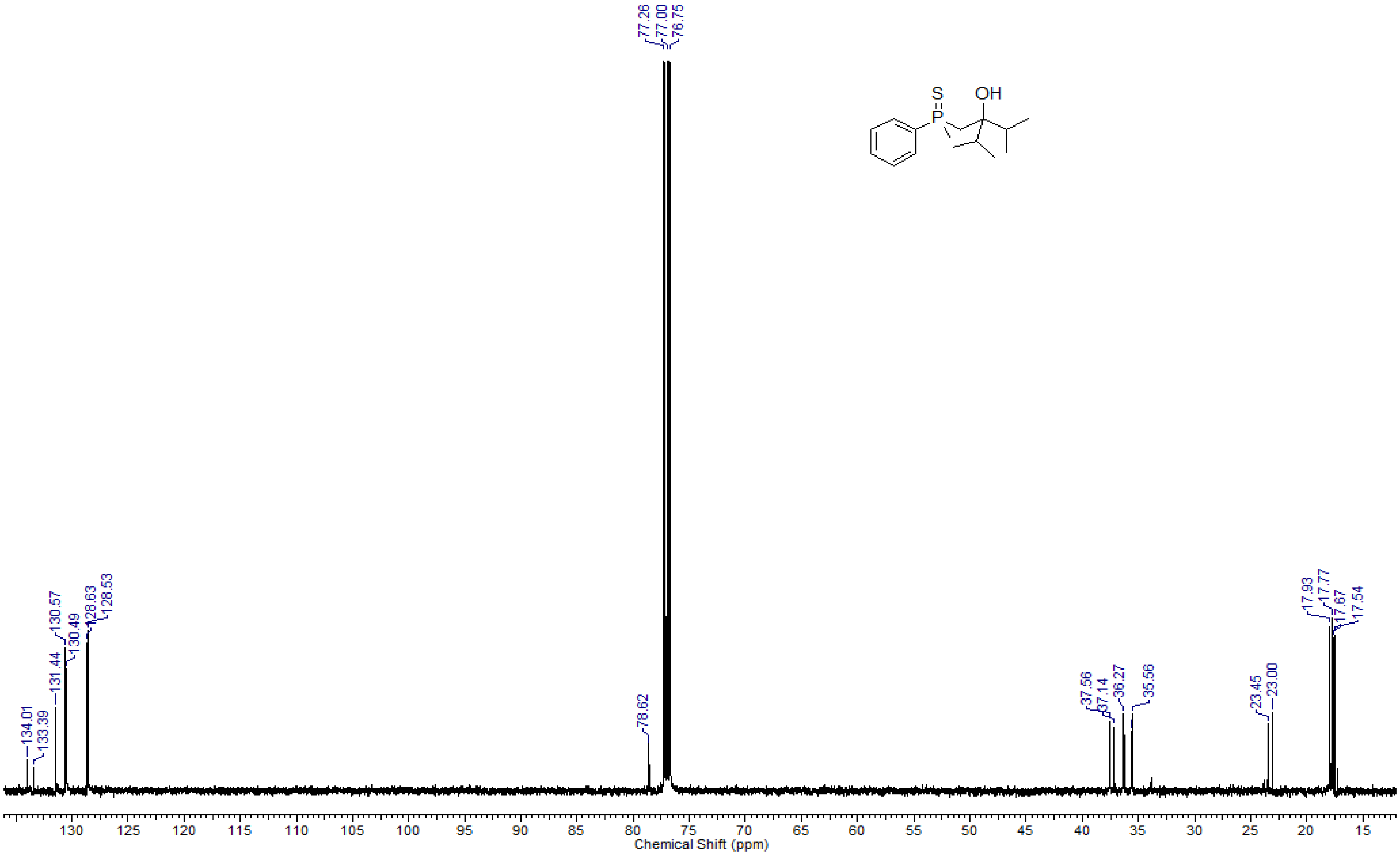

^{13}C NMR spectrum of (2-hydroxy-2-*iso*-propyl-3-methylbutyl)methylphenylphosphine sulfide (**21**) (CDCl3, 126 MHz).

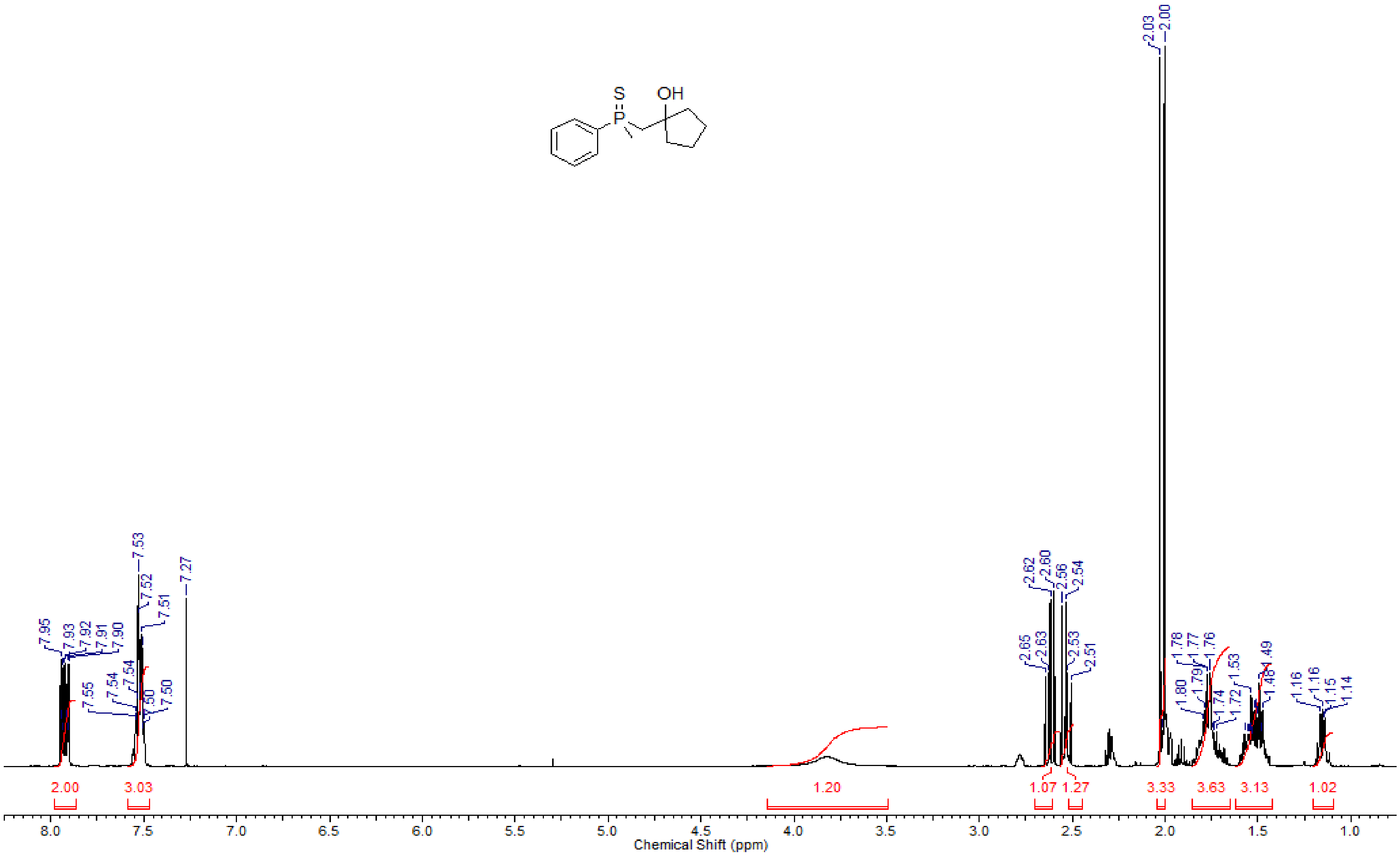

^{1}H NMR spectrum of [(1-hydroxy)cyclopentylmethyl)]methylphenylphosphine sulfide (**22**) (CDCl3, 500 MHz).

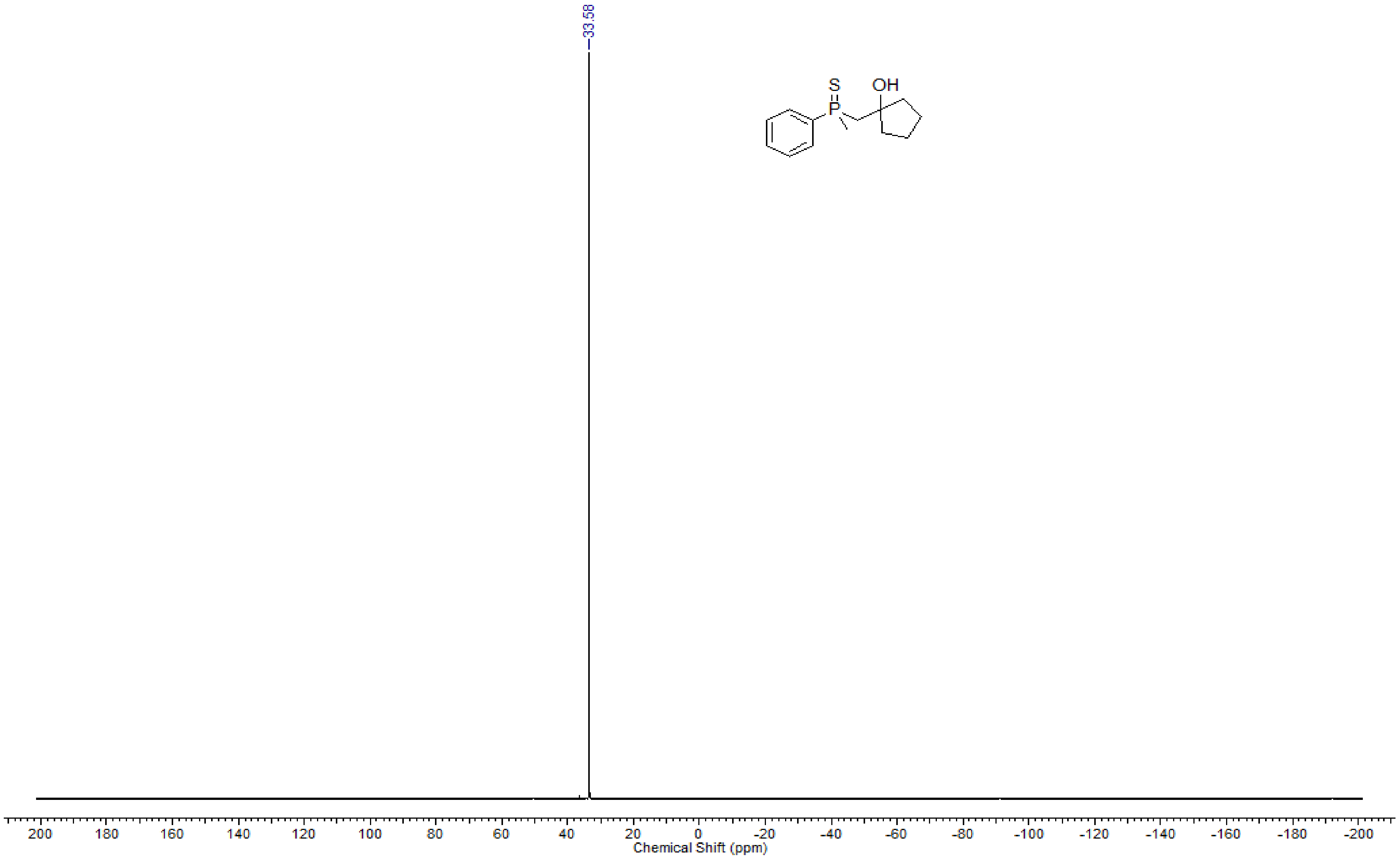

^{31}P NMR spectrum of [(1-hydroxy)cyclopentylmethyl)]methylphenylphosphine sulfide (**22**) (CDCl3, 202 MHz).

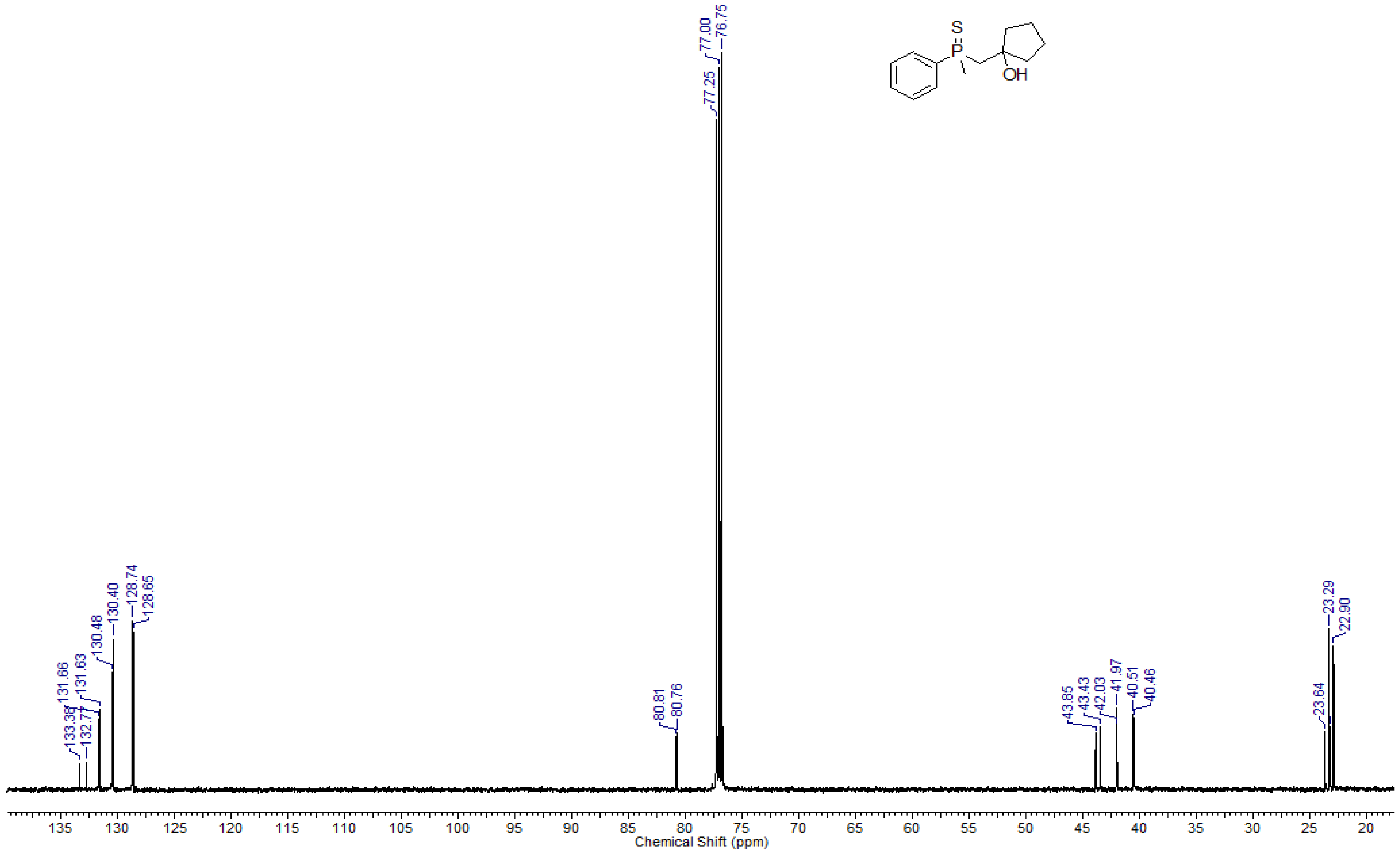

^{13}C NMR spectrum of [(1-hydroxy)cyclopentylmethyl)]methylphenylphosphine sulfide (**22**) (CDCl3, 126 MHz).

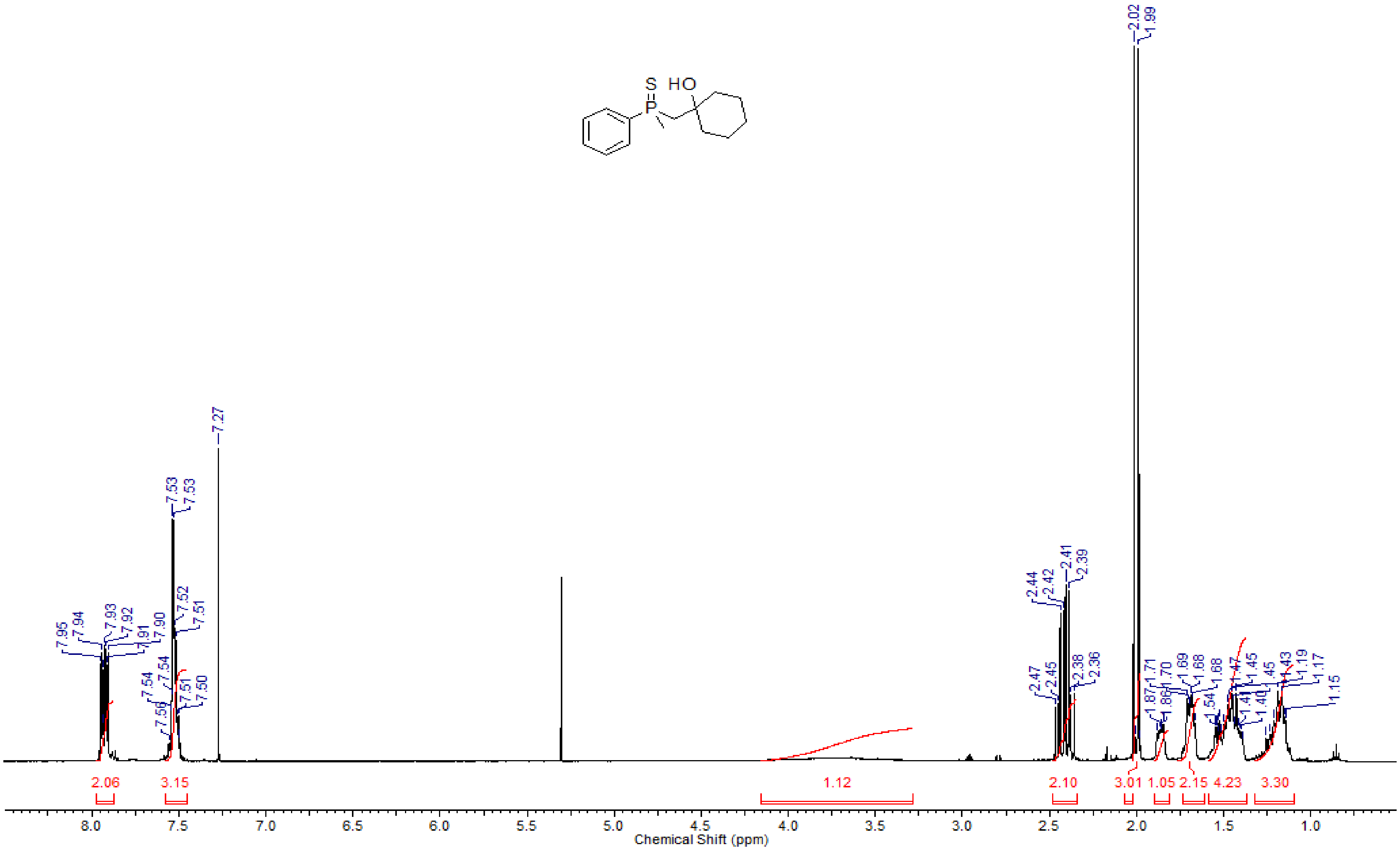

^{1}H NMR spectrum of [(1-hydroxy)cyclohexylmethyl)]methylphenylphosphine sulfide (**23**) (CDCl3, 500 MHz).

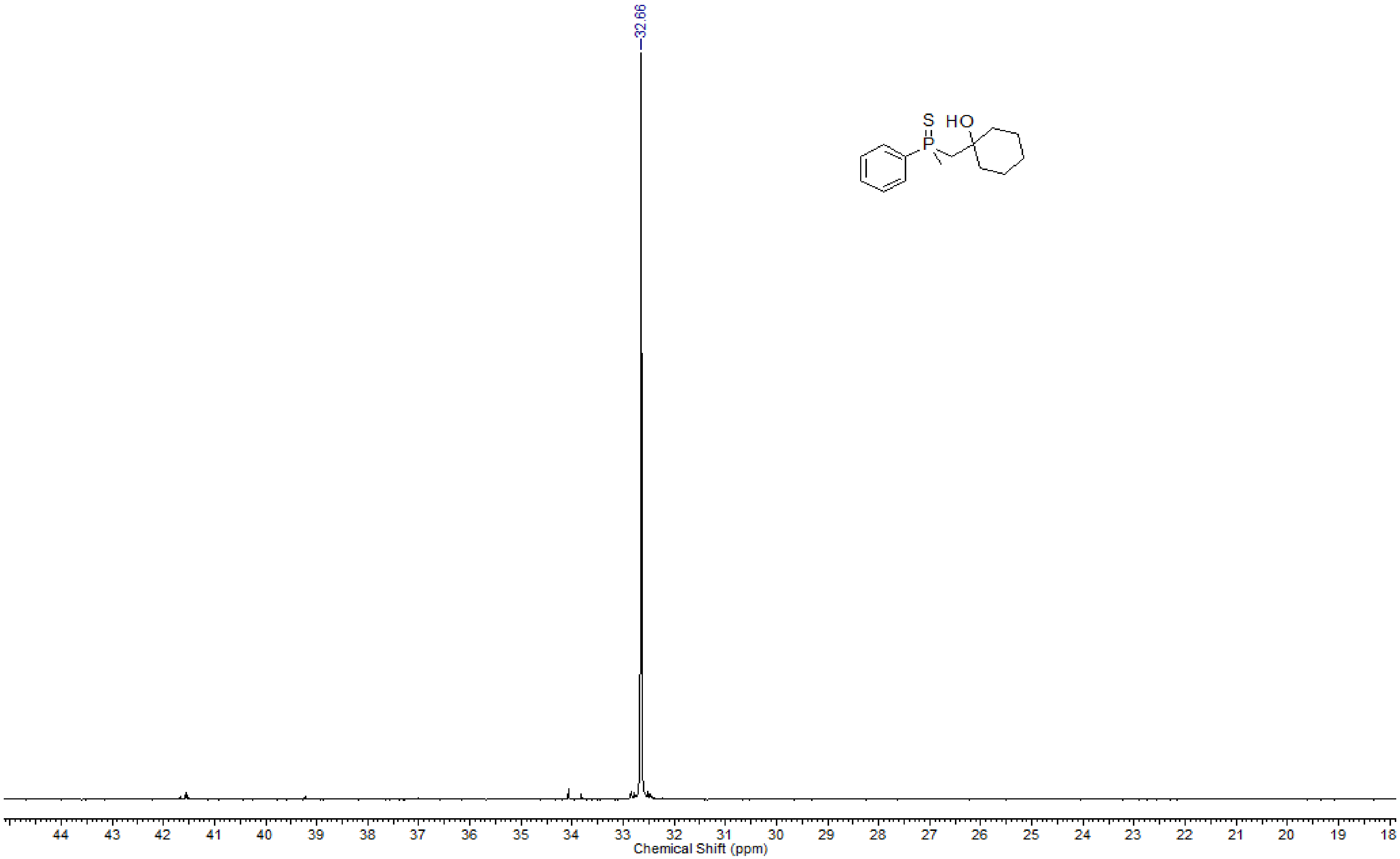

^{31}P NMR spectrum of [(1-hydroxy)cyclohexylmethyl)]methylphenylphosphine sulfide (**23**) (CDCl3, 202 MHz).

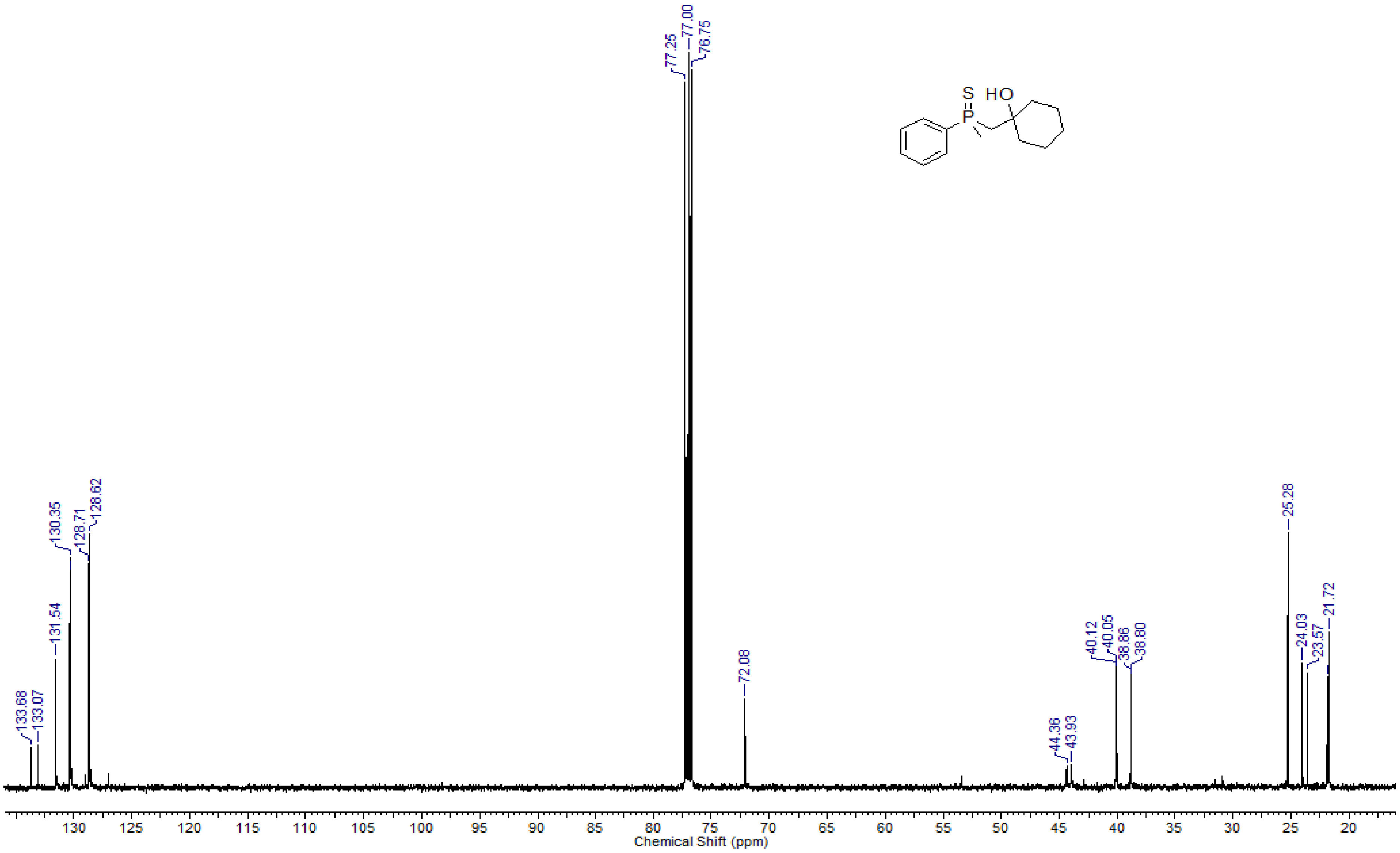

^{13}C NMR spectrum of [(1-hydroxy)cyclohexylmethyl)]methylphenylphosphine sulfide (**23**) (CDCl3, 126 MHz).

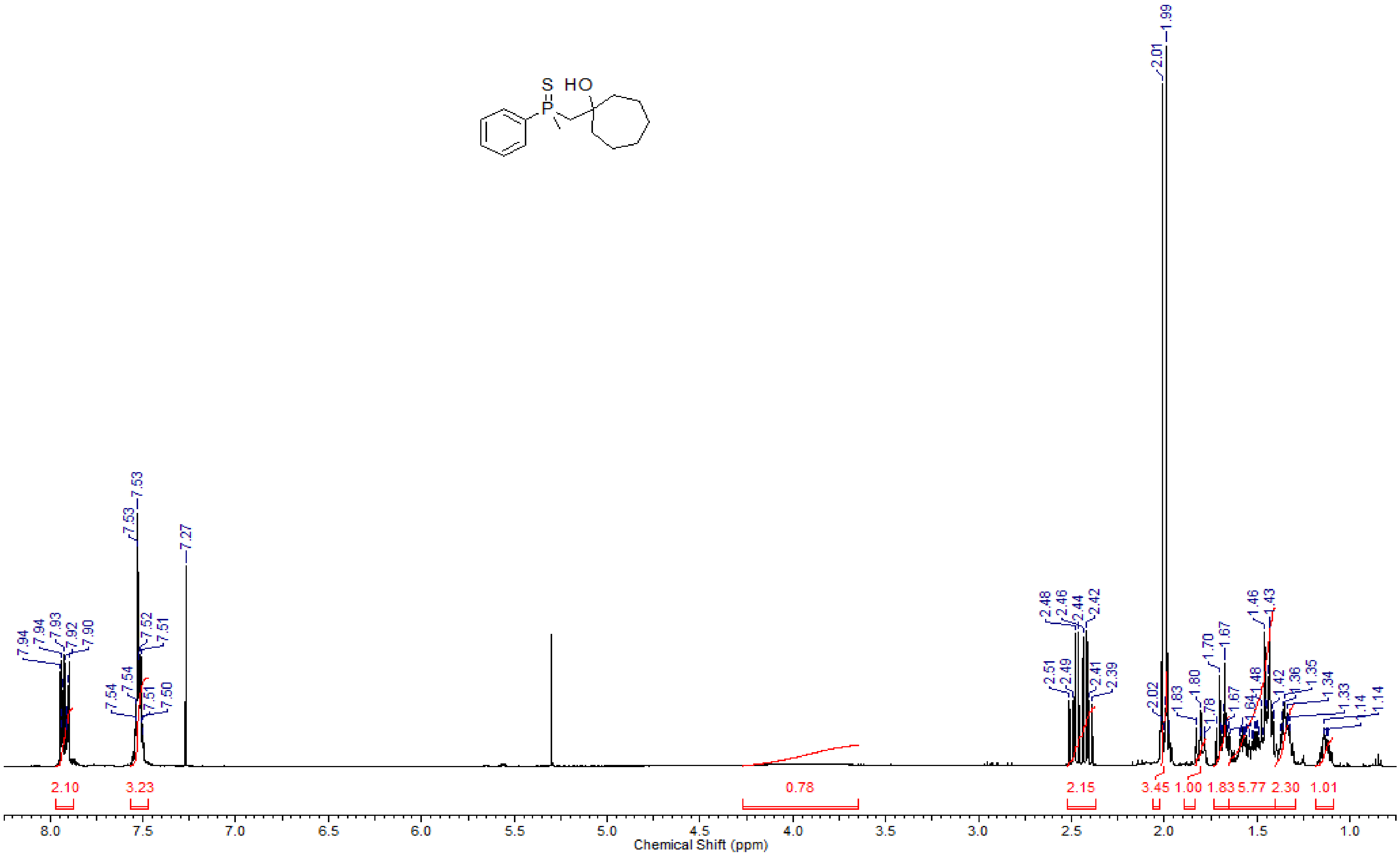

^{1}H NMR spectrum of [(1-hydroxy)cycloheptylmethyl)]methylphenylphosphine sulfide (**24**) (CDCl3, 500 MHz).

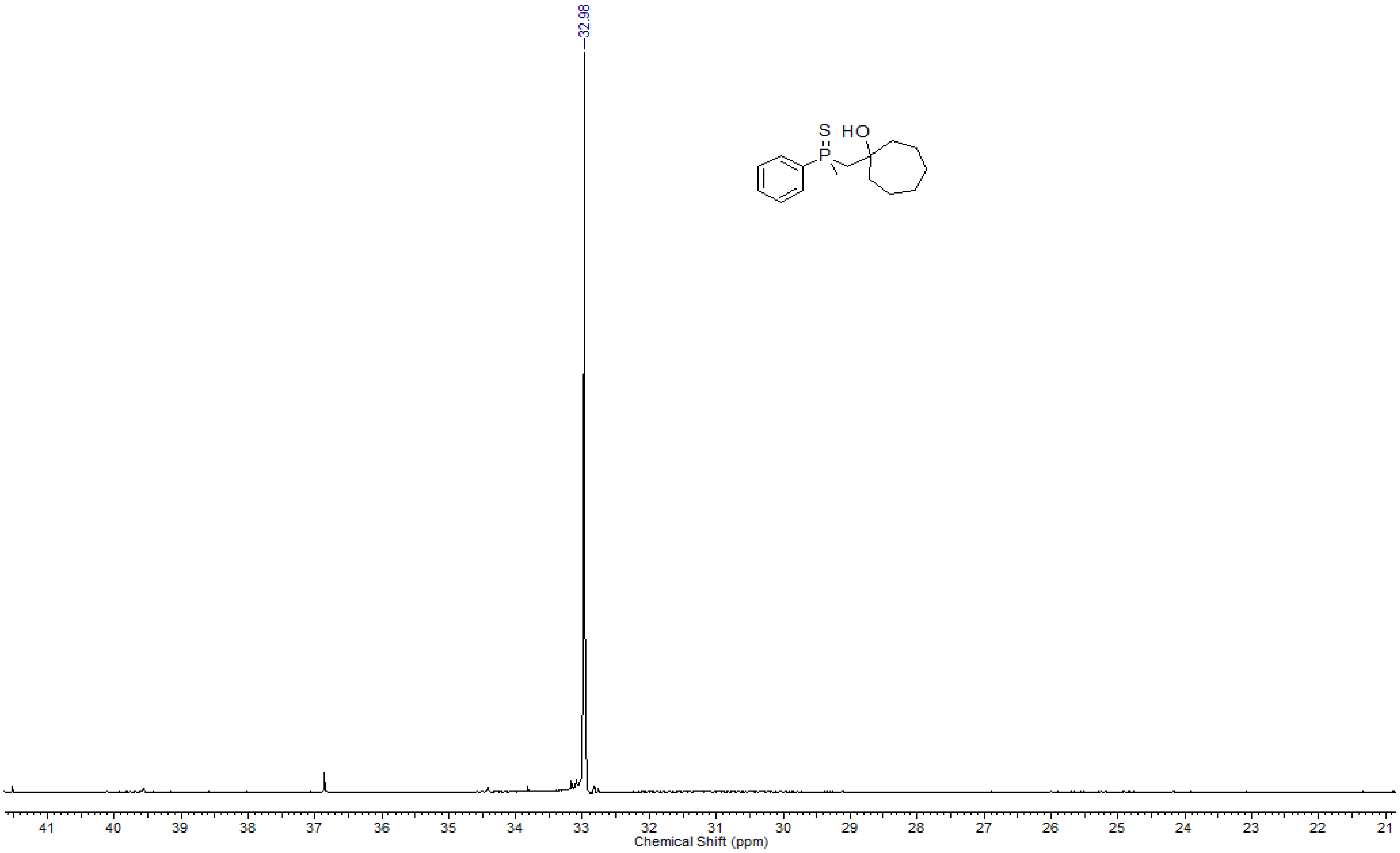

^{31}P NMR spectrum of [(1-hydroxy)cycloheptylmethyl)]methylphenylphosphine sulfide (**24**) (CDCl3, 202 MHz).

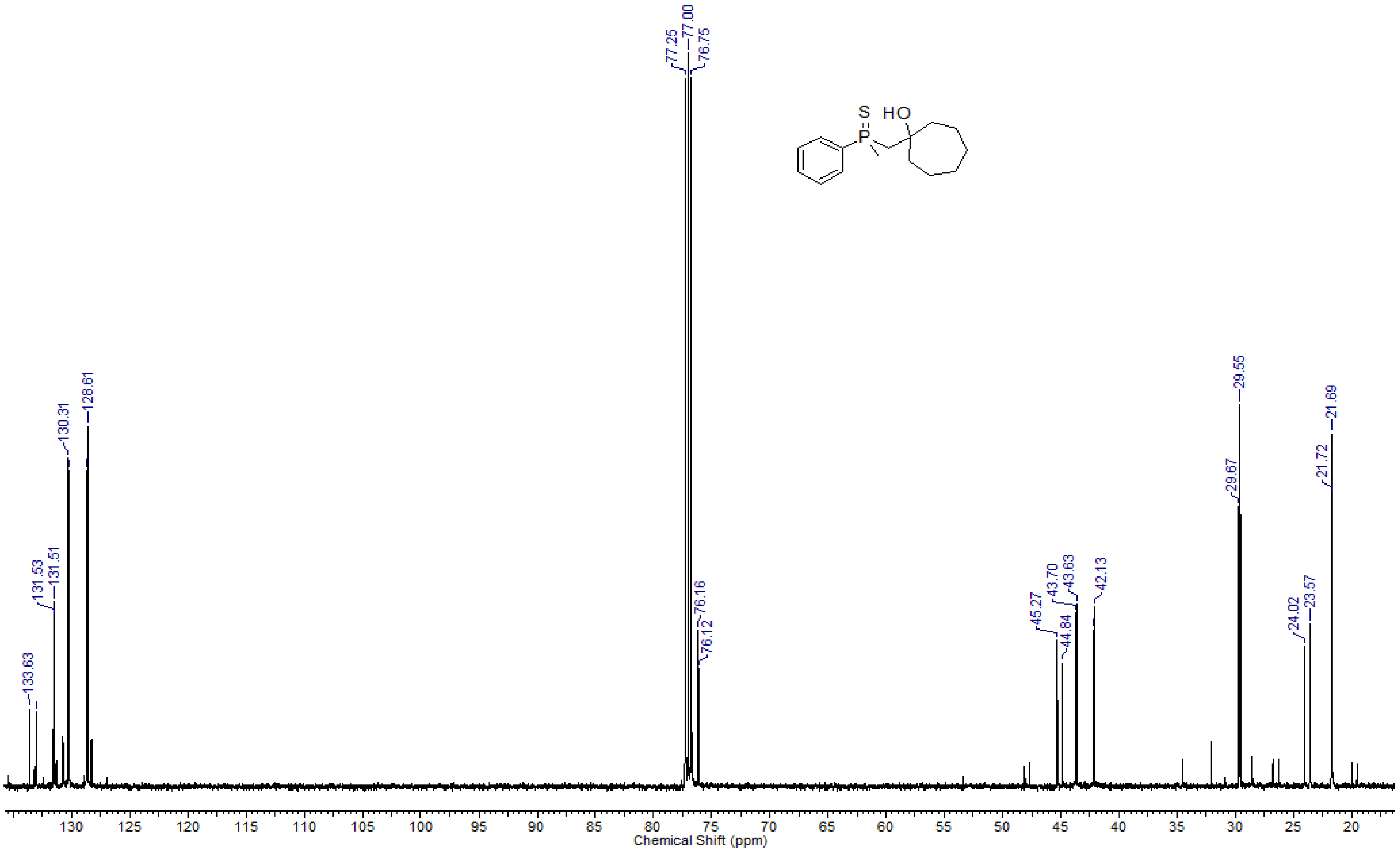

^{13}C NMR spectrum of [(1-hydroxy)cycloheptylmethyl)]methylphenylphosphine sulfide (**24**) (CDCl3, 126 MHz).

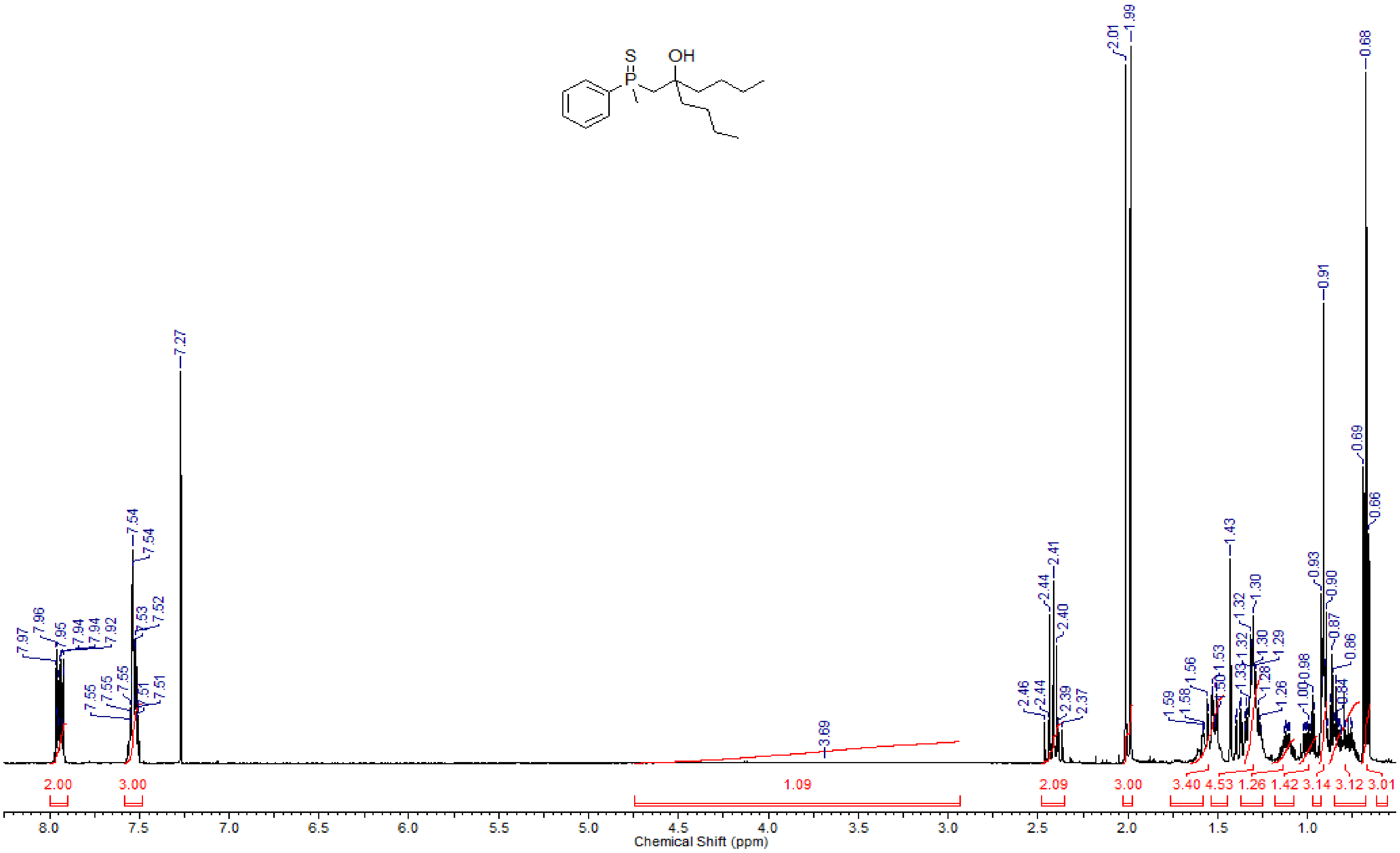

^{1}H NMR spectrum of (2-butyl-2-hydroxyhexyl)methylphenylphosphine sulfide (**25**) ($CDCl_3$, 500 MHz).

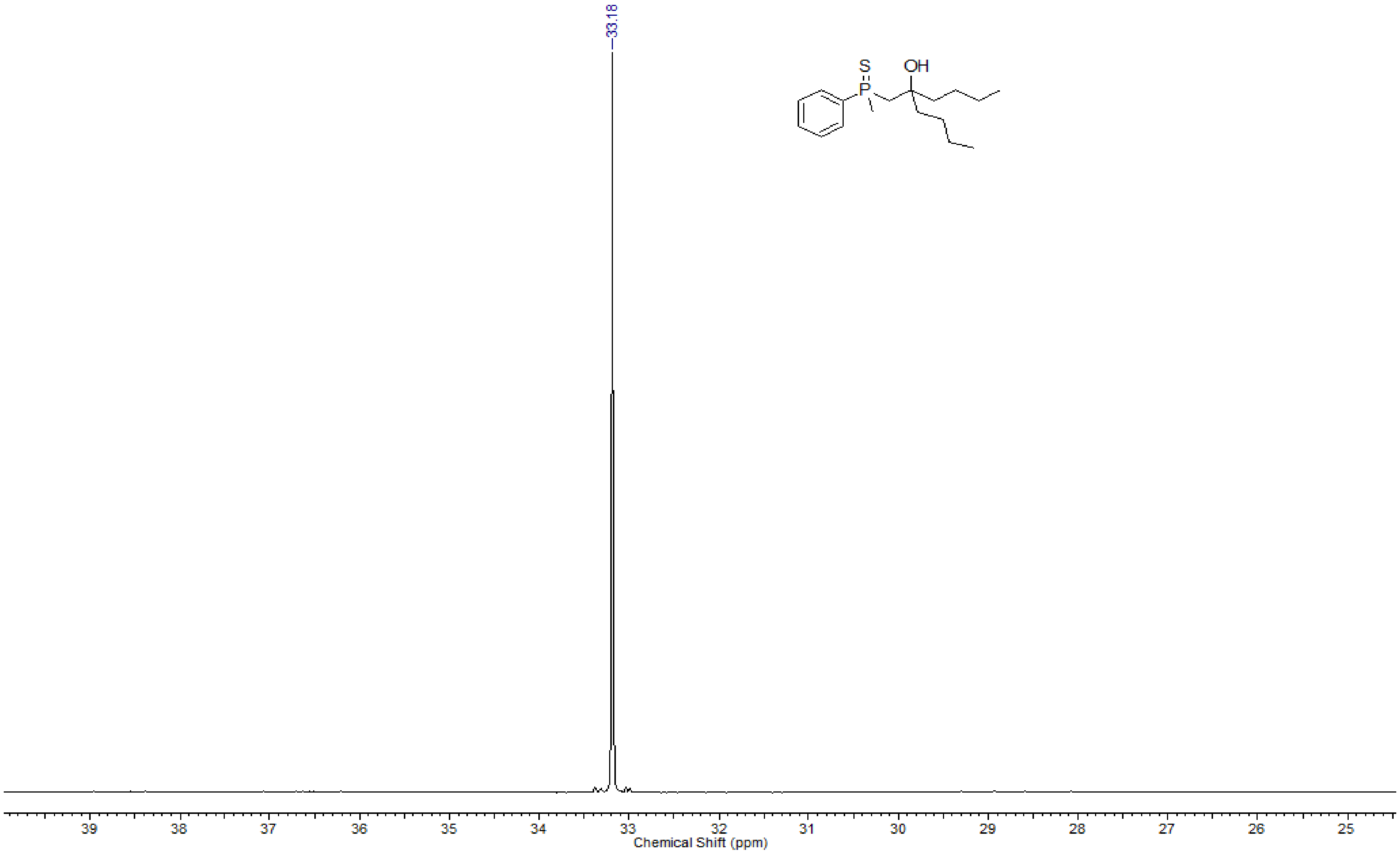

^{31}P NMR spectrum of (2-butyl-2-hydroxyhexyl)methylphenylphosphine sulfide (**25**) (CDCl3, 202 MHz).

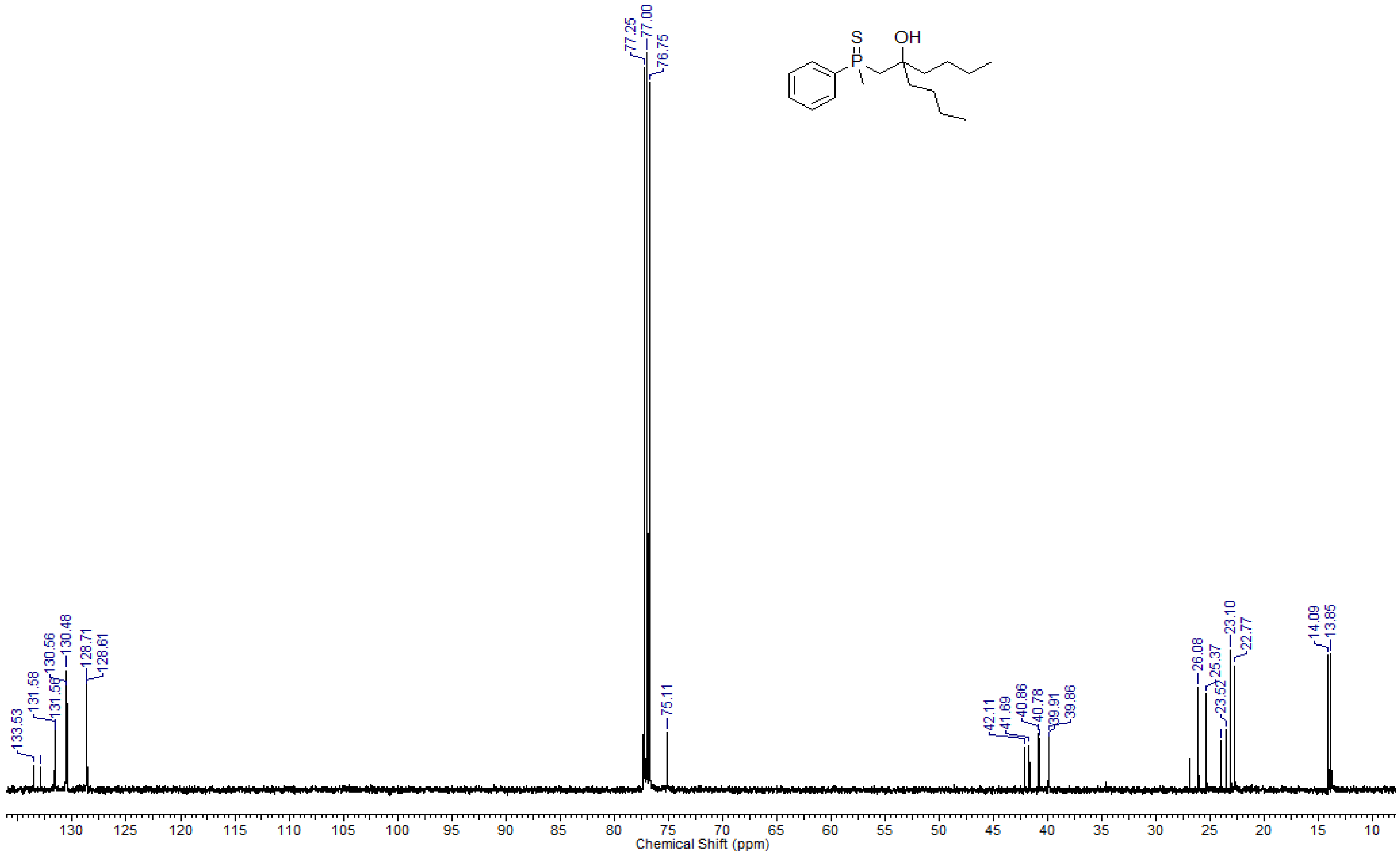

^{13}C NMR spectrum of (2-butyl-2-hydroxyhexyl)methylphenylphosphine sulfide (**25**) (CDCl3, 126 MHz).

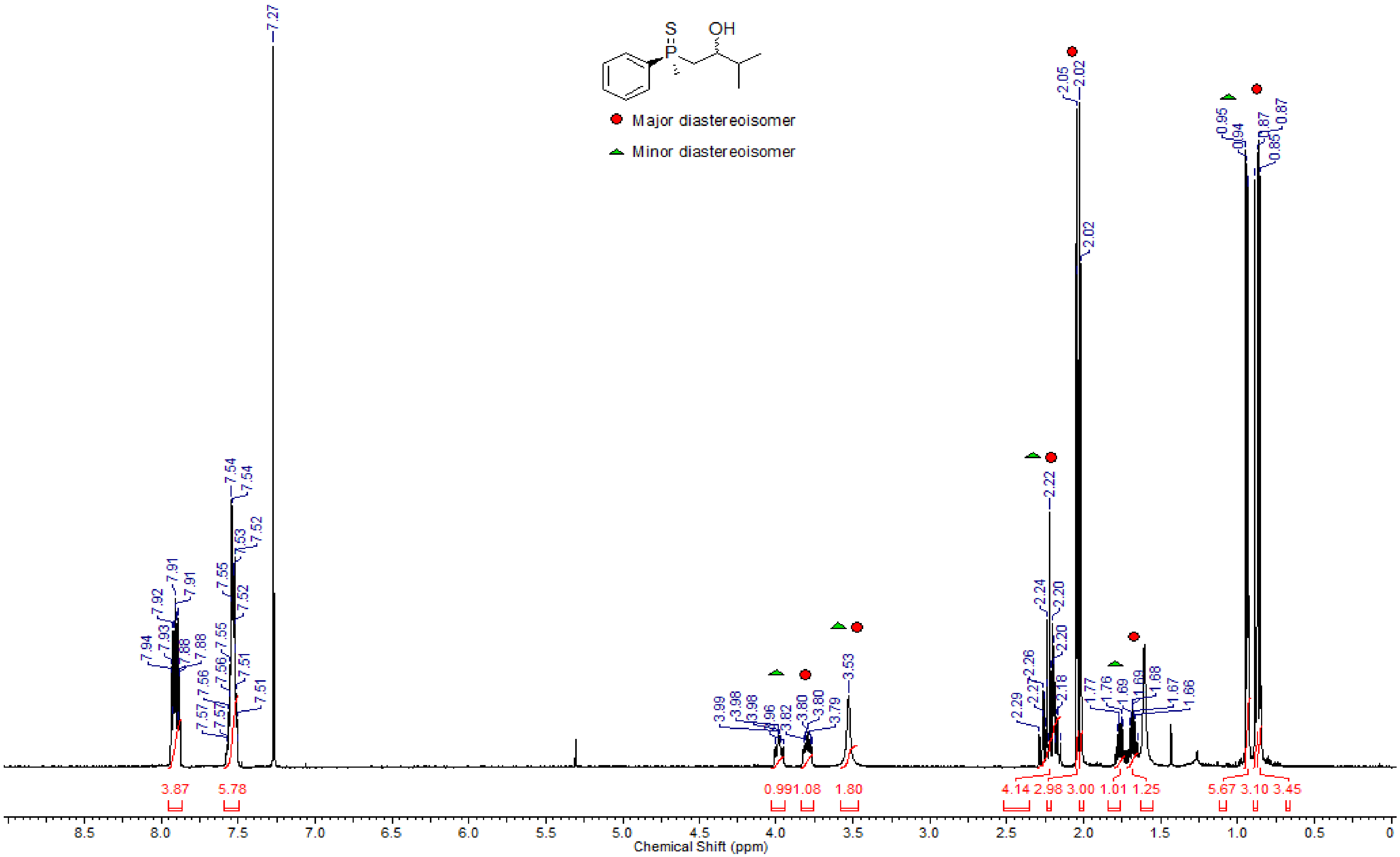

^{1}H NMR spectrum of (S_P)-(2-hydroxy-3-methylbutyl)methylphenylphosphine sulfide (S_P)-(**8**) (CDCl3, 500 MHz).

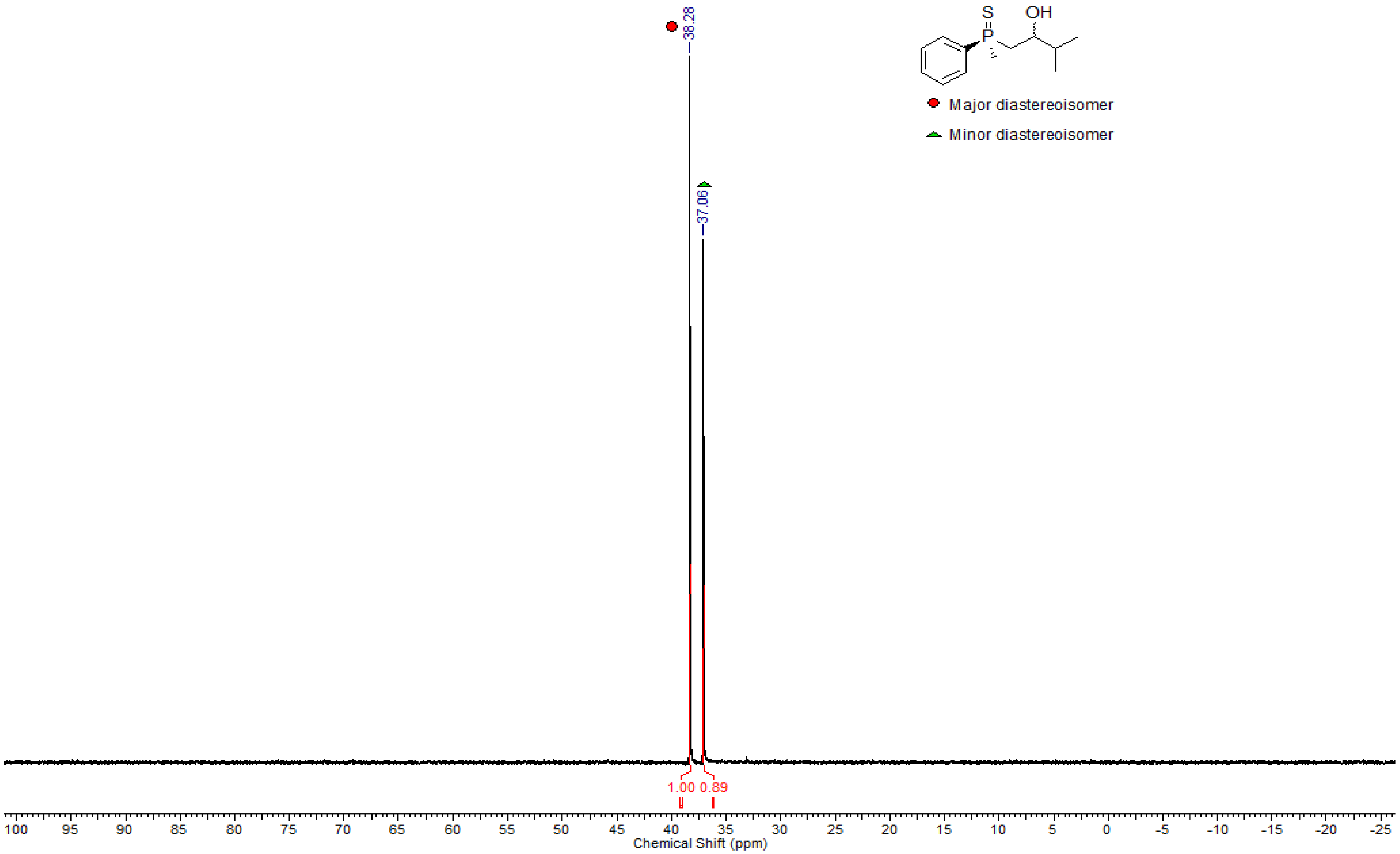

^{31}P NMR spectrum of (S_P)-(2-hydroxy-3-methylbutyl)methylphenylphosphine sulfide (S_P)-(**8**) (CDCl3, 202 MHz).

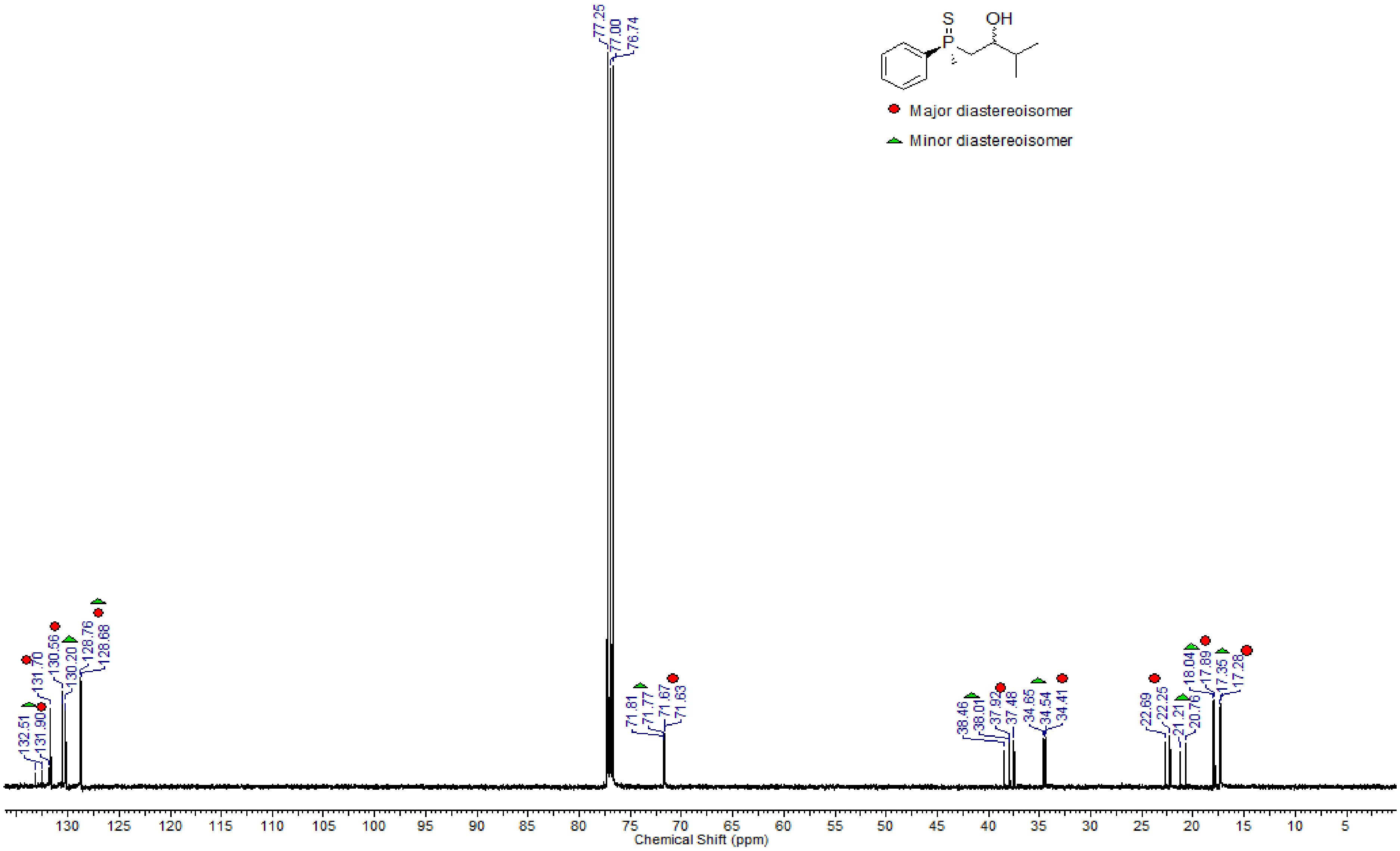

^{13}C NMR spectrum of (S_P)-(2-hydroxy-3-methylbutyl)methylphenylphosphine sulfide (S_P)-(**8**) (CDCl3, 126 MHz).

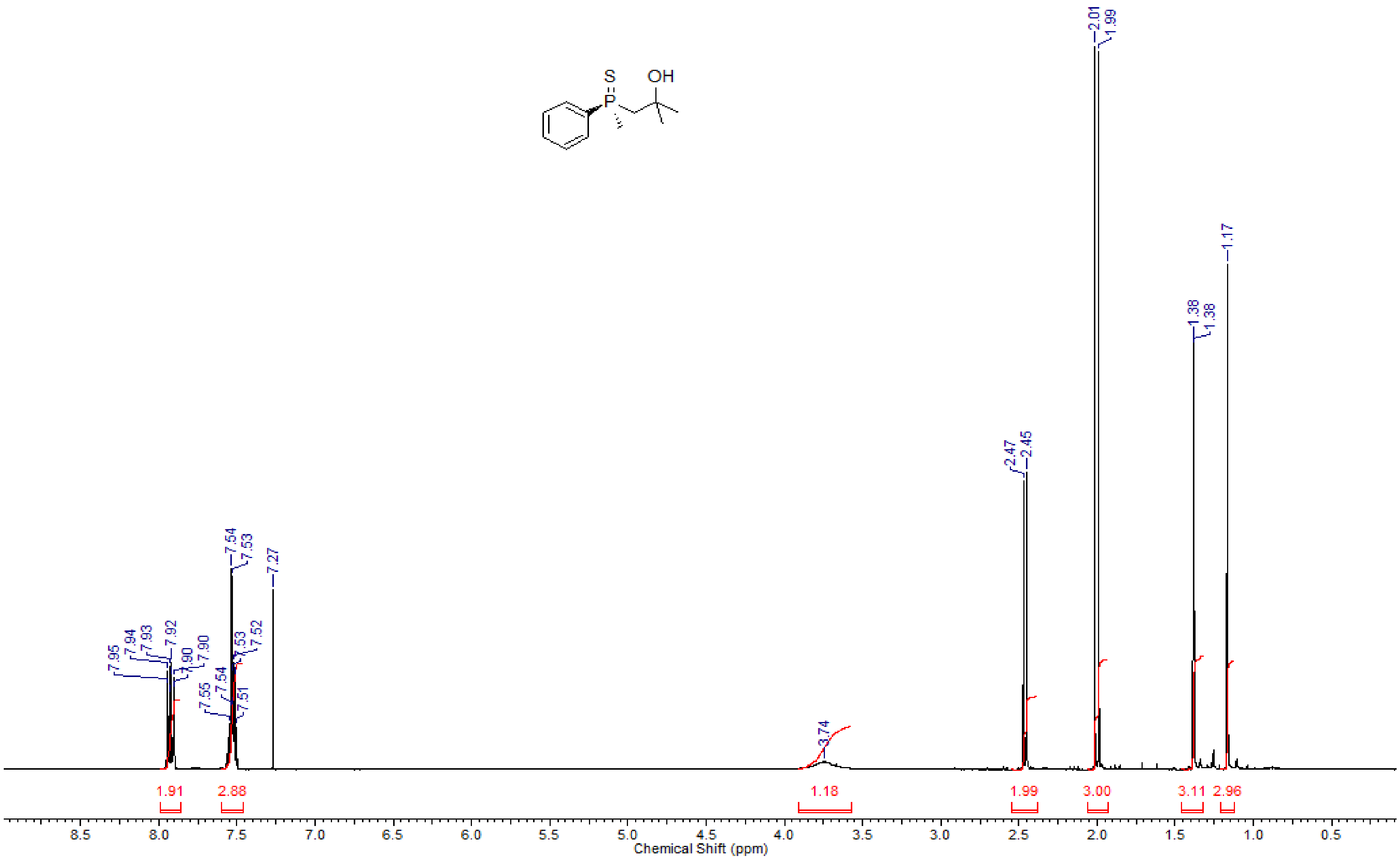

^{1}H NMR spectrum of (S_P)-(2-hydroxy-2-methylpropyl)methylphenylphosphine sulfide (S_P)-(**19**) (CDCl3, 500 MHz).

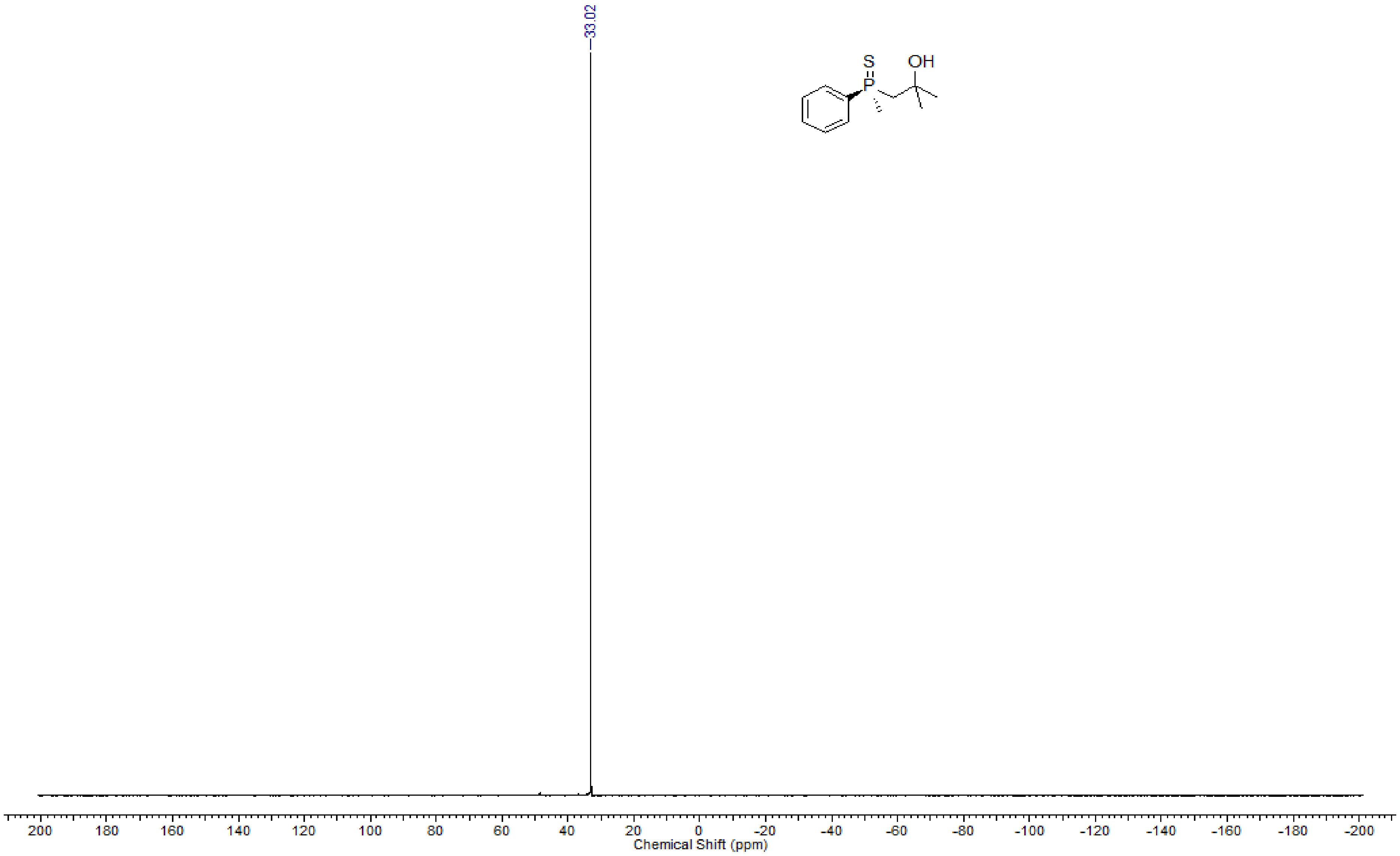

^{31}P NMR spectrum of (S_P)-(2-hydroxy-2-methylpropyl)methylphenylphosphine sulfide (S_P)-(**19**) (CDCl3, 202 MHz).

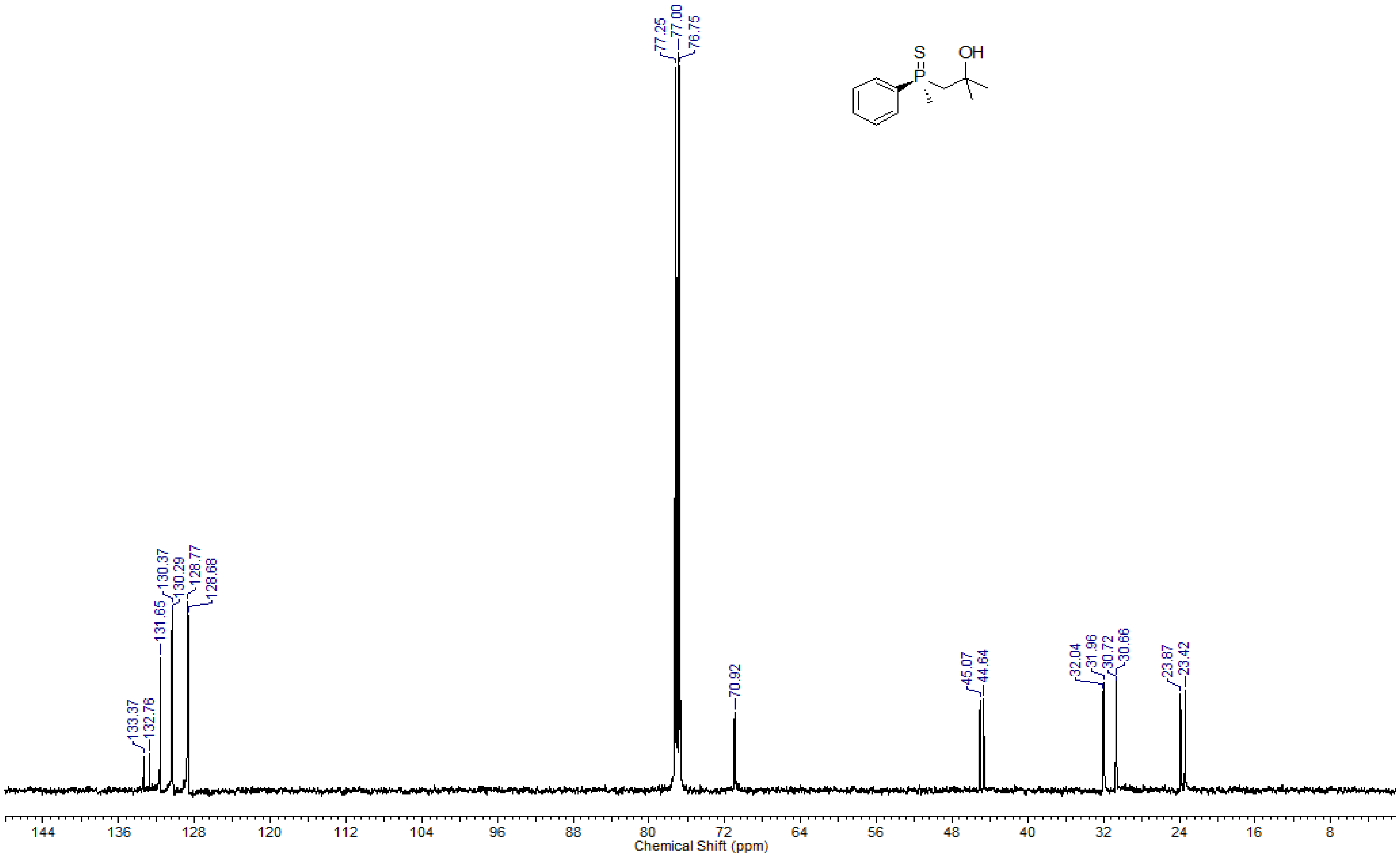

^{13}C NMR spectrum of (S_P)-(2-hydroxy-2-methylpropyl)methylphenylphosphine sulfide (S_P)-(**19**) ($CDCl_3$, 126 MHz).

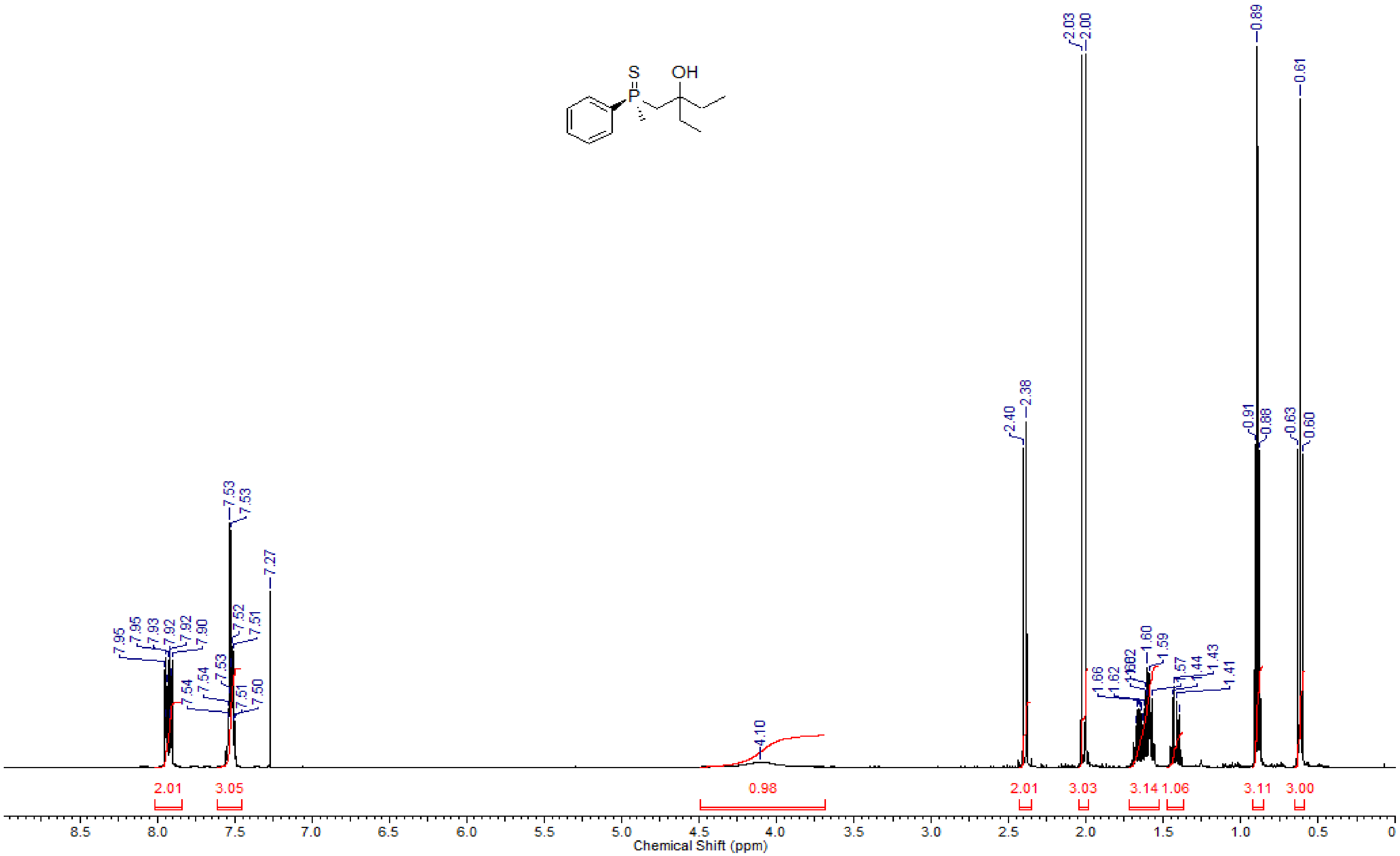

^{1}H NMR spectrum of (S_P)-(2-ethyl-2-hydroxybutyl)methylphenylphosphine sulfide (S_P)-(**20**) (CDCl3, 500 MHz).

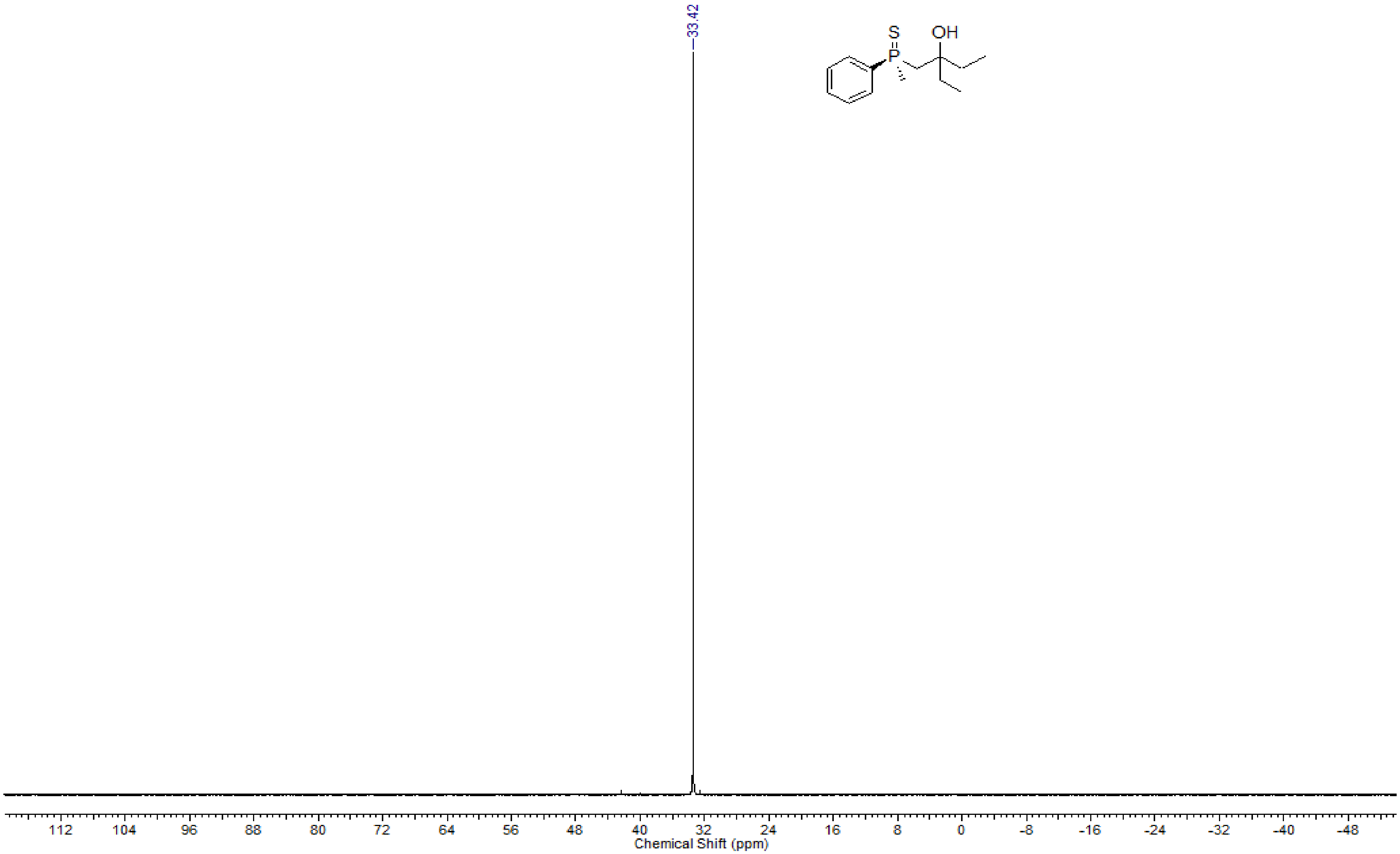

^{31}P NMR spectrum of (*S*P)-(2-ethyl-2-hydroxybutyl)methylphenylphosphine sulfide (*S*P)-(**20**) (CDCl3, 202 MHz).

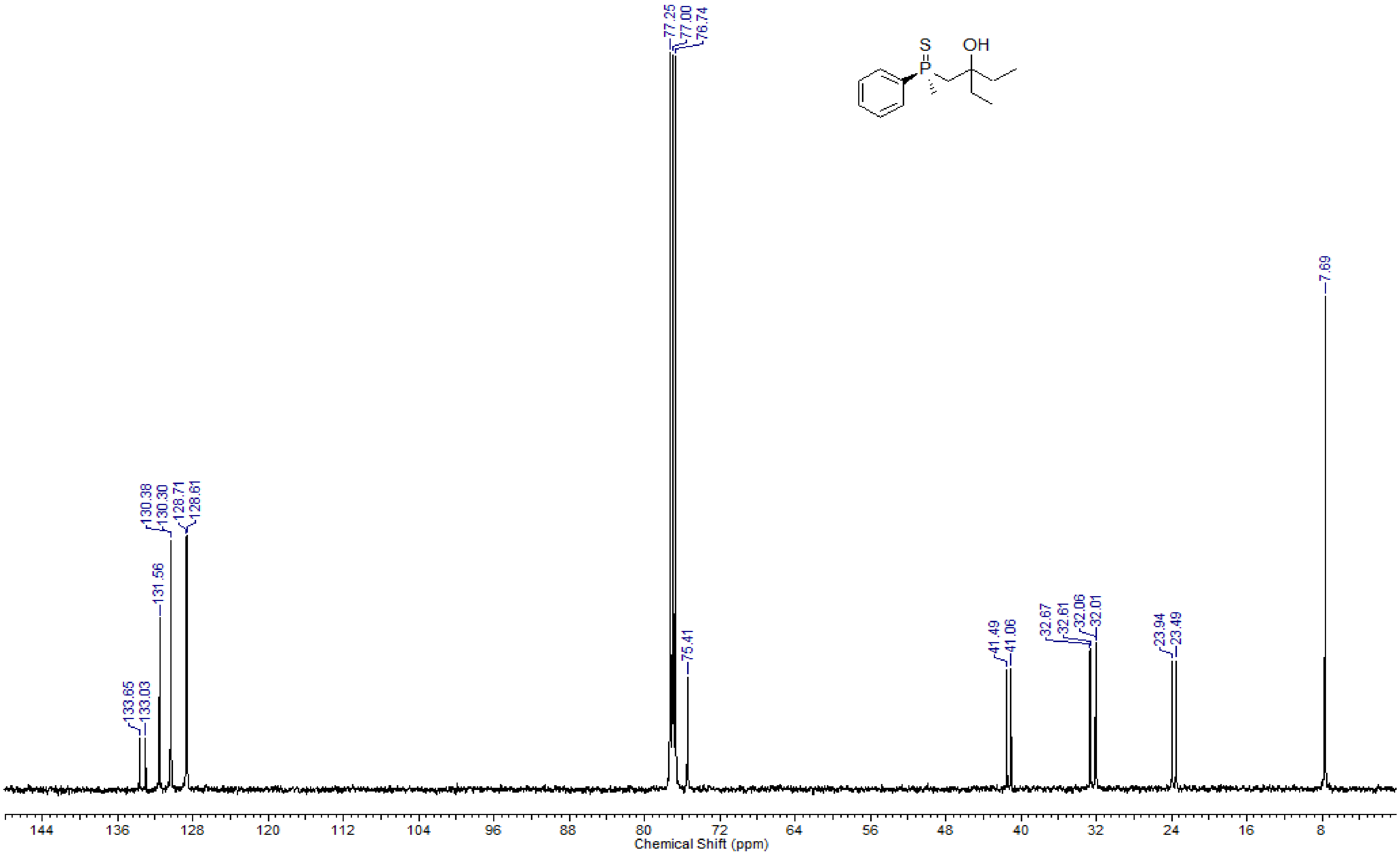

^{13}C NMR spectrum of (S_P)-(2-ethyl-2-hydroxybutyl)methylphenylphosphine sulfide (S_P)-(**20**) (CDCl3, 126 MHz).

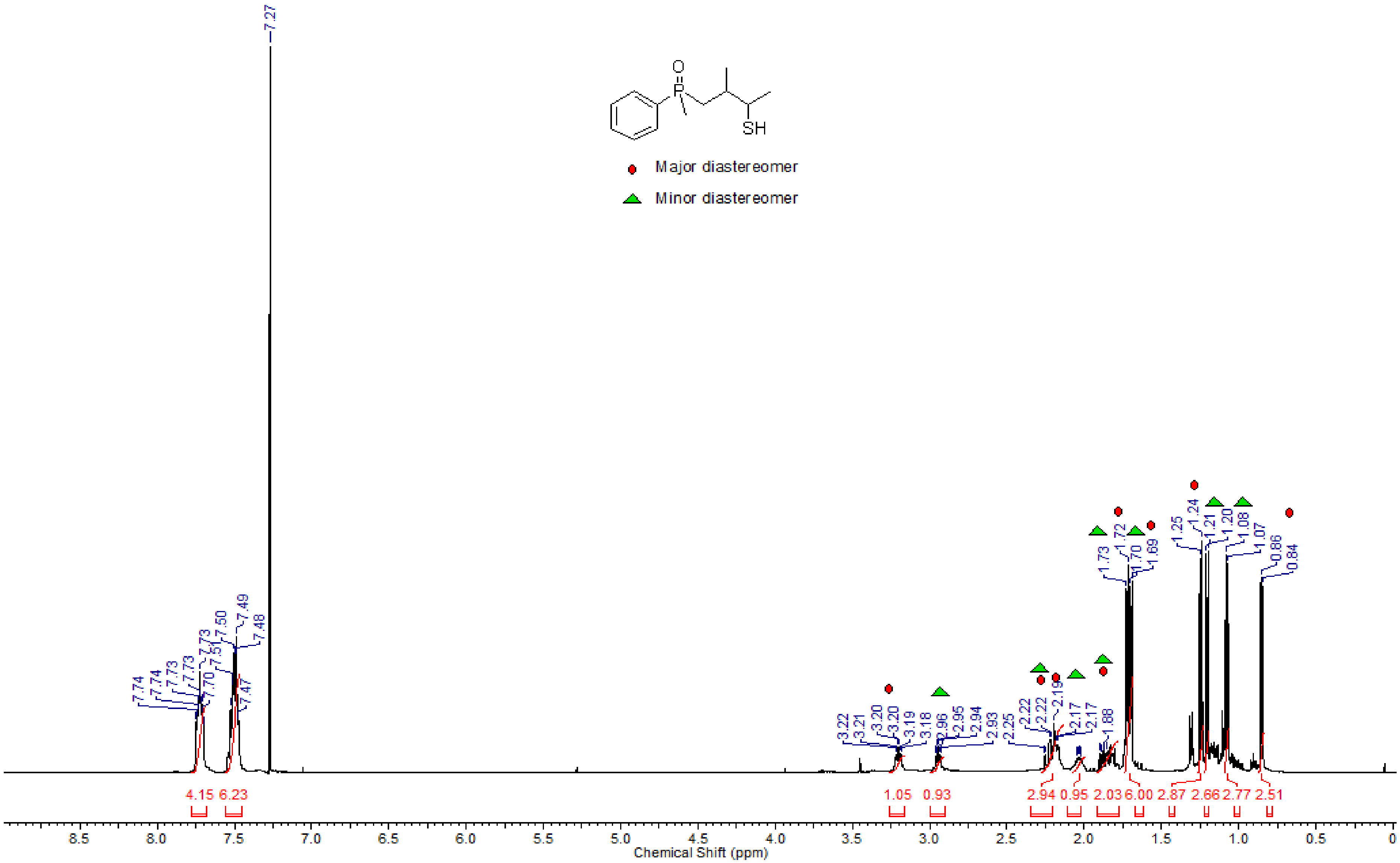

^{1}H NMR spectrum of (2-methyl-3-mercaptobutyl)methylphenylphosphine oxide (**29**) (CDCl3, 500 MHz).

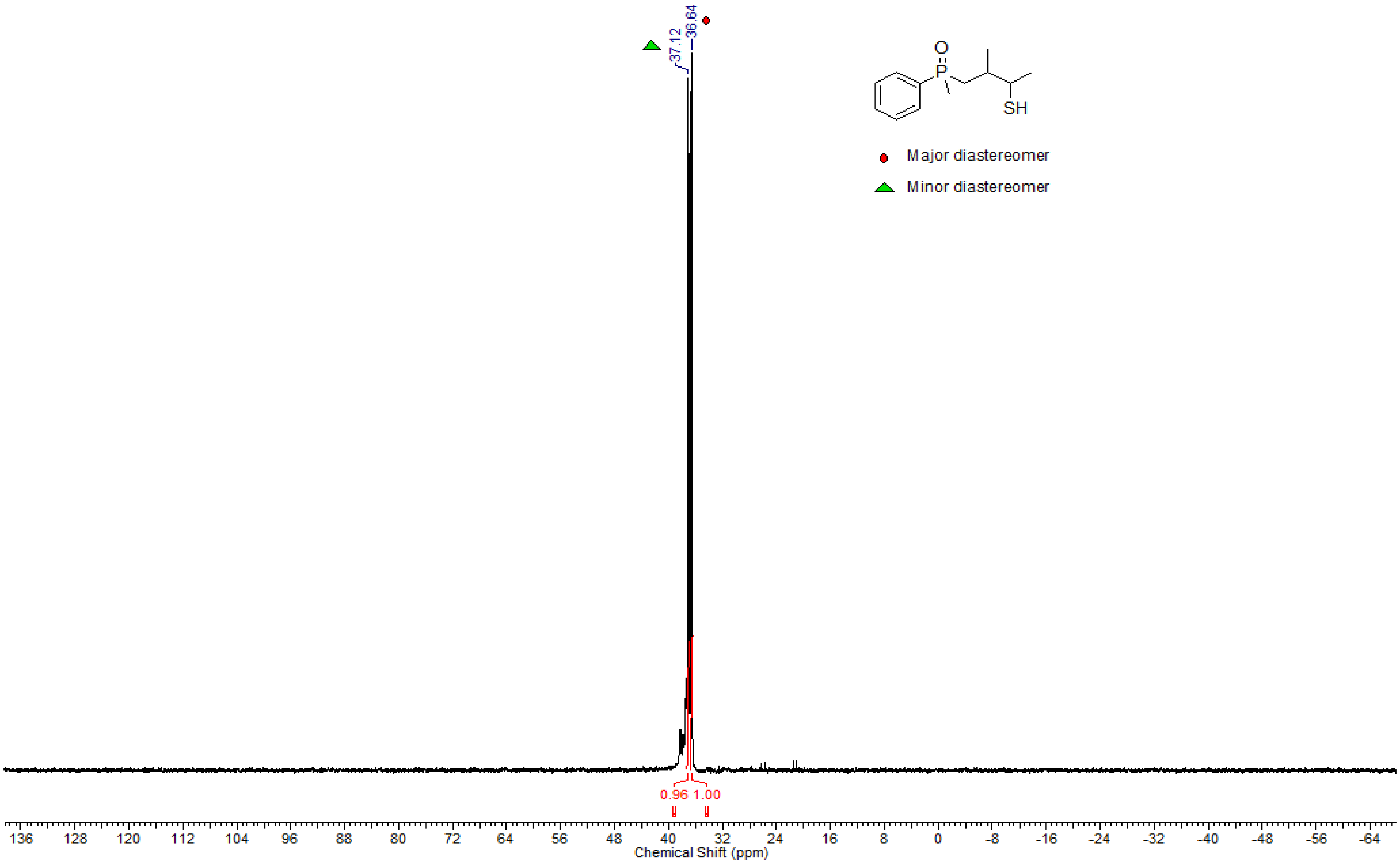

^{31}P NMR spectrum of (2-methyl-3-mercaptobutyl)methylphenylphosphine oxide (**29**) (CDCl3, 202 MHz).

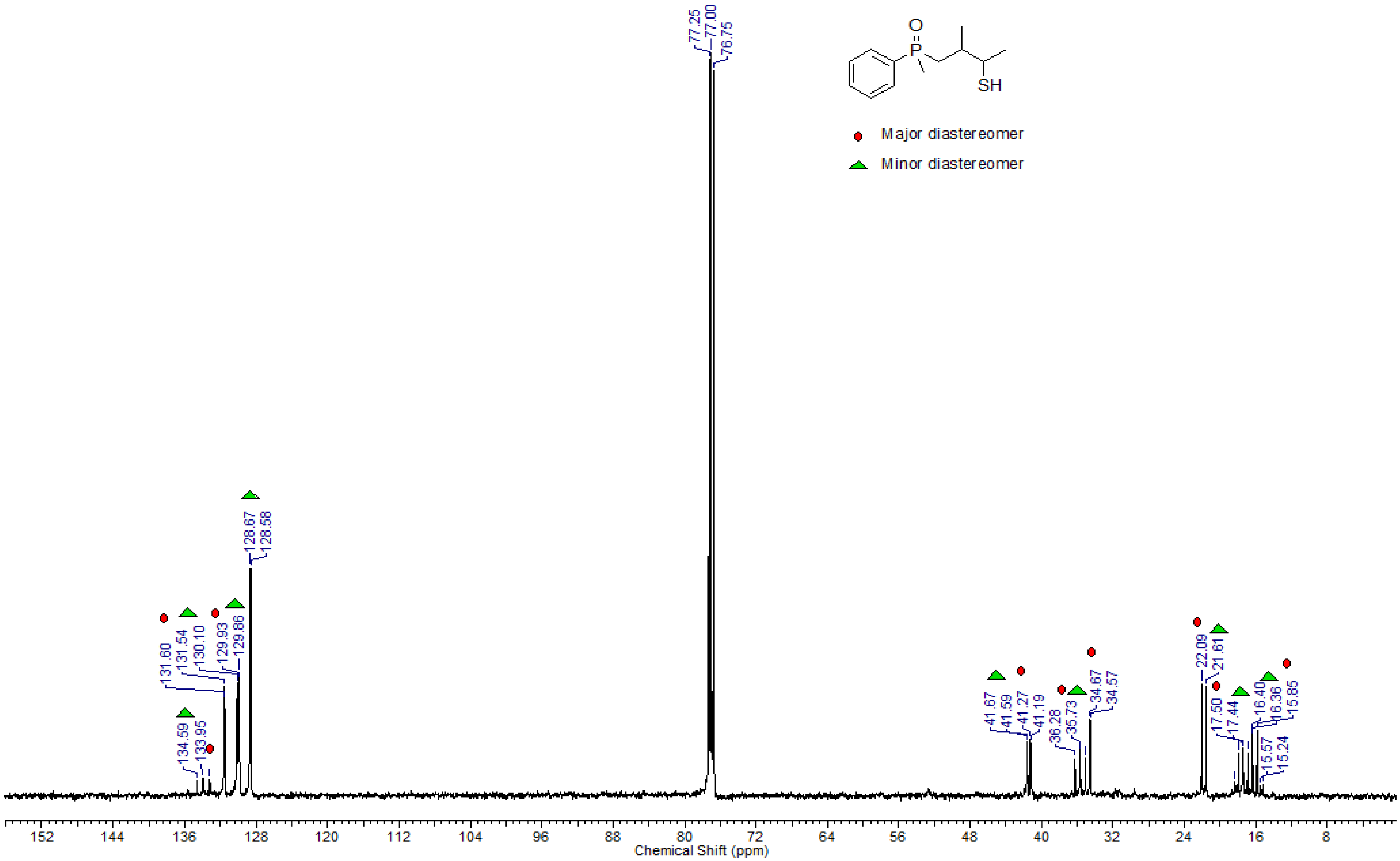

^{13}C NMR spectrum of (2-methyl-3-mercaptobutyl)methylphenylphosphine oxide (**29**) (CDCl3, 126 MHz).

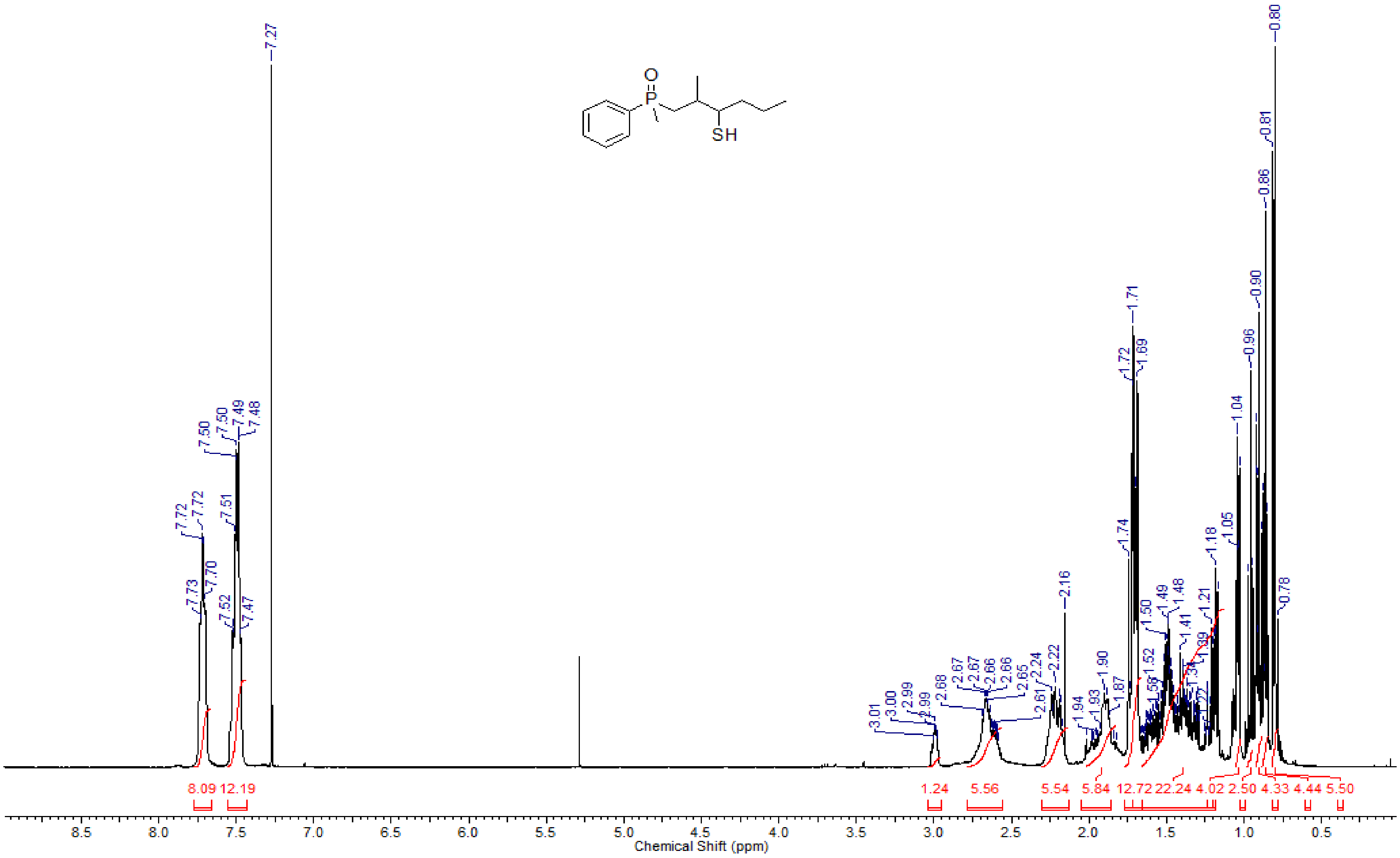

^{1}H NMR spectrum of (2-methyl-3-mercaptohexyl)methylphenylphosphine oxide (**30**) (CDCl3, 500 MHz).

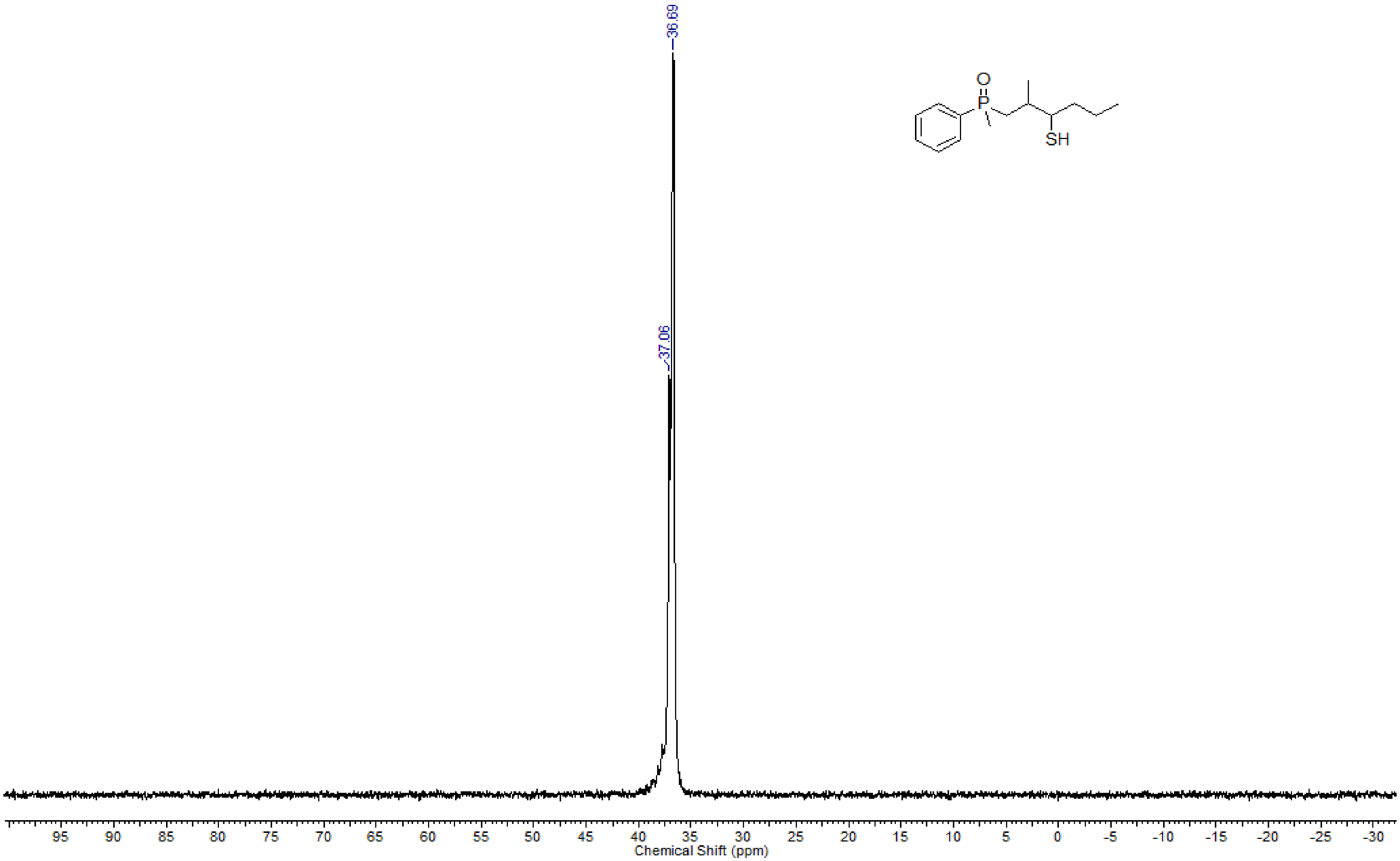

^{31}P NMR spectrum of (2-methyl-3-mercaptohexyl)methylphenylphosphine oxide (**30**) (CDCl3, 202 MHz).

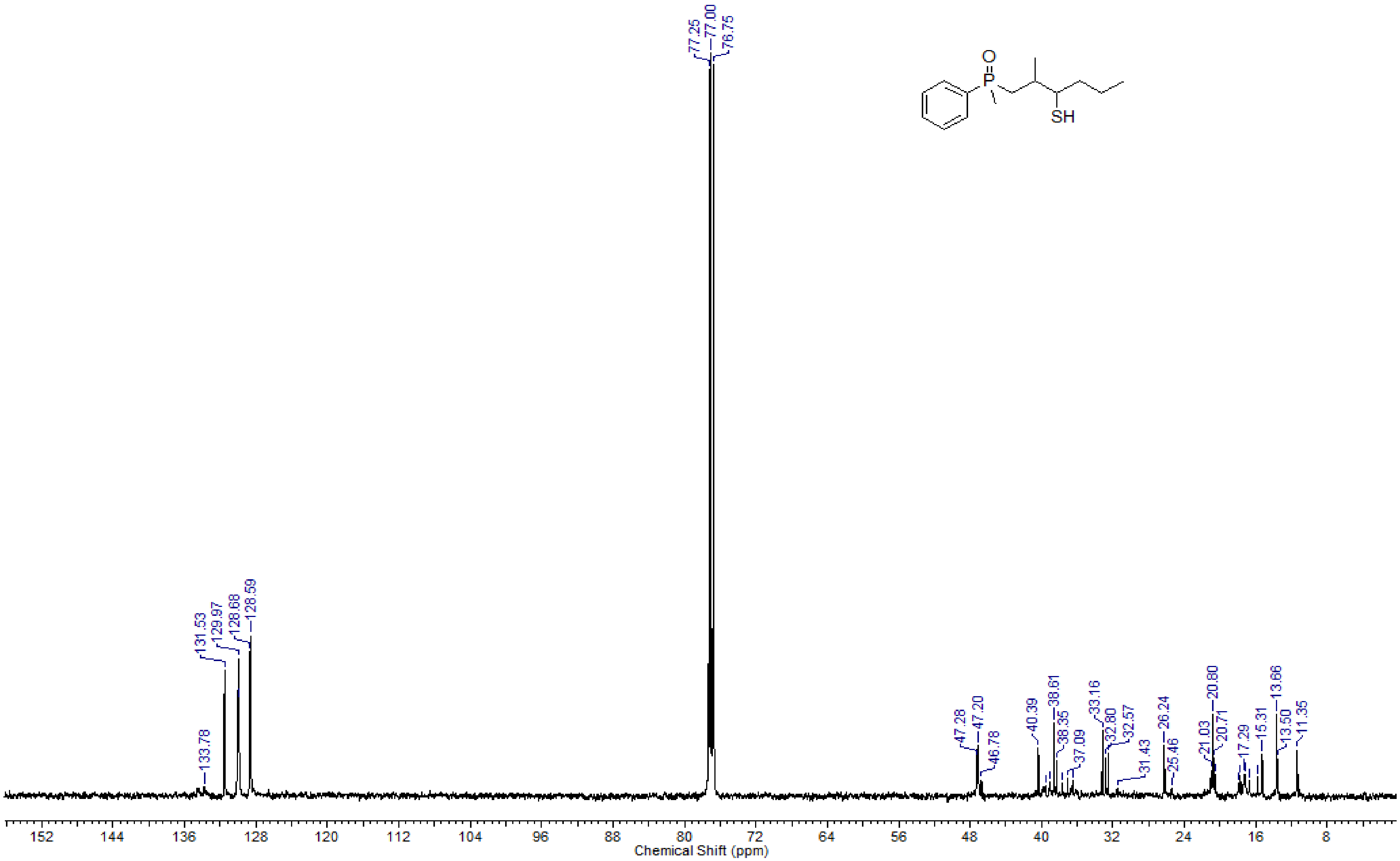

^{13}C NMR spectrum of (2-methyl-3-vhexyl)methylphenylphosphine oxide (**30**) (CDCl3, 126 MHz).

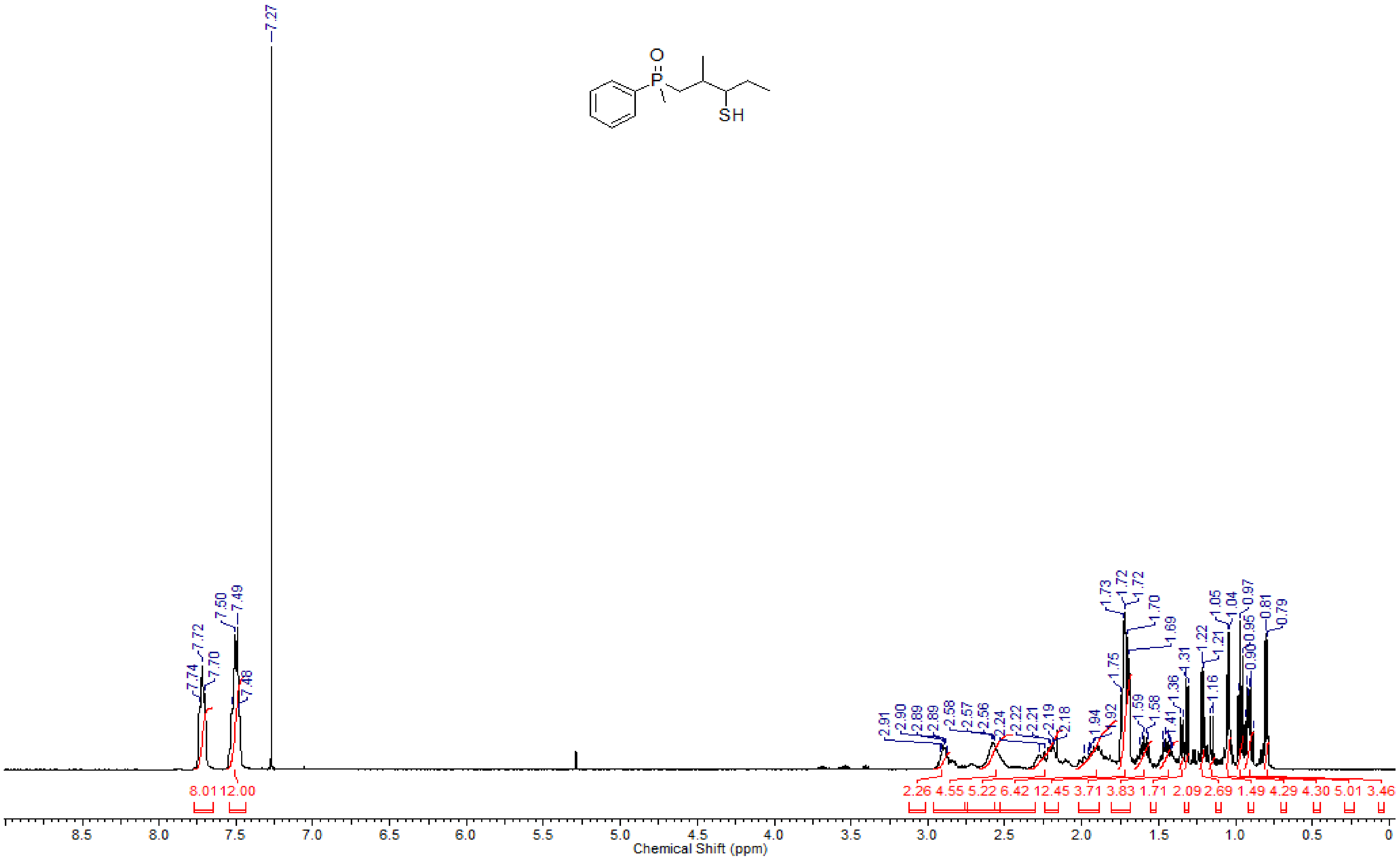

^{1}H NMR spectrum of (2-methyl-3-mercaptopentyl)methylphenylphosphine oxide (**31**) (CDCl3, 500 MHz).

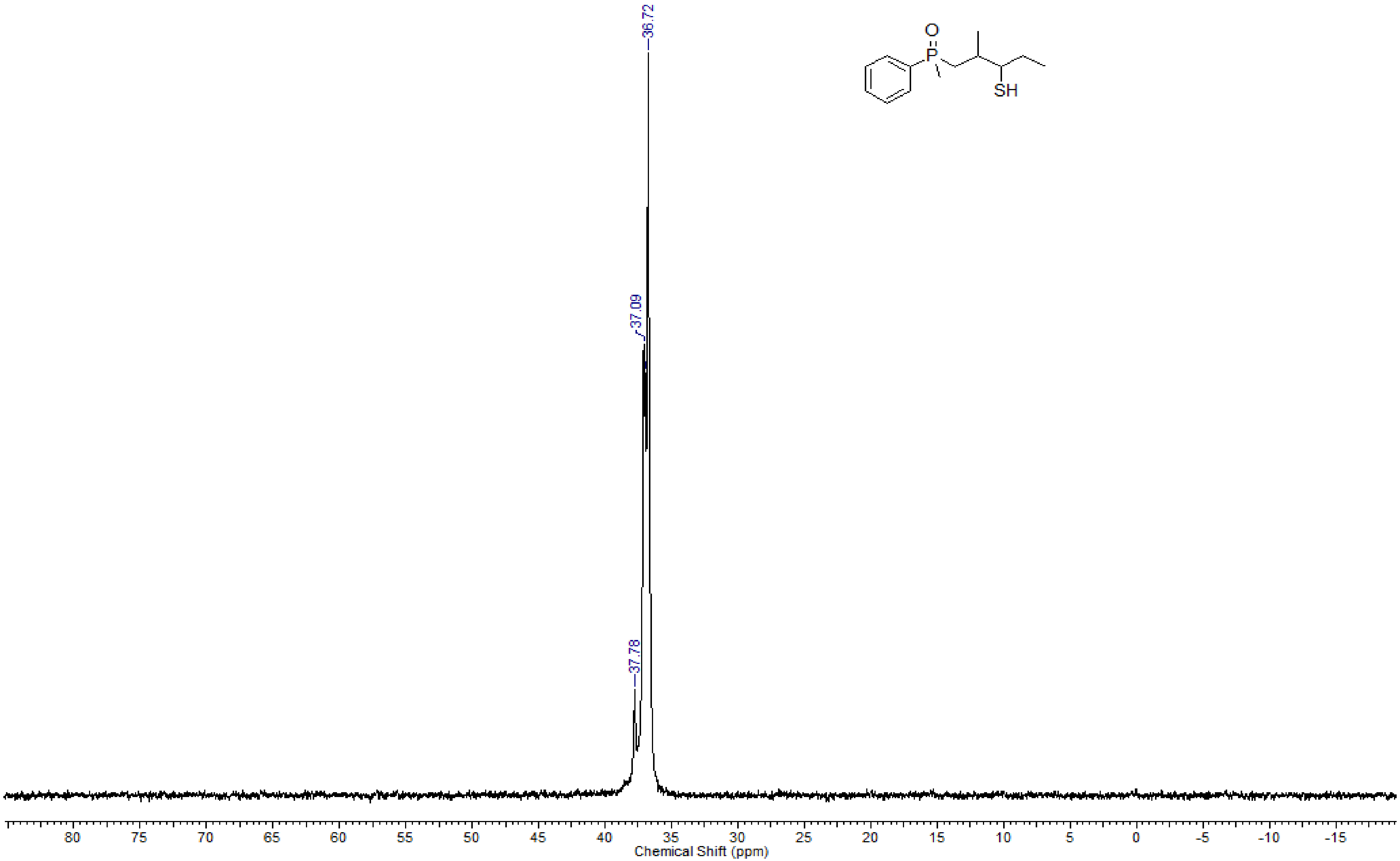

^{31}P NMR spectrum of (2-methyl-3-mercaptopentyl)methylphenylphosphine oxide (**31**) (CDCl3, 202 MHz).

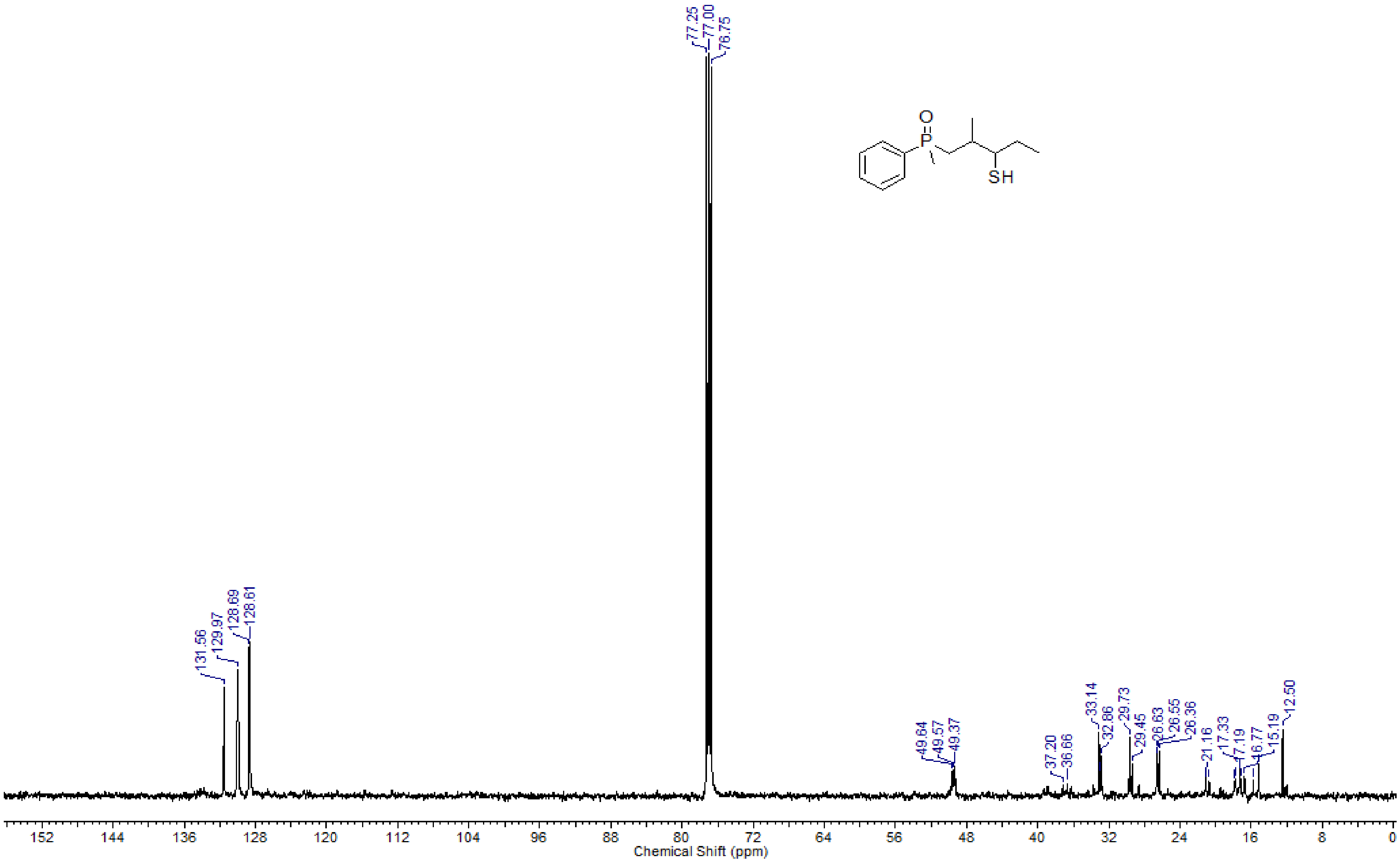

^{13}C NMR spectrum of (2-methyl-3-mercaptopentyl)methylphenylphosphine oxide (**31**) (CDCl3, 126 MHz).

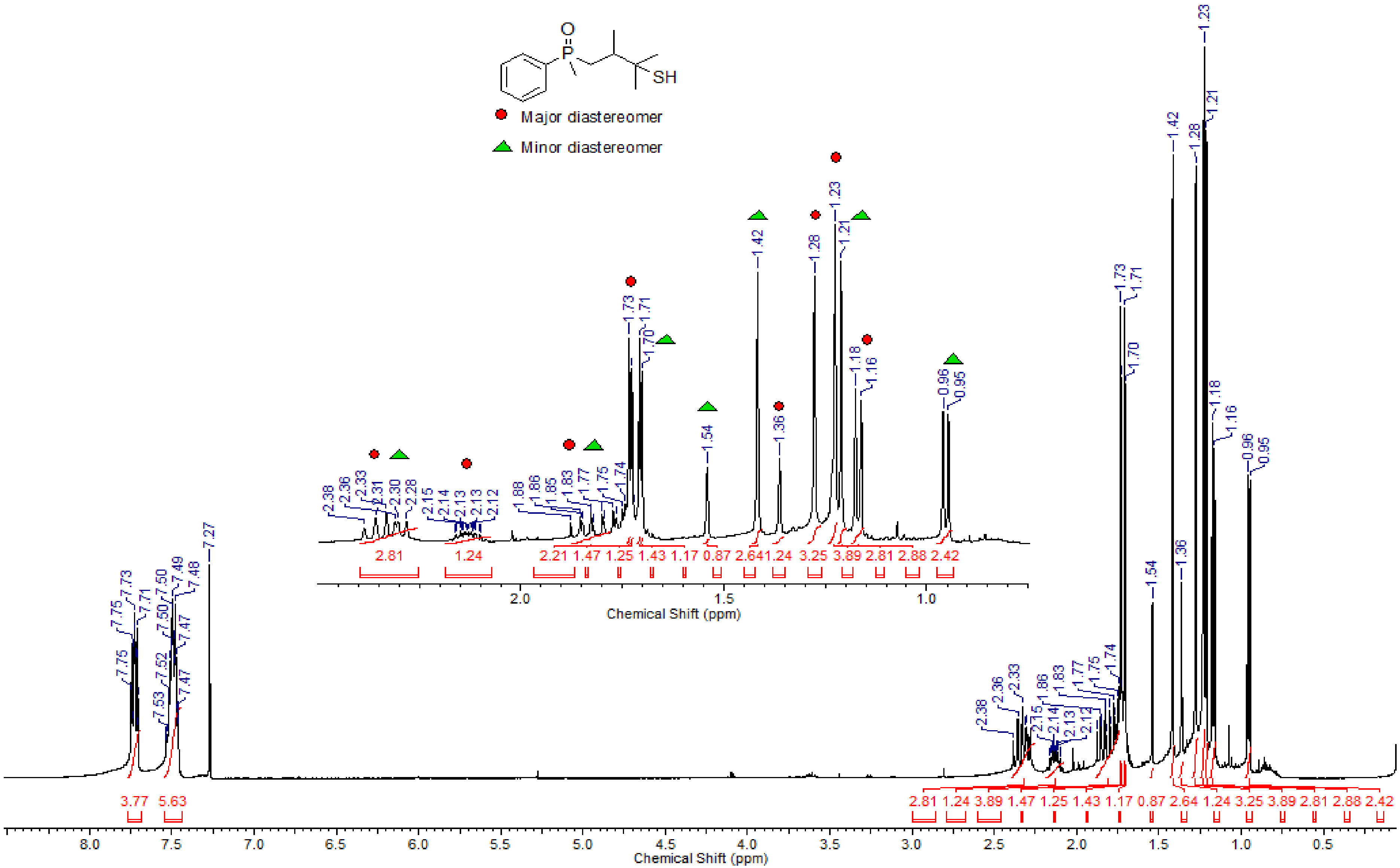

^{1}H NMR spectrum of (2,3-dimethyl-3-mercaptobutyl)methylphenylphosphine oxide (**32**) (CDCl3, 500 MHz).

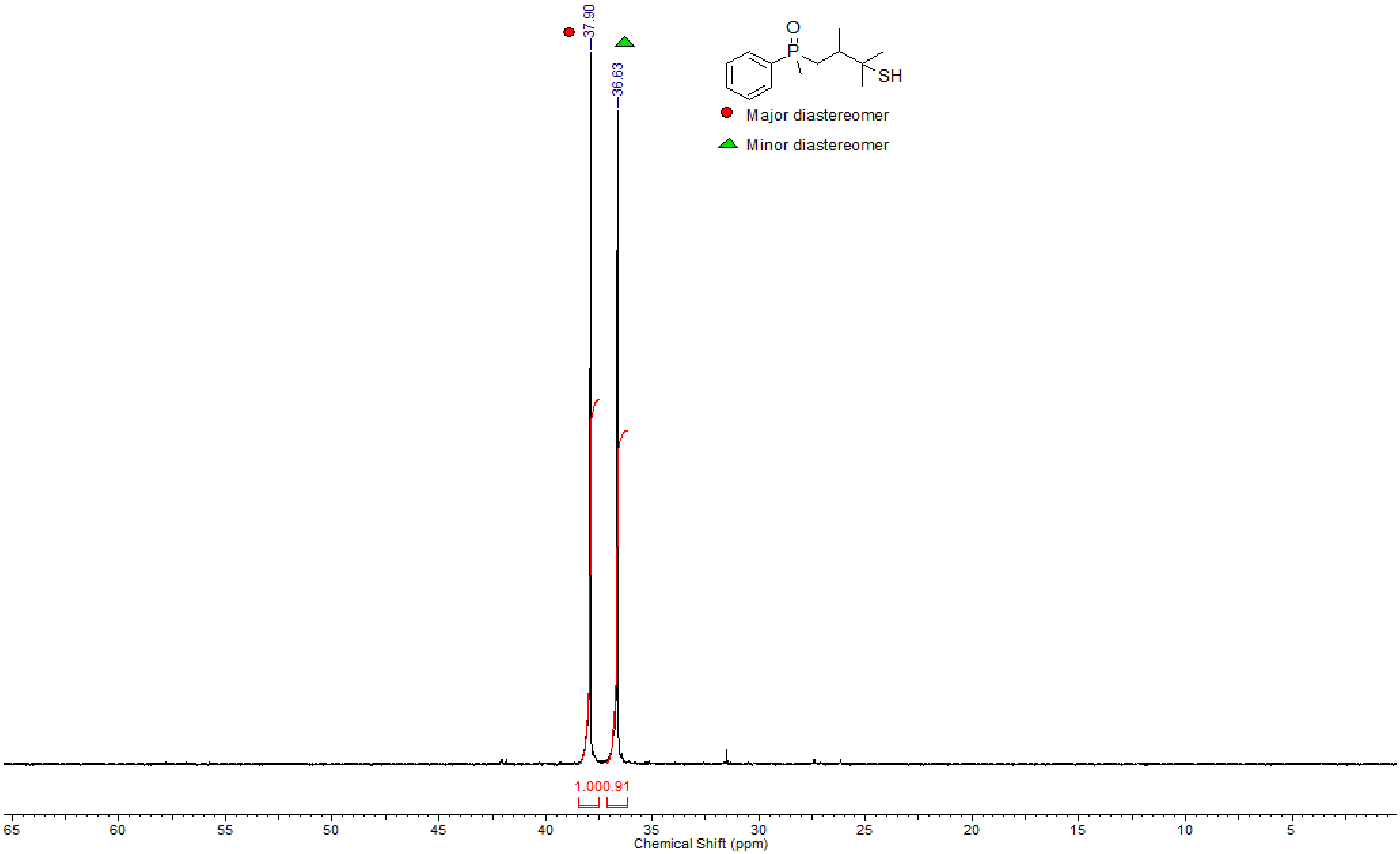

^{31}P NMR spectrum of (2,3-dimethyl-3-mercaptobutyl)methylphenylphosphine oxide (**32**) (CDCl3, 202 MHz).

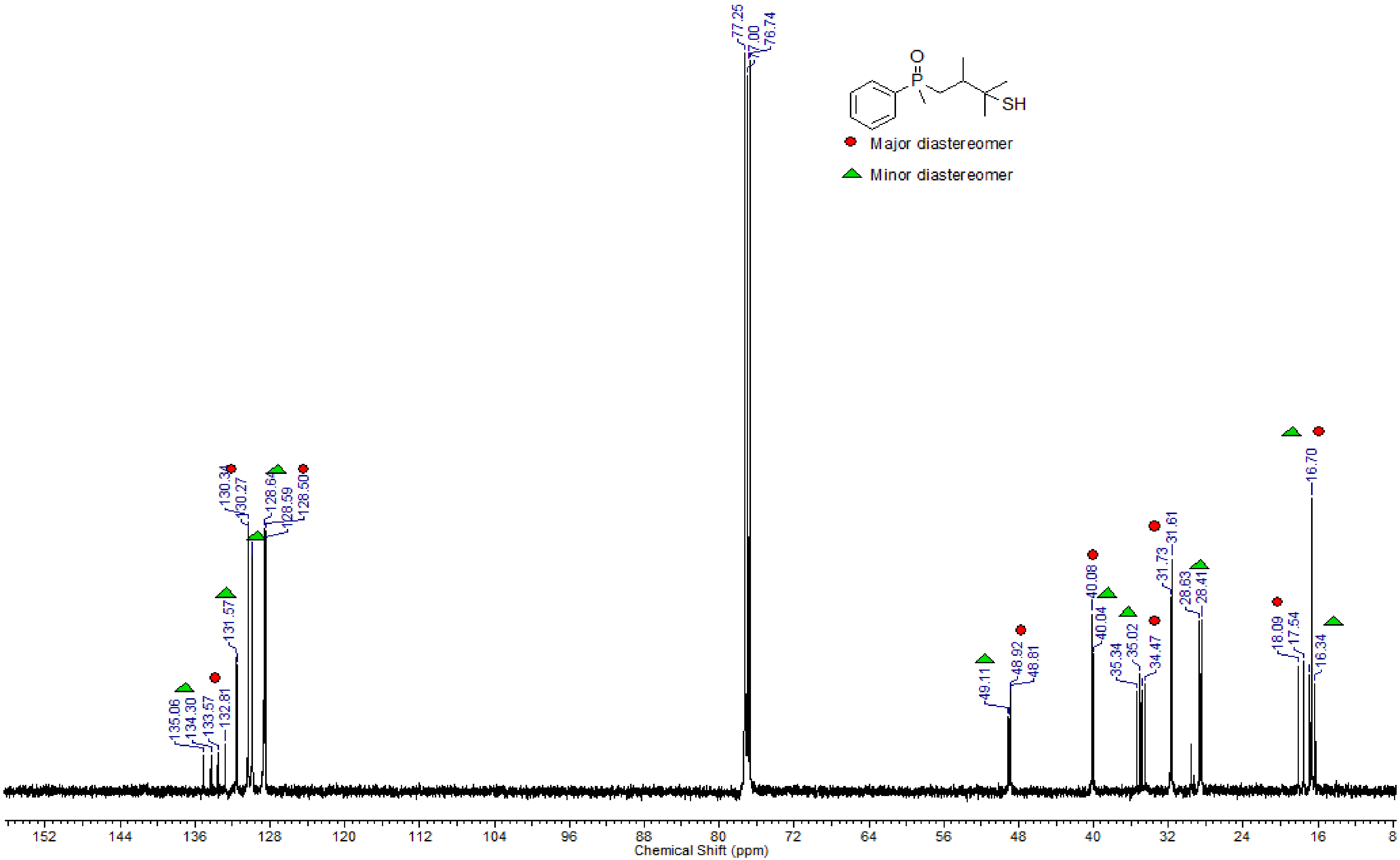

^{13}C NMR spectrum of (2,3-dimethyl-3-mercaptobutyl)methylphenylphosphine oxide (**32**) (CDCl3, 126 MHz).

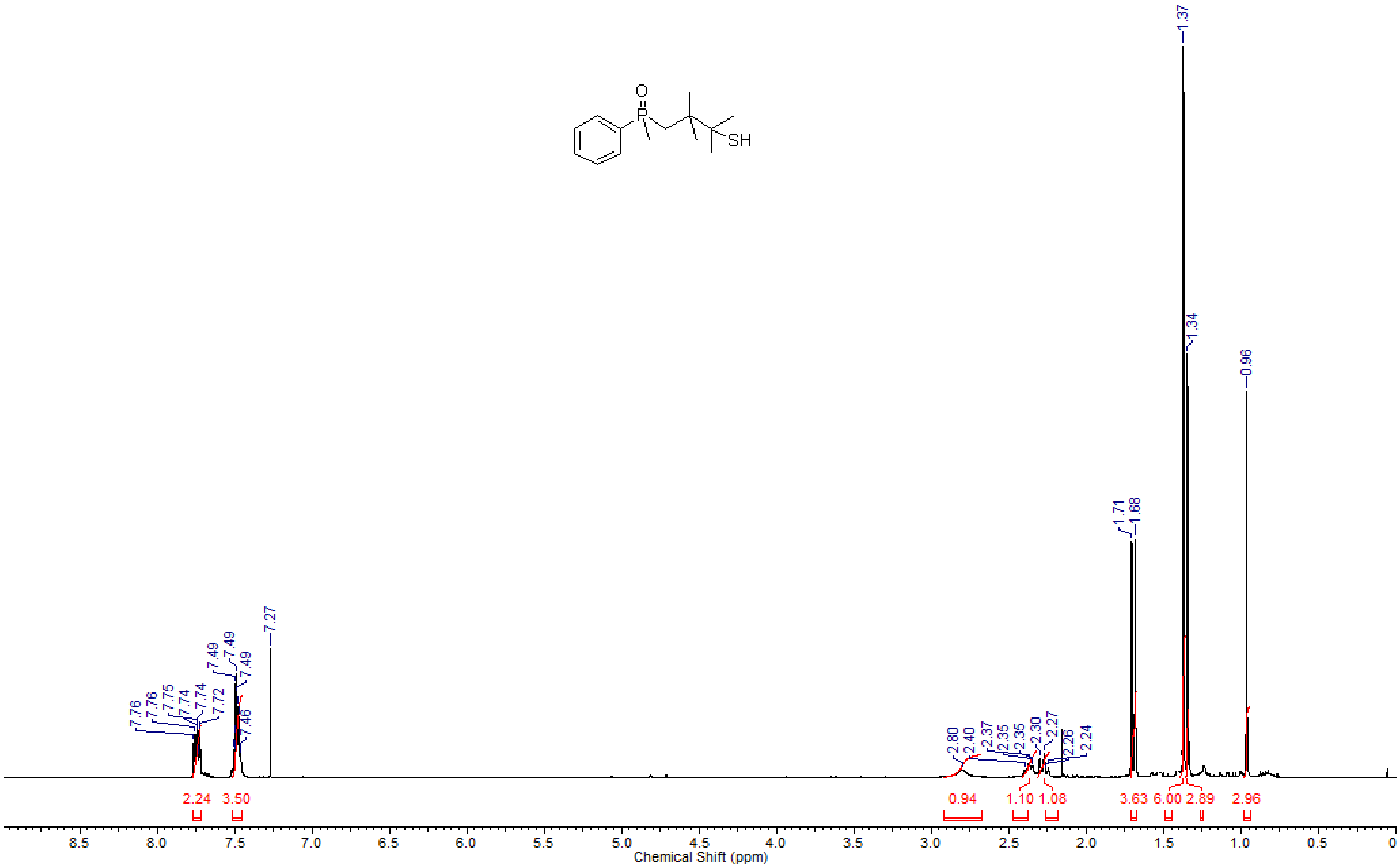

^{1}H NMR spectrum of (2,2-dimethyl-3-mercaptobutyl)methylphenylphosphine oxide (**33**) (CDCl3, 500 MHz).

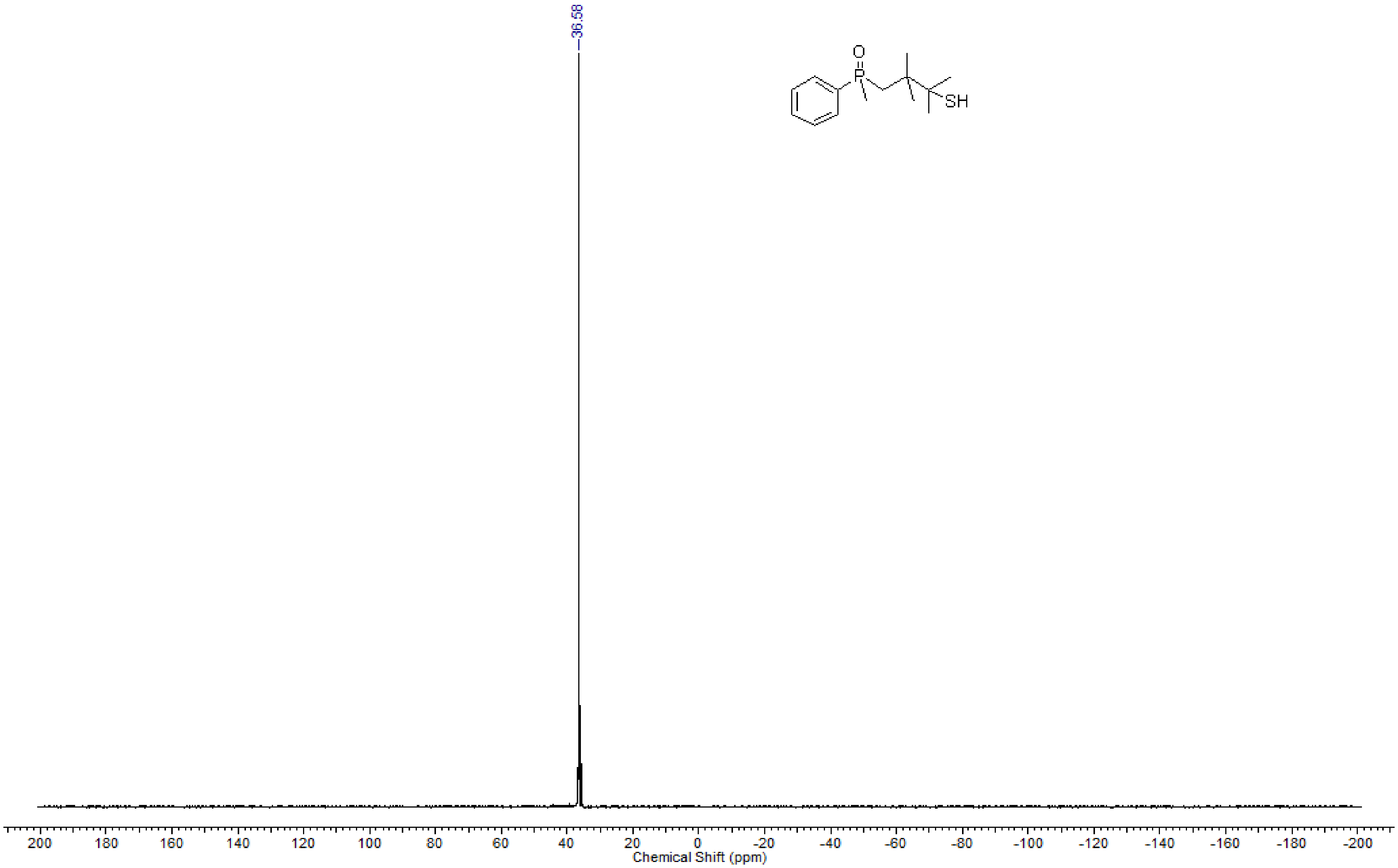

^{31}P NMR spectrum of (2,2-dimethyl-3-mercaptobutyl)methylphenylphosphine oxide (**33**) (CDCl3, 202 MHz).

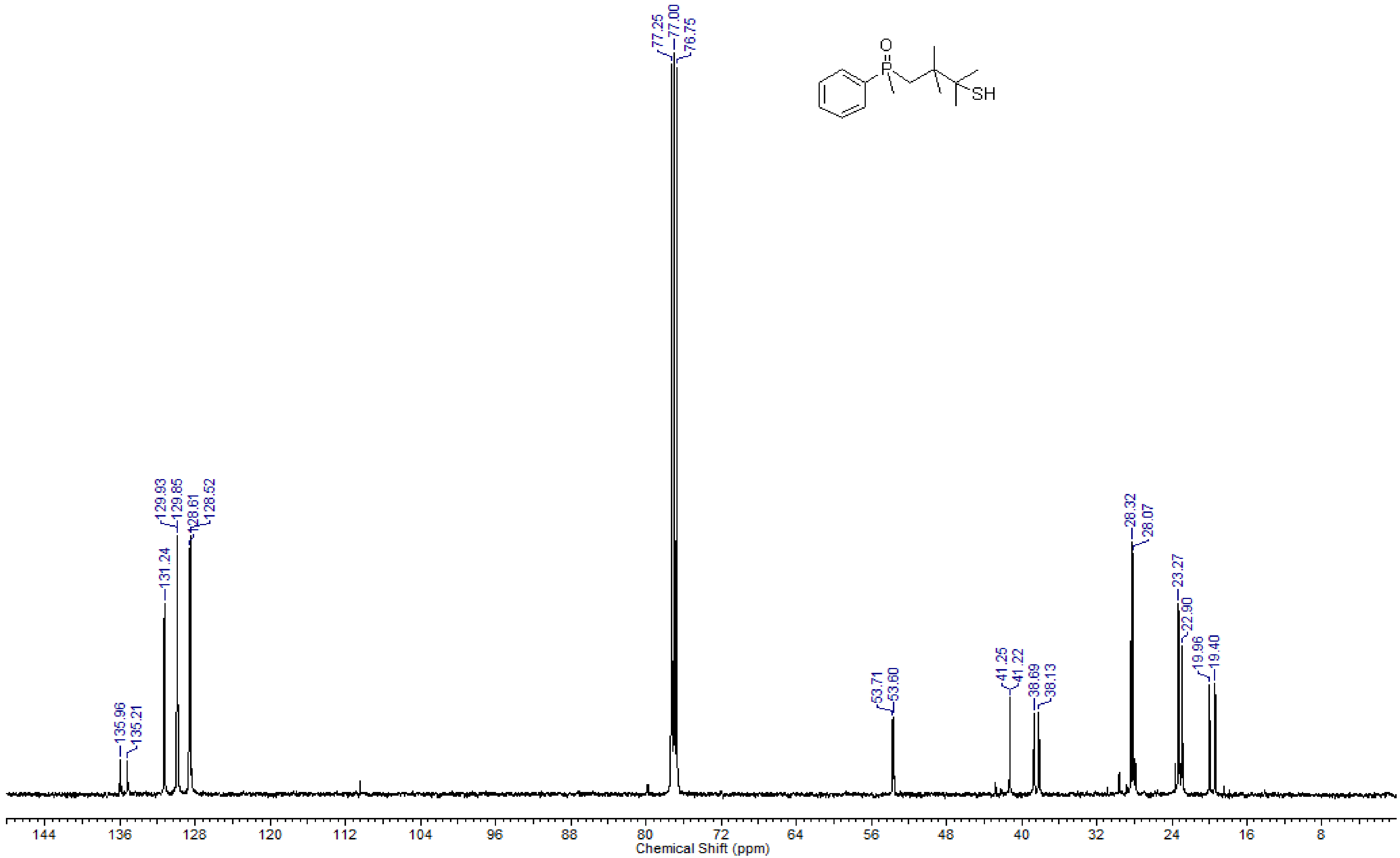

^{13}C NMR spectrum of (2,2-dimethyl-3-mercaptobutyl)methylphenylphosphine oxide (**33**) (CDCl3, 126 MHz).

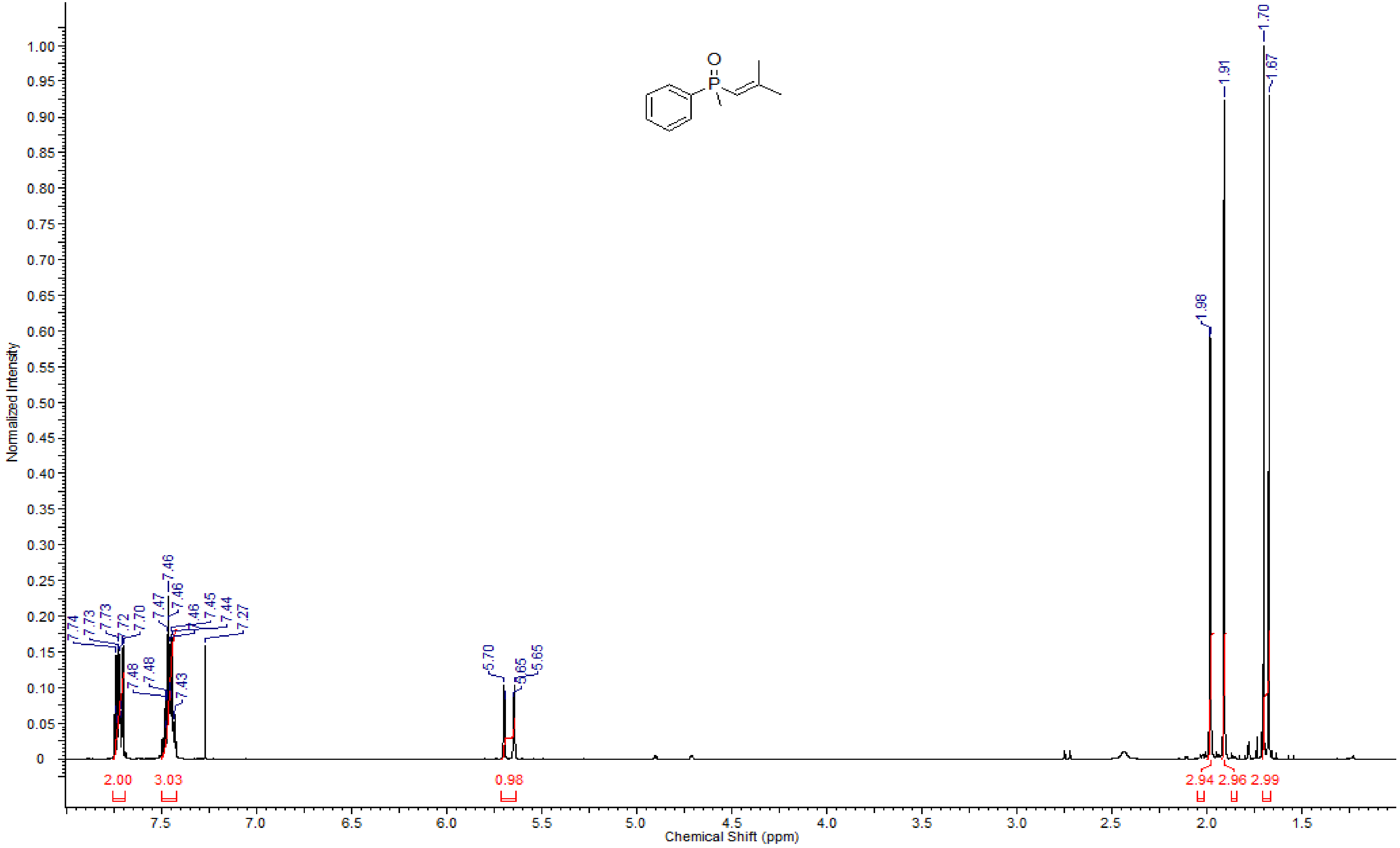

^{1}H NMR spectrum of (2-methylprop-1-enyl)(methylphenyl)phosphine oxide (**34**) (CDCl3, 500 MHz).

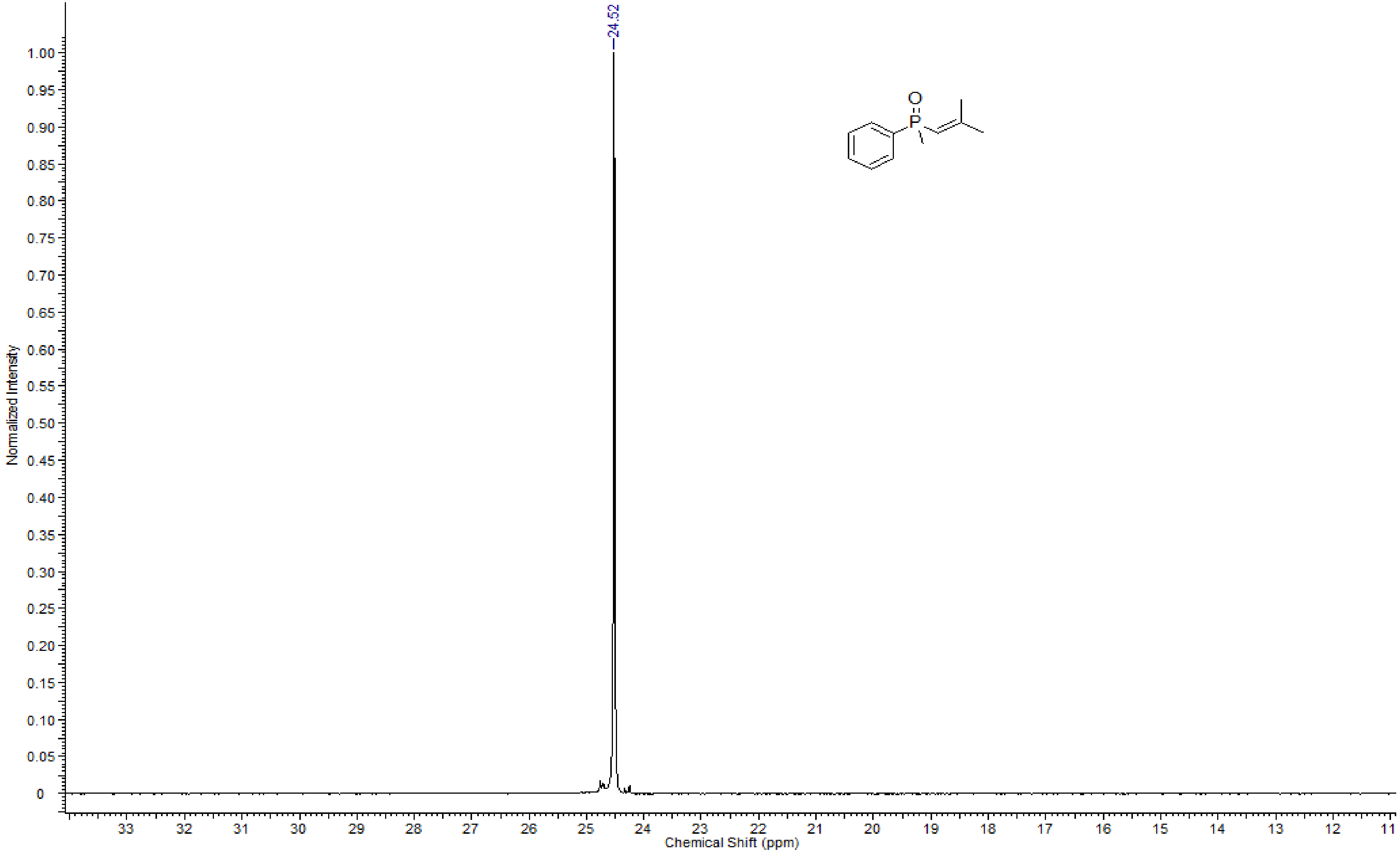

^{31}P NMR spectrum of (2-methylprop-1-enyl)(methylphenyl)phosphine oxide (**34**) (CDCl3, 202 MHz).

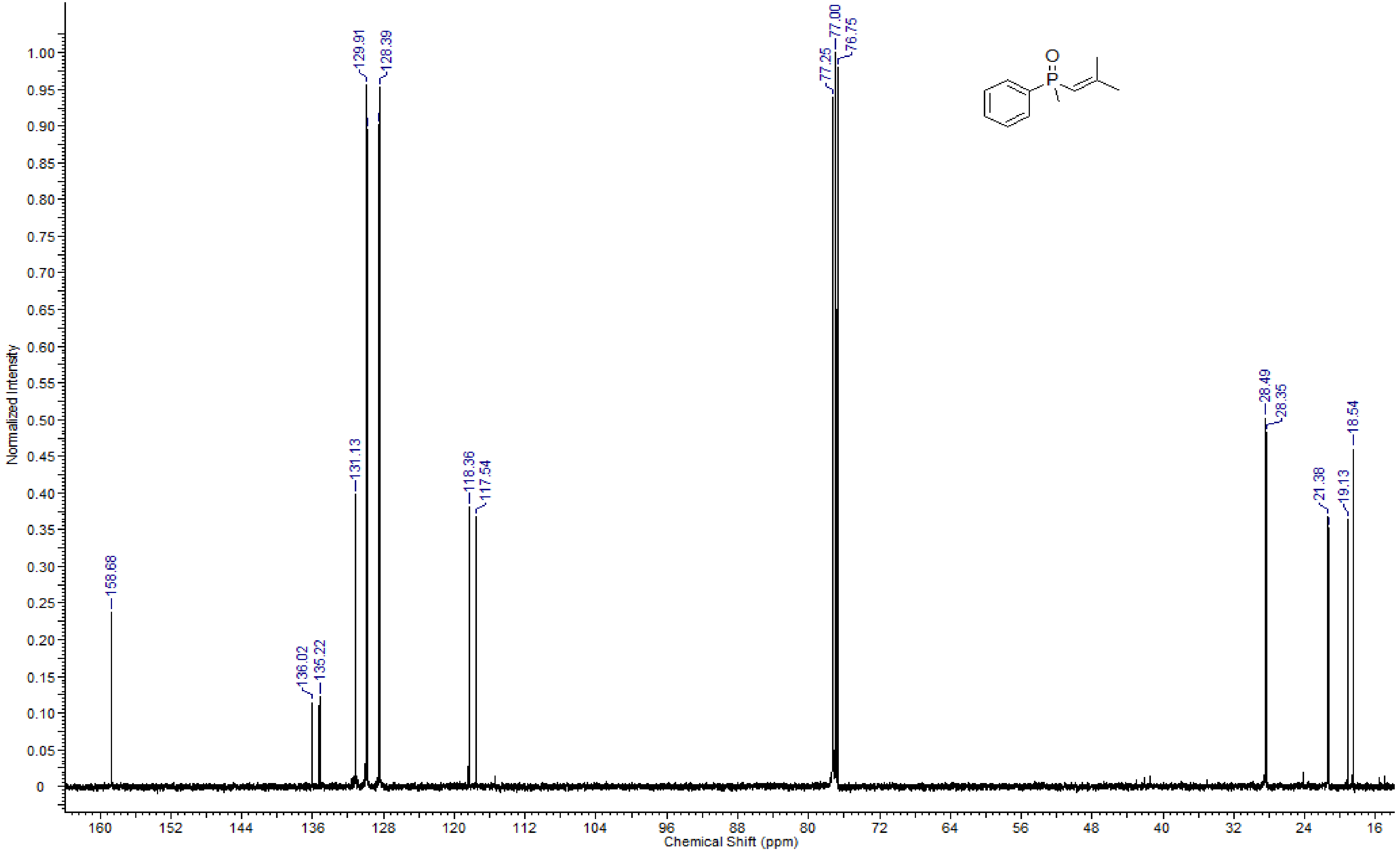

^{13}C NMR spectrum of (2-methylprop-1-enyl)(methylphenyl)phosphine oxide (**34**) (CDCl3, 126 MHz).

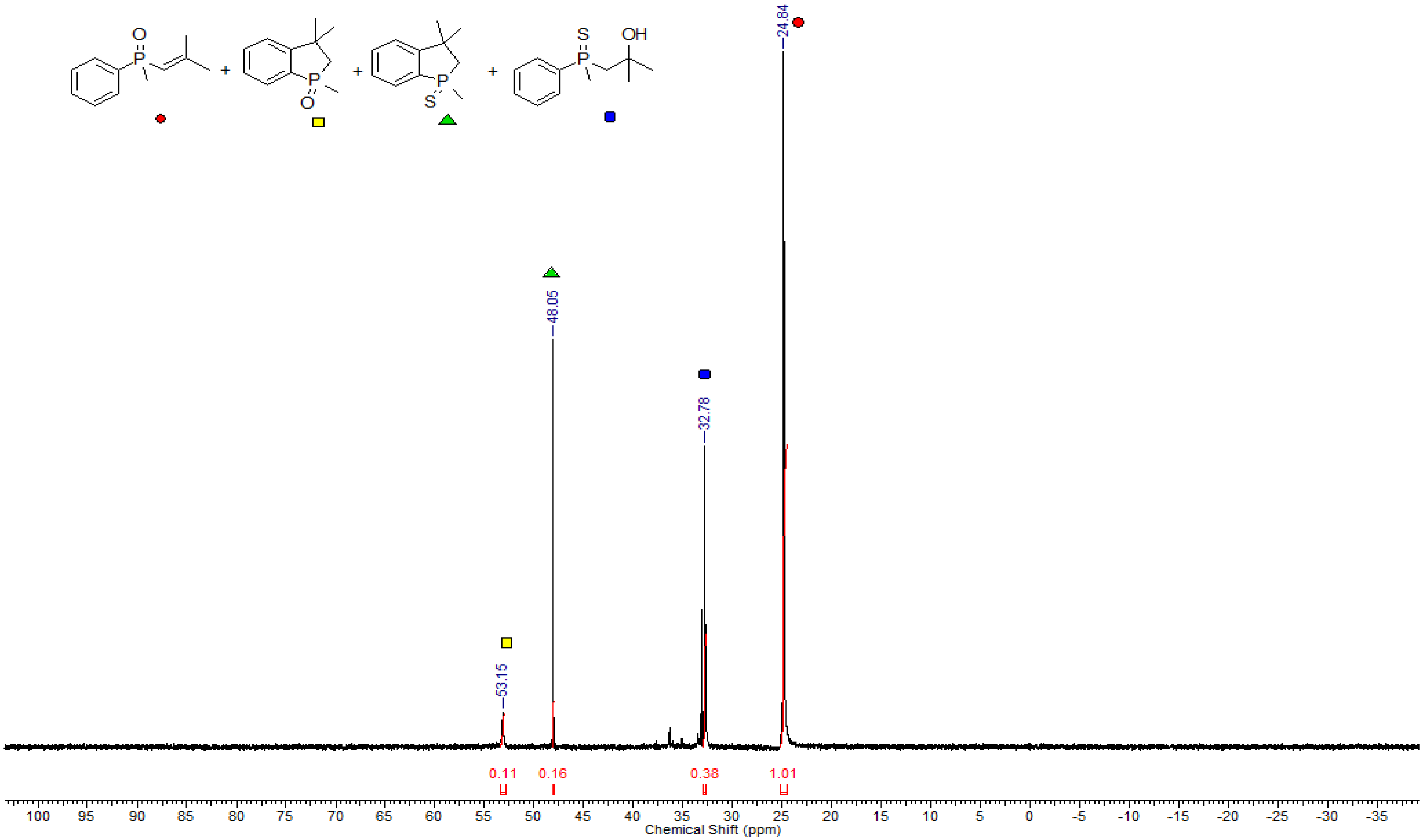

[1]P NMR spectrum of 1,3,3-trimethylphosphindoline 1-sulfide (**35**) with (2-methylprop-1-enyl)(methylphenyl)phosphine oxide (**34**) and 1,3,3-trimethylphosphindoline 1-oxide (**3**) and (2-hydroxy-2-methylpropyl)methylphenylphosphine sulfide (**19**) (CDCl3, 202 MHz).

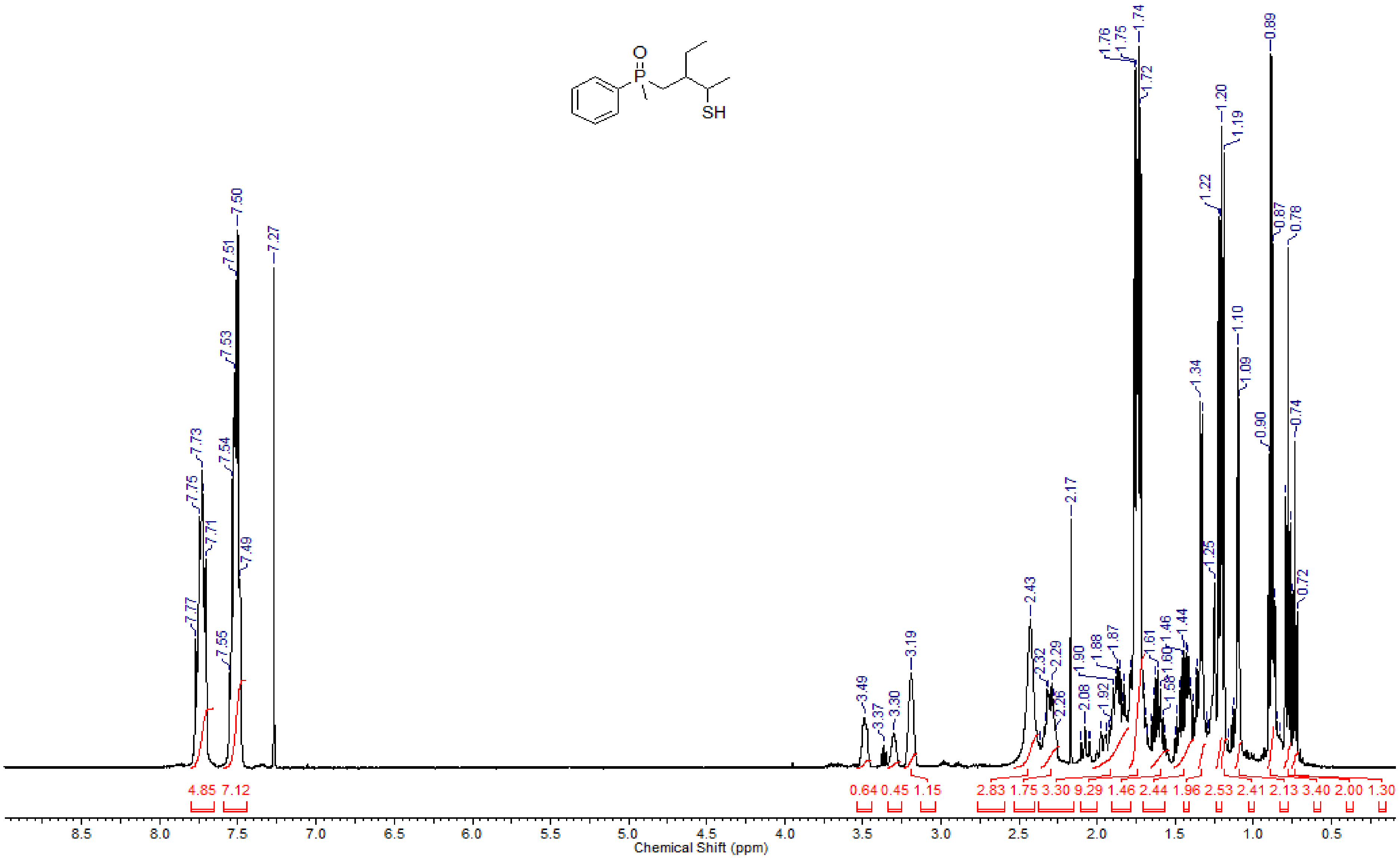

^{1}H NMR spectrum of (2-ethyl-3-mercaptobutyl)methylphenylphosphine oxide (**36**) (CDCl3, 500 MHz).

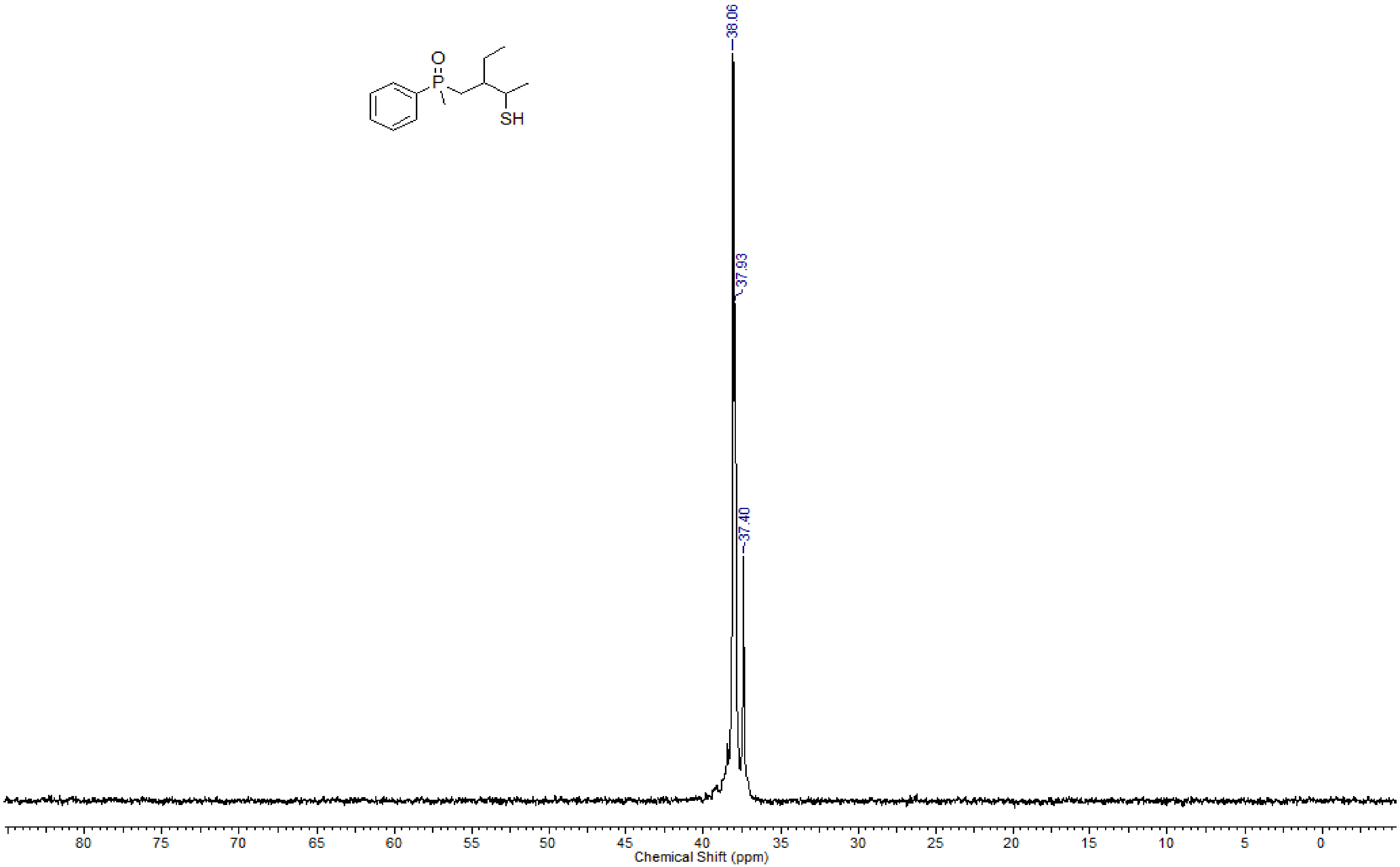

^{31}P NMR spectrum of (2-ethyl-3-mercaptobutyl)methylphenylphosphine oxide (**36**) (CDCl3, 202 MHz).

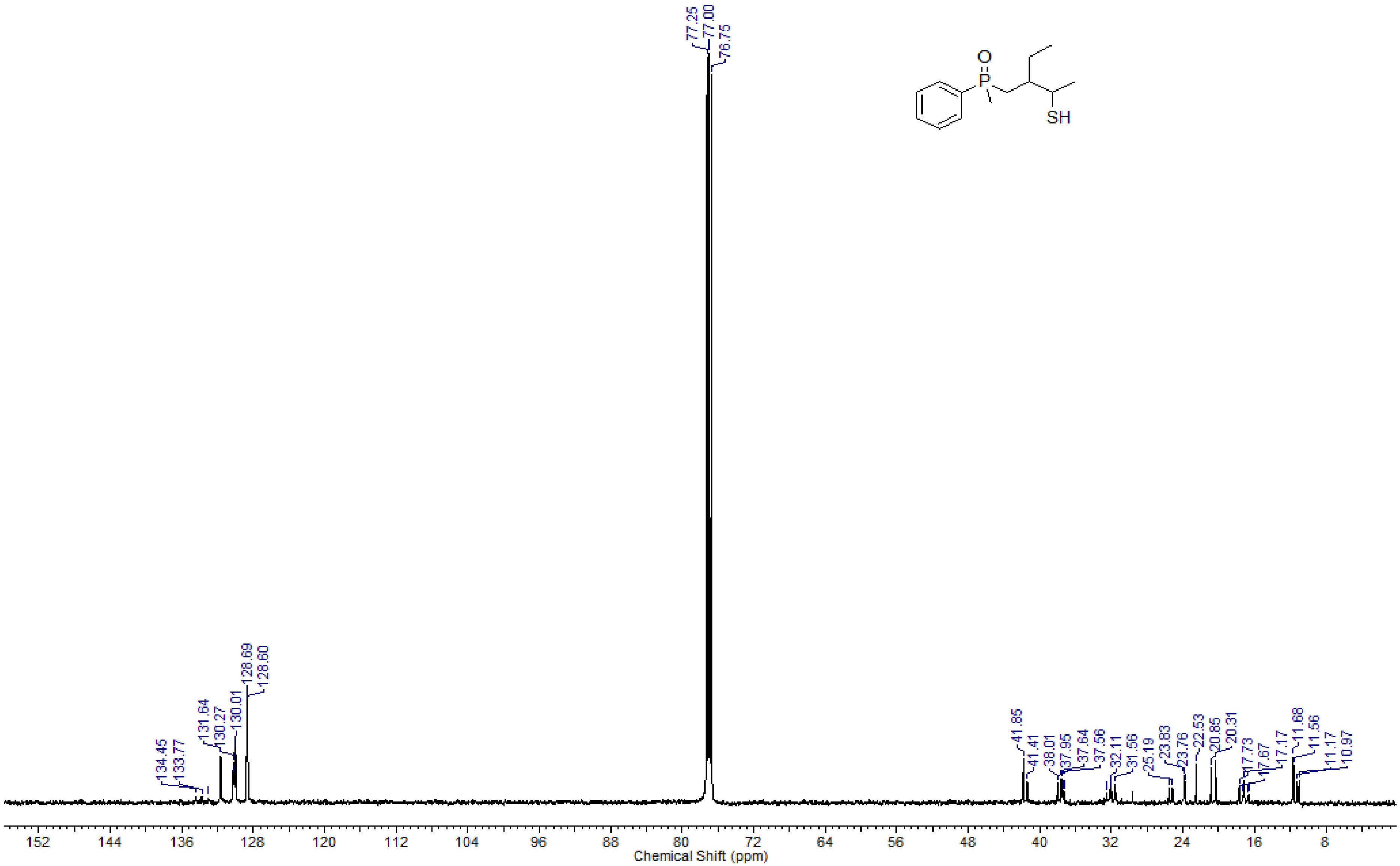

^{13}C NMR spectrum of (2-ethyl-3-mercaptobutyl)methylphenylphosphine oxide (**36**) (CDCl3, 126 MHz).

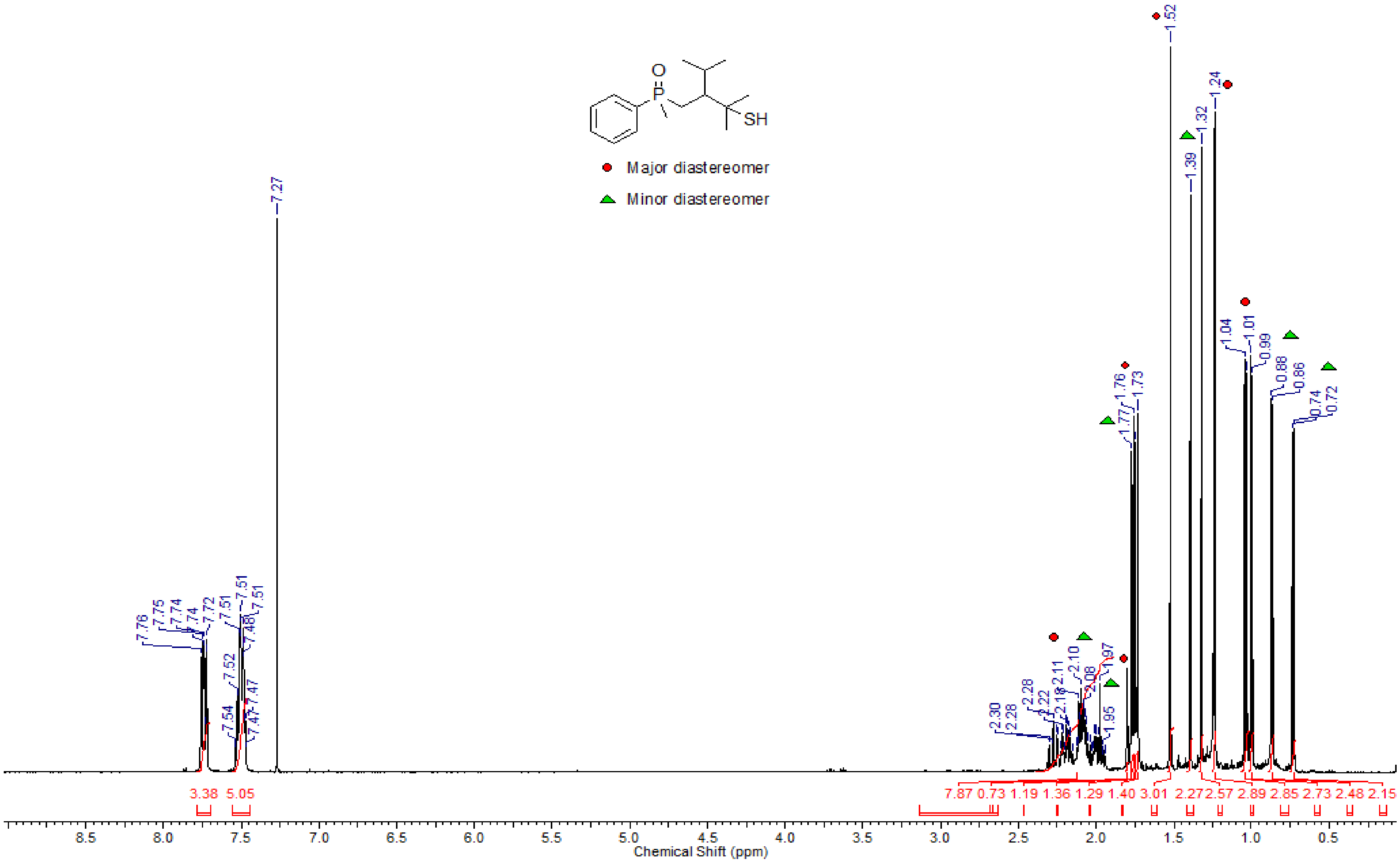

^{1}H NMR spectrum of (2-*iso*propyl-3-methyl-3-mercaptobutyl)methylphenylphosphine oxide (**37**) (CDCl3, 500 MHz).

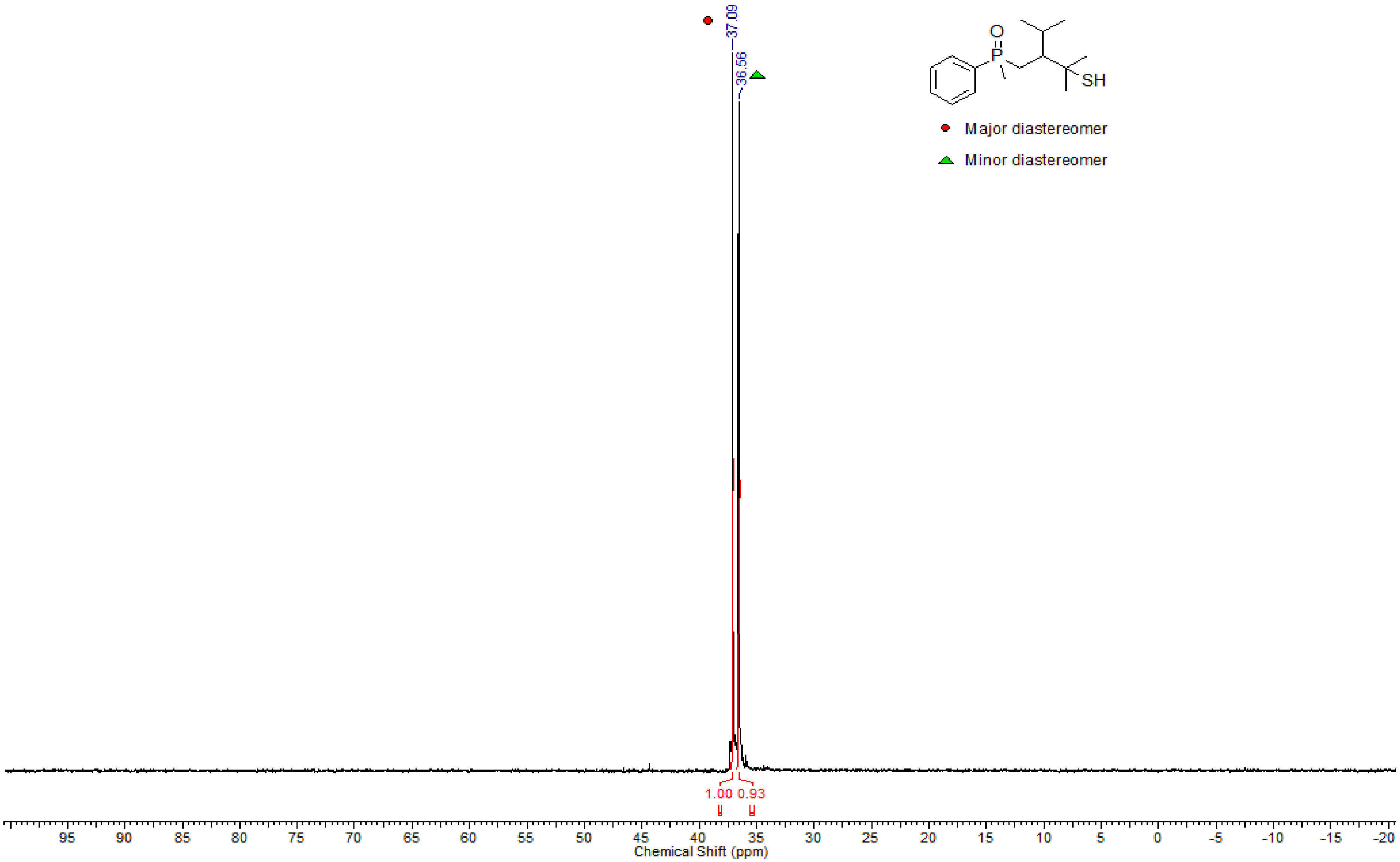

^{31}P NMR spectrum of (2-isopropyl-3-methyl-3-mercaptobutyl)methylphenylphosphine oxide (**37**) ($CDCl_3$, 202 MHz).

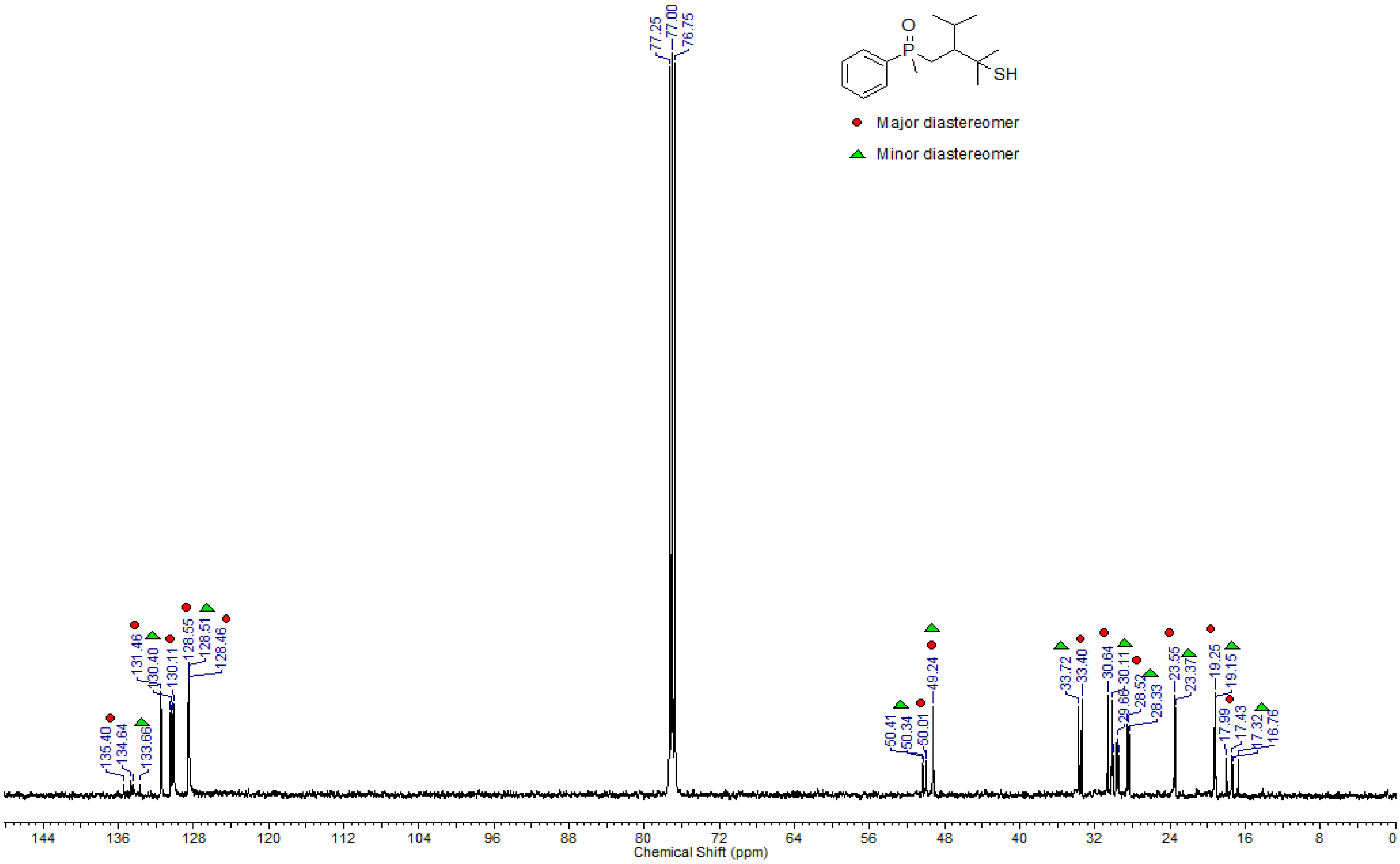

^{13}C NMR spectrum of (2-isopropyl-3-methyl-3-mercaptobutyl)methylphenylphosphine oxide (**37**) (CDCl3, 126 MHz).

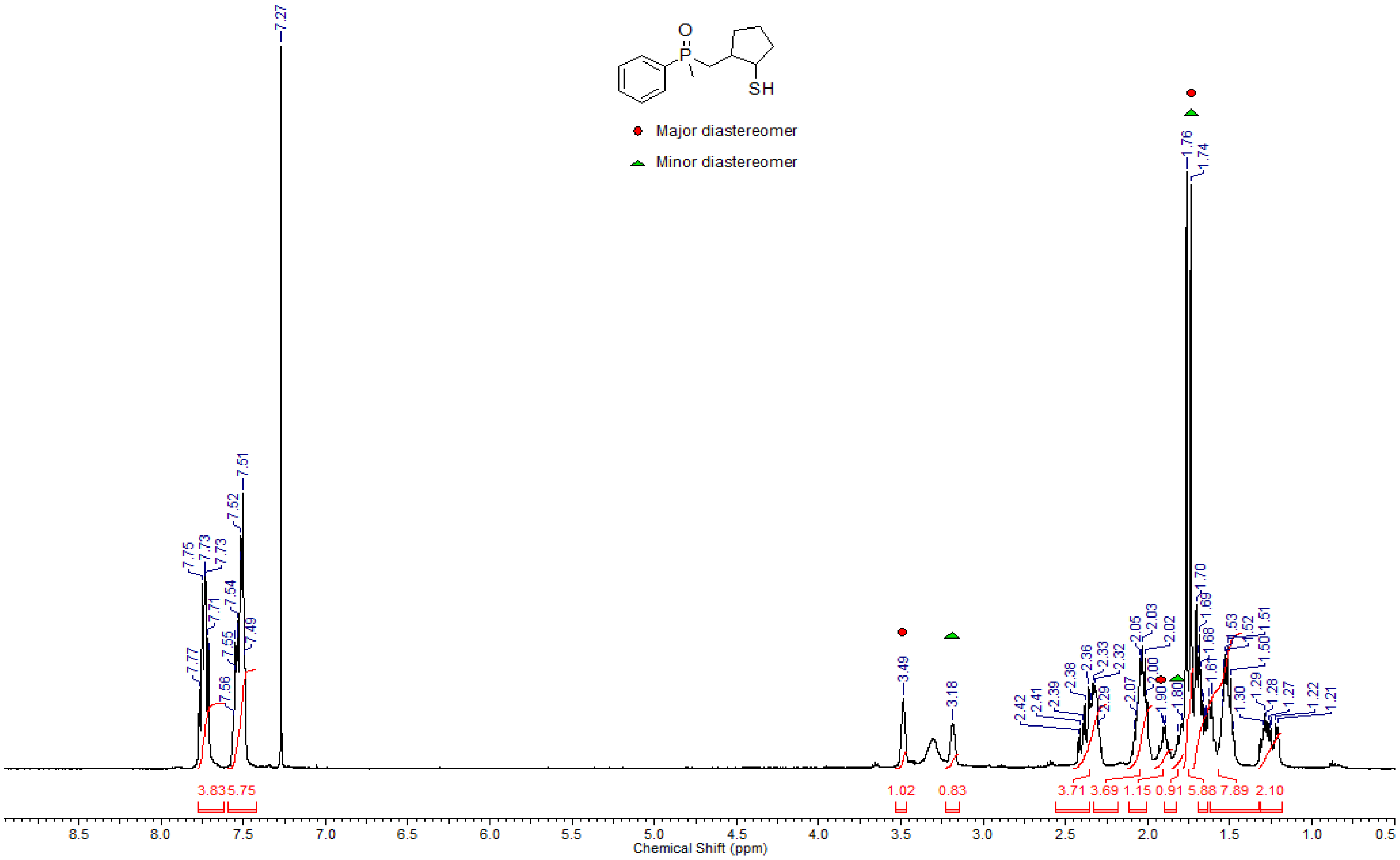

^{1}H NMR spectrum of [(2-mercapto)cyclopentylmethyl)]methylphenylphosphine sulfide (**38**) (CDCl3, 500 MHz).

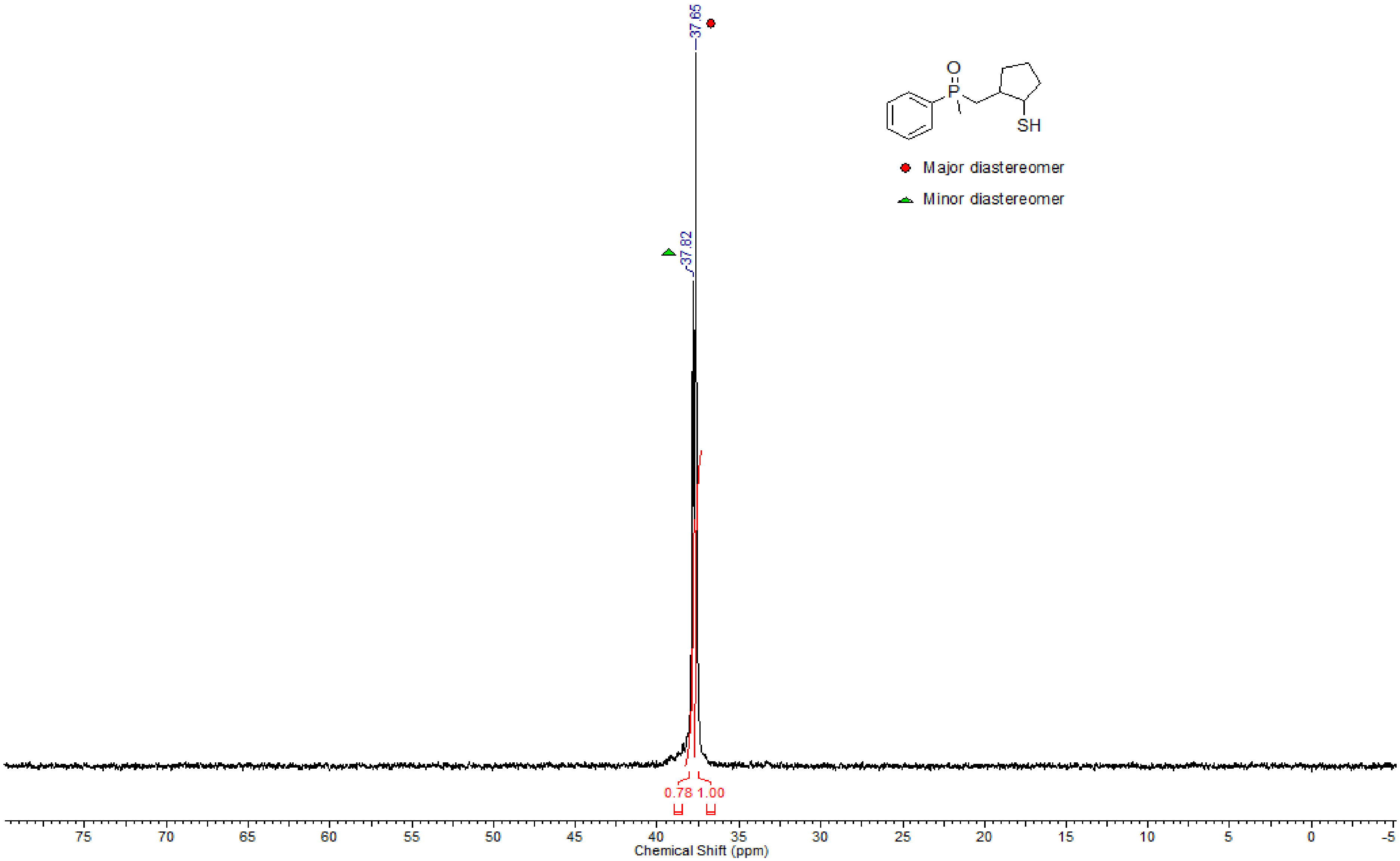

^{31}P NMR spectrum of [(2-mercapto)cyclopentylmethyl)]methylphenylphosphine sulfide (**38**) (CDCl3, 202 MHz).

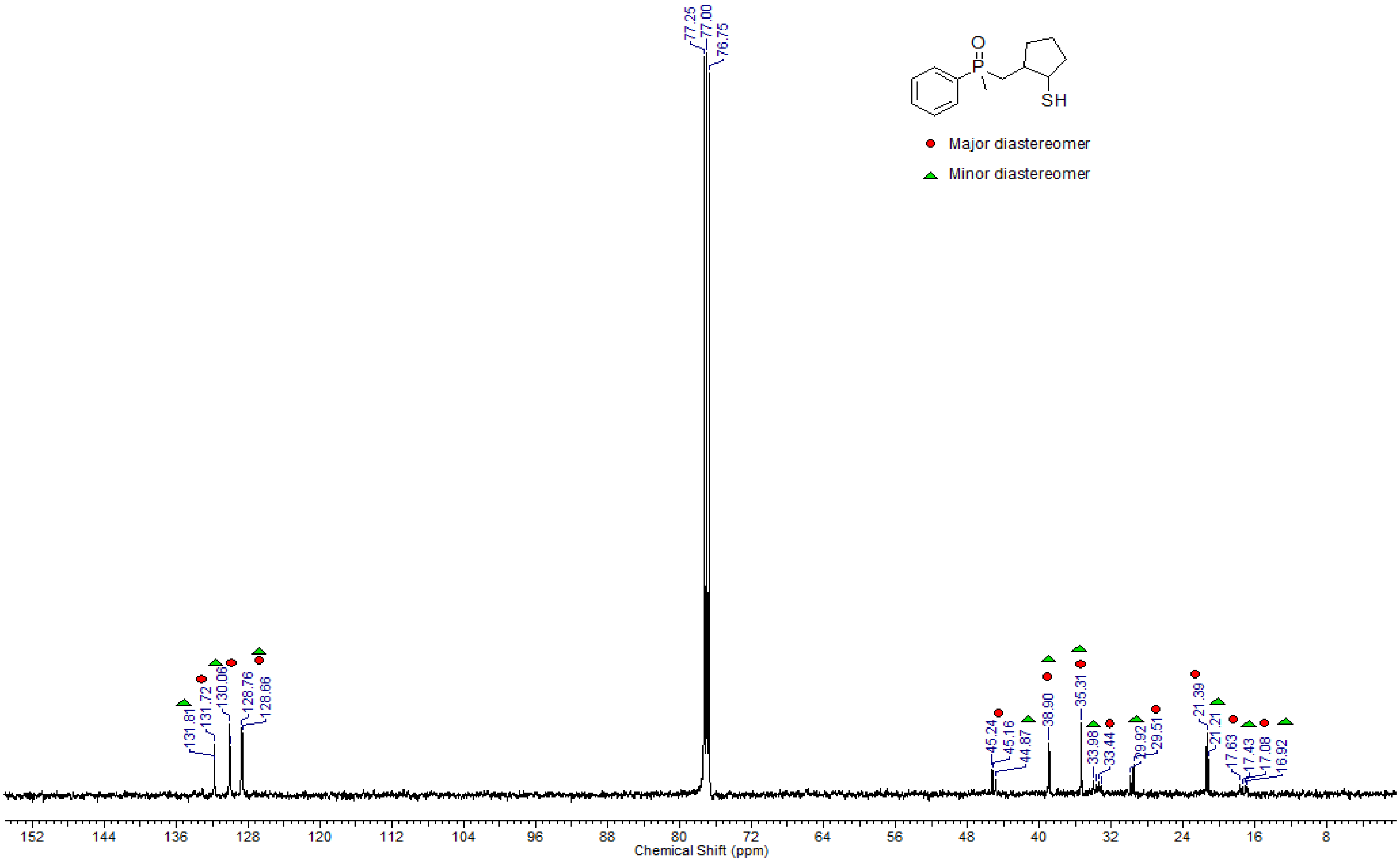

^{13}C NMR spectrum of [(2-mercapto)cyclopentylmethyl)]methylphenylphosphine sulfide (**38**) (CDCl3, 126 MHz).

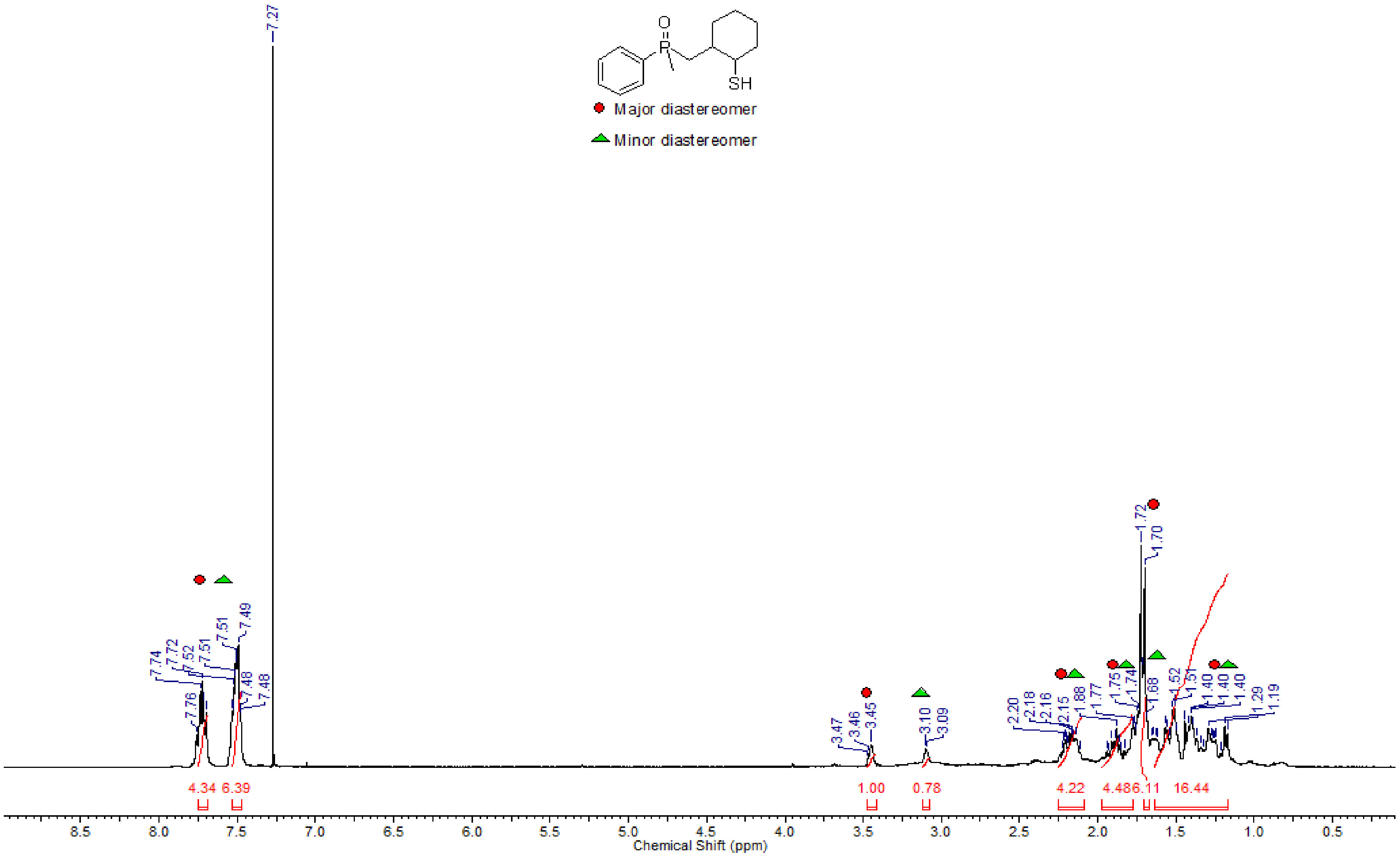

^{1}H NMR spectrum of [(2-mercapto)cyclohexylmethyl)]methylphenylphosphine sulfide (**39**) (CDCl3, 500 MHz).

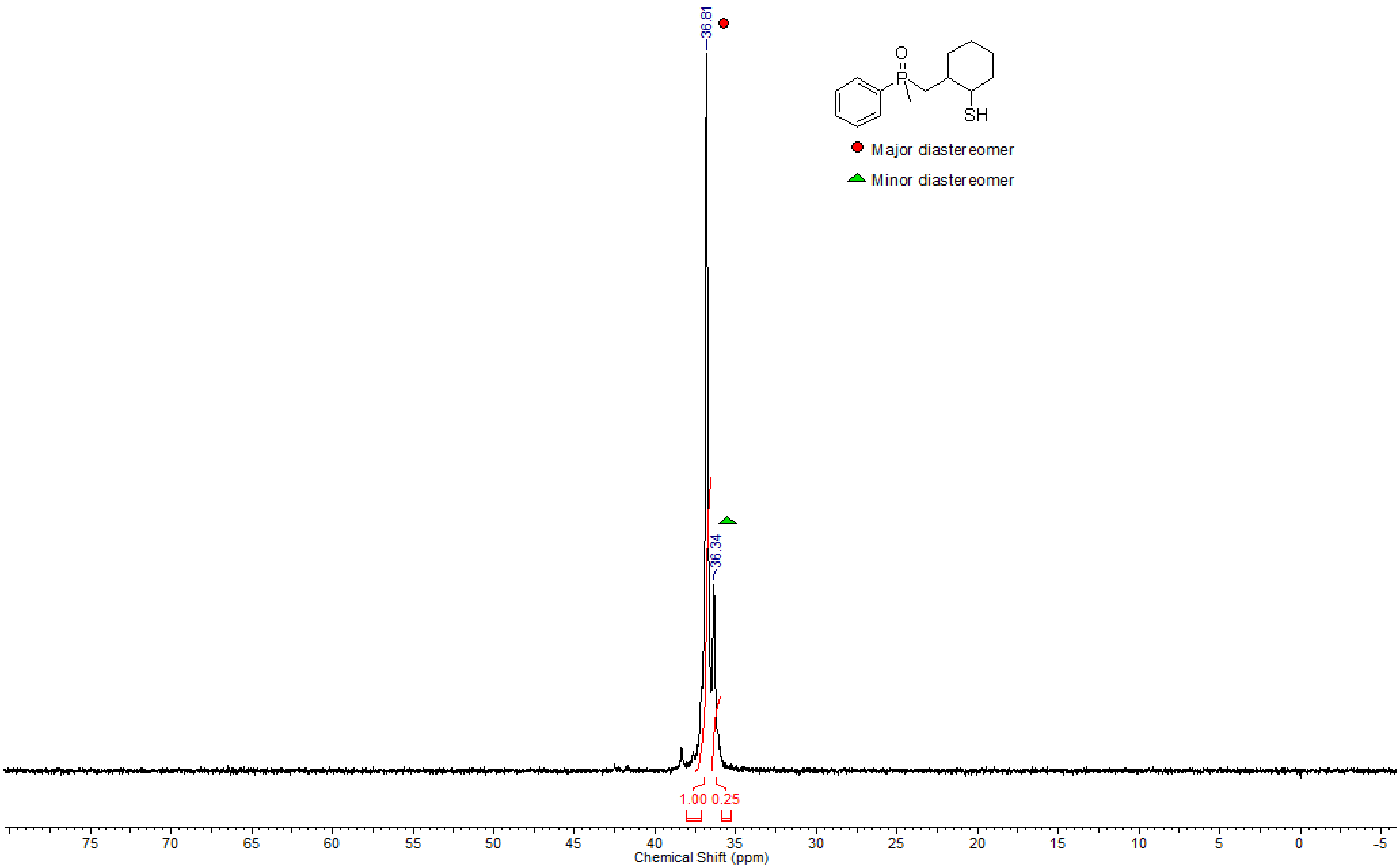

^{31}P NMR spectrum of [(2-mercapto)cyclohexylmethyl)]methylphenylphosphine sulfide (**39**) (CDCl3, 202 MHz).

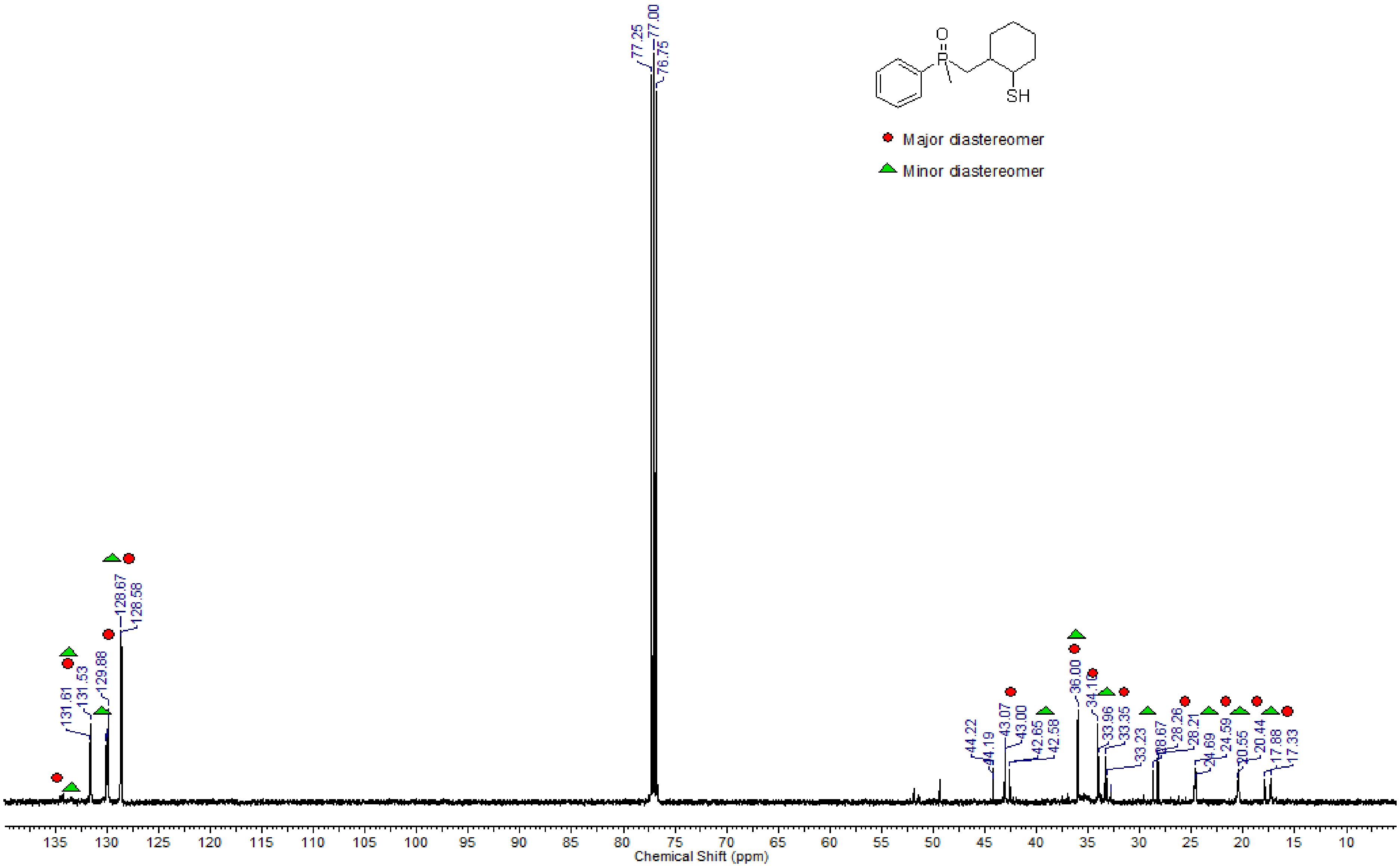

^{13}C NMR spectrum of [(2-mercapto)cyclohexylmethyl)]methylphenylphosphine sulfide (**39**) (CDCl3, 126 MHz).

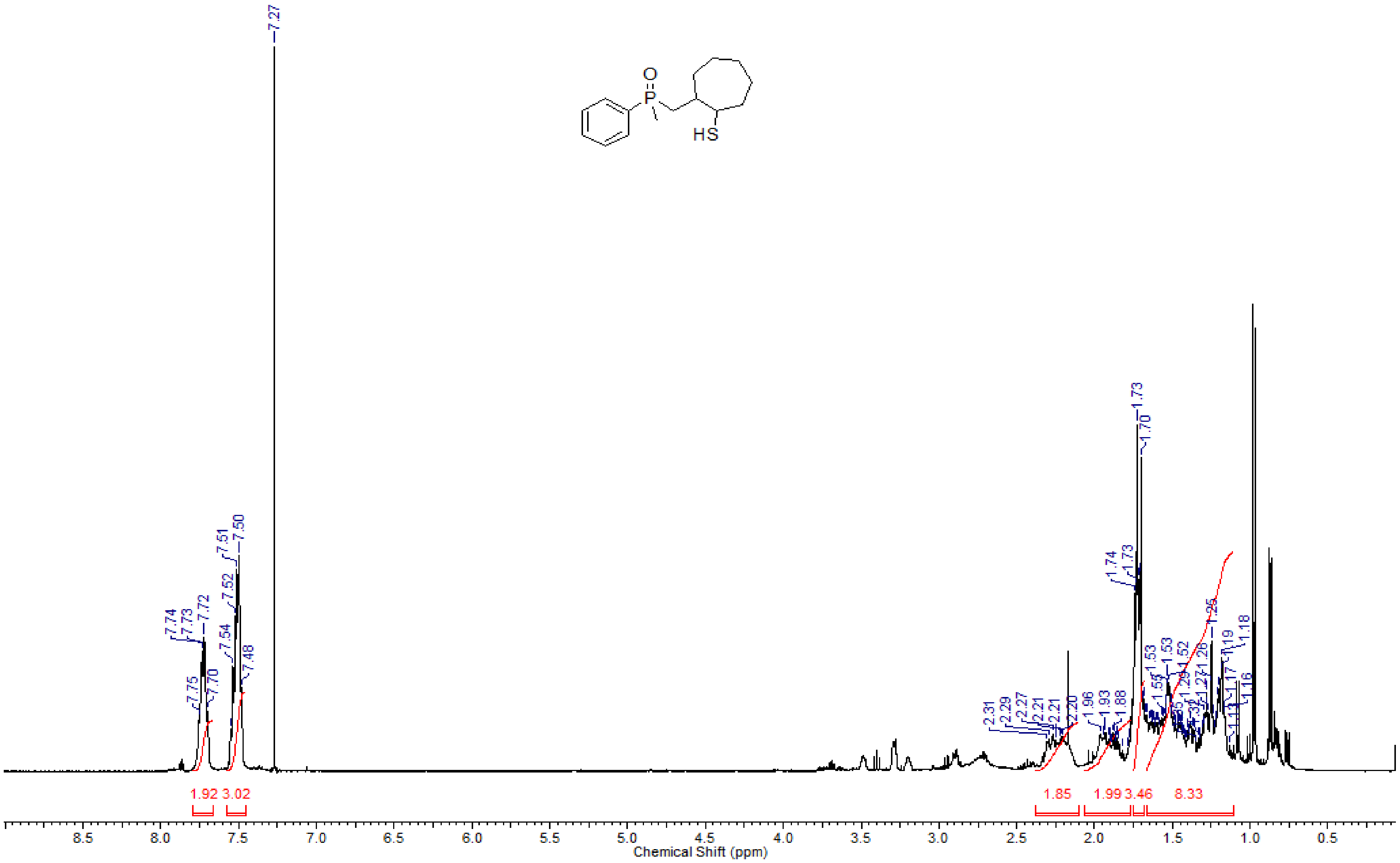

^{1}H NMR spectrum of [(2-mercapto)cycloheptylmethyl)]methylphenylphosphine sulfide (**40**) (CDCl3, 500 MHz).

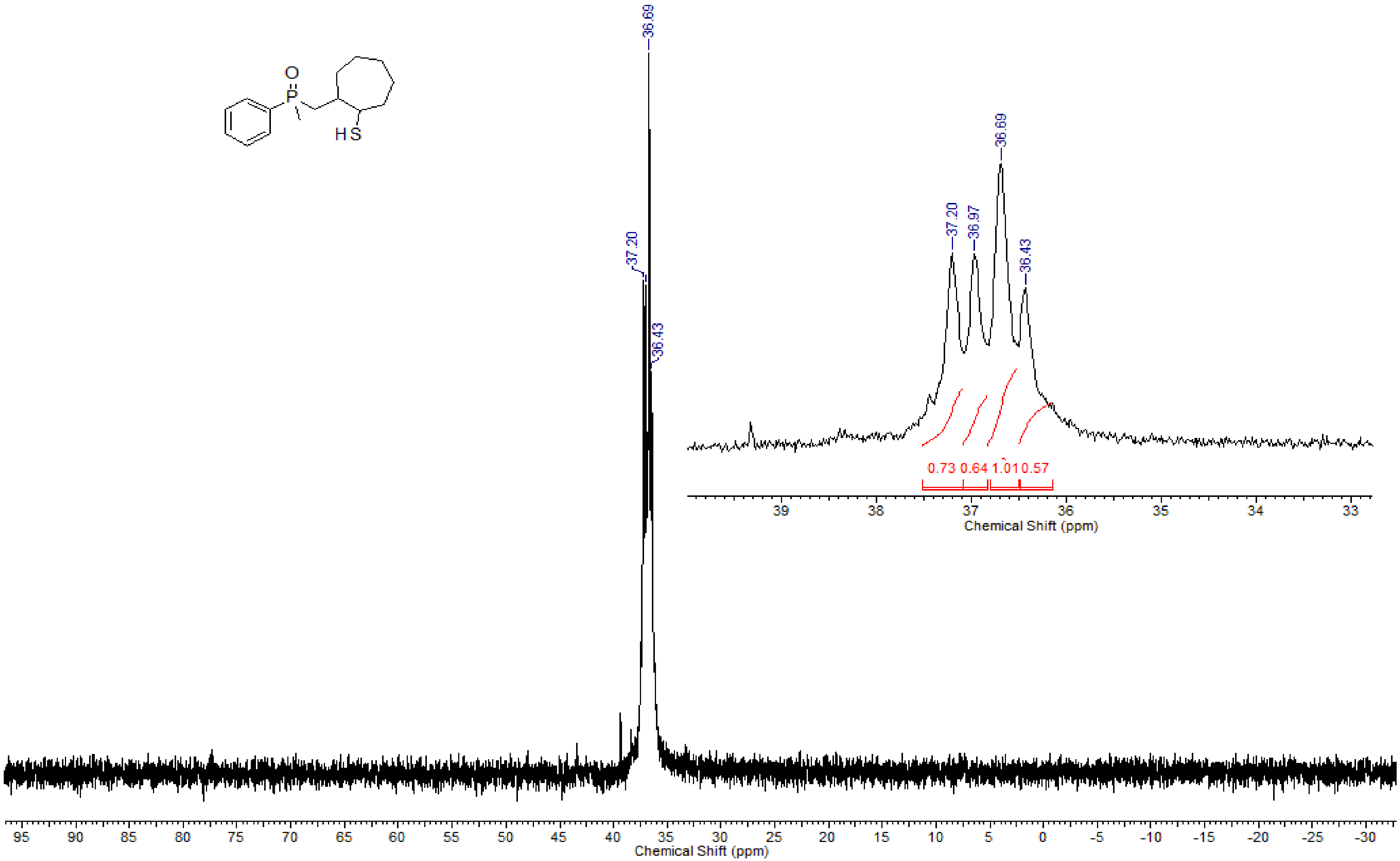

^{31}P NMR spectrum of [(2-mercapto)cycloheptylmethyl)]methylphenylphosphine sulfide (**40**) (CDCl3, 202 MHz).

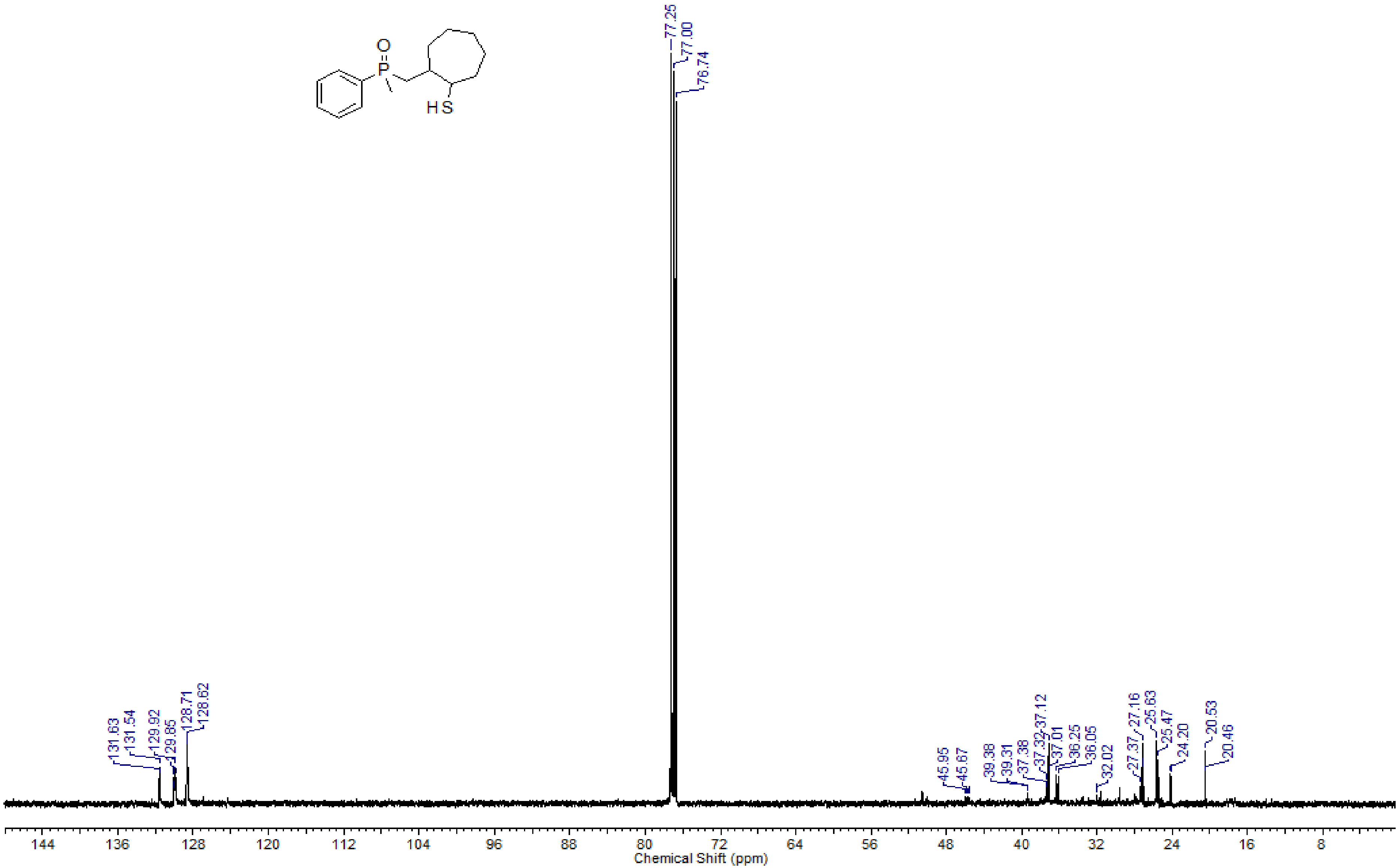

^{13}C NMR spectrum of [(2-mercapto)cycloheptylmethyl)]methylphenylphosphine sulfide (**40**) (CDCl3, 126 MHz).

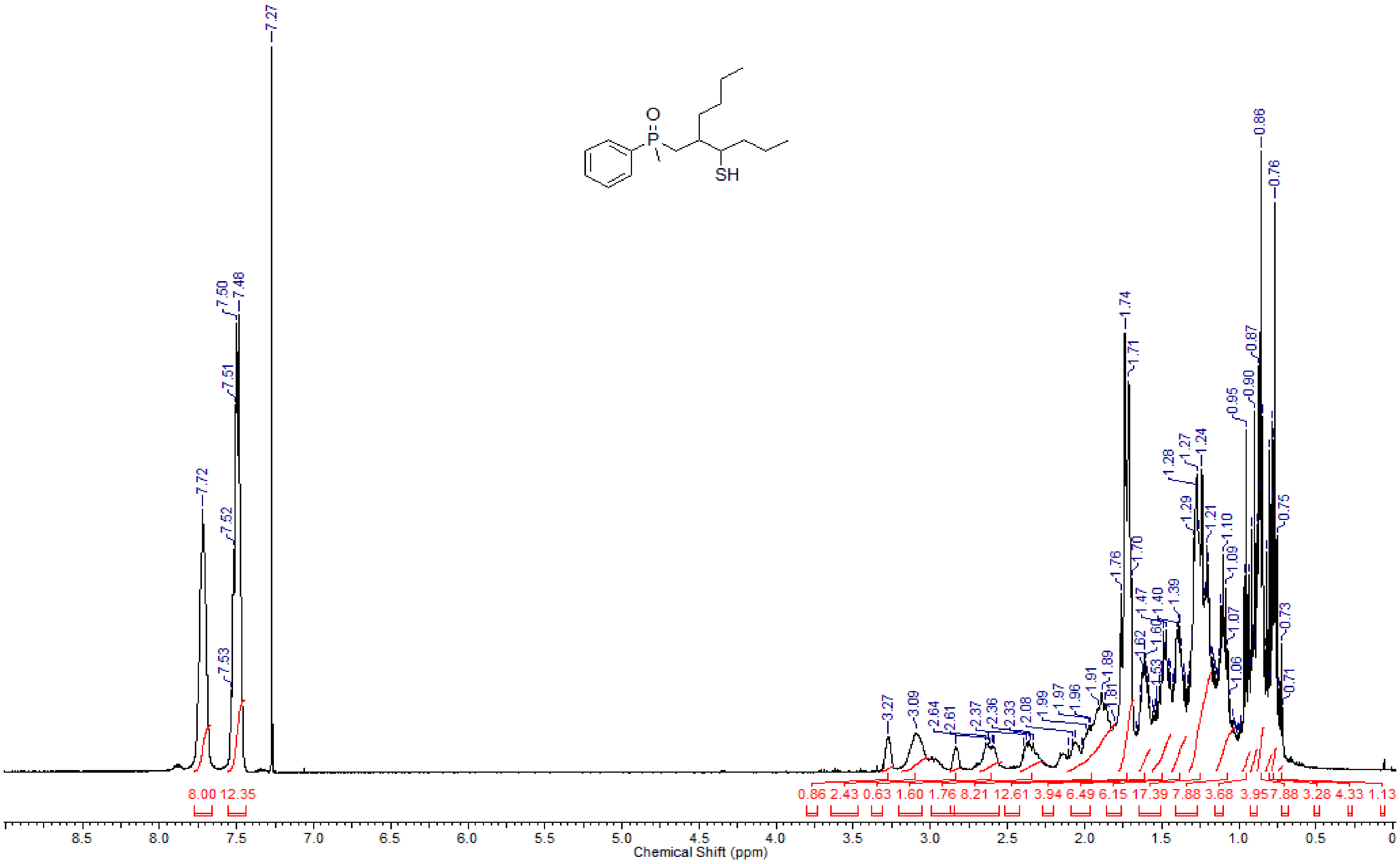

^{1}H NMR spectrum of (2-butyl-3-mercaptohexyl)methylphenylphosphine oxide (**41**) (CDCl3, 500 MHz).

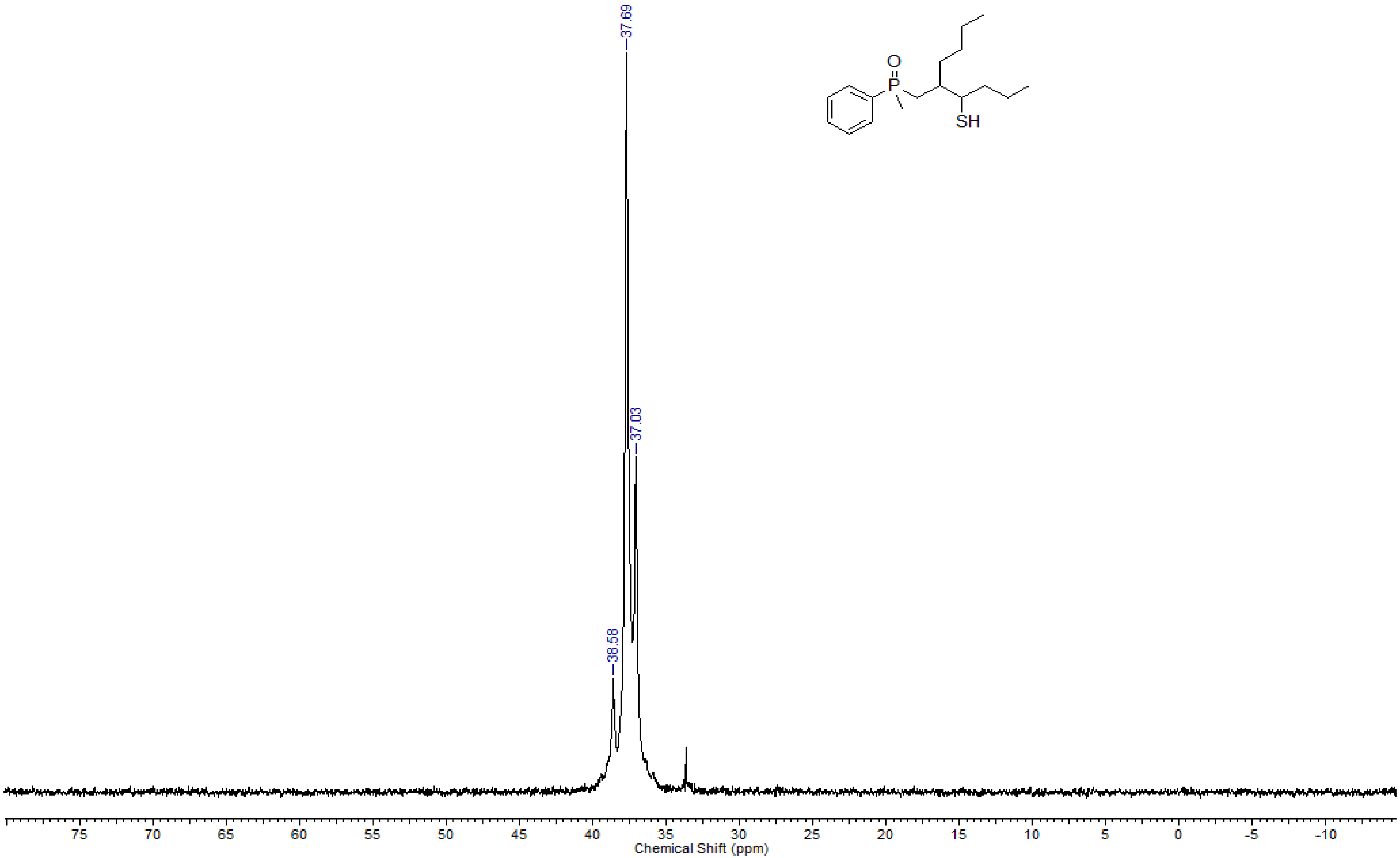

^{31}P NMR spectrum of (2-butyl-3-mercaptohexyl)methylphenylphosphine oxide (**41**) (CDCl3, 202 MHz).

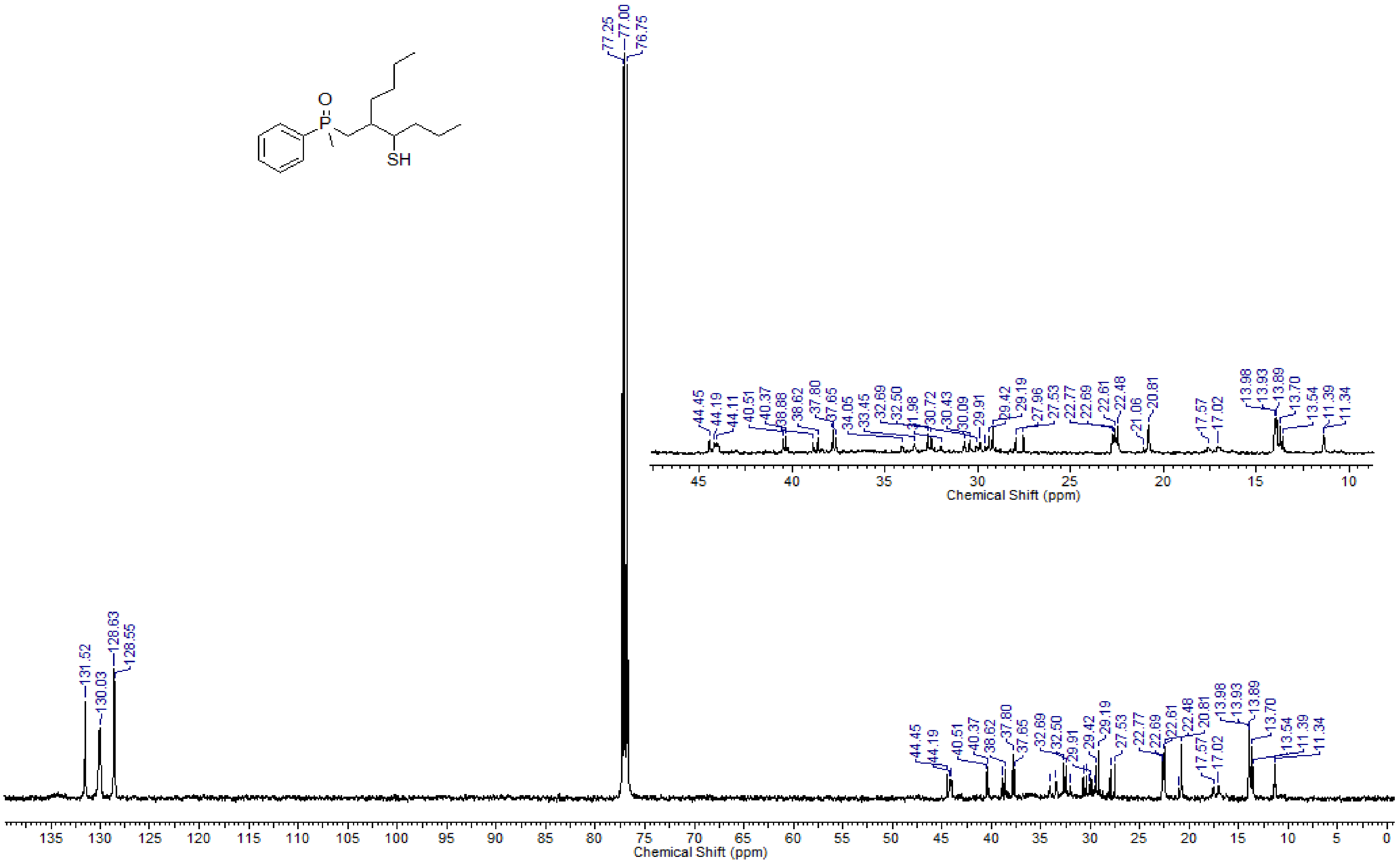

^{13}C NMR spectrum of (2-butyl-3-mercaptohexyl)methylphenylphosphine oxide (**41**) (CDCl3, 126 MHz).

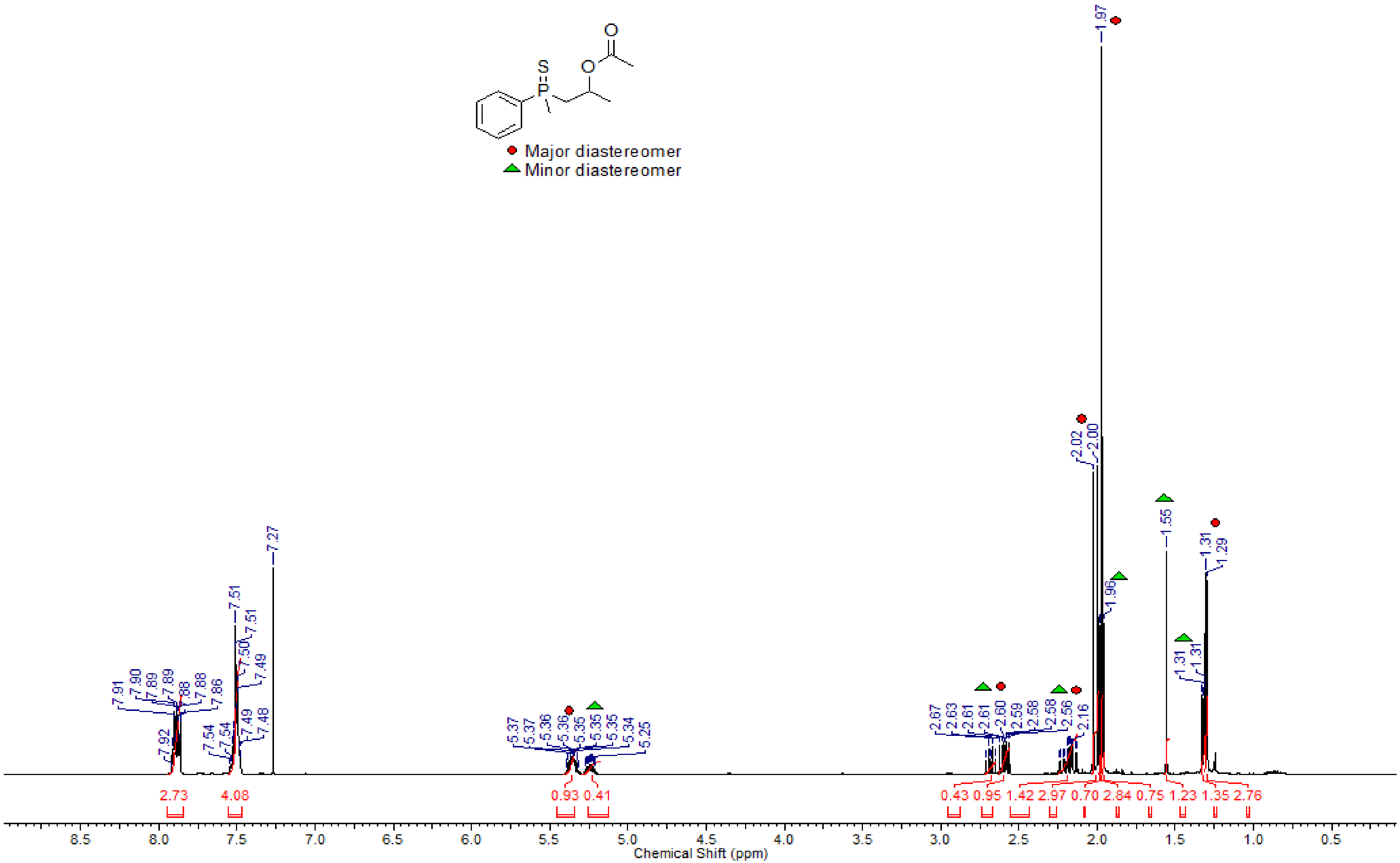

^{1}H NMR spectrum of (2-acetoxypropyl)methylphenylphosphine sulfide (**42**) (CDCl3, 500 MHz).

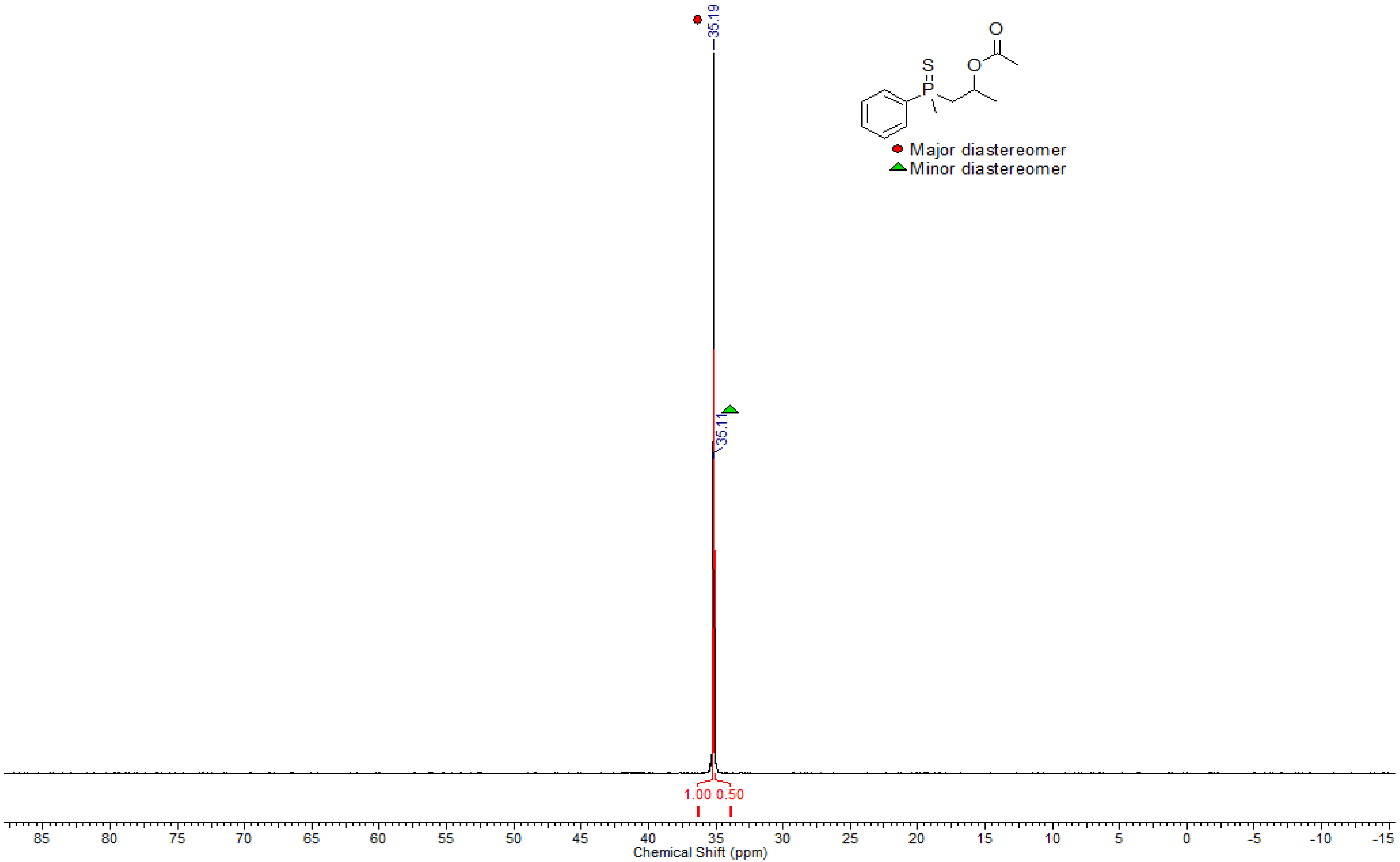

^{31}P NMR spectrum of (2-acetoxypropyl)methylphenylphosphine sulfide (**42**) (CDCl3, 202 MHz).

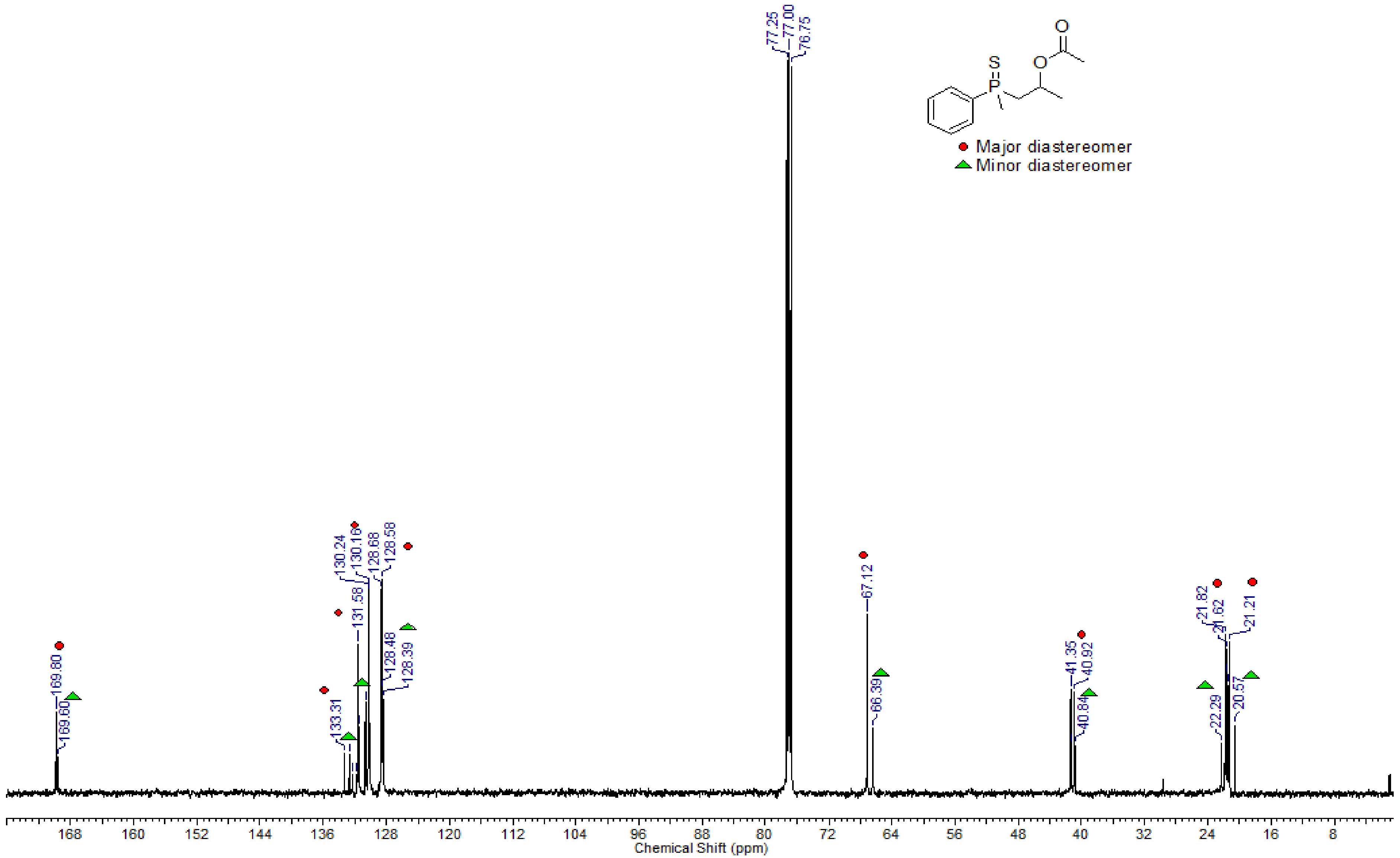

^{13}C NMR spectrum of (2-acetoxypropyl)methylphenylphosphine sulfide (**42**) ($CDCl_3$, 126 MHz).

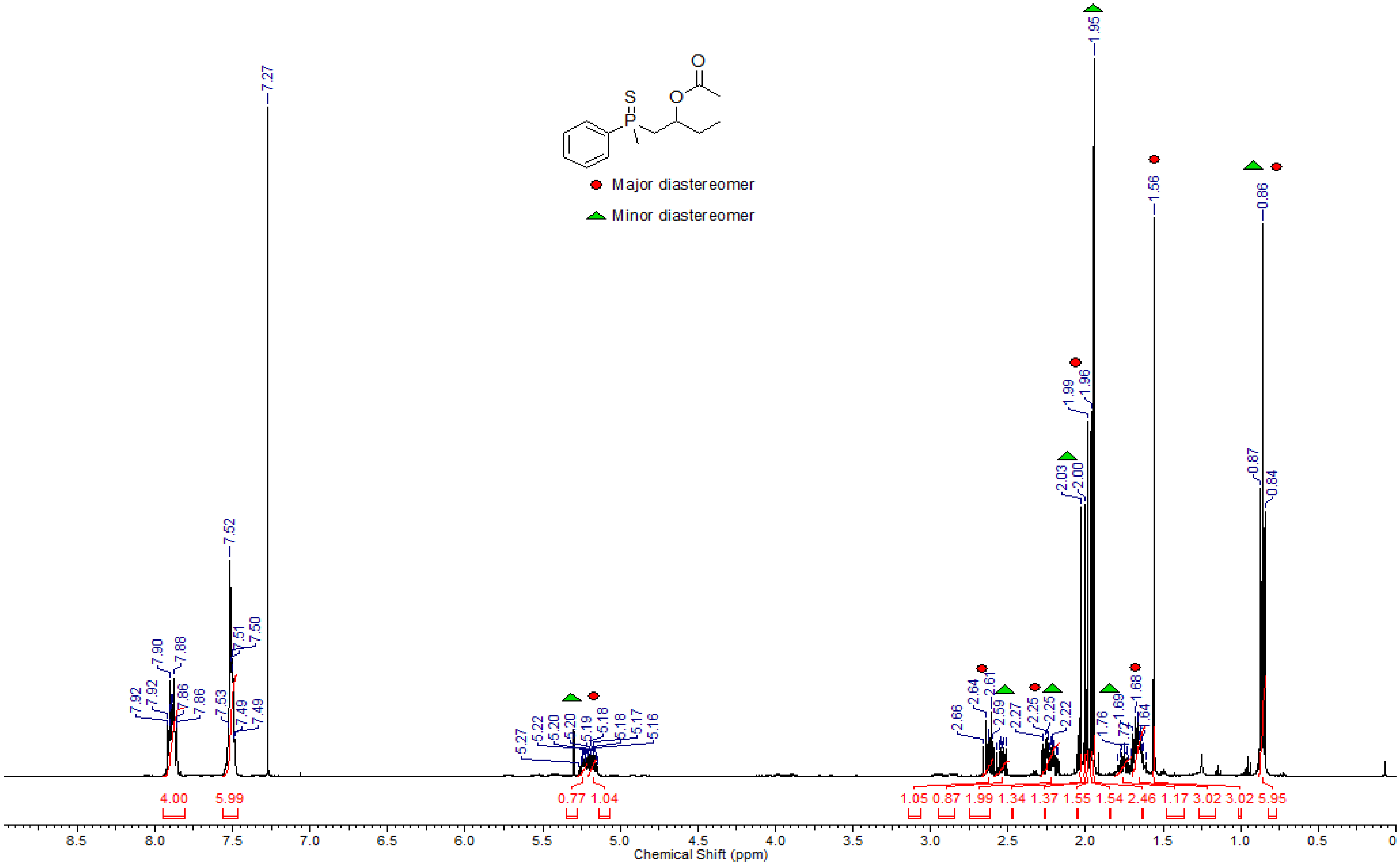

^{1}H NMR spectrum of (2-acetoxybutyl)methylphenylphosphine sulfide (**43**) (CDCl3, 500 MHz).

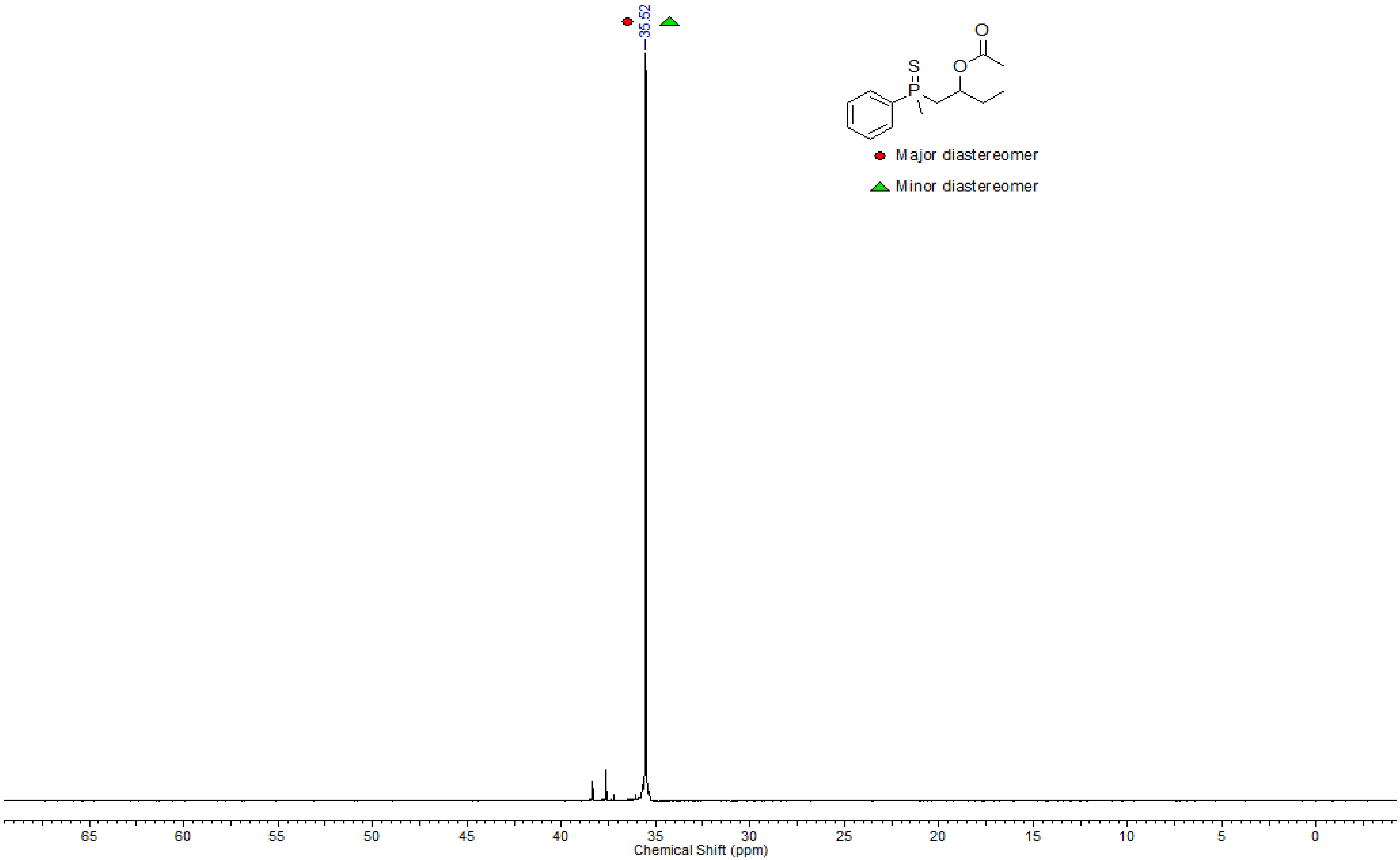

^{31}P NMR spectrum of (2-acetoxybutyl)methylphenylphosphine sulfide (**43**) (CDCl3, 202 MHz).

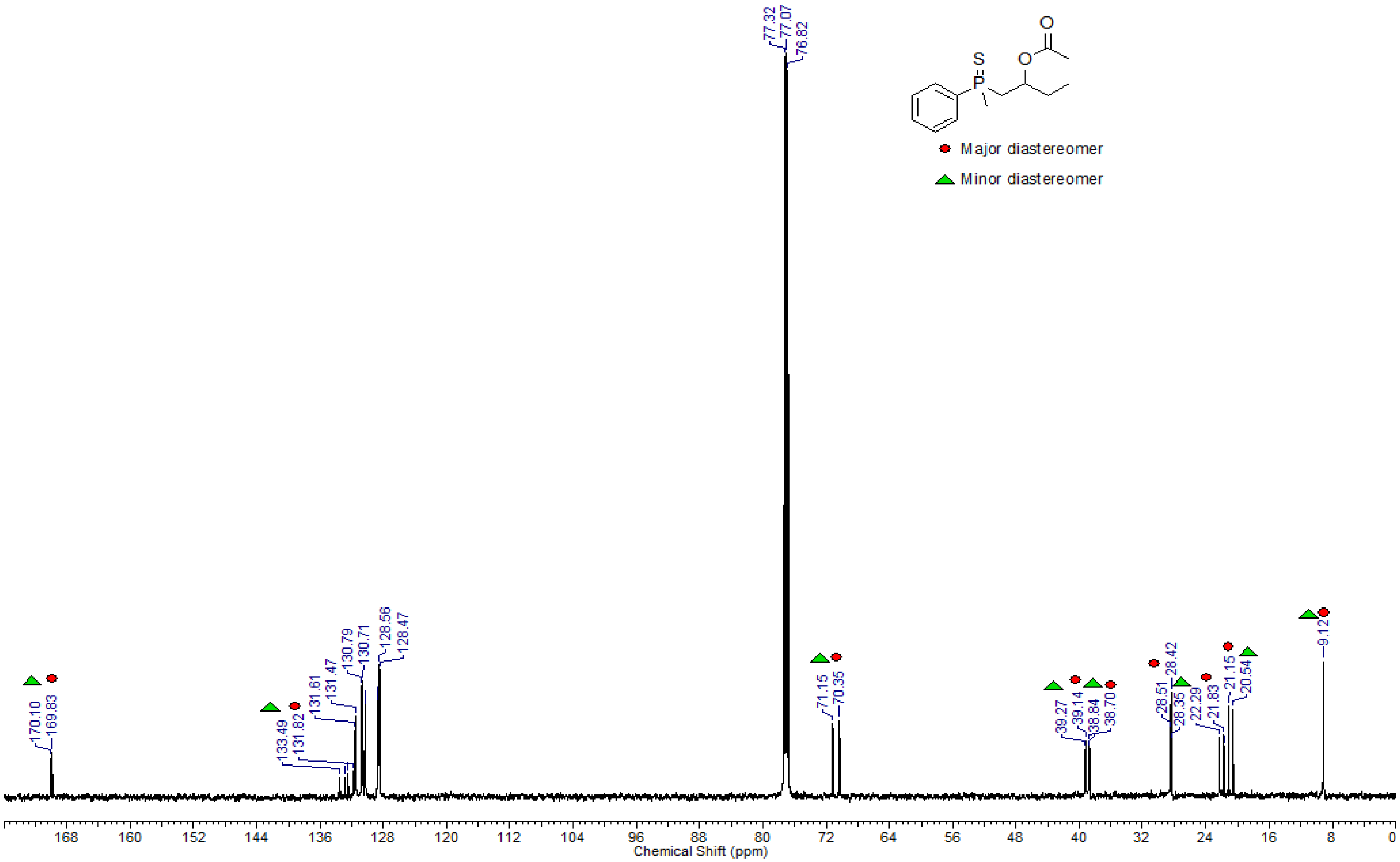

^{13}C NMR spectrum of (2-acetoxybutyl)methylphenylphosphine sulfide (**43**) (CDCl3, 126 MHz).

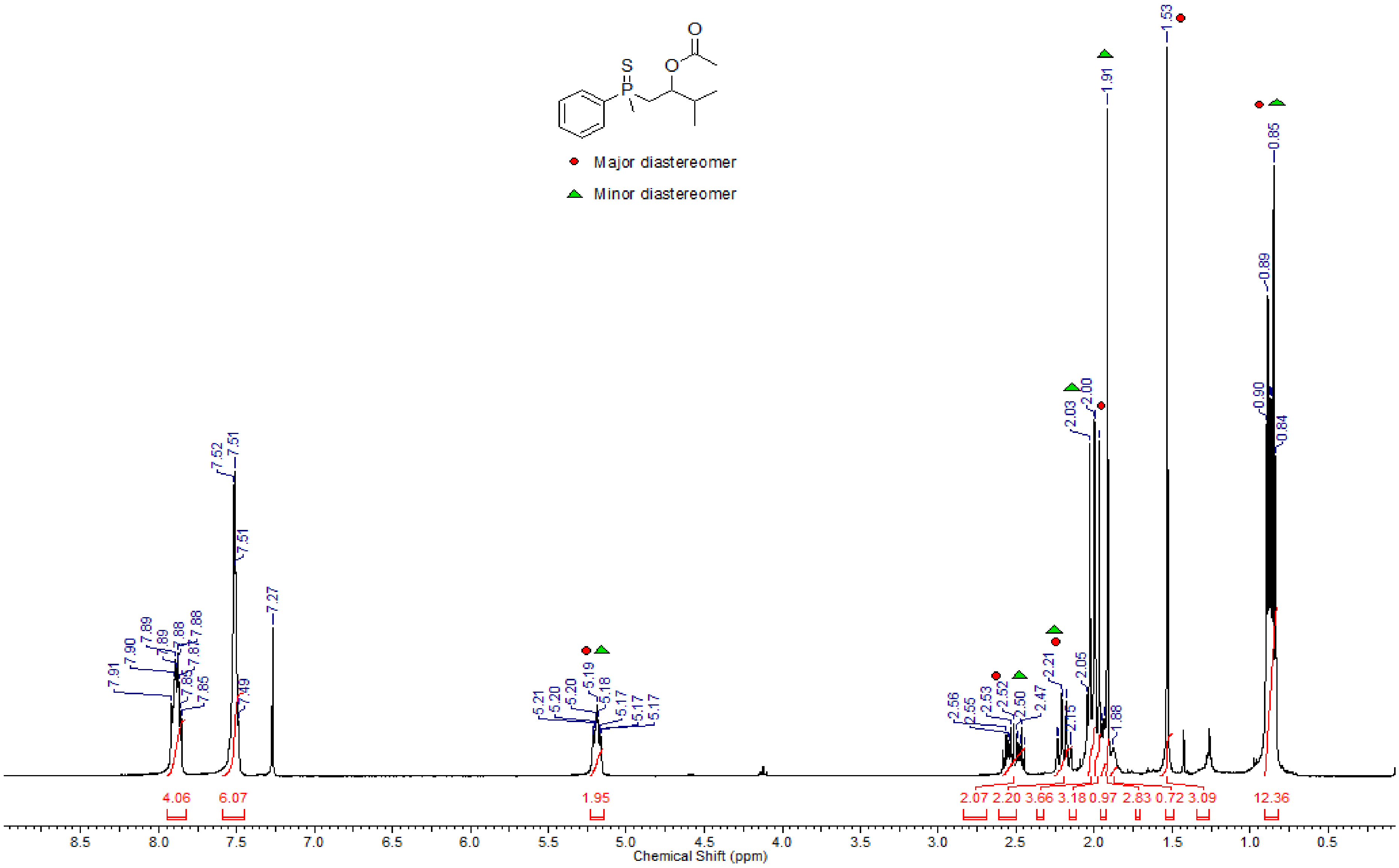

^{1}H NMR spectrum of (2-acetoxy-3-methylbutyl)methylphenylphosphine sulfide (**44**) (CDCl3, 500 MHz).

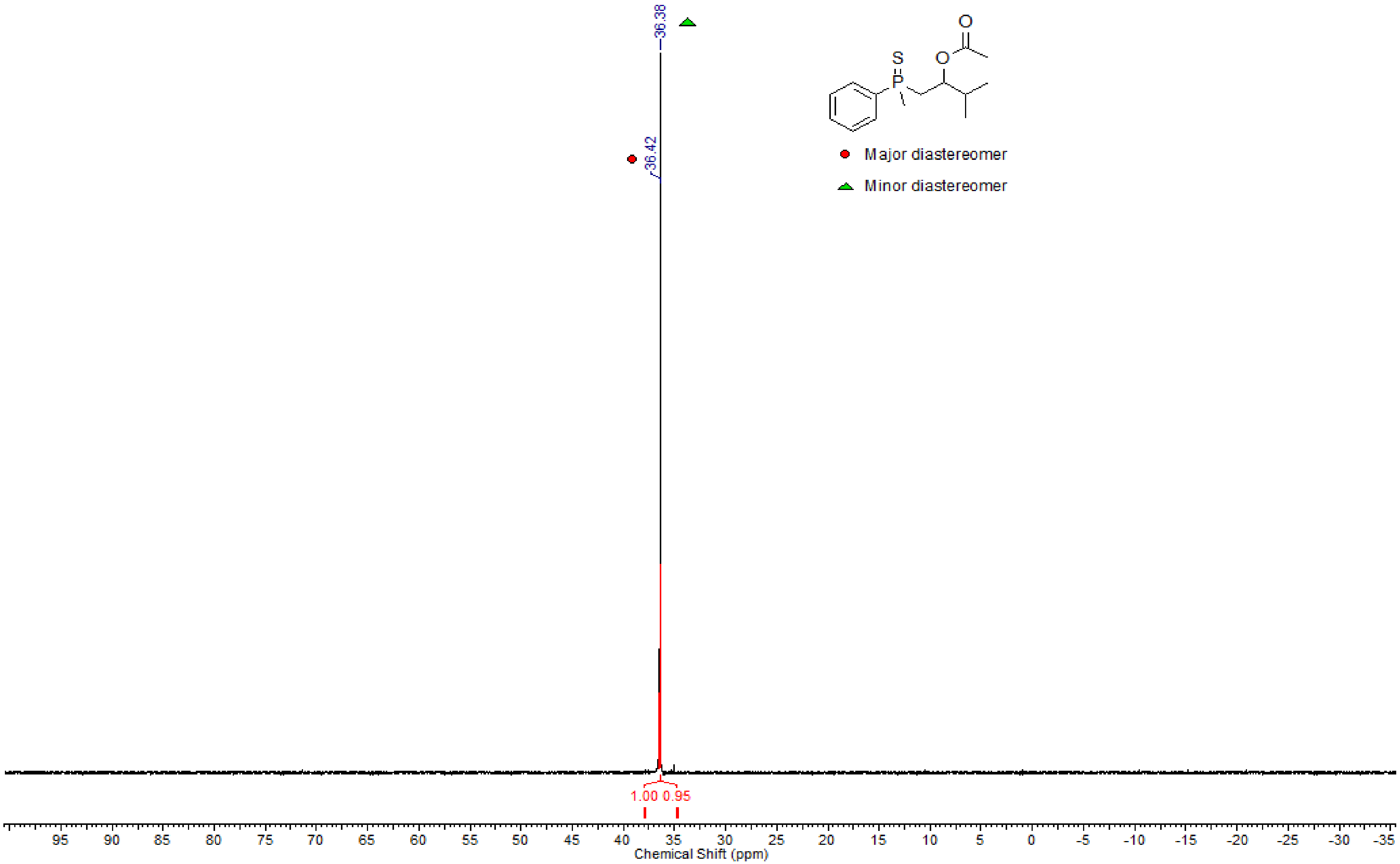

^{31}P NMR spectrum of (2-acetoxy-3-methylbutyl)methylphenylphosphine sulfide (**44**) (CDCl3, 202 MHz).

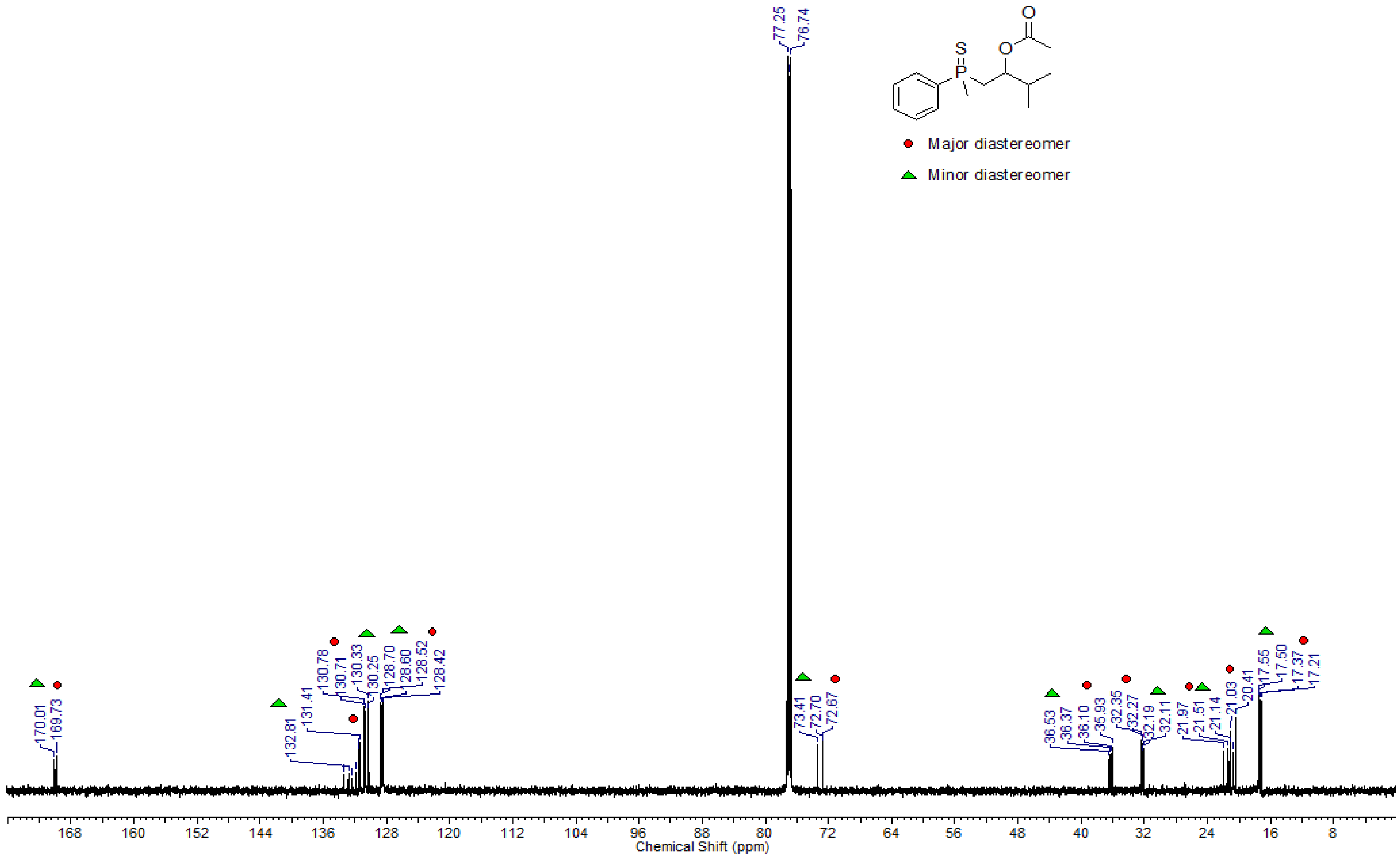

^{13}C NMR spectrum of (2-acetoxy-3-methylbutyl)methylphenylphosphine sulfide (**44**) (CDCl3, 126 MHz).

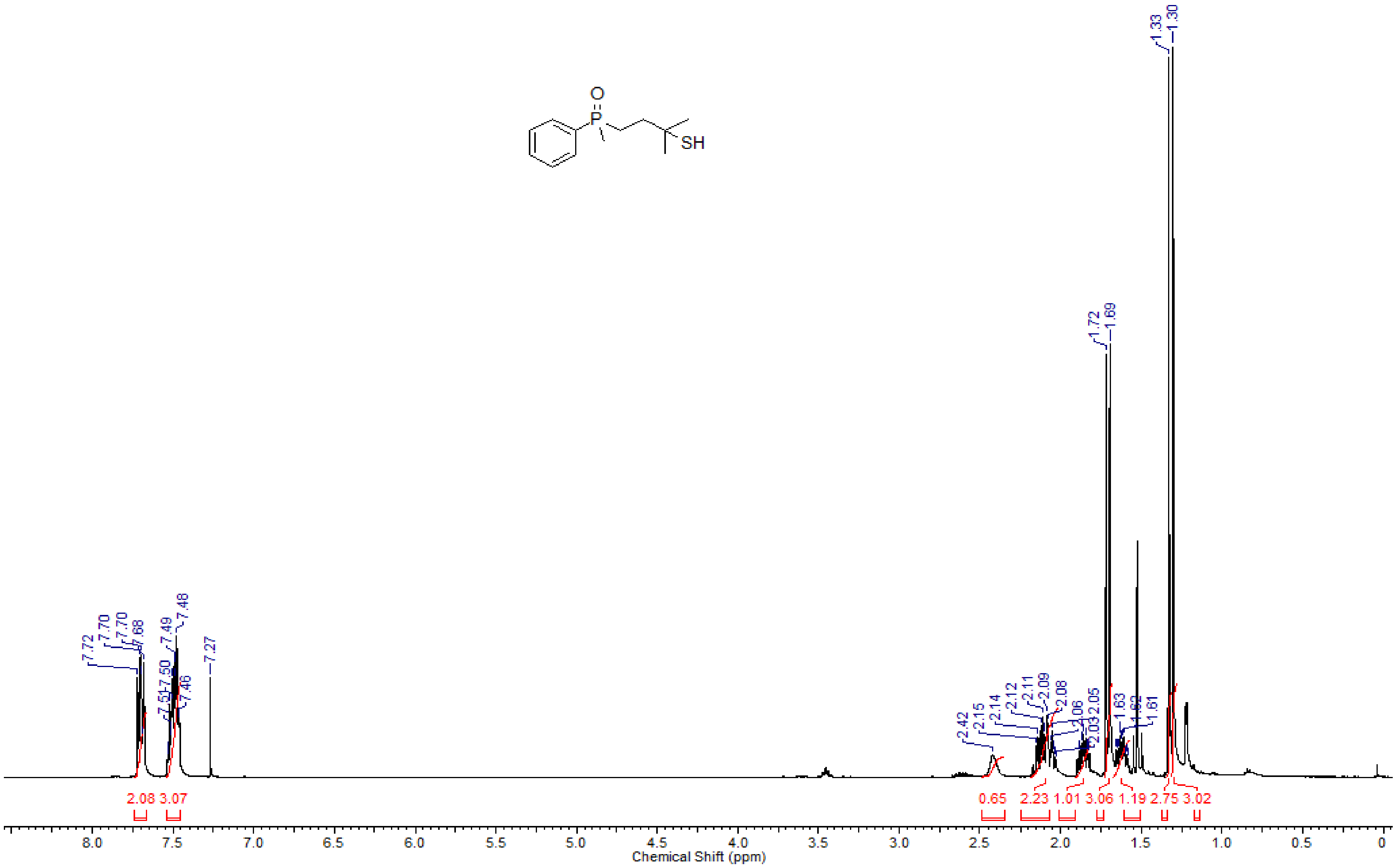

^{1}H NMR spectrum of (3-methyl-3-mercaptobutyl)methylphenylphosphine oxide (**45**) (CDCl3, 500 MHz).

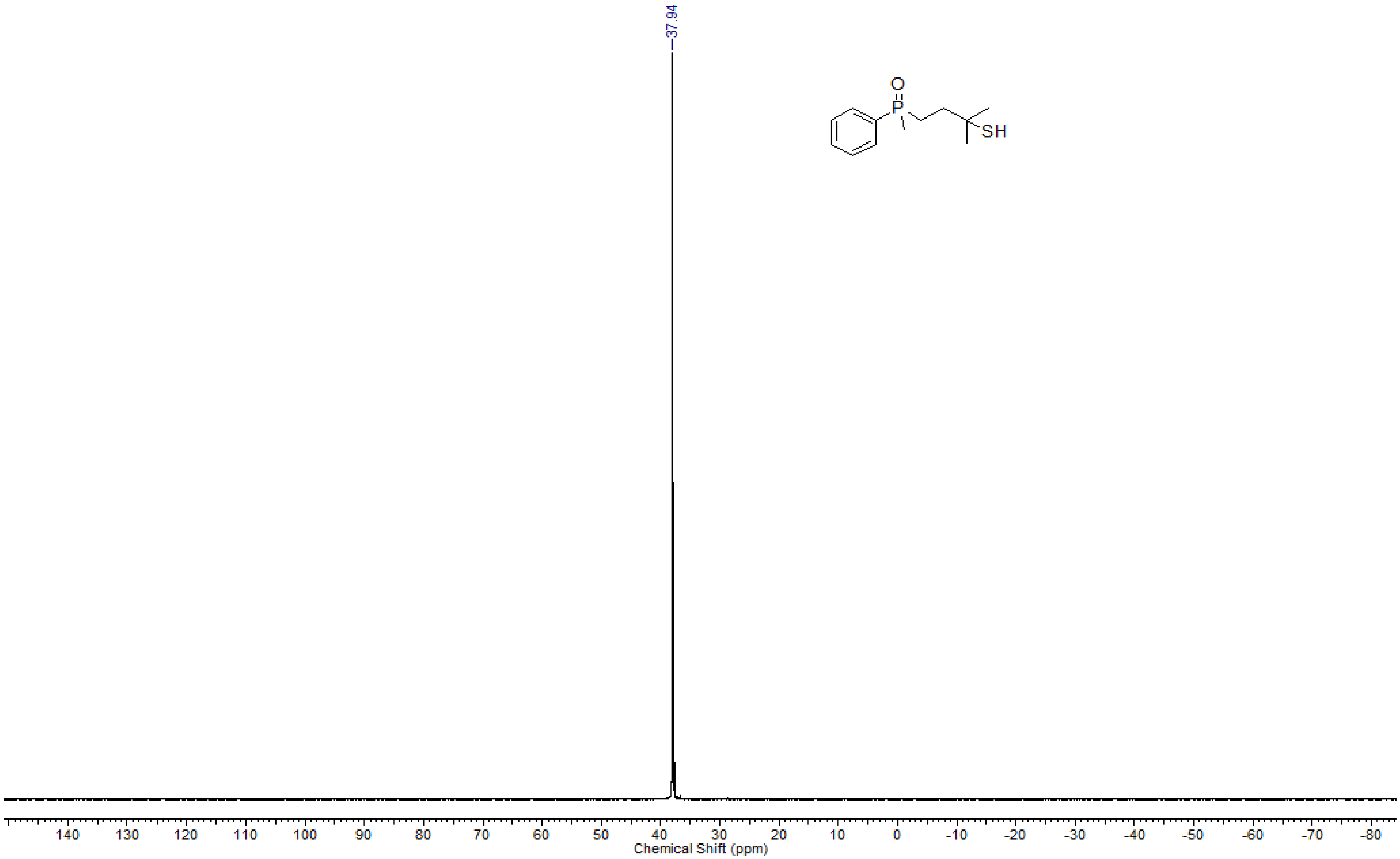

^{31}P NMR spectrum of (3-methyl-3-mercaptobutyl)methylphenylphosphine oxide (**45**) (CDCl3, 202 MHz).

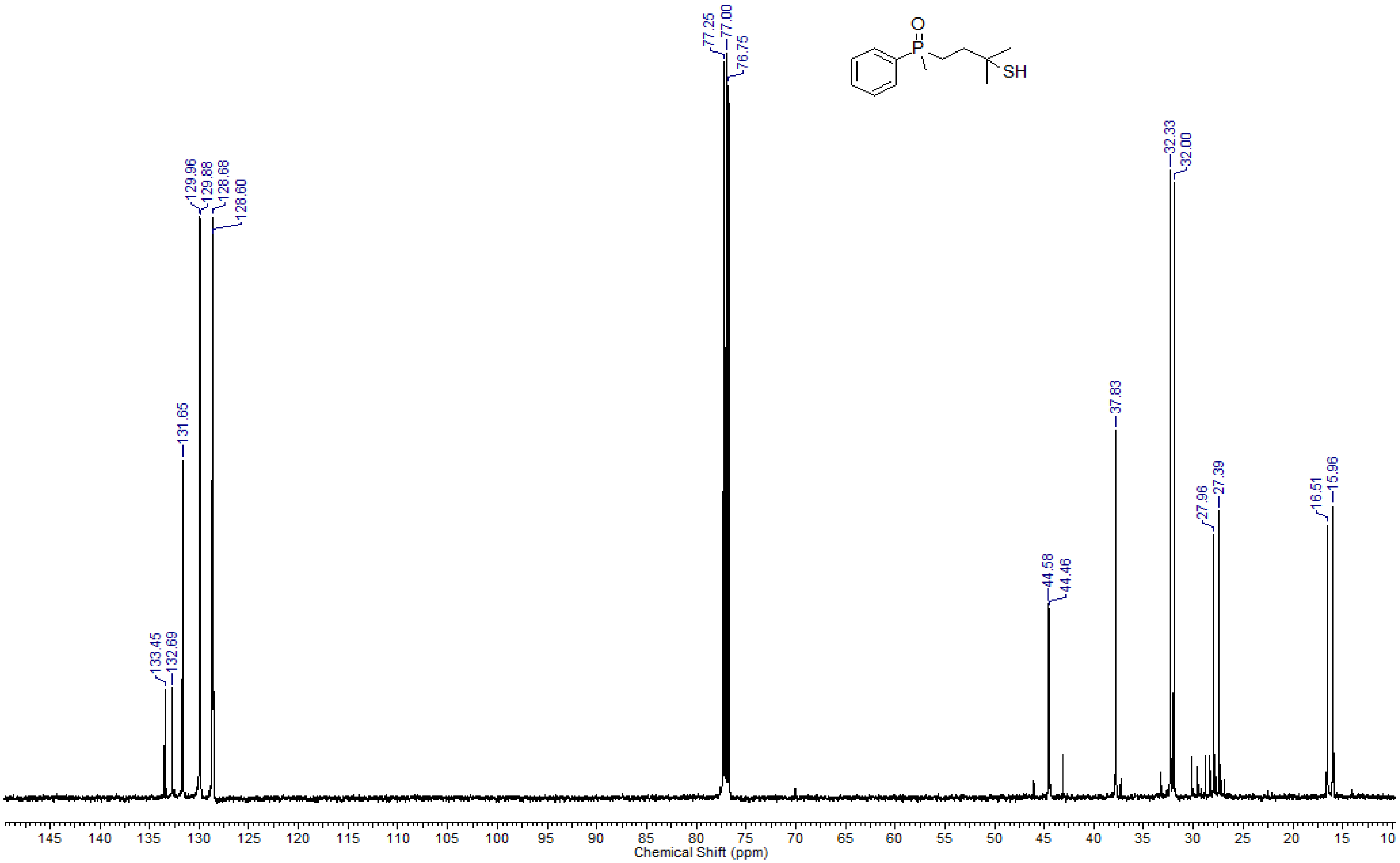

^{13}C NMR spectrum of (3-methyl-3-mercaptobutyl)methylphenylphosphine oxide (**45**) ($CDCl3$, 126 MHz).

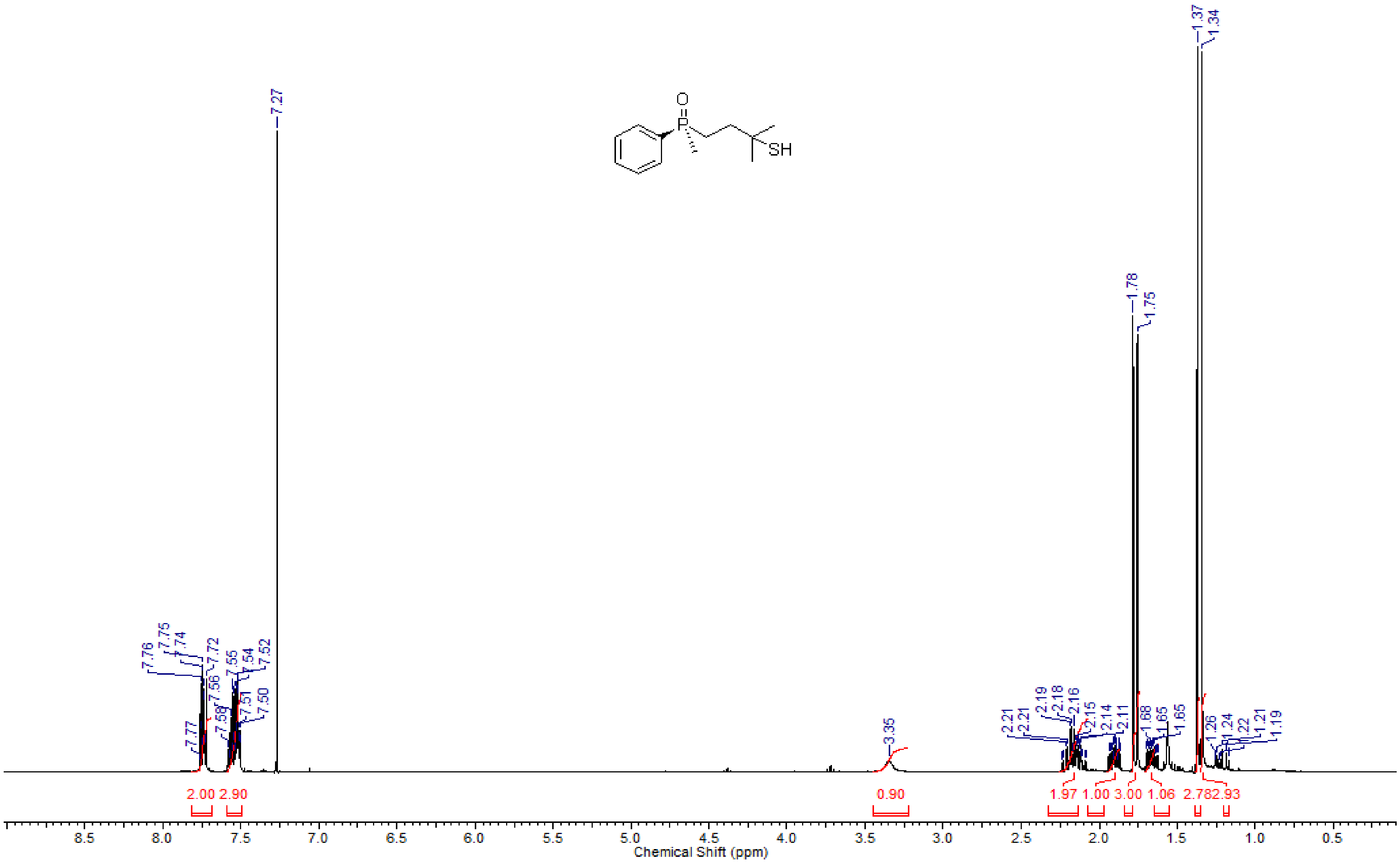

^{1}H NMR spectrum of (R_P)-(3-methyl-3-mercaptobutyl)methylphenylphosphine oxide (R_P)-(**45**) (CDCl3, 500 MHz).

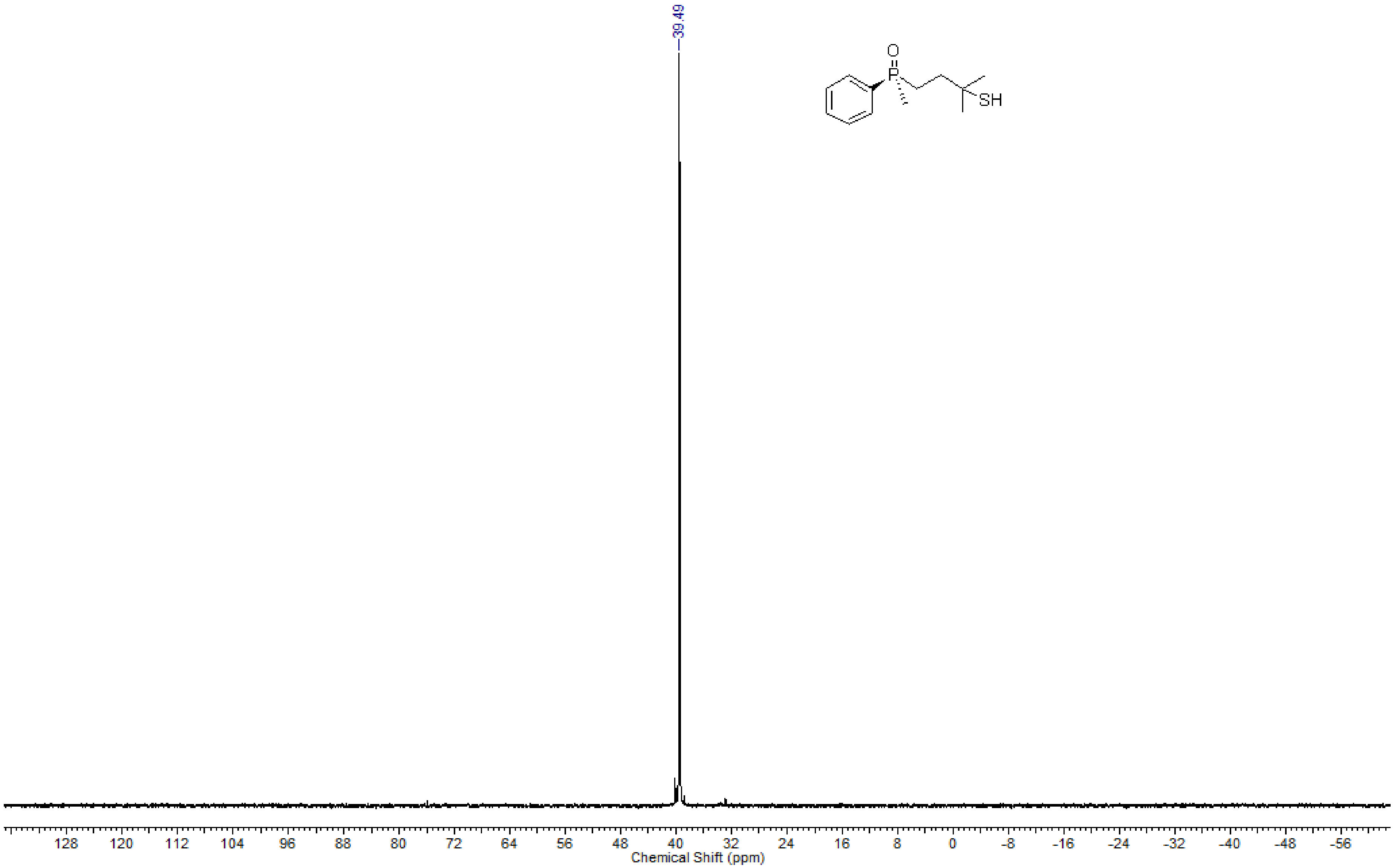

^{31}P NMR spectrum of (R_P)-(3-methyl-3-mercaptobutyl)methylphenylphosphine oxide (R_P)-(**45**) (CDCl3, 202 MHz).

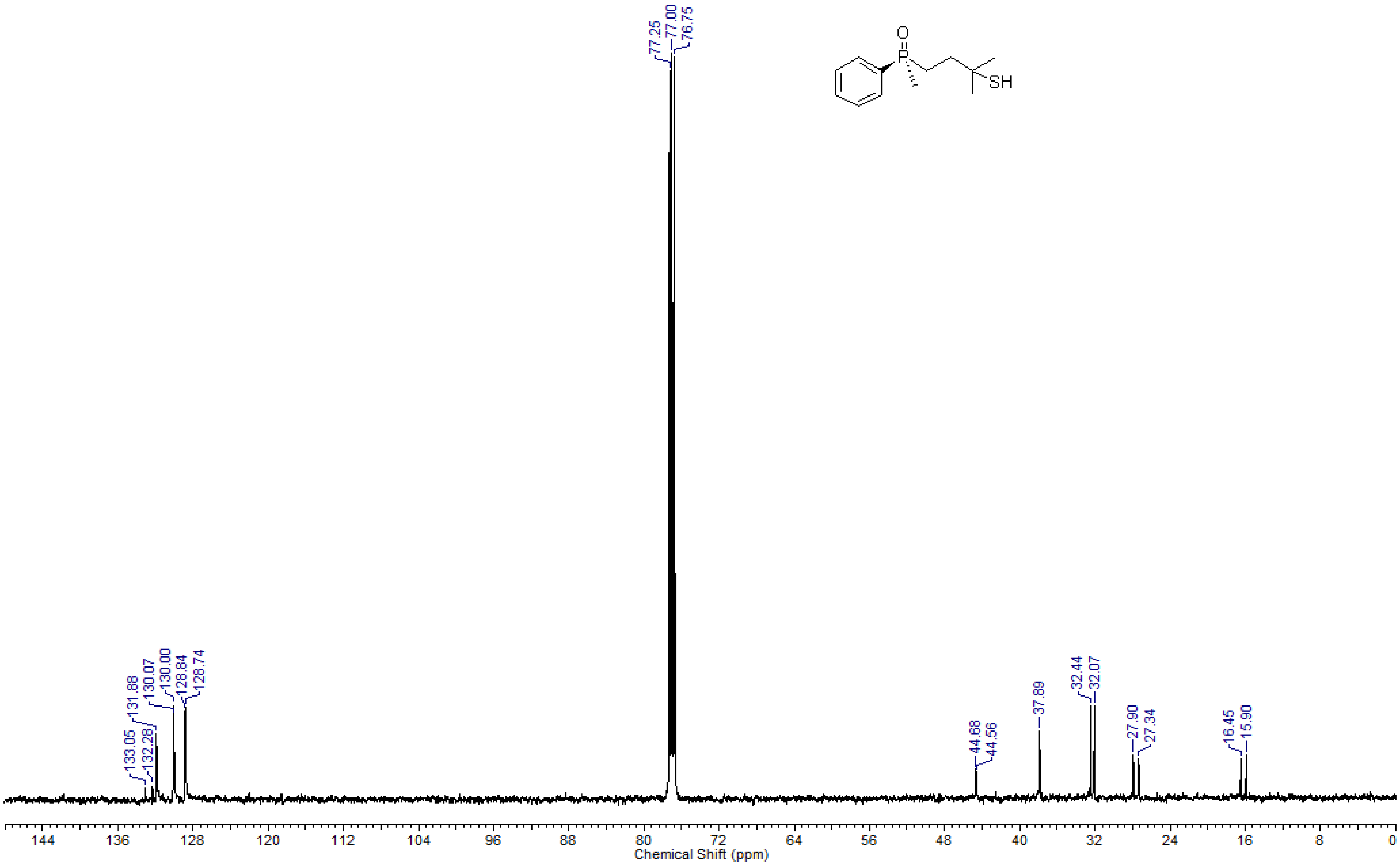

^{13}C NMR spectrum of (*R*P)-(3-methyl-3-mercaptobutyl)methylphenylphosphine oxide (*R*P)-(**45**) (CDCl3, 126 MHz).

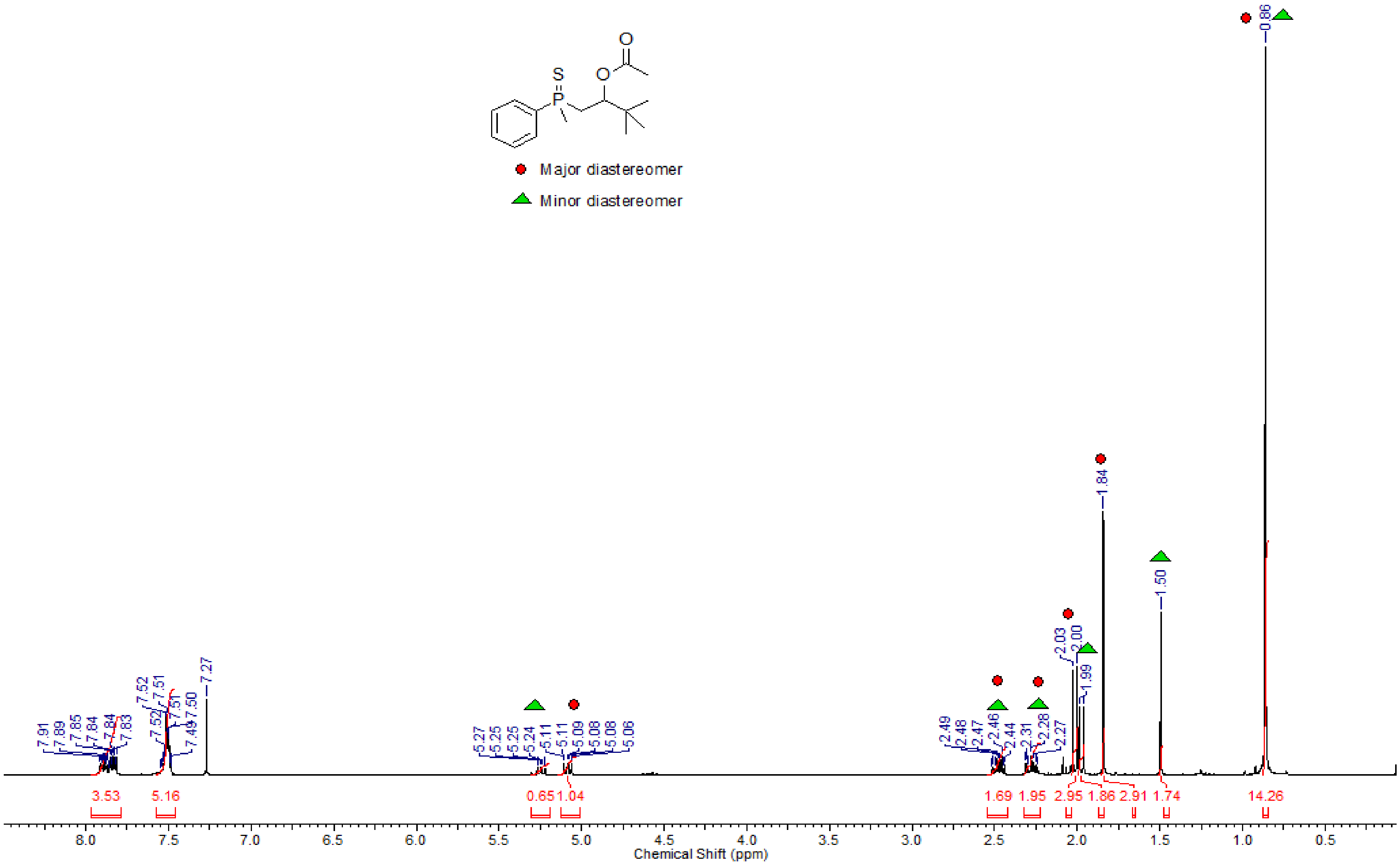

^{1}H NMR spectrum of (2-acetoxy-3,3-dimethylbutyl)methylphenylphosphine sulfide (**46**) (CDCl3, 500 MHz).

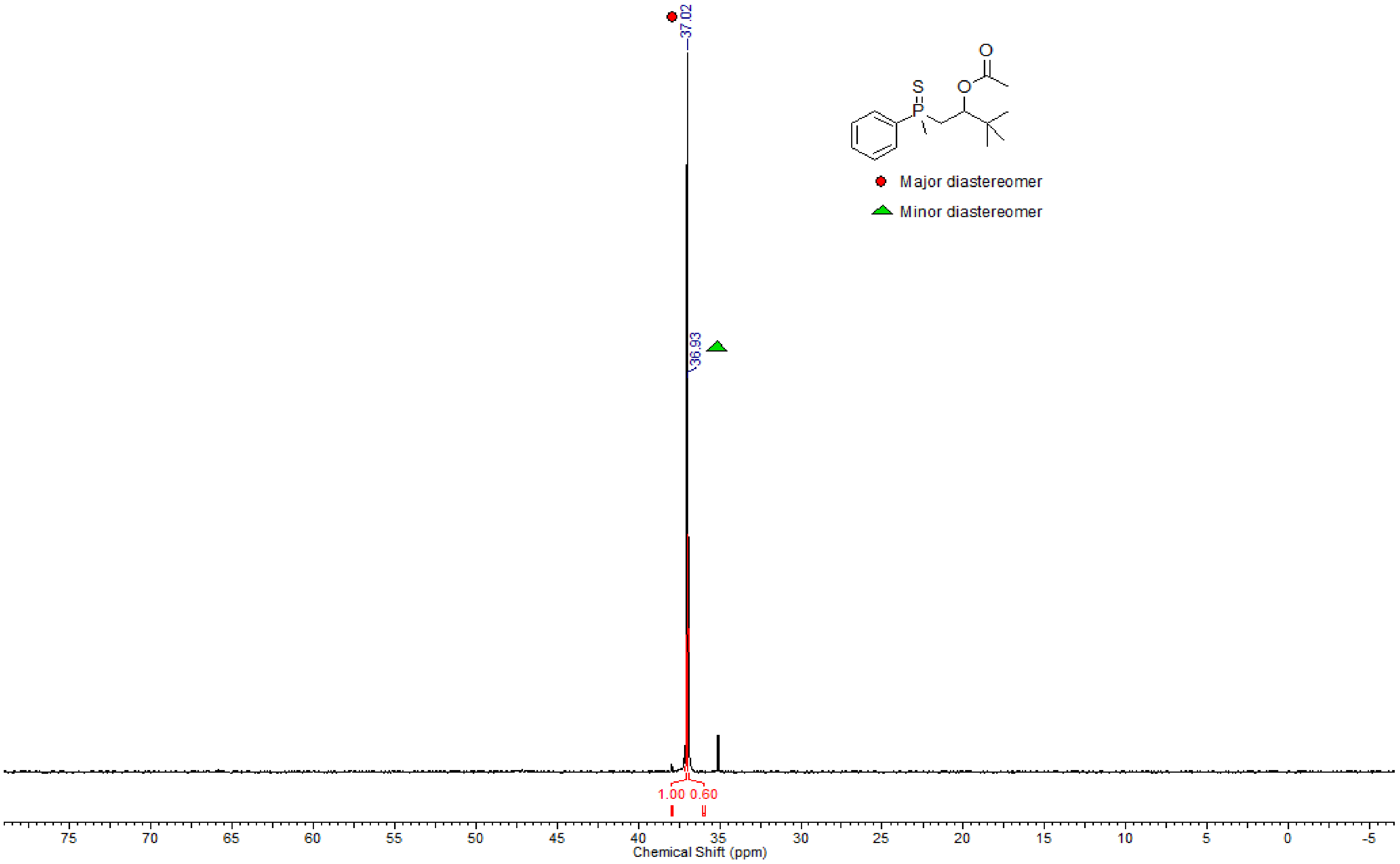

^{31}P NMR spectrum of (2-acetoxy-3,3-dimethylbutyl)methylphenylphosphine sulfide (**46**) (CDCl3, 202 MHz).

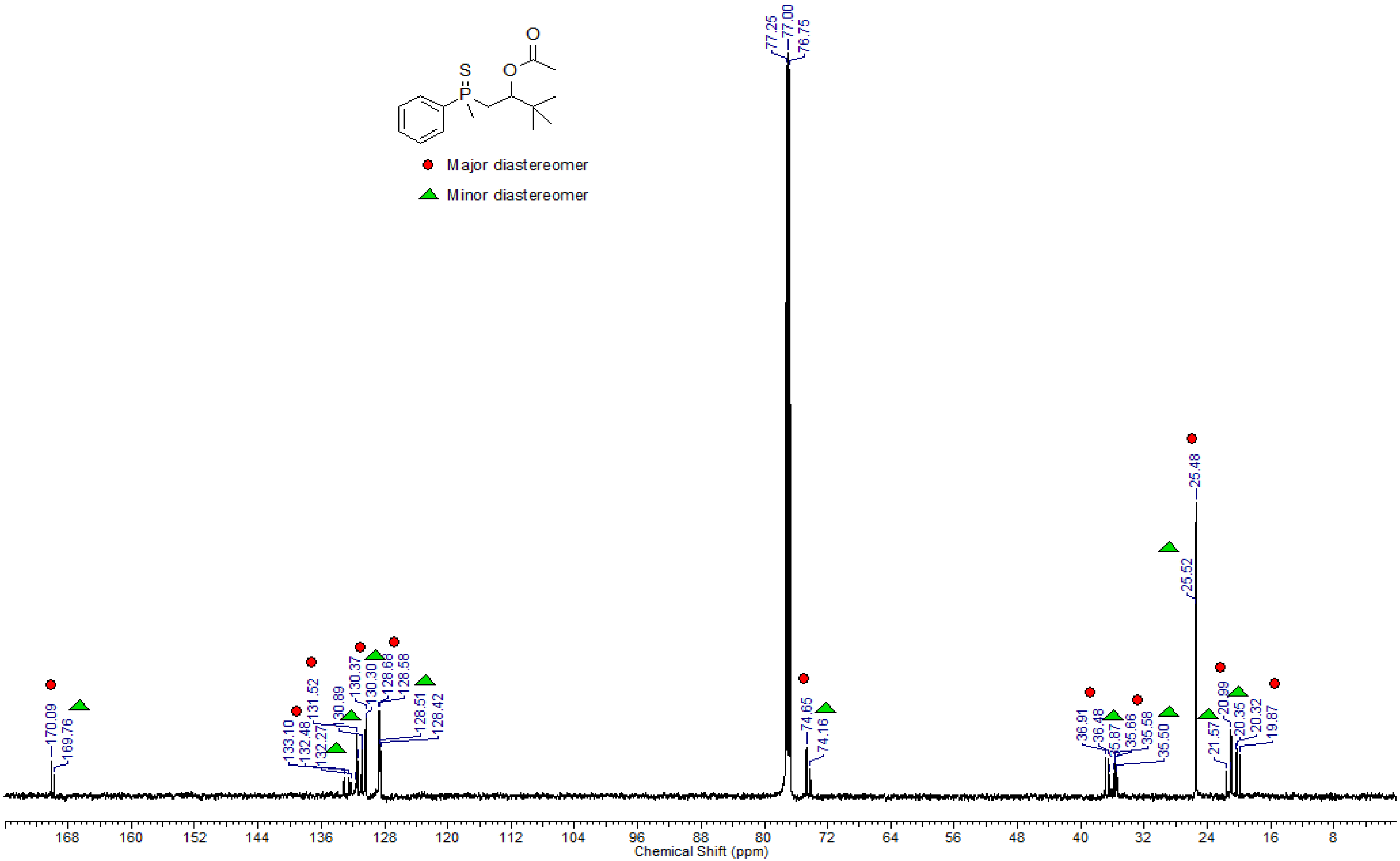

^{13}C NMR spectrum of (2-acetoxy-3,3-dimethylbutyl)methylphenylphosphine sulfide (**46**) (CDCl3, 126 MHz).

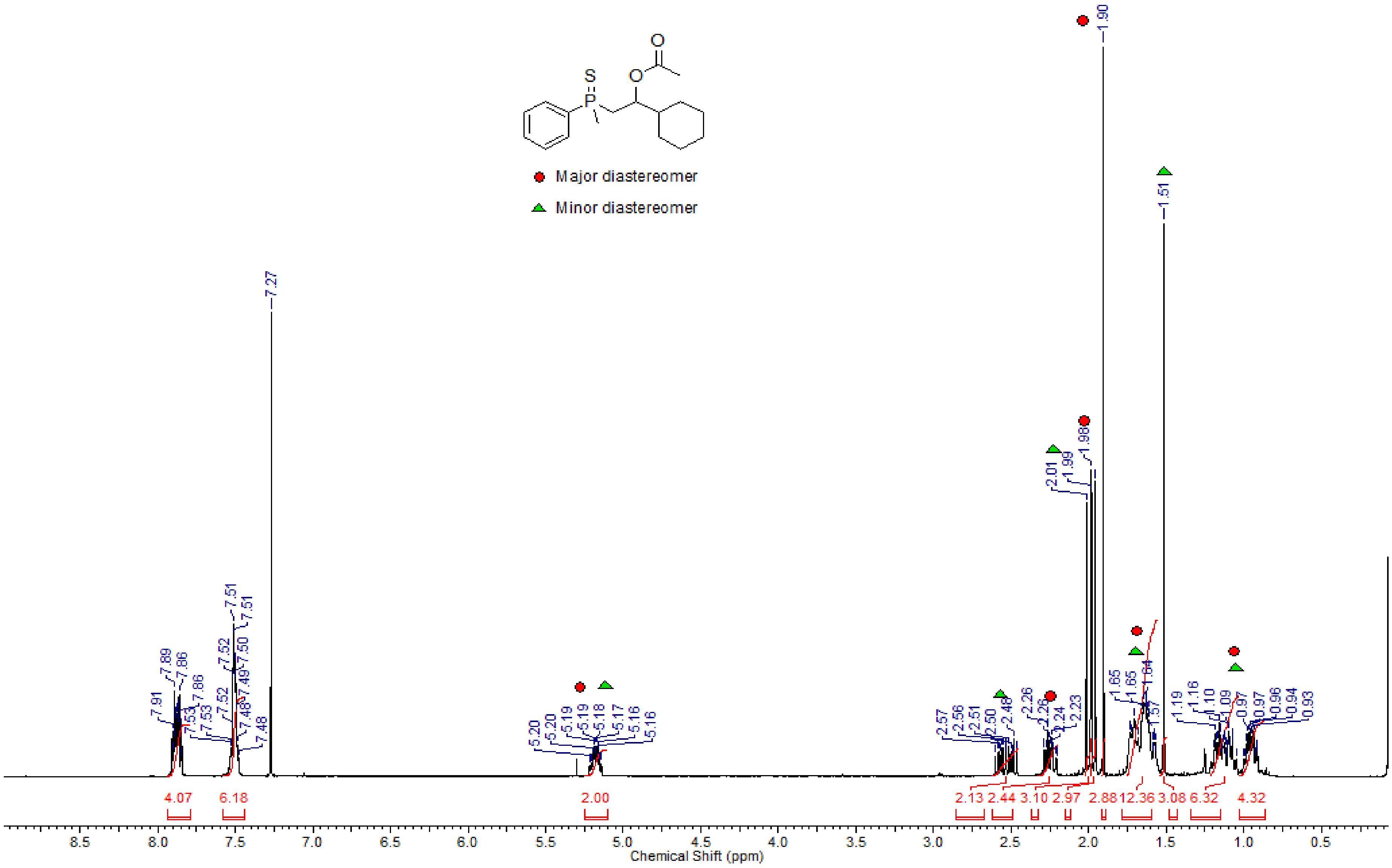

^{1}H NMR spectrum of (2-acetoxy-2-cyclohexylethyl)methylphenylphosphine sulfide (**47**) (CDCl3, 500 MHz).

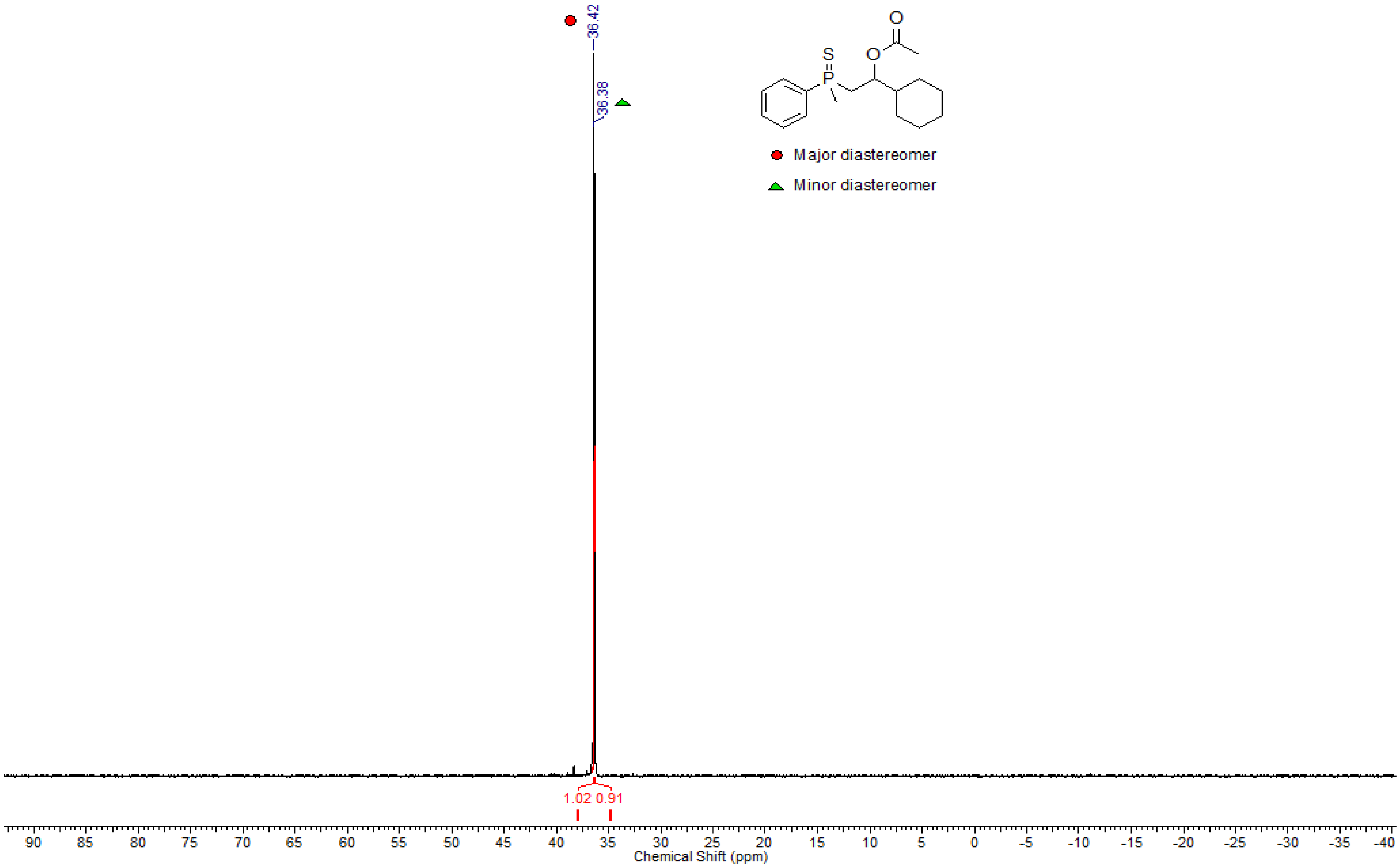

^{31}P NMR spectrum of (2-acetoxy-2-cyclohexylethyl)methylphenylphosphine sulfide (**47**) (CDCl3, 202 MHz).

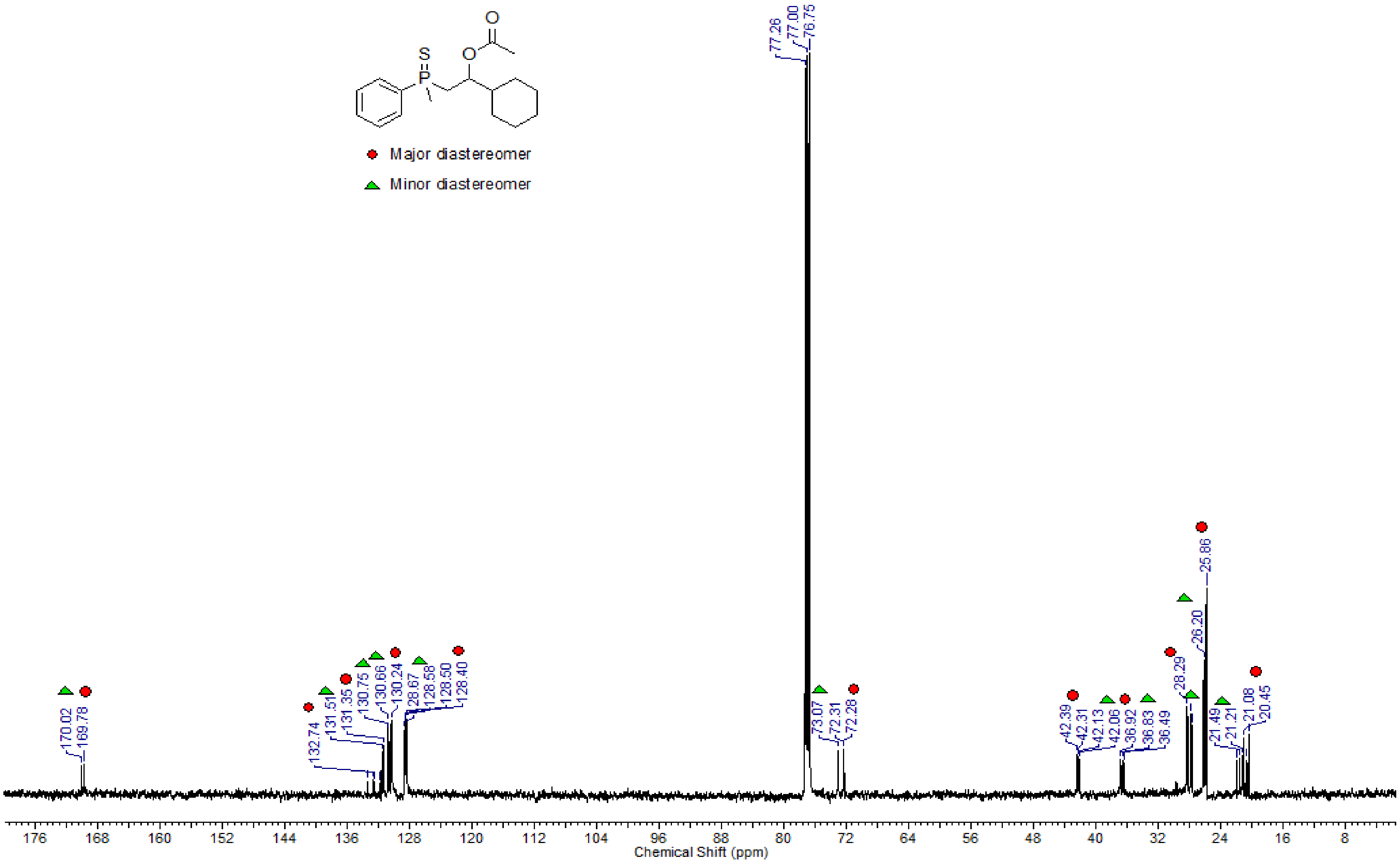

^{13}C NMR spectrum of (2-acetoxy-2-cyclohexylethyl)methylphenylphosphine sulfide (**47**) (CDCl3, 126 MHz).

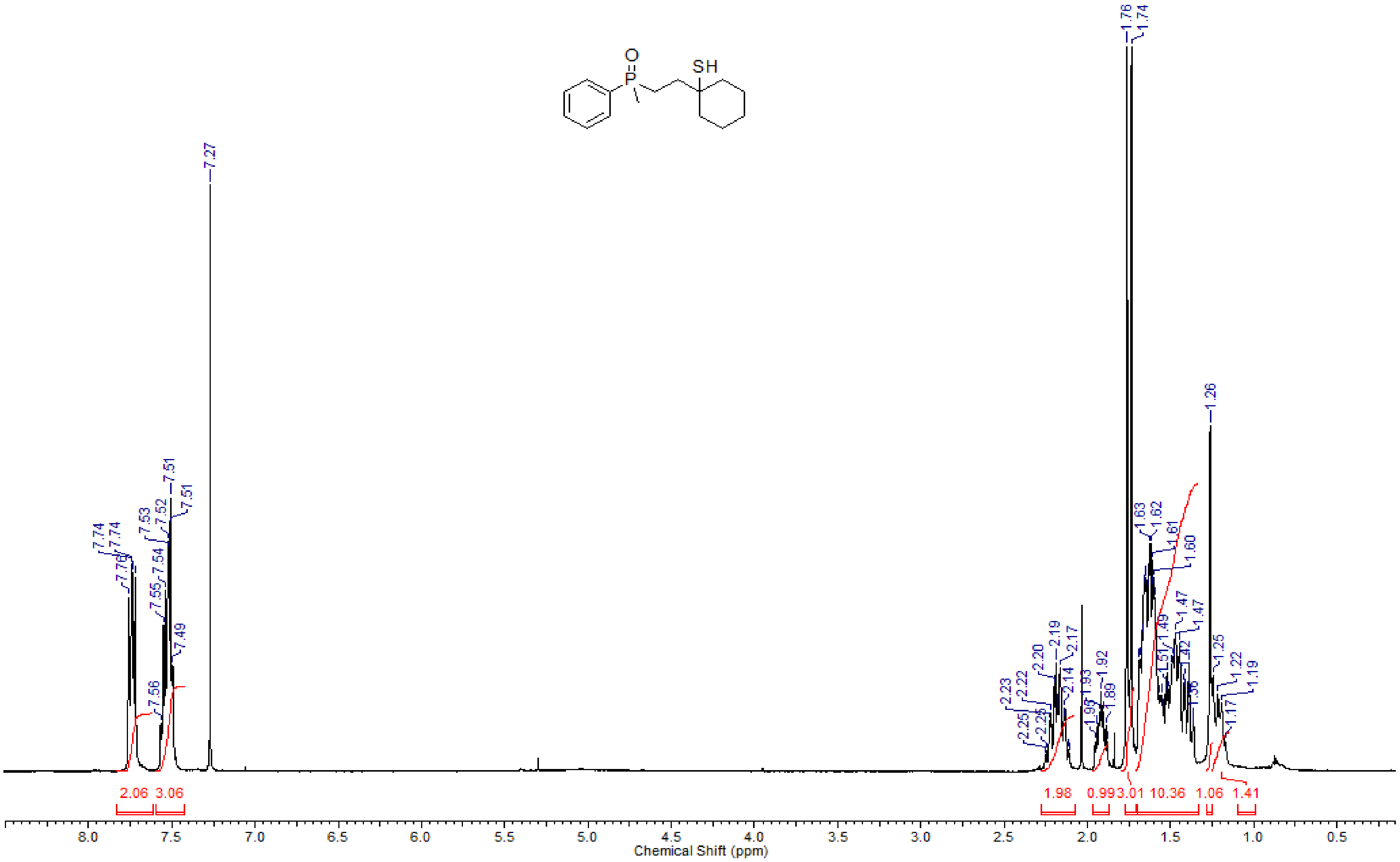

^{1}H NMR spectrum of [(1-mercapto)cyclohexylethyl)]methylphenylphosphine sulfide (**48**) (CDCl3, 500 MHz).

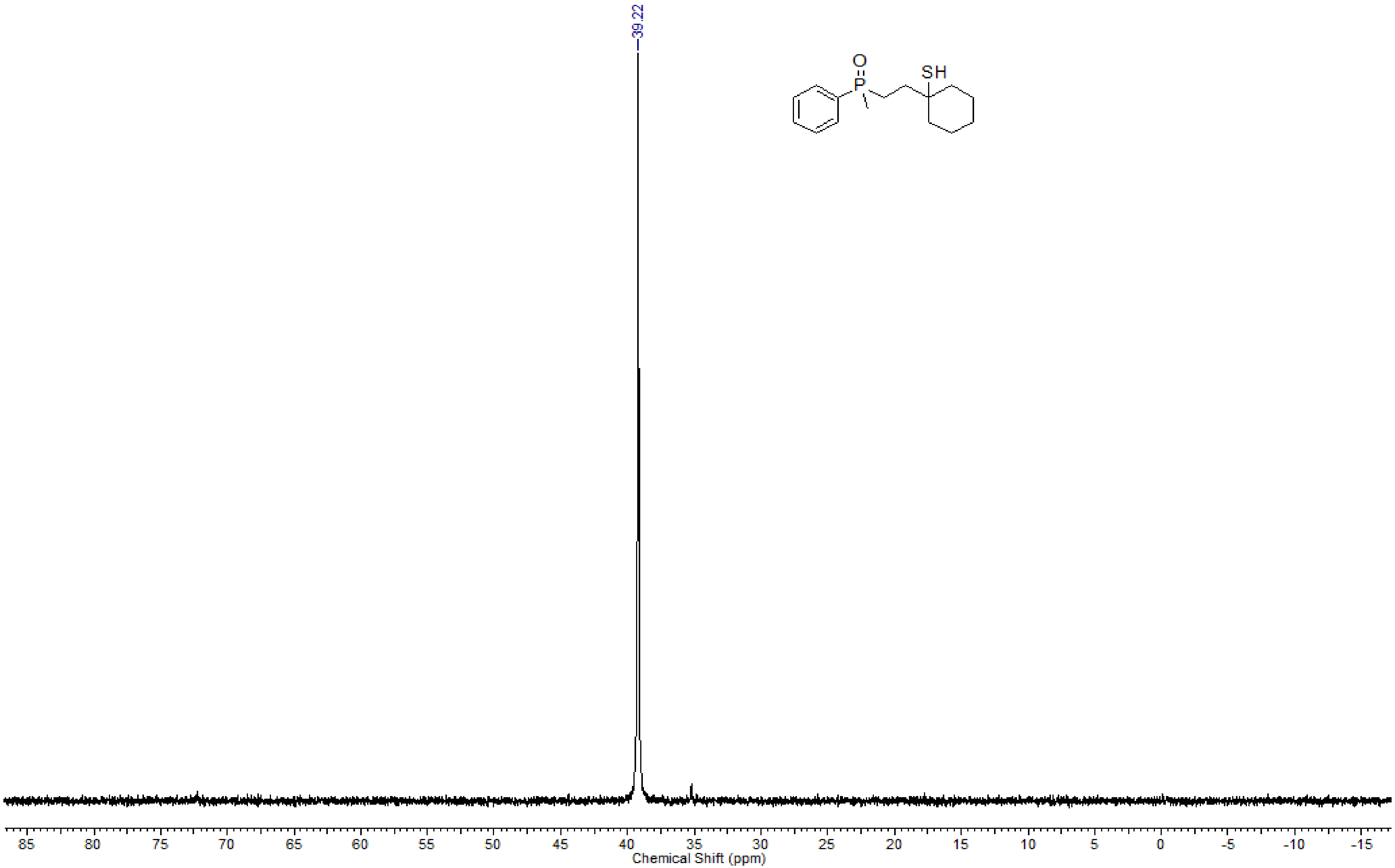

^{31}P NMR spectrum of [(1-mercapto)cyclohexylethyl)]methylphenylphosphine sulfide (**48**) (CDCl3, 202 MHz).

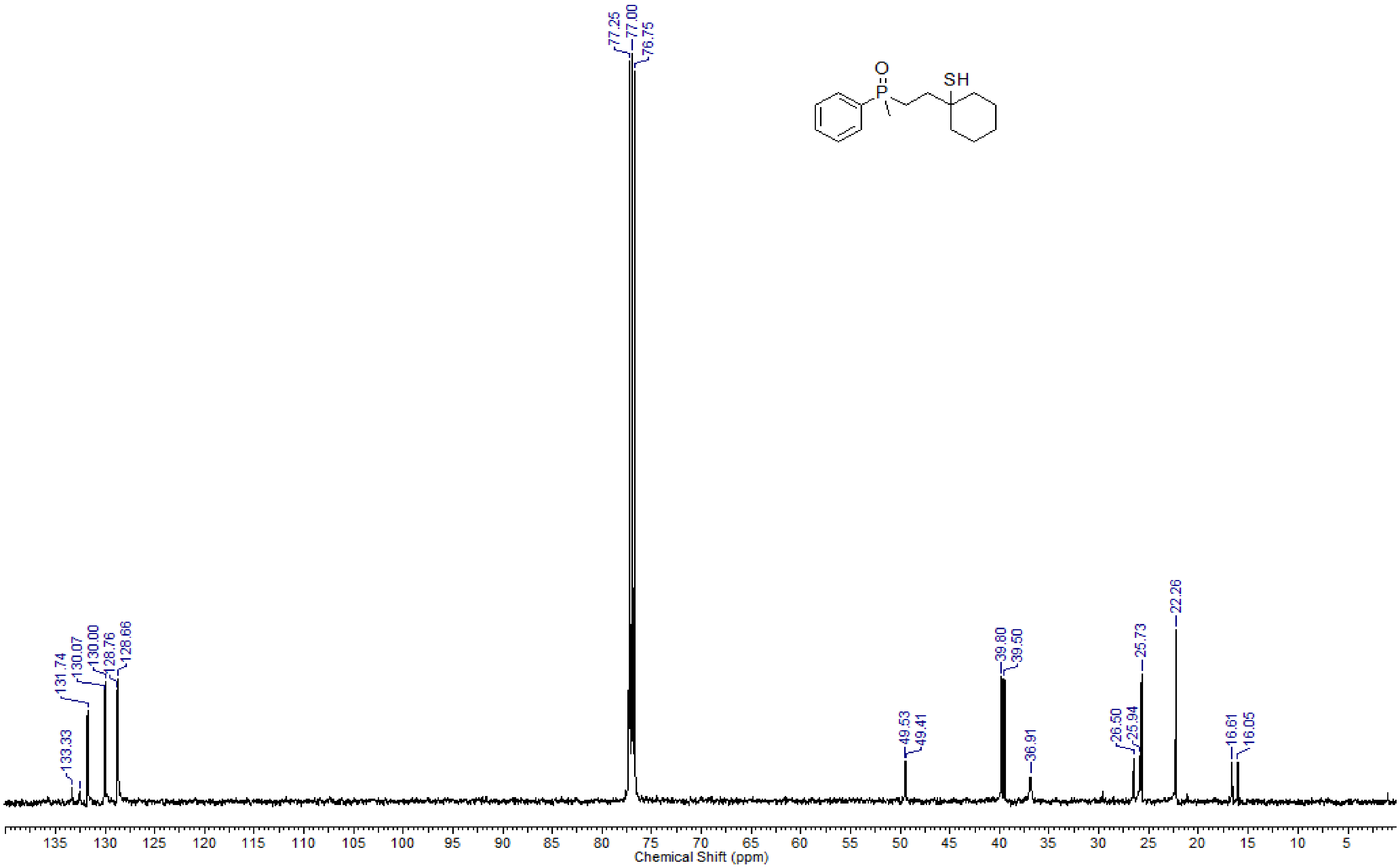

^{13}C NMR spectrum of [(1-mercapto)cyclohexylethyl)]methylphenylphosphine sulfide (**48**) (CDCl3, 126 MHz).

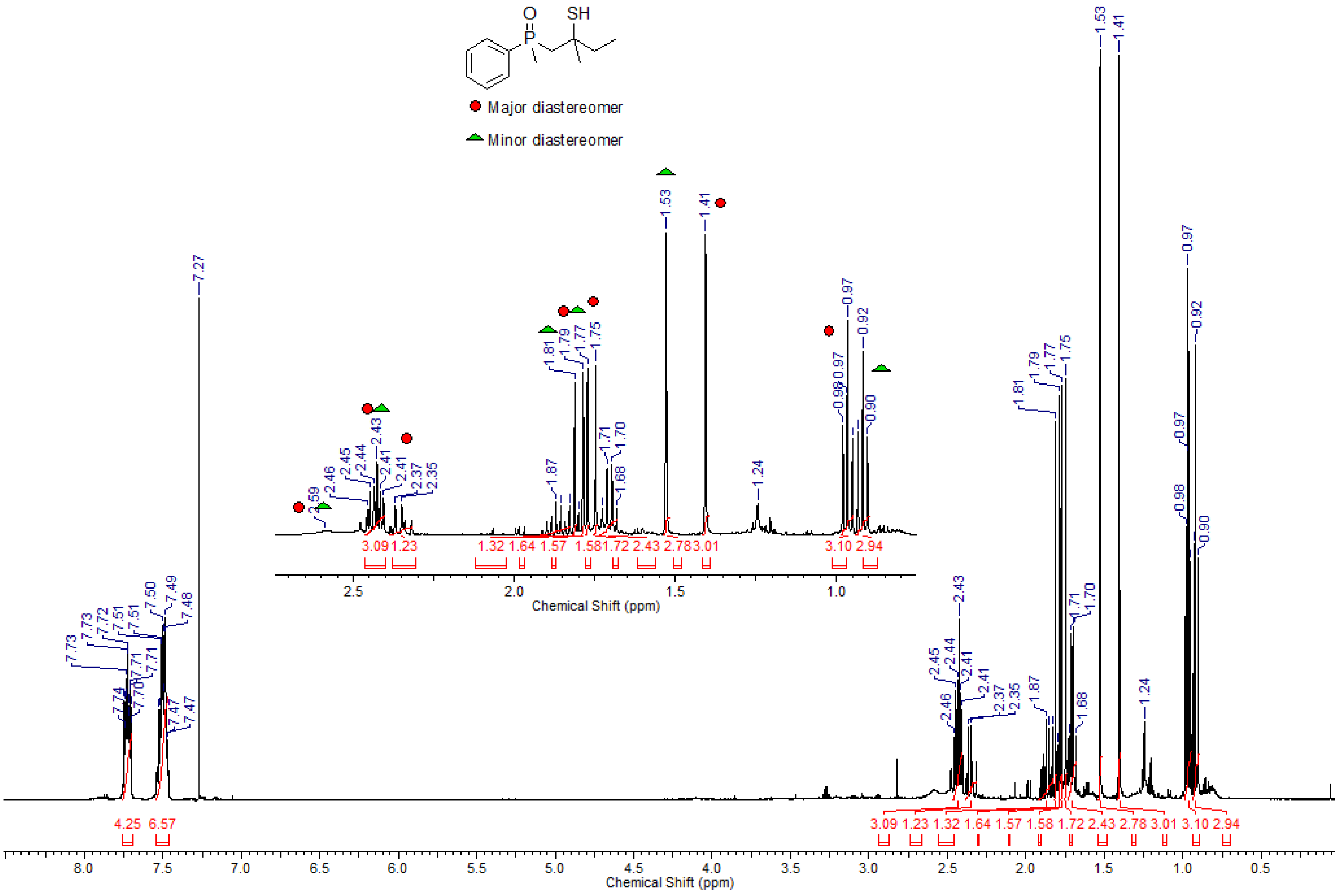

^{1}H NMR spectrum of (2-methyl-2-mercaptobutyl)methylphenylphosphine oxide (**49**) (CDCl3, 500 MHz).

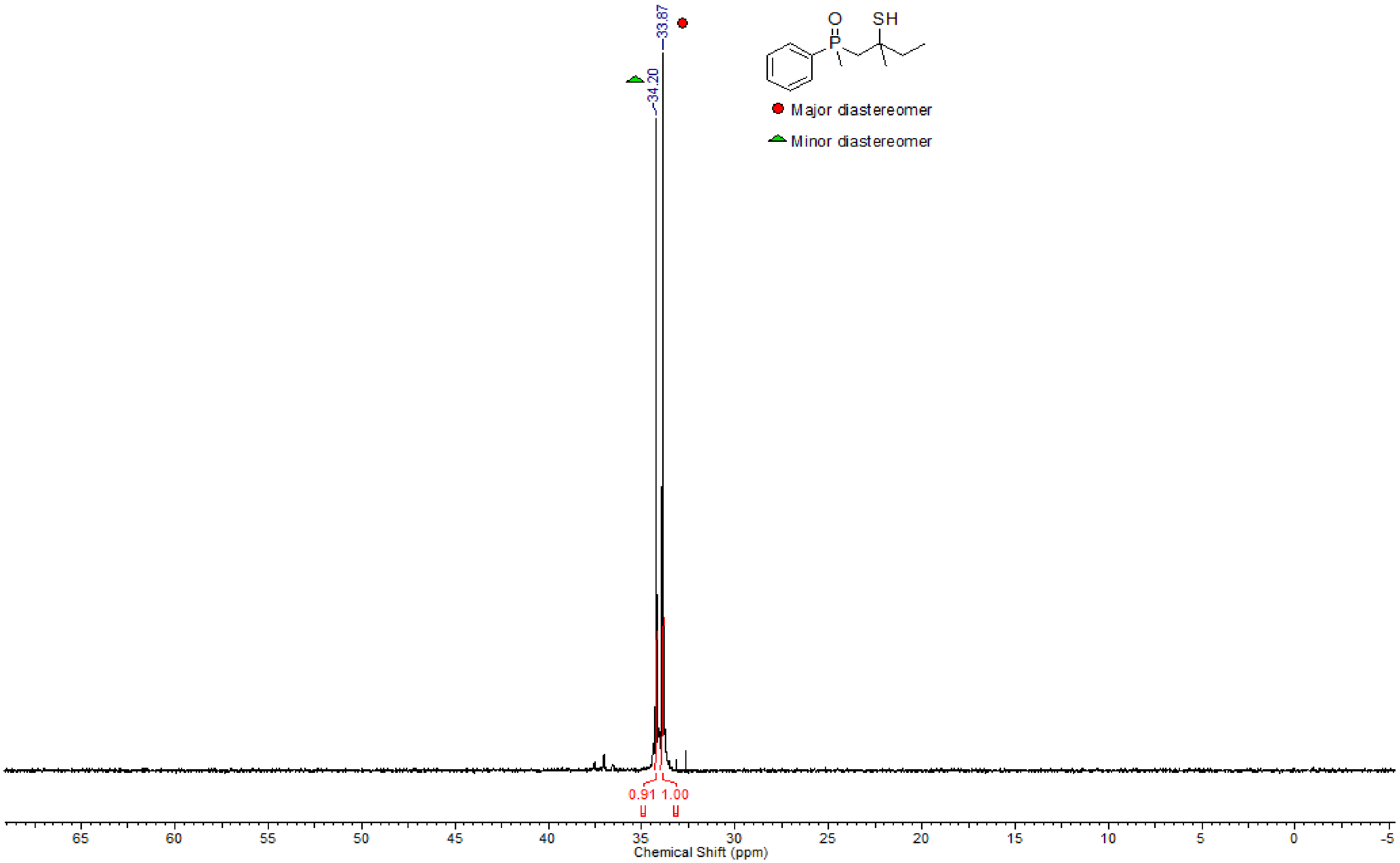

^{31}P NMR spectrum of (2-methyl-2-mercaptobutyl)methylphenylphosphine oxide (**49**) (CDCl3, 202 MHz).

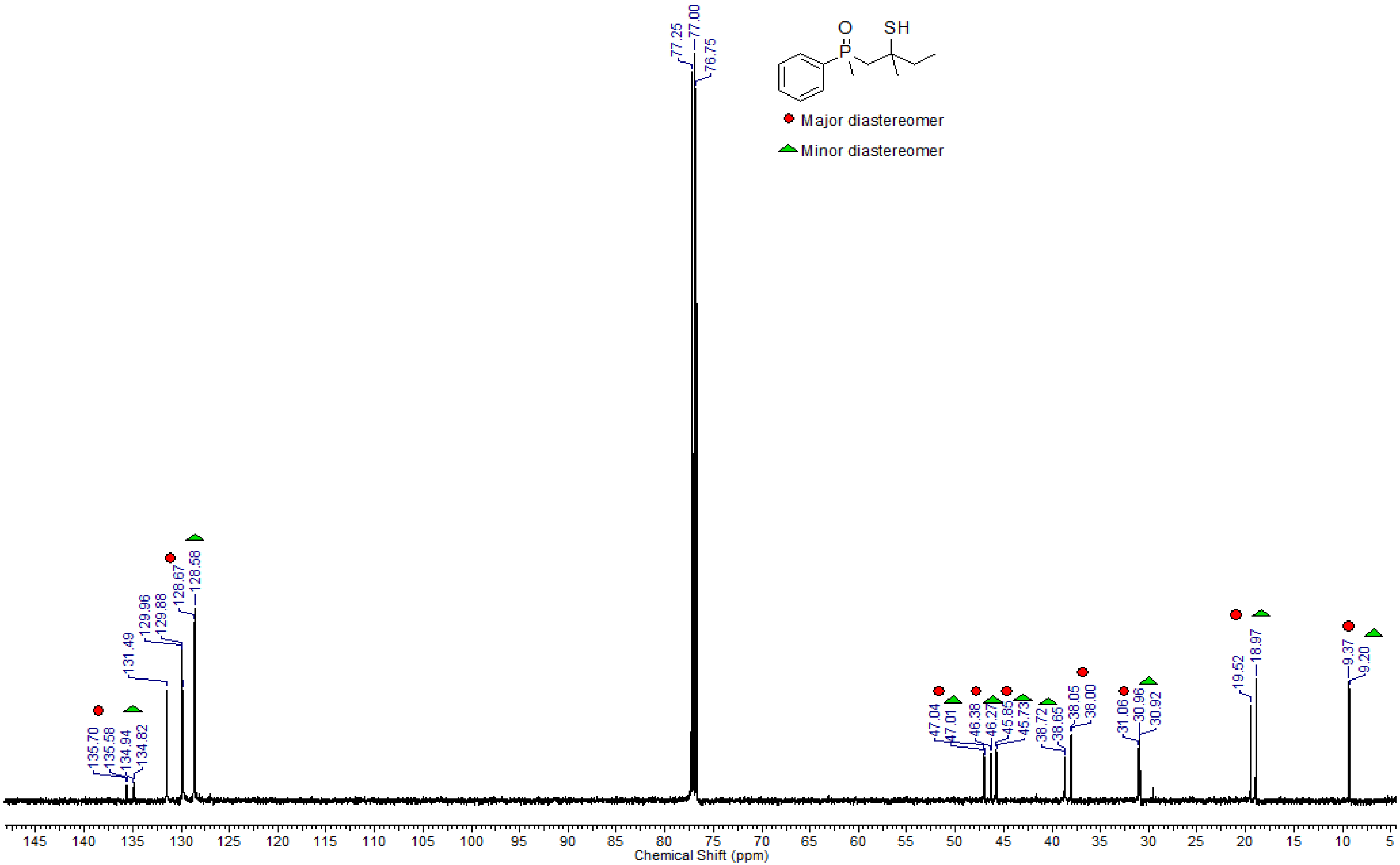

^{13}C NMR spectrum of (2-methyl-2-mercaptobutyl)methylphenylphosphine oxide (**49**) (CDCl3, 126 MHz).

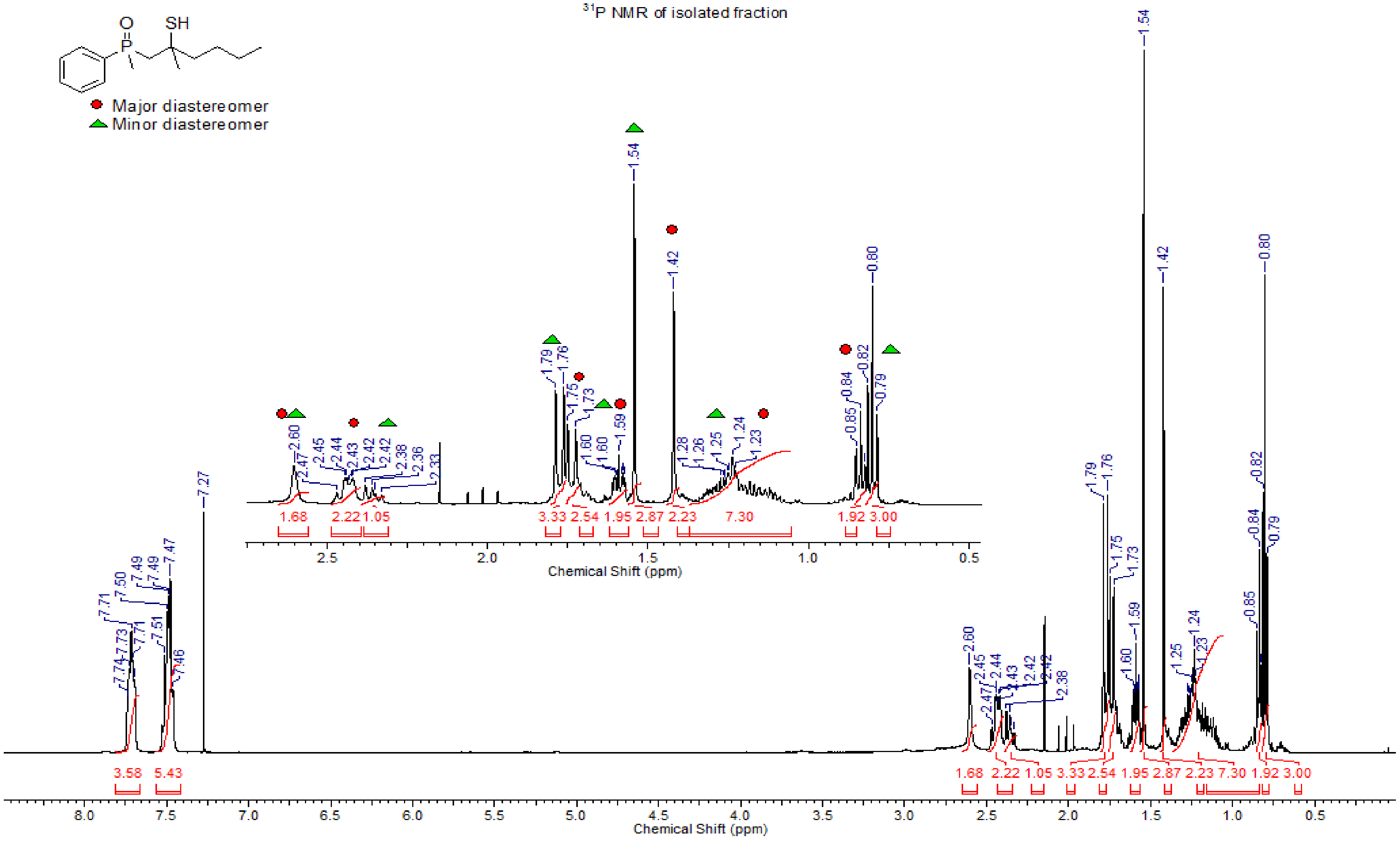

^{1}H NMR spectrum of (2-methyl-2-mercaptohexyl)methylphenylphosphine oxide (**50**) (CDCl3, 500 MHz).

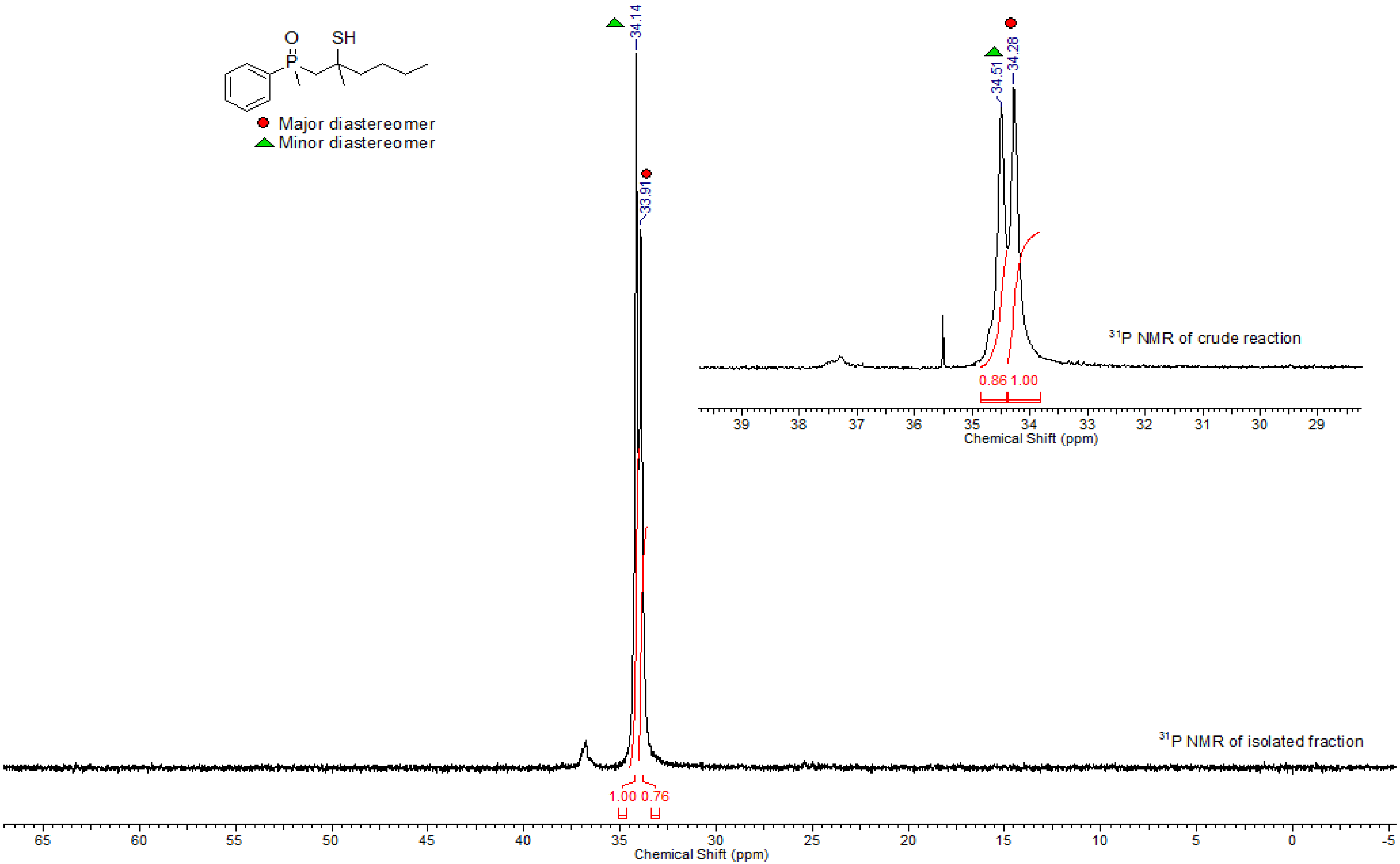

^{31}P NMR spectrum of (2-methyl-2-mercaptohexyl)methylphenylphosphine oxide (**50**) (CDCl3, 202 MHz).

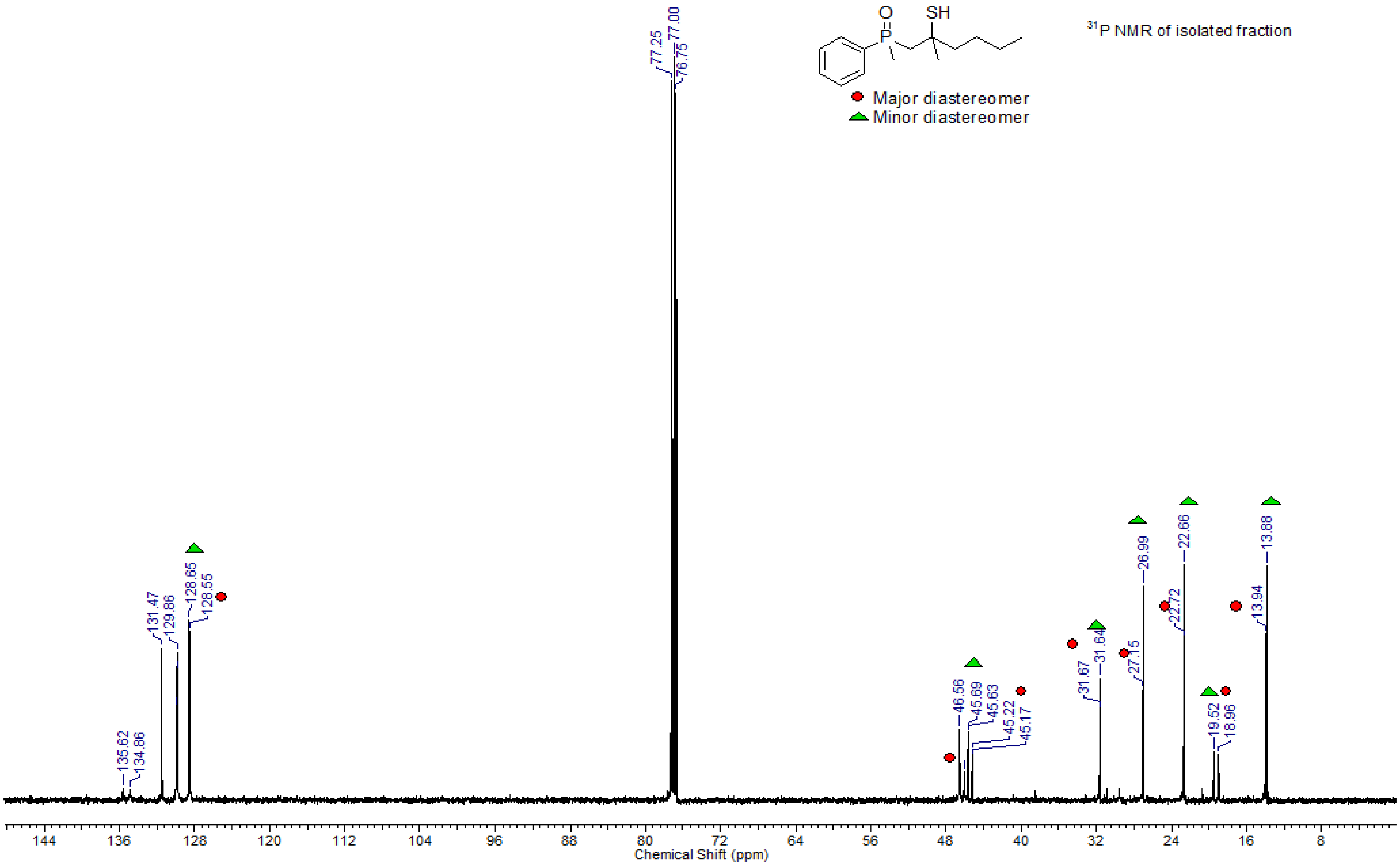

^{13}C NMR spectrum of (2-methyl-2-mercaptohexyl)methylphenylphosphine oxide (**50**) (CDCl3, 126 MHz).

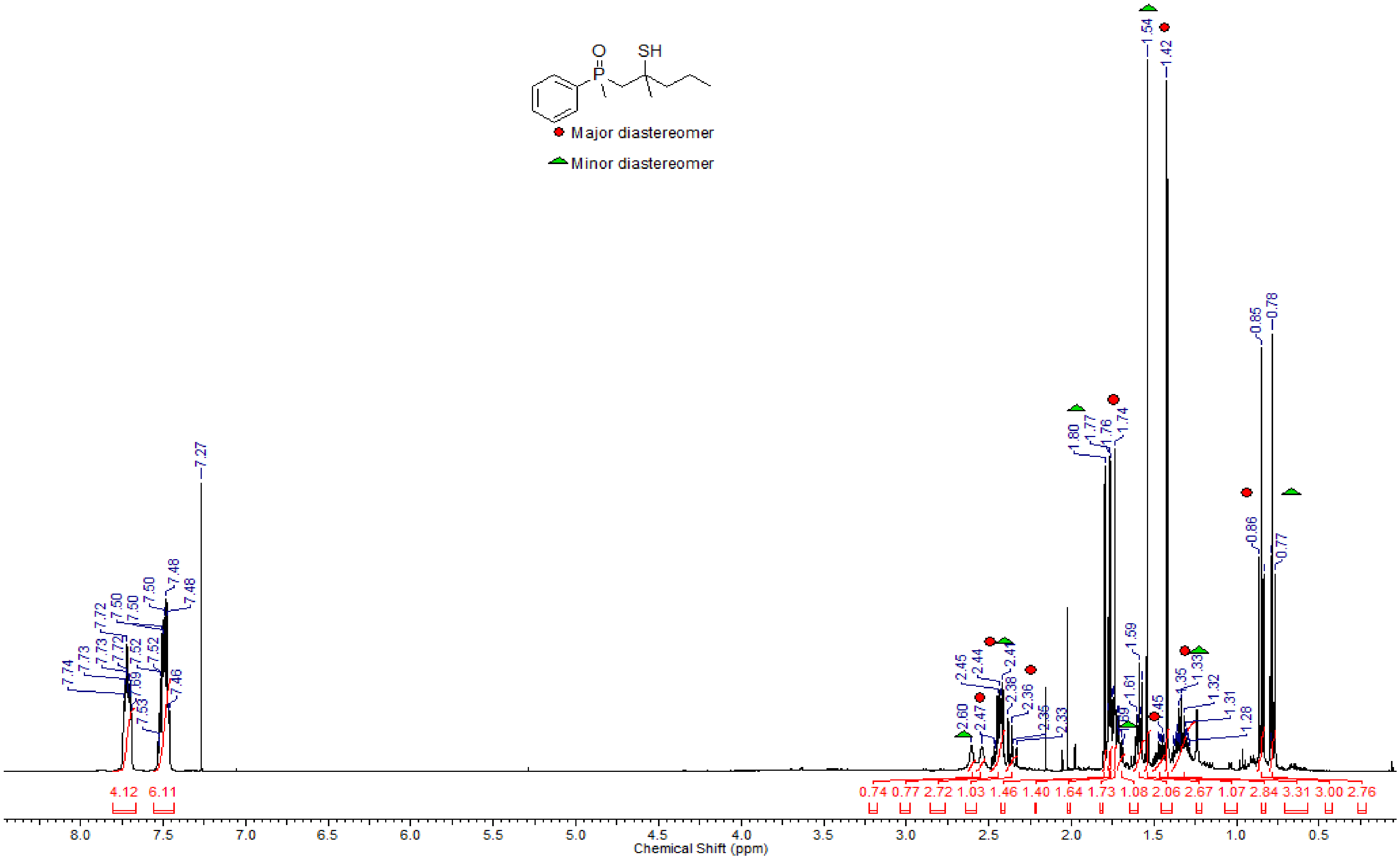

^{1}H NMR spectrum of (2-methyl-2-mercaptopentyl)methylphenylphosphine oxide (**51**) (CDCl3, 500 MHz).

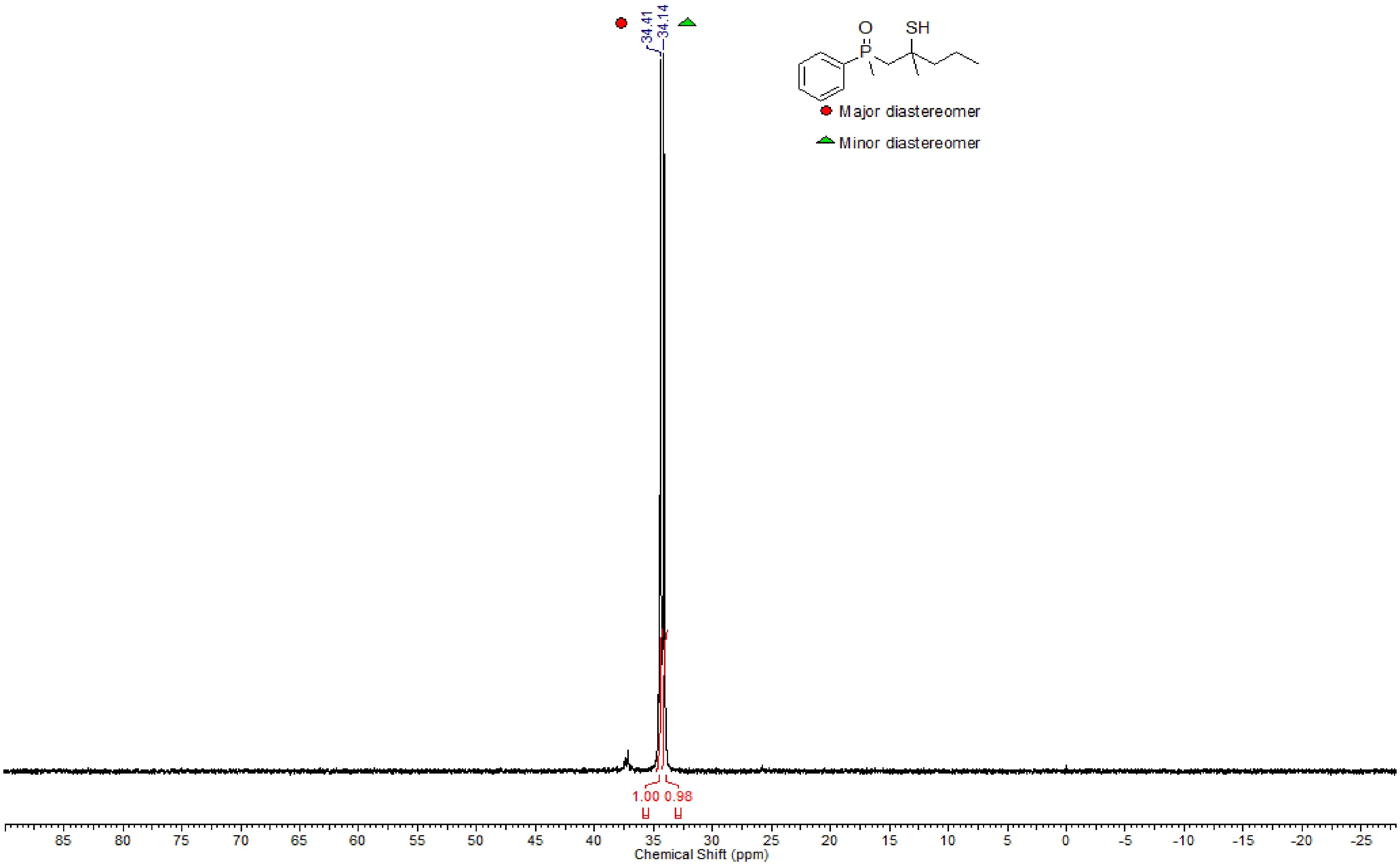

^{31}P NMR spectrum of (2-methyl-2-mercaptopentyl)methylphenylphosphine oxide (**51**) (CDCl3, 202 MHz).

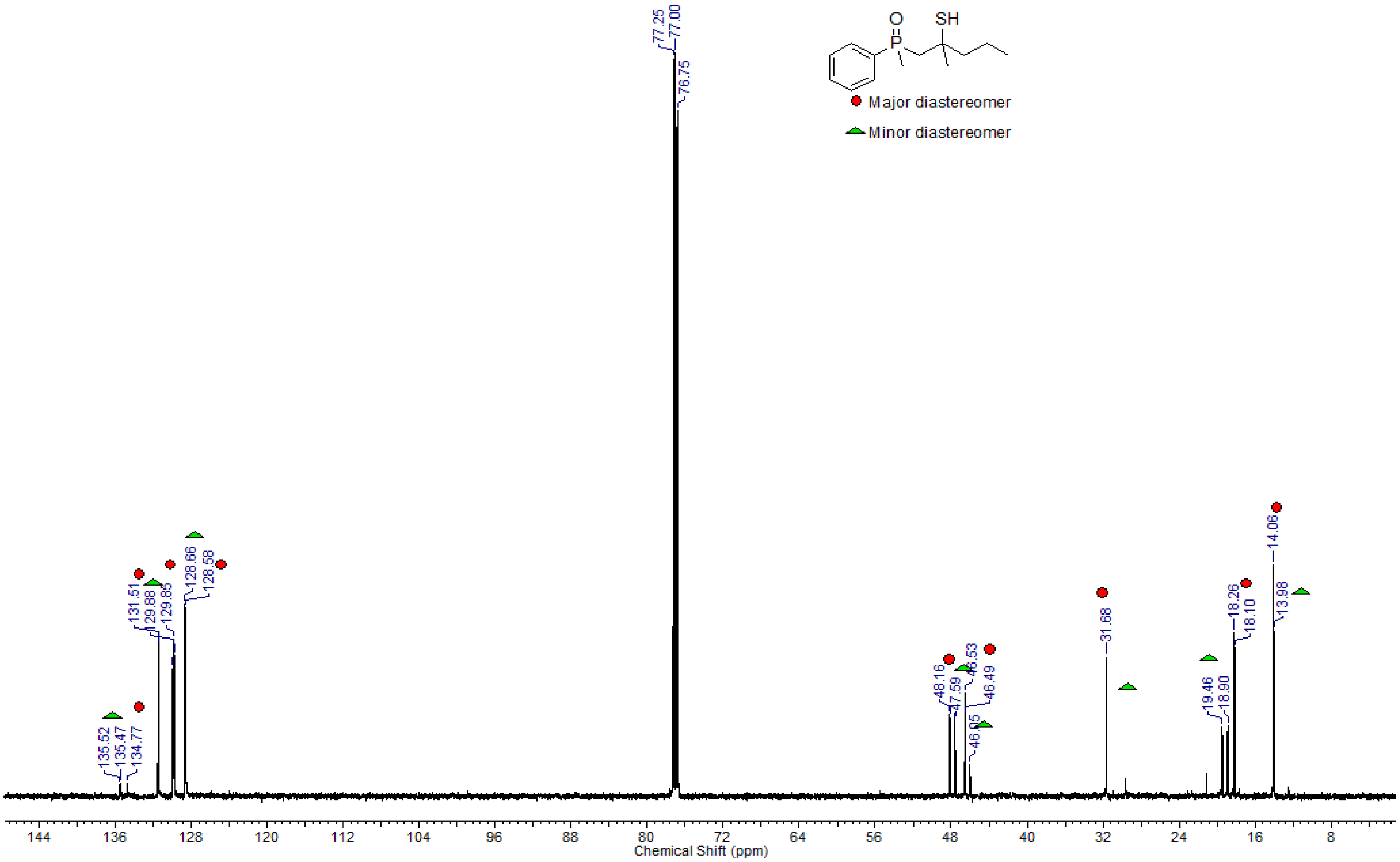

^{13}C NMR spectrum of (2-methyl-2-mercaptopentyl)methylphenylphosphine oxide (**51**) (CDCl3, 126 MHz).

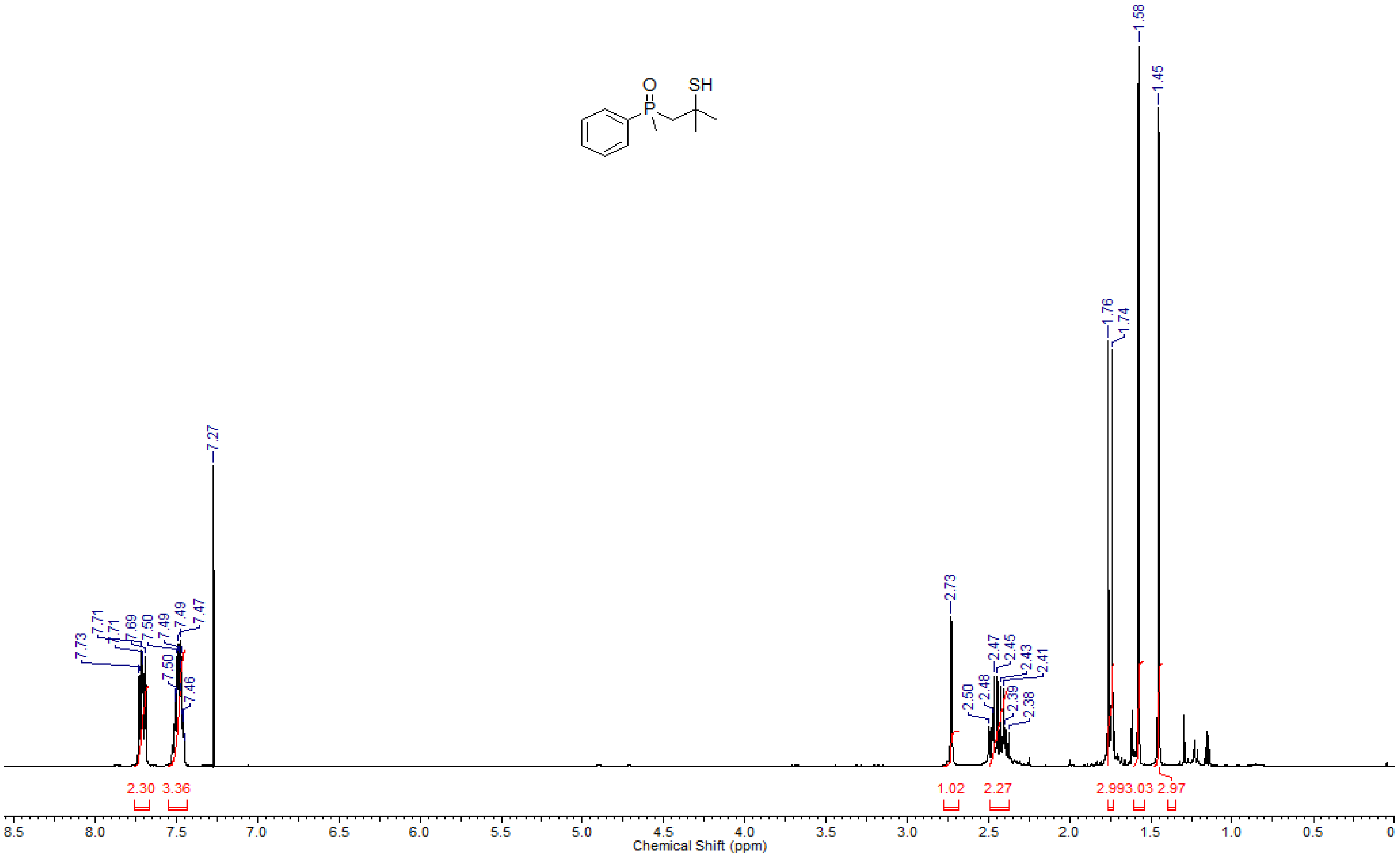

^{1}H NMR spectrum of (2-methyl-2-mercaptopropyl)methylphenylphosphine oxide (**52**) (CDCl3, 500 MHz).

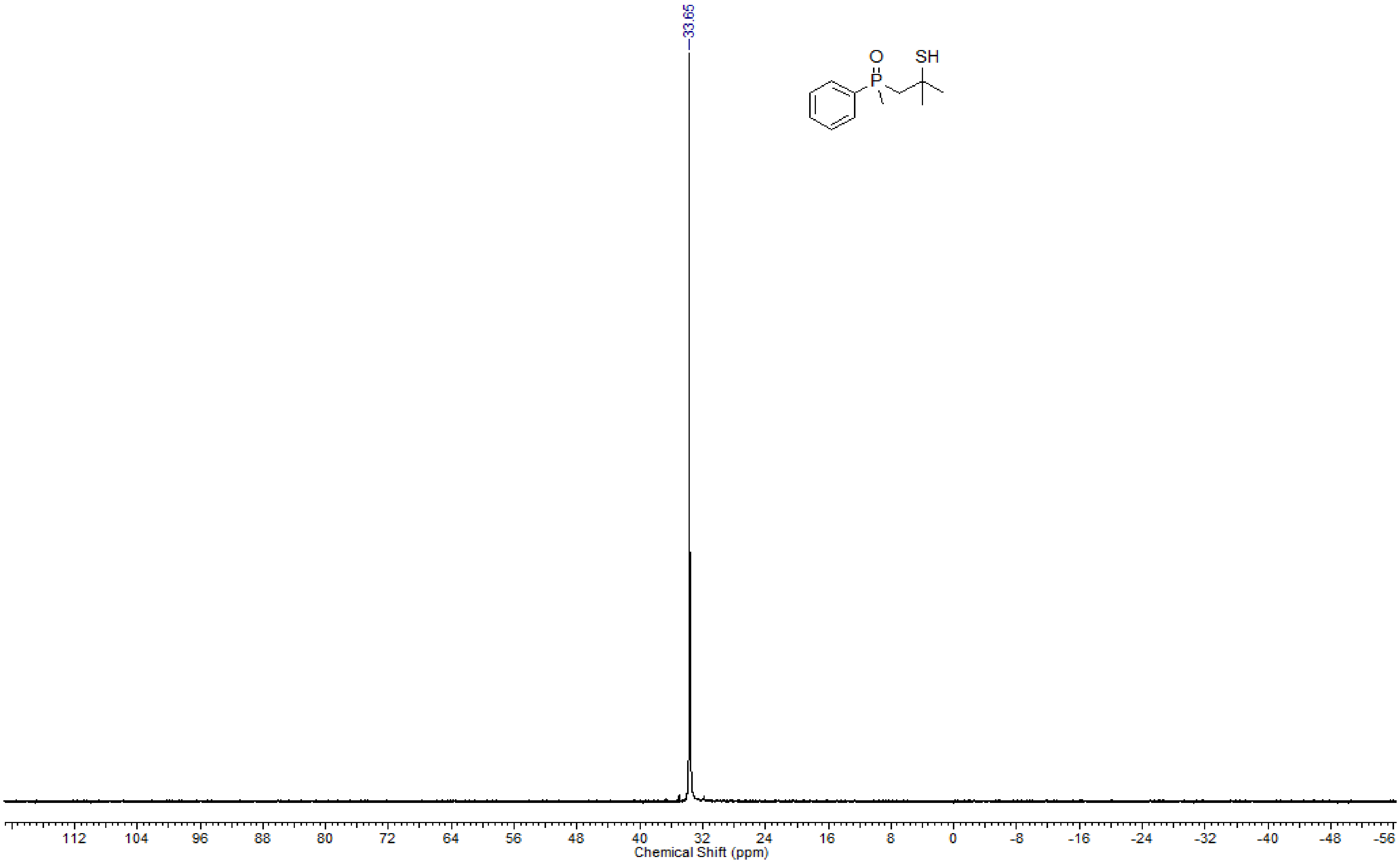

^{31}P NMR spectrum of (2-methyl-2-mercaptopropyl)methylphenylphosphine oxide (**52**) (CDCl3, 202 MHz).

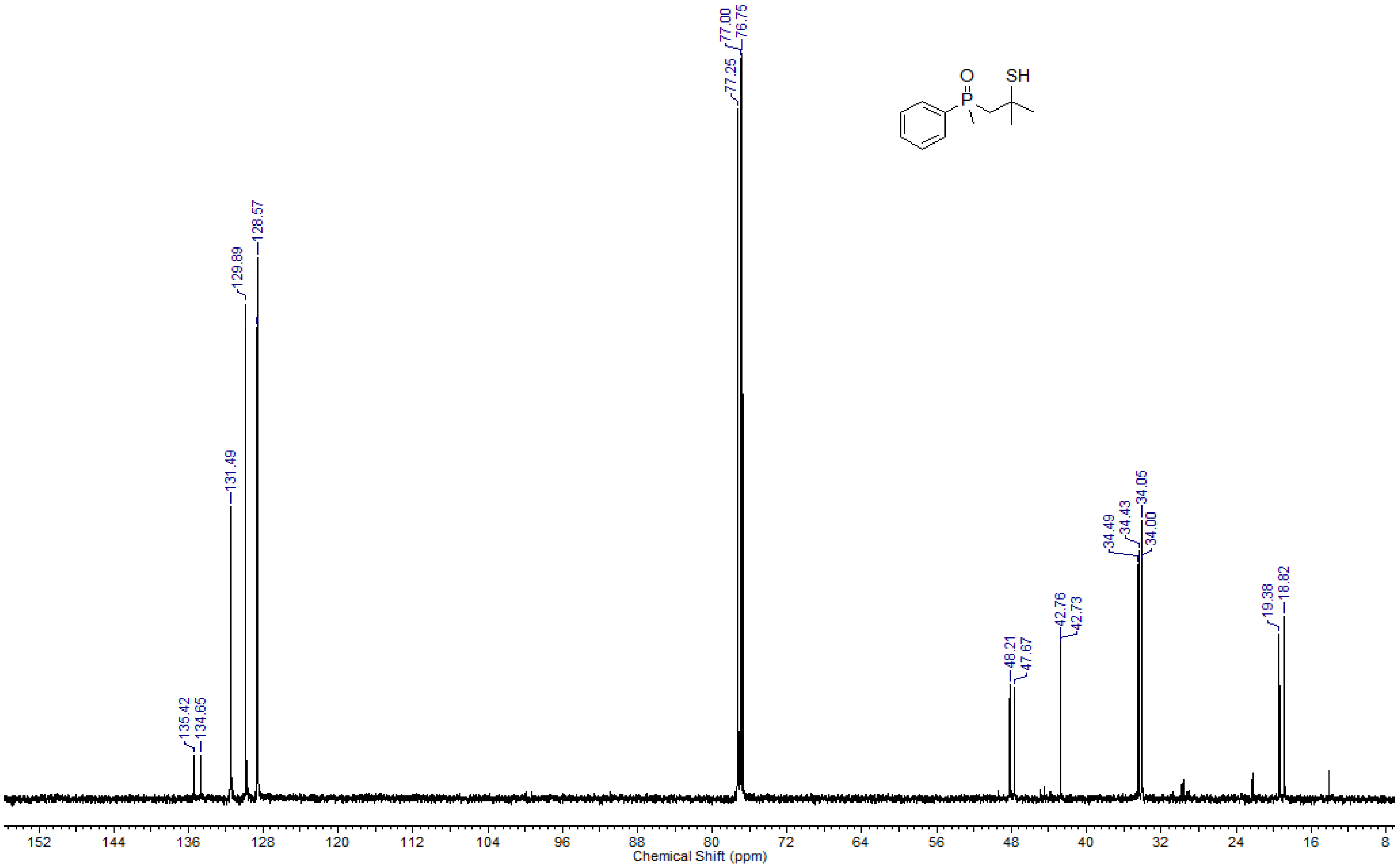

^{13}C NMR spectrum of (2-methyl-2-mercaptopropyl)methylphenylphosphine oxide (**52**) (CDCl3, 126 MHz).

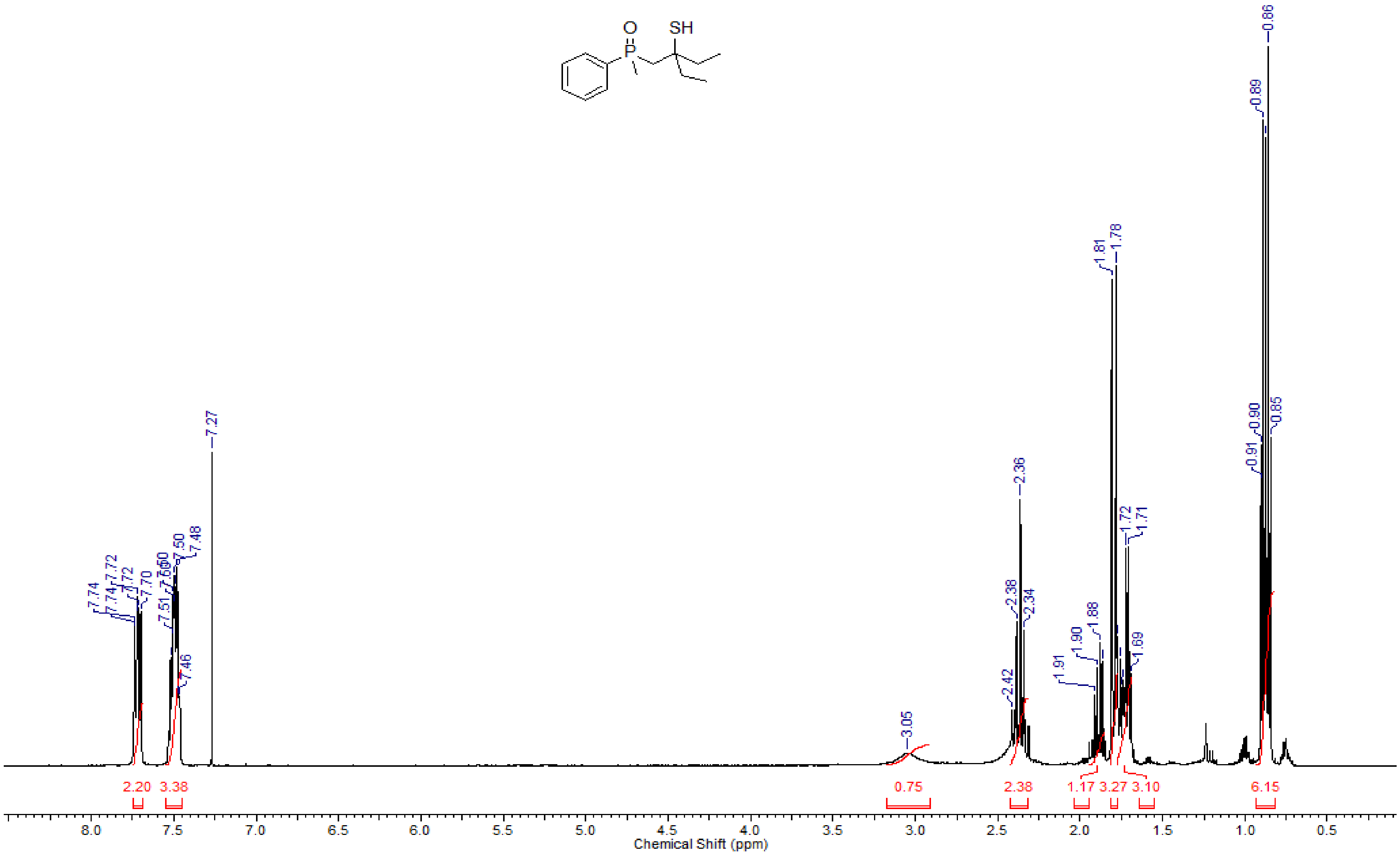

^{1}H NMR spectrum of (2-ethyl-2-mercaptobutyl)methylphenylphosphine oxide (**53**) (CDCl3, 500 MHz).

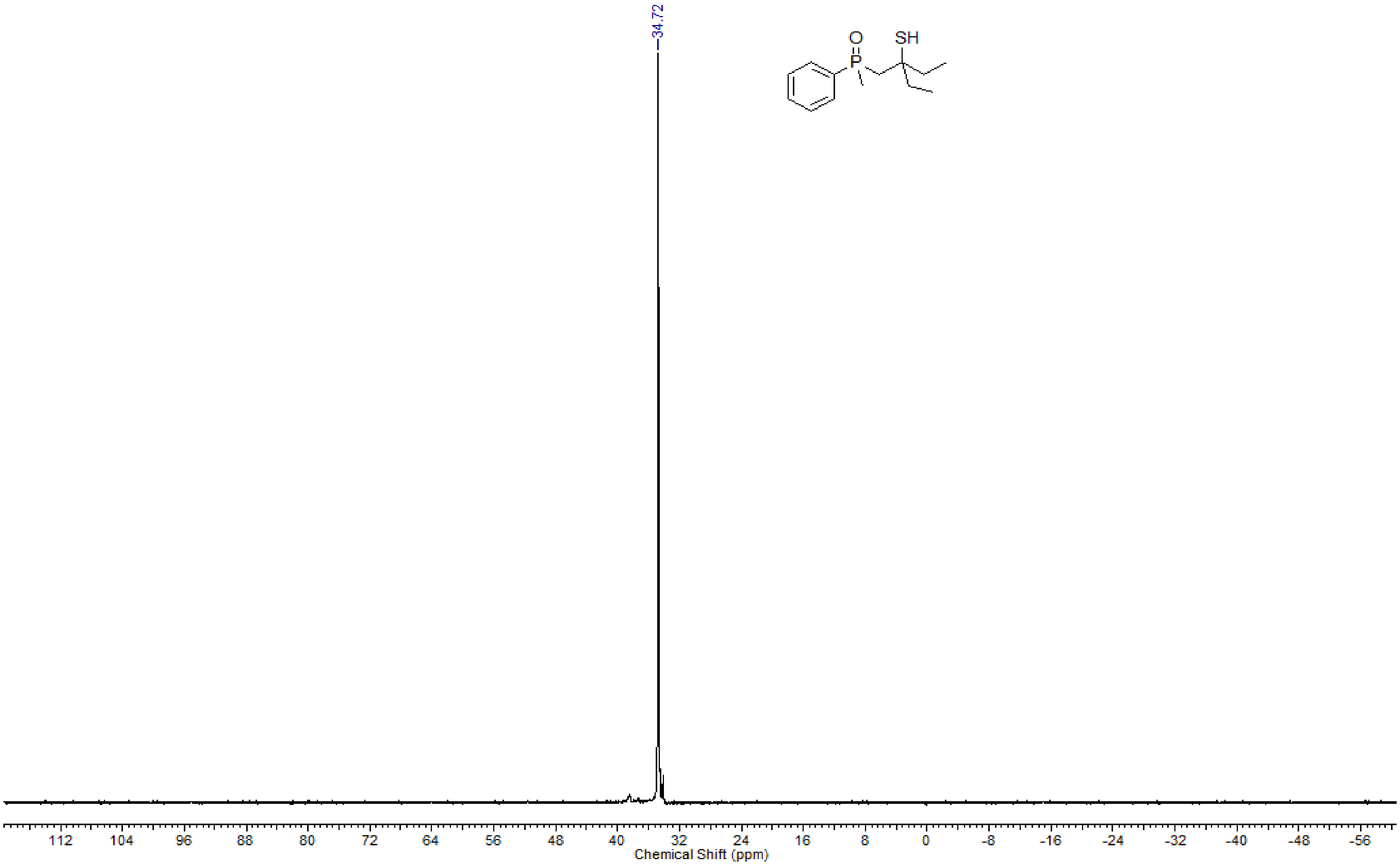

^{31}P NMR spectrum of (2-ethyl-2-mercaptobutyl)methylphenylphosphine oxide (**53**) (CDCl3, 202 MHz).

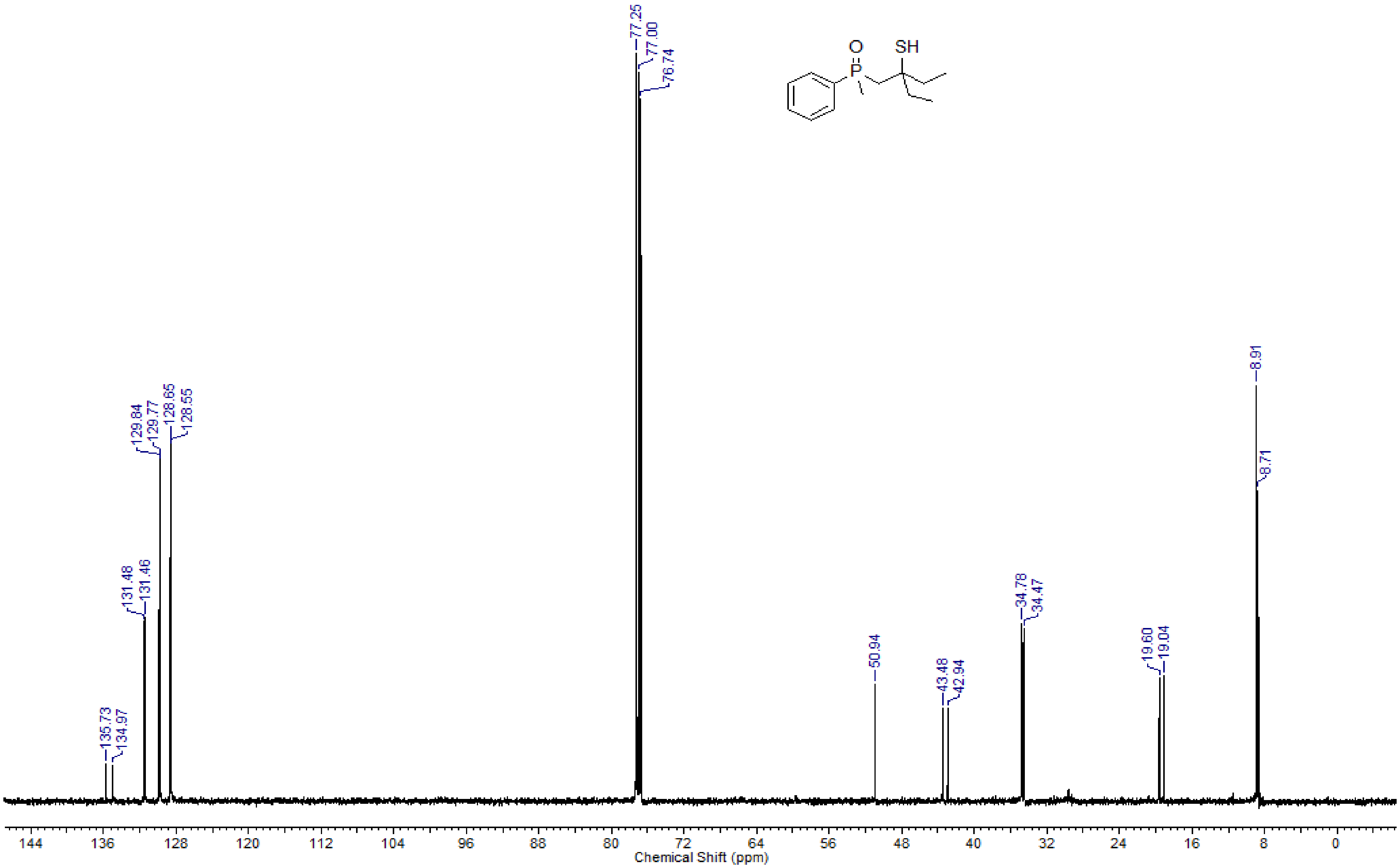

^{13}C NMR spectrum of (2-ethyl-2-mercaptobutyl)methylphenylphosphine oxide (**53**) (CDCl3, 126 MHz).

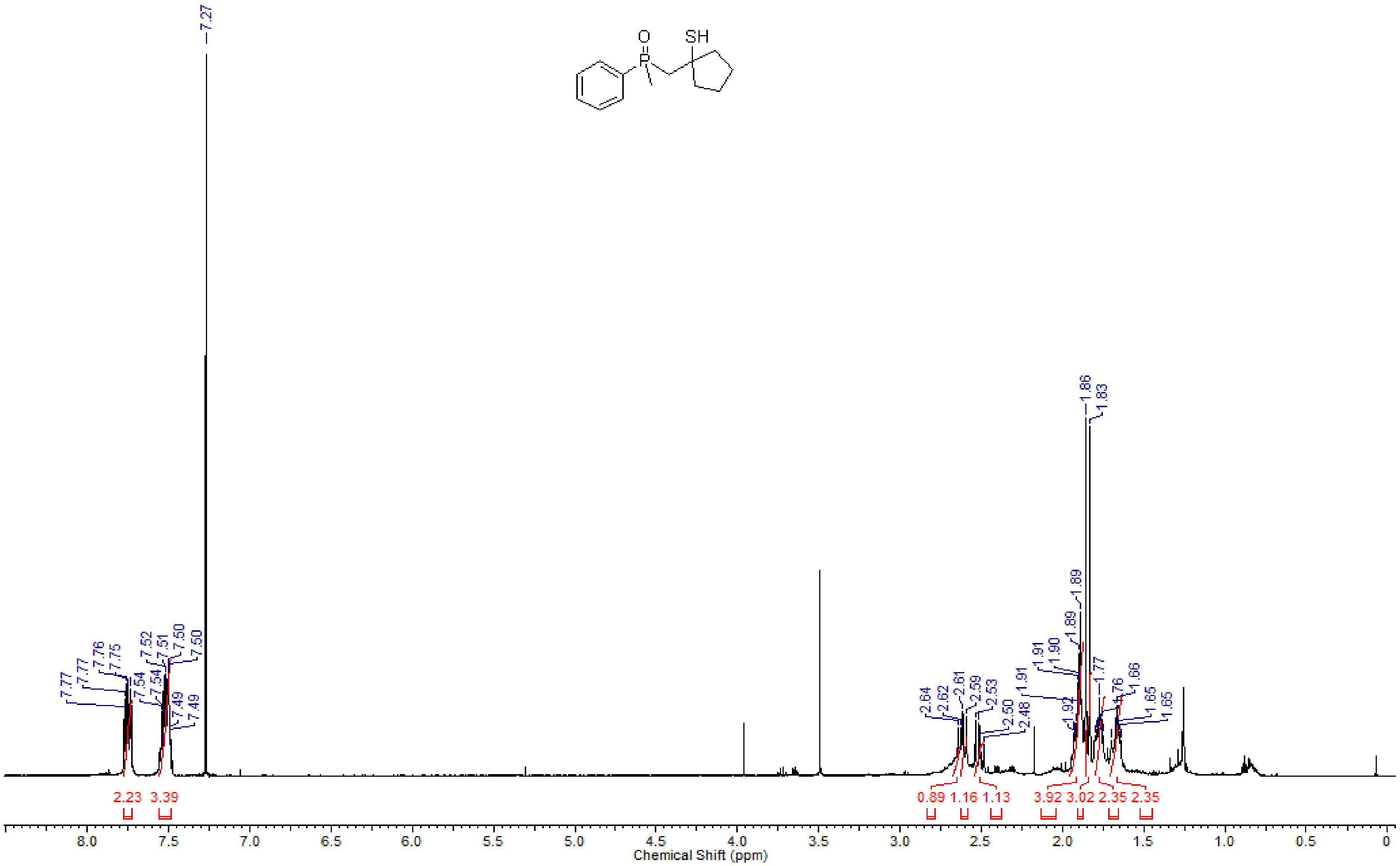

^{1}H NMR spectrum of [(1-mercapto)cyclopentylmethyl)]methylphenylphosphine oxide (**54**) (CDCl3, 500 MHz).

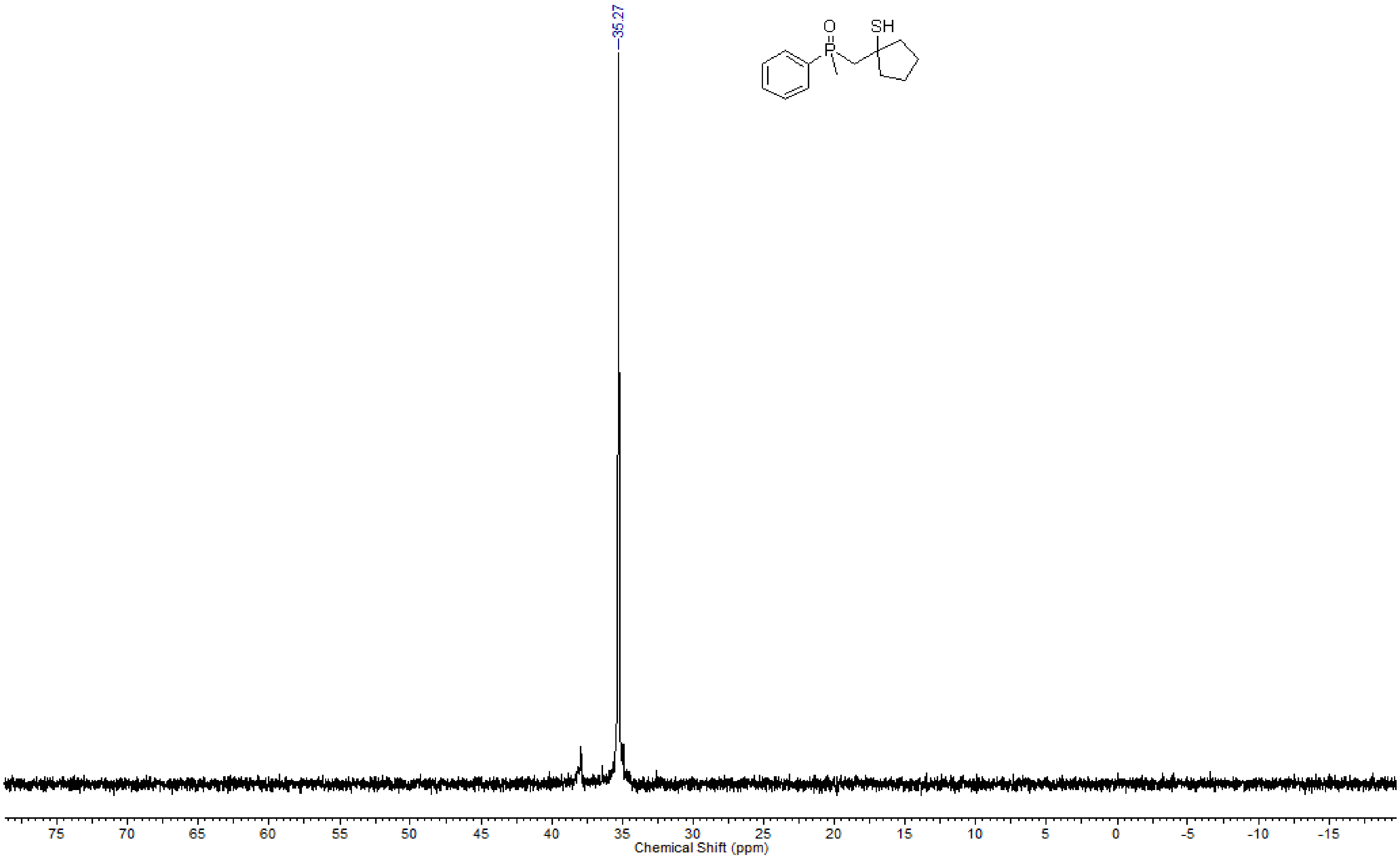

^{31}P NMR spectrum of [(1-mercapto)cyclopentylmethyl)]methylphenylphosphine oxide (**54**) (CDCl3, 202 MHz).

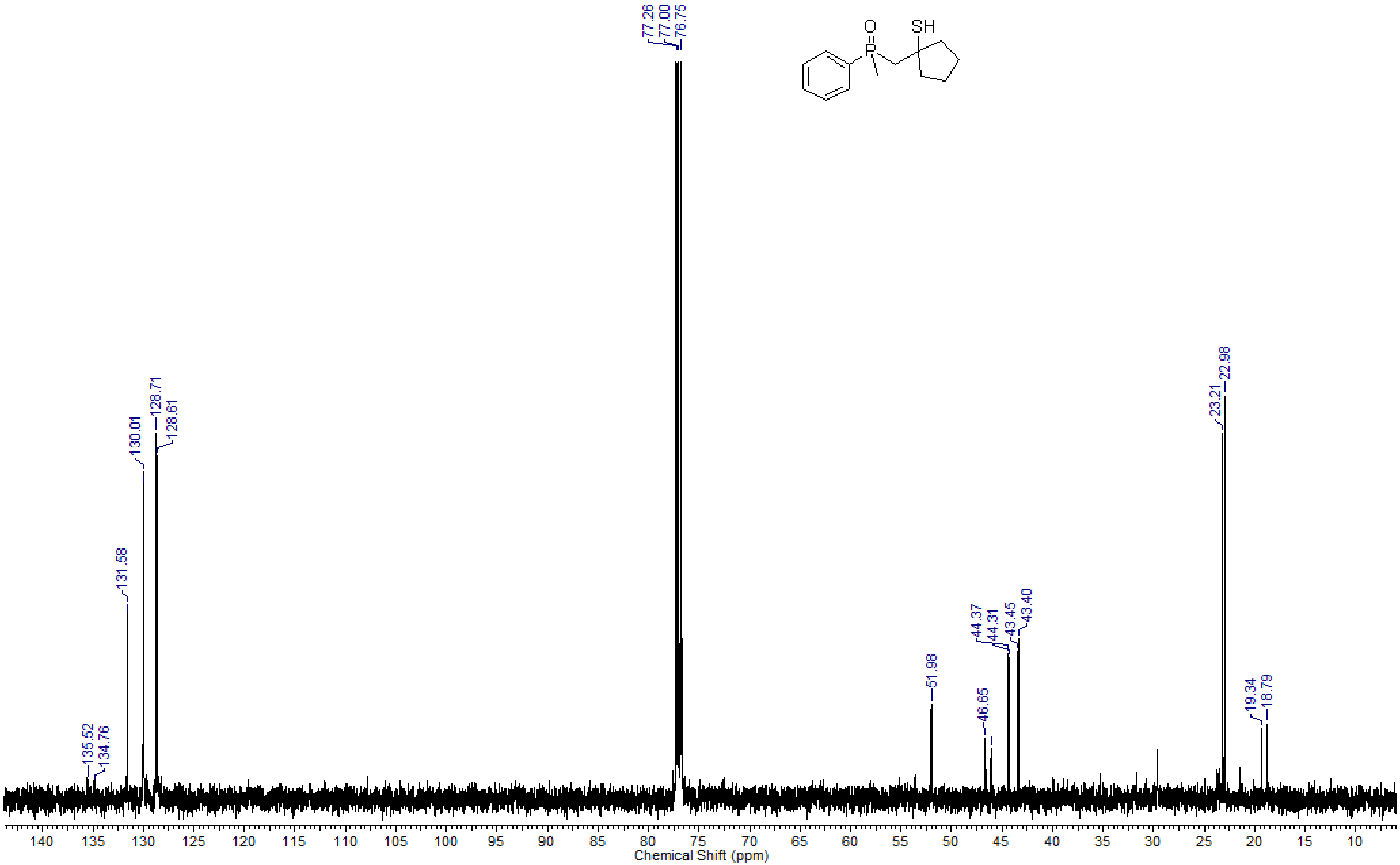

^{13}C NMR spectrum of [(1-mercapto)cyclopentylmethyl)]methylphenylphosphine oxide (**54**) (CDCl3, 126 MHz).

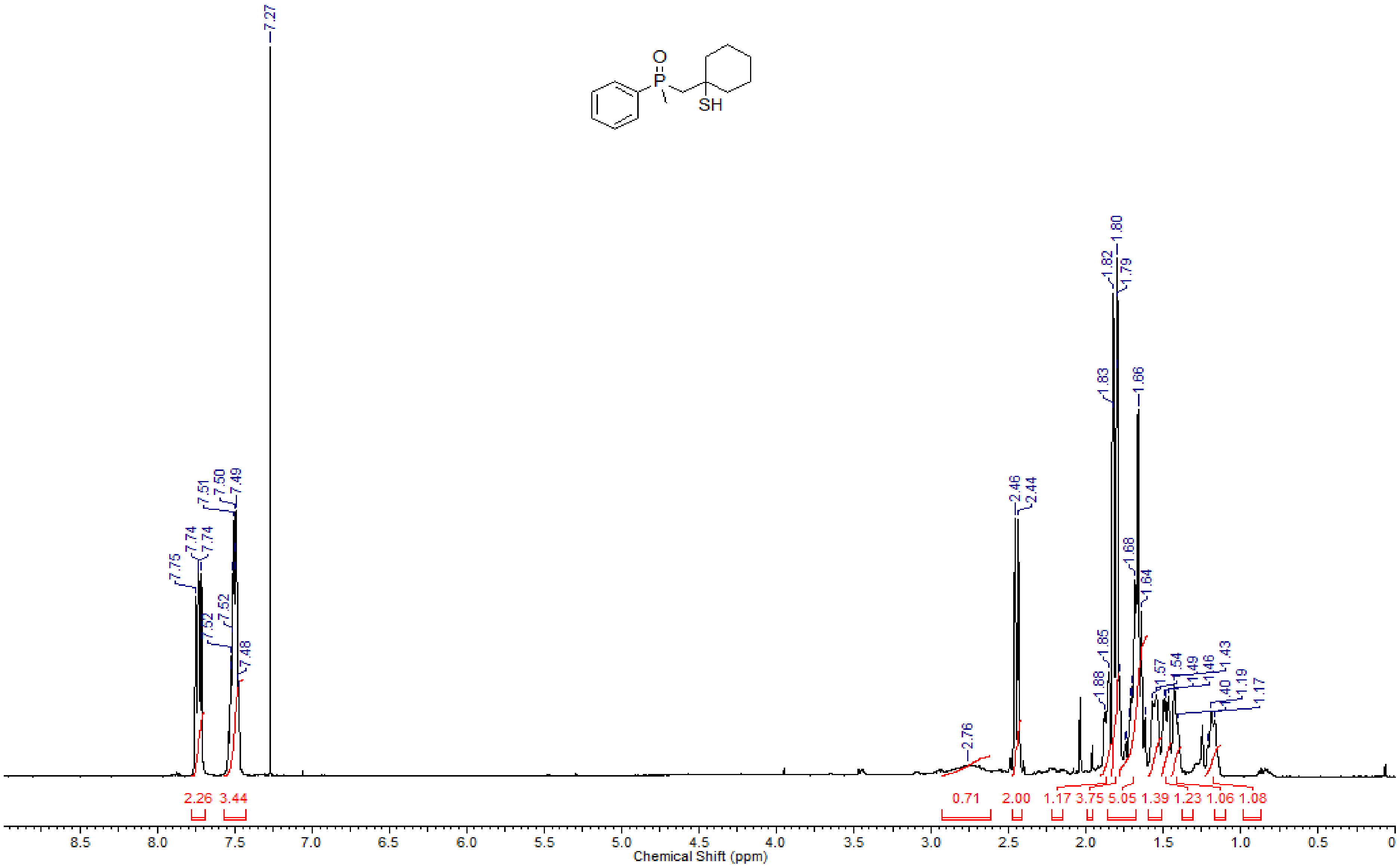

^{1}H NMR spectrum of [(1-mercapto)cyclohexylmethyl)]methylphenylphosphine oxide (**55**) (CDCl3, 500 MHz).

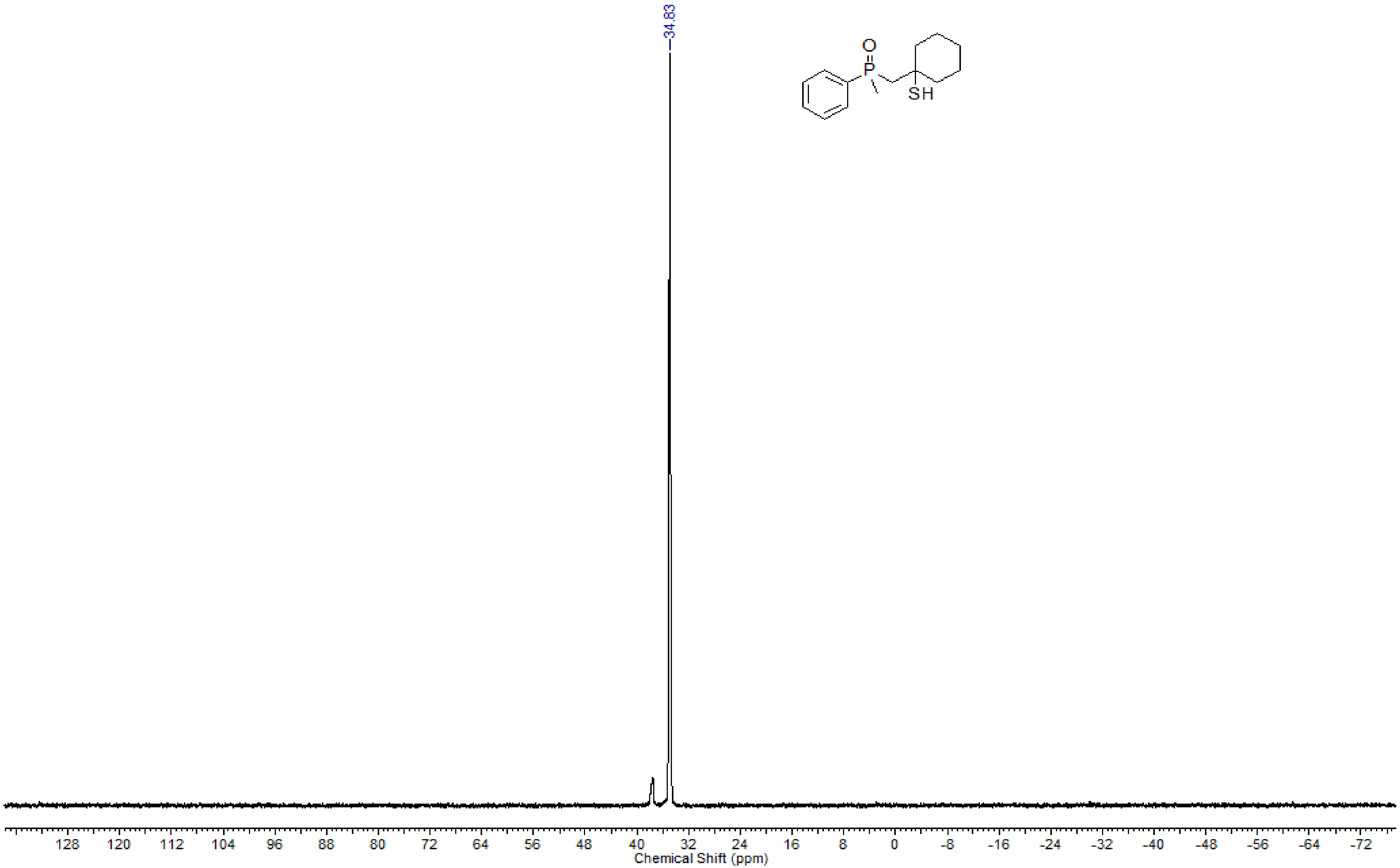

^{31}P NMR spectrum of [(1-mercapto)cyclohexylmethyl)]methylphenylphosphine oxide (**55**) (CDCl3, 202 MHz).

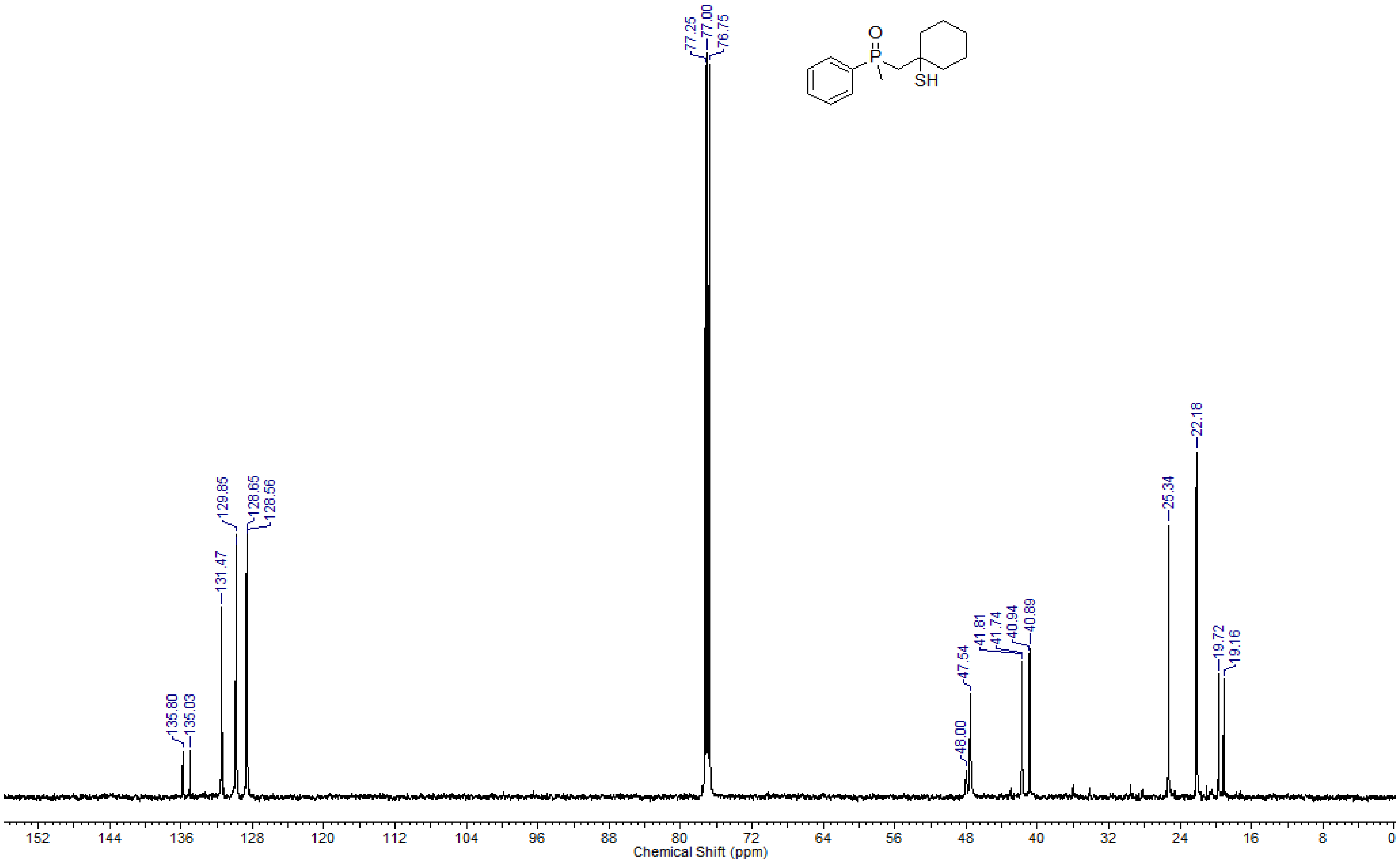

^{13}C NMR spectrum of [(1-mercapto)cyclohexylmethyl)]methylphenylphosphine oxide (**55**) (CDCl3, 126 MHz).

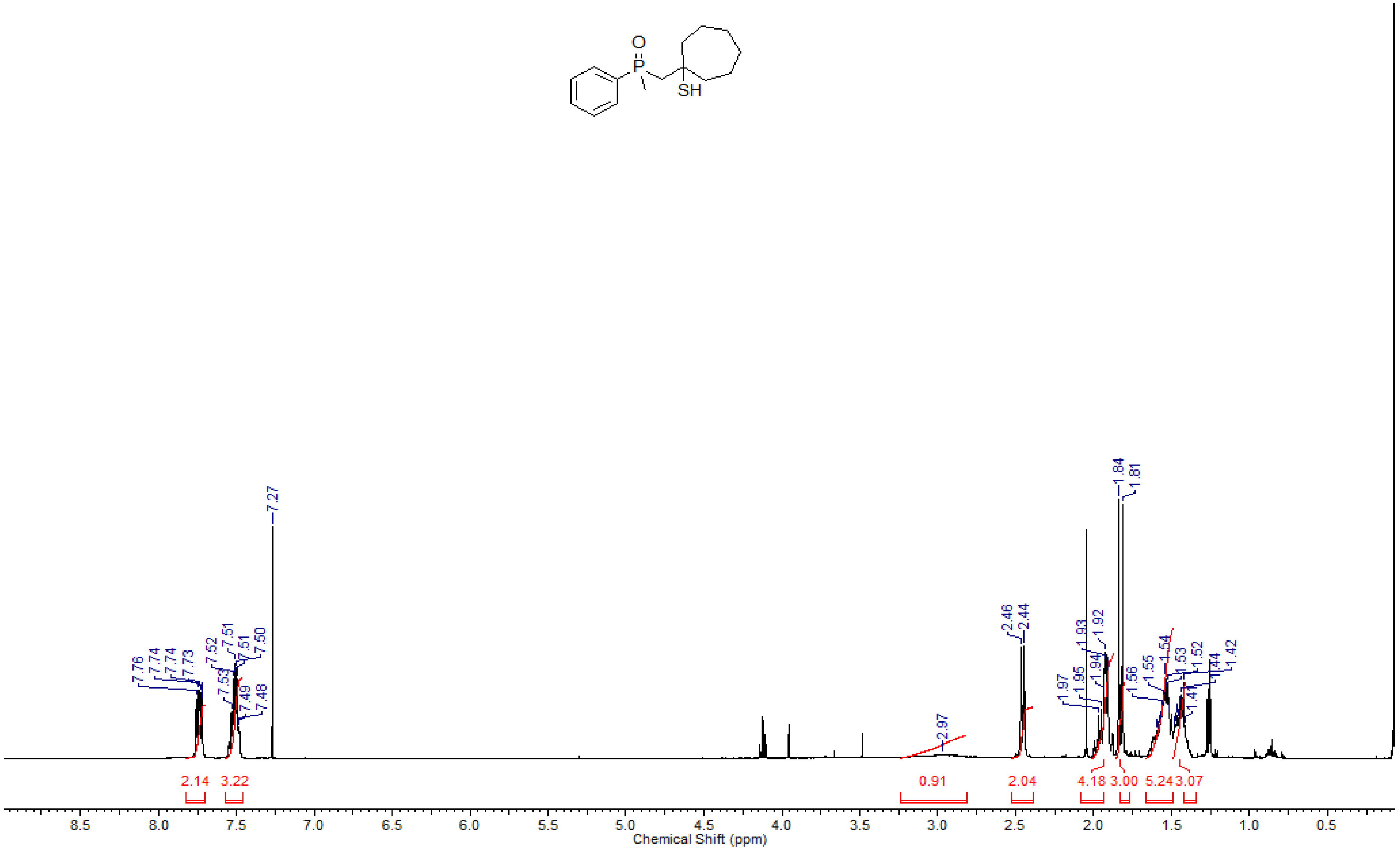

^{1}H NMR spectrum of [(1-mercapto)cycloheptylmethyl)]methylphenylphosphine oxide (**56**) (CDCl3, 500 MHz).

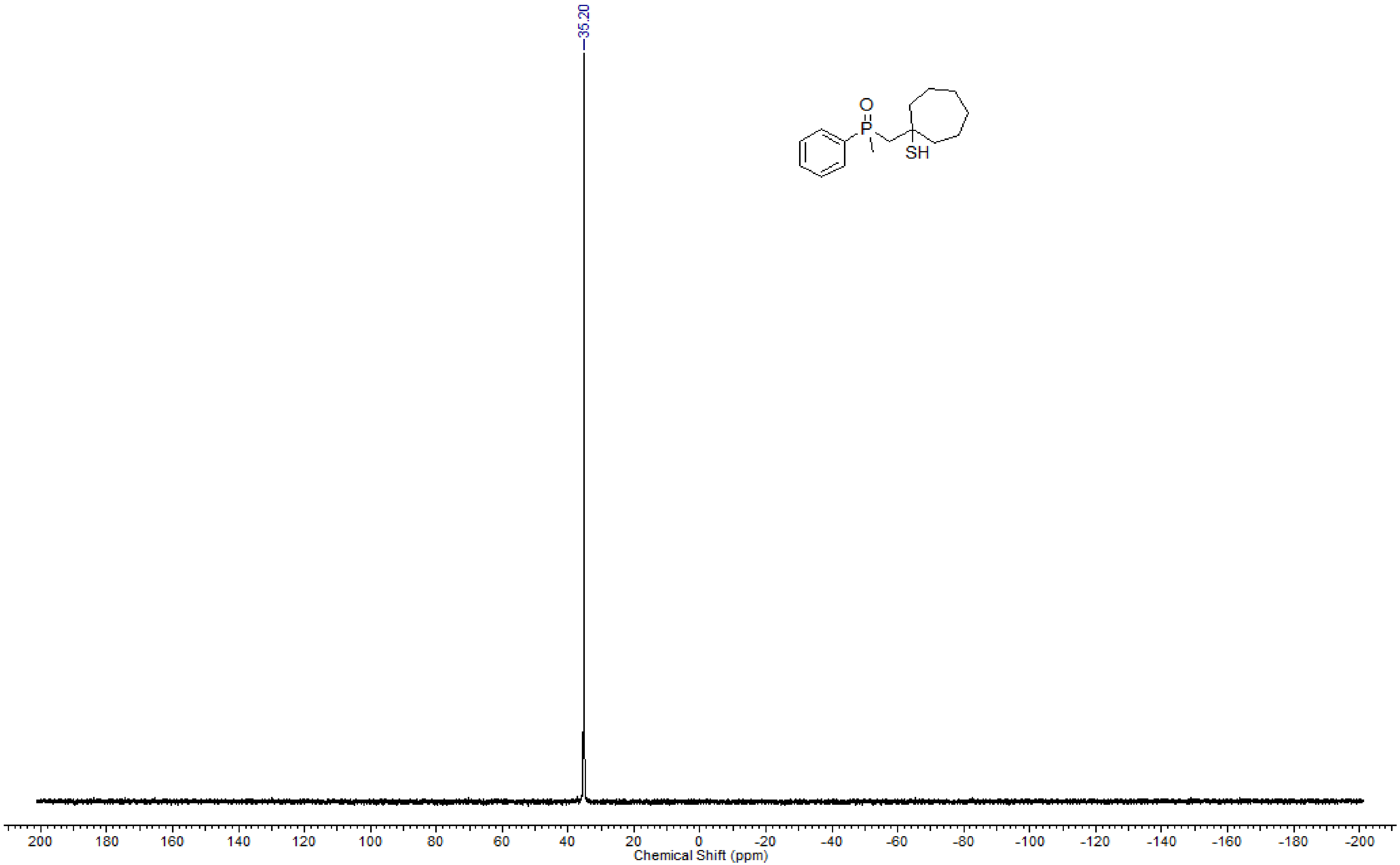

^{31}P NMR spectrum of [(1-mercapto)cycloheptylmethyl)]methylphenylphosphine oxide (**56**) (CDCl3, 202 MHz).

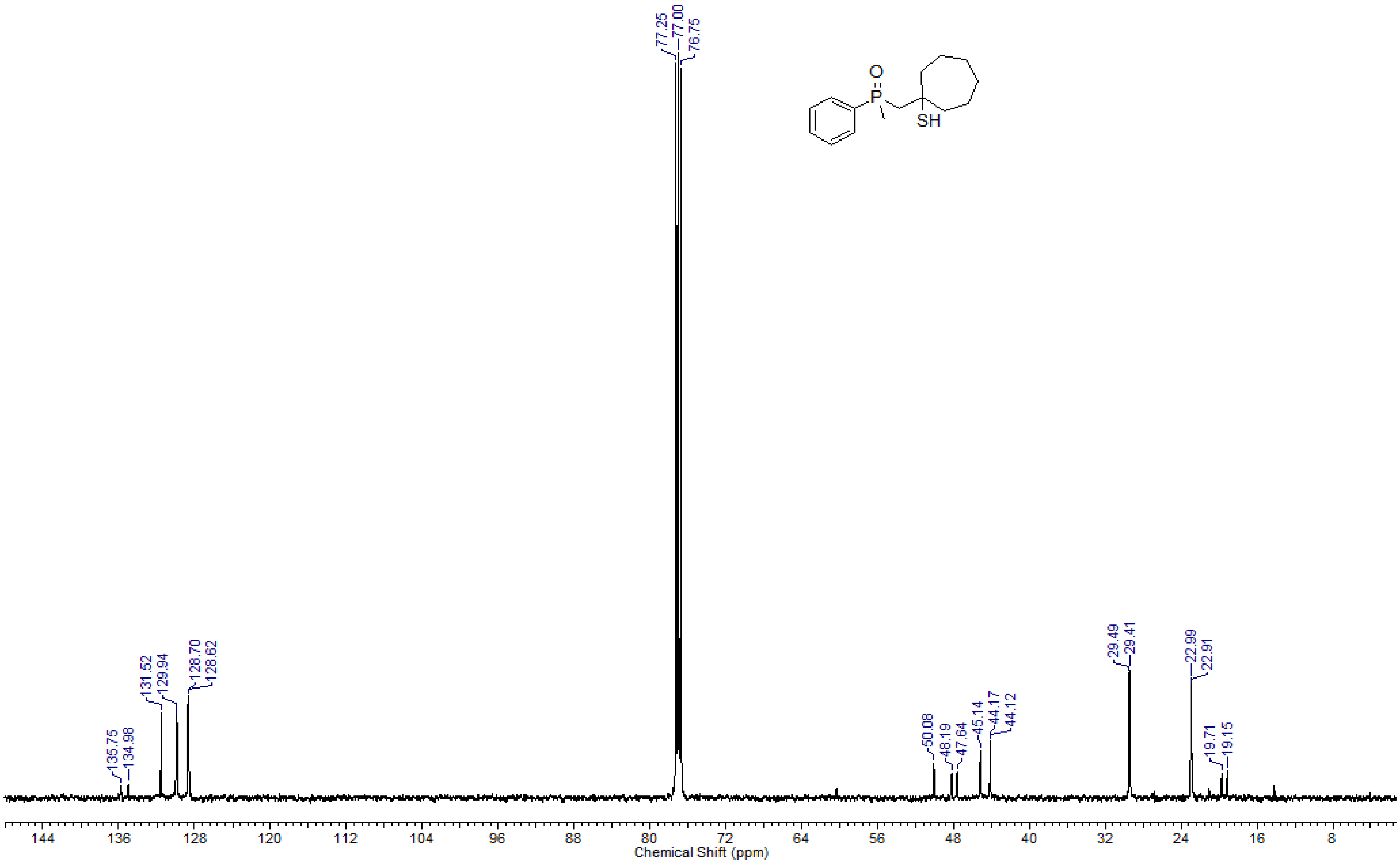

^{13}C NMR spectrum of [(1-mercapto)cycloheptylmethyl)]methylphenylphosphine oxide (**56**) (CDCl3, 126 MHz).

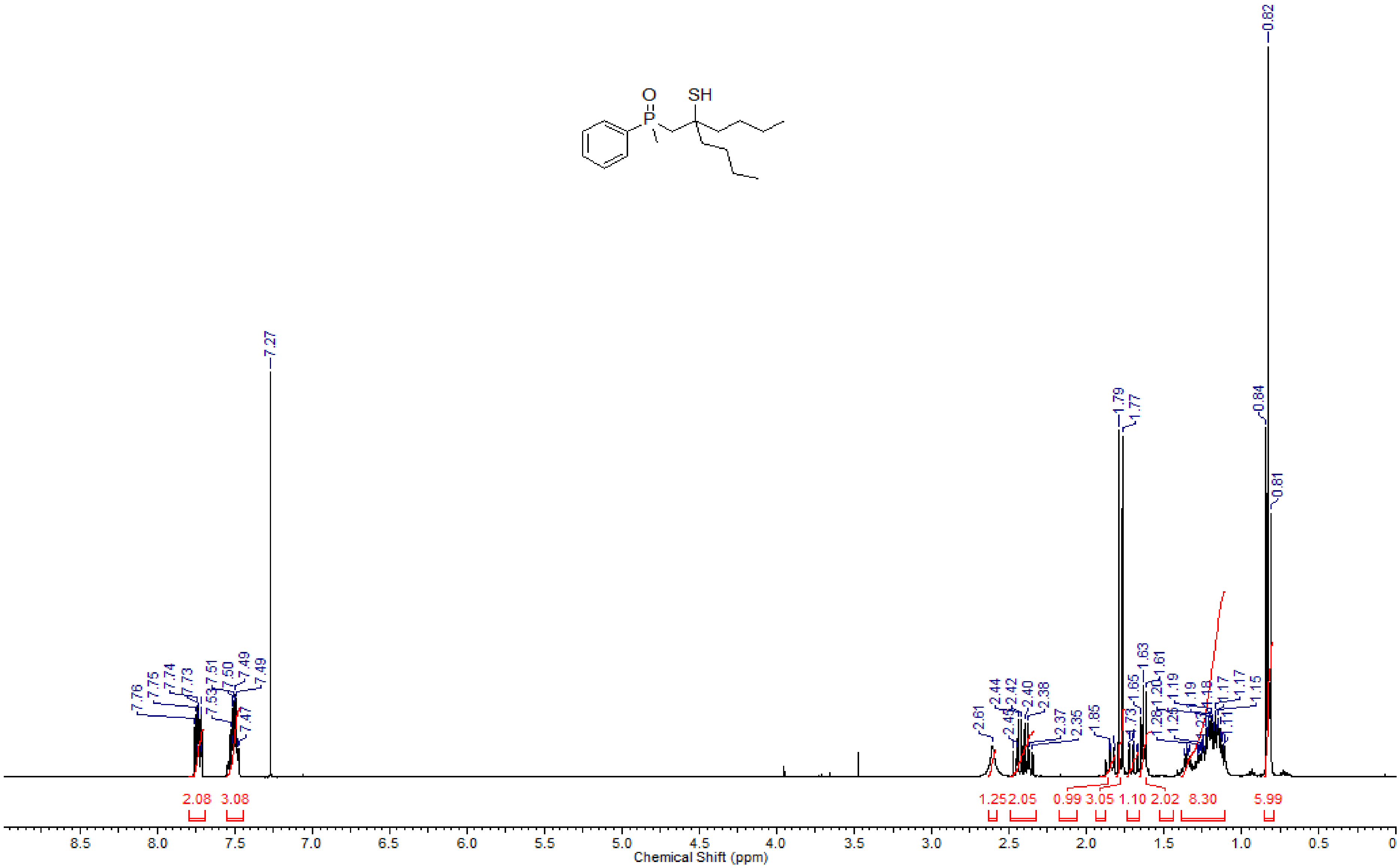

^{1}H NMR spectrum of (2-butyl-2-mercaptohexyl)methylphenylphosphine sulfide (**57**) (CDCl3, 500 MHz).

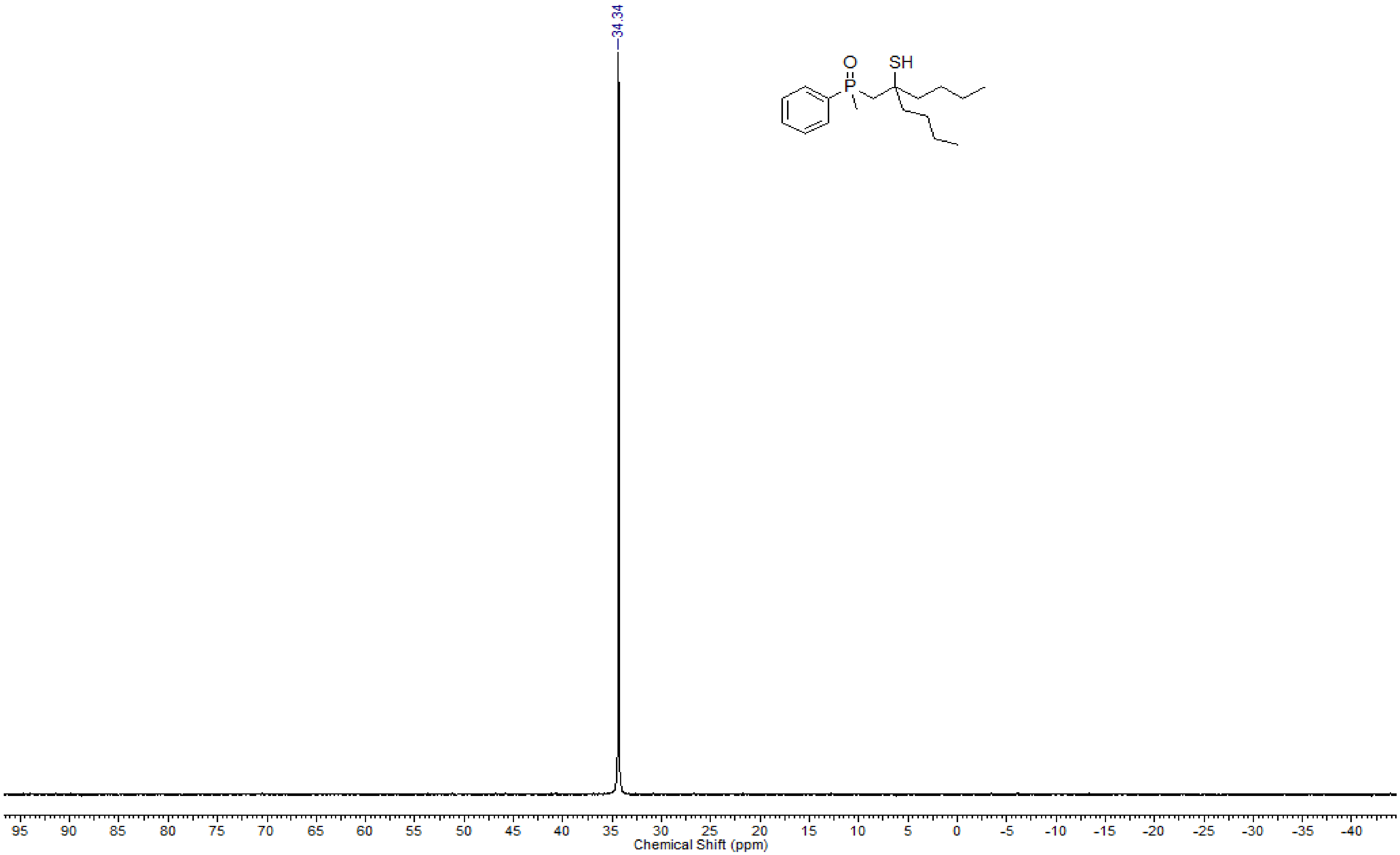

^{31}P NMR spectrum of (2-butyl-2-mercaptohexyl)methylphenylphosphine sulfide (**57**) (CDCl3, 202 MHz).

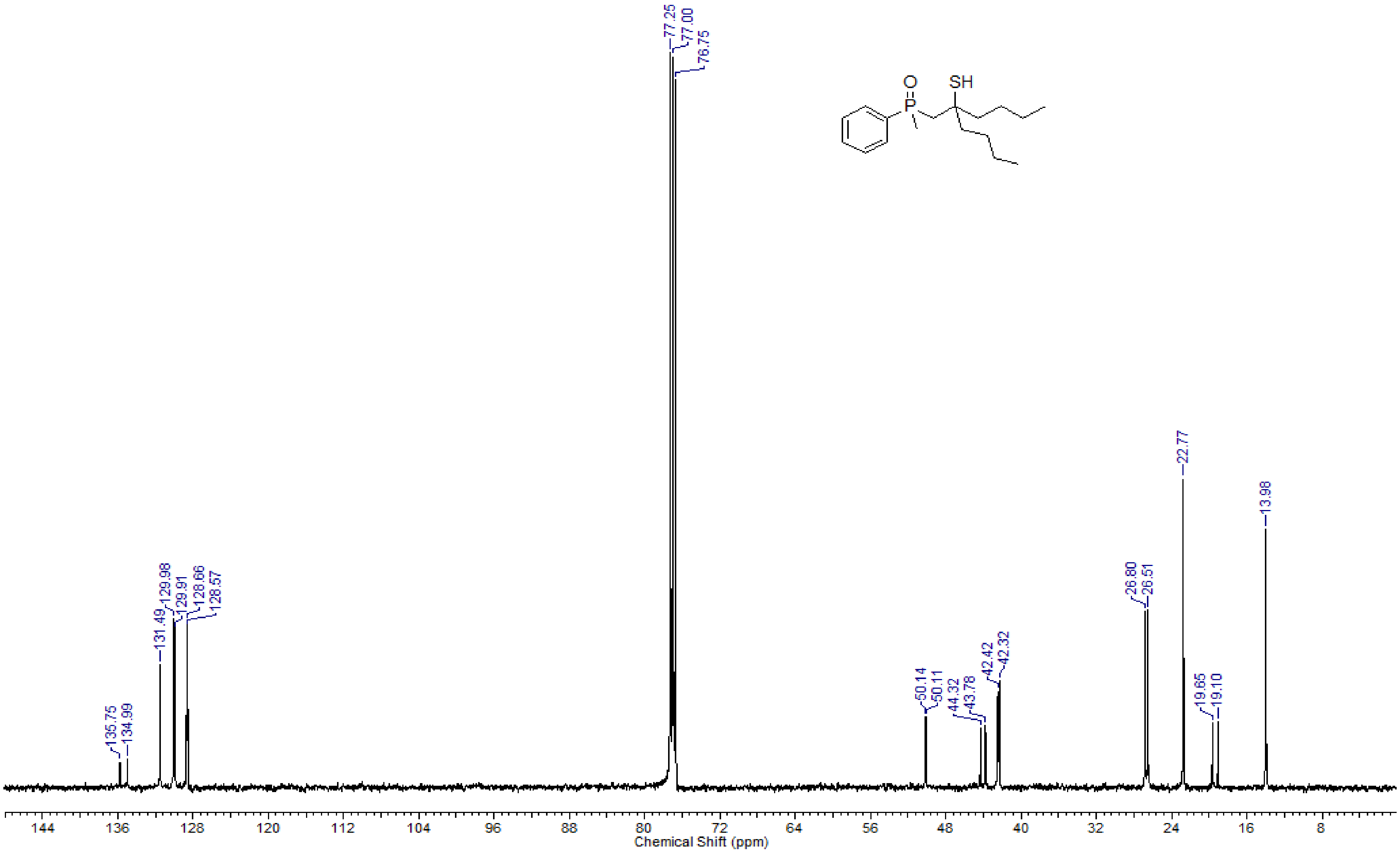

^{13}C NMR spectrum of (2-butyl-2-mercaptohexyl)methylphenylphosphine sulfide (**57**) (CDCl3, 126 MHz).

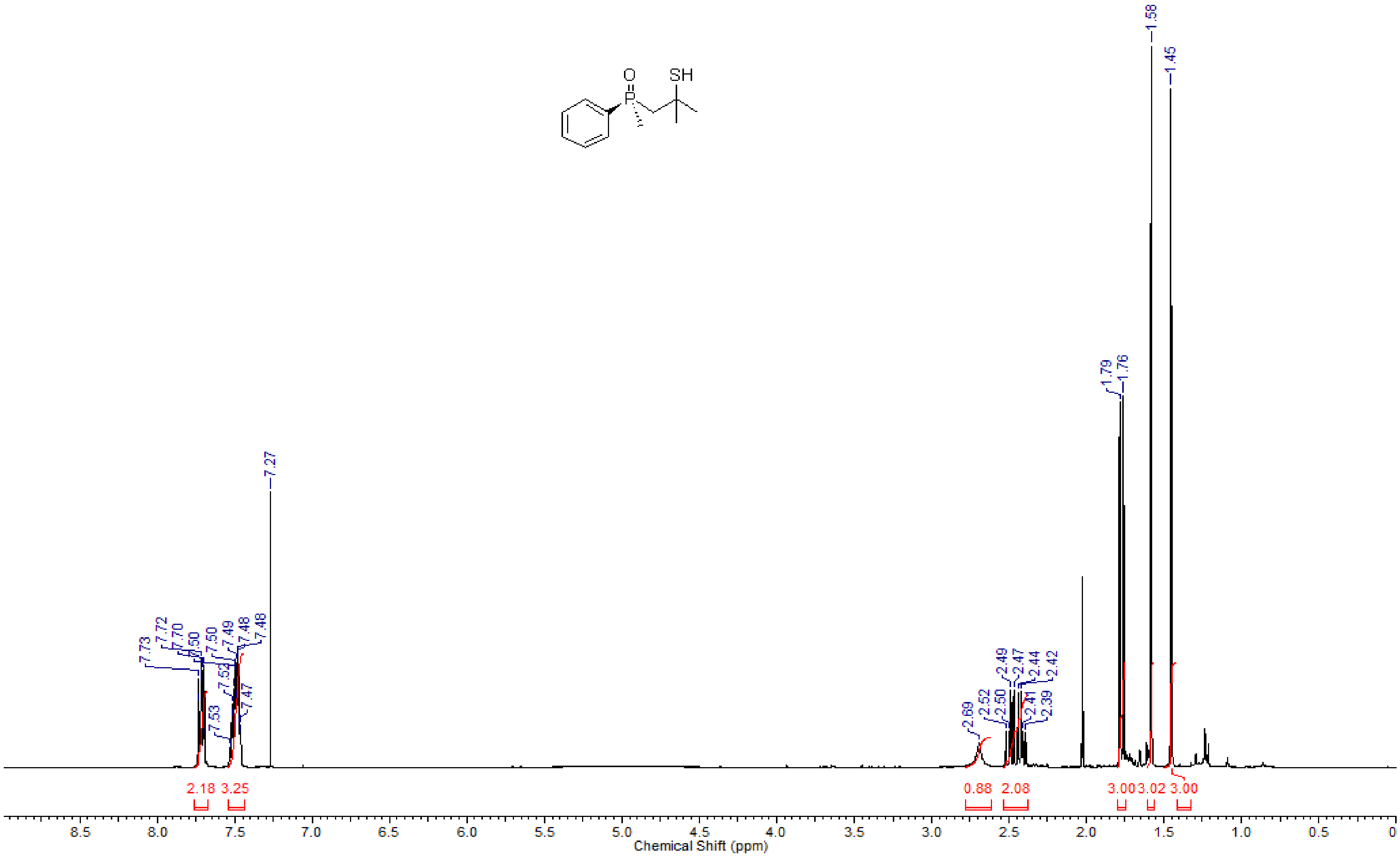

^{1}H NMR spectrum of (R_P)-(2-methyl-2-mercaptopropyl)methylphenylphosphine oxide (R_P)-(**52**) (CDCl3, 500 MHz).

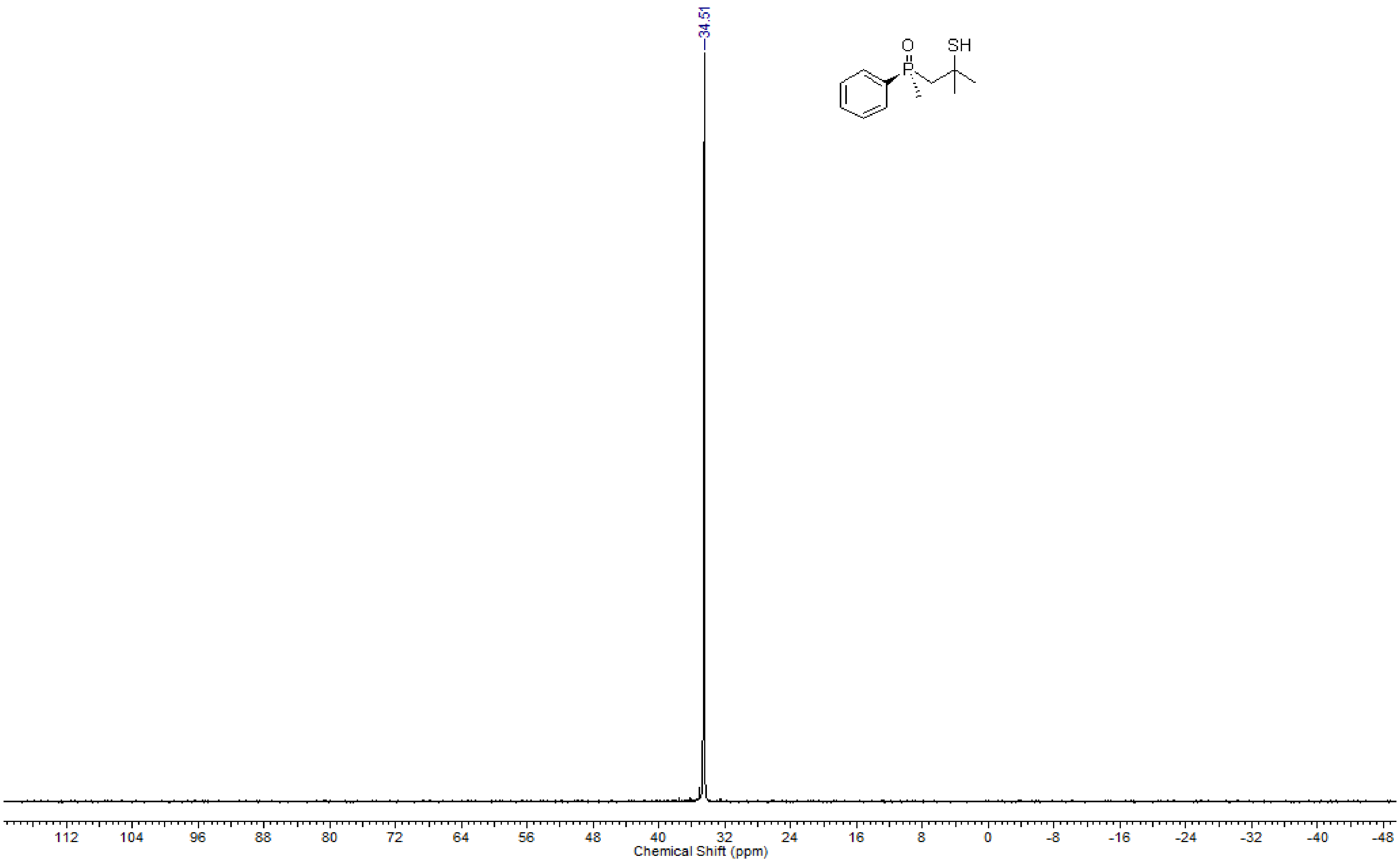

^{31}P NMR spectrum of (RP)-(2-methyl-2-mercaptopropyl)methylphenylphosphine oxide (RP)-(**52**) (CDCl3, 202 MHz).

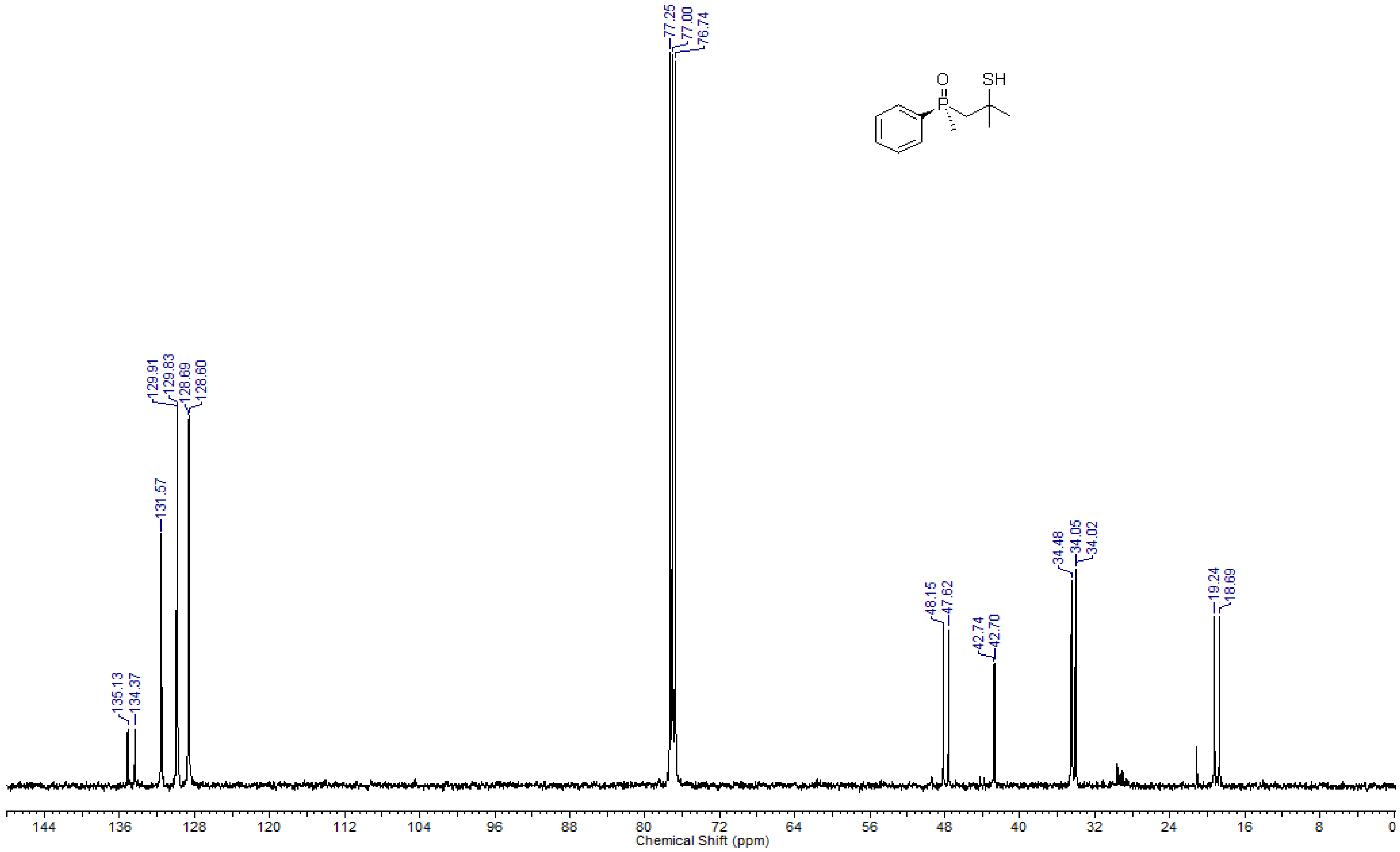

^{13}C NMR spectrum of (*R*P)-(2-methyl-2-mercaptopropyl)methylphenylphosphine oxide (*R*P)-(**52**) (CDCl3, 126 MHz).

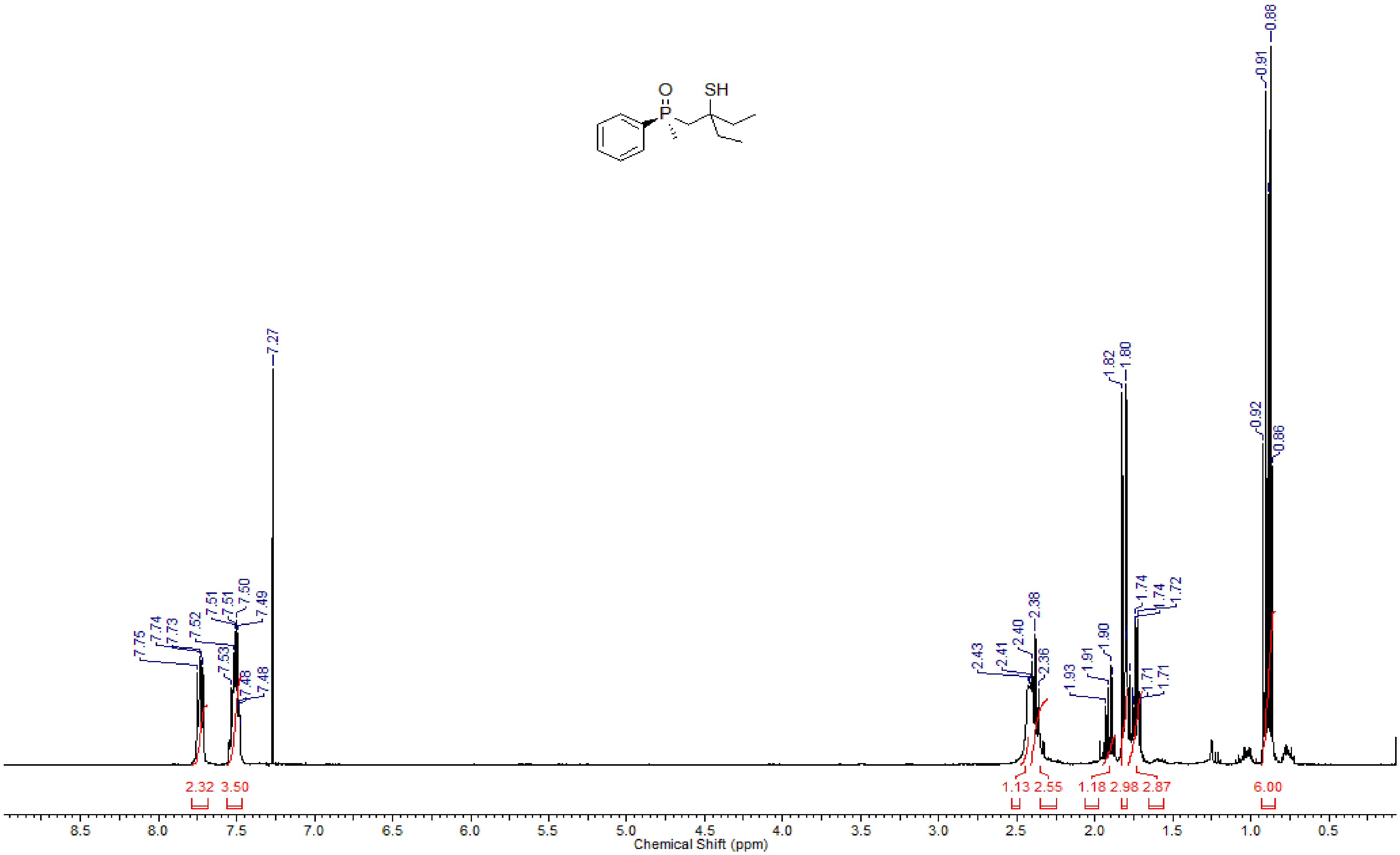

^{1}H NMR spectrum of (R_P)-(2-ethyl-2-mercaptobutyl)methylphenylphosphine oxide (R_P)-(**53**) (CDCl3, 500 MHz).

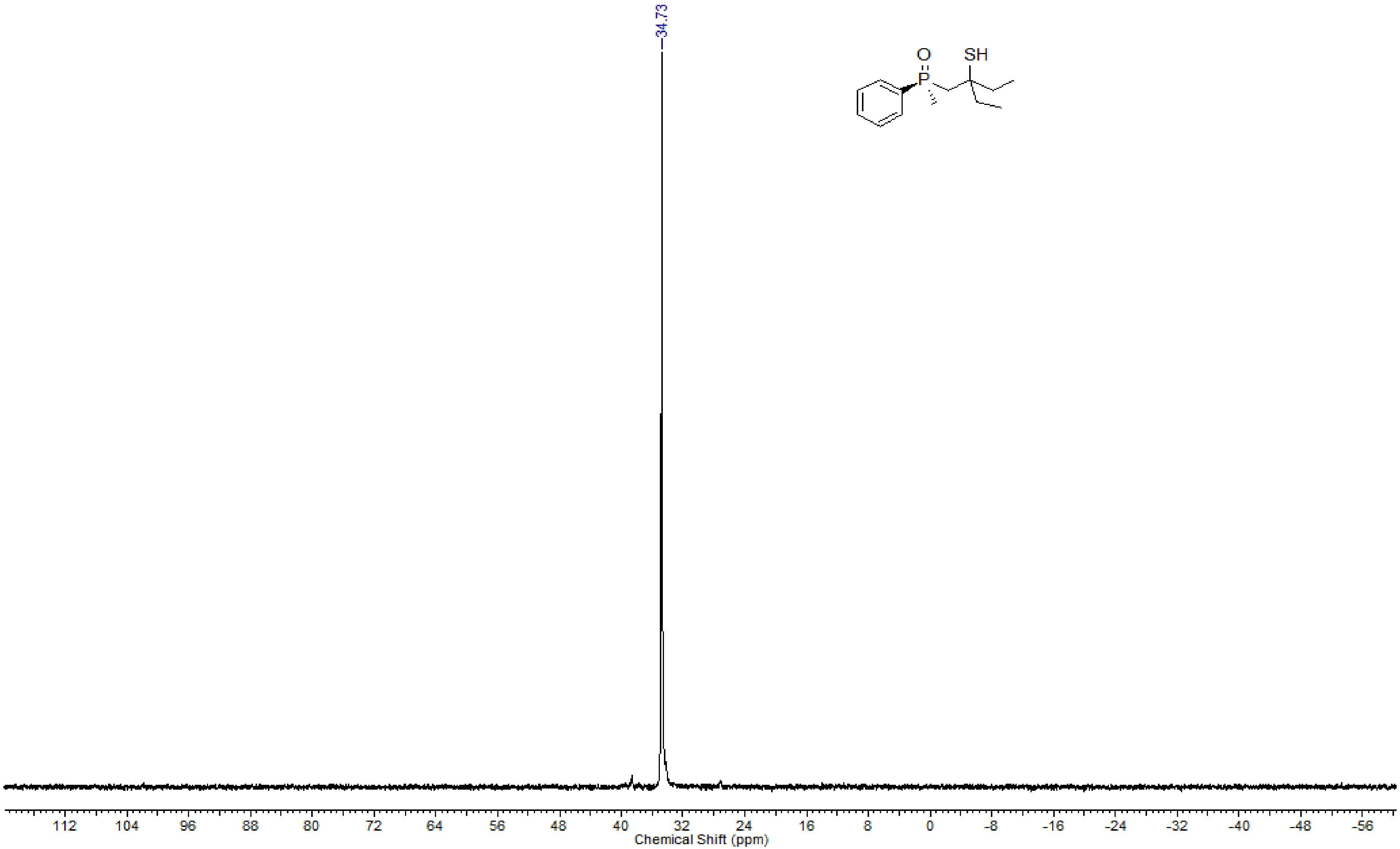

^{31}P NMR spectrum of (R_P)-(2-ethyl-2-mercaptobutyl)methylphenylphosphine oxide (R_P)-(**53**) (CDCl3, 202 MHz).

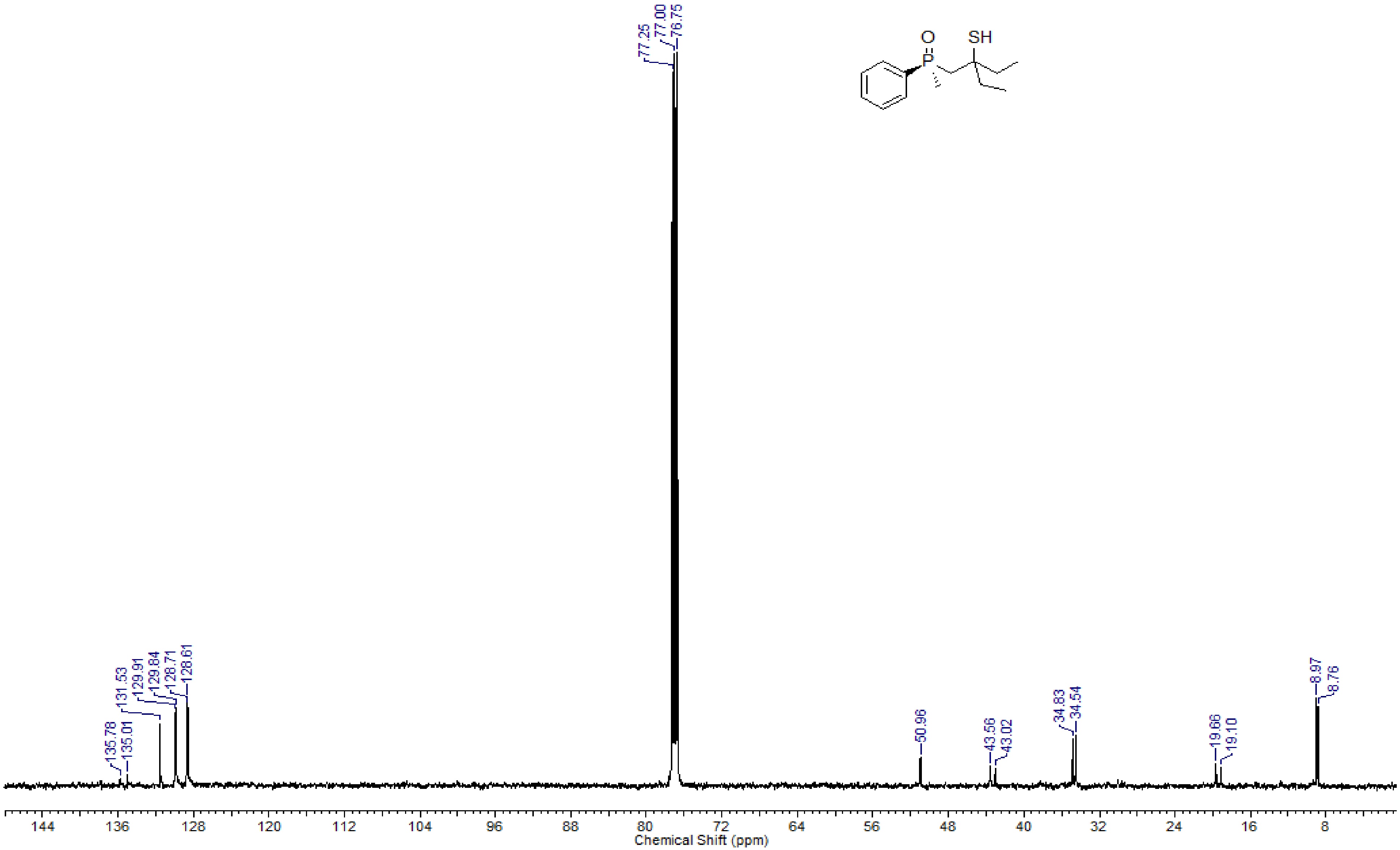

^{13}C NMR spectrum of (R_P)-(2-ethyl-2-mercaptobutyl)methylphenylphosphine oxide (R_P)-(**53**) (CDCl3, 126 MHz).

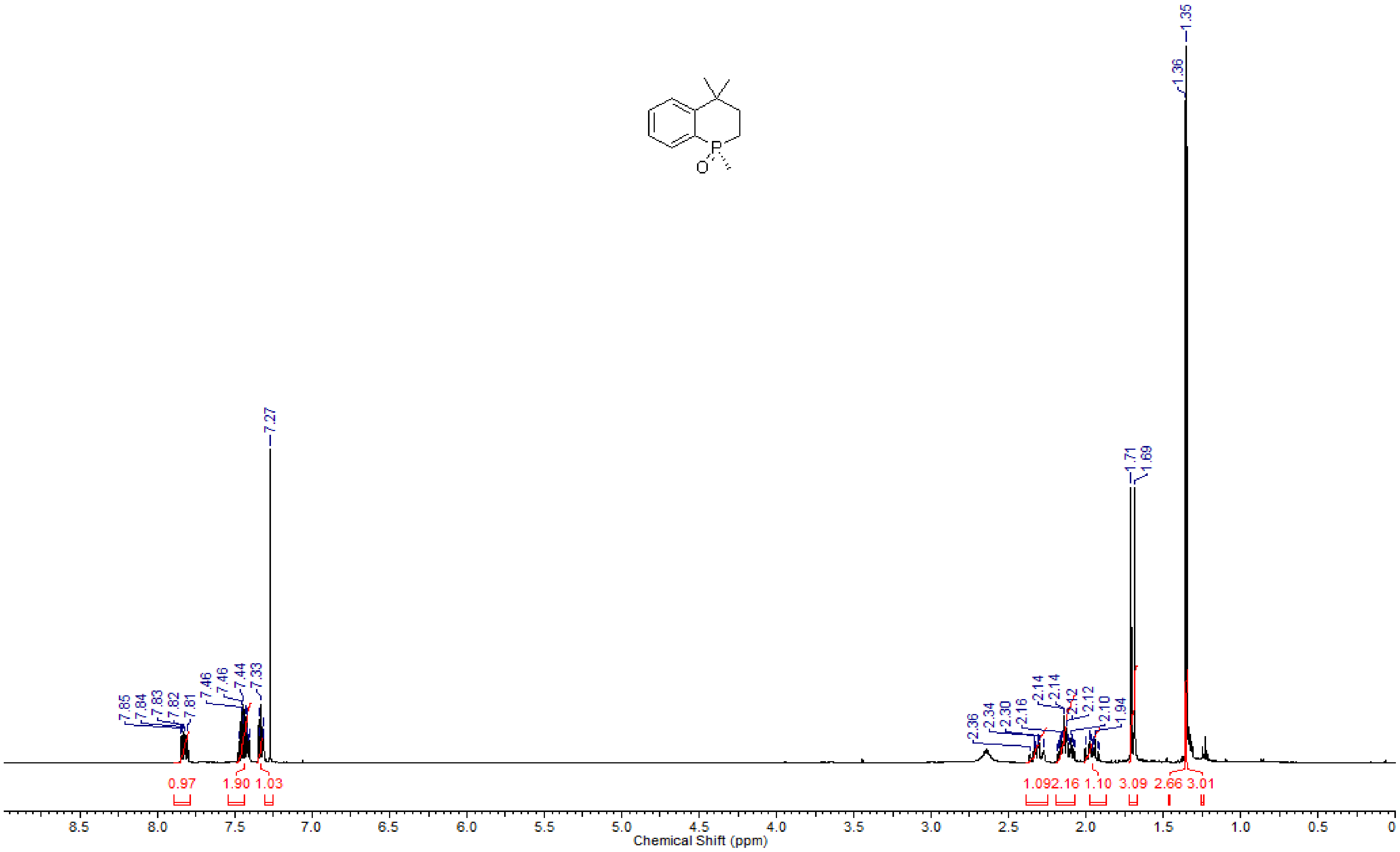

^{1}H NMR spectrum of (R_P)-1,4,4-Trimethyl-1,2,3,4-tetrahydrophosphinoline 1-oxide (R_P)**-26** (CDCl3, 500 MHz).

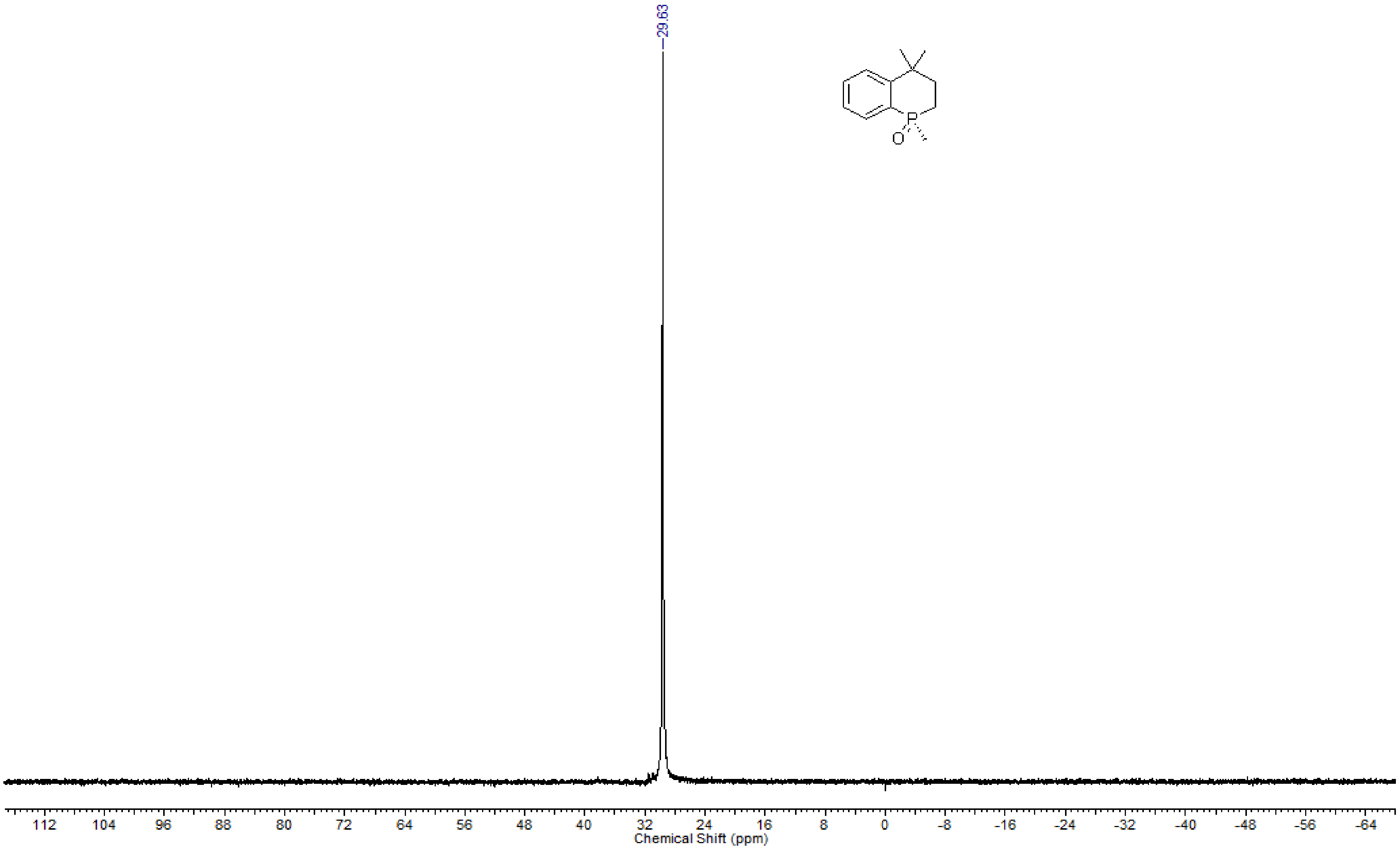

^{31}P NMR spectrum of (R_{P})-1,4,4-Trimethyl-1,2,3,4-tetrahydrophosphinoline 1- oxide (R_{P})-**26** (CDCl3, 202 MHz).

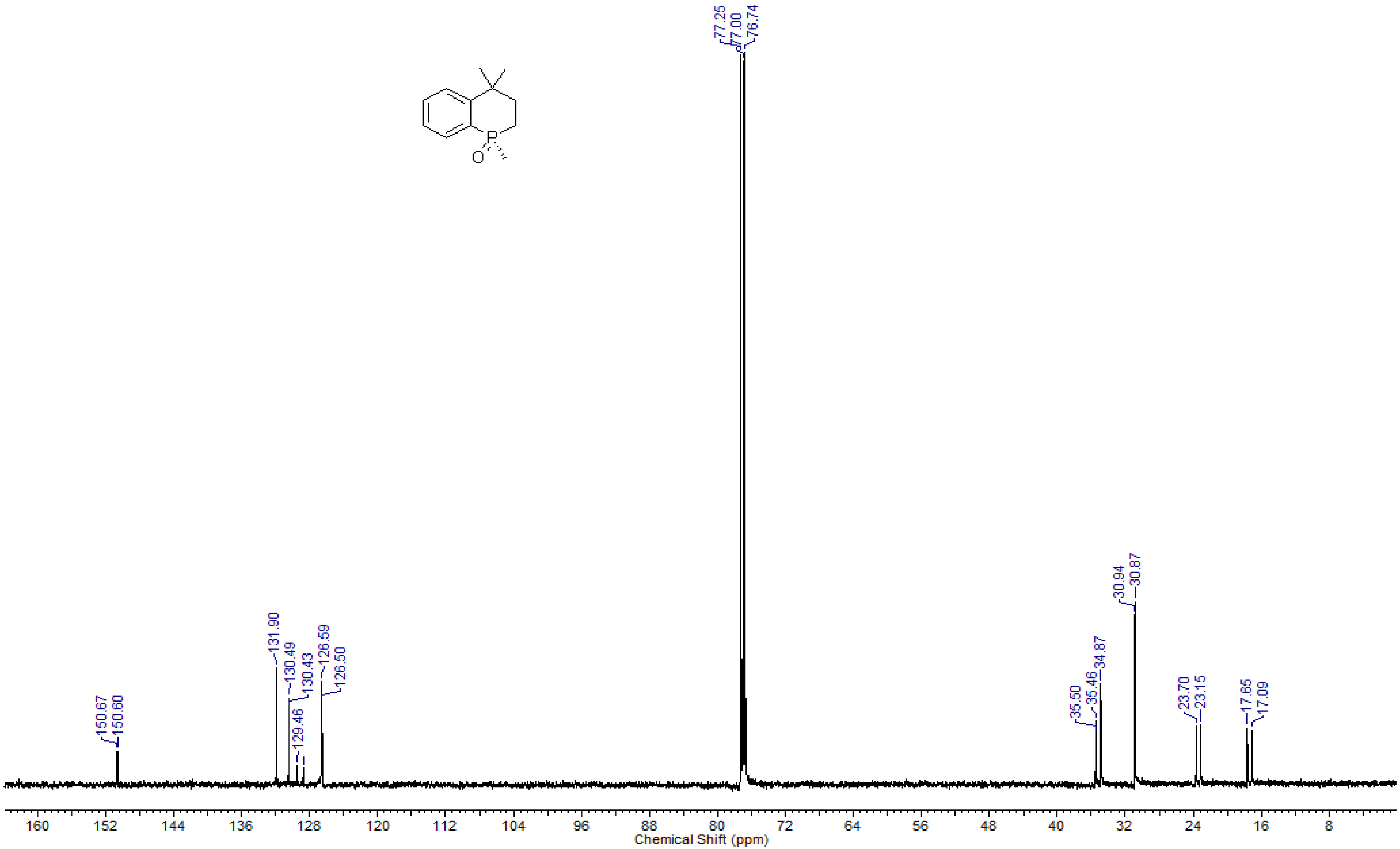

^{13}C NMR spectrum of (R_P)-1,4,4-Trimethyl-1,2,3,4-tetrahydrophosphinoline 1-oxide (R_P)-**26** (CDCl3, 126 MHz).

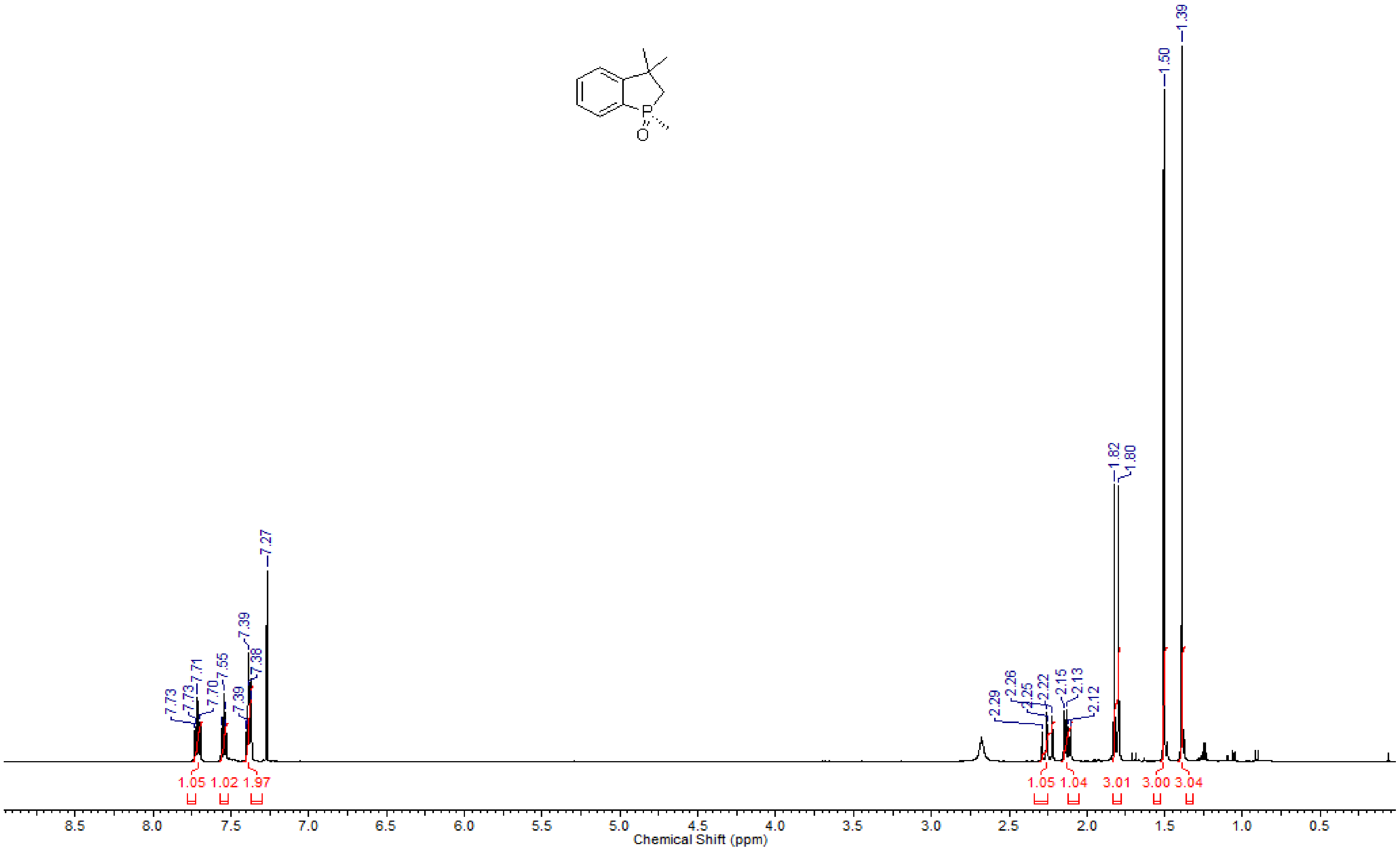

^{1}H NMR spectrum of (R_P)-1,3,3-Trimethylphosphindoline 1-oxide (R_P)-**3** (CDCl3, 500 MHz).

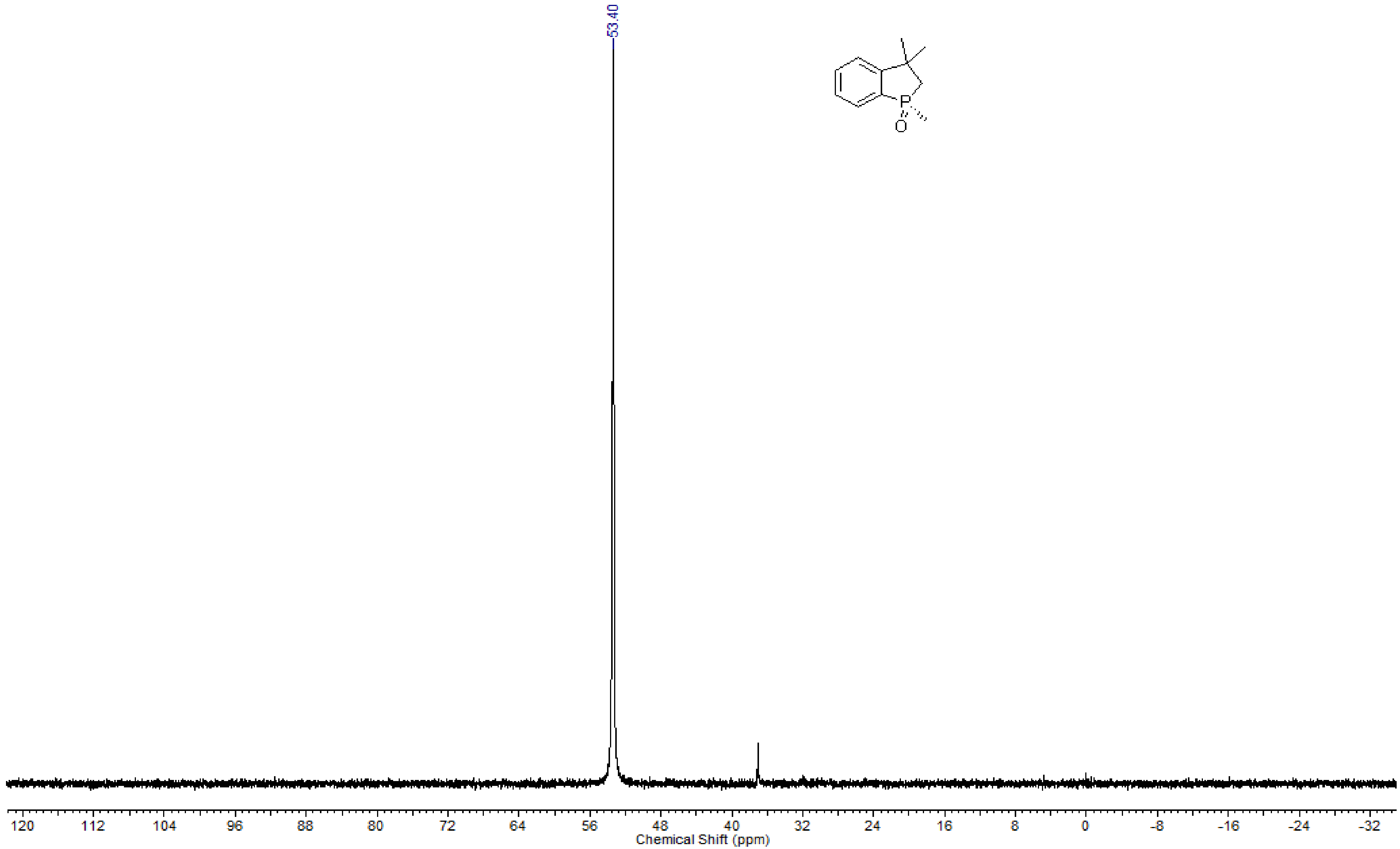

^{31}P NMR spectrum of (R_P)-1,3,3-Trimethylphosphindoline 1-oxide (R_P)-**3** (CDCl3, 202 MHz).

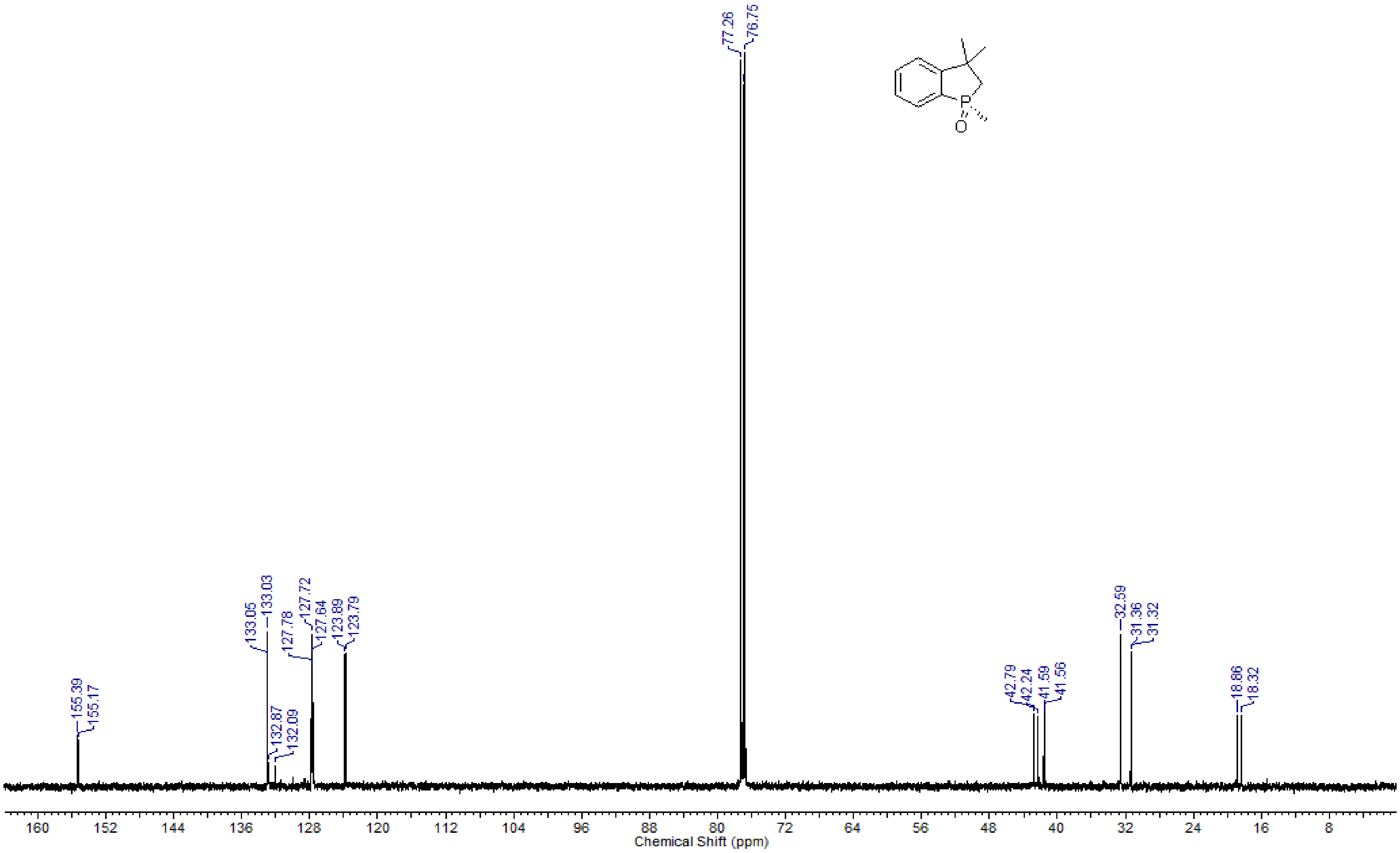

^{13}C NMR spectrum of (R_P)-1,3,3-Trimethylphosphindoline 1-oxide (R_P)-**3** (CDCl3, 126 MHz).

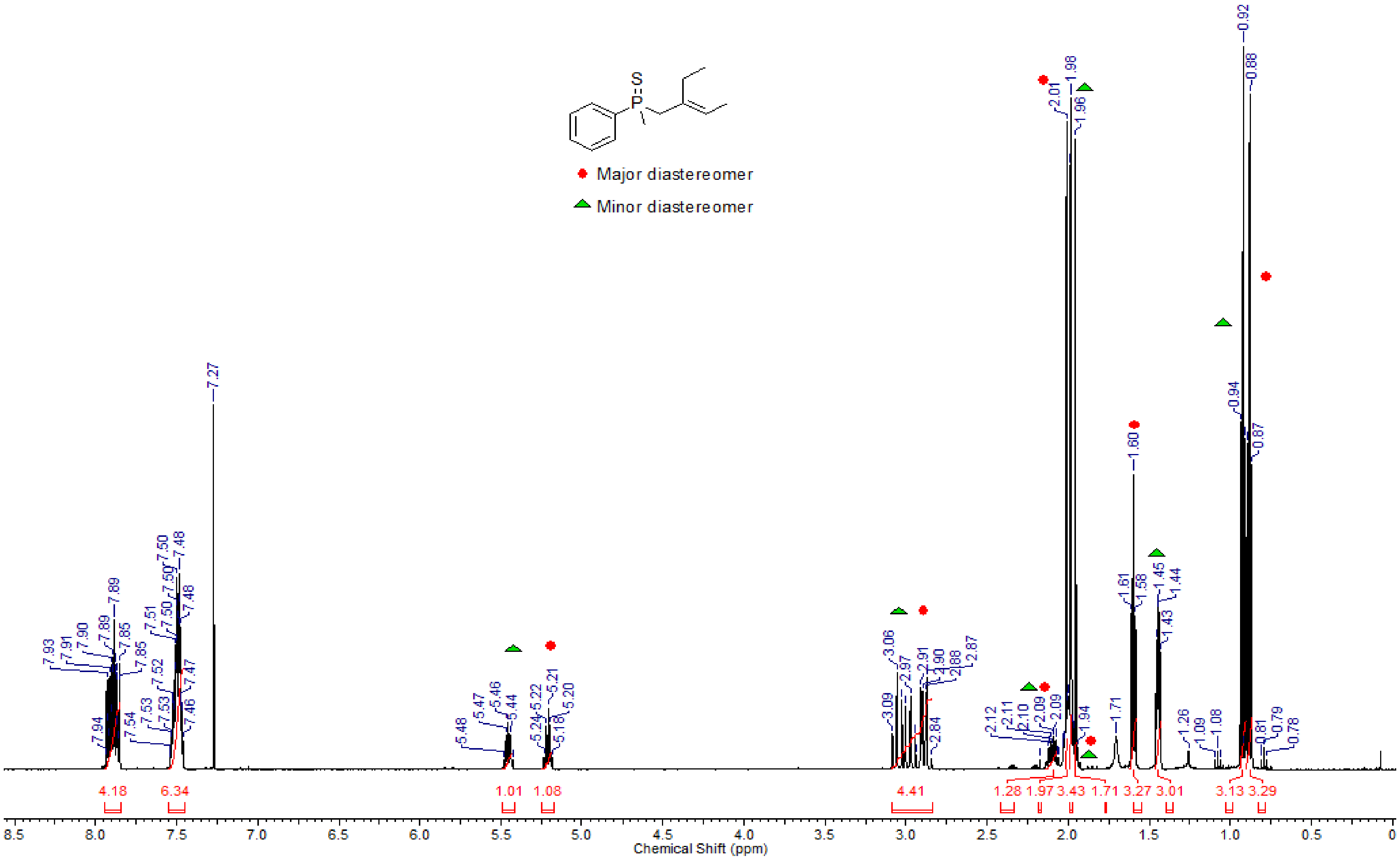

^{1}H NMR spectrum of (2-ethylbut-2-enyl)(methylphenyl)phosphine oxide (**58**) (CDCl3, 500 MHz).

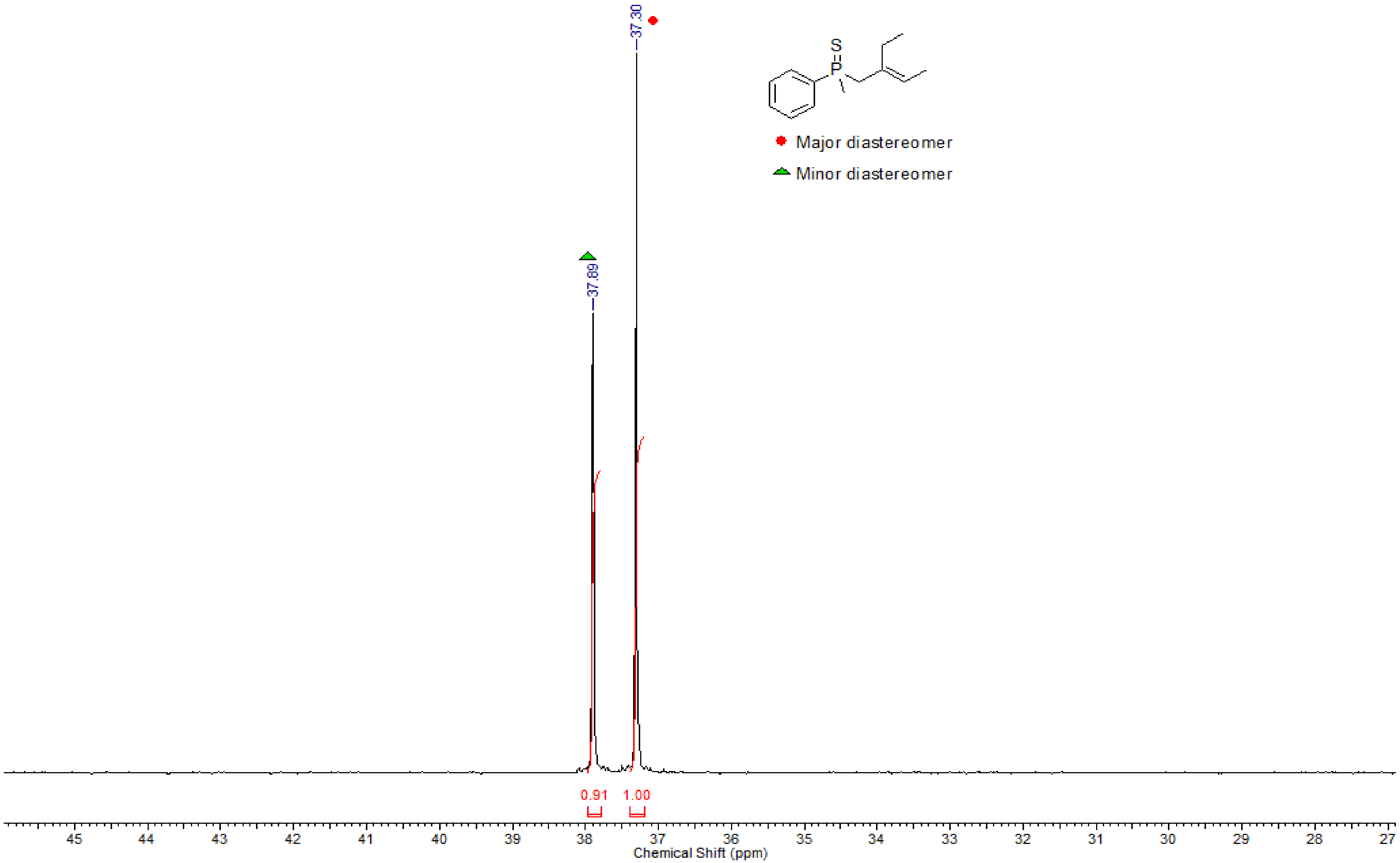

^{31}P NMR spectrum of (2-ethylbut-2-enyl)(methylphenyl)phosphine oxide (**58**) (CDCl3, 202 MHz).

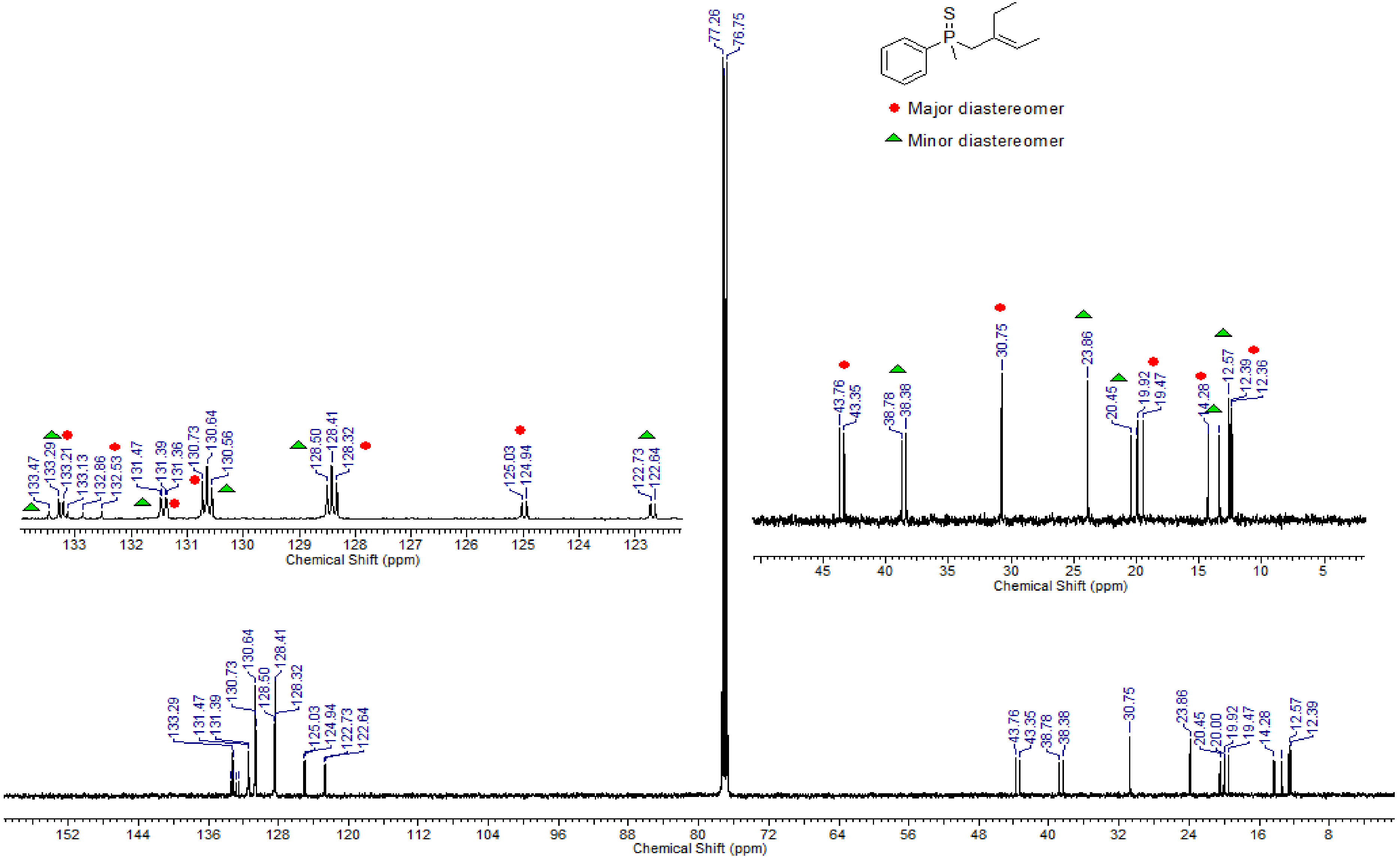

^{13}C NMR spectrum of (2-ethylbut-2-enyl)(methylphenyl)phosphine oxide (**58**) (CDCl3, 126 MHz).

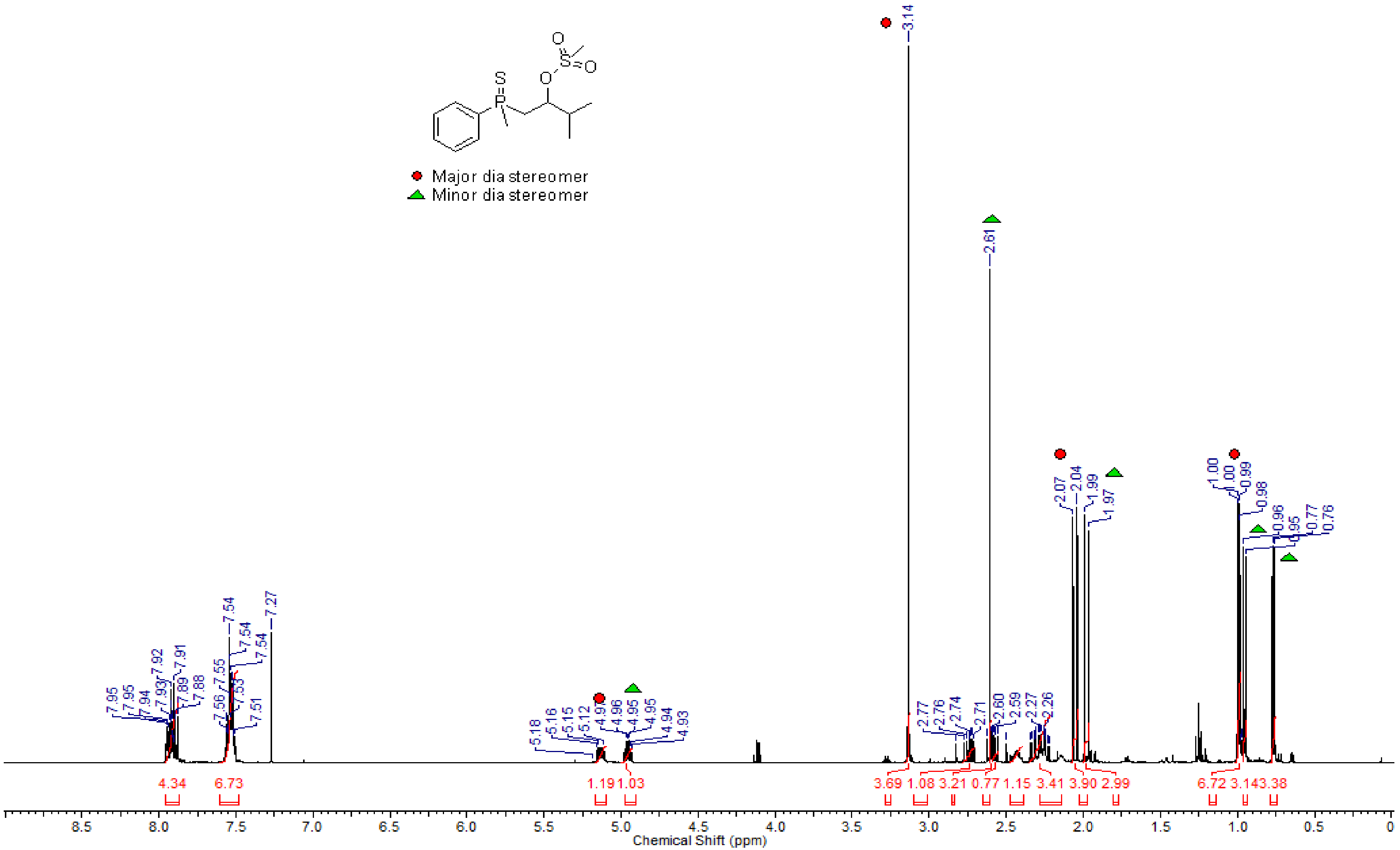

^{1}H NMR spectrum of (2- mesyloxy-3-methylbutyl)methylphenylphosphine sulfide (**59**) (CDCl3, 500 MHz).

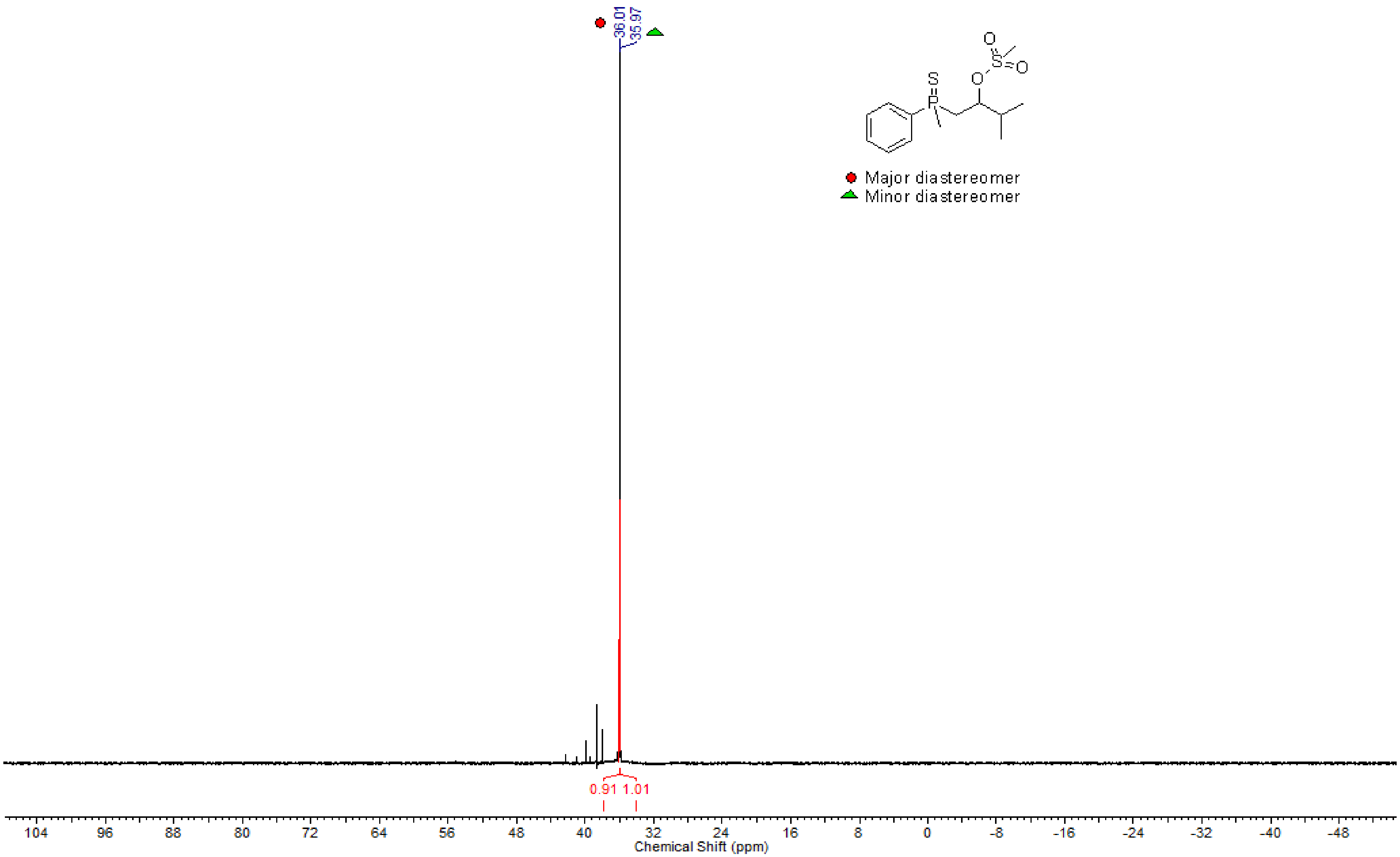

^{31}P NMR spectrum of (2- mesyloxy-3-methylbutyl)methylphenylphosphine sulfide (**59**) (CDCl3, 202 MHz).

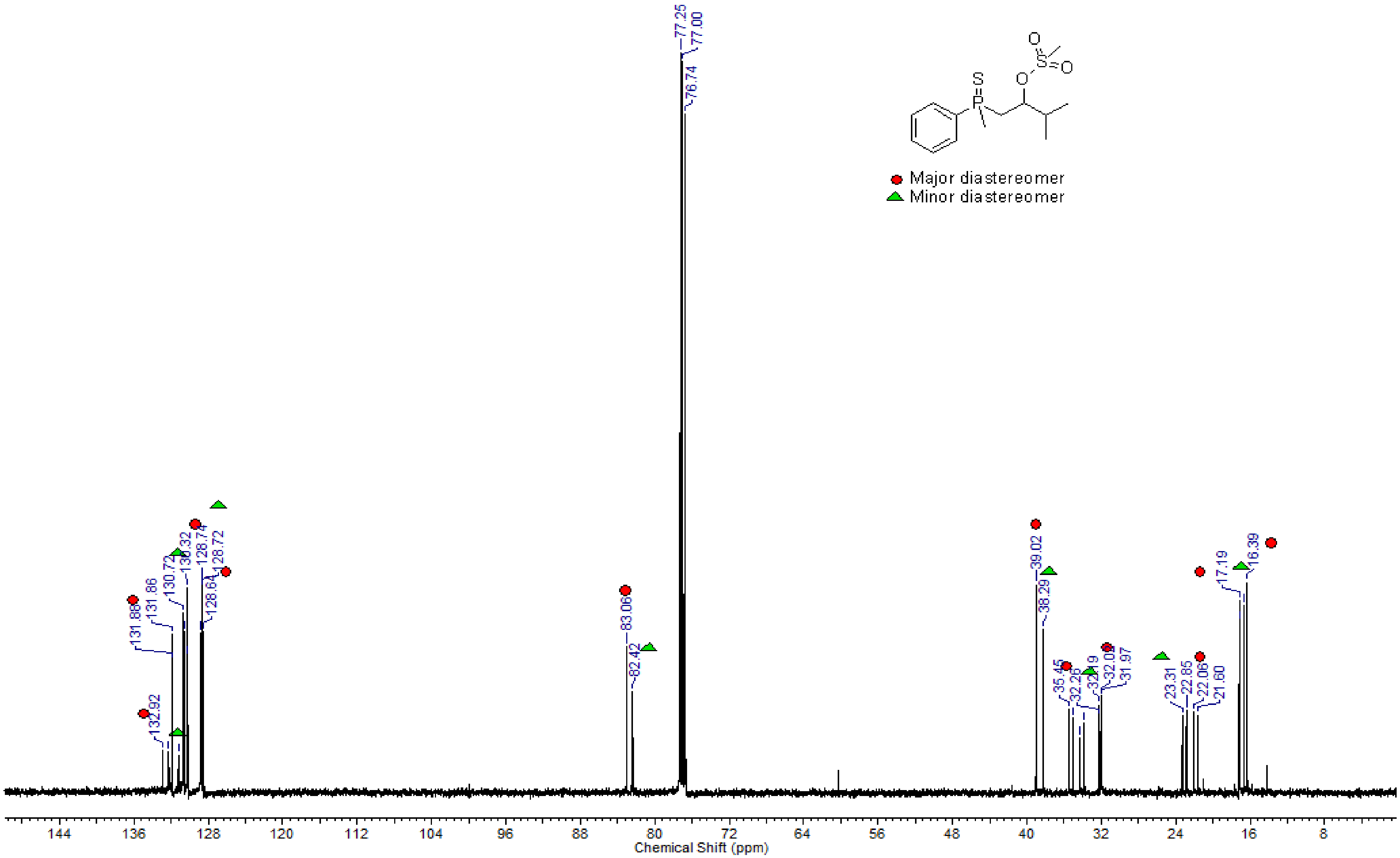

^{13}C NMR spectrum of (2- mesyloxy-3-methylbutyl)methylphenylphosphine sulfide (**59**) (CDCl3, 126 MHz).

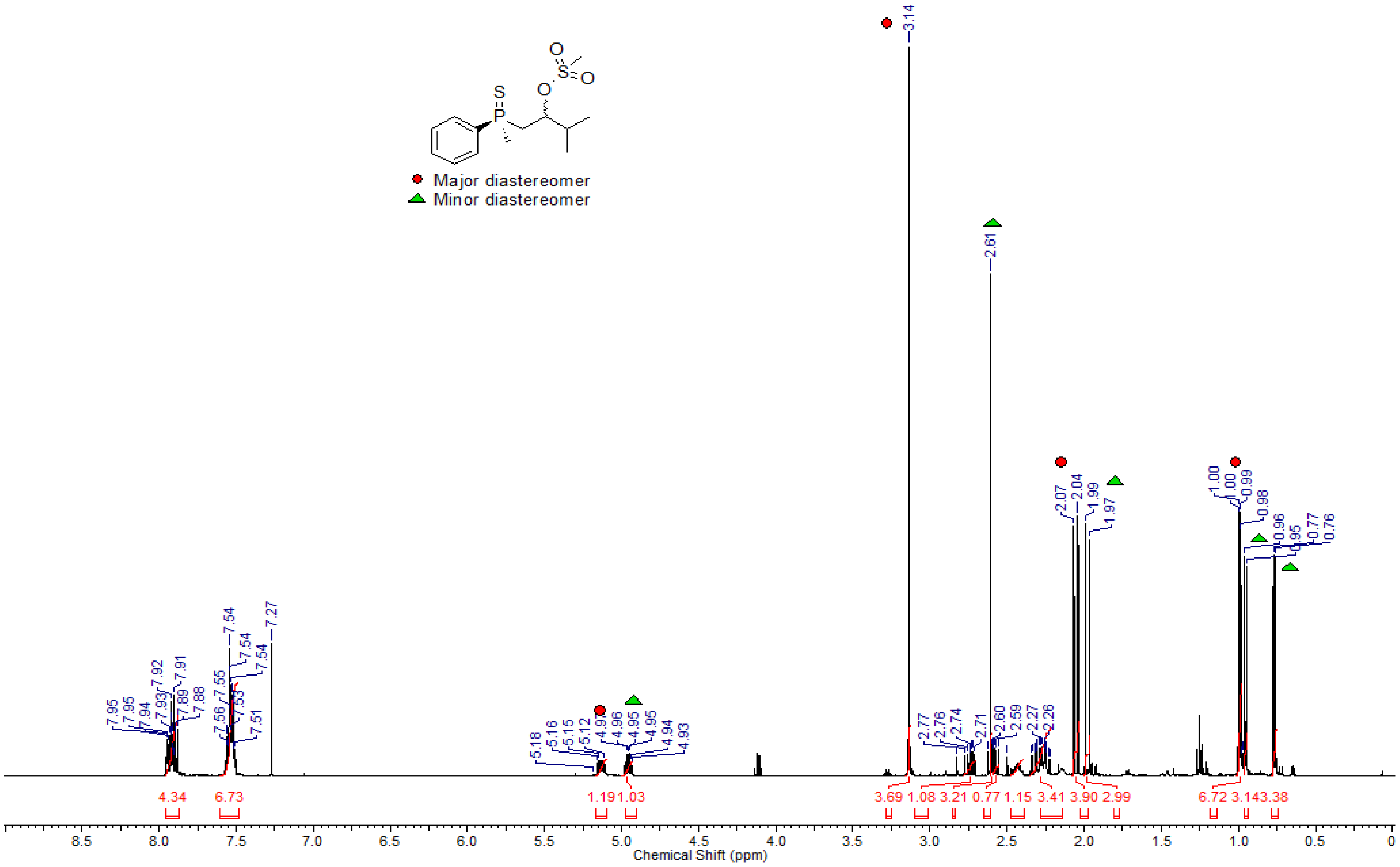

^{1}H NMR spectrum of (S_P)-(2- mesyloxy-3-methylbutyl)methylphenylphosphine sulfide (S_P)-(**59**) (CDCl3, 500 MHz).

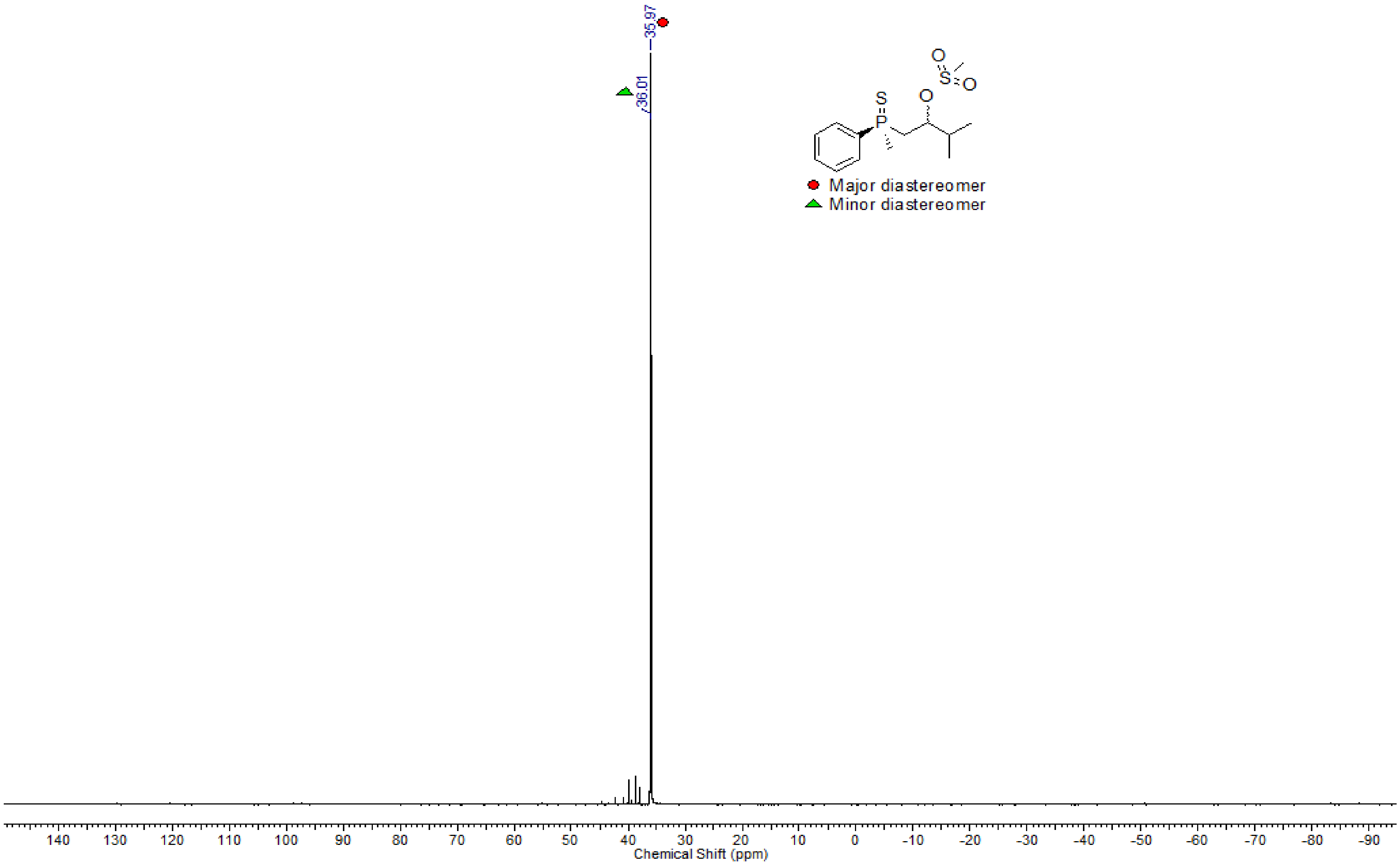

^{31}P NMR spectrum of (*S*P)-(2- mesyloxy-3-methylbutyl)methylphenylphosphine sulfide (*S*P)-(**59**) (CDCl3, 202 MHz).

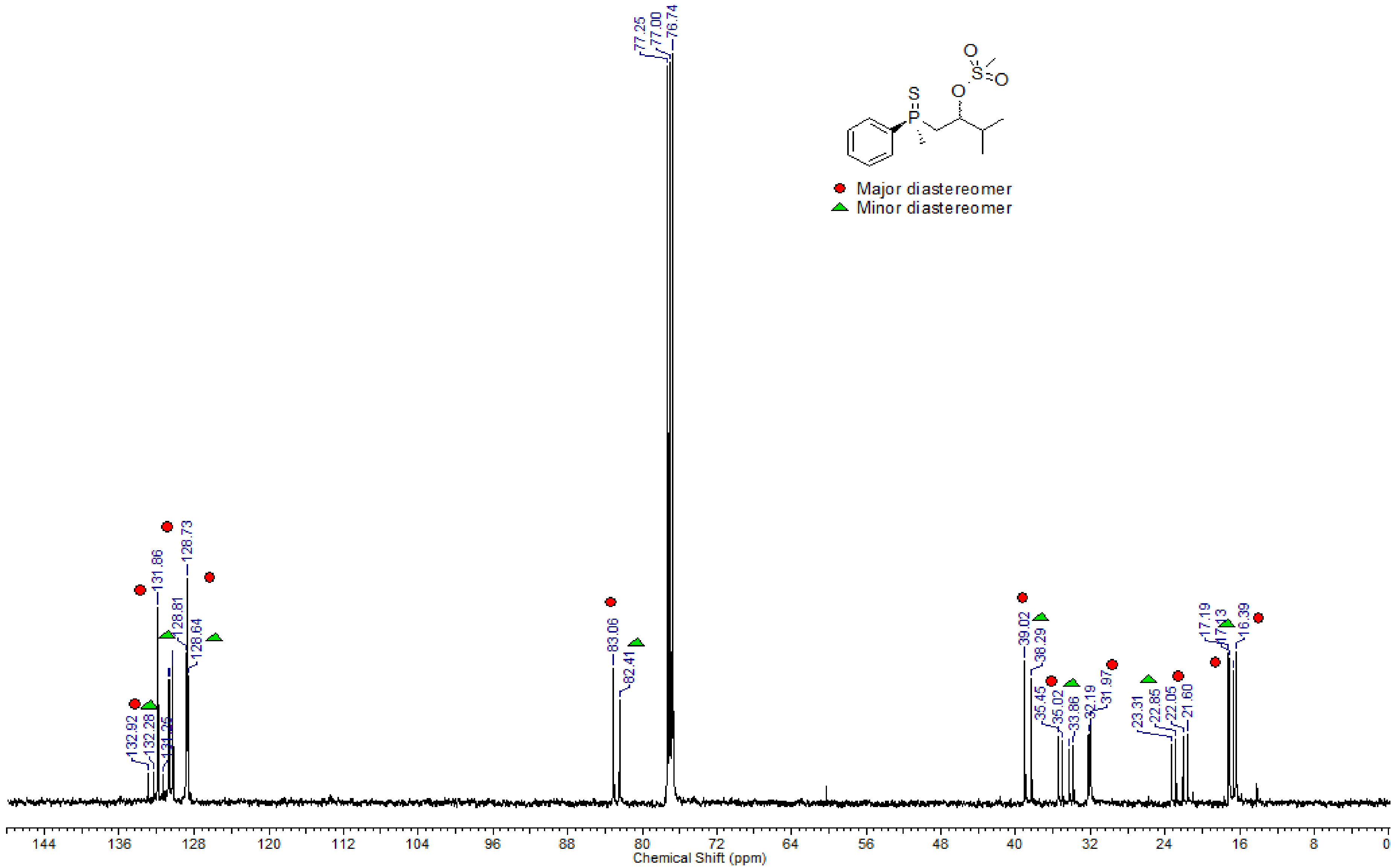

^{13}C NMR spectrum of (S_P)-(2- mesyloxy-3-methylbutyl)methylphenylphosphine sulfide (S_P)-(**59**) (CDCl3, 126 MHz).

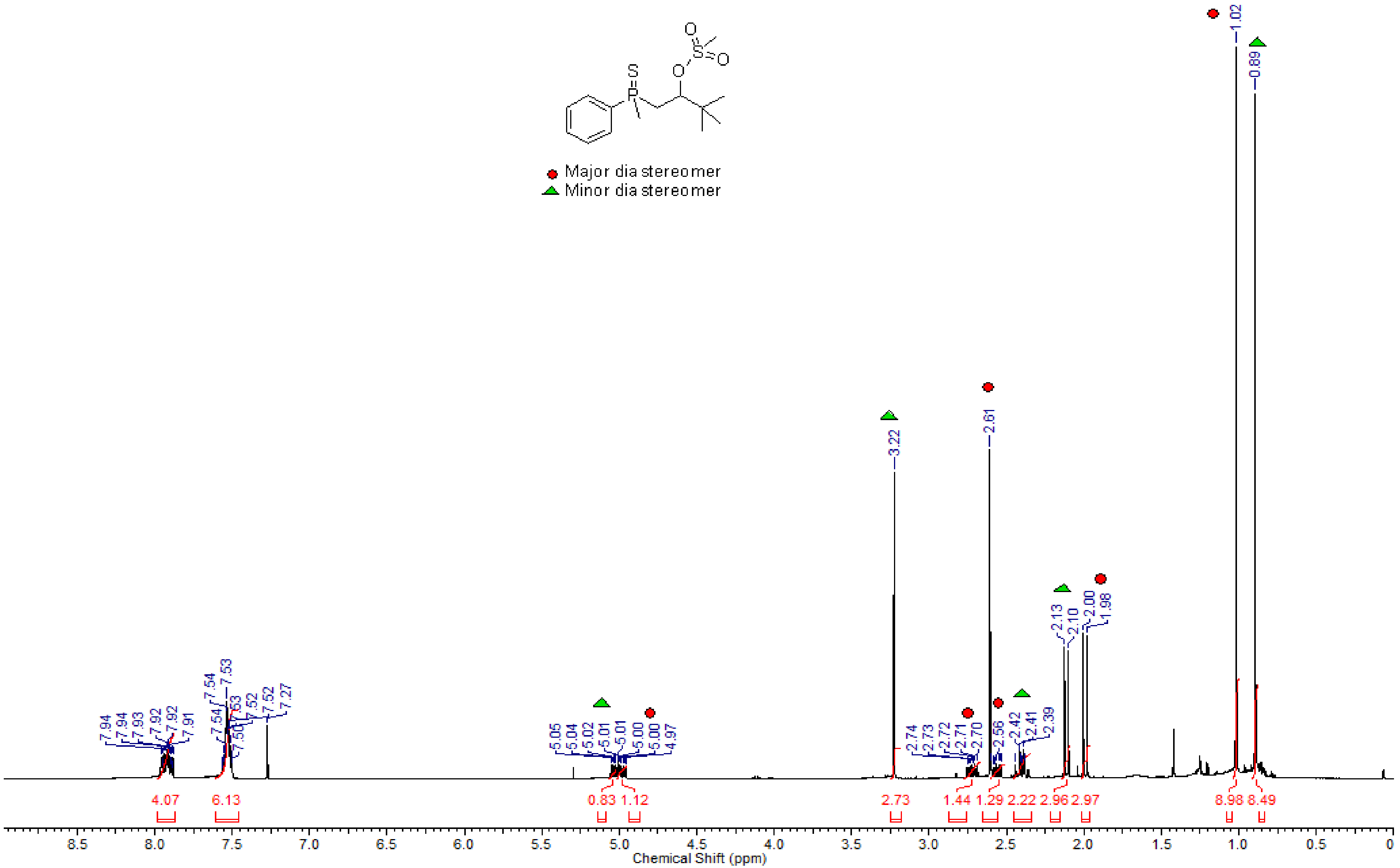

^{1}H NMR spectrum of (2- mesyloxy-3,3-dimethylbutyl)methylphenylphosphine sulfide (**60**) (CDCl3, 500 MHz).

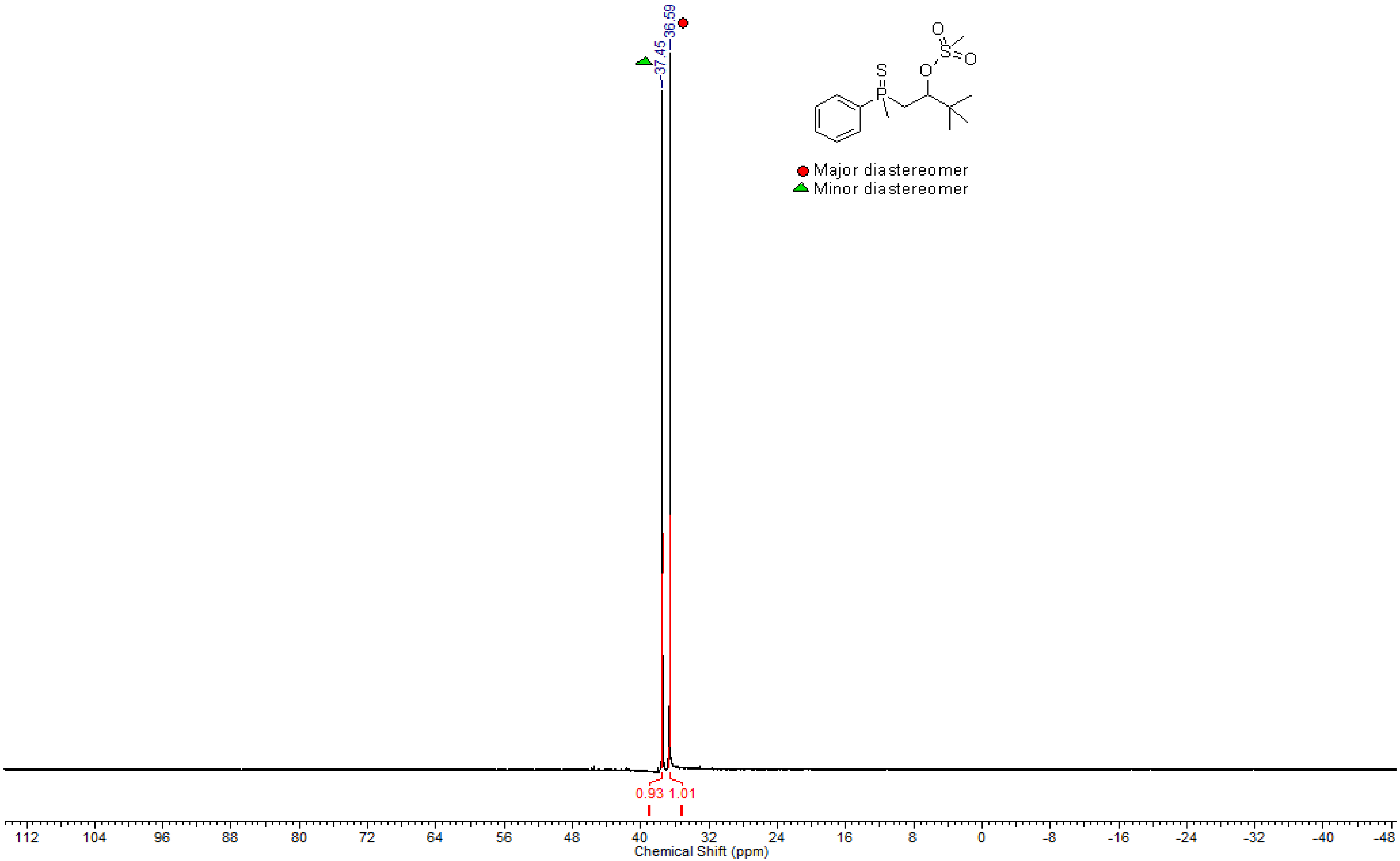

^{31}P NMR spectrum of (2- mesyloxy-3,3-dimethylbutyl)methylphenylphosphine sulfide (**60**) ($CDCl_3$, 202 MHz).

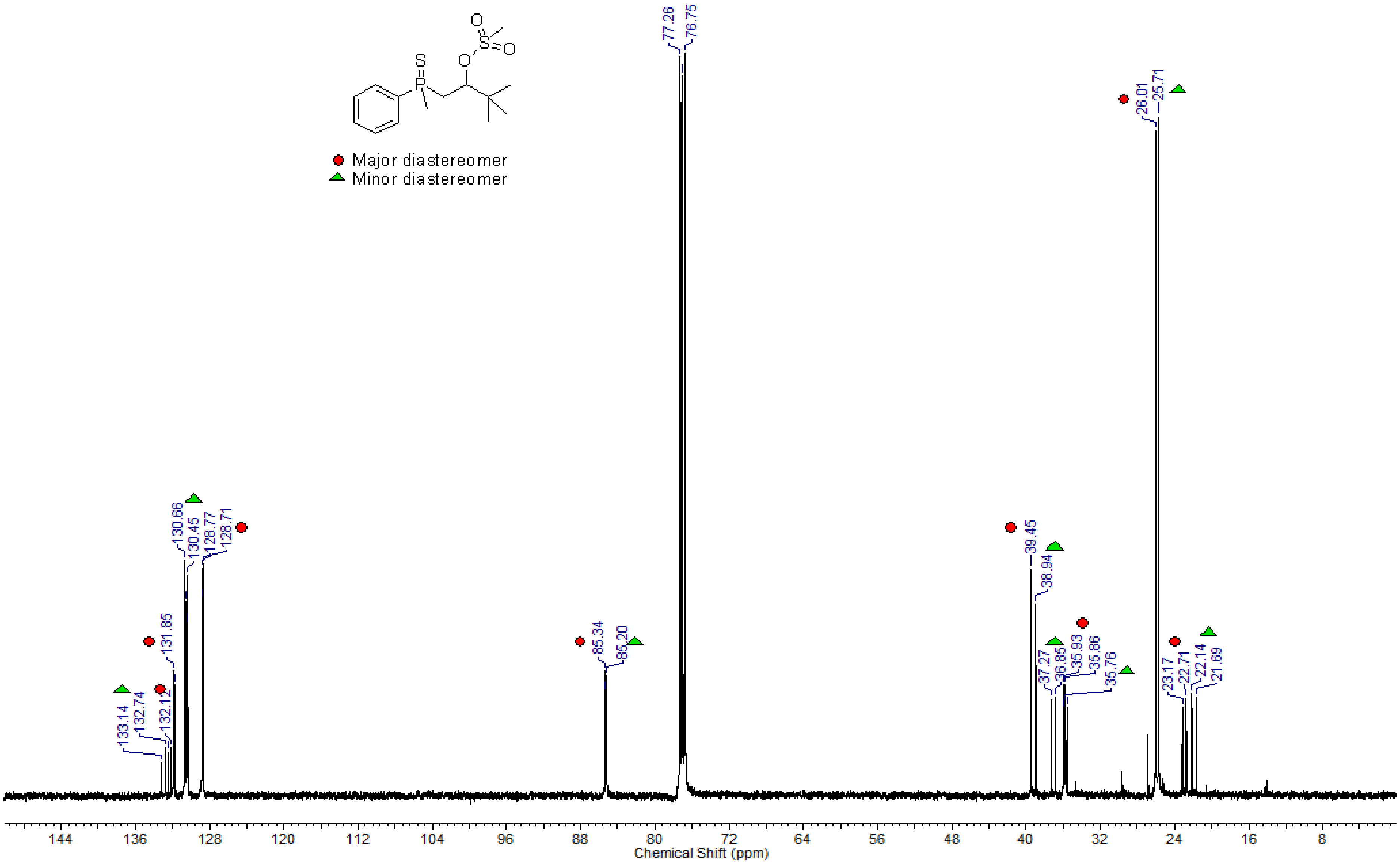

^{13}C NMR spectrum of (2- mesyloxy-3,3-dimethylbutyl)methylphenylphosphine sulfide (**60**) (CDCl3, 126 MHz).

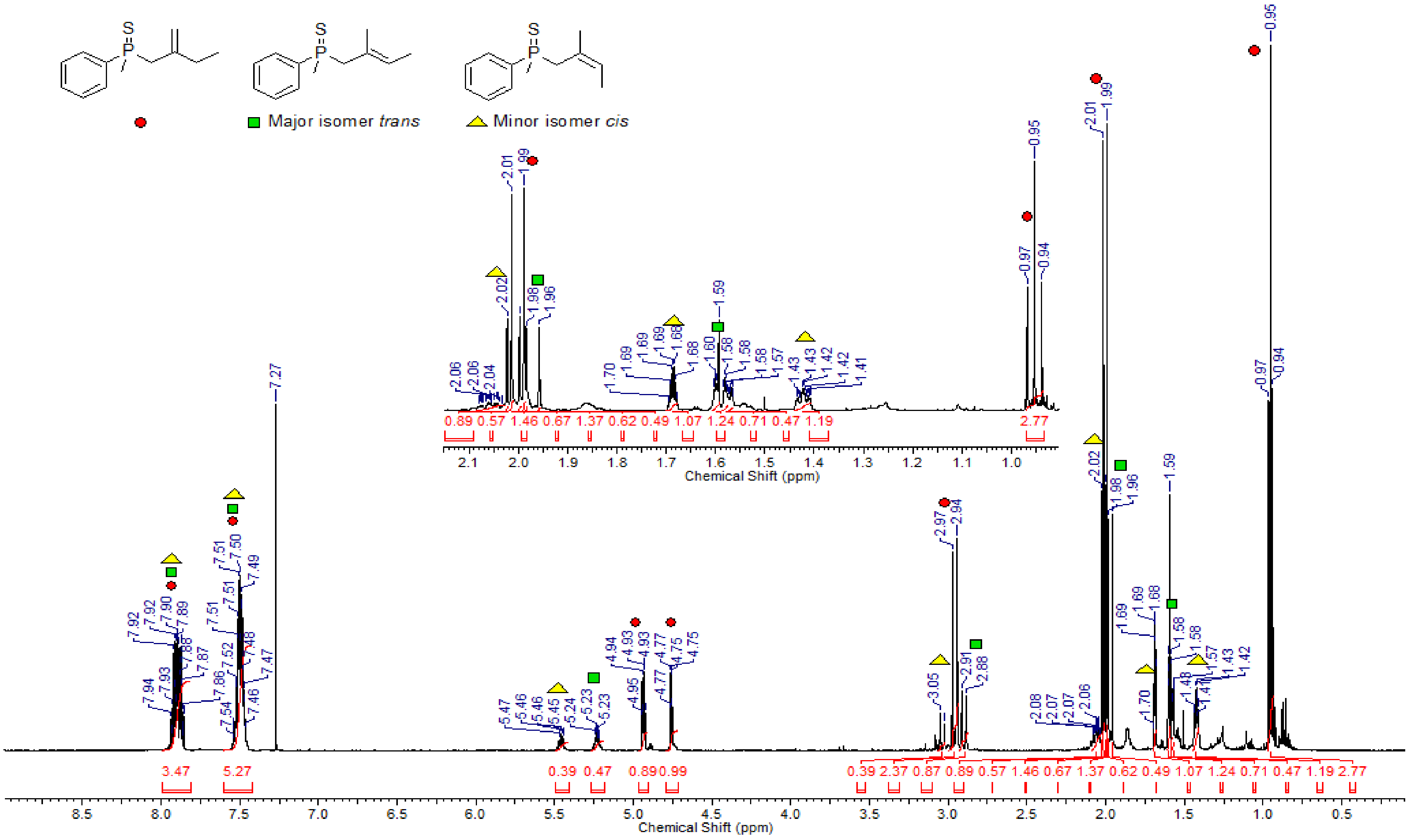

^{1}H NMR spectrum of mixture of (2-methylbut-2-enyl)(methylphenyl)phosphine oxide (**61**) and (2-ethylprop-2-enyl)(methylphenyl)phosphine oxide (**62**) (CDCl3, 500 MHz).

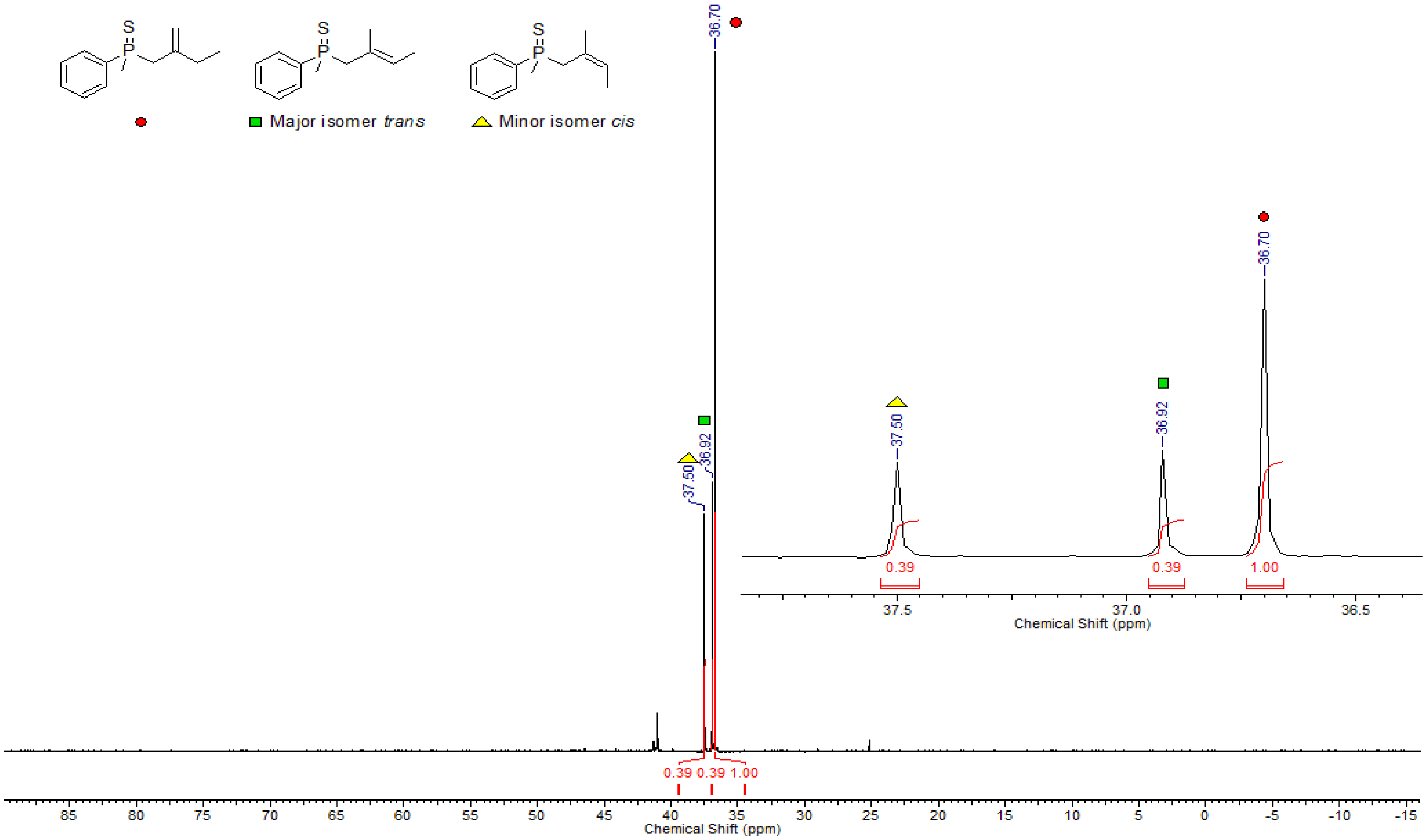

^{31}P NMR spectrum of mixture of (2-methylbut-2-enyl)(methylphenyl)phosphine oxide (**61**) and (2-ethylprop-2-enyl)(methylphenyl)phosphine oxide (**62**) (CDCl3, 202 MHz).

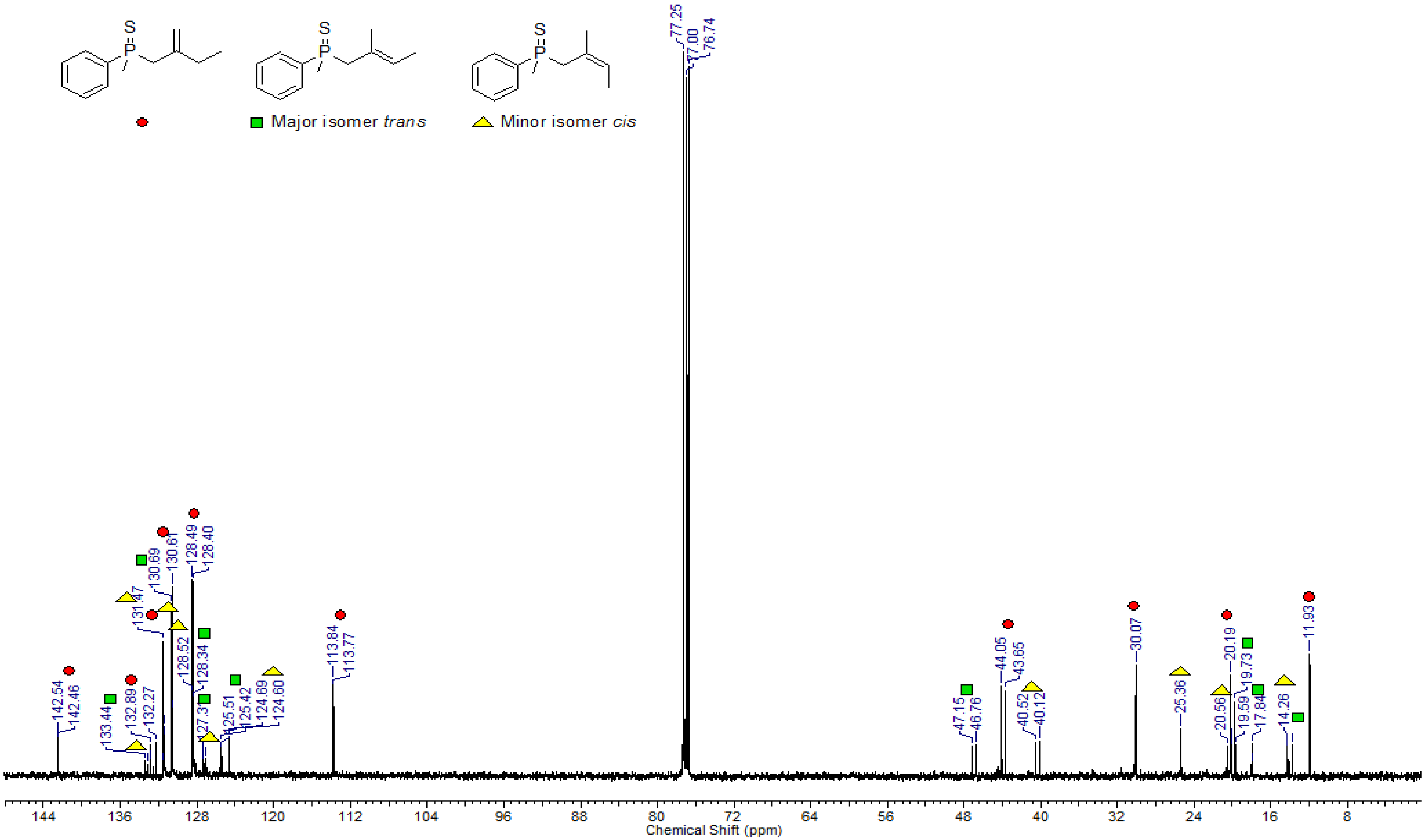

^{13}C NMR spectrum of mixture of (2-methylbut-2-enyl)(methylphenyl)phosphine oxide (**61**) and (2-ethylprop-2-enyl)(methylphenyl)phosphine oxide (**62**) (CDCl3, 126 MHz).

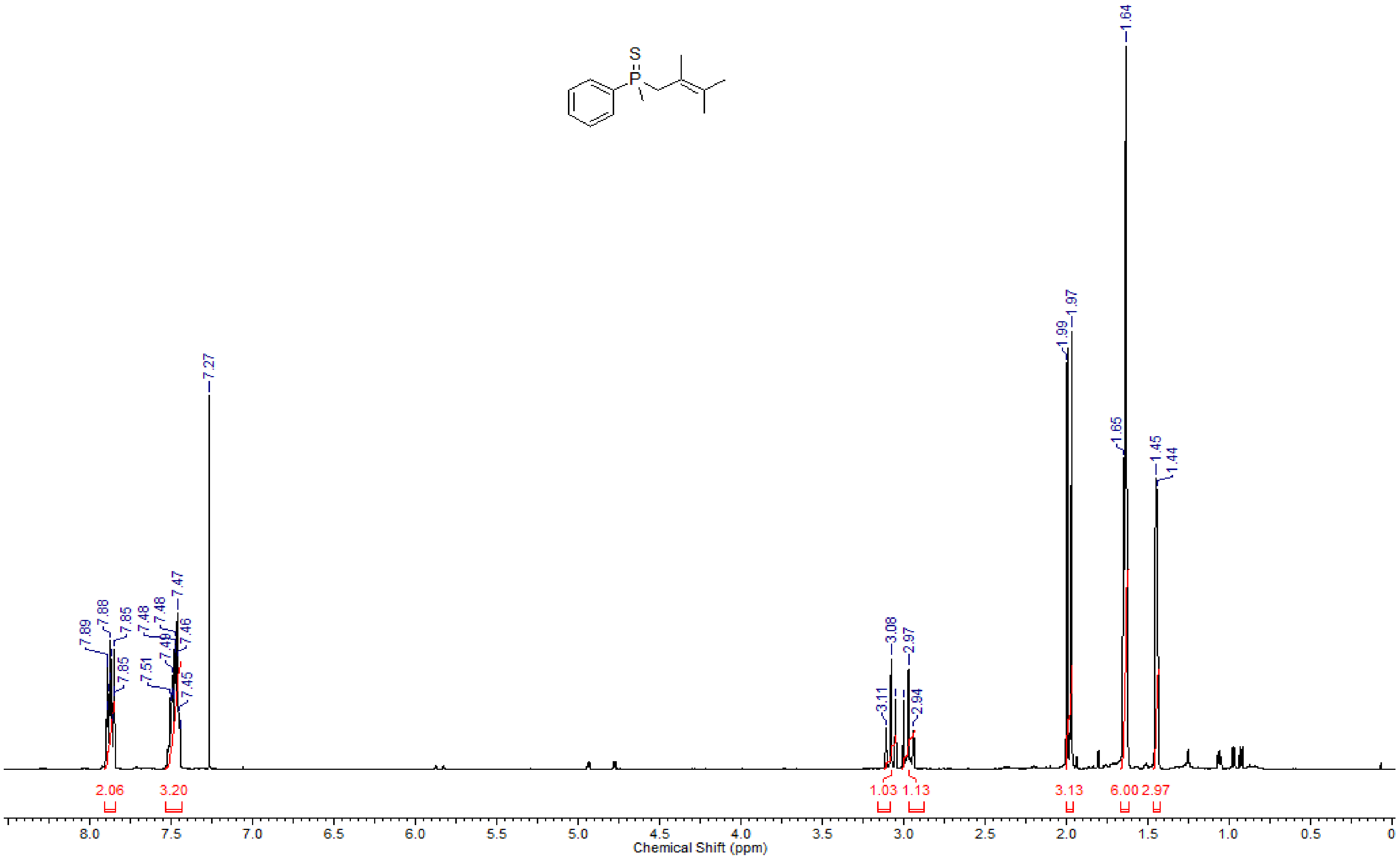

^{1}H NMR spectrum of (2,3-dimethylbut-2-enyl)(methylphenyl)phosphine oxide (**63**) (CDCl3, 500 MHz).

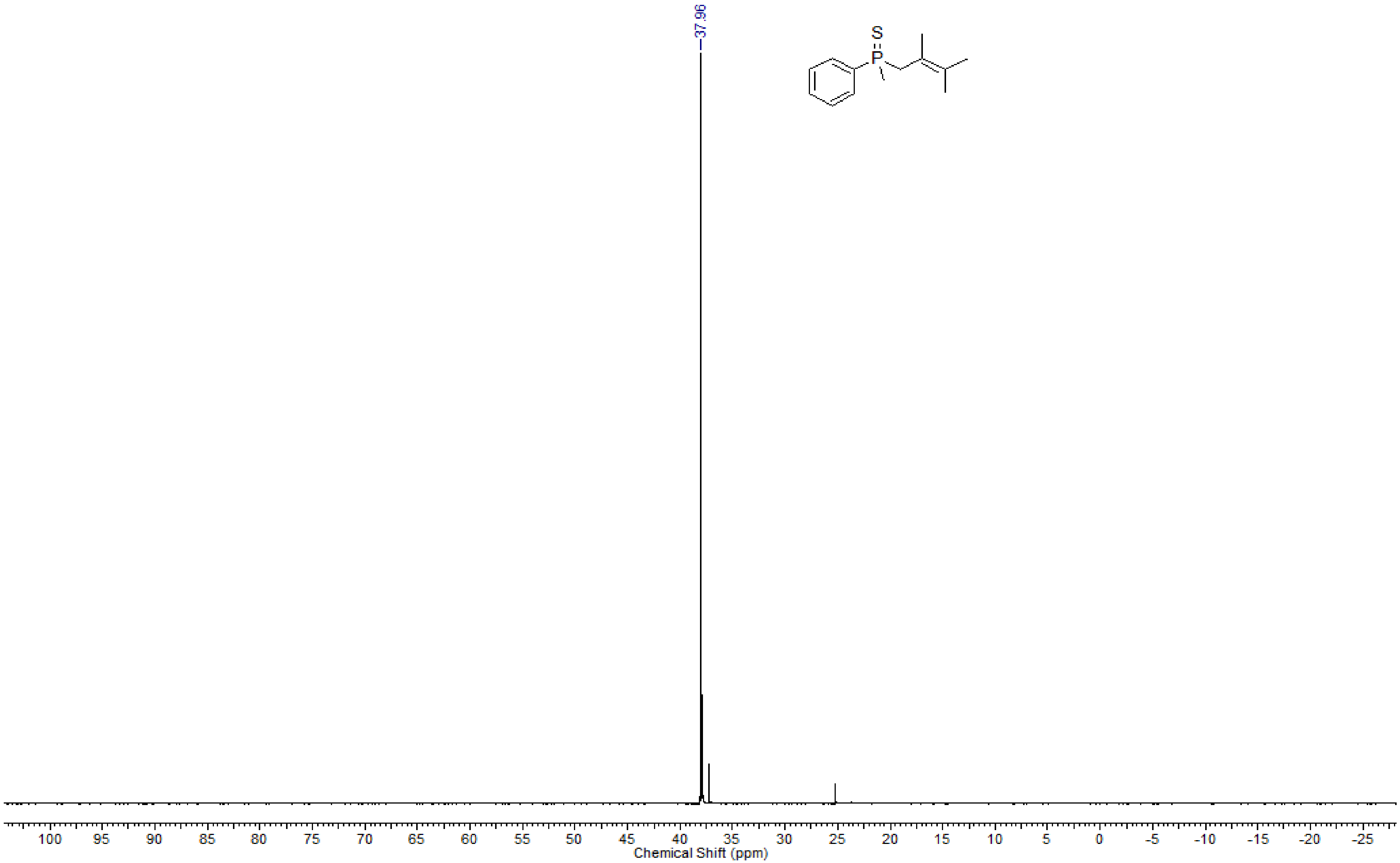

^{31}P NMR spectrum of (2,3-dimethylbut-2-enyl)(methylphenyl)phosphine oxide (**63**) (CDCl3, 202 MHz).

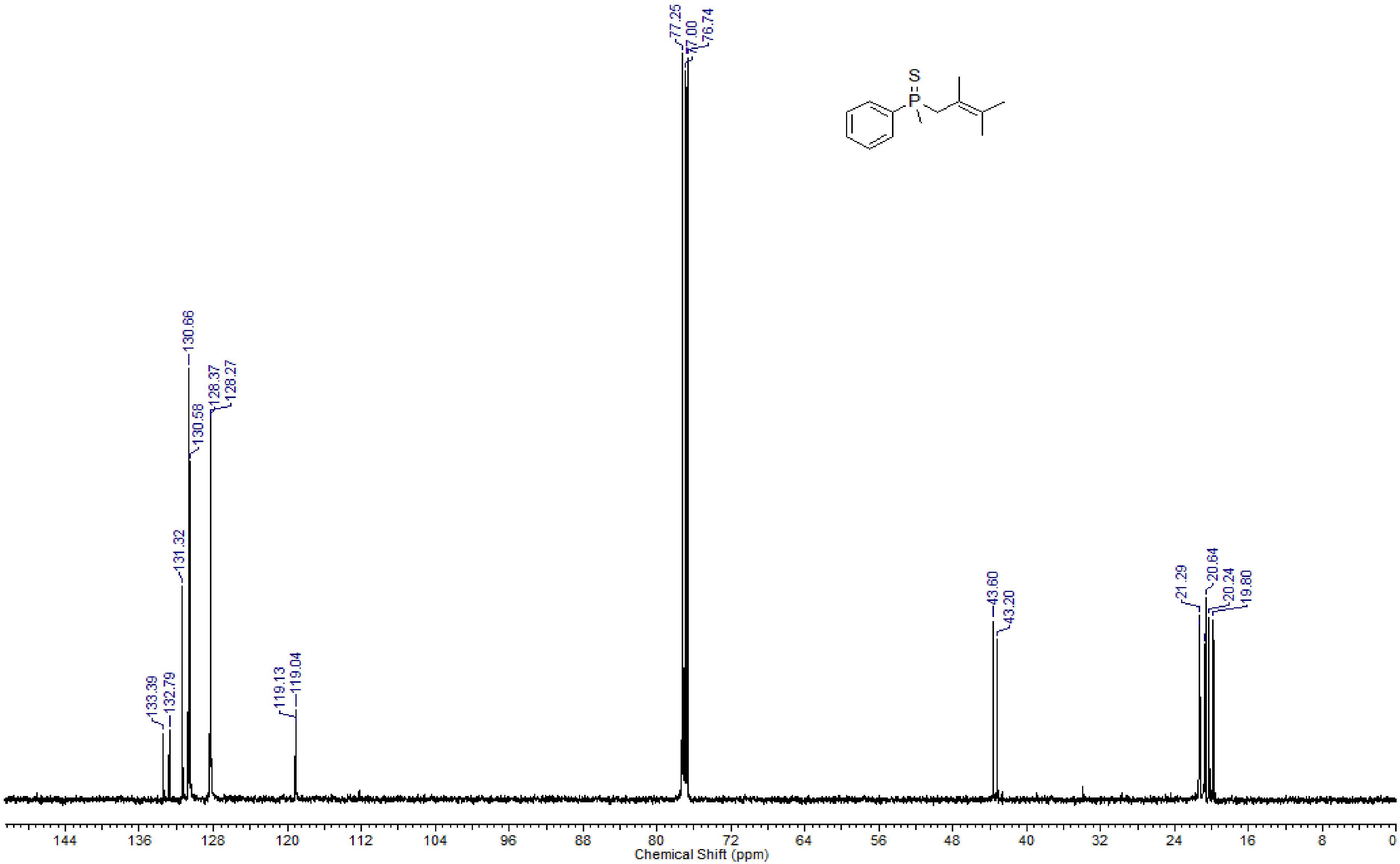

^{13}C NMR spectrum of (2,3-dimethylbut-2-enyl)(methylphenyl)phosphine oxide (**63**) (CDCl3, 126 MHz).

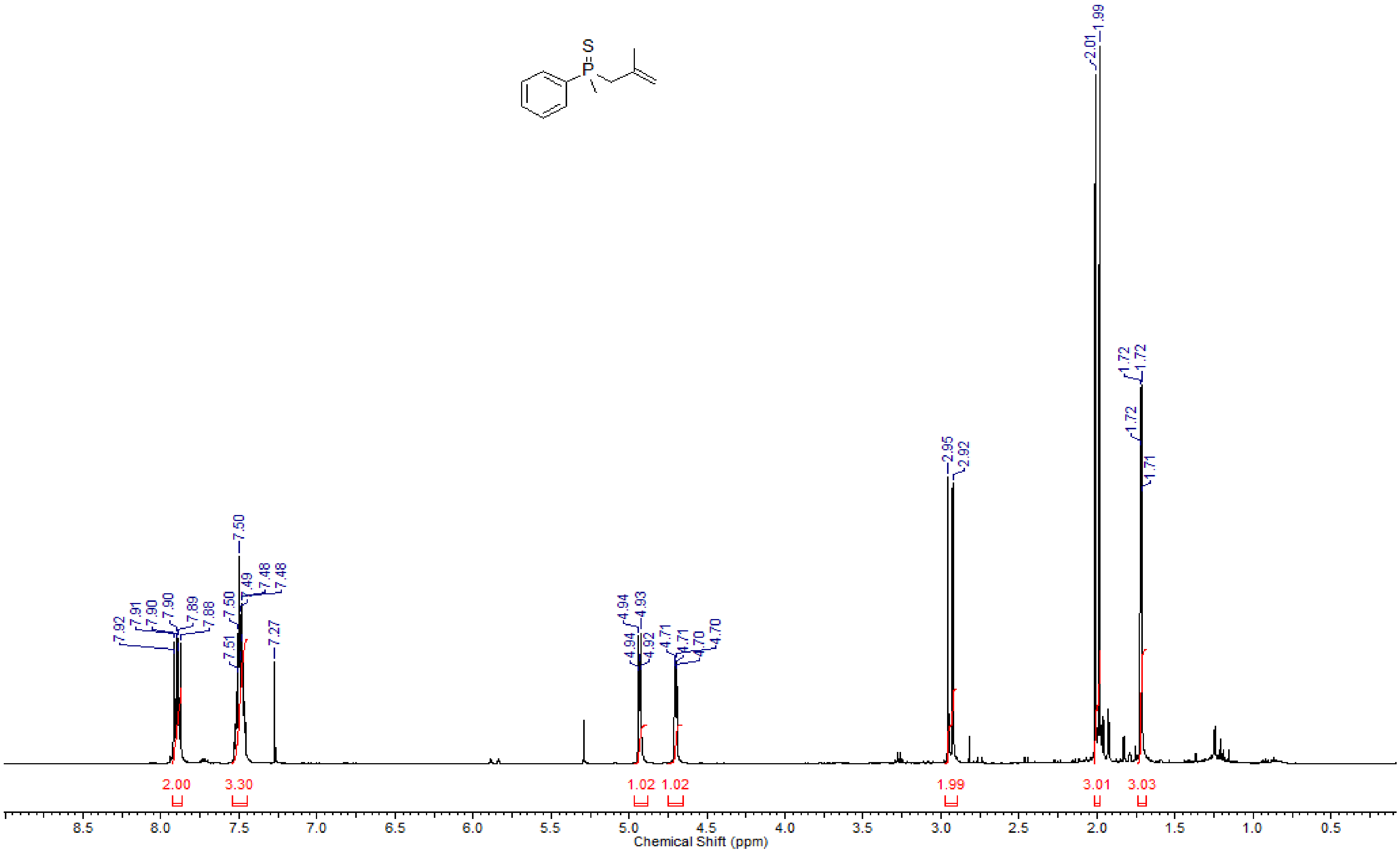

^{1}H NMR spectrum of (2-methylprop-2-enyl)(methylphenyl)phosphine sulfide (**64**) (CDCl3, 500 MHz).

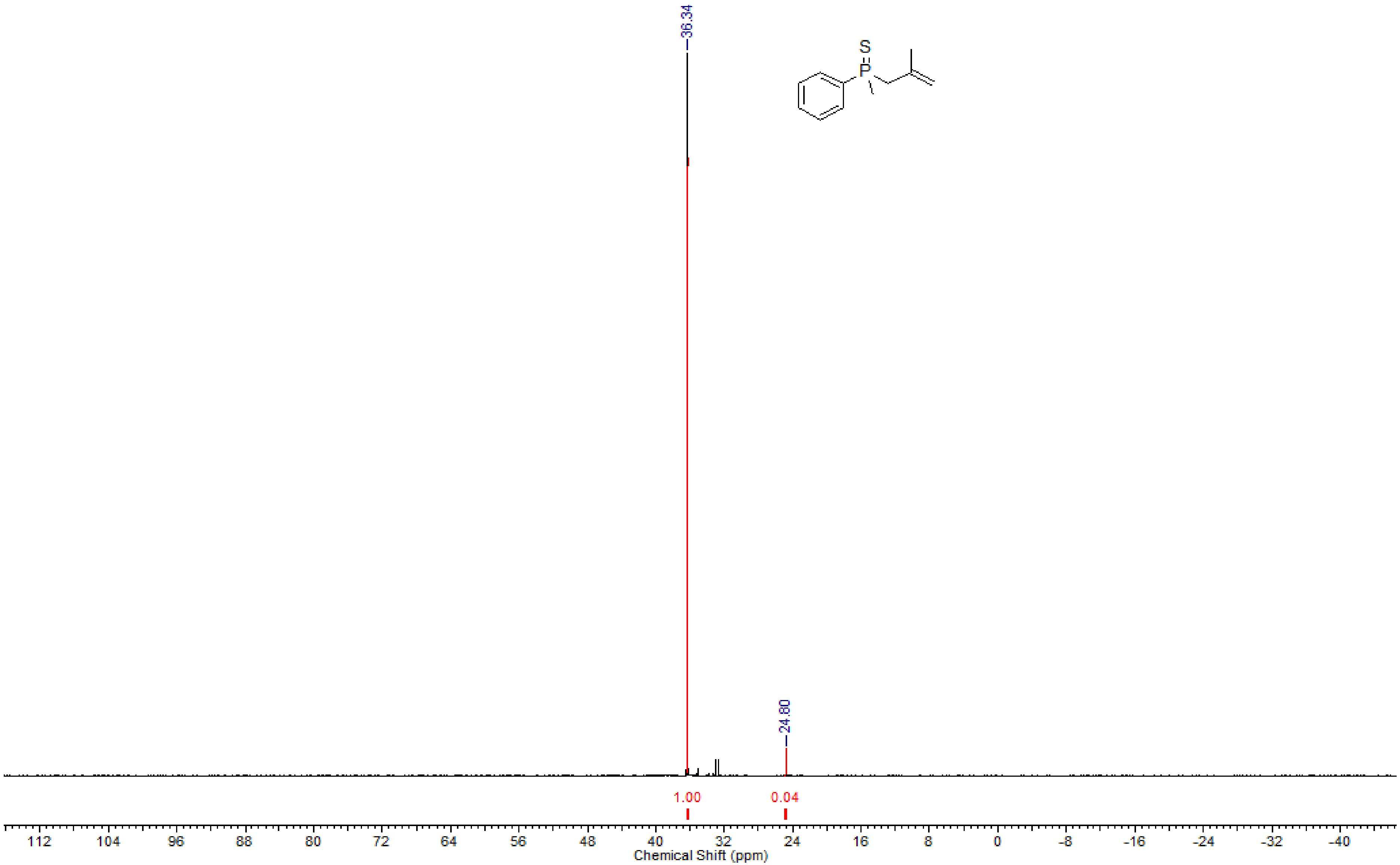

^{31}P NMR spectrum of (2-methylprop-2-enyl)(methylphenyl)phosphine sulfide (**64**) (CDCl3, 202 MHz).

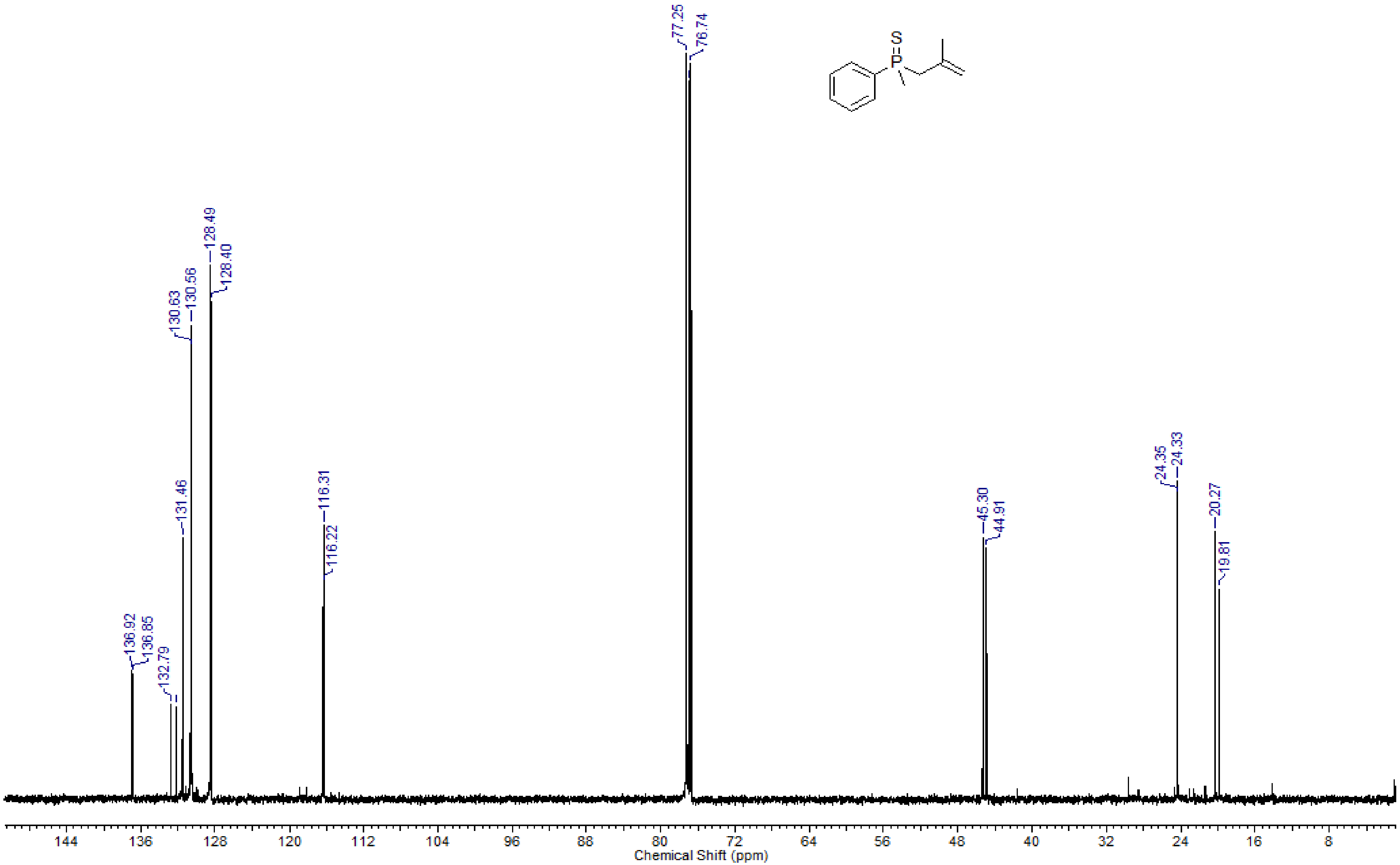

^{13}C NMR spectrum of (2-methylprop-2-enyl)(methylphenyl)phosphine sulfide (**64**) (CDCl3, 126 MHz).

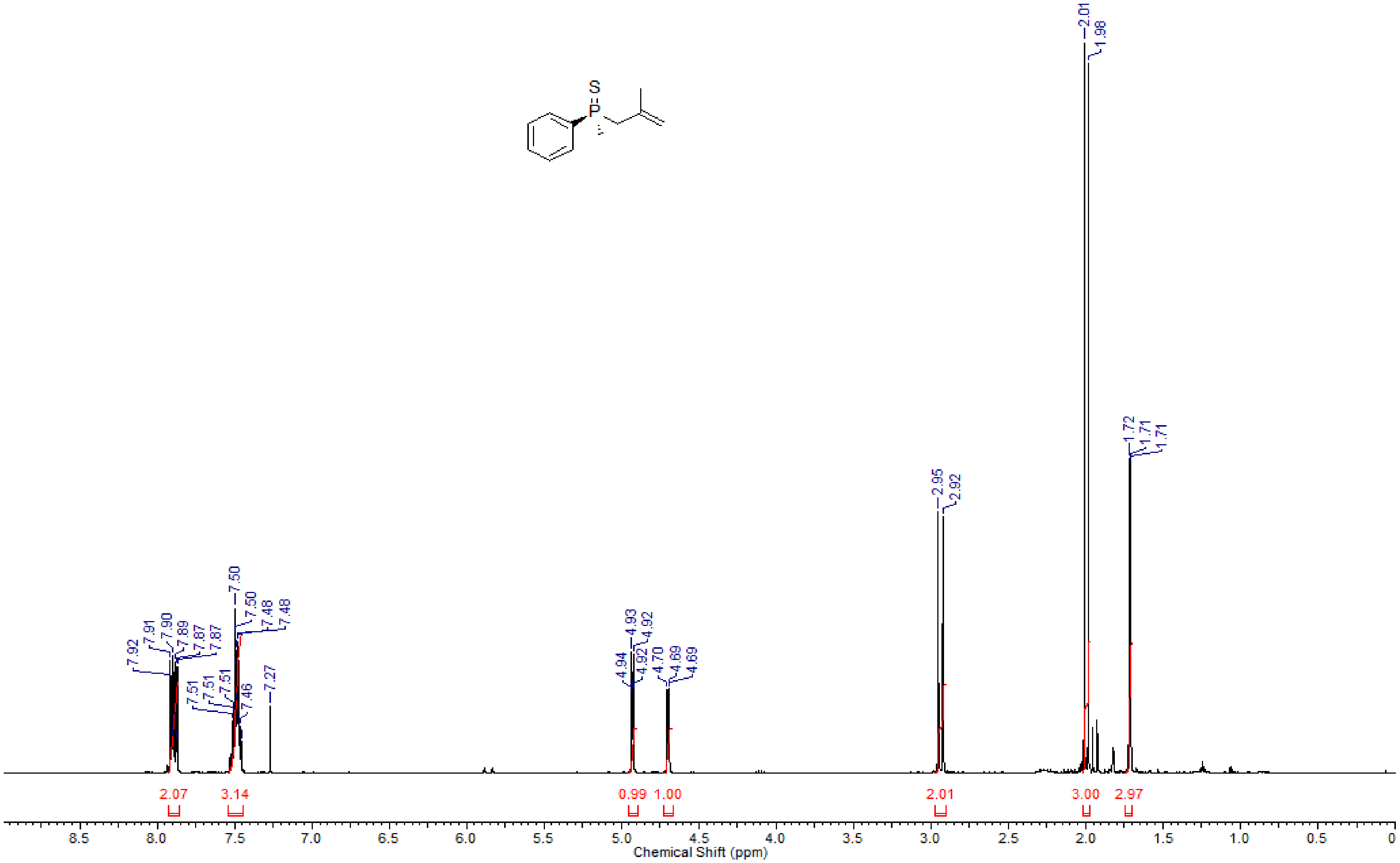

^{1}H NMR spectrum of (S_P)-(2-methylprop-2-enyl)(methylphenyl)phosphine oxide (S_P)-(**64**) (CDCl3, 500 MHz).

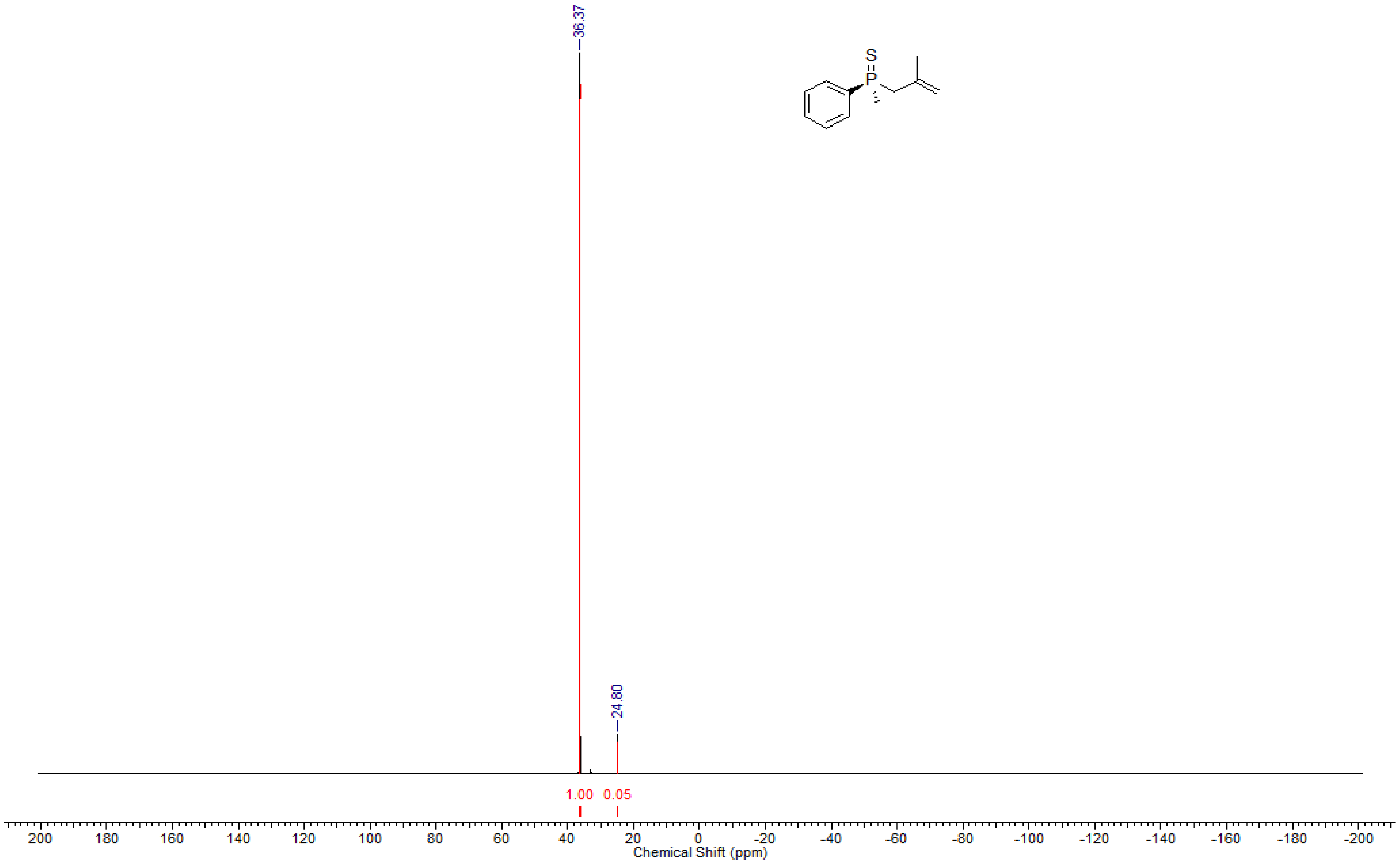

^{31}P NMR spectrum of (S_P)-(2-methylprop-2-enyl)(methylphenyl)phosphine oxide (S_P)-(**64**) (CDCl3, 202 MHz).

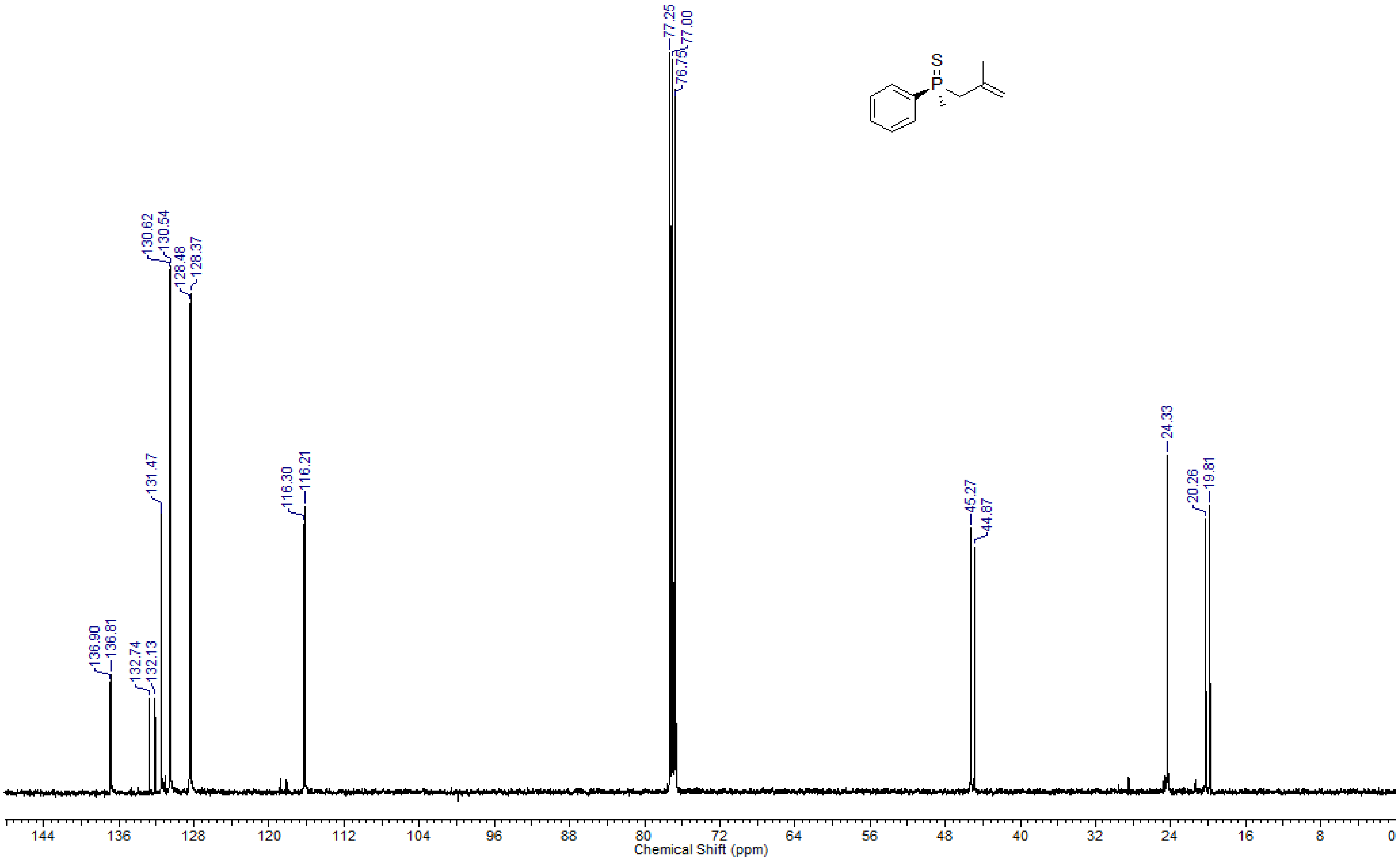

^{13}C NMR spectrum of (S_{P})-(2-methylprop-2-enyl)(methylphenyl)phosphine oxide (S_{P})-(**64**) (CDCl3, 126 MHz).
